# Stereodivergent Csp^3^–Csp^3^ bond construction *via* Lewis base/iridium cooperative catalysis: a modular synthesis of tertiary fluoride-containing stereodiads

**DOI:** 10.1039/d6sc06429a

**Published:** 2026-10-21

**Authors:** Smita Mandal, Maren Pink, Thomas N. Snaddon

**Affiliations:** a Indiana University, Department of Chemistry 800 E. Kirkwood Ave. Bloomington IN 47405 USA tsnaddon@iu.edu https://snaddon.lab.indiana.edu

## Abstract

The synthesis of tertiary fluoride-containing stereogenic centers remains a significant challenge. This challenge is amplified when such units are embedded within vicinal stereocenters arrays, where both absolute and relative stereochemistry must be controlled. Here we disclose a stereodivergent Lewis base–iridium cooperative catalysis method that unites α-fluorinated C1-ammonium enolate and π(allyl)Ir intermediates to construct tertiary fluoride-containing stereodiads. Each catalyst is independently responsible for the configurational outcome of each stereocenter comprising the stereodiad during Csp^3^–Csp^3^ bond construction. The conversion of easily prepared racemic α-fluoro esters to single α-fluoro C1-ammonium enolates *via* Lewis base catalysis is noteworthy.

## Abstract 2

The synthesis of tertiary fluoride-containing stereogenic centers remains a significant challenge.

## Graphical abstract

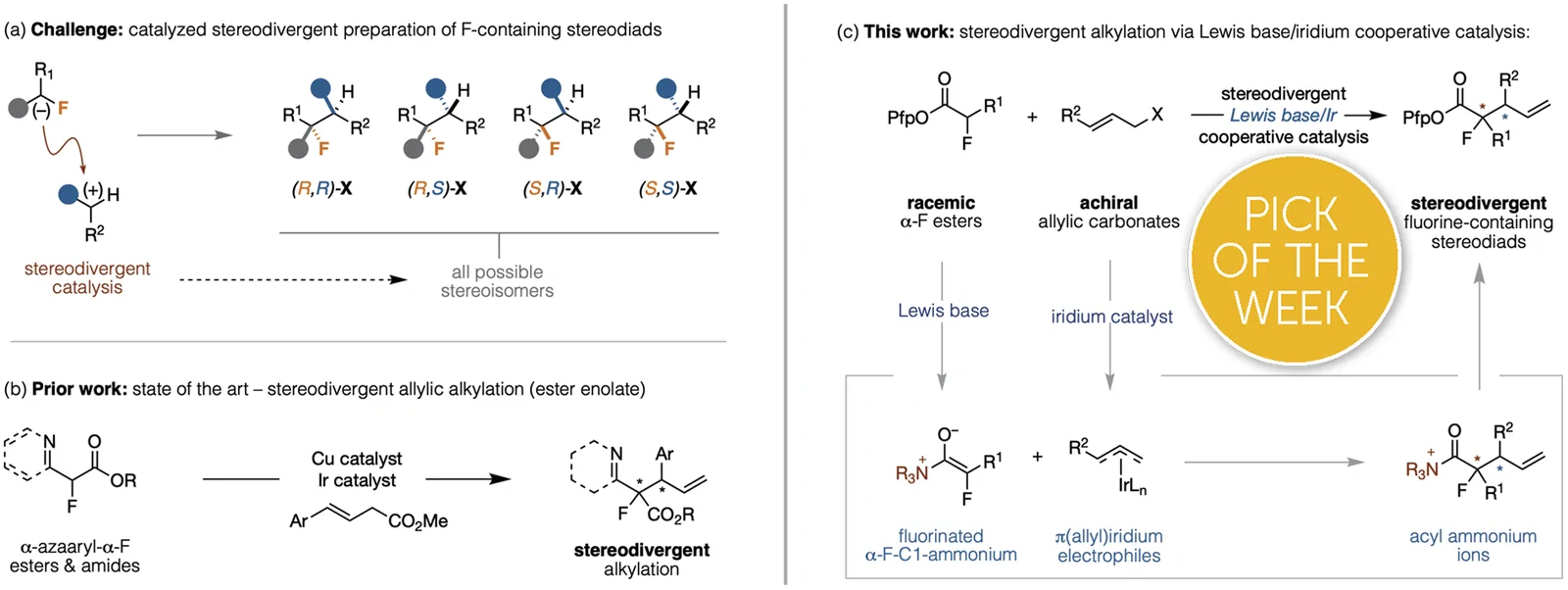

## Introduction

Cooperative catalysis is an enabling and expansive design framework that has the potential to control all aspects of bond formation.^1,2^ Within this nascent area our laboratory has leveraged Lewis base-transition metal cooperativity to develop a suite of enantioselective alkylation reactions where pertinent aspects of reactivity and selectivity are regulated.^3–5^ Our most recent focus has concerned stereodivergent catalysis, where the selective preparation of all possible stereoisomers of vicinal stereodiads is possible *via* convergent bond formation.^4,5^ Such an approach provides an effective and expansive conceptual blueprint to rapidly explore 3D chemical space and its implications for structure–function relationships.^6^ This led us quite naturally to fully substituted fluorinated stereocenters—and the specific challenge of tertiary fluoride-embedded stereodiad preparation (Scheme 1a).^7,8^

**Scheme 1:**
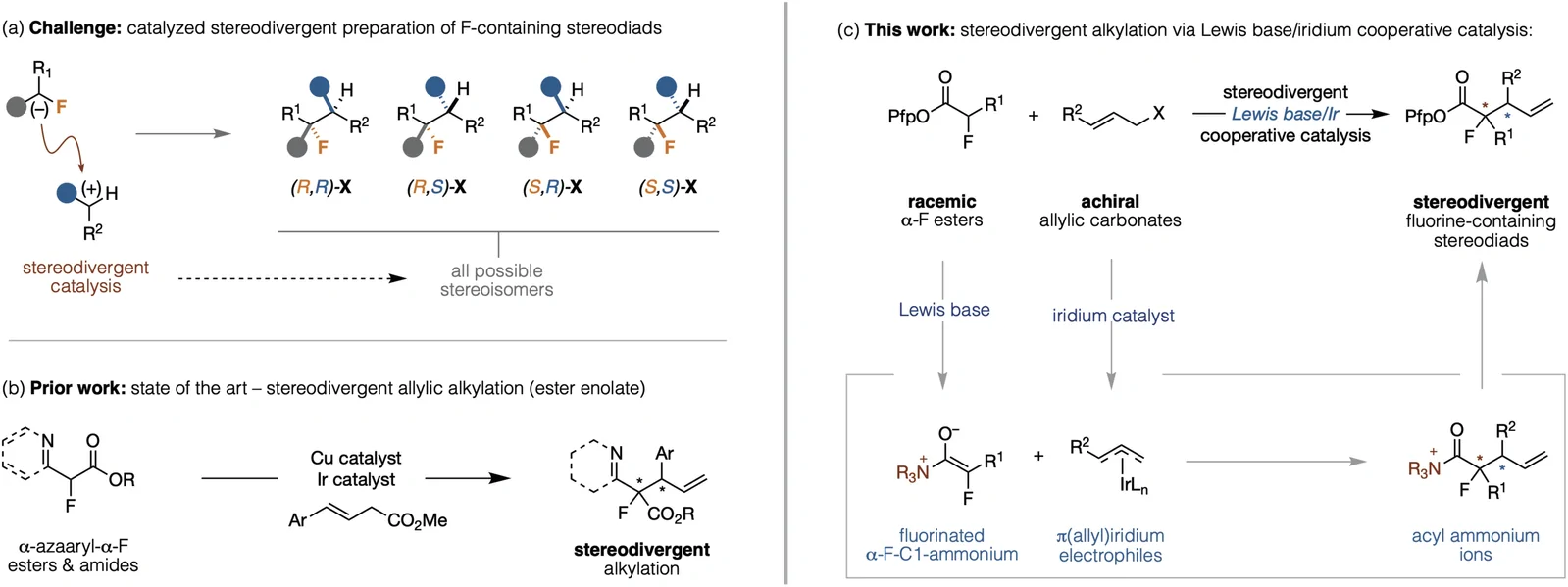
Motivation and approach.

Fluorine holds a position of unrivaled prominence in bioactive molecule design.^9^ Its incorporation enables precise tailoring of the physicochemical and biological properties that are key to molecular stability and function.^10^ Furthermore, Csp^3^–F incorporation permits the regulation of molecular conformation *via* an ensemble of stereoelectronic interactions.^11^ Collectively, these result in an intrinsic and predictive configuration-to-conformation output that has deep-seated implications in fields as disparate as medicinal chemistry^12^ and homogeneous catalysis.^13^ Our initial interests stem directly from (1) the capacity of fluorine to exquisitely preorganize molecular conformation through configuration-specific stereoelectronic interactions, and (2) the persistent challenge associated with catalyzed stereodivergent Csp^3^–Csp^3^ bond formation. Thus, we expected that a solution to (2) would enable a systematic interrogation of (1), and we expected that stereodivergent Lewis base/iridium cooperative catalyzed allylic alkylation would deliver the required direct and general approach. In the seminal disclosure in this area, Hartwig and co-workers described the Cu/Ir-catalyzed stereodivergent allylic alkylation of α-fluorinated α-azaaryl esters which proceeds *via* N-chelated Cu-enolates (Scheme 1b).^14^ Copper chelation provides access to the planar, single configuration enolates that are key to enantiofacial control during alkylation using chiral, non-racemic phosphine supporting ligands.^15^ Inspired by this, we sought a complementary stereodivergent method capable of accommodating simple arenes that cannot benefit from such chelation-based control. Herein, we report the stereodivergent synthesis of tertiary fluoride-embedded vicinal stereodiads *via* cooperative Lewis base–iridium catalyzed allylic alkylation reactions (Scheme 1c). Specifically, we envisioned that α-fluorinated C1-ammonium enolates could be accessed from easily prepared racemic α-fluorinated esters *via* isothiourea Lewis base catalysis and subsequently engaged in stereodivergent bond construction upon union with an appropriate π(allyl)iridium electrophile.^16^ While non-fluorinated C1-ammonium enolates have been successfully merged with π(allyl)iridium electrophiles in a stereodivergent manner previously,^5,17^ the corresponding α-fluorinated congeners have not and the overall impact of the presumed reduced nucleophilicity of the C1-ammonium enolate engendered by the fluorine substituent was unclear.

## Results and discussion

Our efforts began by evaluating the union of racemic α-fluoroester 1 with cinnamyl *t*-butyl carbonate 2 under the action of various isothiourea Lewis bases (A–G) in conjunction with Hartwig's cyclometallated iridium catalyst Ir; a combination which has previously enabled outstanding levels of reactivity and stereocontrol in stereodivergent bond construction in non-fluorinated sterodiads.^5,17^ (Table 1). Beginning with the prototypical isothiourea (*S*)-BTM A in combination with (*R*_*a*_,*R*,*R*)-Ir and Hünig’s base in THF at room temperature we were delighted to obtain the desired allylic alkylation pentafluorophenyl ester (Pfp) product (*R*,*R*)-3 in reasonable yield (48%), with encouraging levels of diastereocontrol (dr = 87 : 13), as a single detectable enantiomer (Entry 1).

**Table 1:** Reaction optimization

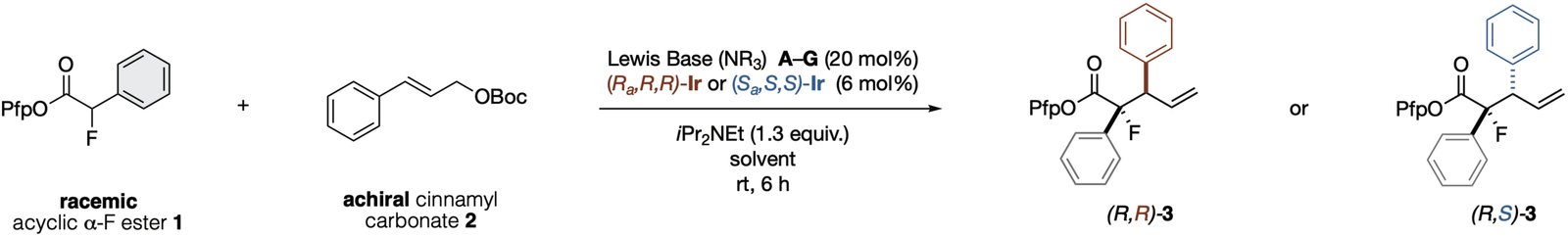								
Entry	Lewis base (NR_3_)	Solvent	Yield (%)		Diastereomeric ratio		Enantiomeric ratio	
			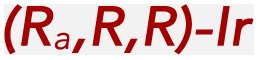	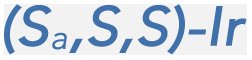	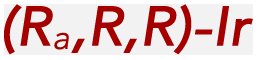	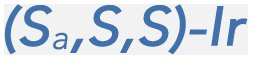	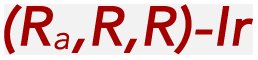	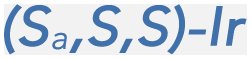
1	(*S*)-BTM (A)	THF	48%	36%	87 : 13	17 : 83	>99 : 1 (*R*,*R*)	>99 : 1 (*R*,*S*)
2	(*S*)-Bn-BTM (B)	THF	71%	63%	95 : 05	08 : 92	>99 : 1 (*R*,*R*)	>99 : 1 (*R*,*S*)
3	(*S*)-Me-BTM (C)	THF	66%	56%	96 : 04	07 : 93	>99 : 1 (*R*,*R*)	>99 : 1 (*R*,*S*)
4	(*R*)-*i*-Pr-BTM (D)	THF	59%	59%	10 : 90	90 : 10	>99 : 1 (*R*,*R*)	>99 : 1 (*R*,*S*)
5	(*S*)-*t*-Bu-BTM (E)	THF	58%	53%	64 : 36	34 : 66	>99 : 1 (*R*,*R*)	>99 : 1 (*R*,*S*)
6	(*S*)-*s*-Bu-BTM (F)	THF	39%	40%	50 : 50	48 : 52	>99 : 1 (*R*,*R*)	>99 : 1 (*R*,*S*)
7	(*S*)-HBTM (G)	THF	37%	28%	48 : 52	46 : 54	>99 : 1 (*R*,*R*)	>99 : 1 (*R*,*S*)
8	(*S*)-Me-BTM (C)	CH_2_Cl_2_	60%	56%	93 : 07	10 : 90	98 : 2 (*R*,*R*)	98 : 2 (*R*,*S*)
9	(*S*)-Me-BTM (C)	PhCH_3_	67%	62%	95 : 05	08 : 92	>99 : 1 (*R*,*R*)	>99 : 1 (*R*,*S*)
10	(*S*)-Me-BTM (C)	MeCN	61%	58%	92 : 08	12 : 88	>99 : 1 (*R*,*R*)	>99 : 1 (*R*,*S*)
11	(*S*)-Me-BTM (C)	DME	67%	57%	96 : 04	08 : 92	>99 : 1 (*R*,*R*)	>99 : 1 (*R*,*S*)
12	(*S*)-Me-BTM (C)	1,4-Dioxane	86%	72%	96 : 04	08 : 92	>99 : 1 (*R*,*R*)	>99 : 1 (*R*,*S*)
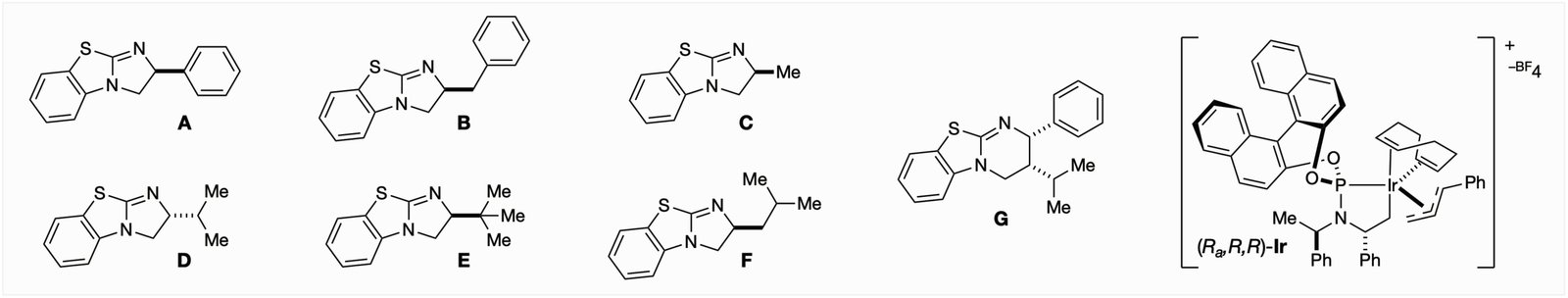								

More importantly, when (*S*)-BTM A was paired with the antipodal (*S*_*a*_,*S*,*S*)-Ir catalyst the diastereoisomeric (*R*,*S*)-3 product was obtained as the major isomer (dr = 17 : 83) in modest yield (36%) and as a single detectable enantiomer (er = >99 : 1). Encouraged by these initial results we evaluated a range of isothiourea catalysts (B–G). Promisingly, all functioned as effective Lewis base catalysts within this cooperative catalysis processes. While both (*S*)-*s*-BuBTM F and (*S*)-HBTM G (Entries 6 and 7) lacked meaningful levels of diastereocontrol (dr = 50 : 50–46 : 54) but otherwise exhibit high levels of enantiocontrol, all other isothioureas (A–E) demonstrated comparable or better levels of efficiency and stereocontrol (Entries 2–5); both (*S*)-Bn-BTM B and (*S*)-Me-BTM C were each markedly better in their capacity for sterodivergent bond construction (Entries 2 and 3, dr = 96 : 4–7 : 93). Given the slightly higher levels of diastereoselection offered by (*S*)-Me-BTM C we then moved to evaluate the effect of the solvent on both the efficiency and stereoselectivity of the reaction (Entries 8–12); this revealed 1,4-dioxane to be most effective (Entry 12). Full assessment of other reaction parameters is available in the SI. Next, we sought to (1) illustrate stereodivergence by preparation of all four alkylation stereoisomers, and (2) conclusively secure the relative and absolute stereochemical constitution of each. This latter endeavor was also driven by the expectation that the ensemble of stereoelectronic interactions uniquely presented by incorporation of the fluorinated stereogenic unit would confer well-defined control over the molecular conformation and would be readily revealed by single-crystal X-ray diffraction. Accordingly, we performed the alkylation reaction under the aegis of all catalyst enantiomer combinations. The stereoisomeric pentafluorophenyl esters (Pfp) produced were converted to the corresponding *p*-bromobenzylamides (3-amide) by treatment of each with *p*-bromobenzylamine (Scheme 2). Each stereoisomeric product was obtained in high overall yield, with excellent levels of diastereoselectivity, and as a single detectable enantiomer. Single crystal X-ray structure analysis of each provided unambiguous confirmation of the expected relative and absolute stereochemistry and further illustrates the remarkable levels of stereocontrol each chiral non-racemic catalyst delivers over each independent stereogenic center, with the Lewis base catalyst (C) dictating the α- and the Ir-catalyst dictating the β-stereogenic center, respectively. Collectively, and in support of our initial expectations, these data substantiate the notion that racemic α-fluoroesters converge to single configuration C1-ammonium enolates and that reaction with chiral non-racemic π(allyl)iridium electrophile intermediates is both efficient and stereodivergent thereby underscoring the modularity of this manifold.

**Scheme 2:**
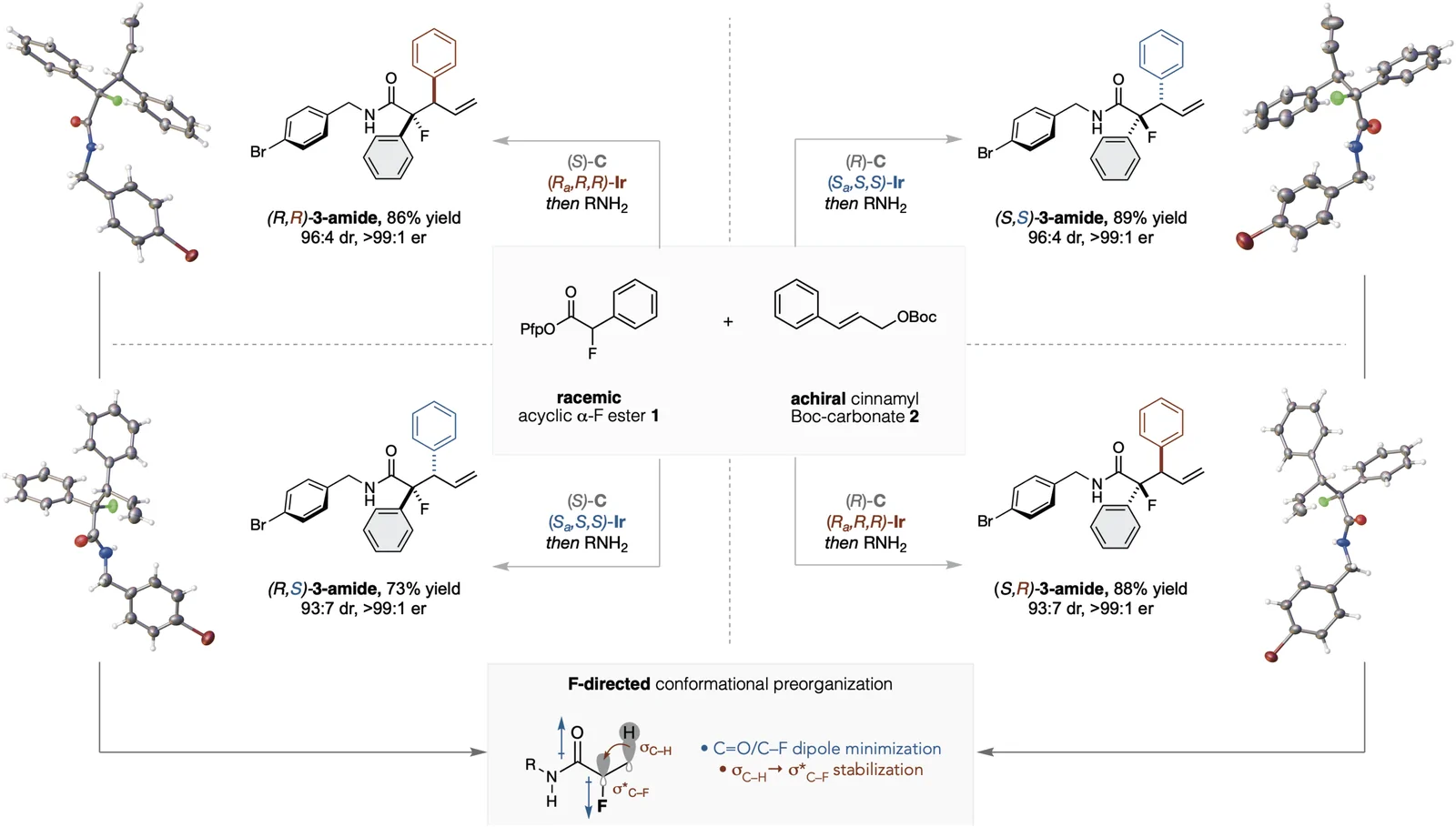
Illustration of stereodivergence.

The stereochemistry of each stereoisomer is consistent with product outcomes reported previously for the union of non-fluorinated isothiourea-derived C1-ammonium enolates with π(allyl)iridium species;^5,17^ this is suggestive of alkylation *via* α-fluorinated (*E*)-O-C1-ammonium enolates (as shown in Scheme 1C). In addition, and as per our expectation, each stereoisomer displayed an acyclic conformation in the solid state consistent with fluorine-directed stereoelectronic preorganization arising from a combination of (1) C=O/C–F dipole minimization, and (2) stabilizing 
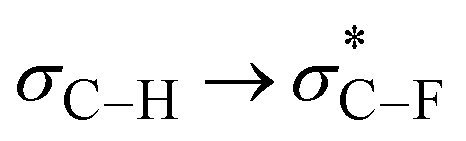
 interaction (Scheme 2, bottom).^18^ In the (*S*,*S*)- and (*R*,*R*)-3-amide stereoisomers this arrangement stabilizes gauche orientation of the aryl rings.

With fully optimized conditions identified and having illustrated preparation of all stereoisomers and secured their relative and absolute stereochemistry, we next moved to evaluate the scope of the reaction. As shown in Scheme 3, we evaluated the scope of the racemic acyclic α-fluoroester component in combination with various cinnamyl *t*-butylcarbonates. Based on our optimization (Table 1) we expected efficient stereodivergent bond construction giving (*R*,*R*) or (*R*,*S*) diastereomers as single detectable enantiomers using either (S)-C/(*R*_*a*_,*R*,*R*)-Ir or (*S*)-C/(*S*_*a*_,*S*,*S*)-Ir catalyst permutations, respectively. Beginning with evaluation of the racemic acyclic α-fluoro ester nucleophile component, as expected, the reaction demonstrated tolerance to the usual substituents (3–14) including p-extended systems (4), halides spanning F–I (6, 9–12), trifluoromethyl (13) and trifluoromethoxy (14). The reaction is limited by two features: both aliphatic Pfp esters and *ortho*-substituted aryl Pfp esters are unreactive. The electrophile scope was similarly tolerant of a range of substitution on the arene component (15–26) including π-extended naphthyl (15), F–Br halide substitution (17–19), nitro (21), and heteroaromatics such as furyl (24), thienyl (25), and indole (26).

**Scheme 3:**
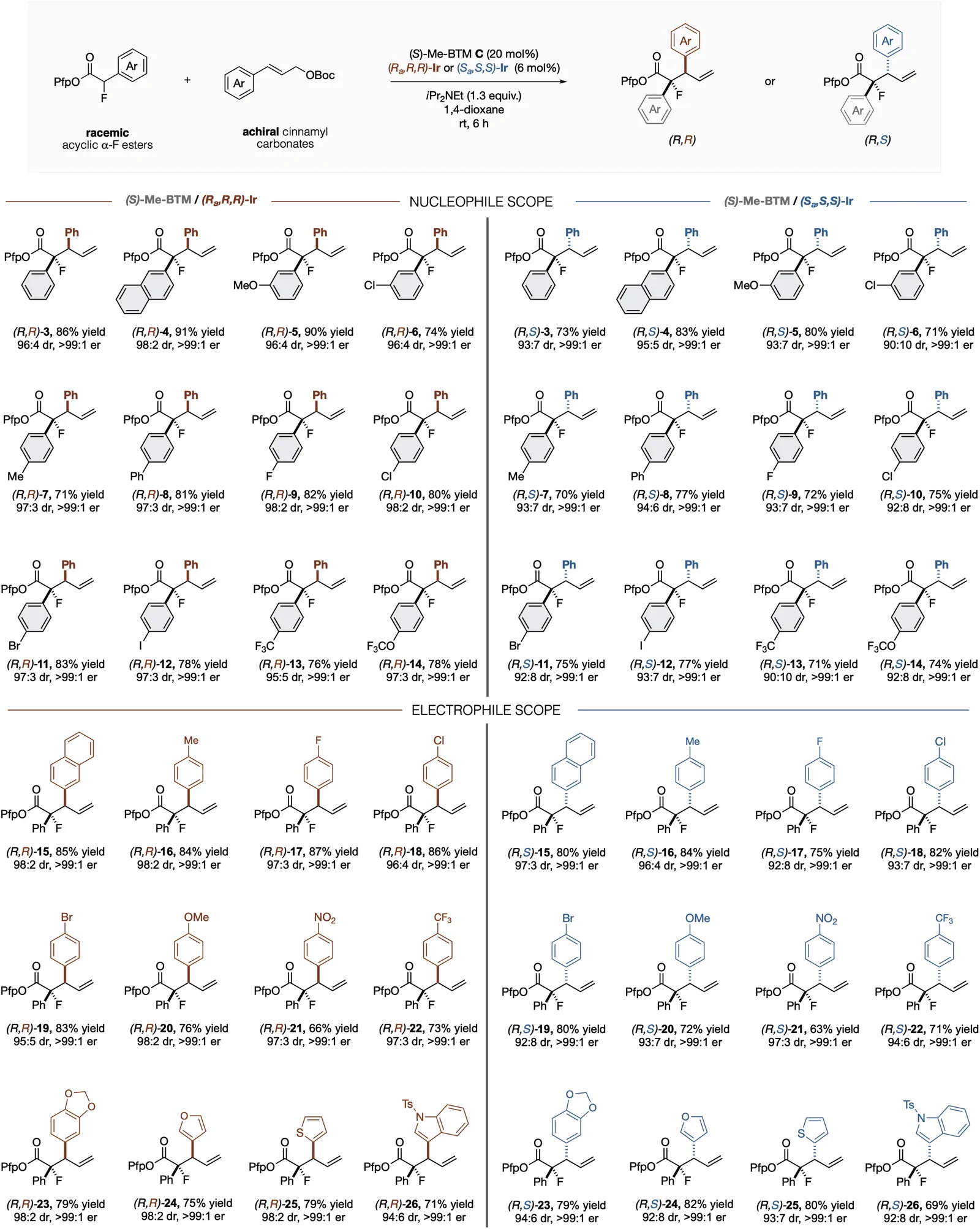
Reaction scope.

With regard to reaction stereoselectivity and stereodivergence, when using the (*S*)-C/(*R*_*a*_,*R*,*R*)-Ir catalyst combination each product in the (*R*,*R*) alkylation series was obtained as essentially a single diastereoisomer and as a single detectable enantiomer. When employing the (*S*)-C/(*S*_*a*_,*S*,*S*)-Ir catalyst combination, production of the (*R*,*S*) alkylation product was also similarly stereoselective. Overall, these data confirm the excellent levels of individual control imparted by each catalyst within this stereodivergent cooperative process.

The pentafluorophenyl ester (Pfp) moiety present in each product provides a useful synthetic handle to effect further transformation under mild conditions (Scheme 4). As expected, (*R*,*R*)-3 and (*R*,*S*)-3 were converted to alcohols 27 upon reduction with lithium aluminum hydride, trans esterified with methanol to methyl esters 28, and converted to primary amides 29 or allyl amides 30 upon treatment with ammonium hydroxide and allylamine, respectively. In each case both structural and stereochemical integrity of the neighbouring tertiary fluoride motif remain intact; no dehydrofluorination was observed.

**Scheme 4:**
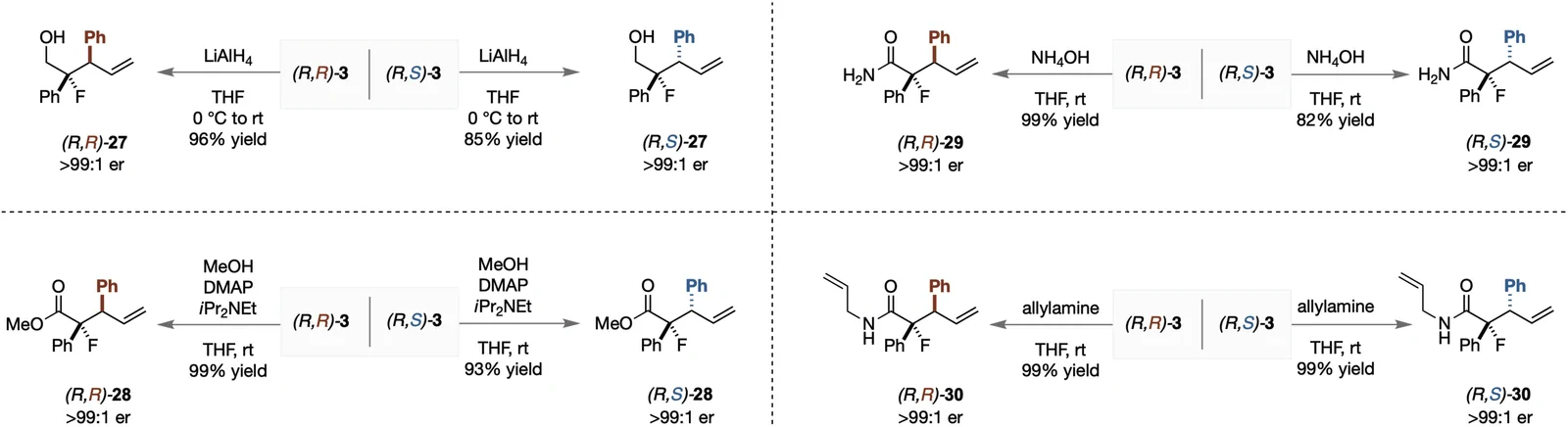
Product transformations.

## Conclusions

In summary, we have developed a stereodivergent Lewis base/iridium catalyzed alkylation that enables the straightforward preparation of vicinal stereogenic centers containing tertiary fluoride. This versatile reaction proceeds *via* the union of easily prepared racemic α-fluoro esters and cinnamyl *t*-butyl carbonates, and allows any stereochemical outcome to be obtained in excellent yields and stereoselectivity by appropriate combination of catalyst permutations. In addition, single crystal structure analyses of benzylamide derivatives of each stereoisomer reveal configuration-specific and fluorine-enabled conformational preorganization governed by dipole–dipole minimization and stabilizing 
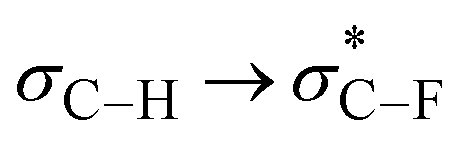
 interactions. The functional potential of such preorganization is the subject of current on-going study in our laboratory. Finally, this synthetic protocol complements existing state of the art catalyzed stereodivergent approaches and those existing strategies predicated on stereospecific and stereoselective C–F bond formation.^7,19^

## Author contributions

S. M. and T. N. S. designed and developed the project. Funding was acquired by T. N. S.; S. M. performed the experiments. M. P. collected and interpreted the X-ray structure data. T. N. S. administered the project and supervised the research. All authors contributed to the manuscript.

## Conflicts of interest

There are no conflicts to declare.

## Supplementary Material

SC-OLF-D6SC06429A-s001

SC-OLF-D6SC06429A-s002

## Data Availability

CCDC 2349144, 2349145, 2349147 and 2349148 contain the supplementary crystallographic data for this paper.^20*a*–*d*^ The data supporting this article have been included as part of the supplementary information (SI). Supplementary information is available. See DOI: https://doi.org/10.1039/d6sc06429a.
