# Supplementary material for: Stereodivergent Csp3–Csp3 bond construction via Lewis base/iridium cooperative catalysis: a modular synthesis of tertiary fluoride-containing stereodiads
Source: Chem Sci. 2026 Oct 21. Online ahead of print. doi: 10.1039/d6sc06429a (PMC13684430; doi:10.1039/d6sc06429a)
Supplement: Supplementary file 1 [file SC-OLF-D6SC06429A-s002.pdf]

Supplementary Information (SI) for Chemical Science.
This journal is © The Royal Society of Chemistry

**Table of Contents**

1. General Information…………………………………………………..………………**S2**
2. Experimental Section
    1.1 Catalysts ………………………………………………………………..……..**S3**
    1.2 Starting material synthesis ……………...…………………………………..….**S3**
    1.3 Reaction optimization………...………………………..………………………..**S8**
    1.4 General Procedure-Synthesis of tertiary fluoride…………………………**S10**
    1.5 Nucleophile scope ……….....…………………………………………………**S12**
    1.6 Electrophile scope …………………………..……………………….….**S71**
    1.7 Product transformations ……………………………..…………………**S127**
3. Confirmation of absolute stereochemistry
    a. X-Ray analysis of (*R*,*R*)-**3-amide**…………………………………..….**S141**
    b. X-Ray analysis of (*R*,*S*)- **3-amide**…..……………………………..……...**S142**
    c. X-Ray analysis of (*S*,*R*)-**3-amide**………………………………...……….**S143**
    d. X-Ray analysis of (*S*,*S*)-**3-amide**………………...…...……….……...**S144**
4. References ……………………………………………………….………………**S145**
5. NMR Spectra………………………………………………………………..……**S146**

## General Information

Commercial reagents were purified prior to use following the guidelines of Perrin and Armarego.[1] Unless otherwise noted, all reactions have been carried out with distilled and degassed solvents under an atmosphere of dry $N_2$ in flame or oven dried glassware with standard vacuum-line techniques. All reactions were carried out in Teflon screw cap reaction vials with magnetic stirring unless otherwise indicated. Dichloromethane, tetrahydrofuran and acetonitrile were purified under a positive pressure of dry argon by passage through two columns of activated alumina. Toluene was purified under a positive pressure of dry argon by passage through columns of activated alumina and Q5 (Grubbs apparatus). 1,2-Dimethoxyethane and 1,4-dioxane were dried overnight over 4 Å molecular sieves prior to use. All workup and purification procedures were carried out with reagent grade solvents (purchased from Sigma-Aldrich) in air. Standard column chromatography techniques using ZEOprep 60/40-63 μm silica gel were used for purification unless otherwise indicated. Liquids and solutions were transferred via syringe or cannula.

$^{1}H$, $^{13}C$ and $^{19}F$ NMR spectra were recorded at room temperature (25 ℃) on Varian Inova or Bruker 500 instrumentation: Varian I400 ($^{1}H$ NMR at 400MHz and $^{19}F$ NMR at 376 MHz) and Bruker 500 ($^{1}H$ NMR at 500 MHz and $^{13}C$ NMR at 126 MHz) using deuterium lock. Data for $^{1}H$ NMR spectra are quoted relative to chloroform peak (7.26 ppm) and data for $^{13}C$ NMR spectra are quoted relative to chloroform peak (77.16 ppm) and are reported in terms of chemical shift (δ ppm). Data for $^{19}F$ NMR spectra are reported in terms of chemical shift (δ ppm). Carbon signals of pentafluorophenyl moiety in α-fluoro pentafluorophenyl esters in $^{13}C$ NMR are not observed clearly due to fluorine splitting. Data are reported as follows: chemical shift, multiplicity (s = singlet, d = doublet, t = triplet, q = quartet, br = broad, m = multiplet), coupling constants (Hz) and integration. Infrared spectra (IR) were obtained on the Bruker TENSOR II FTIR Spectrometer and recorded in wavenumbers ($cm^{-1}$). Melting points were obtained on a Thomas Hoover capillary melting point apparatus without correction. High Resolution Mass (HRMS) analysis was obtained using Electron Impact Ionization (EI), Chemical Ionization (CI), Electrospray (ESI) and Atmospheric Pressure Chemical Ionization (APCI) and reported as m/z (relative intensity) for the [M]+, [M+H]+ or [M+Na]+ molecular ion. Chiral HPLC analyses were performed on an Agilent 1200 Series system.

**Experimental Section**

**Catalysts:**

**(*S*)-(+)-methyl-benzotetramisole** and **(*R*)-(-)-Methyl-benzotetramisole** were prepared from (*S*)-(+)-2-amino-1-propanol and (*R*)-(-)-2-amino-1-propanol, respectively, following the procedure reported by Birman and co-workers.[2,3]

($R_a$,*R*,*R*)-**[Ir]** complex was prepared using (*R*)-phosphoramidite and (*R*)-BINOL and ($S_a$, *S*,*S*)-**[Ir]** complex was prepared using (S)-phosphoramidite and (S)-BINOL following previously reported procedure.[4]

**Starting material synthesis:**

***Nucleophilic partners****: α-Fluoro aryl acetic acid pentafluorophenyl esters*

α-Fluoro aryl acetic acid pentafluorophenyl esters were prepared following the route as shown below.

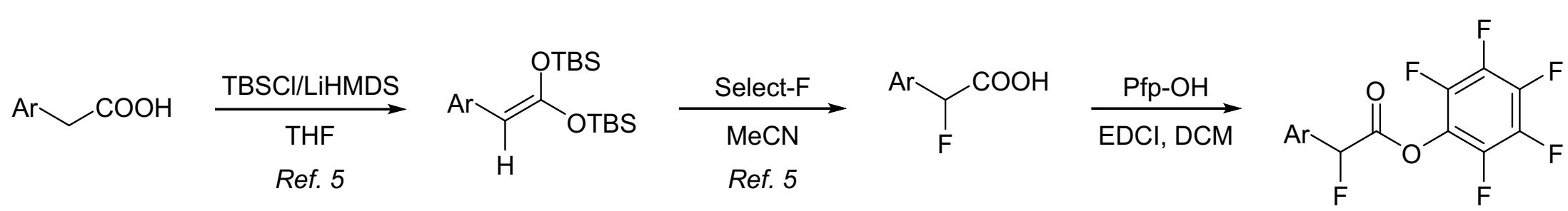


**1a-1l**

**General Procedure A for α-fluoro aryl acetic acid pentafluorophenyl esters:**

α-Fluoro-α-arylcarboxylic acids were synthesized following known literature procedure.[5] For the preparation of pentafluorophenyl ester, the corresponding acid (1.0 equiv.) was dissolved in DCM (0.2 M) in a round bottom flask. Pentafluorophenol (1.2 equiv.) was added to the reaction mixture before cooling to 0 °C. After addition of N-(3-dimethylaminopropyl)-N'-ethylcarbodiimide hydrochloride (1.2 equiv.), the reaction mixture was allowed to warm to room temperature and stirred overnight. The reaction mixture was transferred to a separatory funnel and washed sequentially with water and brine. The organic layer was dried using $Na_2SO_4$ and concentrated under reduced pressure. The pentafluorophenyl ester was purified by column chromatography by wet loading.

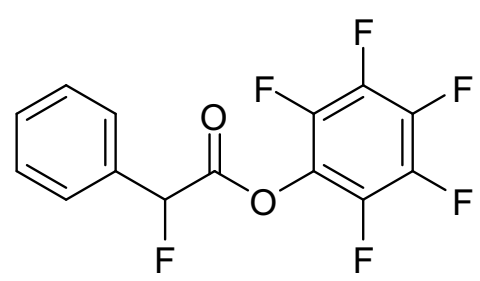
1a

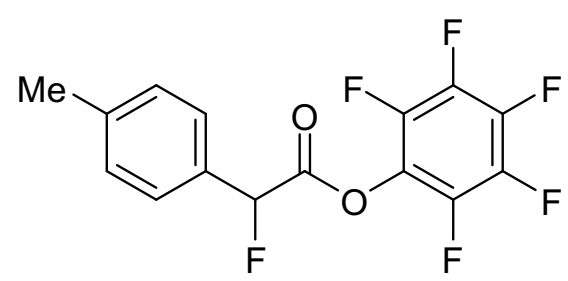
1b

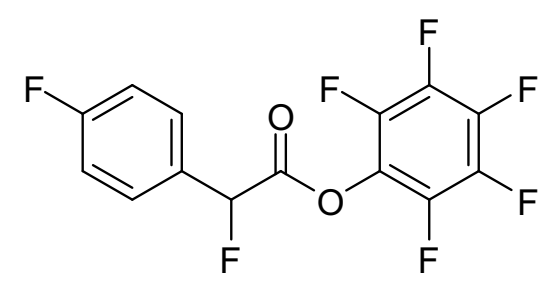
1c

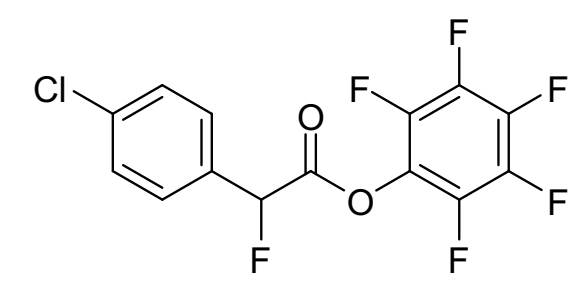
1d

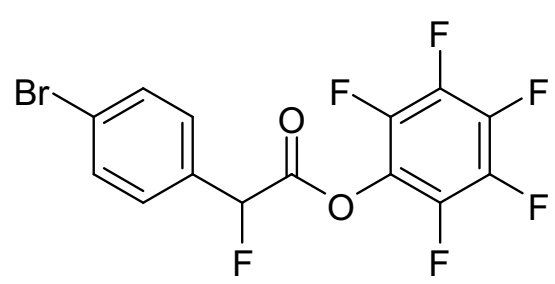
1e

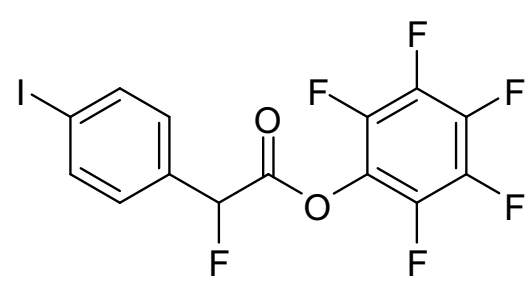
1f

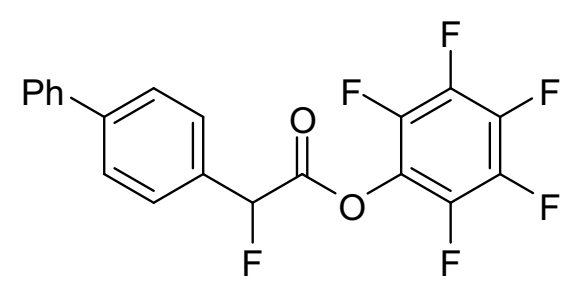
1g

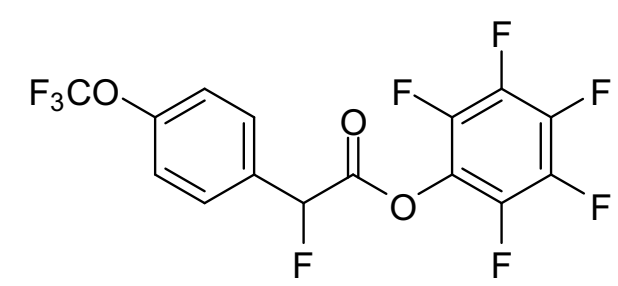
1h

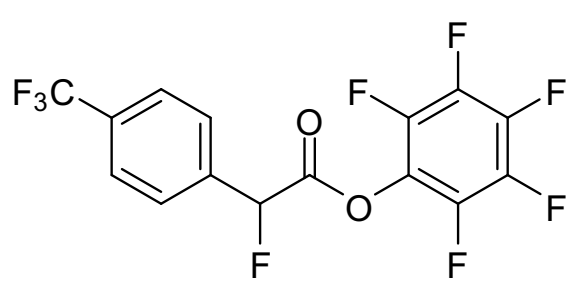
1i

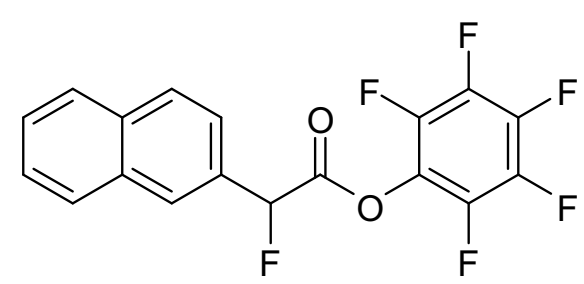
1j

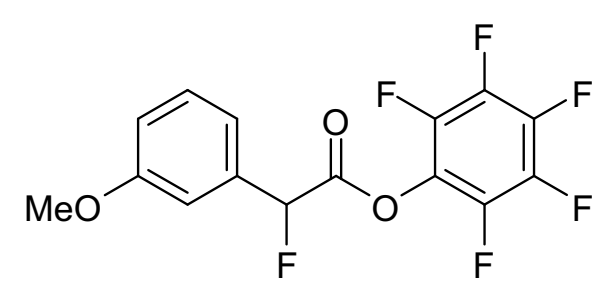
1k

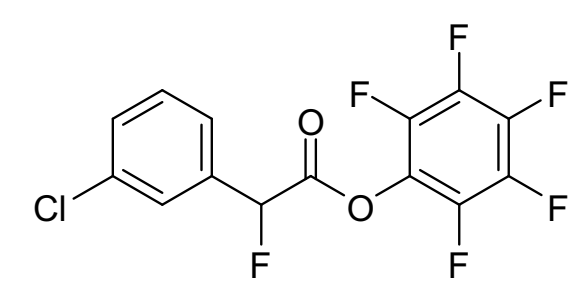
1l

α-Fluoro aryl acetic acid pentafluorophenyl esters **1a-1e, 1g, 1i-1j** has been reported previously.[5]

**Perfluorophenyl 2-fluoro-2-(4-iodophenyl)acetate (1f)**

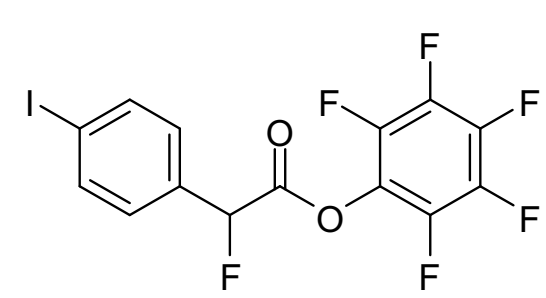

**IR (neat):** 1802, 1516, 1484, 1146, 1122, 1063, 1006, 983, 789 $cm^{-1}$.

**$^{1}H$ NMR (500 MHz, $CDCl_3$):** δ 7.88 – 7.79 (m, 2H), 7.33 – 7.27 (m, 2H), 6.06 (d, *J* = 46.8 Hz, 1H).

**$^{13}C$ NMR (126 MHz, $CDCl_3$):** δ 164.39 (d, *J* = 30.1 Hz), 138.54, 138.29, 132.29 (d, *J* = 20.7 Hz), 128.75 (d, *J* = 5.7 Hz), 88.45 (d, *J* = 189.9 Hz).

**$^{19}F$ NMR (376 MHz, $CDCl_3$):** δ -152.14 – -152.29 (m, 2F), -156.32 (t, *J* = 21.7 Hz, 1F), -161.28 – -161.48 (m, 2F), -179.45 (d, *J* = 46.9 Hz, 1F).

**HRMS (EI):** m/z calcd for [M]+ C14H5F6IO2: 445.9238. Found 445.9245.

**Perfluorophenyl 2-fluoro-2-(4-(trifluoromethoxy)phenyl)acetate (1h)**

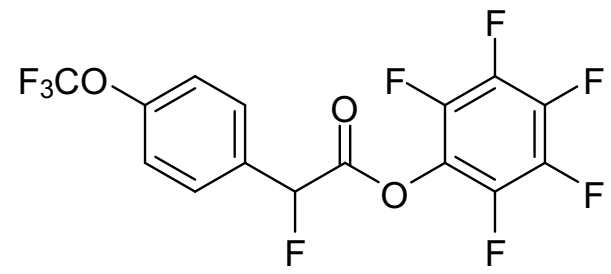


**IR (neat):** 3089, 1807, 1517, 1255, 1167, 1061, 990, 847, 799 $cm^{-1}$.

**$^1$H NMR (500 MHz, $CDCl_3$):** δ 7.63 – 7.59 (m, 2H), 7.36 – 7.31 (m, 2H), 6.14 (d, *J* = 46.8 Hz, 1H).

**$^{13}$C NMR (126 MHz, $CDCl_3$):** δ 164.43 (d, *J* = 30.1 Hz), 150.87, 131.22 (d, *J* = 20.7 Hz), 128.87 (d, *J* = 5.7 Hz), 121.61, 120.49 (q, *J* = 258.3 Hz), 88.14 (d, *J* = 190.0 Hz).

**$^{19}$F NMR (376 MHz, $CDCl_3$):** δ -57.88 (s, 1F), -152.25 – -152.41 (m, 2F), -156.31 (t, *J* = 21.7 Hz, 1F), -161.30 – -161.50 (m, 2F), -178.67 (d, *J* = 46.8 Hz, 1F).

**HRMS (ESI):** *m/z* calcd for $[M+Na]^+$ $C_9H_7F_4NO_2$:260.0305. Found 260.0304 (of the corresponding primary amide)

**Perfluorophenyl 2-fluoro-2-(3-methoxyphenyl)acetate (1k)**

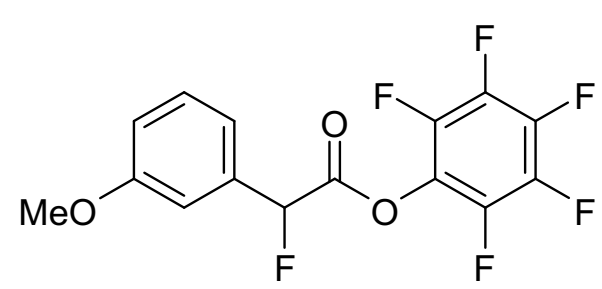


**IR (neat):** 3011, 2946, 2842, 1804, 1603, 1590, 1517, 1492, 1470, 1253, 1146, 1131, 1041, 990, 781, 695, 543 $cm^{-1}$.

**$^1$H NMR (500 MHz, $CDCl_3$):** δ 7.41 – 7.36 (m, 1H), 7.15 – 7.11 (m, 1H), 7.09 – 7.06 (m, 1H), 7.04 – 6.99 (m, 1H), 6.08 (d, *J* = 47.1 Hz, 1H), 3.85 (s, 3H).

**$^{13}$C NMR (126 MHz, $CDCl_3$):** δ 164.80 (d, *J* = 30.6 Hz), 160.26, 133.95 (d, *J* = 20.2 Hz), 130.42, 119.55 (d, *J* = 5.6 Hz), 116.60 (d, *J* = 2.5 Hz), 112.26 (d, *J* = 5.6 Hz), 88.92 (d, *J* = 189.3 Hz), 55.55.

**$^{19}$F NMR (376 MHz, $CDCl_3$):** δ -152.08 – -152.28 (m, 2F), -156.62 (t, *J* = 21.7 Hz, 1F), -161.46 – -161.67 (m, 2F), -177.80 (d, *J* = 47.2 Hz, 1F).

**HRMS (ESI):** *m/z* calcd for $[M+Na]^+ C_{10}H_{11}O_3FNa$: 221.0584. Found 221.0584

**Perfluorophenyl 2-(3-chlorophenyl)-2-fluoroacetate (1l)**

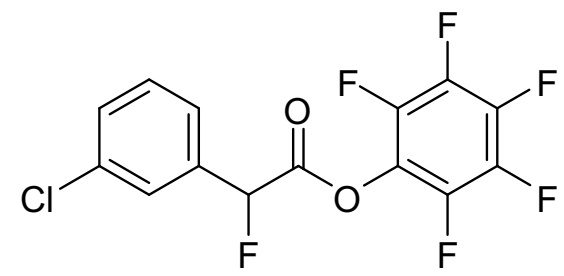


**IR (neat):** 3075, 2989, 2672, 2464, 2088, 1805, 1655, 1599, 1579, 1517, 1475, 1342, 1197, 1147, 1101, 1060, 999, 784, 706, 642, 572, 443 $cm^{-1}$.

**$^1$H NMR (500 MHz, CDCl$_3$):** δ 7.58 – 7.55 (m, 1H), 7.49 – 7.40 (m, 3H), 6.09 (d, *J* = 46.8 Hz, 1H).

**$^{13}$C NMR (126 MHz, CDCl$_3$):** δ 164.32 (d, *J* = 29.8 Hz), 135.36, 134.47 (d, *J* = 20.7 Hz), 130.84 (d, *J* = 2.1 Hz), 130.63, 127.30 (d, *J* = 6.1 Hz), 125.08 (d, *J* = 5.6 Hz), 88.17 (d, *J* = 190.7 Hz).

**$^{19}$F NMR (376 MHz, CDCl$_3$):** δ -152.12 – -152.30 (m, 2F), -156.30 (t, *J* = 21.8 Hz, 1F), -161.21 – -161.52 (m, 2F), -180.20 (d, *J* = 46.8 Hz, 1F).

**HRMS (EI):** *m/z* calcd for $[M]^+ C_9H_8ClFO_2$:202.0197. Found 202.0198 (of the corresponding methyl ester).

### Electrophilic partners: cinnamyl carbonates

Cinnamyl carbonates **2a-2m** were prepared following reported procedures.[7-9]

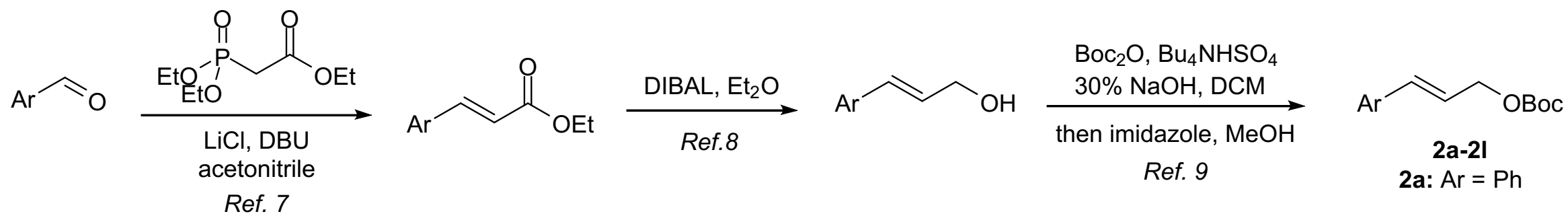


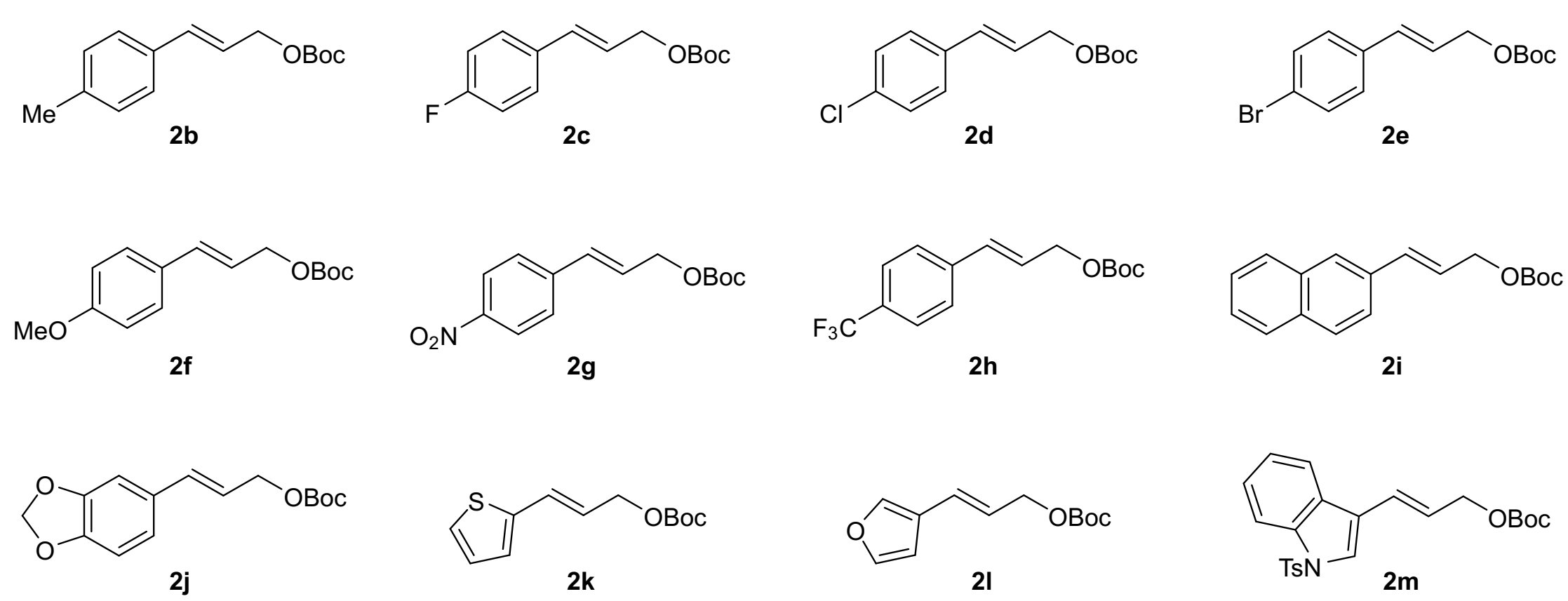

## Reaction Optimization

## **Table S1:** Optimization of catalyst loading

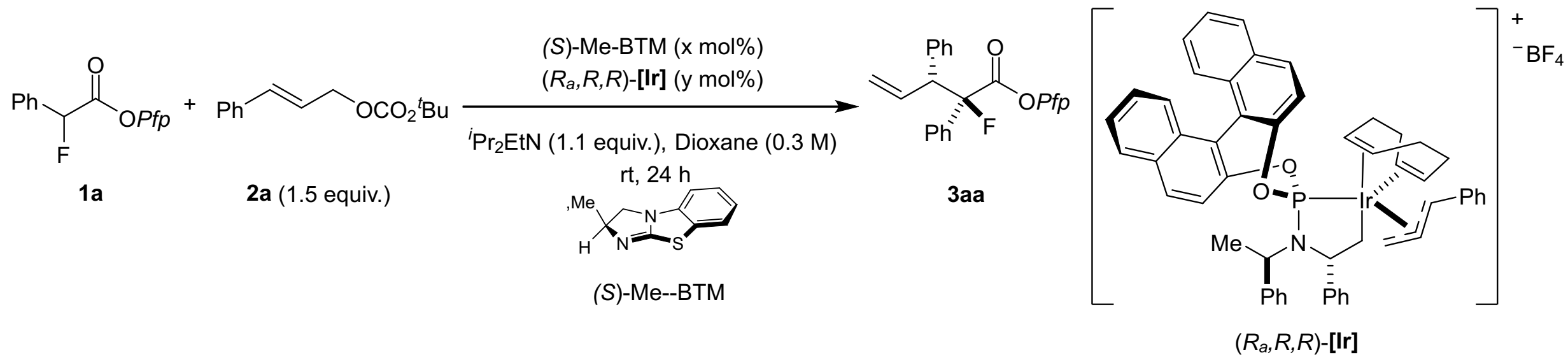


**1a** **2a** (1.5 equiv.) **3aa**

| Entry | Catalysts Load | | Yield[a] | *dr*[b] | *er*[c] |
|---|---|---|---|---|---|
| | (***S***)-Me-BTM | (*Ra,R,R*)-**[Ir]** | | | |
| 1 | 10 mol% | 2 mol% | 59% | 97:3 | >99:1 (*R,R*) |
| | 20 mol% | | 59% | 98:1 | >99:1 (*R,R*) |
| 2 | 10 mol% | 3 mol% | 72% | 96:4 | >99:1 (*R,R*) |
| | 20 mol% | | 75% | 97.5:2.5 | >99:1 (*R,R*) |
| 3 | 10 mol% | 4 mol% | 80.5% | 96:4 | >99:1 (*R,R*) |
| | 20 mol% | | 79.5% | 97:3 | >99:1 (*R,R*) |
| 4 | 10 mol% | 6 mol% | 85% | 95:5 | >99:1 (*R,R*) |
| | 20 mol% | | 86% | 96:4 | >99:1 (*R,R*) |

[a]Yield determined by $^{1}$H-NMR analysis of the unpurified reaction mixture (0.05 mmol) using mesitylene as an internal standard. [b]Determined by $^{19}$F-NMR analysis of the unpurified reaction mixture (0.05 mmol). [c]Enantiomeric ratio is determined by chiral HPLC analysis of the unpurified reaction mixture in comparison with the racemate.

**Table S2:** Optimization of reaction parameters

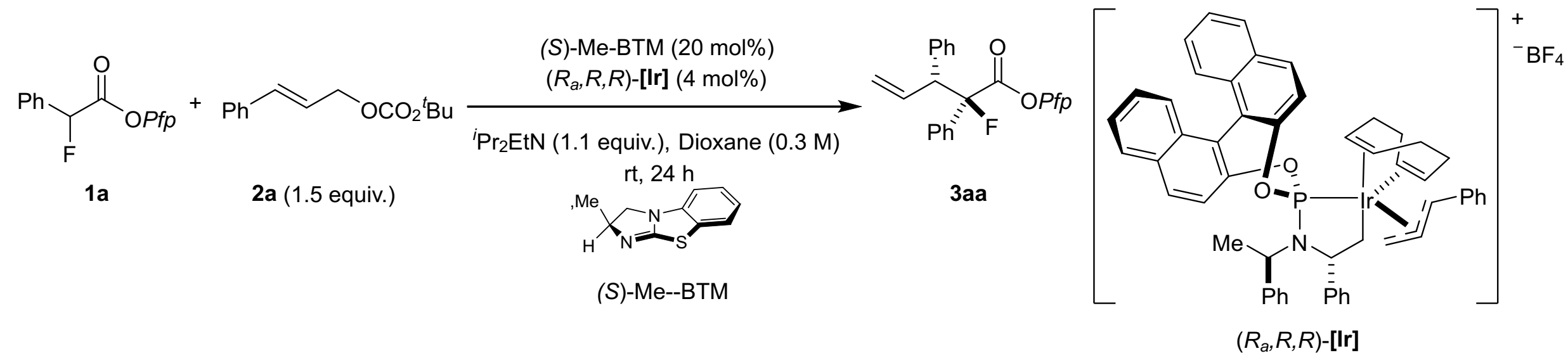


| Entry | Parameter | Yield[a] | *dr*[b] | *er*[c] |
|---|---|---|---|---|
| 1 | No change | 79.5% | 97:3 | >99:1 (*R,R*) |
| 2 | 1.1 equiv. of **2a** | 71.5% | 97:3 | >99:1 (*R,R*) |
| 3 | 1.3 equiv. of **2a** | 74% | 97:3 | >99:1 (*R,R*) |
| 4 | 1.3 equiv. of DIPEA | 89.5% | 97:3 | >99:1 (*R,R*) |
| 5 | 1.5 equiv. of DIPEA | 78% | 96:4 | >99:1 (*R,R*) |
| 6[d] | 0.2 M | 84% | 96:4 | >99:1 (*R,R*) |
| 7[d] | 0.5 M | 87.5% | 96.5:3.5 | >99:1 (*R,R*) |
| **8[d]** | **6 hours** | **89.5%** | **97:3** | **>99:1 (*R,R*)** |

[a]Yield determined by $^{1}$H-NMR analysis. [b]Determined by $^{19}$F-NMR analysis of the unpurified reaction mixture. [c]**D**etermined by chiral HPLC analysis of the unpurified reaction mixture in comparison with the racemate.[d]Using 1.3 equiv. of DIPEA

**General Procedure B: Allylation of α-fluoro aryl acetic acid pentafluorophenyl esters**

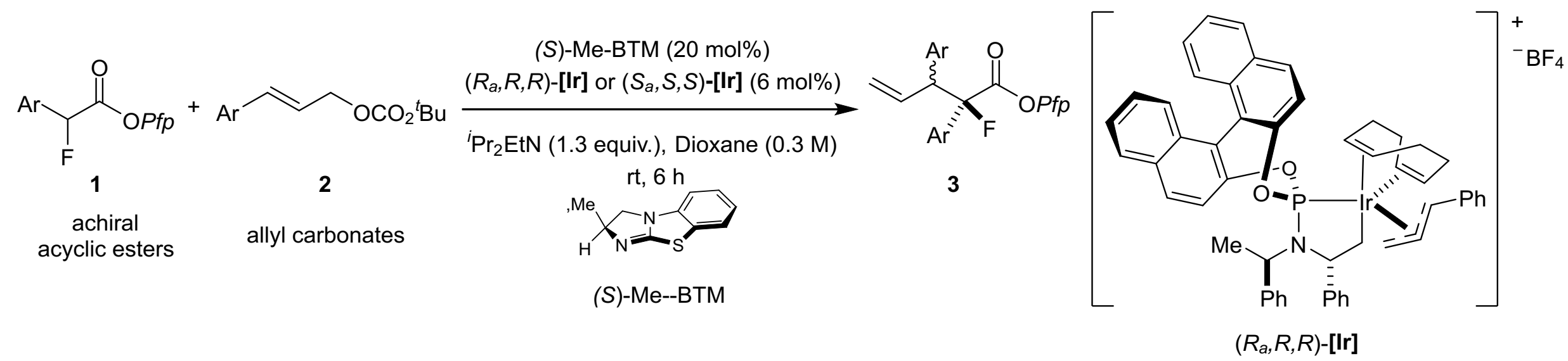


Allyl t-butyl carbonate **2** (0.15 mmol, 1.5 equiv.), α-fluoro aryl acetic acid pentafluorophenyl ester **1** (0.1 mmol, 1 equiv.), (*S*)-Me-BTM (3.8 mg, 20 mol%) and the specified **[Ir]**-catalyst (4.2 mg, 4 mol%) were added sequentially to an oven dried 1 dram vial equipped with stirrer bar. The vial was then sealed with a cap containing PTFE/silicone septa and purged with $N_2$ × 3 times before adding 1,4-dioxane (0.3 ml, 0.3 M) and DIPEA (22.6 µL, 0.13 mmol, 1.3 equiv.). The reaction mixture was then stirred at room temperature for 6 hours. After 6 hours, the cap was removed, and the reaction mixture was diluted with 1 ml of petroleum ether. After stirring for 5 minutes, the reaction mixture was passed through a 1-inch plug of deactivated silica gel.[10] The vial was then rinsed with 25% diethyl ether in petroleum ether and passed again through the silica plug. After removal of solvent under vacuum, mesitylene (7 µL, 0.05 mmol, 0.5 equiv.) was added as an internal standard. The crude reaction mixture was then diluted with $CDCl_3$ and analyzed by both $^1H$ and $^{19}F$ NMR. The reaction mixture was then concentrated under vacuum and the crude material was purified by column chromatography using deactivated silica gel[10] by wet loading with the specified eluent.

The ratio of diastereomers was determined by $^{19}F$-NMR analysis of the crude reaction mixture. The product was isolated by column chromatography as a mixture of two diastereomers strongly favoring one diastereomer over the other. The enantiomeric ratio was obtained by chiral HPLC analysis of the corresponding primary amide.

Silica gel was deactivated following a reported procedure.[10] In a 1L round-bottom flask, 100g of silica gel was suspended in 200 ml of dry chloroform. The flask was cooled to 0 °C using an ice-bath before dropwise addition of ethyltrichlorosilane (4.2 ml, 31 mmol).

Thereafter, the flask was closed and shaken vigorously to mix (HCl is formed). The mixture was allowed to warm to rt and stirred overnight under $N_2$ (suspension becomes yellow). The silica gel was collected by filtration using a Büchner funnel and subsequently washed twice with 200 mL portions of chloroform, followed by three washes with 200 mL portions of methanol. It was then transferred to a round-bottom flask and dried under vacuum at 40 °C using an oil bath.

**General Procedure C: Conversion of pentafluorophenyl ester products to corresponding primary amides for chiral HPLC analysis**

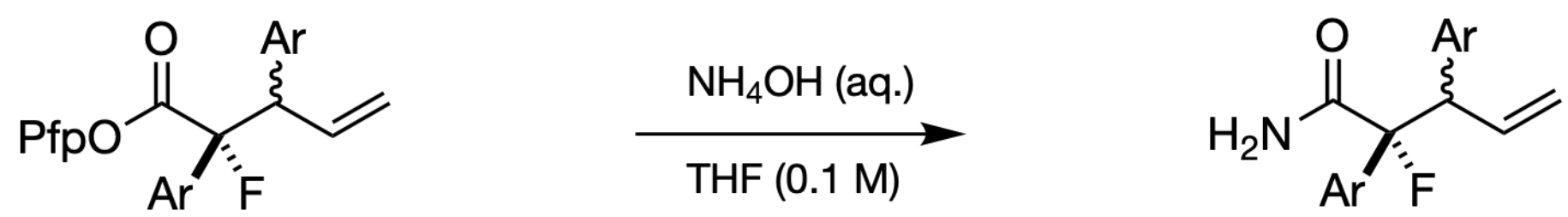


Pentafluorophenyl ester **3** (0.05 mmol, 1equiv.) was dissolved in THF (0.5 ml, 0.1 M) in a 1-dram vial equipped with stirrer bar. $NH_4OH$ (0.1 ml) was added, and the reaction mixture was stirred for 1 hour. After complete conversion, the solvent was removed under reduced pressure and the residue was redissolved in ethyl acetate (10mL) and transferred to a separatory funnel. The organic layer was washed sequentially with $Na_2CO_3$ (2M aq.) (3 x 10mL) and brine (1x10mL). The organic layer was dried with anhydrous $Na_2SO_4$ and filtered. The solvent was removed under reduced pressure to afford the corresponding primary amide which was used directly for HPLC analysis without further purification.

The enantiomeric ratio was assigned by HPLC analysis of the product against a racemic sample. Racemic samples were obtained by performing the reaction with (*rac*)-**[Ir]** and (*rac*)-Bn-BTM to afford mixture of all 4 stereoisomers (in most cases, formation of (*R*,*R*)-**3–26** and (*S*,*S*)-**3–26** (obtained in ~ 1:1 ratio) were favored over formation of (*R*,*S*)-**3–26** and (*S*,*R*)-**3–26)** (obtained in ~ 1:1 ratio)). The notations "$t_{major}$" and "$t_{minor}$" refer to the HPLC retention times of the major and minor enantiomers of the major diastereomer. Other retention times refer to the minor diastereomer.

***Note***: *For $^{1}$H NMR, the shifts of the characteristic peaks of the minor diastereomer have been reported (marked with 'asterisk') when present in >5% integral value. Only the shifts of the major diastereomer have been reported in case of $^{13}$C and $^{19}$F NMR for clarity purpose.*

## NUCLEOPHILE SCOPE

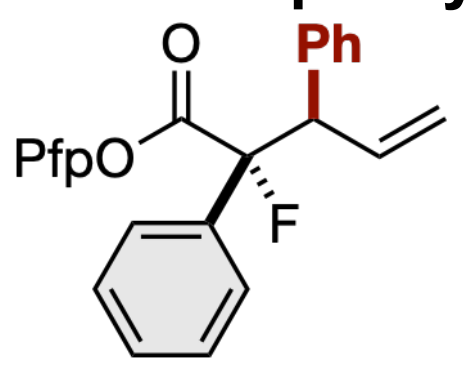

**Perfluorophenyl (2*R*,3*R*)-2-fluoro-2,3-diphenylpent-4-enoate (*R*,*R*)-3):**Prepared according to General Procedure B using pentafluorophenyl ester **1a** (32 mg, 0.1 mmol, 1 equiv.), *t*-butyl carbonate **2a** (35.1 mg, 0.15 mmol, 1.5 equiv.), (*R<sub>a</sub>,R,R*)**-[Ir]** (4.2 mg, 4 mol%), (*S*)-Me-BTM (3.8 mg, 20 mol%) and DIPEA (22.6 µL, 0.13 mmol, 1.3 equiv.) in 1,4-dioxane (0.3 ml, 0.3 M). The *title compound* was obtained (37.5 mg, 0.086 mmol, 86%) as a colorless oil following purification by column chromatography [$SiO_2$, 0.25% Ethyl Acetate in Petroleum Ether]. The enantiomeric ratio (>99:1 *er*) of the corresponding para-bromobenzylamide amide was determined by chiral HPLC in comparison with the racemate. Diastereomeric ratio (96:4) was determined by $^{19}$F-NMR analysis of the crude reaction mixture.

**IR (film):** 3065 (w), 3032 (w), 1781 (m), 1639 (w), 1602 (w), 1451 (w), 1518 (s), 1202 (m), 1180 (m), 1008 (s), 995 (s), 734 (s), 694 (s) cm$^{-1}$.

**$^1$H NMR (500 MHz, $CDCl_3$):** δ 7.39 – 7.31 (m, 2H), 7.21 – 7.14 (m, 3H), 7.08 – 7.03 (m, 5H), 6.31 (ddd, *J* = 17.0, 10.2, 8.7 Hz, 1H), 5.32 (d, *J* = 17.0 Hz, 1H), 5.28 (d, *J* = 10.3 Hz, 1H), 4.33 (dd, *J* = 30.0, 10.0 Hz, 1H).

**$^{13}$C NMR (126 MHz, $CDCl_3$):** δ 166.5 (d, *J* = 28.1 Hz), 136.6, 135.1 (d, *J* = 22.4 Hz), 134.5 (d, *J* = 5.1 Hz), 129.6 (d, *J* = 2.3 Hz), 129.0, 128.5 (d, *J* = 2.2 Hz), 128.3, 127.4, 125.0 (d, *J* = 10.0 Hz), 119.9, 99.8 (d, *J* = 201.5 Hz), 57.5 (d, *J* = 19.0 Hz).

**$^{19}$F NMR (376 MHz, $CDCl_3$):** δ -151.36 – -151.54 (m, 2F), -156.97 (t, *J* = 21.7 Hz, 1F), -161.35 – -162.07 (m, 2F), -174.59 (d, *J* = 33.0 Hz, 1F).

**HRMS (EI):** m/z calcd for $[M]^+$ $C_{23}H_{14}F_6O_2$: 436.0898. Found: 436.0902.

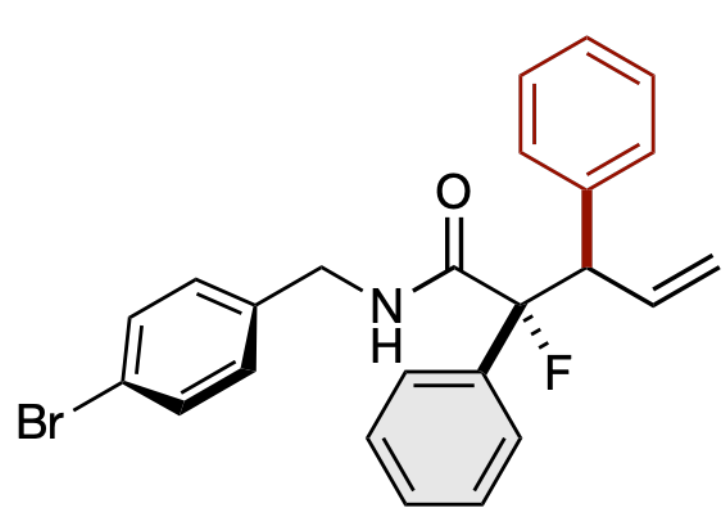

**(2*R*,3*R*)-N-(4-bromobenzyl)-2-fluoro-2,3-diphenylpent-4-enamide (*R*,*R*)-3-amide):** Pentafluorophenyl ester (*R*,*R*)-**3** (57.2 mg, 0.13 mmol, 1 equiv.) was dissolved in THF (1.3 ml, 0.1 M) in a 1-dram vial equipped with stirrer bar. 4-Bromobenzylamine (21.3 µ, 0.17 mmol, 1.3 equiv.) and

diisopropylethylamine (34 μl, 0.19 mmol, 1.5 equiv.) were added and the reaction mixture was stirred at room temperature. After 1 hour, the reaction mixture was diluted with EtOAc and transferred to a separatory funnel. The organic layer was washed sequentially with 1N HCl (10 ml), $Na_2CO_3$ (2M aq.) (3 x 10mL) and brine (10 ml). The organic layer was dried with anhydrous $Na_2SO_4$, filtered and the solvent was removed under reduced pressure. The title compound was obtained (54.6 mg, 0.012 mmol, 96%) as white solid following purification by column chromatography [$SiO_2$, 5% to 30% Ethyl Acetate in Petroleum Ether]. The enantiomeric ratio (>99:1 *er*) was determined by chiral HPLC in comparison with the racemate.

**IR (film):** 3365 (br), 3031 (w), 1660 (s), 1524 (s), 1488 (m), 1449 (m), 714 (m), 696 (m) $cm^{-1}$.

**$^1$H NMR (500 MHz, $CDCl_3$):** δ 7.46 – 7.39 (m, 4H), 7.23 – 7.16 (m, 3H), 7.14 – 7.04 (m, 7H), 6.80 (q, *J* = 6.0 Hz, 1H), 6.26 (ddd, *J* = 17.1, 10.3, 8.6 Hz, 1H), 5.24 (d, *J* = 17.1 Hz, 1H), 5.19 (dd, *J* = 10.2, 1.6 Hz, 1H), 4.55 (dd, *J* = 15.1, 6.6 Hz, 1H), 4.41 (dd, *J* = 34.5, 8.5 Hz, 1H), 4.24 (dd, *J* = 15.0, 5.3 Hz, 1H).

**$^{13}$C NMR (126 MHz, $CDCl_3$):** δ 170.0 (d, *J* = 21.2 Hz), 137.8, 137.1 (d, *J* = 22.1 Hz), 136.9, 135.3 (d, *J* = 5.8 Hz), 131.9, 129.6 (d, *J* = 2.2 Hz), 129.6, 128.1, 128.0 (d, *J* = 2.0 Hz), 126.9, 124.9 (d, *J* = 10.5 Hz), 121.6, 119.2, 101.7 (d, *J* = 196.9 Hz), 57.4 (d, *J* = 19.4 Hz), 42.8.

**$^{19}$F NMR (376 MHz, $CDCl_3$):** δ -173.20 (d, *J* = 34.7 Hz, 1F).

**HRMS (ESI):** m/z calcd for $[M+Na]^+$ $C_{24}H_{21}ONBrFNa$: 460.0683. Found: 460.0683.

HPLC analysis using a chiral column (Chiralpak IA 3μ column 22 ˚C, 1.0 ml/min, 95:5 hexane:isopropanol, 210 nm, $t_{major}$ = 16.965 min, $t_{minor}$ = 14.566 min, t = 20.022 min, t = 23.882 min).

(±)**-3-amide:** (*rac*)-**[Ir]** and (*rac*)-Bn-BTM

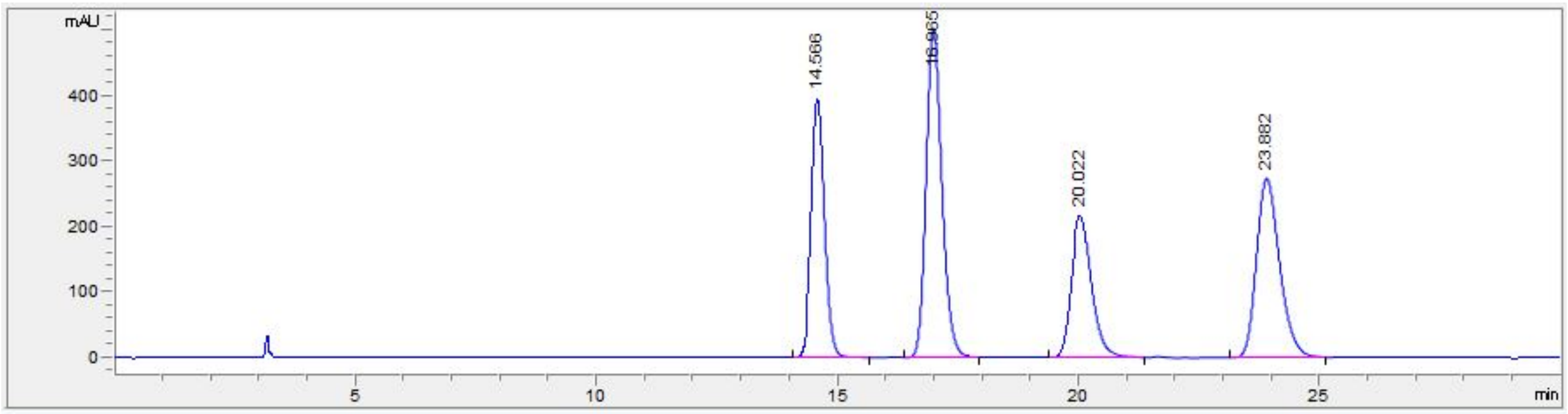


| # | Time | Area | Height | Width | Area% | Symmetry |
|---|---|---|---|---|---|---|
| 1 | 14.566 | 7409.9 | 395.5 | 0.2886 | 21.701 | 0.839 |
| 2 | 16.965 | 11424.7 | 502.6 | 0.3492 | 33.458 | 0.796 |
| 3 | 20.022 | 6194.1 | 217.2 | 0.4348 | 18.140 | 0.756 |
| 4 | 23.882 | 9117.1 | 273.3 | 0.5112 | 26.701 | 0.75 |

(*R*,*R*)-**3-amide:** ($R_a$,*R,R*)-**[Ir]** and (*S*)-Me-BTM

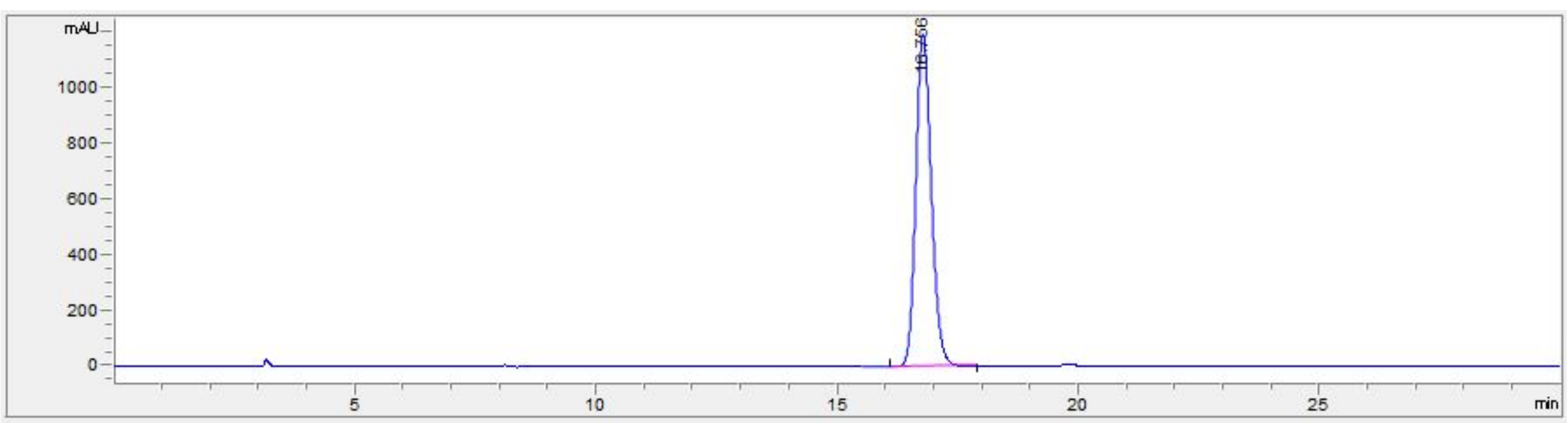


| # | Time | Area | Height | Width | Area% | Symmetry |
|---|---|---|---|---|---|---|
| 1 | 16.756 | 26600.4 | 1191.3 | 0.3445 | 100.000 | 0.813 |

**Perfluorophenyl (2*R*,3*S*)-2-fluoro-2,3-diphenylpent-4-enoate (*R*,*S*)-3:** Prepared

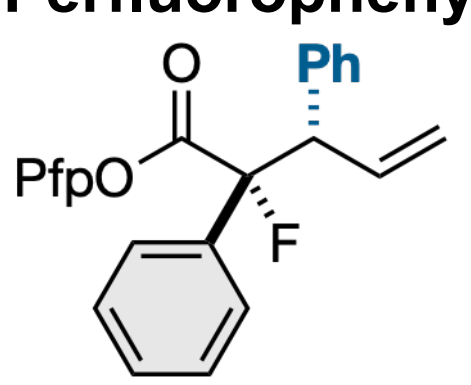


according to General Procedure B using pentafluorophenyl ester **1a** (32 mg, 0.1 mmol, 1 equiv.), *t*-butyl carbonate **2a** (35.1 mg, 0.15 mmol, 1.5 equiv.) and ($S_a$,*S,S*)-**[Ir]** (4.2 mg, 4 mol%), (*S*)-Me-BTM (3.8 mg, 20 mol%) and DIPEA (22.6 µL, 0.13 mmol, 1.3 equiv.) in 1,4-dioxane (0.3 ml, 0.3 M). The *title compound* was obtained (31.8 mg, 0.073 mmol, 73%) as a white solid following purification by column chromatography [$SiO_2$, 0.25% Ethyl Acetate in Petroleum Ether]. The enantiomeric ratio (>99:1 *er*) of the corresponding para-bromobenzylamide was determined by chiral HPLC in comparison with the racemate. Diastereomeric ratio (93:7) was determined by $^{19}$F-NMR analysis of the crude reaction mixture.

**IR (film):** 3065 (w), 3033 (w), 1780 (m), 1639 (w), 1603 (w), 1497 (m), 1517 (s), 1196 (m), 1007 (s), 996 (s), 739 (s), 696 (s) $cm^{-1}$.

**$^1$H NMR (500 MHz, $CDCl_3$):** δ 7.72 – 7.68 (m, 2H), 7.51 – 7.46 (m, 4H), 7.45 – 7.41 (m, 1H), 7.38 (t, *J* = 7.3 Hz, 2H), 7.36 – 7.29 (m, 1H), ***6.39 (ddd, J = 17.1, 10.3, 8.8 Hz)**, 5.98 (ddd, *J* = 17.1, 10.3, 7.9 Hz, 1H), ***5.47 – 5.32 (m)**, 5.07 (d, *J* = 10.3 Hz, 1H), 4.92 (d, *J* = 17.1 Hz, 1H), 4.42 (dd, *J* = 34.0, 8.0, 1H).

**$^{13}$C NMR (126 MHz, $CDCl_3$):** δ 166.0 (d, *J* = 28.6 Hz), 137.6, 135.7 (d, *J* = 22.6 Hz), 133.9 (d, *J* = 4.9 Hz), 129.6 (d, *J* = 3.1 Hz), 129.4, 128.9, 128.8, 128.0, 125.3 (d, *J* = 10.2 Hz), 119.9, 99.2 (d, *J* = 201.3 Hz), 57.0 (d, *J* = 18.7 Hz).

**$^{19}$F NMR (376 MHz, $CDCl_3$):** δ -151.58 – -152.01 (m, 2F), -157.24 (t, *J* = 21.7 Hz, 1F), -161.97 – -162.20 (m, 2F), -173.51 (d, *J* = 33.8 Hz, 1F).

**HRMS (EI):** m/z calcd for $[M]^+$ $C_{23}H_{14}F_6O_2$: 436.0898. Found: 436.0904.

**(2*R*,3*S*)-N-(4-bromobenzyl)-2-fluoro-2,3-diphenylpent-4-enamide (*R*,*S*)-3-amide):**

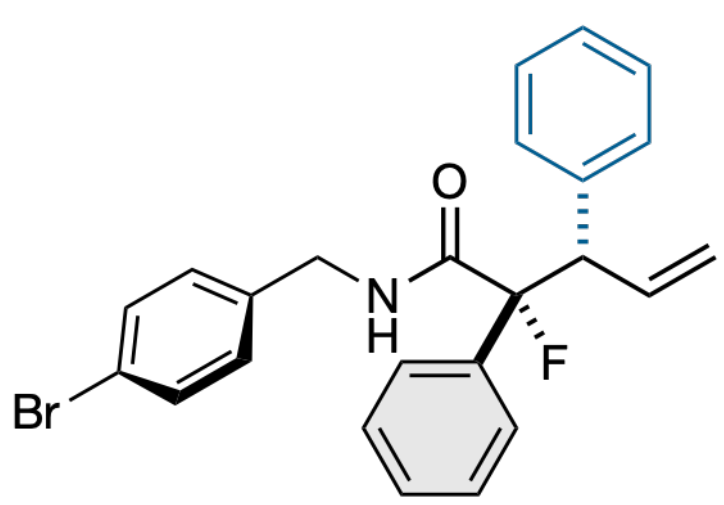


Pentafluorophenyl ester (*R*,*S*)-**3** (54 mg, 0.12 mmol, 1 equiv.) was dissolved in THF (1.2 ml, 0.1 M) in a 1-dram vial equipped with stirrer bar. 4-Bromobenzylamine (20.3 µ, 0.16 mmol, 1.3 equiv.) and diisopropylethylamine (32.4 µl, 0.19 mmol, 1.5 equiv.) were added and the reaction mixture was stirred at room temperature. After 1 hour, the reaction mixture was diluted with EtOAc and transferred to a separatory funnel. The organic layer was washed sequentially with 1N HCl (10 ml), $Na_2CO_3$ (2M aq.) (3 x 10mL) and brine (10 ml). The organic layer was dried with anhydrous $Na_2SO_4$, filtered and the solvent was removed under reduced pressure. The title compound was obtained (52.7 mg, 0.12 mmol, 97%) as a white solid following purification by column chromatography [$SiO_2$, 5% to 30% Ethyl Acetate in Petroleum Ether]. The enantiomeric ratio (>99:1 *er*) was determined by chiral HPLC in comparison with the racemate.

**IR (film):** 3411 (m), 2922 (m), 1662 (s), 1523 (s), 1486 (m), 1447 (m), 737 (m) $cm^{-1}$.

**$^1$H NMR (500 MHz, CDCl$_3$):** δ 7.70 (dt, *J* = 6.5, 1.3 Hz, 2H), 7.47 – 7.39 (m, 4H), 7.39 – 7.30 (m, 4H), 7.25 – 7.21 (m, 2H), 6.50 – 6.38 (m, 3H), 5.96 (ddd, *J* = 17.1, 10.4, 8.2 Hz, 1H), 4.99 (d, *J* = 10.4 Hz, 1H), 4.87 (dt, *J* = 17.1, 1.4 Hz, 1H), 4.54 – 4.33 (m, 2H), 3.78 (dd, *J* = 15.3, 4.3 Hz, 1H).

**$^{13}$C NMR (126 MHz, CDCl$_3$):** δ 169.6 (d, *J* = 21.6 Hz), 138.5, 137.4 (d, *J* = 22.1 Hz), 136.5, 134.5 (d, *J* = 4.6 Hz), 131.6, 130.2 (d, *J* = 2.3 Hz), 128.9, 128.6, 128.4 (d, *J* = 2.5 Hz), 127.4, 125.1 (d, *J* = 11.3 Hz), 121.2, 119.4, 101.3 (d, *J* = 198.2 Hz), 56.9 (d, *J* = 18.7 Hz), 42.2.

**$^{19}$F NMR (376 MHz, CDCl$_3$):** δ -175.99 (dd, *J* = 37.0, 5.8 Hz).

**HRMS (ESI):** m/z calcd for $[M+Na]^+$ $C_{24}H_{21}ONBrFNa$: 460.0683. Found: 460.0664.

HPLC analysis using a chiral column (Chiralpak IA 3μ column 22 ˚C, 1.0 ml/min, 95:5 hexane:isopropanol, 210 nm, $t_{major}$ = 20.022 min, $t_{minor}$ = 23.882 min, t = 14.566 min, t = 16.965 min).

(±)-**3-amide:** (*rac*)-**[Ir]** and (*rac*)-Bn-BTM

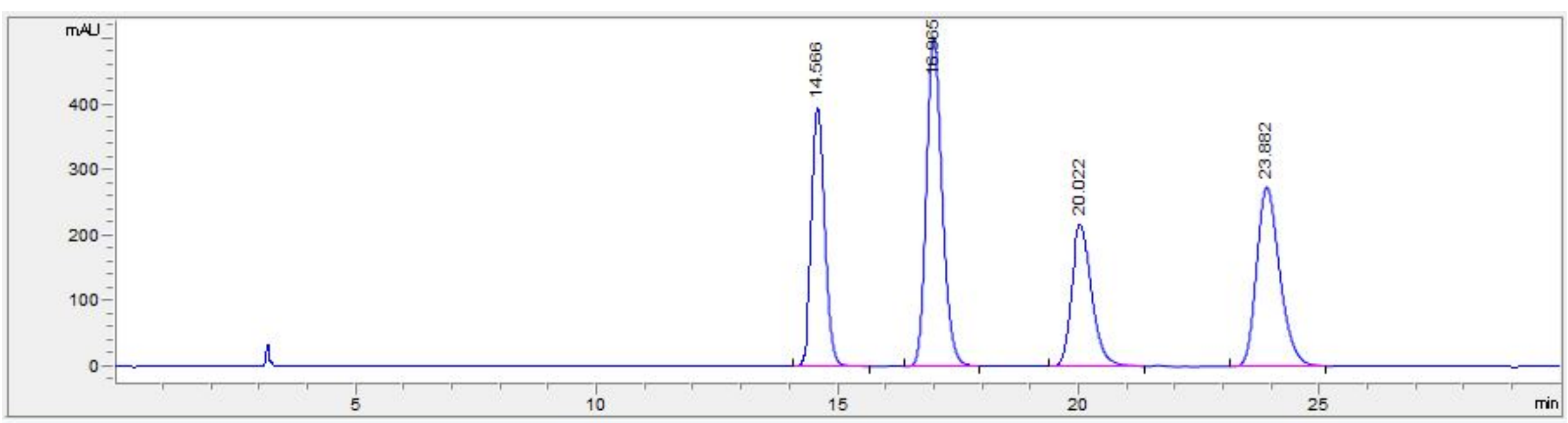


| # | Time | Area | Height | Width | Area% | Symmetry |
|---|---|---|---|---|---|---|
| 1 | 14.566 | 7409.9 | 395.5 | 0.2886 | 21.701 | 0.839 |
| 2 | 16.965 | 11424.7 | 502.6 | 0.3492 | 33.458 | 0.796 |
| 3 | 20.022 | 6194.1 | 217.2 | 0.4348 | 18.140 | 0.756 |
| 4 | 23.882 | 9117.1 | 273.3 | 0.5112 | 26.701 | 0.75 |

(*R*,*S*)-**3-amide:** ($S_a$,*S*,*S*)-**[Ir]** and (*S*)-Me-BTM

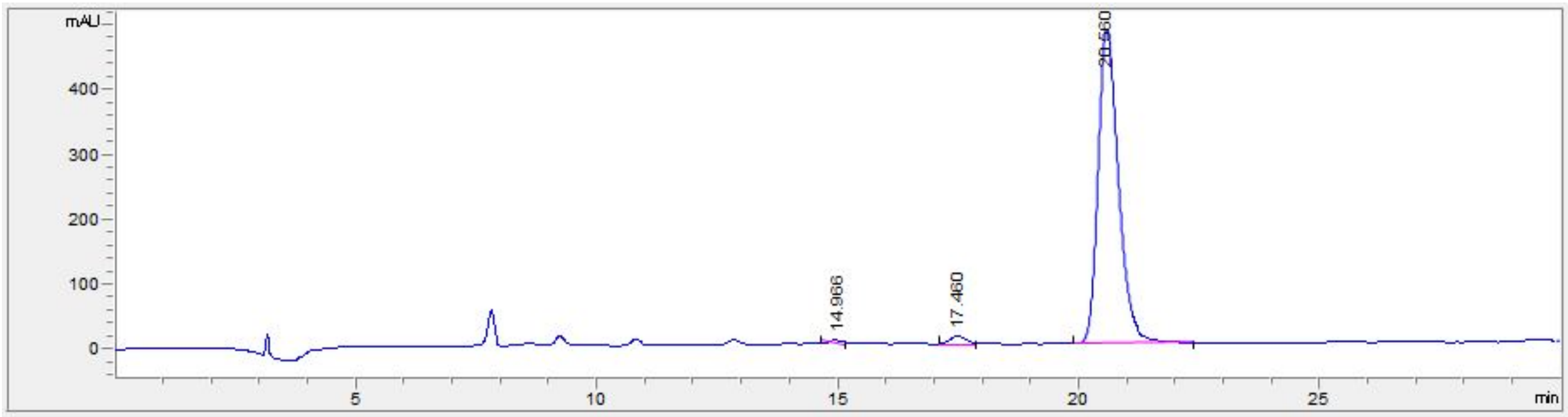


| # | Time | Area | Height | Width | Area% | Symmetry |
|---|---|---|---|---|---|---|
| 1 | 14.966 | 97.3 | 5.2 | 0.3114 | 0.655 | 0.359 |
| 2 | 17.46 | 386.7 | 14 | 0.4606 | 2.603 | 0.851 |
| 3 | 20.56 | 14369.5 | 485.2 | 0.4515 | 96.742 | 0.695 |

**Perfluorophenyl (2*S*,3*R*)-2-fluoro-2,3-diphenylpent-4-enoate (*S*,*R*)-3):** Prepared

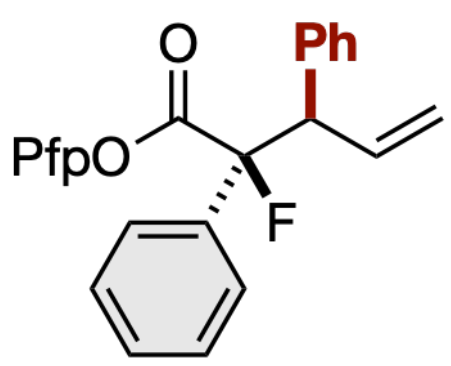


according to General Procedure B using pentafluorophenyl ester **1a** (32 mg, 0.1 mmol, 1 equiv.), *t*-butyl carbonate **2a** (35.1 mg, 0.15 mmol, 1.5 equiv.), ($R_a$,*R*,*R*)-**[Ir]** (4.2 mg, 4 mol%), (*R*)-Me-BTM (3.8 mg, 20 mol%) and DIPEA (22.6 µL, 0.13 mmol, 1.3 equiv.) in 1,4-dioxane (0.3 ml, 0.3 M). The *title compound* was obtained (38.4 mg, 0.088 mmol, 88%) as a white solid following purification by column chromatography [$SiO_2$, 0.25% Ethyl Acetate in Petroleum Ether]. The enantiomeric ratio (>99:1 *er*) of the corresponding para-bromobenzylamide amide was determined by chiral HPLC in comparison with the racemate. Diastereomeric ratio (95:5) was determined by $^{19}$F-NMR analysis of the crude reaction mixture.

**IR (film):** 3065 (w), 3032 (w), 1800 (m), 1781 (m), 1639 (w), 1602 (w), 1518 (s), 1471 (w), 1196 (m), 1008 (s), 997 (s), 740 (m), 696 (m) $cm^{-1}$.

**$^1$H NMR (500 MHz, $CDCl_3$):** δ 7.73 – 7.67 (m, 2H), 7.53 – 7.45 (m, 4H), 7.45 – 7.41 (m, 1H), 7.38 (t, *J* = 7.4 Hz, 2H), 7.34 – 7.31 (m, 1H), ****6.44 – 6.33 (m),*** 5.98 (ddd, *J* = 17.1, 10.3, 7.9 Hz, 1H), ****5.42 – 5.34 (m)***, 5.07 (d, *J* = 10.2 Hz, 1H), 4.91 (d, *J* = 17.2 Hz, 1H), 4.42 (dd, *J* = 33.5, 8.0, 1H).

**$^{13}$C NMR (126 MHz, $CDCl_3$):** δ 166.0 (d, *J* = 28.6 Hz), 137.6, 135.7 (d, *J* = 22.6 Hz), 133.9 (d, *J* = 5.0 Hz), 129.6 (d, *J* = 3.1 Hz), 129.4, 128.9, 128.8, 128.0, 125.3 (d, *J* = 10.2 Hz), 119.9, 99.2 (d, *J* = 201.6 Hz), 57.0 (d, *J* = 18.8 Hz).

**$^{19}$F NMR (376 MHz, $CDCl_3$):** δ -151.61 – -151.84 (m, 2F), -157.24 (t, *J* = 21.7 Hz, 1F), -161.93 – -162.30 (m, 2F), -173.51 (d, *J* = 33.8 Hz, 1F).

**HRMS (EI):** m/z calcd for $[M]^+$ $C_{23}H_{14}F_6O_2$: 436.0898. Found: 436.0897.

**(2*S*,3*R*)-N-(4-bromobenzyl)-2-fluoro-2,3-diphenylpent-4-enamide (*S*,*R*)-3-amide):**

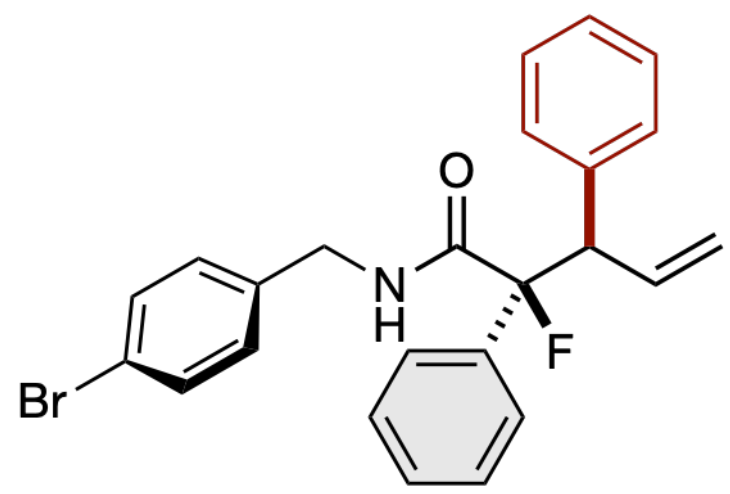

Pentafluorophenyl ester (*S*,*R*)-**3** (48.9 mg, 0.11 mmol, 1 equiv.) was dissolved in THF (1.1 ml, 0.1 M) in a 1-dram vial equipped with stirrer bar. 4-Bromobenzylamine (18.4 μl, 0.14 mmol, 1.3 equiv.) and diisopropylethylamine (28.7 μl, 0.16 mmol, 1.5 equiv.) were added and the reaction mixture was stirred at room temperature. After 1 hour, the reaction mixture was diluted with EtOAc and transferred to a separatory funnel. The organic layer was washed sequentially with 1N HCl (10 ml), $Na_2CO_3$ (2M aq.) (3 x 10mL) and brine (10 ml). The organic layer was dried with anhydrous $Na_2SO_4$, filtered and the solvent was removed under reduced pressure. The title compound was obtained (46.1 mg, 0.105 mmol, 95%) as white solid following purification by column chromatography [$SiO_2$, 5% to 30% Ethyl Acetate in Petroleum Ether]. The enantiomeric ratio (>99:1 *er*) was determined by chiral HPLC in comparison with the racemate.

**IR (film):** 3411 (w), 2922 (w), 1661 (s), 1523 (s), 1486 (m), 1447 (m), 737 (s) $cm^{-1}$.

**$^1$H NMR (500 MHz, $CDCl_3$):** δ 7.79 – 7.66 (m, 2H), 7.46 – 7.39 (m, 4H), 7.39 – 7.31 (m, 4H), 7.25 – 7.21 (m, 2H), 6.51 – 6.38 (m, 3H), 5.96 (ddd, *J* = 17.1, 10.4, 8.2 Hz, 1H), 4.99 (dt, *J* = 10.3, 1.2 Hz, 1H), 4.87 (dt, *J* = 17.1, 1.4 Hz, 1H), 4.51 – 4.36 (m, 2H), 3.78 (dd, *J* = 15.3, 4.2 Hz, 1H).

**$^{13}$C NMR (126 MHz, $CDCl_3$):** δ 169.6 (d, *J* = 21.6 Hz), 138.5, 137.4 (d, *J* = 22.0 Hz), 136.5, 134.5 (d, *J* = 4.7 Hz), 131.6, 130.2 (d, *J* = 2.3 Hz), 128.9, 128.6, 128.4 (d, *J* = 2.5 Hz), 127.4, 125.1 (d, *J* = 11.3 Hz), 121.2, 119.4, 101.3 (d, *J* = 198.3 Hz), 56.8 (d, *J* = 18.6 Hz), 42.2.

**$^{19}$F NMR (376 MHz, $CDCl_3$):** δ -176.01 (dd, *J* = 36.8, 5.7 Hz, 1F).

**HRMS (ESI):** m/z calcd for $[M+Na]^+$ $C_{24}H_{21}ONBrFNa$: 460.0683. Found: 460.0683.

HPLC analysis using a chiral column (Chiralpak IA 3µ column 22 ˚C, 1.0 ml/min, 95:5 hexane:isopropanol, 210 nm, $t_{major}$ = 23.882 min, $t_{minor}$ = 20.022 min, t = 14.566 min, t = 16.965 min).

(+)-**3-amide:** (*rac*)-**[Ir]** and (*rac*)-Bn-BTM

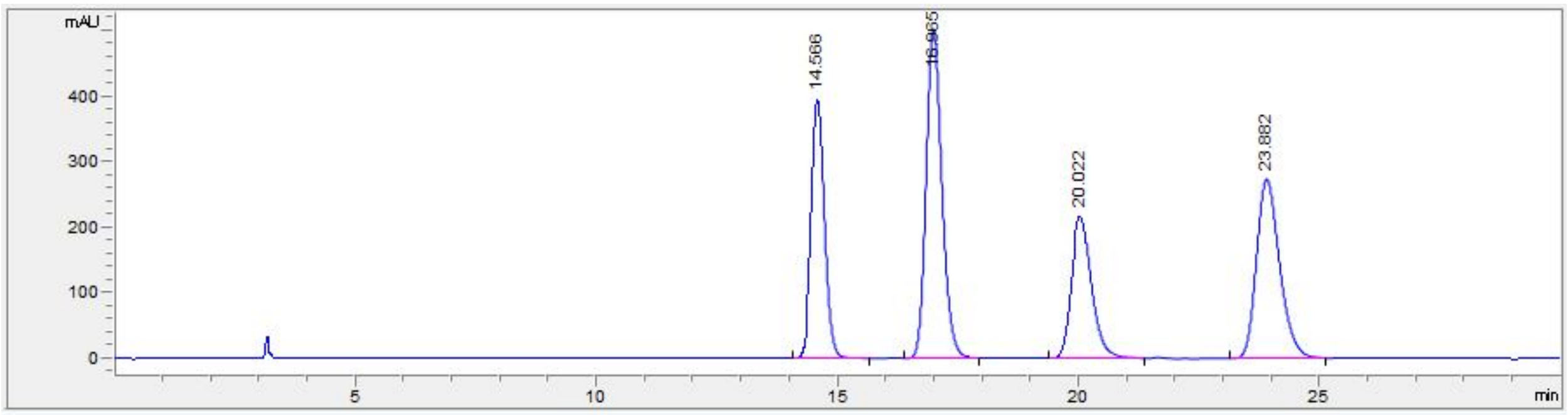


| # | Time | Area | Height | Width | Area% | Symmetry |
|---|---|---|---|---|---|---|
| 1 | 14.566 | 7409.9 | 395.5 | 0.2886 | 21.701 | 0.839 |
| 2 | 16.965 | 11424.7 | 502.6 | 0.3492 | 33.458 | 0.796 |
| 3 | 20.022 | 6194.1 | 217.2 | 0.4348 | 18.140 | 0.756 |
| 4 | 23.882 | 9117.1 | 273.3 | 0.5112 | 26.701 | 0.75 |

(*S*,*R*)-**3-amide:** ($R_a$,*R*,*R*)-**[Ir]** and (*R*)-Me-BTM

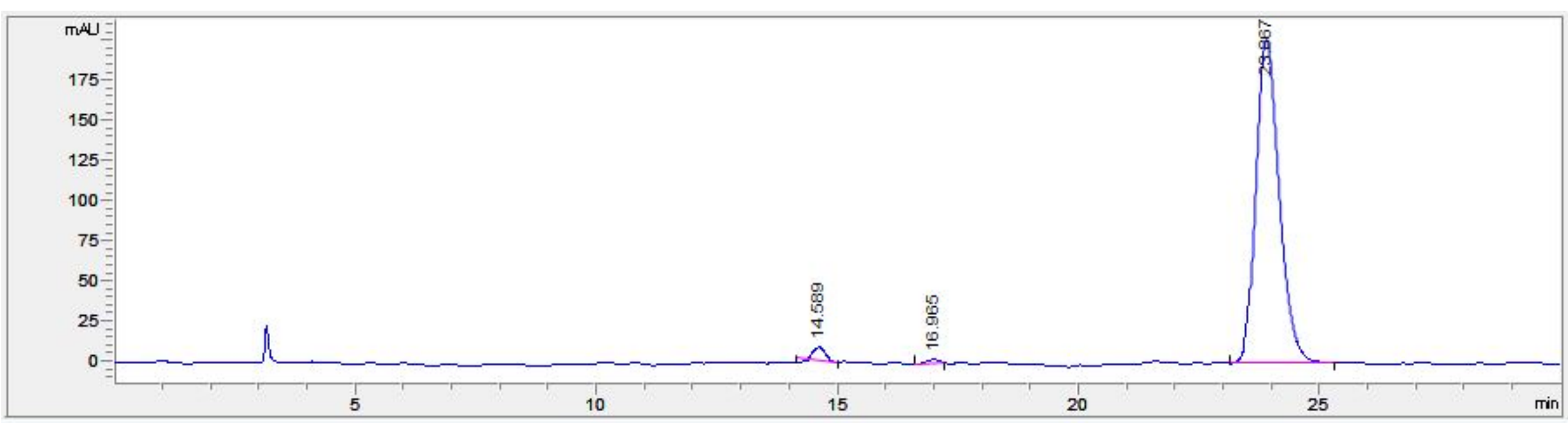


| # | Time | Area | Height | Width | Area% | Symmetry |
|---|---|---|---|---|---|---|
| 1 | 14.589 | 154.1 | 8.8 | 0.2926 | 2.192 | 0.727 |
| 2 | 16.965 | 60.2 | 2.8 | 0.3555 | 0.856 | 1.512 |
| 3 | 23.867 | 6815.4 | 203.4 | 0.5169 | 96.952 | 0.746 |

**Perfluorophenyl (2*S*,3*S*)-2-fluoro-2,3-diphenylpent-4-enoate (*S*,*S*)-3):** Prepared

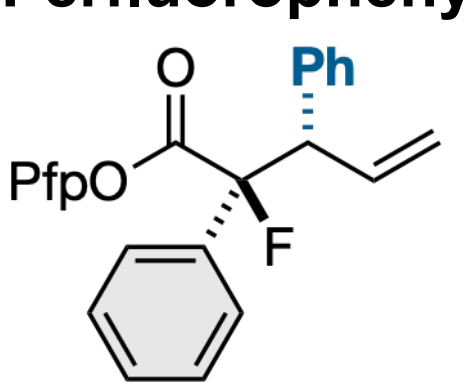


according to General Procedure B using pentafluorophenyl ester **1a** (32 mg, 0.1 mmol, 1 equiv.), *t*-butyl carbonate **2a** (35.1 mg, 0.15 mmol, 1.5 equiv.), ($S_a$,*S*,*S*)-**[Ir]** (4.2 mg, 4 mol%), (*R*)-Me-BTM (3.8 mg, 20 mol%) and DIPEA (22.6 µL, 0.13 mmol, 1.3 equiv.) in 1,4-dioxane (0.3 ml, 0.3 M). The *title compound* was obtained (39 mg, 0.089 mmol, 89%) as a colorless oil following purification by column chromatography [$SiO_2$, 0.25% Ethyl Acetate in Petroleum

Ether]. The enantiomeric ratio (>99:1 *er*) of the corresponding para-bromobenzylamide was determined by chiral HPLC in comparison with the racemate. Diastereomeric ratio (98:2) was determined by $^{19}$F-NMR analysis of the crude reaction mixture.

**IR (film):** 3065 (w), 3032 (w), 1782 (m), 1639 (w), 1602 (w), 1519 (s), 1472 (w), 1203 (m), 1181 (m), 1010 (s), 998 (s) cm$^{-1}$.

**$^{1}$H NMR (500 MHz, $CDCl_3$):** δ 7.46 – 7.40 (m, 2H), 7.31 – 7.22 (m, 3H), 7.18 – 7.09 (m, 5H), 6.40 (ddd, *J* = 17.1, 10.3, 8.8 Hz, 1H), 5.40 (d, *J* = 17.1 Hz, 1H), 5.36 (d, *J* = 9.8 Hz, 1H), 4.41 (dd, *J* = 33.0, 9.0, 1H).

**$^{13}$C NMR (126 MHz, $CDCl_3$):** δ 166.5 (d, *J* = 28.2 Hz), 136.6, 135.1 (d, *J* = 22.5 Hz), 134.5 (d, *J* = 5.2 Hz), 129.6 (d, *J* = 2.3 Hz), 129.0, 128.5 (d, *J* = 2.1 Hz), 128.3, 127.4, 125.0 (d, *J* = 9.8 Hz), 119.9, 99.8 (d, *J* = 201.4 Hz), 57.5 (d, *J* = 19.0 Hz).

**$^{19}$F NMR (376 MHz, $CDCl_3$):** δ -151.36 – -151.56 (m, 2F), -156.98 (t, *J* = 21.7 Hz, 1F), -161.72 – -162.01 (m, 2F), -174.58 (d, *J* = 33.0 Hz, 1F).

**HRMS (EI):** m/z calcd for $[M]^+$ $C_{23}H_{14}F_6O_2$: 436.0898. Found: 436.0897.

**(2*S*,3*S*)-N-(4-bromobenzyl)-2-fluoro-2,3-diphenylpent-4-enamide (*S*,*S*)-3-amide):**

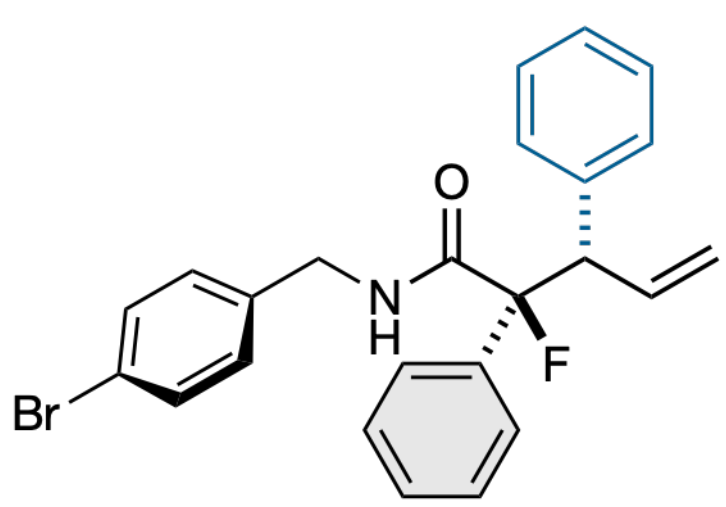


Pentafluorophenyl ester (*S*,*S*)-**3** (42.7 mg, 0.1 mmol, 1 equiv.) was dissolved in THF (1 ml, 0.1 M) in a 1-dram vial equipped with stirrer bar. 4-Bromobenzylamine (16.4 µl, 0.13 mmol, 1.3 equiv.) and diisopropylethylamine (26 µl, 0.15 mmol, 1.5 equiv.) were added and the reaction mixture was stirred at room temperature. After 1 hour, the reaction mixture was diluted with EtOAc and transferred to a separatory funnel. The organic layer was washed sequentially with 1N HCl (10 ml), $Na_2CO_3$ (2M aq.) (3 x 10mL) and brine (10 ml). The organic layer was dried with anhydrous $Na_2SO_4$, filtered and the solvent was removed under reduced pressure. The title compound was obtained (42.5 mg, 0.1 mmol, 100%) as white solid following purification by column chromatography [$SiO_2$, 5% to 30% Ethyl Acetate in Petroleum Ether]. The enantiomeric ratio (>99:1 *er*) was determined by chiral HPLC in comparison with the racemate.

**IR (film):** 3364 (br), 3061 (w), 3031 (w), 2924 (w), 1659 (s), 1523 (s), 1488 (m), 1449 (m), 714 (m), 696 (m) $cm^{-1}$.

**$^{1}$H NMR (500 MHz, $CDCl_3$):** δ 7.47 – 7.38 (m, 4H), 7.22 – 7.15 (m, 3H), 7.14 – 7.04 (m, 7H), 6.79 (q, *J* = 6.0 Hz, 1H), 6.26 (ddd, *J* = 17.1, 10.3, 8.6 Hz, 1H), 5.24 (d, *J* = 17.1 Hz, 1H), 5.19 (dd, *J* = 10.3, 1.6 Hz, 1H), 4.55 (dd, *J* = 15.1, 6.6 Hz, 1H), 4.41 (dd, *J* = 34.5, 8.5 Hz, 1H), 4.24 (dd, *J* = 15.1, 5.3 Hz, 1H).

**$^{13}$C NMR (126 MHz, $CDCl_3$):** δ 170.0 (d, *J* = 21.5 Hz), 137.8, 137.1 (d, *J* = 22.1 Hz), 136.9, 135.3 (d, *J* = 5.6 Hz), 131.9, 129.6 (d, *J* = 2.2 Hz), 129.6, 128.1, 128.1, 128.00(d, *J* = 2.0 Hz), 126.9, 124.9 (d, *J* = 10.5 Hz), 121.6, 119.2, 101.7 (d, *J* = 196.7 Hz), 57.4 (d, *J* = 19.6 Hz), 42.8.

**$^{19}$F NMR (376 MHz, $CDCl_3$):** δ -173.21 (d, *J* = 34.4 Hz, 1H).

**HRMS (ESI):** m/z calcd for $[M+Na]^+$ $C_{24}H_{21}ONBrFNa$: 460.0683. Found: 460.0682.

HPLC analysis using a chiral column (Chiralpak IA 3μ column 22 ˚C, 1.0 ml/min, 95:5 hexane:isopropanol, 210 nm, $t_{major}$ = 14.566 min, $t_{minor}$ = 16.965 min, t = 20.022 min, t = 23.882 min).

(<u>+</u>)-**3-amide:** (*rac*)-**[Ir]** and (*rac*)-Bn-BTM

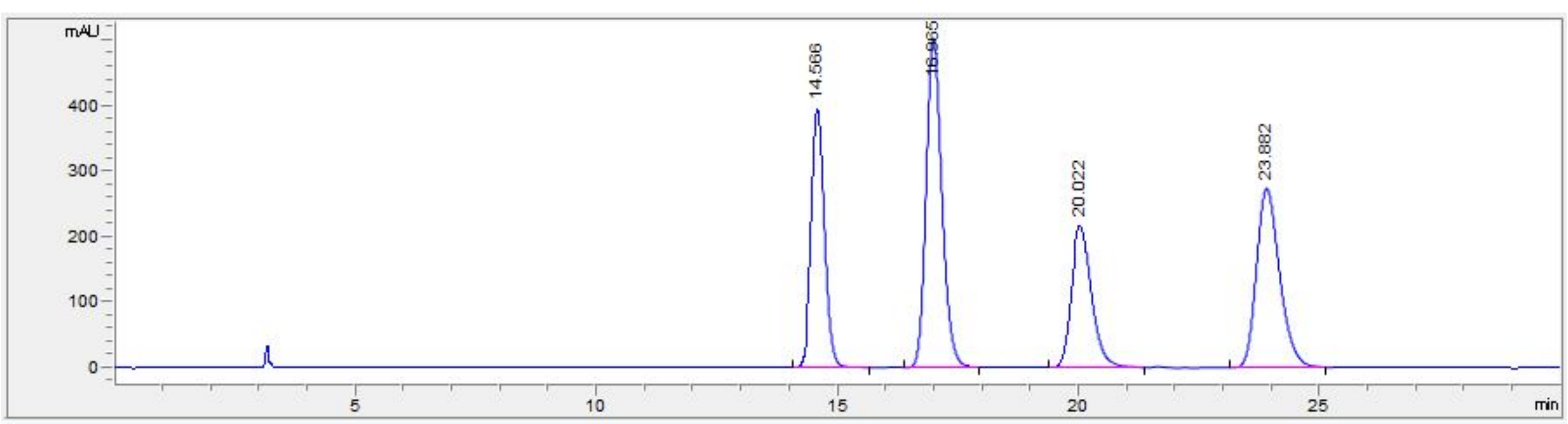


| # | Time | Area | Height | Width | Area% | Symmetry |
|---|---|---|---|---|---|---|
| 1 | 14.566 | 7409.9 | 395.5 | 0.2886 | 21.701 | 0.839 |
| 2 | 16.965 | 11424.7 | 502.6 | 0.3492 | 33.458 | 0.796 |
| 3 | 20.022 | 6194.1 | 217.2 | 0.4348 | 18.140 | 0.756 |
| 4 | 23.882 | 9117.1 | 273.3 | 0.5112 | 26.701 | 0.75 |

($S$,$S$)-**3-amide:** ($S_a$,$S$,$S$)-**[Ir]** and ($R$)-Me-BTM

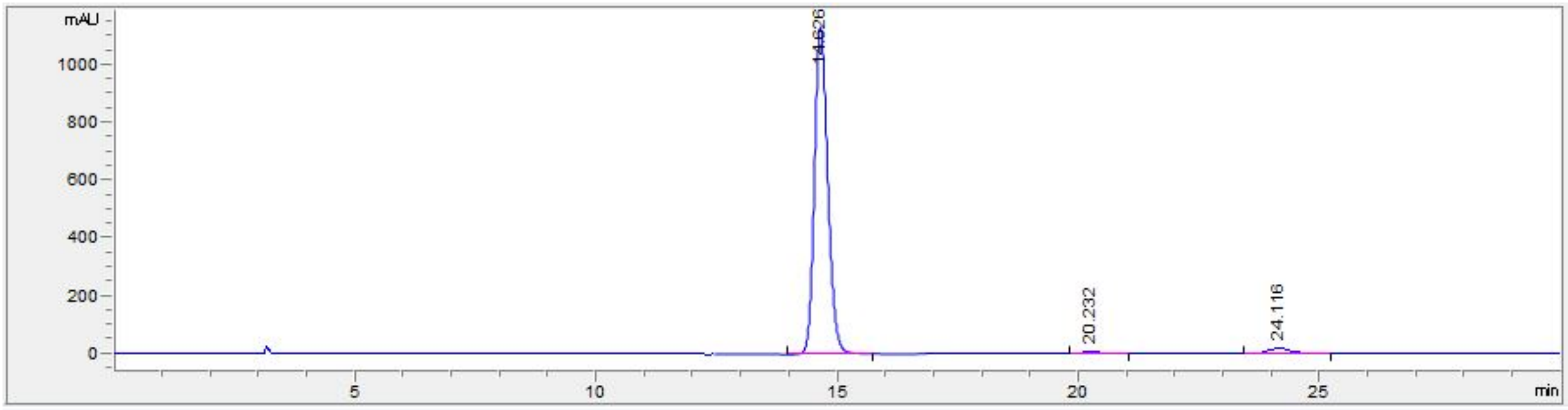

| # | Time | Area | Height | Width | Area% | Symmetry |
|---|---|---|---|---|---|---|
| 1 | 14.626 | 21759.8 | 1133.5 | 0.296 | 96.147 | 0.786 |
| 2 | 20.232 | 225.9 | 8 | 0.4348 | 0.998 | 0.726 |
| 3 | 24.116 | 646.1 | 18.6 | 0.5192 | 2.855 | 0.815 |

**Perfluorophenyl (2*R*,3*R*)-2-fluoro-3-phenyl-2-(p-tolyl)pent-4-enoate (*R*,*R*)-7):**

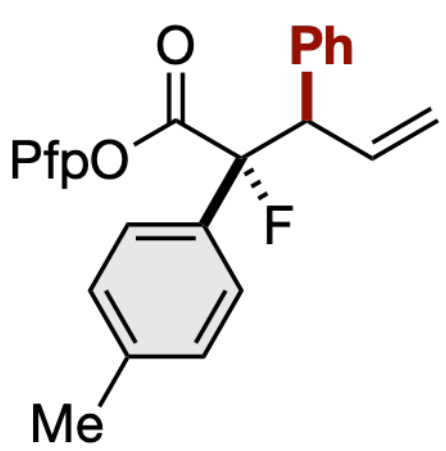

Prepared according to General Procedure B using pentafluorophenyl ester **1b** (33.4 mg, 0.1 mmol, 1 equiv.), *t*-butyl carbonate **2a** (35.1 mg, 0.15 mmol, 1.5 equiv.), ($R_a$,$R$,$R$)-**[Ir]** (4.2 mg, 4 mol%), ($S$)-Me-BTM (3.8 mg, 20 mol%) and DIPEA (22.6 µL, 0.13 mmol, 1.3 equiv.) in 1,4-dioxane (0.3 ml, 0.3 M). The *title compound* was obtained (32.2 mg, 0.0715 mmol, 71.5%) as a colorless oil following purification by column chromatography [$SiO_2$, 0.25% Ethyl Acetate in Petroleum Ether]. The enantiomeric ratio (>99:1 *er*) of the corresponding primary amide was determined by chiral HPLC in comparison with the racemate. Diastereomeric ratio (97:3) was determined by $^{19}$F-NMR analysis of the crude reaction mixture.

**IR (film):** 3032 (w), 2927 (w), 1782 (m), 1639 (w), 1603 (w), 1518 (s), 1471 (w), 1455 (w), 1202 (m), 1179 (m), 1009 (s), 996 (s), 797 (m), 698 (s) cm$^{-1}$.

**$^1$H NMR (500 MHz, $CDCl_3$):** δ 7.30 (d, $J$ = 8.4 Hz, 2H), 7.17 – 7.12 (m, 5H), 7.06 (d, $J$ = 8.0 Hz, 2H), 6.38 (ddd, $J$ = 17.1, 10.3, 8.8 Hz, 1H), 5.38 (d, $J$ = 17.1 Hz, 1H), 5.34 (dd, $J$ = 10.3, 1.3 Hz, 1H), 4.40 (dd, $J$ = 33, 8.5 Hz, 1H).

**$^{13}$C NMR (126 MHz, $CDCl_3$):** δ 166.6 (d, $J$ = 28.4 Hz), 138.8, 136.8, 134.7 (d, $J$ = 5.2 Hz), 132.1 (d, $J$ = 22.6 Hz), 129.7 (d, $J$ = 2.5 Hz), 129.2 (d, $J$ = 1.8 Hz), 128.3, 127.3, 125.0 (d, $J$ = 9.6 Hz), 119.8, 99.9 (d, $J$ = 200.8 Hz), 57.4 (d, $J$ = 18.9 Hz), 21.2.

**$^{19}$F NMR (376 MHz, CDCl$_3$):** δ -151.12 – -151.54 (m, 2F), -157.08 (t, *J* = 21.6 Hz, 1F), -161.51 – -162.08 (m, 2F), -173.94 (d, *J* = 33.0 Hz, 1F).

**HRMS (EI):** m/z calcd for $[M]^+$ $C_{24}H_{16}F_6O_2$: 450.1054. Found: 450.1058.

**(2*R*,3*R*)-2-Fluoro-3-phenyl-2-(p-tolyl)pent-4-enamide (*R*,*R*)-7-NH$_2$):**

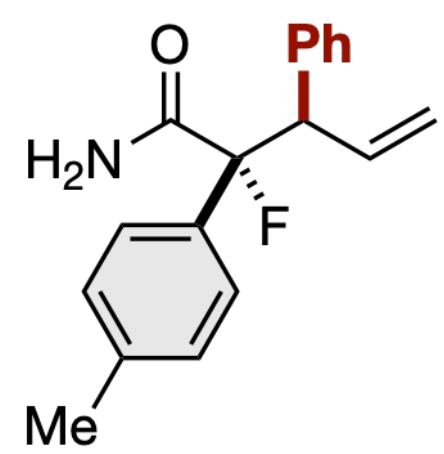


**IR (film):** 3429 (m), 3179 (br), 3088 (w), 3033 (w), 1667 (s), 1602 (w), 1453 (w), 1396 (m) cm$^{-1}$.

**$^{1}$H NMR (500 MHz, CDCl$_3$):** δ 7.32 – 7.27 (m, 2H), 7.16 – 7.07 (m, 5H), 6.99 (d, *J* = 8.0 Hz, 2H), 6.40 (s, 1H), 6.28 (ddd, *J* = 16.5, 10.7, 8.5 Hz, 1H), 5.51 (s, 1H), 5.34 – 5.18 (m, 2H), 4.37 (dd, *J* = 35, 8.5 Hz, 1H), 2.23 (s, 3H).

**$^{13}$C NMR (126 MHz, CDCl$_3$):** δ 172.7 (d, *J* = 22.9 Hz), 137.9, 137.8, 135.4 (d, *J* = 5.4 Hz), 133.9 (d, *J* = 22.4 Hz), 129.7 (d, *J* = 2.3 Hz), 128.7 (d, *J* = 1.9 Hz), 128.1, 126.9, 124.8 (d, *J* = 10.2 Hz), 119.1, 101.7 (d, *J* = 196.9 Hz), 56.9 (d, *J* = 19.4 Hz), 21.1.

**$^{19}$F NMR (376 MHz, CDCl$_3$):** δ -170.85 (d, *J* = 34.6 Hz, 1F).

**HRMS (APCI):** m/z calcd for $[M+H]^+$ $C_{18}H_{19}ONF$: 284.1445. Found: 284.1445.

HPLC analysis using a chiral column (Chiralpak IB 3μ column 22 ˚C, 0.5 ml/min, 95:5 hexane:isopropanol, 210 nm, $t_{major}$ = 19.118 min, $t_{minor}$ = 24.148 min, t = 22.882 min, t = 26.837 min).

(±)-**7:** (*rac*)-**[Ir]** and (*rac*)-Bn-BTM

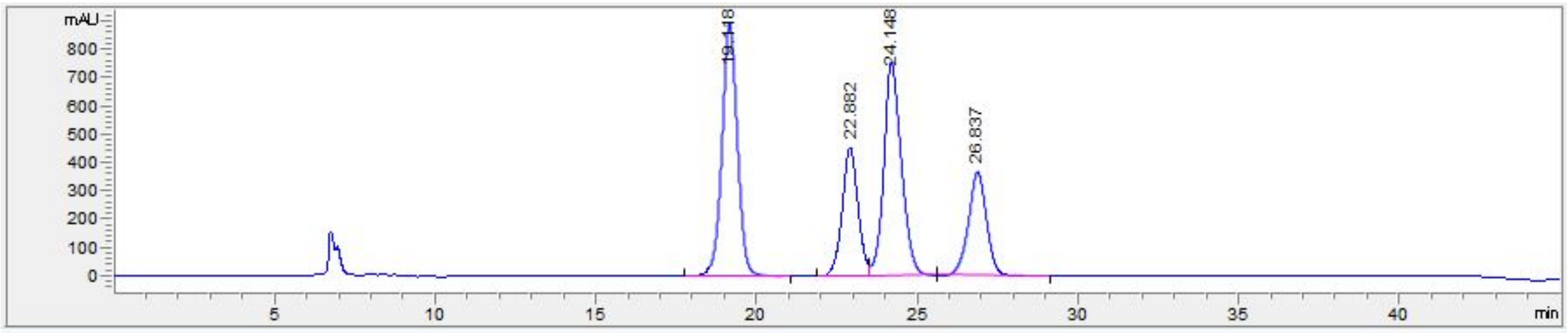


| # | Time | Area | Height | Width | Area% | Symmetry |
|---|---|---|---|---|---|---|
| 1 | 19.118 | 29786.1 | 893.1 | 0.5111 | 33.814 | 0.924 |
| 2 | 22.882 | 15303.9 | 451.7 | 0.5113 | 17.374 | 0.938 |
| 3 | 24.148 | 28552.2 | 755.7 | 0.5668 | 32.413 | 0.713 |
| 4 | 26.837 | 14445.4 | 368.4 | 0.5874 | 16.399 | 1.019 |

***(R,R)*-7:** ($R_a$,*R*,*R*)-**[Ir]** and (*S*)-Me-BTM

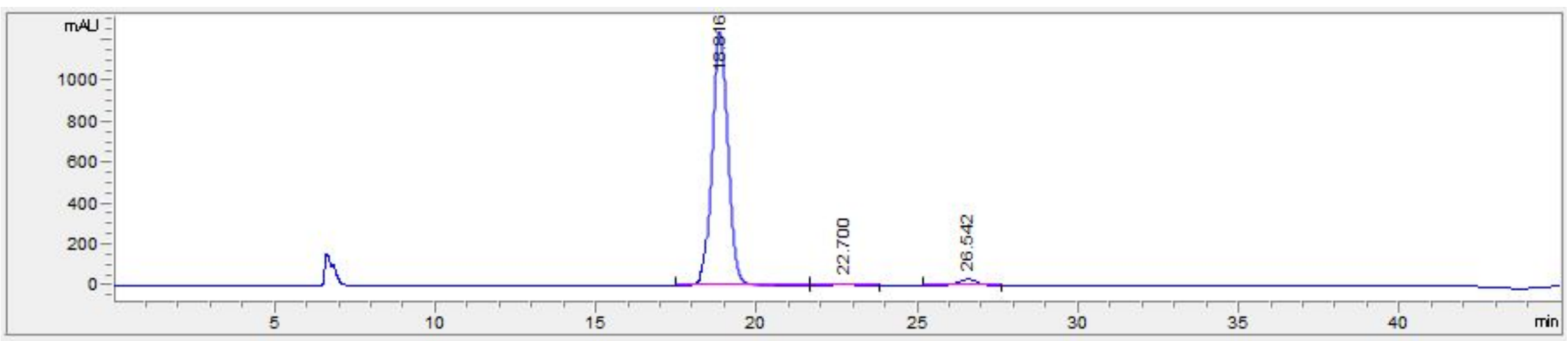


| # | Time | Area | Height | Width | Area% | Symmetry |
|---|---|---|---|---|---|---|
| 1 | 18.816 | 42610.2 | 1253 | 0.5187 | 96.616 | 0.876 |
| 2 | 22.7 | 292.4 | 7.8 | 0.552 | 0.663 | 1.111 |
| 3 | 26.542 | 1200.1 | 30.5 | 0.5954 | 2.721 | 1.149 |

**Perfluorophenyl (2*R*,3*S*)-2-fluoro-3-phenyl-2-(p-tolyl)pent-4-enoate (*R*,*S*)-7):**

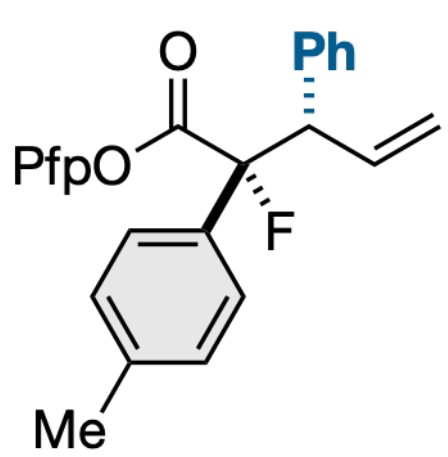


Prepared according to General Procedure B using pentafluorophenyl ester **1b** (33.4 mg, 0.1 mmol, 1 equiv.), *t*-butyl carbonate **2a** (35.1 mg, 0.15 mmol, 1.5 equiv.), ($S_a$,*S*,*S*)-**[Ir]** (4.2 mg, 4 mol%), (*S*)-Me-BTM (3.8 mg, 20 mol%) and DIPEA (22.6 µL, 0.13 mmol, 1.3 equiv.) in 1,4-dioxane (0.3 ml, 0.3 M). The *title compound* was obtained (35.4 mg, 0.079 mmol, 79%) as a colorless oil following purification by column chromatography [$SiO_2$, 0.25% Ethyl Acetate in Petroleum Ether]. The enantiomeric ratio (>99:1 *er*) of the corresponding primary amide was determined by chiral HPLC in comparison with the racemate. Diastereomeric ratio (93:7) was determined by $^{19}$F-NMR analysis of the crude reaction mixture.

**IR (film):** 3033 (w), 2927 (w), 1780 (w), 1781 (w), 1640 (w), 1603 (w), 1519 (s), 1455 (w), 1185 (m), 1010 (s), 998 (s), 700 (m) $cm^{-1}$.

**$^1$H NMR (500 MHz, $CDCl_3$):** δ 7.50 (d, *J* = 8.4 Hz, 2H), 7.44 – 7.38 (m, 2H), 7.29 (dd, *J* = 8.3, 6.5 Hz, 2H), 7.26 – 7.22 (m, 1H), 7.21 – 7.17 (m, 2H), ***6.30 (ddd, J = 17.1, 10.2, 8.8 Hz)***, 5.91 (ddd, *J* = 17.1, 10.3, 7.8 Hz, 1H), ***5.30 (d, J = 17.1 Hz)***, ***5.26 (d, J = 10.3 Hz)**, 5.00 (d, *J* = 10.2 Hz, 1H), 4.85 (d, *J* = 17.1 Hz, 1H), 4.33 (dd, *J* = 34.0, 7.5 Hz, 1H), 2.32 (s, 3H), ***2.19 (s)***.

**$^{13}$C NMR (126 MHz, $CDCl_3$):** δ 166.1 (d, *J* = 28.8 Hz), 139.3, 137.7, 134.0 (d, *J* = 5.0 Hz), 132.8 (d, *J* = 22.9 Hz), 129.6 (d, *J* = 3.2 Hz), 129.6 (d, *J* = 2.7 Hz), 128.8, 127.9, 125.3 (d, *J* = 10.1 Hz), 119.8, 99.3 (d, *J* = 200.9 Hz), 56.9 (d, *J* = 18.8 Hz), 21.3.

**$^{19}$F NMR (376 MHz, $CDCl_3$):** δ -151.54 – -151.86 (m, 2F), -157.35 (t, *J* = 21.7 Hz, 1F), -162.06 – -162.37 (m, 2F), -173.15 (d, *J* = 33.8 Hz, 1F).

**HRMS (EI):** m/z calcd for $[M]^+$ $C_{24}H_{16}F_6O_2$: 450.1054. Found: 450.1060.

**(2*R*,3*S*)-2-Fluoro-3-phenyl-2-(p-tolyl)pent-4-enamide ((*R*,*S*)-7-$NH_2$):**

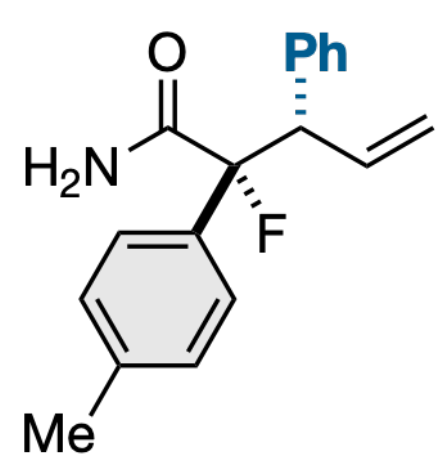


**IR (film):** 3436 (m), 3172 (br), 1667 (s), 1602 (w), 1513 (w), 1452 (w), 1402 (w) $cm^{-1}$.

**$^1$H NMR (500 MHz, $CDCl_3$):** δ 7.58 – 7.50 (m, 2H), 7.46 – 7.40 (m, 2H), 7.37 – 7.30 (m, 2H), 7.29 – 7.25 (m, 1H), 7.21 (d, *J* = 8.0 Hz, 2H), 6.02 (s, 1H), 5.96 (ddd, *J* = 17.1, 10.4, 8.2 Hz, 1H), ***5.29 – 5.20 (m)***, 5.05 (s, 1H), 5.00 (dt, *J* = 10.3, 1.2 Hz, 1H), 4.88 (dt, *J* = 17.1, 1.4 Hz, 1H), 4.38 (dd, *J* = 36.5, 8.5 Hz, 1H), 2.36 (s, 3H), ***2.23 (s).***

**$^{13}$C NMR (126 MHz, $CDCl_3$):** δ 172.2 (d, *J* = 23.2 Hz), 138.6, 138.2, 134.7 (d, *J* = 4.5 Hz), 134.4 (d, *J* = 22.4 Hz), 130.0 (d, *J* = 2.3 Hz), 129.1 (d, *J* = 1.8 Hz), 128.4, 127.3, 125.1 (d, *J* = 10.7 Hz), 119.3, 101.2 (d, *J* = 198.3 Hz), 56.3 (d, *J* = 18.8 Hz), 21.2.

**$^{19}$F NMR (376 MHz, $CDCl_3$):** δ -172.85 (d, *J* = 36.2 Hz, 1F).

**HRMS (APCI):** m/z calcd for $[M+H]^+$ $C_{18}H_{19}ONF$: 284.1445. Found: 284.1445.

HPLC analysis using a chiral column (Chiralpak IB 3μ column 22 ˚C, 0.5 ml/min, 95:5 hexane:isopropanol, 210 nm, $t_{major}$ = 26.837 min, $t_{minor}$ = 22.882 min, t = 19.118 min, t = 24.148 min).

(<u>+</u>)-**7:** (*rac*)-**[Ir]** and (*rac*)-Bn-BTM

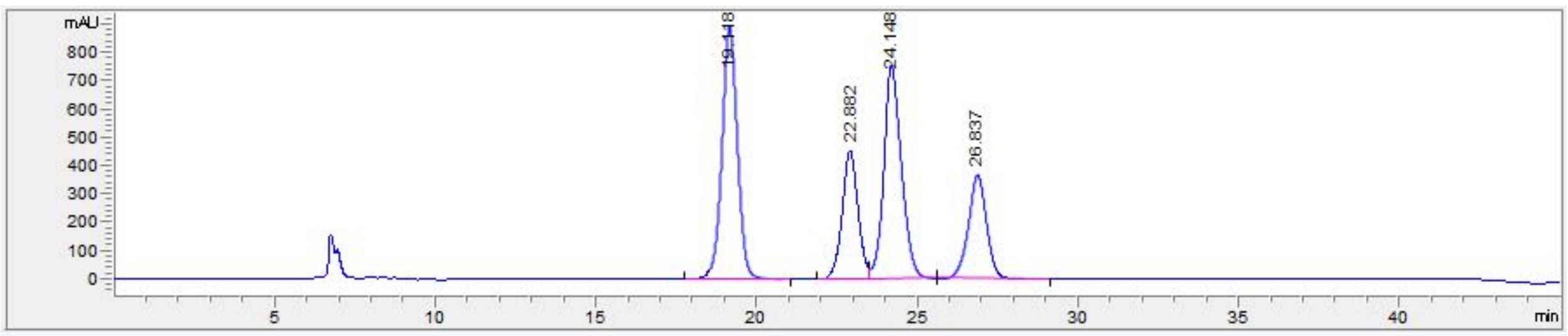


| # | Time | Area | Height | Width | Area% | Symmetry |
|---|---|---|---|---|---|---|
| 1 | 19.118 | 29786.1 | 893.1 | 0.5111 | 33.814 | 0.924 |
| 2 | 22.882 | 15303.9 | 451.7 | 0.5113 | 17.374 | 0.938 |
| 3 | 24.148 | 28552.2 | 755.7 | 0.5668 | 32.413 | 0.713 |
| 4 | 26.837 | 14445.4 | 368.4 | 0.5874 | 16.399 | 1.019 |

(*R*,*S*)-**7:** (*S<sub>a</sub>,S,S*)-**[Ir]** and (*S*)-Me-BTM

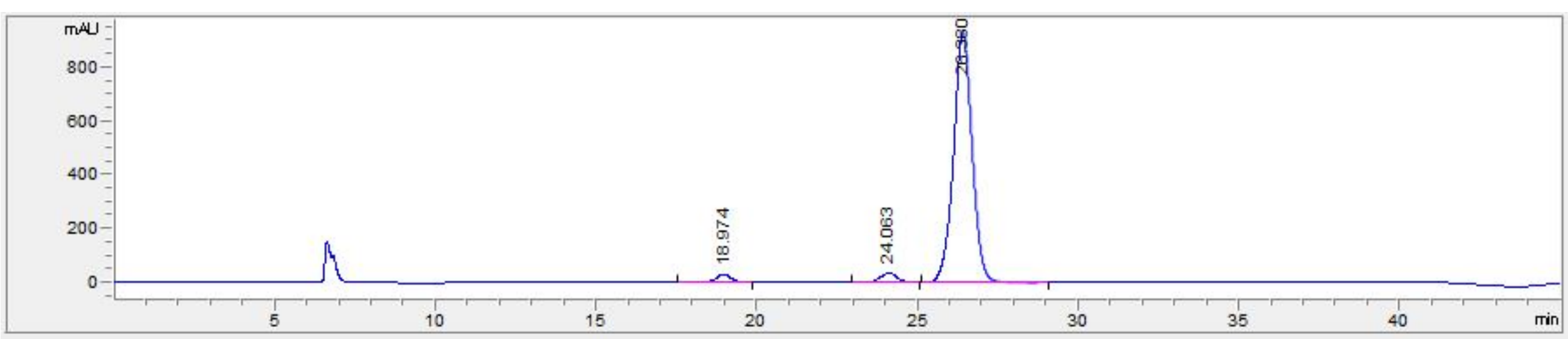


| # | Time | Area | Height | Width | Area% | Symmetry |
|---|---|---|---|---|---|---|
| 1 | 18.974 | 1042.4 | 30.2 | 0.5224 | 2.703 | 1.224 |
| 2 | 24.063 | 1291.9 | 35.6 | 0.5496 | 3.350 | 1.044 |
| 3 | 26.38 | 36227.8 | 929.9 | 0.5805 | 93.947 | 0.939 |

**Perfluorophenyl (2*R*,3*R*)-2-fluoro-2-(4-fluorophenyl)-3-phenylpent-4-enoate (*R*,*R*)-9:**

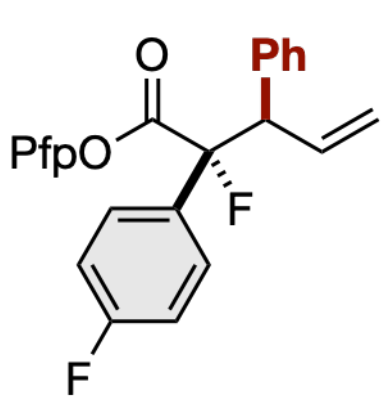


Prepared according to General Procedure B using pentafluorophenyl ester **1c** (33.8 mg, 0.1 mmol, 1 equiv.), *t*-butyl carbonate **2a** (35.1 mg, 0.15 mmol, 1.5 equiv.), ($R_a$,*R*,*R*)-**[Ir]** (4.2 mg, 4 mol%), (*S*)-Me-BTM (3.8 mg, 20 mol%) and DIPEA (22.6 μL, 0.13 mmol, 1.3 equiv.) in 1,4-dioxane (0.3 ml, 0.3 M). The *title compound* was obtained (37.3 mg, 0.0082 mmol, 82%) as a white solid following purification by column chromatography [$SiO_2$, 0.25% Ethyl Acetate in Petroleum Ether]. The enantiomeric ratio (>99:1 *er*) of the corresponding primary amide

was determined by chiral HPLC in comparison with the racemate. Diastereomeric ratio (98:2) was determined by $^{19}$F-NMR analysis of the crude reaction mixture.

**IR (film):** 3065 (w), 3032 (w), 1783 (m), 1639 (w), 1604 (w), 1518 (s), 1510 (s), 1455 (w), 1203 (m), 1182 (m), 1010 (s), 996 (s), 809 (m), 699 (s) cm$^{-1}$

**$^1$H NMR (500 MHz, CDCl$_3$):** δ 7.44 – 7.36 (m, 2H), 7.21 – 7.08 (m, 5H), 6.95 (t, *J* = 8.6 Hz, 2H), 6.37 (ddd, *J* = 17.1, 10.3, 8.8 Hz, 1H), 5.43 – 5.31 (m, 2H), 4.36 (dd, *J* = 33.0, 9.0 Hz, 1H).

**$^{13}$C NMR (126 MHz, CDCl$_3$):** δ 166.4 (d, *J* = 27.9 Hz), 163.0 (d, *J* = 248.9 Hz), 136.4, 134.3 (d, *J* = 5.3 Hz), 131.0 (dd, *J* = 23.0, 3.2 Hz), 129.6 (d, *J* = 2.3 Hz), 128.5, 127.5, 127.1 (dd, *J* = 10.0, 8.4 Hz), 120.1, 115.6 (dd, *J* = 21.5, 1.7 Hz), 99.6 (d, *J* = 202.0 Hz), 57.7 (d, *J* = 19.0 Hz).

**HRMS (EI):** m/z calcd for $[M]^+$ $C_{23}H_{13}F_7O_2$: 454.0804. Found: 454.0801.

**$^{19}$F NMR (376 MHz, CDCl$_3$):** δ -112.56 (ddd, *J* = 13.6, 8.5, 5.0 Hz, 1F), -151.48 – -151.66 (m, 2F), -156.73 (t, *J* = 21.6 Hz, 1F), -161.58 – -161.82 (m, 2F), -173.70 (d, *J* = 33.1 Hz, 1F).

**(2*R*,3*R*)-2-Fluoro-2-(4-fluorophenyl)-3-phenylpent-4-enamide ((*R*,*R*)-9-NH$_2$):**

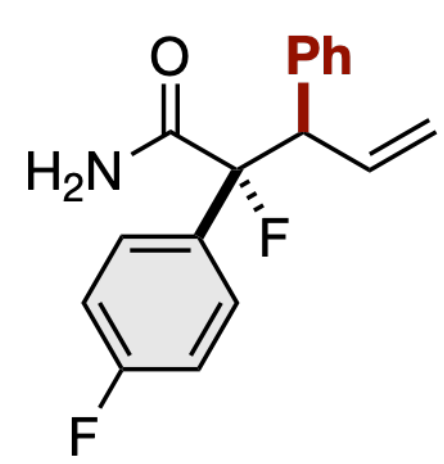


**IR (film):** 3433 (m), 3184 (br), 1670 (s), 1608 (w), 1509 (m), 1452 (w), 1415 (w), 1395 (w) cm$^{-1}$.

**$^1$H NMR (500 MHz, CDCl$_3$):** δ 7.45 – 7.33 (m, 2H), 7.20 – 7.08 (m, 3H), 7.06 (dt, *J* = 7.7, 1.6 Hz, 2H), 6.86 (t, *J* = 8.6 Hz, 2H), 6.42 (s, 1H), 6.33 – 6.22 (m, 1H), 5.51 (s, 1H), 5.34 – 5.18 (m, 2H), 4.32 (dd, *J* = 35.0, 8.5 Hz, 1H).

**$^{13}$C NMR (126 MHz, CDCl$_3$):** δ 172.4 (d, *J* = 22.6 Hz), 162.6 (d, *J* = 246.8 Hz), 137.5, 134.9 (d, *J* = 5.4 Hz), 132.7 (dd, *J* = 22.5, 3.2 Hz), 129.6 (d, *J* = 2.2 Hz), 128.2, 127.1,

126.8 (dd, $J$ = 10.5, 8.3 Hz), 119.4, 114.9 (dd, $J$ = 21.7, 1.8 Hz), 101.3 (d, $J$ = 197.6 Hz), 57.3 (d, $J$ = 19.4 Hz).

**$^{19}$F NMR (376 MHz, $CDCl_3$):** δ -114.3 (ddd, $J$ = 13.8, 8.7, 5.3 Hz, 1F), -170.8 (d, $J$ = 34.9 Hz, 1F).

**HRMS (APCI):** m/z calcd for $[M+H]^+$ $C_{17}H_{16}ONF_2$: 288.1194. Found: 288.1195.

HPLC analysis using a chiral column (Chiralpak IB 3μ column 22 ˚C, 0.5 ml/min, 95:5 hexane:isopropanol, 210 nm, $t_{major}$ = 19.467 min, $t_{minor}$ = 24.351 min, t = 26.533 min, t = 30.53 min).

(±)-**9:** (*rac*)-**[Ir]** and (*rac*)-Bn-BTM

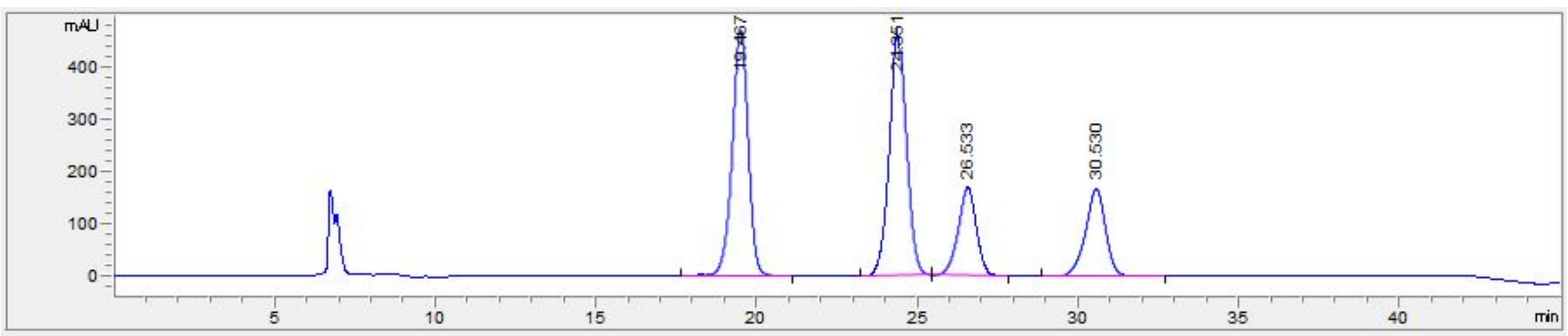


| # | Time | Area | Height | Width | Area% | Symmetry |
|---|---|---|---|---|---|---|
| 1 | 19.467 | 16352 | 471.8 | 0.5303 | 34.212 | 1.079 |
| 2 | 24.351 | 17536.7 | 470.8 | 0.5666 | 36.690 | 0.93 |
| 3 | 26.533 | 6584.2 | 168.4 | 0.5963 | 13.775 | 1.083 |
| 4 | 30.53 | 7323.6 | 167.9 | 0.6528 | 15.323 | 1.145 |

(*R*,*R*)-**9:** (*R$_a$*,*R*,*R*)-**[Ir]** and (*S*)-Me-BTM

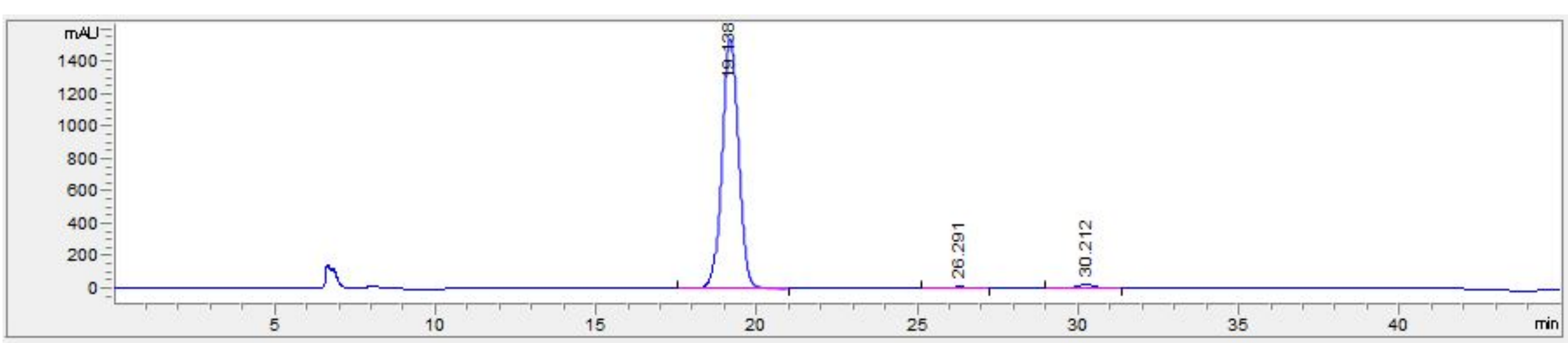


| # | Time | Area | Height | Width | Area% | Symmetry |
|---|---|---|---|---|---|---|
| 1 | 19.138 | 55230 | 1556.6 | 0.552 | 97.381 | 0.909 |
| 2 | 26.291 | 439.1 | 11 | 0.5786 | 0.774 | 1.209 |
| 3 | 30.212 | 1046.3 | 24.4 | 0.6327 | 1.845 | 1.208 |

**Perfluorophenyl (2*R*,3*S*)-2-fluoro-2-(4-fluorophenyl)-3-phenylpent-4-enoate ((*R,S*)-9):**

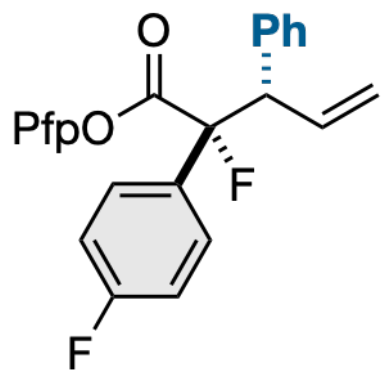


Prepared according to General Procedure B using pentafluorophenyl ester **1c** (33.8 mg, 0.1 mmol, 1 equiv.), *t*-butyl carbonate **2a** (35.1 mg, 0.15 mmol, 1.5 equiv.) and ($S_a$,*S*,*S*)-**[Ir]** catalyst. The *title compound* was obtained (32.8 mg, 0.072 mmol, 72%) as a white solid following purification by column chromatography [$SiO_2$, 0.25% Ethyl Acetate in Petroleum Ether]. The enantiomeric ratio (>99:1 *er*) of the corresponding primary amide was determined by chiral HPLC in comparison with the racemate. Diastereomeric ratio (93:7) was determined by $^{19}$F-NMR analysis of the crude reaction mixture.

**IR (film):** 3065 (w), 3034 (w), 1800 (w), 1781 (m), 1640 (w), 1604 (w), 1518 (s), 1510 (s), 1197 (m), 1009 (s), 997 (s), 700 (m) cm$^{-1}$.

**$^{1}$H NMR (500 MHz, $CDCl_3$):** δ 7.72 – 7.63 (m, 2H), 7.50 – 7.43 (m, 2H), 7.40 – 7.31 (m, 3H), 7.16 (t, *J* = 8.5 Hz, 2H), ***6.37 (ddd, J = 17.0, 10.3, 8.7 Hz)***, 5.97 (ddd, *J* = 17.1, 10.3, 8.1 Hz, 1H), ***5.44 – 5.32 (m)***, 5.09 (d, *J* = 10.4 Hz, 1H), 4.93 (d, *J* = 17.1 Hz, 1H), 4.37 (dd, *J* = 34.0, 8.0 Hz, 1H).

**$^{13}$C NMR (126 MHz, $CDCl_3$):** δ 165.9 (d, *J* = 28.6 Hz), 163.3 (d, *J* = 250.5 Hz), 137.3, 133.6 (d, *J* = 5.0 Hz), 131.6 (dd, *J* = 23.1, 3.3 Hz), 129.5 (d, *J* = 3.0 Hz), 128.9, 128.1, 127.4 (dd, *J* = 10.4, 8.5 Hz), 120.1, 115.9 (dd, *J* = 21.8, 1.9 Hz), 99.0 (d, *J* = 201.9 Hz), 57.2 (d, *J* = 18.8 Hz).

**$^{19}$F NMR (376 MHz, $CDCl_3$):** δ -112.24 (tt, *J* = 8.7, 4.9 Hz, 1F), -151.73 – -151.95 (m, 2F), -157.00 (t, *J* = 21.7 Hz, 1F), -161.85 – -162.05 (m, 2F), -172.66 (d, *J* = 33.5 Hz, 1F).

**HRMS (EI):** m/z calcd for $[M]^+$ $C_{23}H_{13}F_7O_2$: 454.0804. Found: 454.0797.

**(2*R*,3*S*)-2-Fluoro-2-(4-fluorophenyl)-3-phenylpent-4-enamide ((*R*,*S*)-9-NH2):**

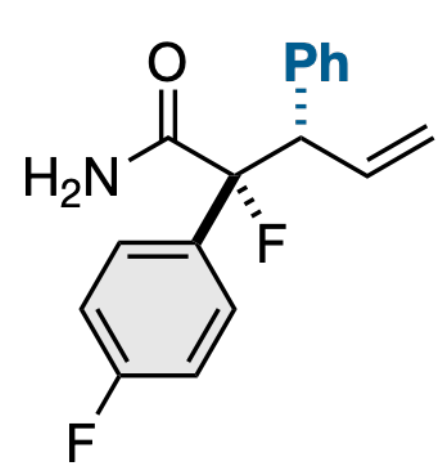

**IR (film):** 3441 (m), 3176 (br), 1671 (s), 1604 (w), 1508 (m), 1452 (w), 1414 (w) $cm^{-1}$.

**$^{1}$H NMR (500 MHz, $CDCl_3$):** δ 7.70 – 7.62 (m, 2H), 7.44 – 7.38 (m, 2H), 7.35 – 7.30 (m, 2H), 7.30 – 7.24 (m, 1H), 7.08 (t, *J* = 8.6 Hz, 2H), ***6.42 (s)**, ***6.27 (dt, J = 17.9, 9.0 Hz)**, 6.03 (s, 1H), 5.94 (ddd, *J* = 17.1, 10.3, 8.5 Hz, 1H), ***5.47 (s)**, ***5.38 – 5.21 (m)**, 5.07 (s, 1H), 5.00 (d, *J* = 10.3 Hz, 1H), 4.86 (d, *J* = 17.1 Hz, 1H), 4.32 (dd, *J* = 36.0, 8.5 Hz, 1H).

**$^{13}$C NMR (126 MHz, $CDCl_3$)**: δ 171.9 (d, *J* = 23.3 Hz), 162.9 (d, *J* = 246.9 Hz), 138.2, 134.2 (d, *J* = 4.5 Hz), 133.1 (dd, *J* = 22.4, 3.2 Hz), 129.9 (d, *J* = 2.3 Hz), 128.5, 127.5, 127.2 (dd, *J* = 11.2, 8.2 Hz), 119.6, 115.3 (dd, *J* = 21.6, 2.2 Hz), 100.8 (d, *J* = 198.7 Hz), 56.8 (d, *J* = 18.8 Hz).

**$^{19}$F NMR (376 MHz, $CDCl_3$):** δ -114.08 (s, 1F), -172.36 (d, *J* = 35.8 Hz, 1F).

**HRMS (APCI):** m/z calcd for $[M+H]^+$ $C_{17}H_{16}ONF_2$: 288.1194. Found: 288.1194.
HPLC analysis using a chiral column (Chiralpak IB 3μ column 22 ˚C, 0.5 ml/min, 95:5 hexane:isopropanol, 210 nm, $t_{major}$ = 30.53 min, $t_{minor}$ = 26.533 min, t = 19.467 min, t = 24.351 min).

(±)-**9:** (*rac*)-**[Ir]** and (*rac*)-Bn-BTM

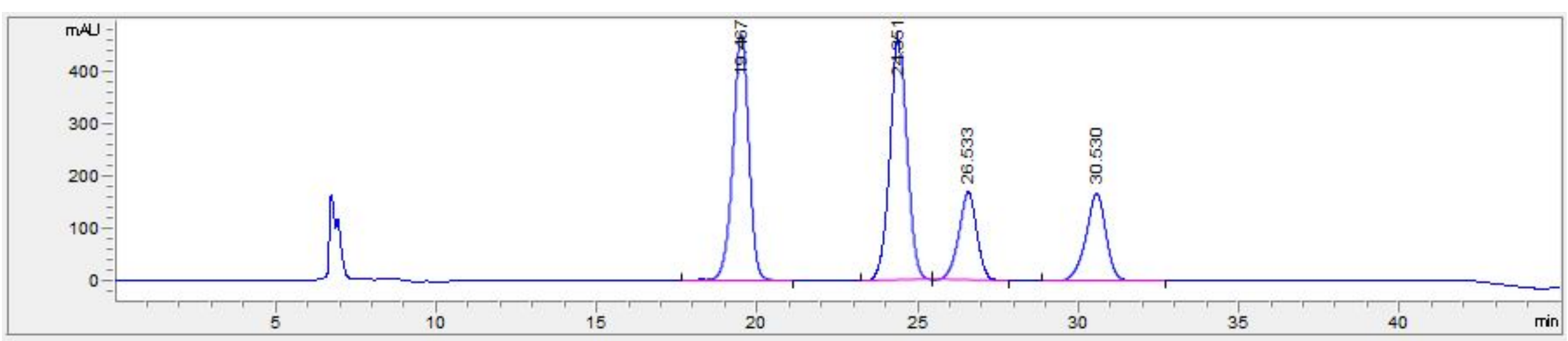


| # | Time | Area | Height | Width | Area% | Symmetry |
|---|---|---|---|---|---|---|
| 1 | 19.467 | 16352 | 471.8 | 0.5303 | 34.212 | 1.079 |
| 2 | 24.351 | 17536.7 | 470.8 | 0.5666 | 36.690 | 0.93 |
| 3 | 26.533 | 6584.2 | 168.4 | 0.5963 | 13.775 | 1.083 |
| 4 | 30.53 | 7323.6 | 167.9 | 0.6528 | 15.323 | 1.145 |

($R$,$S$)-**9:** ($S_a$,$S$,$S$)-**[Ir]** and ($S$)-Me-BTM

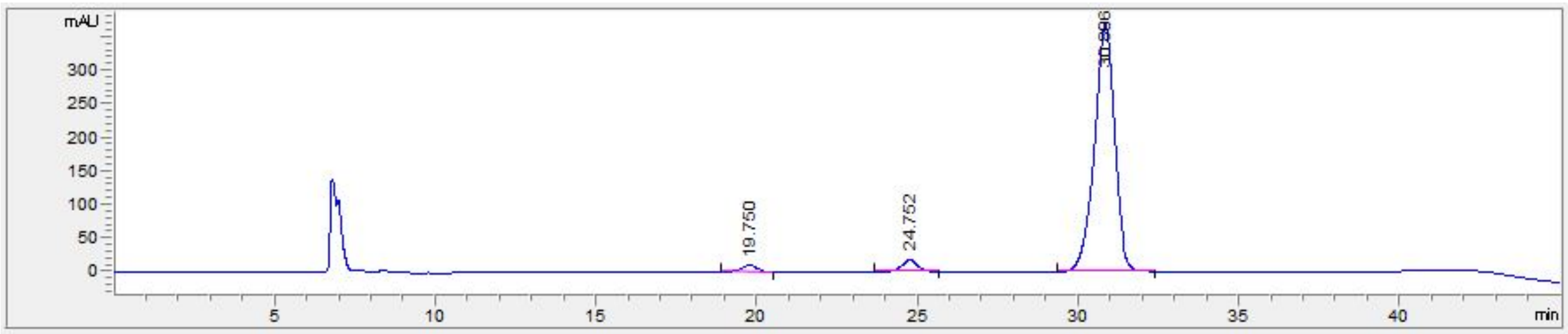


| # | Time | Area | Height | Width | Area% | Symmetry |
|---|---|---|---|---|---|---|
| 1 | 19.75 | 336.5 | 9.8 | 0.5184 | 1.968 | 1.118 |
| 2 | 24.752 | 696.2 | 18.4 | 0.576 | 4.071 | 1.115 |
| 3 | 30.806 | 16069.6 | 367.8 | 0.6557 | 93.961 | 1.103 |

**Perfluorophenyl (2$R$,3$R$)-2-(4-chlorophenyl)-2-fluoro-3-phenylpent-4-enoate ($R$,$R$)-10:**

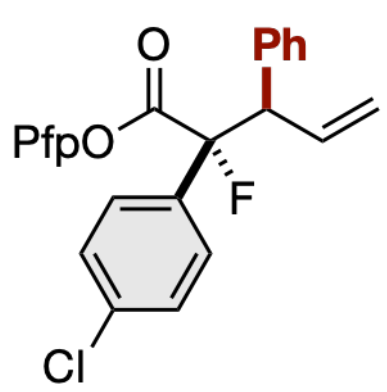


Prepared according to General Procedure B using pentafluorophenyl ester **1d** (35.4 mg, 0.1 mmol, 1 equiv.), *t*-butyl carbonate **2a** (35.1 mg, 0.15 mmol, 1.5 equiv.) and ($R_a$,$R$,$R$)-**[Ir]** catalyst. The *title compound* was obtained (38 mg, 0.081 mmol, 81%) as a colorless oil following purification by column chromatography [$SiO_2$, 0.25% Ethyl Acetate in Petroleum Ether]. The enantiomeric ratio (>99:1 *er*) of the corresponding primary amide was determined by chiral HPLC in comparison with the racemate. Diastereomeric ratio (97:3) was determined by $^{19}$F-NMR analysis of the crude reaction mixture.

**IR (film):** 3063 (w), 3033 (w), 2928 (w), 1783 (w), 1640 (w), 1597 (w), 1520 (s), 1493 (w), 1204 (w), 1182 (w), 1012 (m), 999 (m), 699 (w) cm$^{-1}$.

**$^1$H NMR (500 MHz, $CDCl_3$):** δ 7.29 (d, $J$ = 8.7 Hz, 2H), 7.22 – 7.14 (m, 2H), 7.12 – 7.01 (m, 5H), 6.29 (ddd, $J$ = 17.1, 10.2, 8.8 Hz, 1H), 5.37 – 5.22 (m, 2H), 4.27 (dd, $J$ = 33.0, 9.0 Hz, 1H).

**$^{13}$C NMR (126 MHz, $CDCl_3$):** δ 166.3 (d, $J$ = 27.9 Hz), 136.2, 135.2, 134.2 (d, $J$ = 5.1 Hz), 133.7 (d, $J$ = 23.0 Hz), 129.6 (d, $J$ = 2.3 Hz), 128.8 (d, $J$ = 2.0 Hz), 128.5, 127.6, 126.5 (d, $J$ = 9.9 Hz), 120.2, 99.5 (d, $J$ = 202.4 Hz), 57.6 (d, $J$ = 18.9 Hz).

**$^{19}$F NMR (376 MHz, $CDCl_3$):** δ -151.38 – -151.63 (m, 2F), -156.64 (t, $J$ = 21.8 Hz, 1F), -161.50 – -161.81 (m, 2F), -174.30 (d, $J$ = 33.0 Hz, 1F).

**HRMS (EI):** m/z calcd for $[M]^+$ $C_{23}H_{13}ClF_6O_2$: 470.0508. Found: 470.0506.

**(2*R*,3*R*)-2-(4-Chlorophenyl)-2-fluoro-3-phenylpent-4-enamide ((*R*,*R*)-10-NH$_2$):**

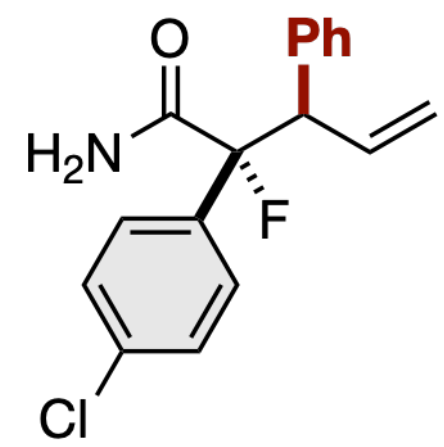

**IR (film):** 3428 (m), 3186 (br), 3088 (w), 3032 (w), 1670 (s), 1601 (w), 1491 (w), 1453 (w), 1396 (w), 698 (w) $cm^{-1}$.

**$^1$H NMR (500 MHz, $CDCl_3$):** δ 7.40 – 7.31 (m, 2H), 7.20 – 7.09 (m, 5H), 7.09 – 7.00 (m, 2H), 6.42 (s, 1H), 6.34 – 6.18 (m, 1H), 5.58 (s, 1H), 5.29 – 5.22 (m, 2H), 4.32 (dd, *J* = 35.0, 8.5 Hz, 1H).

**$^{13}$C NMR (126 MHz, $CDCl_3$):** δ 172.2 (d, *J* = 22.6 Hz), 137.3, 135.4 (d, *J* = 22.5 Hz), 134.8 (d, *J* = 5.4 Hz), 134.2, 129.6 (d, *J* = 2.2 Hz), 128.3, 128.2 (d, *J* = 1.8 Hz), 127.2, 126.4 (d, *J* = 10.4 Hz), 119.5, 101.3 (d, *J* = 198.1 Hz), 57.2 (d, *J* = 19.3 Hz).

**$^{19}$F NMR (376 MHz, $CDCl_3$):** δ -171.50 (d, *J* = 35.2 Hz, 1F).

**HRMS (ESI):** m/z calcd for $[M+Na]^+$ $C_{17}H_{15}ONClFNa$: 326.0718. Found: 326.0718.
HPLC analysis using a chiral column (Chiralpak IB 3μ column 22 ˚C, 0.5 ml/min, 95:5 hexane:isopropanol, 210 nm, $t_{major}$ = 20.135 min, $t_{minor}$ = 24.071 min, t = 26.116 min, t = 31.858 min).

(±)-**10:** (*rac*)-**[Ir]** and (*rac*)-Bn-BTM

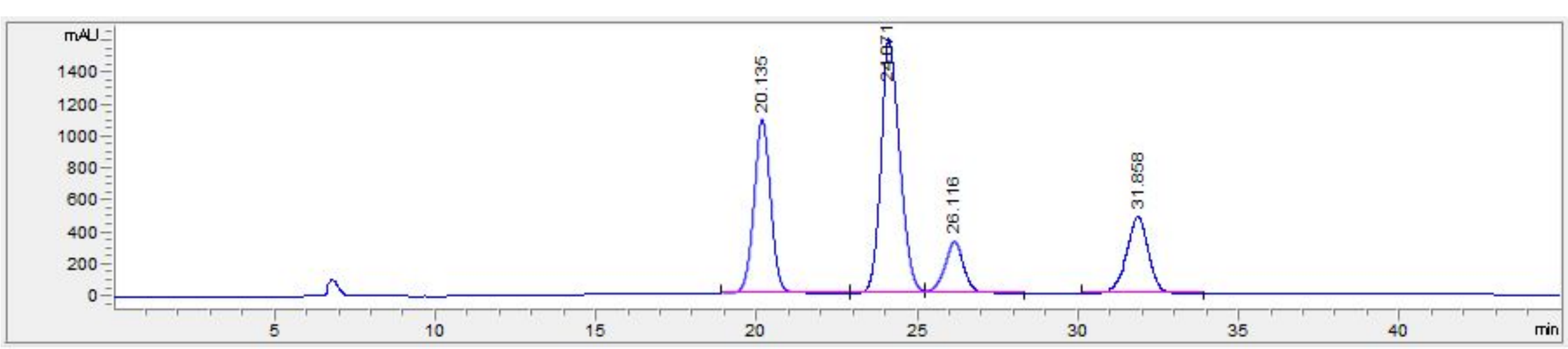


| # | Time | Area | Height | Width | Area% | Symmetry |
|---|---|---|---|---|---|---|
| 1 | 20.135 | 38625 | 1077.2 | 0.5483 | 27.783 | 0.939 |
| 2 | 24.071 | 63922.4 | 1591.7 | 0.6045 | 45.980 | 0.686 |
| 3 | 26.116 | 13598.3 | 320.8 | 0.6346 | 9.781 | 1.007 |
| 4 | 31.858 | 22876.2 | 480.1 | 0.7119 | 16.455 | 1.126 |

(*R*,*R*)-**10:** (*R*$_a$,*R*,*R*)-**[Ir]** and (*S*)-Me-BTM

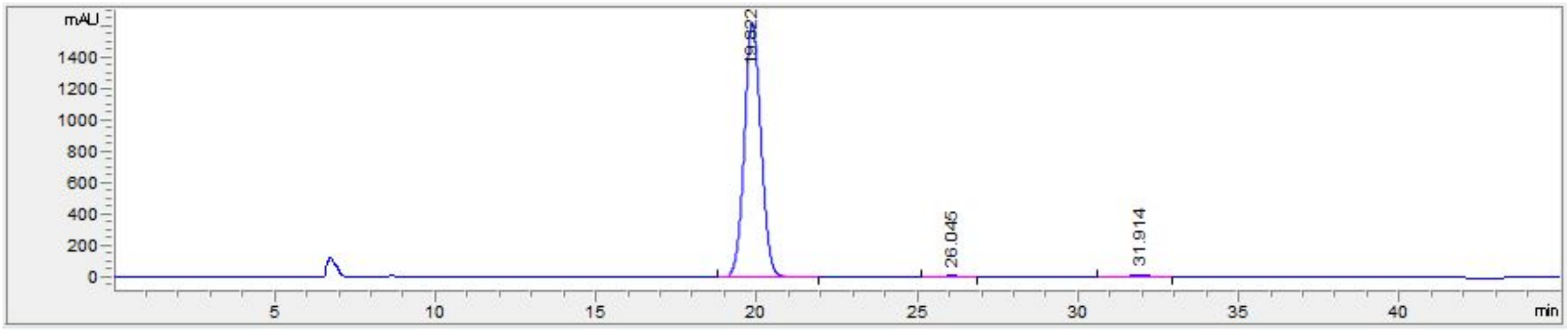


| # | Time | Area | Height | Width | Area% | Symmetry |
|---|---|---|---|---|---|---|
| 1 | 19.822 | 58400.8 | 1622.9 | 0.5578 | 98.185 | 0.863 |
| 2 | 26.045 | 405.1 | 10.4 | 0.5297 | 0.681 | 1.158 |
| 3 | 31.914 | 674.3 | 14.7 | 0.6429 | 1.134 | 1.308 |

**Perfluorophenyl (2*R*,3*S*)-2-(4-chlorophenyl)-2-fluoro-3-phenylpent-4-enoate ((*R*,*S*)-10):**

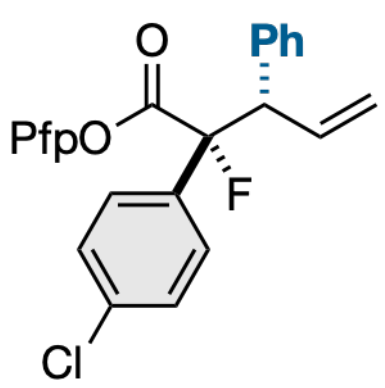


Prepared according to General Procedure B using pentafluorophenyl ester **1d** (35.4 mg, 0.1 mmol, 1 equiv.), *t*-butyl carbonate **2a** (35.1 mg, 0.15 mmol, 1.5 equiv.), (*S*$_a$,*S*,*S*)-**[Ir]** (4.2 mg, 4 mol%), (*S*)-Me-BTM (3.8 mg, 20 mol%) and DIPEA (22.6 μL, 0.13 mmol, 1.3 equiv.) in 1,4-dioxane (0.3 ml, 0.3 M). The *title compound* was obtained (35.6 mg, 0.076 mmol, 76%) as a white solid following purification by column chromatography [$SiO_2$, 0.25% Ethyl Acetate in Petroleum Ether]. The enantiomeric ratio (>99:1 *er*) of the corresponding primary amide was determined by chiral HPLC in comparison with the racemate. Diastereomeric ratio (92:8) was determined by $^{19}$F-NMR analysis of the crude reaction mixture.

**IR (film):** 3063 (w), 3033 (w), 2928 (w), 1801 (w), 1781 (m), 1640 (w), 1597 (w), 1518 (s), 1493 (m), 1198 (m), 1010 (s), 998 (s), 700 (m) cm$^{-1}$.

**$^1$H NMR (500 MHz, $CDCl_3$):** δ 7.64 (d, *J* = 8.7 Hz, 2H), 7.50 – 7.43 (m, 4H), 7.41 – 7.30 (m, 3H), ***6.36 (ddd, J = 17.0, 10.3, 8.8 Hz)**, 5.96 (ddd, *J* = 17.1, 10.3, 8.1 Hz, 1H), ***5.49 – 5.31 (m)**, 5.08 (d, *J* = 10.3 Hz, 1H), 4.93 (d, *J* = 17.1 Hz, 1H), 4.35 (dd, *J* = 33.5, 8.5 Hz, 1H).

**$^{13}$C NMR (126 MHz, $CDCl_3$):** δ 165.8 (d, *J* = 28.5 Hz), 137.2, 135.6, 134.3 (d, *J* = 23.1 Hz), 133.5 (d, *J* = 5.0 Hz), 129.5 (d, *J* = 3.1 Hz), 129.1 (d, *J* = 2.2 Hz), 128.9, 128.1, 126.8 (d, *J* = 10.4 Hz), 120.2, 98.9 (d, *J* = 202.3 Hz), 57.2 (d, *J* = 18.8 Hz).

**$^{19}$F NMR (376 MHz, $CDCl_3$):** δ -151.66 – -151.86 (m, 2F), -156.92 (t, *J* = 21.6 Hz, 1F), -161.77 – -161.97 (m, 2F), -173.36 (d, *J* = 33.7 Hz, 1F).

**HRMS (EI):** m/z calcd for $[M]^+$ $C_{23}H_{13}ClF_6O_2$: 470.0508. Found: 470.0506.

**(2*R*,3*S*)-2-(4-Chlorophenyl)-2-fluoro-3-phenylpent-4-enamide ((*R*,*S*)-10-NH$_2$):**

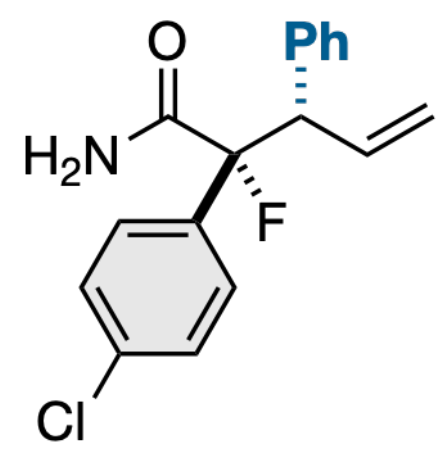

**IR (film):** 3434 (m), 3175 (br), 1668 (s), 1603 (w), 1489 (w), 1452 (w), 1398 (w), 600 (w) $cm^{-1}$.

**$^1$H NMR (500 MHz, $CDCl_3$):** δ 7.64 – 7.58 (m, 2H), 7.43 – 7.35 (m, 4H), 7.35 – 7.30 (m, 2H), 7.30 – 7.24 (m, 1H), 6.02 (s, 1H), 5.93 (ddd, *J* = 17.1, 10.4, 8.5 Hz, 1H), ***5.30 – 5.21 (m)***, 5.08 (s, 1H), 5.01 (dt, *J* = 10.3, 1.1 Hz, 1H), 4.87 (dt, *J* = 17.1, 1.4 Hz, 1H), 4.31 (dd, *J* = 35.5, 8.5 Hz, 1H).

**$^{13}$C NMR (126 MHz, $CDCl_3$):** δ 171.7 (d, *J* = 22.8 Hz), 138.1, 135.8 (d, *J* = 22.5 Hz), 134.6, 134.1 (d, *J* = 4.5 Hz), 129.9 (d, *J* = 2.3 Hz), 128.6 (d, *J* = 2.0 Hz), 128.51 127.5, 126.7 (d, *J* = 11.0 Hz), 119.7, 100.8 (d, *J* = 199.2 Hz), 56.8 (d, *J* = 18.9 Hz).

**$^{19}$F NMR (376 MHz, $CDCl_3$):** δ -173.00 (d, *J* = 35.9 Hz, 1F).

**HRMS (ESI):** m/z calcd for $[M+Na]^+$ $C_{17}H_{15}ONClFNa$: 326.0718. Found: 326.0720.

HPLC analysis using a chiral column (Chiralpak IB 3μ column 22 ˚C, 0.5 ml/min, 95:5 hexane:isopropanol, 210 nm, $t_{major}$ = 31.858 min, $t_{minor}$ = 26.116 min, t = 20.135 min, t = 24.071 min).

(±)-**10:** (*rac*)-**[Ir]** and (*rac*)-Bn-BTM

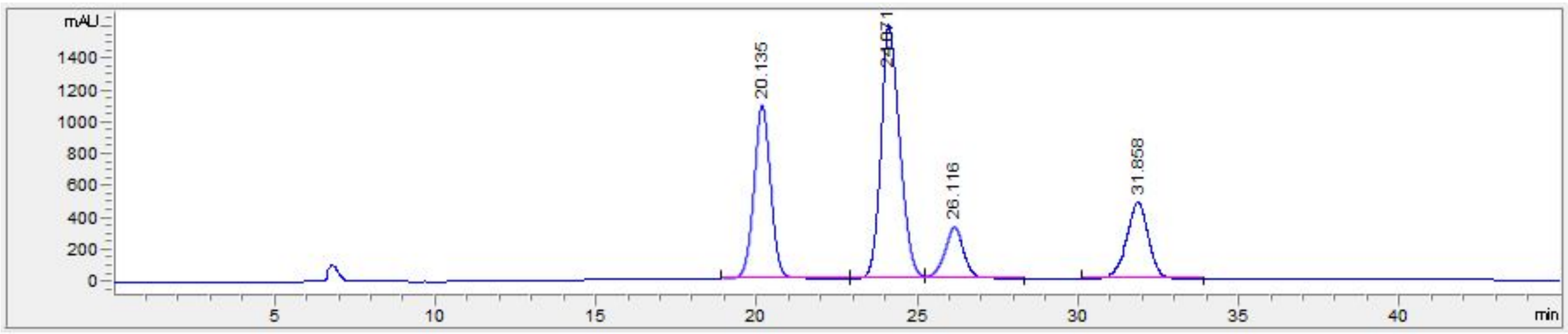

| # | Time | Area | Height | Width | Area% | Symmetry |
|---|---|---|---|---|---|---|
| 1 | 20.135 | 38625 | 1077.2 | 0.5483 | 27.783 | 0.939 |
| 2 | 24.071 | 63922.4 | 1591.7 | 0.6045 | 45.980 | 0.686 |
| 3 | 26.116 | 13598.3 | 320.8 | 0.6346 | 9.781 | 1.007 |
| 4 | 31.858 | 22876.2 | 480.1 | 0.7119 | 16.455 | 1.126 |

(*R*,*S*)-**10:** ($S_a$,*S*,*S*)-**[Ir]** and (*S*)-Me-BTM

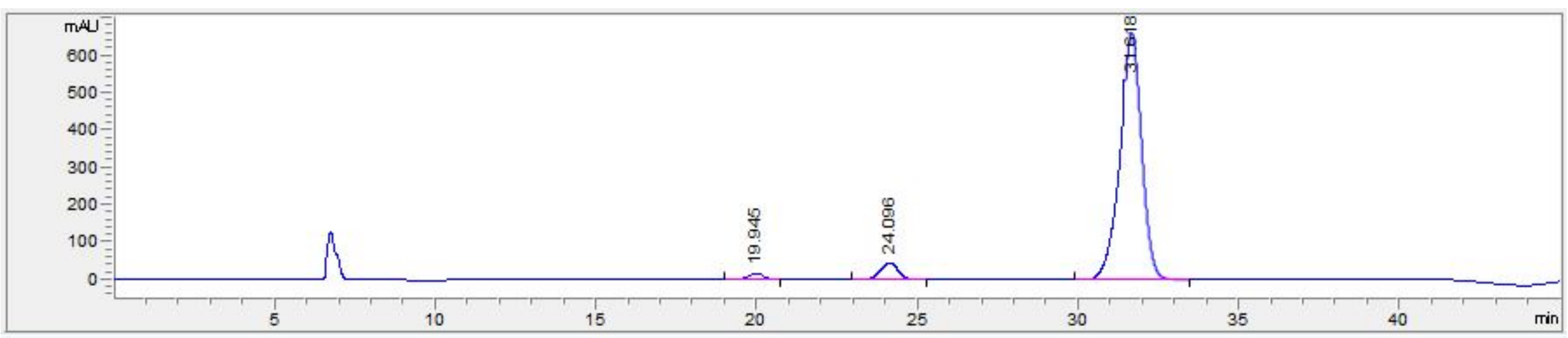

| # | Time | Area | Height | Width | Area% | Symmetry |
|---|---|---|---|---|---|---|
| 1 | 19.945 | 563.5 | 16.3 | 0.5278 | 1.688 | 1.192 |
| 2 | 24.096 | 1757.6 | 46.1 | 0.5766 | 5.264 | 1.086 |
| 3 | 31.618 | 31069.7 | 668.6 | 0.6902 | 93.049 | 1.112 |

**Perfluorophenyl (2*R*,3*R*)-2-(4-bromophenyl)-2-fluoro-3-phenylpent-4-enoate ((*R*,*R*)-11):** Prepared according to General Procedure B using pentafluorophenyl ester **1e** (39.9 mg, 0.1 mmol, 1 equiv.), *t*-butyl carbonate **2a** (35.1 mg, 0.15 mmol, 1.5 equiv.) and ($R_a$,*R*,*R*)-**[Ir]** catalyst. The *title compound* was obtained (42.8 mg, 0.083 mmol, 83%) as a colorless oil following purification by column chromatography [$SiO_2$, 0.25% Ethyl Acetate in Petroleum Ether]. The enantiomeric ratio (>99:1 *er*) of the corresponding primary amide was determined by chiral HPLC in comparison with the racemate. Diastereomeric ratio (97:3) was determined by $^{19}$F-NMR analysis of the crude reaction mixture.

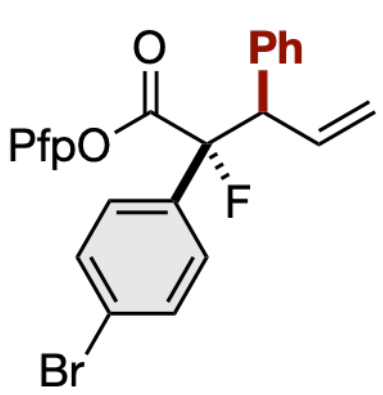


**IR (film):** 3065 (w), 3032 (w), 1782 (m), 1640 (w), 1591 (w), 1518 (s), 1489 (m), 1203 (m), 1181 (m), 1010 (s), 996 (s), 801 (m), 698 (m) cm$^{-1}$.

**$^1$H NMR (500 MHz, CDCl$_3$):** δ 7.42 – 7.36 (m, 2H), 7.30 (d, *J* = 8.7 Hz, 2H), 7.18 – 7.14 (m, 3H), 7.13 – 7.10 (m, 2H), 6.36 (ddd, *J* = 17.1, 10.3, 8.8 Hz, 1H), 5.41 – 5.33 (m, 2H), 4.35 (dd, *J* = 33.0, 9.0 Hz, 1H).

**$^{13}$C NMR (126 MHz, CDCl$_3$):** δ 166.2 (d, *J* = 28.0 Hz), 136.2, 134.3 (d, *J* = 21.5 Hz), 134.2 (d, *J* = 5.0 Hz), 131.7 (d, *J* = 1.8 Hz), 129.6 (d, *J* = 2.3 Hz), 128.5, 127.6, 126.8 (d, *J* = 9.9 Hz), 123.4, 120.2, 99.6 (d, *J* = 202.3 Hz), 57.6 (d, *J* = 19.0 Hz).

**$^{19}$F NMR (376 MHz, CDCl$_3$):** δ -151.41 – -151.56 (m, 2F), -156.63 (t, *J* = 21.7 Hz, 1F), -161.51 – -161.75 (m, 2F), -174.44 (d, *J* = 33.1 Hz, 1F).

**HRMS (EI):** m/z calcd for $[M]^+$ $C_{23}H_{13}BrF_6O_2$: 514.0003. Found: 514.0000.

**(2*R*,3*R*)-2-(4-Bromophenyl)-2-fluoro-3-phenylpent-4-enamide ((*R*,*R*)-4ea):**

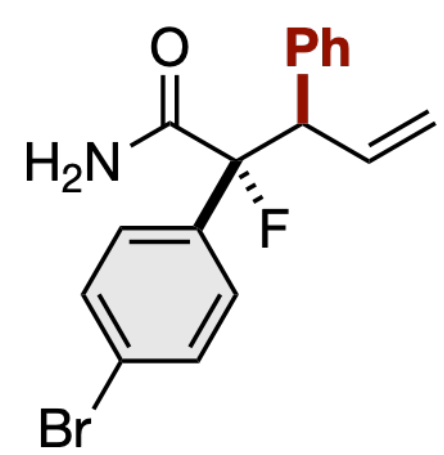


**IR (film):** 3425 (m), 3182 (br), 3087 (w), 3031 (w), 1667 (s), 1601 (w), 1487 (w), 1453 (w), 1393 (m), 617 (w) cm$^{-1}$.

**$^1$H NMR (500 MHz, CDCl$_3$):** δ 7.36 – 7.27 (m, 4H), 7.17 – 7.08 (m, 3H), 7.06 (dt, *J* = 7.6, 1.6 Hz, 2H), 6.41 (s, 1H), 6.33 – 6.16 (m, 1H), 5.55 (s, 1H), 5.29 – 5.17 (m, 2H), 4.31 (dd, *J* = 35.0, 8.5 Hz, 1H).

**$^{13}$C NMR (126 MHz, CDCl$_3$):** δ 172.1 (d, *J* = 22.7 Hz), 137.3, 136.0 (d, *J* = 22.4 Hz), 134.8 (d, *J* = 5.4 Hz), 131.2 (d, *J* = 2.0 Hz), 129.6 (d, *J* = 2.3 Hz), 128.3, 127.2, 126.7 (d, *J* = 10.4 Hz), 122.5, 119.5, 101.3 (d, *J* = 198.1 Hz), 57.1 (d, *J* = 19.3 Hz).

**$^{19}$F NMR (376 MHz, CDCl$_3$):** δ -171.78 (d, *J* = 35.0 Hz, 1F).

**HRMS (APCI):** m/z calcd for $[M+H]^+$ $C_{17}H_{16}ONBrF$: 348.0394. Found: 348.0395.

HPLC analysis using a chiral column (Chiralpak IB 3μ column 22 ˚C, 0.5 ml/min, 95:5 hexane:isopropanol, 210 nm, $t_{major}$ = 20.991 min, $t_{minor}$ = 25.336 min, t = 26.908 min, t = 33.291 min).

(±)-**10:** (*rac*)-**[Ir]** and (*rac*)-Bn-BTM

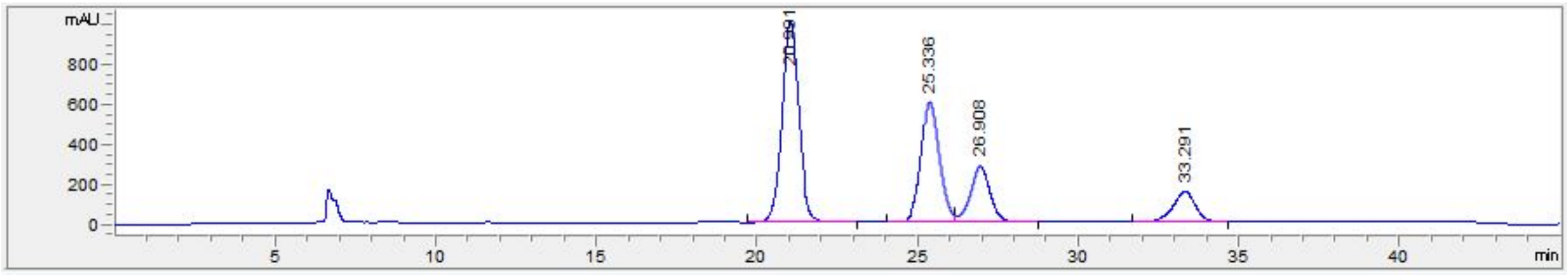


| # | Time | Area | Height | Width | Area% | Symmetry |
|---|---|---|---|---|---|---|
| 1 | 20.991 | 36832.1 | 1010.4 | 0.5553 | 46.309 | 0.886 |
| 2 | 25.336 | 23651.3 | 605.7 | 0.5876 | 29.737 | 0.81 |
| 3 | 26.908 | 11823.9 | 283.5 | 0.6286 | 14.866 | 0.971 |
| 4 | 33.291 | 7228 | 151.2 | 0.7098 | 9.088 | 1.139 |

(*R*,*R*)-**10:** ($R_a$,*R*,*R*)-**[Ir]** and (*S*)-Me-BTM

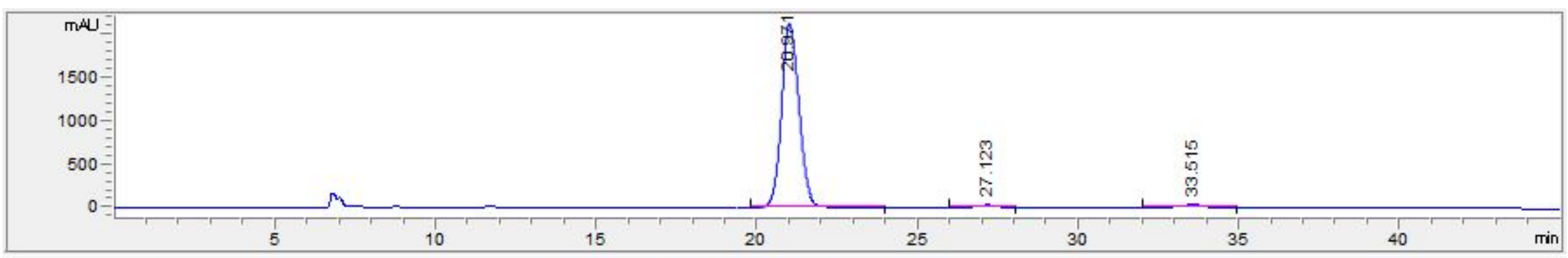


| # | Time | Area | Height | Width | Area% | Symmetry |
|---|---|---|---|---|---|---|
| 1 | 20.971 | 80459 | 2111.2 | 0.5886 | 96.411 | 0.763 |
| 2 | 27.123 | 1245.5 | 30.5 | 0.62 | 1.492 | 1.174 |
| 3 | 33.515 | 1749.3 | 36 | 0.719 | 2.096 | 1.227 |

**Perfluorophenyl (2*R*,3*S*)-2-(4-bromophenyl)-2-fluoro-3-phenylpent-4-enoate ((*R*,*S*)-11):**

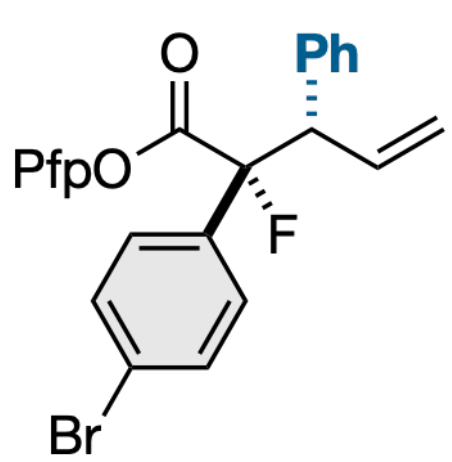


Prepared according to General Procedure B using pentafluorophenyl ester **1e** (39.9 mg, 0.1 mmol, 1 equiv.), *t*-butyl carbonate **2a** (35.1 mg, 0.15 mmol, 1.5 equiv.) and ($S_a$,*S*,*S*)-**[Ir]** catalyst. The *title compound* was obtained (38.9 mg, 0.0755 mmol, 75.5%) as a white solid following purification by column chromatography [$SiO_2$, 0.25% Ethyl Acetate in Petroleum Ether]. The enantiomeric ratio (>99:1 *er*) of the corresponding primary amide was determined by chiral HPLC in comparison with the racemate. Diastereomeric ratio (92:8) was determined by $^{19}$F-NMR analysis of the crude reaction mixture.

**IR (film):** 3065 (w), 3033 (w), 1780 (m), 1639 (w), 1591 (w), 1517 (s), 1489 (m), 1219 (m), 1196 (m), 1009 (s), 996 (s), 780 (m), 699 (s) cm$^{-1}$.

**$^1$H NMR (500 MHz, $CDCl_3$):** δ 7.61 (d, *J* = 8.7 Hz, 2H), 7.57 (d, *J* = 8.9 Hz, 2H), 7.49 – 7.43 (m, 2H), 7.42 – 7.31 (m, 3H), ***6.36 (ddd, J = 17.1, 10.2, 8.8 Hz)***, 5.96 (ddd, *J* = 17.1,

10.3, 8.1 Hz, 2H), ****5.44 – 5.31 (m)***, 5.09 (d, *J* = 10.2 Hz, 1H), 4.93 (d, *J* = 17.1 Hz, 2H), 4.34 (dd, *J* = 33.5, 8.0 Hz, 1H).

**$^{13}$C NMR (126 MHz, $CDCl_3$):** δ 165.7 (d, *J* = 28.5 Hz), 137.2, 134.9 (d, *J* = 23.1 Hz), 133.5 (d, *J* = 5.0 Hz), 132.1 (d, *J* = 2.1 Hz), 129.5 (d, *J* = 2.9 Hz), 128.9, 128.1, 127.1 (d, *J* = 10.4 Hz), 123.8, 120.3, 99.0 (d, *J* = 202.3 Hz), 57.2 (d, *J* = 18.8 Hz).

**$^{19}$F NMR (376 MHz, $CDCl_3$):** δ -151.65 – -151.86 (m, 2F), -156.90 (t, *J* = 21.6 Hz, 1F), -161.74 – -162.04 (m, 2F), -173.54 (d, *J* = 33.6 Hz, 1F).

**HRMS (EI):** m/z calcd for $[M]^+$ $C_{23}H_{13}BrF_6O_2$: 514.0003. Found: 514.0002.

**(2*R*,3*S*)-2-(4-Bromophenyl)-2-fluoro-3-phenylpent-4-enamide ((*R*,*S*)-11-$NH_2$):**

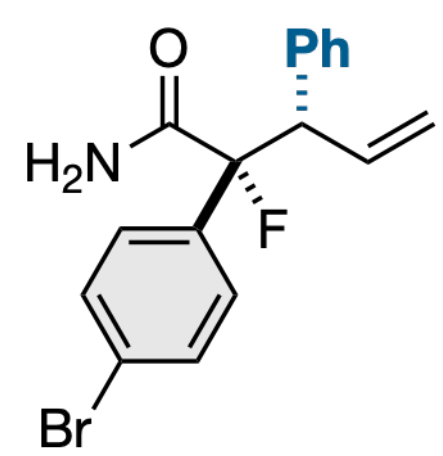


**IR (film):** 3433 (m), 3176 (br), 1667 (s), 1603 (w), 1487 (w), 1452 (w), 1394 (w), 600 (m) $cm^{-1}$.

**$^1$H NMR (500 MHz, $CDCl_3$):** δ 7.58 – 7.50 (m, 4H), 7.40 (dt, *J* = 8.1, 1.5 Hz, 2H), 7.36 – 7.30 (m, 2H), 7.30 – 7.27 (m, 1H)*,* ****6.41 (s)*, *6.34 – 6.20 (m)***, 5.93 (ddd, *J* = 17.1, 10.3, 8.5 Hz, 1H), ****5.50 (s)***, ****5.29 – 5.22 (m)***, 5.09 (s, 1H), 5.01 (dt, *J* = 10.4, 1.1 Hz, 1H), 4.87 (d, *J* = 16.9 Hz, 1H), 4.30 (dd, *J* = 36.0, 8.5 Hz. 1H).

**$^{13}$C NMR (126 MHz, $CDCl_3$):** δ 171.6 (d, *J* = 23.0 Hz), 138.1, 136.4 (d, *J* = 22.5 Hz), 134.0 (d, *J* = 4.6 Hz), 131.5 (d, *J* = 2.2 Hz), 129.9 (d, *J* = 2.4 Hz), 128.5, 127.5, 127.0 (d, *J* = 11.0 Hz), 122.8, 119.7, 100.8 (d, *J* = 199.4 Hz), 56.7 (d, *J* = 18.9 Hz).

**$^{19}$F NMR (376 MHz, $CDCl_3$):** δ -173.25 (d, *J* = 35.9 Hz, 1F).

**HRMS (APCI):** m/z calcd for $[M+H]^+$ $C_{17}H_{16}ONBrF$: 348.0394. Found: 348.0394.
HPLC analysis using a chiral column (Chiralpak IB 3μ column 22 ˚C, 0.5 ml/min, 95:5 hexane:isopropanol, 210 nm, $t_{major}$ = 33.291 min, $t_{minor}$ = 26.908 min, t = 20.991 min, t = 25.336 min).

(±)-**11:** (*rac*)-**[Ir]** and (*rac*)-Bn-BTM

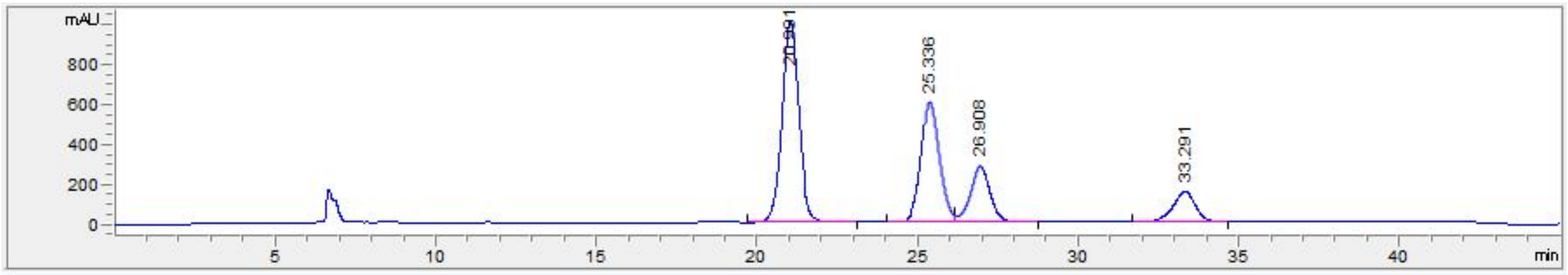


| # | Time | Area | Height | Width | Area% | Symmetry |
|---|---|---|---|---|---|---|
| 1 | 20.991 | 36832.1 | 1010.4 | 0.5553 | 46.309 | 0.886 |
| 2 | 25.336 | 23651.3 | 605.7 | 0.5876 | 29.737 | 0.81 |
| 3 | 26.908 | 11823.9 | 283.5 | 0.6286 | 14.866 | 0.971 |
| 4 | 33.291 | 7228 | 151.2 | 0.7098 | 9.088 | 1.139 |

(*R*,*S*)-**11:** ($S_a$,*S*,*S*)-**[Ir]** and (*S*)-Me-BTM

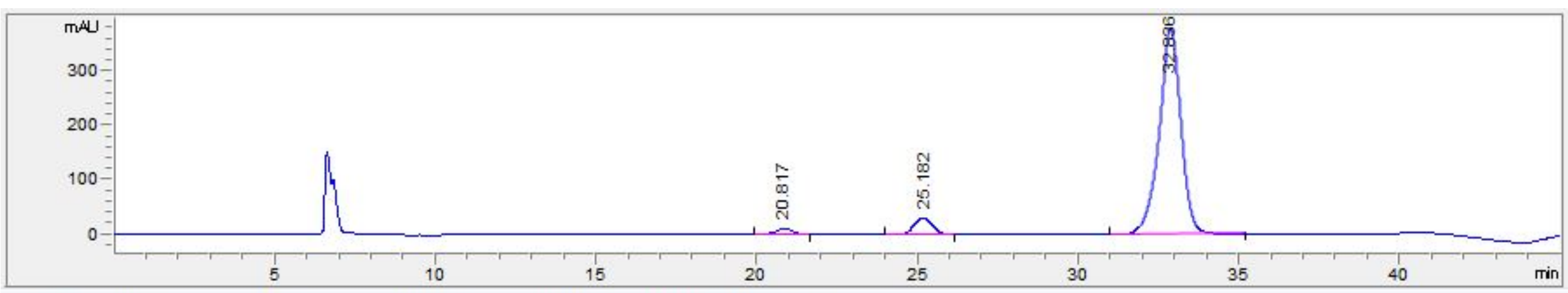


| # | Time | Area | Height | Width | Area% | Symmetry |
|---|---|---|---|---|---|---|
| 1 | 20.817 | 378.9 | 10.6 | 0.5201 | 1.955 | 1.185 |
| 2 | 25.182 | 1197.3 | 30.9 | 0.5879 | 6.177 | 1.101 |
| 3 | 32.836 | 17808.2 | 378.9 | 0.6963 | 91.869 | 1.088 |

**Perfluorophenyl (2*R*,3*R*)-2-fluoro-2-(4-iodophenyl)-3-phenylpent-4-enoate ((*R*,*R*)-12):**

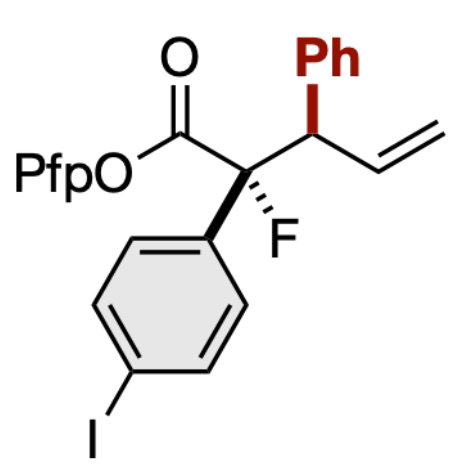


Prepared according to General Procedure B using pentafluorophenyl ester **1f** (44.6 mg, 0.1 mmol, 1 equiv.), *t*-butyl carbonate **2a** (35.1 mg, 0.15 mmol, 1.5 equiv.) and ($R_a$,*R*,*R*)-**[Ir]** (4.2 mg, 4 mol%), (*S*)-Me-BTM (3.8 mg, 20 mol%) and DIPEA (22.6 µL, 0.13 mmol, 1.3 equiv.) in 1,4-dioxane (0.3 ml, 0.3 M). The *title compound* was obtained (41.5 mg, 0.074 mmol, 74%) as a colorless oil following purification by column chromatography [$SiO_2$, 0.25% Ethyl Acetate in Petroleum Ether]. The enantiomeric ratio (>99:1 *er*) of the corresponding primary amide was determined by chiral HPLC in comparison with the racemate. Diastereomeric ratio (98:2) was determined by $^{19}$F-NMR analysis of the crude reaction mixture.

**IR (film):** 3064 (w), 3031 (w), 1782 (m), 1639 (w), 1586 (w), 1517 (s), 1487 (m), 1202 (m), 1180 (m), 1008 (s), 995 (s), 799 (m), 698 (s) $cm^{-1}$.

**$^{1}$H NMR (500 MHz, CDCl$_3$):** δ 7.60 (d, *J* = 8.4 Hz, 2H), 7.19 – 7.08 (m, 7H), 6.36 (ddd, *J* = 17.1, 10.3, 8.8 Hz, 1H), 5.44 – 5.29 (m, 2H), 4.34 (dd, *J* = 33.0, 8.5 Hz, 1H).

**$^{13}$C NMR (126 MHz, CDCl$_3$):** δ 166.2 (d, *J* = 27.9 Hz), 137.6 (d, *J* = 1.9 Hz), 136.2, 135.0 (d, *J* = 23.0 Hz), 134.2 (d, *J* = 5.1 Hz), 129.6 (d, *J* = 2.3 Hz), 128.5, 127.6, 126.9 (d, *J* = 9.8 Hz), 120.2, 99.6 (d, *J* = 202.4 Hz), 95.3, 57.5 (d, *J* = 19.0 Hz).

**$^{19}$F NMR (376 MHz, CDCl$_3$):** δ -151.34 – -151.53 (m, 2F), -156.63 (t, *J* = 21.6 Hz, 1F), -161.47 – -161.76 (m, 2F), -174.68 (d, *J* = 33.1 Hz, 1F).

**HRMS (EI):** m/z calcd for $[M]^+$ $C_{23}H_{13}F_6IO_2$: 561.9864. Found: 561.9867.

**(2*R*,3*R*)-2-Fluoro-2-(4-iodophenyl)-3-phenylpent-4-enamide ((*R,R*)-12-NH$_2$):**

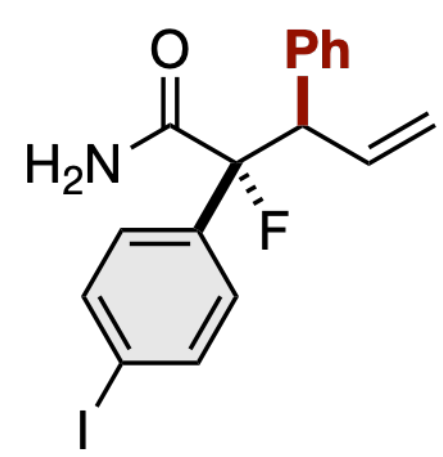


**IR (film):** 3425 (m), 3182 (br), 3086 (w) , 3031 (w), 1668 (s), 1600 (w), 1485 (w), 1452 (w), 1390 (m), 697 (m) cm$^{-1}$.

**$^{1}$H NMR (500 MHz, CDCl$_3$):** δ 7.51 (d, *J* = 8.5 Hz, 2H), 7.22 – 7.09 (m, 5H), 7.06 (dt, *J* = 7.7, 1.6 Hz, 2H), 6.41 (s, 1H), 6.33 – 6.19 (m, 1H), 5.50 (s, 1H), 5.32 – 5.19 (m, 2H), 4.31 (dd, *J* = 34.5, 8.0 Hz, 1H).

**$^{13}$C NMR (126 MHz, CDCl$_3$):** δ 172.0 (d, *J* = 22.9 Hz), 137.3, 137.1 (d, *J* = 2.0 Hz), 136.7 (d, *J* = 22.4 Hz), 134.8 (d, *J* = 5.4 Hz), 129.6 (d, *J* = 2.2 Hz), 128.3, 127.2, 126.8 (d, *J* = 10.2 Hz), 119.5, 101.3 (d, *J* = 198.2 Hz), 94.4, 57.0 (d, *J* = 19.3 Hz).

**$^{19}$F NMR (376 MHz, CDCl$_3$):** δ -171.95 (d, *J* = 34.7 Hz, 1F).

**HRMS (ESI):** m/z calcd for $[M+Na]^+$ $C_{17}H_{15}ONFINa$: 418.0075. Found: 418.0074.
HPLC analysis using a chiral column (Cellulose 1 3μ column 22 ˚C, 1.0 ml/min, 95:5 hexane:isopropanol, 210 nm, $t_{major}$ = 17.861 min, $t_{minor}$ = 23.193 min, t = 27.647 min, t = 38.225 min).

(±)-**12:** (*rac*)-**[Ir]** and (*rac*)-Bn-BTM

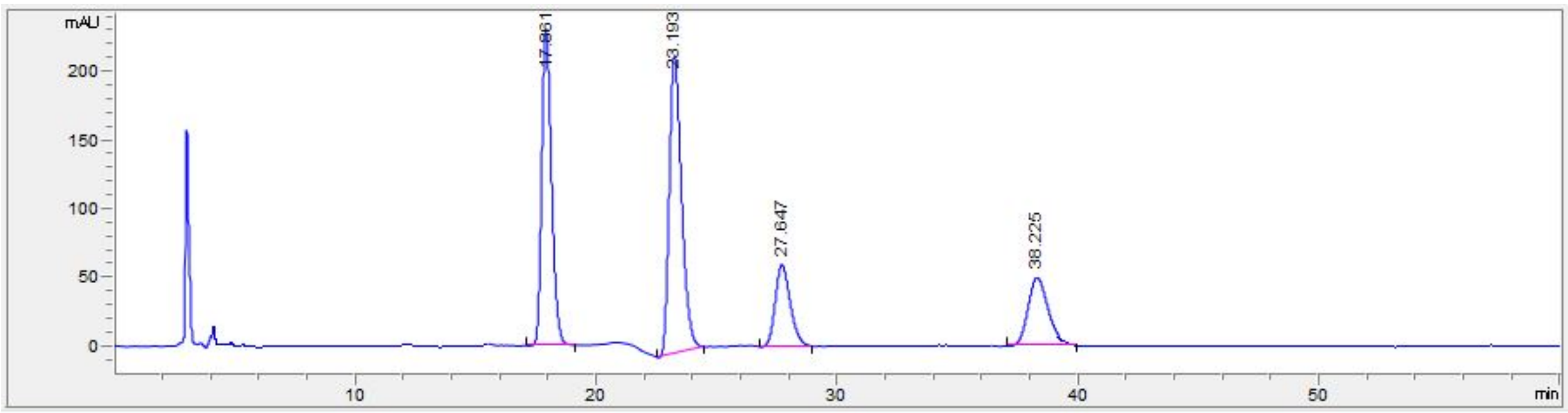


| # | Time | Area | Height | Width | Area% | Symmetry |
|---|---|---|---|---|---|---|
| 1 | 17.861 | 6499.2 | 229.9 | 0.4378 | 32.980 | 0.74 |
| 2 | 23.193 | 7694.8 | 216.6 | 0.5464 | 39.047 | 0.699 |
| 3 | 27.647 | 2589.2 | 59.6 | 0.6405 | 13.139 | 0.768 |
| 4 | 38.225 | 2923.2 | 49.6 | 0.858 | 14.834 | 0.746 |

(*R*,*R*)-**12:** ($R_a$,*R*,*R*)-**[Ir]** and (*S*)-Me-BTM

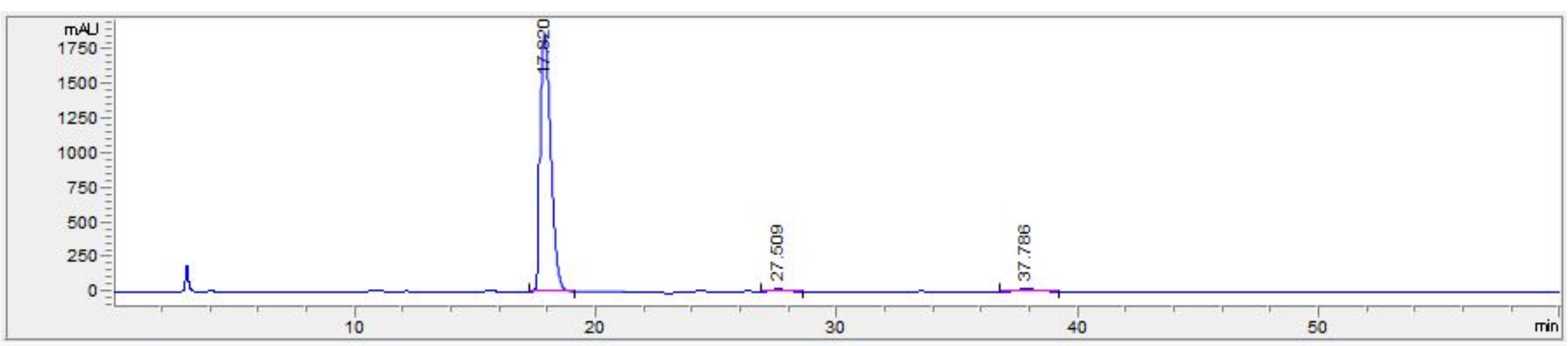


| # | Time | Area | Height | Width | Area% | Symmetry |
|---|---|---|---|---|---|---|
| 1 | 17.82 | 56404.6 | 1868.5 | 0.4782 | 96.507 | 0.612 |
| 2 | 27.509 | 789.3 | 19.1 | 0.6096 | 1.350 | 0.688 |
| 3 | 37.786 | 1252.1 | 19.7 | 0.8733 | 2.142 | 0.717 |

**Perfluorophenyl (2*R*,3*S*)-2-fluoro-2-(4-iodophenyl)-3-phenylpent-4-enoate ((*R*,*S*)-12)**: Prepared according to General Procedure B using pentafluorophenyl ester **1f** (44.6 mg, 0.1 mmol, 1 equiv.), *t*-butyl carbonate **2a** (35.1 mg, 0.15 mmol, 1.5 equiv.) and ($S_a$,*S*,*S*)-**[Ir]** (4.2 mg, 4 mol%), (*S*)-Me-BTM (3.8 mg, 20 mol%) and DIPEA (22.6 µL, 0.13 mmol, 1.3 equiv.) in 1,4-dioxane (0.3 ml, 0.3 M). The *title compound* was obtained (43.5 mg, 0.077 mmol, 77%) as a white solid following purification by column chromatography [$SiO_2$, 0.25% Ethyl Acetate in Petroleum Ether]. The enantiomeric ratio (>99:1 *er*) of the corresponding primary amide was determined by

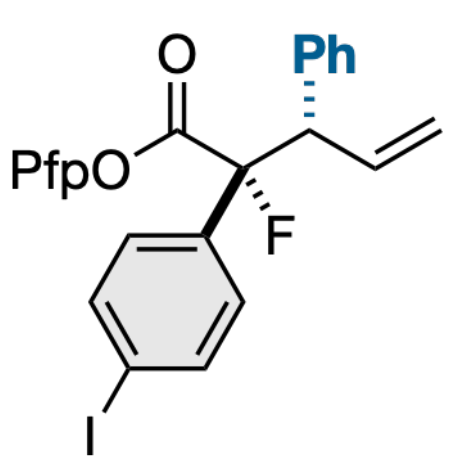

chiral HPLC in comparison with the racemate. Diastereomeric ratio (92:8) was determined by $^{19}$F-NMR analysis of the crude reaction mixture.

**IR (film):** 3065 (w), 3032 (w), 1799 (w), 1780 (m), 1639 (w), 1586 (w), 1517 (s), 1486 (m), 1219 (m), 1188 (m), 1008 (s), 996 (s), 798 (m), 700 (m) cm$^{-1}$.

**$^{1}$H NMR (500 MHz, CDCl$_3$):** δ 7.74 (d, *J* = 8.4 Hz, 2H), 7.41 – 7.34 (m, 4H), 7.33 – 7.22 (m, 3H), ***6.39 – 6.20 (m)***, 5.88 (ddd, *J* = 17.1, 10.3, 8.1 Hz, 1H), ***5.41 – 5.23 (m)***, 5.01 (d, *J* = 10.2 Hz, 1H), 4.85 (d, *J* = 17.1 Hz, 1H), 4.26 (dd, *J* = 33.5, 8 Hz, H).

**$^{13}$C NMR (126 MHz, CDCl$_3$):** δ 165.7 (d, *J* = 28.4 Hz), 138.0 (d, *J* = 2.0 Hz), 137.2, 135.6 (d, *J* = 23.0 Hz), 133.5 (d, *J* = 5.0 Hz), 129.5 (d, *J* = 3.0 Hz), 128.9, 128.1, 127.2 (d, *J* = 10.2 Hz), 120.3, 99.0 (d, *J* = 202.5 Hz), 95.7, 57.1 (d, *J* = 18.8 Hz).

**$^{19}$F NMR (376 MHz, CDCl$_3$):** δ -151.62 – -151.81 (m, 2F), -156.90 (t, *J* = 21.8 Hz, 1F), -161.75 – -161.96 (m, 2F), -173.83 (d, *J* = 33.5 Hz, 1F).

**HRMS (EI):** m/z calcd for $[M]^+$ $C_{23}H_{13}F_6IO_2$: 561.9864. Found: 561.9866.

**(2*R*,3*S*)-2-Fluoro-2-(4-iodophenyl)-3-phenylpent-4-enamide ((*R*,*S*)-12-NH$_2$):**

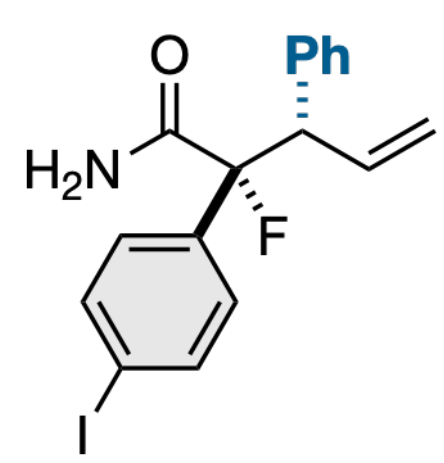


**IR (film):** 3436 (m), 3170 (br), 1667 (s), 1608 (w), 1487 (w), 1452 (w), 1392 (w), 610 (m) cm$^{-1}$.

**$^{1}$H NMR (500 MHz, CDCl$_3$):** δ 7.77 – 7.69 (m, 2H), 7.45 – 7.37 (m, 4H), 7.37 – 7.30 (m, 2H), 7.29 – 7.24 (m, 1H), ***6.40 (s)***, ***6.30 – 6.19 (m)***, 6.01 (s, 1H), 5.93 (ddd, *J* = 17.1, 10.3, 8.5 Hz, 1H), ***5.45 (s)***, ***5.32 – 5.20 (m)***, 5.06 (d, *J* = 5.7 Hz, 1H), 5.01 (dt, *J* = 10.5, 1.1 Hz, 1H), 4.87 (dd, *J* = 17.1, 1.4 Hz, 1H), 4.30 (dd, *J* = 36.0, 8.5 Hz, 1H).

**$^{13}$C NMR (126 MHz, CDCl$_3$):** δ 171.6 (d, *J* = 22.9 Hz), 138.1, 137.5 (d, *J* = 2.1 Hz), 137.1 (d, *J* = 22.4 Hz), 134.0 (d, *J* = 4.5 Hz), 129.9 (d, *J* = 2.3 Hz), 128.5, 127.5, 127.2 (d, *J* = 11.0 Hz), 119.8, 100.8 (d, *J* = 199.6 Hz), 94.6, 56.6 (d, *J* = 18.8 Hz).

**$^{19}$F NMR (376 MHz, CDCl$_3$):** δ -173.48 (d, $J$ = 36.0 Hz, 1F).

**HRMS (ESI):** m/z calcd for $[M+Na]^+$ $C_{17}H_{15}ONFINa$: 418.0075. Found: 418.0075.

HPLC analysis using a chiral column (Cellulose 1 3μ column 22 ˚C, 1.0 ml/min, 95:5 hexane:isopropanol, 210 nm, $t_{major}$ = 38.225 min, $t_{minor}$ = 27.647 min, t = 17.861 min, t = 23.193 min).

(±)-**12:** (*rac*)-**[Ir]** and (*rac*)-Bn-BTM

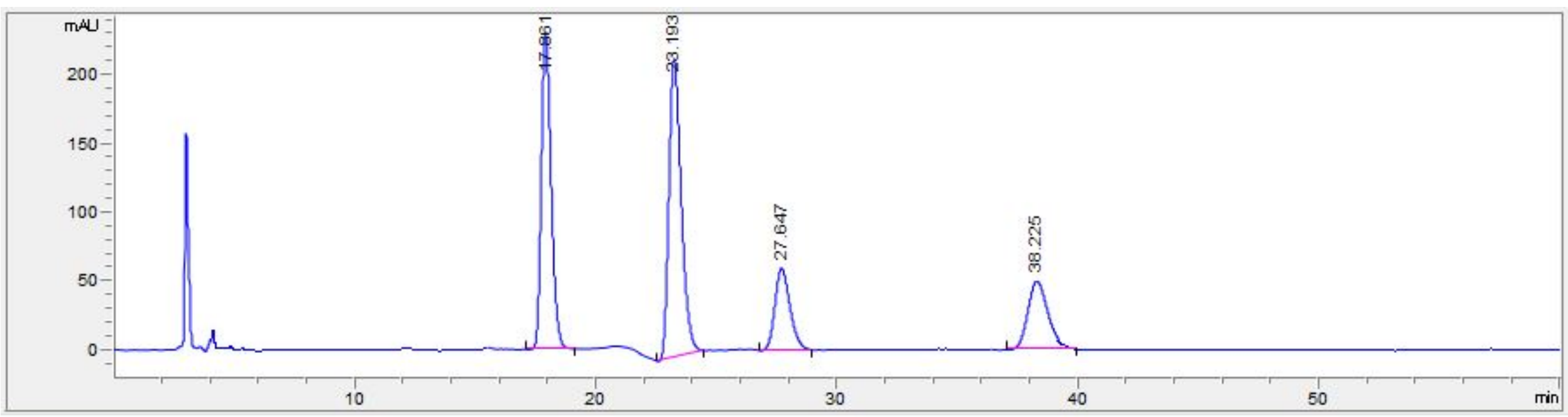


| # | Time | Area | Height | Width | Area% | Symmetry |
|---|---|---|---|---|---|---|
| 1 | 17.861 | 6499.2 | 229.9 | 0.4378 | 32.980 | 0.74 |
| 2 | 23.193 | 7694.8 | 216.6 | 0.5464 | 39.047 | 0.699 |
| 3 | 27.647 | 2589.2 | 59.6 | 0.6405 | 13.139 | 0.768 |
| 4 | 38.225 | 2923.2 | 49.6 | 0.858 | 14.834 | 0.746 |

(*R*,*S*)-**12:** ($S_a$,*S*,*S*)-**[Ir]** and (*S*)-Me-BTM

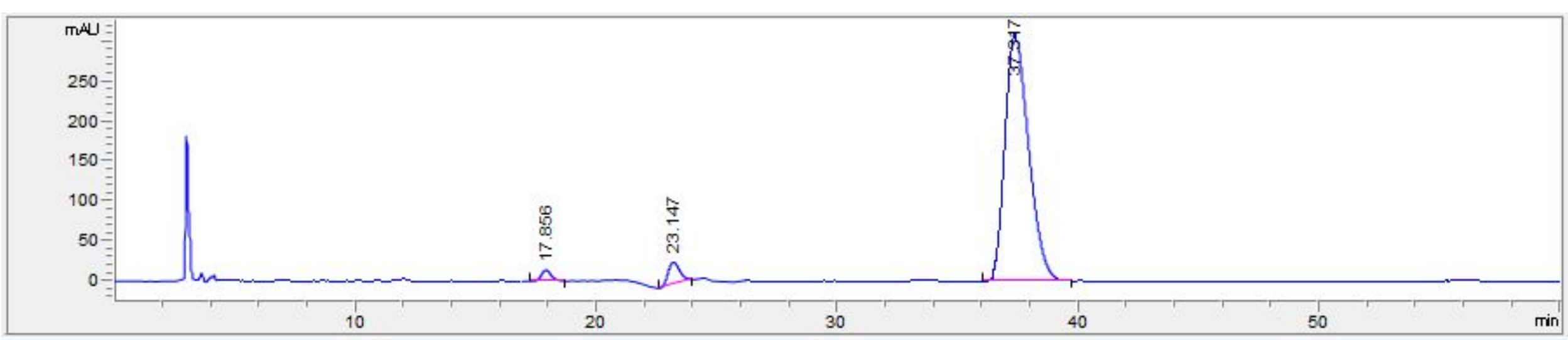


| # | Time | Area | Height | Width | Area% | Symmetry |
|---|---|---|---|---|---|---|
| 1 | 17.856 | 407.2 | 14 | 0.4445 | 1.838 | 0.831 |
| 2 | 23.147 | 964.8 | 28.2 | 0.5258 | 4.354 | 0.808 |
| 3 | 37.317 | 20785.9 | 310.6 | 1.0207 | 93.808 | 0.662 |

**Perfluorophenyl (2*R*,3*R*)-2-([1,1'-biphenyl]-4-yl)-2-fluoro-3-phenylpent-4-enoate**

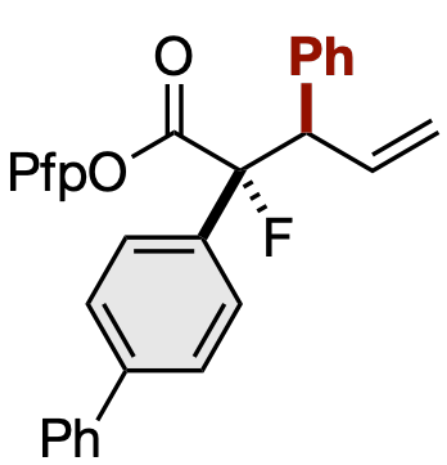


**((*R*,*R*)-8):** Prepared according to General Procedure B using pentafluorophenyl ester **1g** (39.6 mg, 0.1 mmol, 1 equiv.), *t*-butyl carbonate **2a** (35.1 mg, 0.15 mmol, 1.5 equiv.) and ($R_a$,*R*,*R*)-**[Ir]** (4.2 mg, 4 mol%), (*S*)-Me-BTM (3.8 mg, 20 mol%) and DIPEA (22.6 μL, 0.13 mmol, 1.3 equiv.) in 1,4-dioxane (0.3 ml, 0.3 M). The *title compound*

was obtained (41.5 mg, 0.0081 mmol, 81%) as a white solid following purification by column chromatography [$SiO_2$, 0.25% Ethyl Acetate in Petroleum Ether]. The enantiomeric ratio (>99:1 *er*) of the corresponding primary amide was determined by chiral HPLC in comparison with the racemate. Diastereomeric ratio (97:3) was determined by $^{19}$F-NMR analysis of the crude reaction mixture.

**IR (film):** 3062 (w), 3032 (w), 1782 (m), 1639 (w), 1601 (w), 1518 (s), 1488 (m), 1204 (m), 1181 (m), 1007 (s), 995 (s), 762 (m), 698 (s) cm$^{-1}$.

**$^1$H NMR (500 MHz, $CDCl_3$):** δ 7.57 – 7.51 (m, 2H), 7.52 – 7.47 (m, 4H), 7.45 – 7.38 (m, 2H), 7.37 – 7.30 (m, 1H), 7.21 – 7.10 (m, 5H), 6.41 (ddd, *J* = 17.1, 10.3, 8.8 Hz, 1H), 5.45 – 5.34 (m, 2H), 4.44 (dd, *J* = 33.0, 9.0 Hz, 1H).

**$^{13}$C NMR (126 MHz, $CDCl_3$):** δ 166.5 (d, *J* = 28.2 Hz), 141.6, 140.1, 136.6, 134.5 (d, *J* = 5.0 Hz), 134.0 (d, *J* = 22.5 Hz), 129.7 (d, *J* = 2.3 Hz), 128.9, 128.4, 127.8, 127.4, 127.2, 127.1 (d, *J* = 1.8 Hz), 125.5 (d, *J* = 9.6 Hz), 120.0, 99.9 (d, *J* = 201.6 Hz), 57.5 (d, *J* = 19.1 Hz).

**$^{19}$F NMR (376 MHz, $CDCl_3$):** δ -151.24 – -151.37 (m, 2F), -156.91 (t, *J* = 21.7 Hz, 1F), -161.81 (dd, *J* = 21.8, 17.6 Hz, 2F), -174.04 (d, *J* = 33.5 Hz, 1F).

**HRMS (EI):** m/z calcd for $[M]^+$ $C_{29}H_{18}F_6O_2$: 512.1211. Found: 512.1213.

**(2*R*,3*R*)-2-([1,1'-Biphenyl]-4-yl)-2-fluoro-3-phenylpent-4-enamide ((*R*,*R*)-8-$NH_2$):**

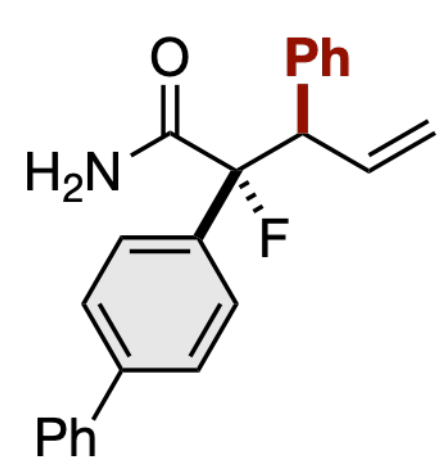


**IR (film):** 3429 (m), 3177 (br), 3083 (w), 3030 (w), 1669 (s), 1601 (w), 1488 (m), 1452 (w), 1392 (m) cm$^{-1}$.

**$^1$H NMR (500 MHz, $CDCl_3$):** δ 7.54 – 7.45 (m, 4H), 7.45 – 7.36 (m, 4H), 7.34 – 7.29 (m, 1H), 7.17 – 7.04 (m, 5H), 6.45 (s, 1H), 6.31 (ddd, *J* = 17.0, 10.3, 8.4 Hz, 1H), 5.47 (d, *J* = 6.1 Hz, 1H), 5.34 – 5.19 (m, 2H), 4.42 (dd, *J* = 34.5, 8.5 Hz, 1H).

**$^{13}$C NMR (126 MHz, $CDCl_3$):** δ 172.5 (d, *J* = 22.7 Hz), 140.8, 140.5, 137.7, 135.9 (d, *J* = 22.2 Hz), 135.2 (d, *J* = 5.4 Hz), 129.7 (d, *J* = 2.3 Hz), 128.9, 128.2, 127.6, 127.1, 127.0, 126.7 (d, *J* = 1.9 Hz), 125.4 (d, *J* = 10.2 Hz), 119.3, 101.7 (d, *J* = 197.5 Hz), 57.1 (d, *J* = 19.4 Hz).

**$^{19}$F NMR (376 MHz, $CDCl_3$):** δ -171.04 (d, *J* = 34.9 Hz, 1F).

**HRMS (ESI):** m/z calcd for $[M+Na]^+$ $C_{23}H_{20}ONFNa$: 368.1421. Found: 368.1423.

HPLC analysis using a chiral column (Chiralpak IB 3μ column 22 ˚C, 1.0 ml/min, 95:5 hexane:isopropanol, 210 nm, $t_{major}$ = 25.892 min, $t_{minor}$ = 16.747 min, t = 14.203 min, t = 29.628 min).

(±)-**8:** (*rac*)-**[Ir]** and (*rac*)-Bn-BTM

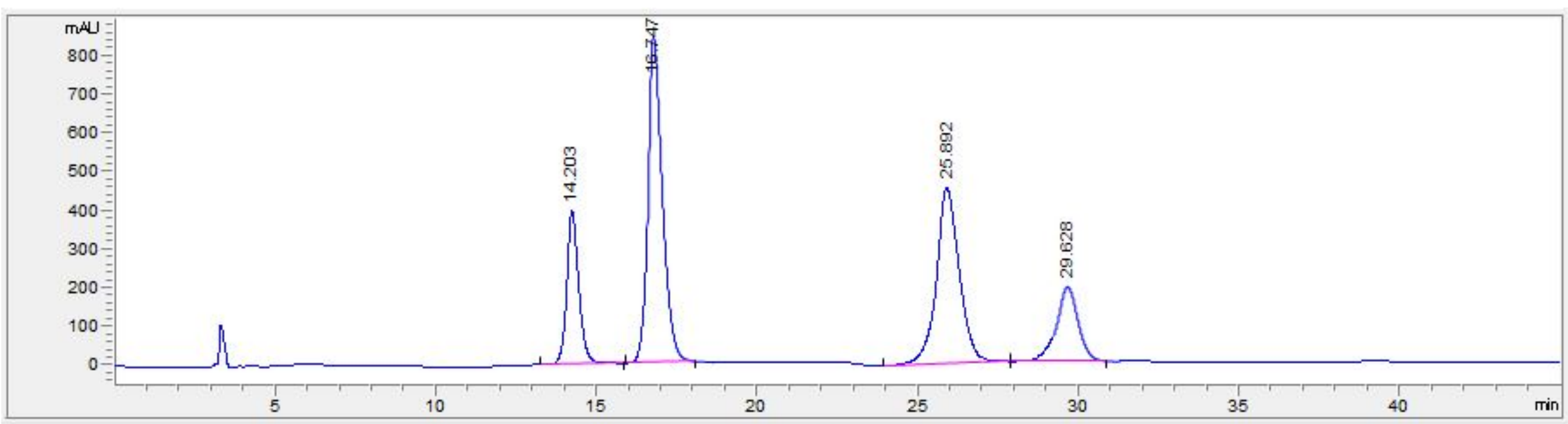


| # | Time | Area | Height | Width | Area% | Symmetry |
|---|---|---|---|---|---|---|
| 1 | 14.203 | 10081.1 | 395.5 | 0.3794 | 14.922 | 0.728 |
| 2 | 16.747 | 25855.8 | 845.8 | 0.4507 | 38.272 | 0.593 |
| 3 | 25.892 | 22530 | 456.2 | 0.7382 | 33.349 | 0.831 |
| 4 | 29.628 | 9091.9 | 193.3 | 0.7008 | 13.458 | 1.042 |

(*R*,*R*)-**8:** (*R<sub></sub>*$_a$,*R*,*R*)-**[Ir]** and (*S*)-Me-BTM

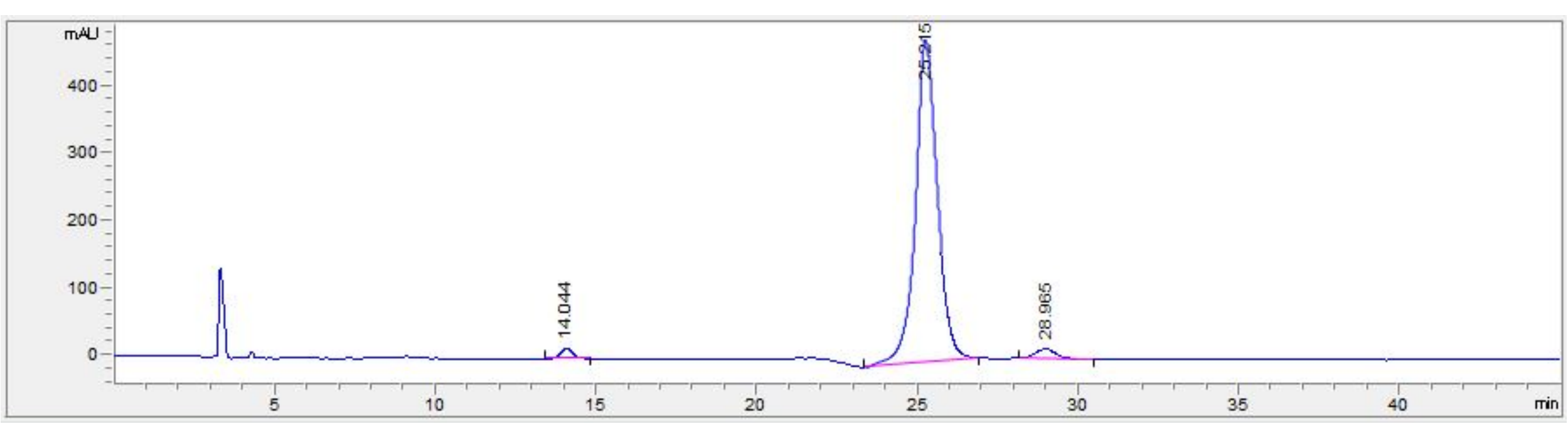


| # | Time | Area | Height | Width | Area% | Symmetry |
|---|---|---|---|---|---|---|
| 1 | 14.044 | 412.7 | 15.9 | 0.392 | 1.710 | 0.931 |
| 2 | 25.215 | 23079.7 | 477.5 | 0.7038 | 95.620 | 0.843 |
| 3 | 28.965 | 644.6 | 15.2 | 0.6225 | 2.670 | 0.892 |

**Perfluorophenyl (2*R*,3*S*)-2-([1,1'-biphenyl]-4-yl)-2-fluoro-3-phenylpent-4-enoate**

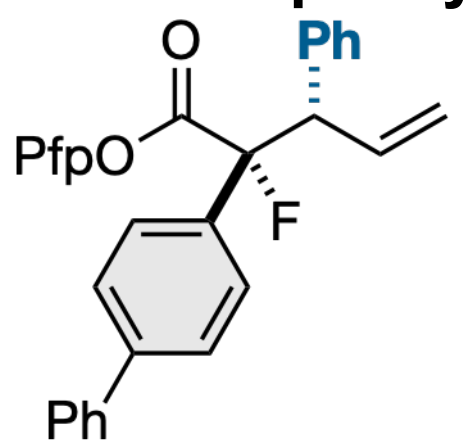


**((*R*,*S*)-8):** Prepared according to General Procedure B using pentafluorophenyl ester **1g** (39.6 mg, 0.1 mmol, 1 equiv.), *t*-butyl carbonate **2a** (35.1 mg, 0.15 mmol, 1.5 equiv.) and ($S_a$,*S*,*S*)-**[Ir]** (4.2 mg, 4 mol%), (*S*)-Me-BTM (3.8 mg, 20 mol%) and DIPEA (22.6 µL, 0.13 mmol, 1.3 equiv.) in 1,4-dioxane (0.3 ml, 0.3 M). The *title compound* was obtained (39.5 mg, 0.077 mmol, 77%) as a white solid following purification by column chromatography [$SiO_2$, 0.25% Ethyl Acetate in Petroleum Ether]. The enantiomeric ratio (>99:1 *er*) of the corresponding primary amide was determined by chiral HPLC in comparison with the racemate. Diastereomeric ratio (94:6) was determined by $^{19}$F-NMR analysis of the crude reaction mixture.

**IR (film):** 3063 (w), 3033 (w), 1799 (w), 1778 (m), 1639 (w), 1601 (w), 1517 (s), 1488 (m), 1190 (m), 1006 (s), 996 (s), 746 (s), 698 (s) cm$^{-1}$.

**$^1$H NMR (500 MHz, $CDCl_3$):** δ 7.69 (d, *J* = 8.5 Hz, 2H), 7.62 (d, *J* = 8.3 Hz, 2H), 7.56 (dd, *J* = 8.3, 1.3 Hz, 2H), 7.45 – 7.35 (m, 4H), 7.33 – 7.29 (m, 3H), 7.28 – 7.24 (m, 1H), 5.95 (ddd, *J* = 17.2, 10.3, 7.9 Hz, 1H), ***5.39 – 5.24 (m)***, 5.03 (d, *J* = 10.4 Hz, 1H), 4.88 (d, *J* = 17.1 Hz, 1H), 4.38 (dd, *J* = 34.0, 8.0 Hz, 1H).

**$^{13}$C NMR (126 MHz, $CDCl_3$):** δ 166.0 (d, *J* = 28.6 Hz), 142.2, 140.2, 137.5, 134.6 (d, *J* = 22.9 Hz), 133.9 (d, *J* = 4.9 Hz), 129.6 (d, *J* = 3.1 Hz), 129.0, 128.9, 128.0, 127.9, 127.5 (d, *J* = 2.1 Hz), 127.3, 125.8 (d, *J* = 10.1 Hz), 120.0, 99.3 (d, *J* = 201.5 Hz), 57.0 (d, *J* = 18.8 Hz).

**$^{19}$F NMR (376 MHz, $CDCl_3$):** δ -151.49 – -151.65 (m, 2F), -157.18 (t, *J* = 21.7 Hz, 1F), -161.93 – -162.13 (m, 2F), -173.13 (d, *J* = 33.7 Hz, 1F).

**HRMS (EI):** m/z calcd for $[M]^+$ $C_{29}H_{18}F_6O_2$: 512.1211. Found: 512.1209.

**(2*R*,3*S*)-2-([1,1'-Biphenyl]-4-yl)-2-fluoro-3-phenylpent-4-enamide ((*R*,*S*)-8-NH$_2$):**

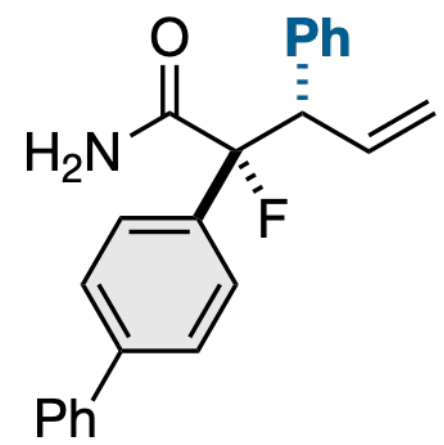

**IR (film):** 3433 (m), 3167 (br), 1668 (s), 1602 (w), 1488 (w), 1451 (w), 1405 (w) cm$^{-1}$.

**$^1$H NMR (500 MHz, DMSO):** δ 7.74 – 7.66 (m, 6H), 7.53 (s, 1H), 7.48 (t, *J* = 7.7 Hz, 2H), 7.43 – 7.35 (m, 3H), 7.32 (t, *J* = 7.5 Hz, 2H), 7.28 – 7.22 (m, 1H), 7.19 – 7.14 (m, 1H), 5.93 (ddd, *J* = 17.1, 10.3, 8.7 Hz, 1H), 4.95 (dd, *J* = 10.3, 1.9 Hz, 1H), 4.90 (d, *J* = 17.4 Hz, 1H), 4.50 (dd, *J* = 35, 8.5 Hz, 1H).

**$^{13}$C NMR (126 MHz, DMSO):** δ 170.8 (d, *J* = 23.5 Hz), 139.6, 139.5, 139.0, 137.4 (d, *J* = 22.8 Hz), 135.6 (d, *J* = 4.6 Hz), 129.6 (d, *J* = 2.4 Hz), 129.0, 128.0, 127.6, 126.8, 126.7, 126.2, 125.6 (d, *J* = 10.4 Hz), 118.7, 99.9 (d, *J* = 199.9 Hz), 55.0 (d, *J* = 18.6 Hz).

**$^{19}$F NMR (376 MHz, DMSO):** δ -171.98 (d, *J* = 35.0 Hz, 1F).

**HRMS (ESI):** m/z calcd for [M+Na]$^+$ $C_{23}H_{20}ONFNa$: 368.1421. Found: 368.1422.
HPLC analysis using a chiral column (Chiralpak IB 3μ column 22 ˚C, 1.0 ml/min, 95:5 hexane:isopropanol, 210 nm, $t_{major}$ = 29.628 min, $t_{minor}$ = 14.203 min, t = 16.747 min, t = 25.892 min).

(±)-**8:** (*rac*)-**[Ir]** and (*rac*)-Bn-BTM

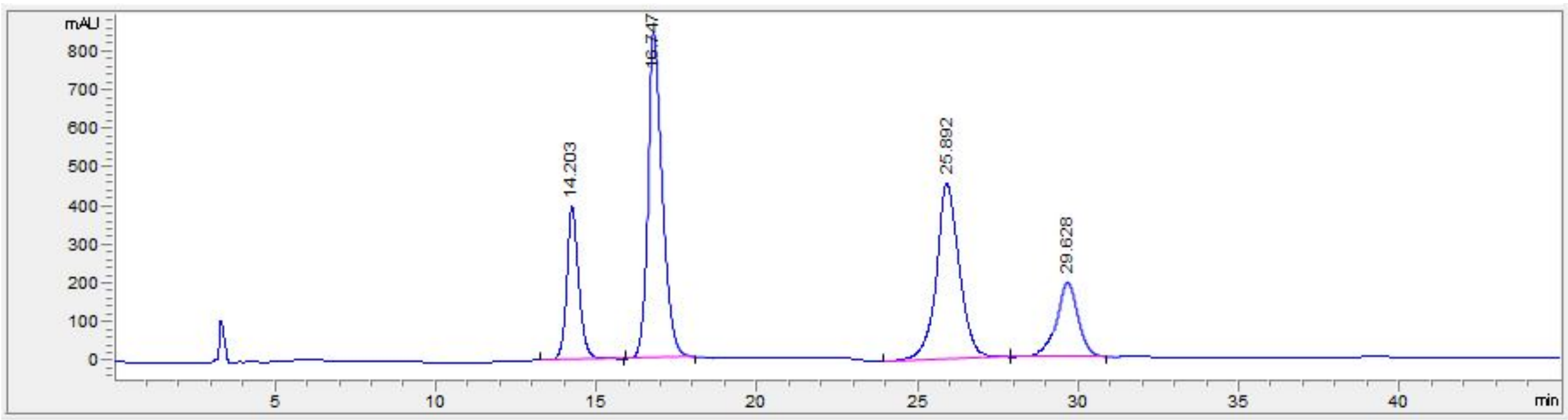


| # | Time | Area | Height | Width | Area% | Symmetry |
|---|---|---|---|---|---|---|
| 1 | 14.203 | 10081.1 | 395.5 | 0.3794 | 14.922 | 0.728 |
| 2 | 16.747 | 25855.8 | 845.8 | 0.4507 | 38.272 | 0.593 |
| 3 | 25.892 | 22530 | 456.2 | 0.7382 | 33.349 | 0.831 |
| 4 | 29.628 | 9091.9 | 193.3 | 0.7008 | 13.458 | 1.042 |

(*R*,*S*)-**8:** ($S_a$,*S*,*S*)-**[Ir]** and (*S*)-Me-BTM

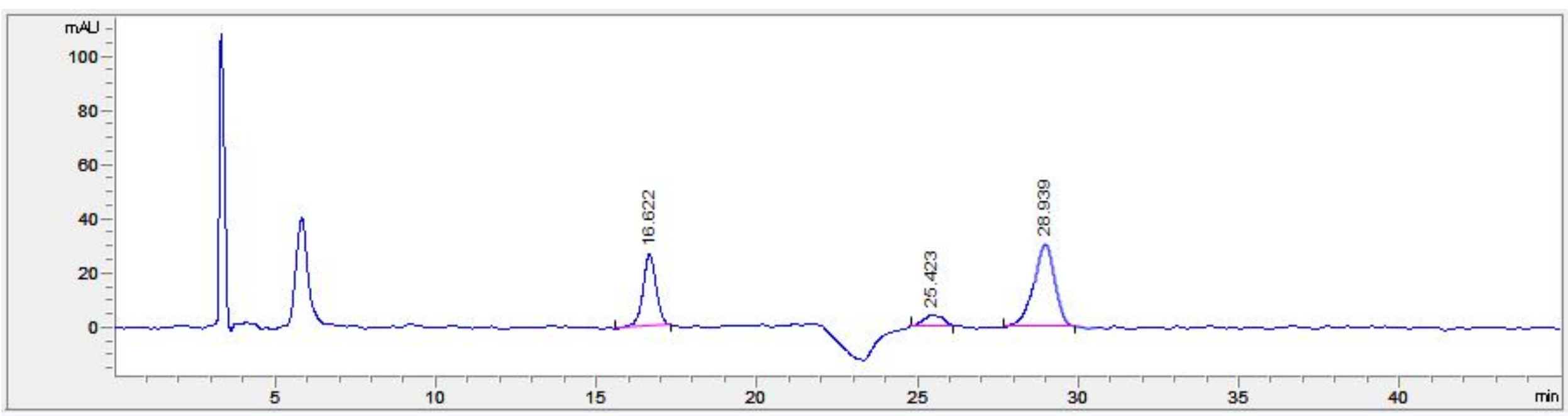


| # | Time | Area | Height | Width | Area% | Symmetry |
|---|---|---|---|---|---|---|
| 1 | 16.622 | 767.3 | 26.7 | 0.4398 | 33.177 | 0.993 |
| 2 | 25.423 | 162.7 | 3.9 | 0.6965 | 7.036 | 0.71 |
| 3 | 28.939 | 1382.8 | 30.5 | 0.6741 | 59.787 | 1.209 |

**Perfluorophenyl (2*R*,3*R*)-2-fluoro-3-phenyl-2-(4-(trifluoromethoxy)phenyl)pent-4-enoate ((*R*,*R*)-14):**

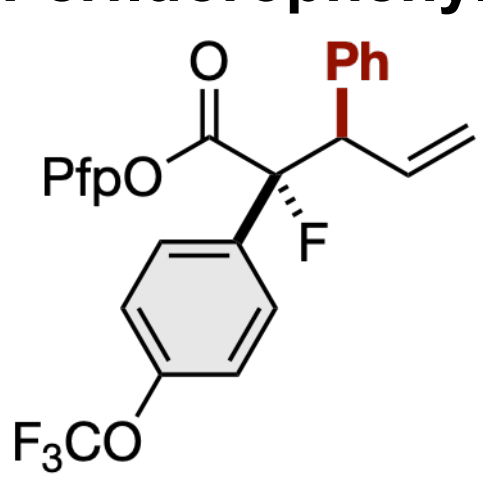


Prepared according to General Procedure B using pentafluorophenyl ester **1h** (40.4 mg, 0.1 mmol, 1 equiv.), *t*-butyl carbonate **2a** (35.1 mg, 0.15 mmol, 1.5 equiv.) and ($R_a$,*R*,*R*)-**[Ir]** (4.2 mg, 4 mol%), (*S*)-Me-BTM (3.8 mg, 20 mol%) and DIPEA (22.6 μL, 0.13 mmol, 1.3 equiv.) in 1,4-dioxane (0.3 ml, 0.3 M). The *title compound* was obtained (40.7 mg, 0.078 mmol, 78%) as a colorless oil following purification by column chromatography [$SiO_2$, 0.25% Ethyl Acetate in Petroleum Ether]. The enantiomeric ratio (>99:1 *er*) of the corresponding primary amide was determined by chiral HPLC in comparison with the racemate. Diastereomeric ratio (97:3) was determined by $^{19}$F-NMR analysis of the crude reaction mixture.

**IR (film):** 3033 (w), 2927 (w), 1783 (m), 1640 (w), 1596 (w), 1519 (s), 1472 (w), 1256 (s), 1206 (s), 1159 (s), 1010 (s), 997 (s), 700 (m) $cm^{-1}$.

**$^{1}$H NMR (500 MHz, $CDCl_3$):** δ 7.45 (d, *J* = 8.9 Hz, 2H), 7.19 – 7.06 (m, 7H), 6.38 (ddd, *J* = 17.1, 10.3, 8.8 Hz, 1H), 5.59 – 5.22 (m, 2H), 4.34 (dd, *J* = 33.0, 9.0 Hz, 1H).

**$^{13}$C NMR (126 MHz, $CDCl_3$):** δ 166.2 (d, *J* = 27.7 Hz), 149.6, 136.1, 134.1 (d, *J* = 5.1 Hz), 133.7 (d, *J* = 23.0 Hz), 129.5 (d, *J* = 2.3 Hz), 128.5, 127.6, 126.8 (d, *J* = 10.0 Hz), 120.7, 120.4 (q, *J* = 258.8 Hz), 120.2, 99.5 (d, *J* = 202.7 Hz), 57.8 (d, *J* = 19.0 Hz).

**$^{19}$F NMR (376 MHz, $CDCl_3$):** δ -57.94 (s, 3F), -151.40 – -151.59 (m, 2F), -156.58 (t, *J* = 21.7 Hz, 1F), -161.48 – -161.73 (m, 2F), -174.11 (d, *J* = 32.9 Hz, 1F).

**HRMS (EI):** m/z calcd for $[M]^+$ $C_{24}H_{13}F_9O_3$: 520.0721. Found: 520.0700.

**(2*R*,3*R*)-2-Fluoro-3-phenyl-2-(4-(trifluoromethoxy)phenyl)pent-4-enamide ((*R*,*R*)-14-$NH_2$):**

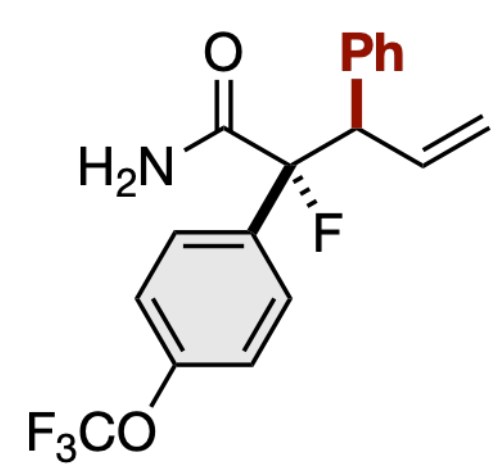


**IR (film):** 3433 (m), 3187 (br), 3088 (w), 1671 (s), 1603 (w), 1509 (m), 1454 (w), 1396 (w), 1280 (s), 1221 (s), 1161 (s) $cm^{-1}$.

**$^{1}$H NMR (500 MHz, $CDCl_3$):** δ 7.50 – 7.40 (m, 2H), 7.17 – 7.08 (m, 3H), 7.07 – 6.96 (m, 4H), 6.43 (s, 1H), 6.35 – 6.17 (m, 1H), 5.61 (s, 1H), 5.33 – 5.20 (m, 2H), 4.32 (dd, *J* = 35.0, 8.5 Hz, 1H).

**$^{13}$C NMR (126 MHz, $CDCl_3$):** δ 172.1 (d, *J* = 22.6 Hz), 149.1, 137.2, 135.5 (d, *J* = 22.5 Hz), 134.7 (d, *J* = 5.3 Hz), 129.5 (d, *J* = 2.0 Hz), 128.2, 127.2, 126.6 (d, *J* = 10.5 Hz), 120.5 (q, *J* = 257.5 Hz), 120.3, 119.6, 101.2 (d, *J* = 198.4 Hz), 57.4 (d, *J* = 19.4 Hz).

**$^{19}$F NMR (376 MHz, $CDCl_3$):** δ -57.94 (s, 3F), -171.39 (d, *J* = 34.6 Hz, 1F).

**HRMS (ESI):** m/z calcd for $[M+Na]^+$ $C_{18}H_{15}O_2NF_4Na$: 376.0931. Found: 376.0931.

HPLC analysis using a chiral column (Chiralpak IB 3μ column 22 ˚C, 0.5 ml/min, 95:5 hexane:isopropanol, 210 nm, $t_{major}$ = 17.627 min, $t_{minor}$ = 19.615 min, t = 23.294 min, t = 30.101 min).

(<u>+</u>)-**14:** (*rac*)-**[Ir]** and (*rac*)-Bn-BTM

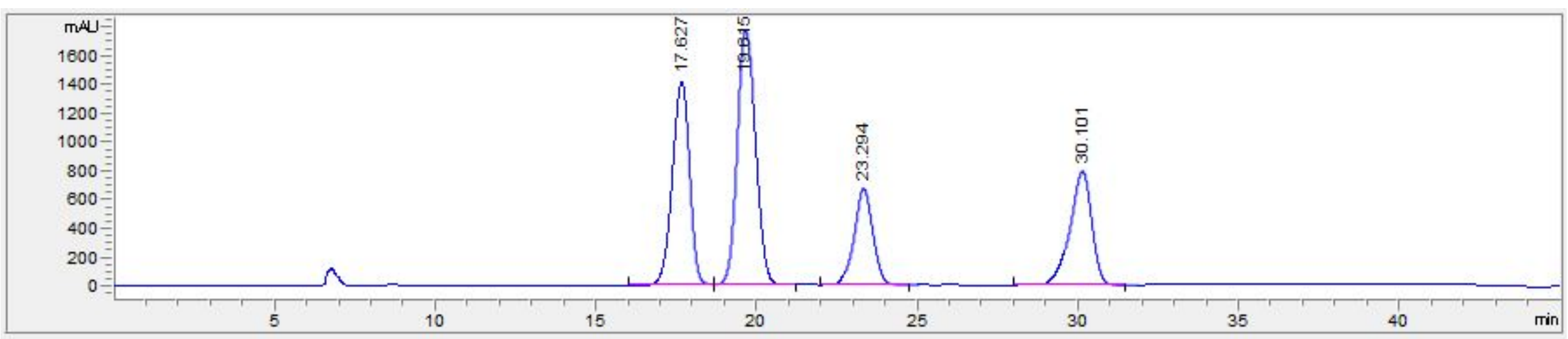


| # | Time | Area | Height | Width | Area% | Symmetry |
|---|---|---|---|---|---|---|
| 1 | 17.627 | 52405.4 | 1410.8 | 0.5854 | 28.027 | 1.106 |
| 2 | 19.615 | 71043.5 | 1765.5 | 0.6275 | 37.995 | 0.762 |
| 3 | 23.294 | 26745.7 | 668.8 | 0.5986 | 14.304 | 0.99 |
| 4 | 30.101 | 36788.8 | 787.7 | 0.7029 | 19.675 | 1.311 |

(*R*,*R*)-**14:** ($R_a$,*R*,*R*)-**[Ir]** and (*S*)-Me-BTM

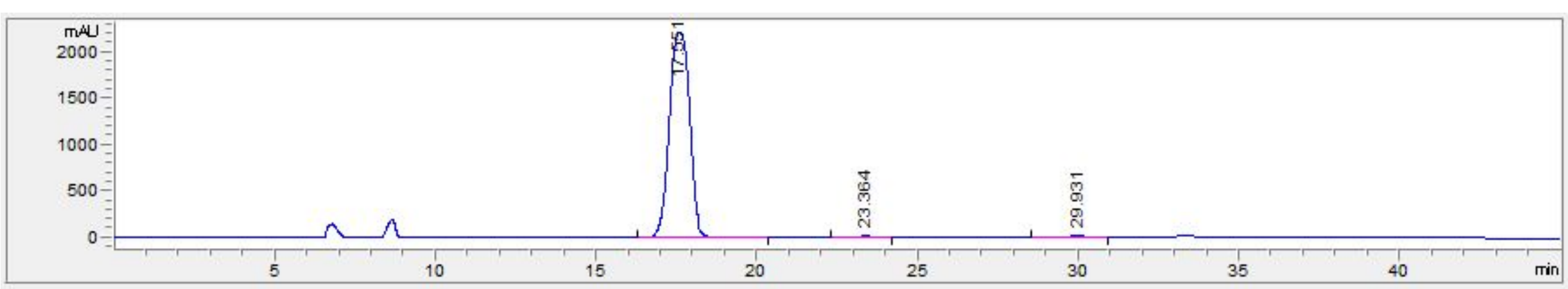


| # | Time | Area | Height | Width | Area% | Symmetry |
|---|---|---|---|---|---|---|
| 1 | 17.551 | 96030.2 | 2212.4 | 0.5984 | 98.420 | 0.896 |
| 2 | 23.364 | 672.1 | 17 | 0.5824 | 0.689 | 1.298 |
| 3 | 29.931 | 869.7 | 19.4 | 0.6658 | 0.891 | 1.318 |

**Perfluorophenyl (2*R*,3*S*)-2-fluoro-3-phenyl-2-(4-(trifluoromethoxy)phenyl)pent-4-enoate ((*R*,*S*)-14):** Prepared according to General Procedure B using pentafluorophenyl ester **1h** (40.4 mg, 0.1 mmol, 1 equiv.), *t*-butyl carbonate **2a** (35.1 mg, 0.15 mmol, 1.5 equiv.) and ($S_a$,*S*,*S*)-**[Ir]** (4.2 mg, 4 mol%), (*S*)-Me-BTM (3.8 mg, 20 mol%) and DIPEA (22.6 µL, 0.13 mmol, 1.3 equiv.) in 1,4-dioxane (0.3 ml, 0.3 M). The *title compound* was obtained (38.5 mg, 0.074 mmol, 74%) as a colorless oil following purification by column chromatography [$SiO_2$, 0.25% Ethyl Acetate in Petroleum Ether]. The enantiomeric ratio (>99:1 *er*) of the corresponding primary amide was determined by chiral HPLC in comparison with the racemate. Diastereomeric ratio (92:8) was determined by $^{19}$F-NMR analysis of the crude reaction mixture.

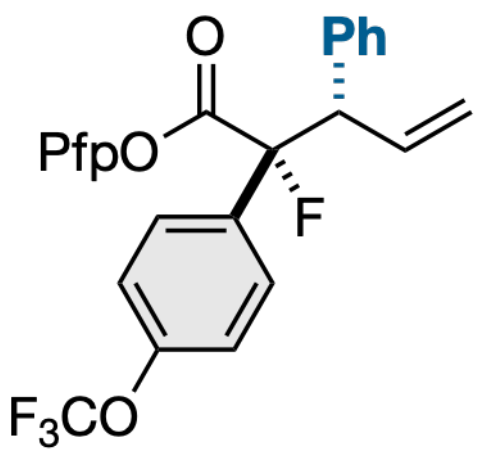


**IR (film):** 3036 (w), 2926 (w), 1801 (w), 1782 (m), 1640 (w), 1603 (w), 1519 (s), 1472 (w), 1257 (s), 1212 (s), 1171 (s), 1010 (s), 998 (s), 700 (m) cm$^{-1}$.

**$^1$H NMR (500 MHz, CDCl$_3$):** δ 7.74 (d, *J* = 8.9 Hz, 2H), 7.51 – 7.44 (m, 2H), 7.41 – 7.29 (m, 5H), ****6.38 (dt, J = 17.1, 9.6 Hz)***, 5.96 (ddd, *J* = 17.1, 10.3, 8.1 Hz, 1H), ****5.46 – 5.32 (m)***, 5.09 (d, *J* = 10.3 Hz, 1H), 4.93 (d, *J* = 17.1 Hz, 1H), 4.37 (dd, *J* = 33.5, 8.5 Hz, 1H).

**$^{13}$C NMR (126 MHz, CDCl$_3$):** δ 165.8 (d, *J* = 28.4 Hz), 150.0, 137.1, 134.3 (d, *J* = 23.1 Hz), 133.4 (d, *J* = 5.0 Hz), 129.5 (d, *J* = 2.9 Hz), 129.0, 128.2, 127.2 (d, *J* = 10.3 Hz), 121.0, 120.6 (q, *J* = 258.8 Hz), 120.3, 98.9 (d, *J* = 202.6 Hz), 57.3 (d, *J* = 18.8 Hz).

**$^{19}$F NMR (376 MHz, CDCl$_3$):** δ -57.94 (s, 3F), -151.41 – -151.56 (m, 2F), -156.58 (t, *J* = 21.7 Hz, 1F), -161.47 – -161.74 (m, 2F), -174.11 (d, *J* = 32.9 Hz, 1F).

**HRMS (EI):** m/z calcd for $[M]^+$ $C_{24}H_{13}F_9O_3$: 520.0721. Found: 520.0725.

**(2*R*,3*S*)-2-Fluoro-3-phenyl-2-(4-(trifluoromethoxy)phenyl)pent-4-enamide ((*R*,*S*)-14-NH$_2$):**

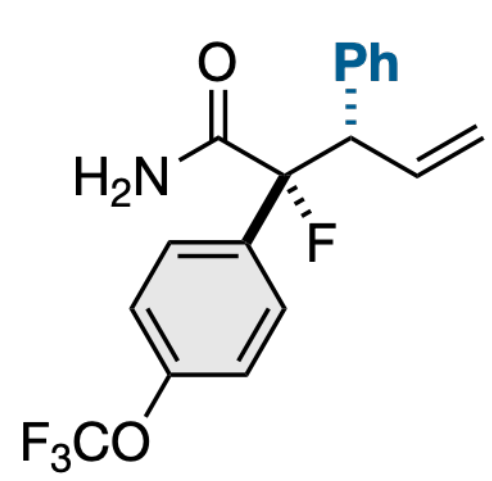

**IR (film):** 3442 (m), 3184 (br), 1672 (s), 1606 (w), 1511 (w), 1453 (w), 1400 (w), 1279 (s), 1222 (m), 1162 (m) $cm^{-1}$.

**$^1$H NMR (500 MHz, $CDCl_3$):** δ 7.77 – 7.66 (m, 2H), 7.41 (dt, *J* = 8.1, 1.6 Hz, 2H), 7.36 – 7.31 (m, 2H), 7.30 – 7.27 (m, 1H), 7.27 – 7.21 (m, 2H), ***6.43 (s)**, ***6.35 – 6.21 (m)**, 6.03 (s, 1H), 5.93 (ddd, *J* = 17.1, 10.3, 8.5 Hz, 1H), ***5.52 (s)**, ***5.31 – 5.23 (m)**, 5.11 (d, *J* = 4.9 Hz, 1H), 5.01 (dt, *J* = 10.2, 1.2 Hz, 1H), 4.87 (d, *J* = 17.1 Hz, 1H), 4.44 – 4.21 (dd, *J* = 36.0, 8.5 Hz, 1H).

**$^{13}$C NMR (126 MHz, $CDCl_3$):** δ 171.7 (d, *J* = 22.9 Hz), 149.4, 138.0, 135.9 (d, *J* = 22.6 Hz), 134.0 (d, *J* = 4.6 Hz), 129.9 (d, *J* = 2.3 Hz), 128.5, 127.6, 126.9 (d, *J* = 11.1 Hz), 120.7, 120.6 (q, *J* = 257.6 Hz), 119.8, 100.8 (d, *J* = 199.5 Hz), 56.8 (d, *J* = 18.8 Hz).

**$^{19}$F NMR (376 MHz, $CDCl_3$):** δ -57.84 (s, 3F), -172.73 (d, *J* = 36.1 Hz, 1F).

**HRMS (ESI):** m/z calcd for $[M+Na]^+$ $C_{18}H_{15}O_2NF_4Na$: 376.0931. Found: 376.0932.

HPLC analysis using a chiral column (Chiralpak IB 3μ column 22 ˚C, 0.5 ml/min, 95:5 hexane:isopropanol, 210 nm, $t_{major}$ = 30.101 min, $t_{minor}$ = 23.294 min, t = 17.627 min, t = 19.615 min).

(±)-**14:** (*rac*)-**[Ir]** and (*rac*)-Bn-BTM

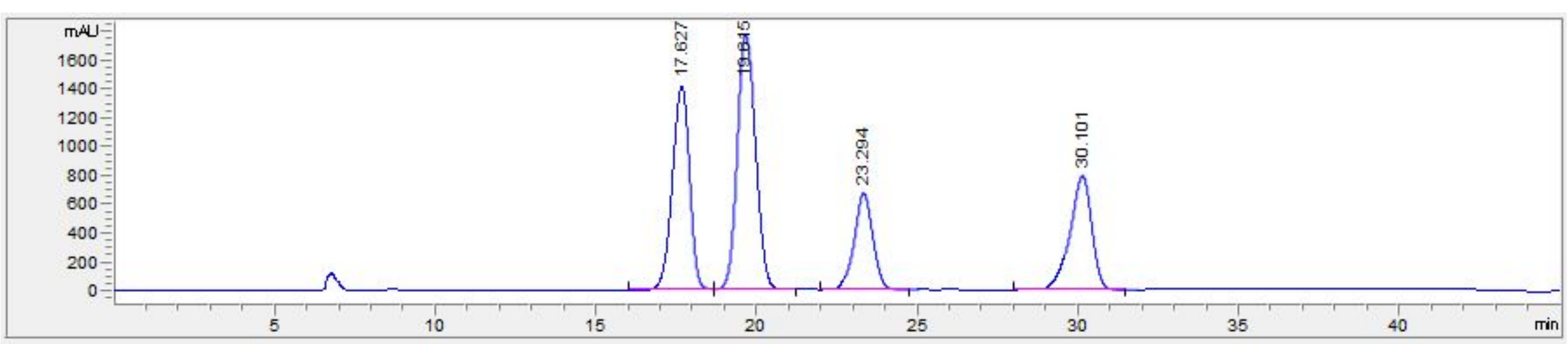


| # | Time | Area | Height | Width | Area% | Symmetry |
|---|---|---|---|---|---|---|
| 1 | 17.627 | 52405.4 | 1410.8 | 0.5854 | 28.027 | 1.106 |
| 2 | 19.615 | 71043.5 | 1765.5 | 0.6275 | 37.995 | 0.762 |
| 3 | 23.294 | 26745.7 | 668.8 | 0.5986 | 14.304 | 0.99 |
| 4 | 30.101 | 36788.8 | 787.7 | 0.7029 | 19.675 | 1.311 |

(*R*,*S*)-**14:** (*S$_a$*,*S*,*S*)-**[Ir]** and (*S*)-Me-BTM

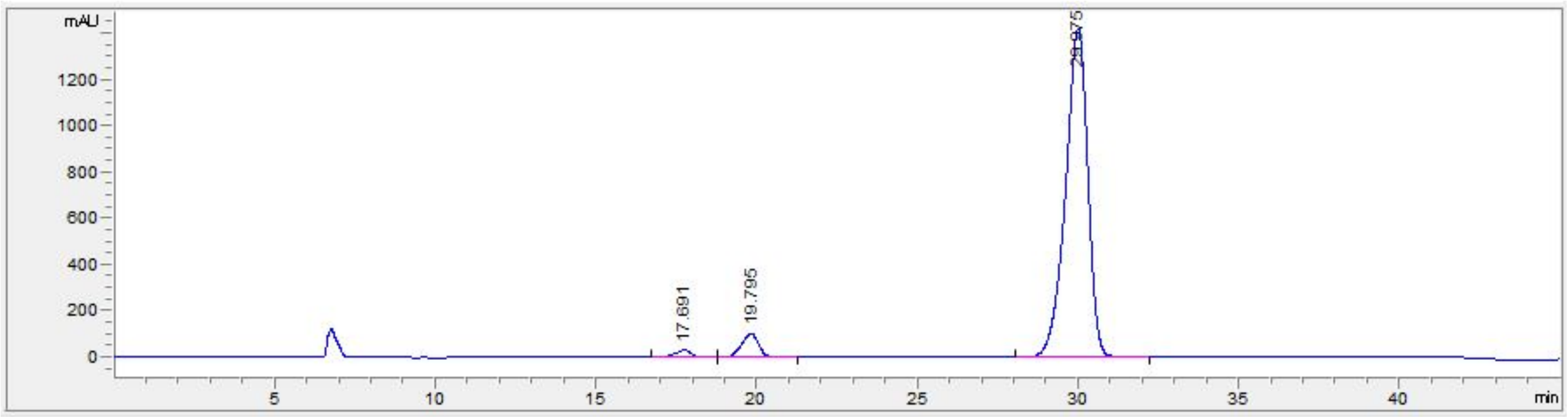


| # | Time | Area | Height | Width | Area% | Symmetry |
|---|---|---|---|---|---|---|
| 1 | 17.691 | 1064.2 | 29.5 | 0.5584 | 1.475 | 1.321 |
| 2 | 19.795 | 3723.3 | 102.6 | 0.5613 | 5.160 | 1.172 |
| 3 | 29.975 | 67375.1 | 1422.9 | 0.7144 | 93.366 | 1.269 |

**Perfluorophenyl (2*R*,3*R*)-2-fluoro-3-phenyl-2-(4-(trifluoromethyl)phenyl)pent-4-enoate ((*R*,*R*)-13):** Prepared according to General Procedure B using pentafluorophenyl ester **1i** (38.8 mg, 0.1 mmol, 1 equiv.), *t*-butyl carbonate **2a** (35.1 mg, 0.15 mmol, 1.5 equiv.), ($R_a$,*R*,*R*)-**[Ir]** (4.2 mg, 4 mol%), (*S*)-Me-BTM (3.8 mg, 20 mol%) and DIPEA (22.6 µL, 0.13 mmol, 1.3 equiv.) in 1,4-dioxane (0.3 ml, 0.3 M). The *title compound* was obtained (38.4 mg, 0.076 mmol, 76%) as a colorless oil following purification by column chromatography [$SiO_2$, 0.25% Ethyl Acetate in Petroleum Ether]. The enantiomeric ratio (>99:1 *er*) of the corresponding primary amide was determined by chiral HPLC in comparison with the racemate. Diastereomeric ratio (95:5) was determined by $^{19}$F-NMR analysis of the crude reaction mixture.

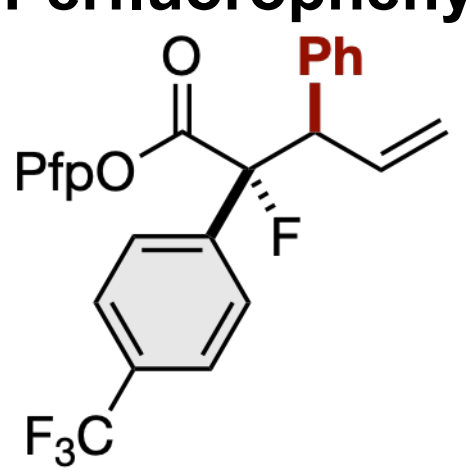


**IR (film):** 3033 (w), 1784 (m), 1640 (w), 1620 (w), 1519 (s), 1472 (w), 1326 (s), 1172 (m), 1129 (m), 1012 (s), 998 (s) cm$^{-1}$.

**$^1$H NMR (500 MHz, $CDCl_3$):** δ 7.56 (d, *J* = 8.4 Hz, 2H), 7.53 (d, *J* = 8.5 Hz, 2H), 7.20 – 7.12 (m, 3H), 7.10 (dt, *J* = 7.0, 1.5 Hz, 2H), 6.38 (ddd, *J* = 17.1, 10.2, 8.8 Hz, 1H), ****5.95 (ddd, J = 17.7, 10.3, 8.3 Hz)***, 5.44 – 5.34 (m, 2H), ****5.08 (d, J = 10.3 Hz)***, ****4.92 (d, J = 17.1 Hz)***, 4.38 (dd, *J* = 33.0, 9.0 Hz, 1H).

**$^{13}$C NMR (126 MHz, $CDCl_3$):** δ 166.1 (d, *J* = 27.9 Hz), 139.0 (d, *J* = 22.7 Hz), 136.0, 134.0 (d, *J* = 5.1 Hz), 131.2 (q, *J* = 32.4 Hz), 129.5 (d, *J* = 2.3 Hz), 128.6, 127.7, 125.6 (d, *J* = 10.1 Hz), 125.5 – 125.4 (m), 123.8 (q, *J* = 272.7 Hz), 120.4, 99.6 (d, *J* = 203.2 Hz), 57.8 (d, *J* = 18.9 Hz).

**$^{19}$F NMR (376 MHz, CDCl$_3$):** δ -62.92 (s, 3F), -151.40 – -151.61 (m, 3F), -156.46 (t, *J* = 21.7 Hz, 1F), -161.39 – -161.65 (m, 2F), -174.69 (d, *J* = 33.0 Hz, 1F).

**HRMS (EI):** m/z calcd for $[M]^+$ $C_{24}H_{13}F_9O_2$: 504.0772. Found: 504.0772.

**(2*R*,3*R*)-2-Fluoro-3-phenyl-2-(4-(trifluoromethyl)phenyl)pent-4-enamide ((*R*,*R*)-13-NH$_2$):**

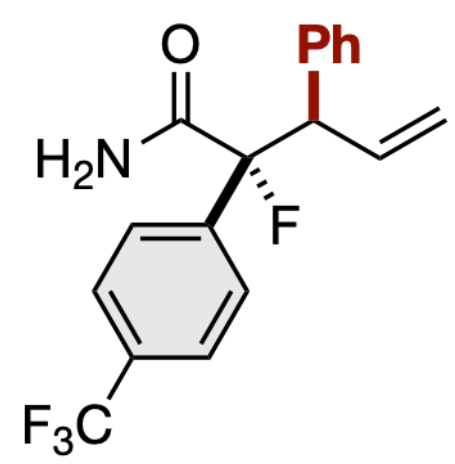


**IR (film):** 3435 (m), 3184 (br), 1673 (s), 1603 (w), 1493 (w), 1454 (w), 1415 (w), 1334 (s), 1130 (s) cm$^{-1}$.

**$^1$H NMR (500 MHz, CDCl$_3$):** δ 7.55 (d, *J* = 8.3 Hz, 2H), 7.44 (d, *J* = 8.3 Hz, 2H), 7.17 – 7.08 (m, 3H), 7.08 – 7.01 (m, 2H), 6.44 (s, 1H), 6.36 – 6.21 (m, 1H), 5.48 (s, 1H), 5.34 – 5.23 (m, 2H), 4.36 (dd, *J* = 35.0, 8.5 Hz, 1H).

**$^1$H NMR (500 MHz, CDCl$_3$):** δ 7.55 (d, *J* = 8.3 Hz, 2H), 7.44 (d, *J* = 8.3 Hz, 2H), 7.17 – 7.08 (m, 3H), 7.08 – 7.01 (m, 2H), 6.44 (s, 1H), 6.36 – 6.21 (m, 1H), ****6.05 (s)***, ****5.97 – 5.87 (m)***, 5.48 (s, 1H), 5.34 – 5.23 (m, 2H), ****5.08 (s)***, ****5.00 (d, J = 10.3 Hz)***, ****4.87 (d, J = 17.1 Hz)***, 4.36 (dd, *J* = 35.0, 8.5 Hz, 1H).

**$^{13}$C NMR (126 MHz, CDCl$_3$):** δ 171.7 (d, *J* = 22.8 Hz), 140.7 (d, *J* = 22.1 Hz), 137.1, 134.6 (d, *J* = 5.5 Hz), 130.3 (d, *J* = 32.6 Hz), 129.5 (d, *J* = 2.2 Hz), 128.3, 127.3, 125.4 (d, *J* = 10.6 Hz), 125.0 (t, *J* = 3.1 Hz), 119.7, 101.3 (d, *J* = 199.0 Hz), 57.3 (d, *J* = 19.3 Hz).

**$^{19}$F NMR (376 MHz, CDCl$_3$):** δ -62.80 (s, 3F), -172.08 (d, *J* = 35.2 Hz, 1F).

**HRMS (ESI):** m/z calcd for $[M\text{-}HF]^+$ $C_{18}H_{15}ONF_3$: 318.1100. Found: 318.1100.

HPLC analysis using a chiral column (Chiralpak IB 3µ column 22 ˚C, 0.5 ml/min, 95:5 hexane:isopropanol, 210 nm, $t_{major}$ = 19.391 min, $t_{minor}$ = 22.039 min, t = 26.175 min, t = 33.546 min).

(±)-**13:** (*rac*)-**[Ir]** and (*rac*)-Bn-BTM

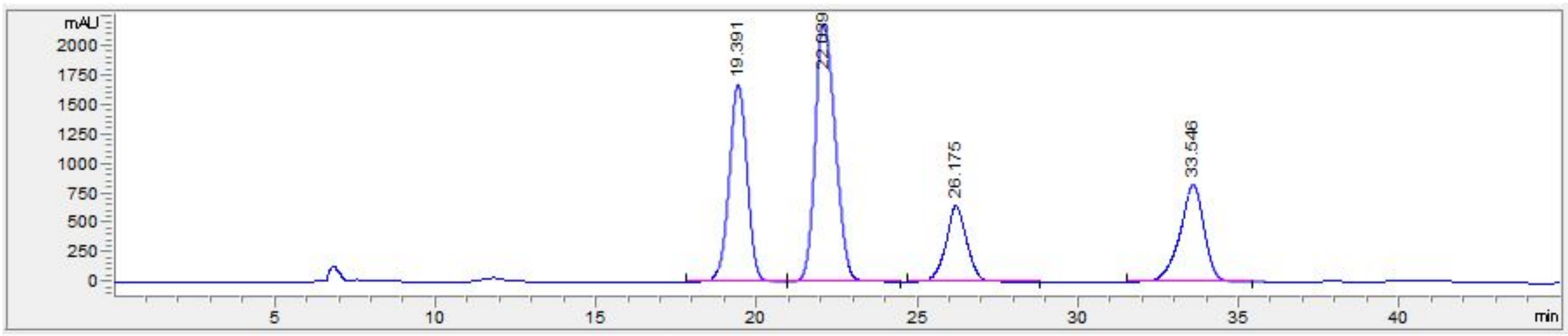


| # | Time | Area | Height | Width | Area% | Symmetry |
|---|---|---|---|---|---|---|
| 1 | 19.391 | 63927 | 1659.9 | 0.5953 | 27.907 | 0.973 |
| 2 | 22.039 | 94728.1 | 2165 | 0.6705 | 41.353 | 0.662 |
| 3 | 26.175 | 28342.8 | 643.4 | 0.6539 | 12.373 | 0.902 |
| 4 | 33.546 | 42074.1 | 818.8 | 0.7695 | 18.367 | 1.187 |

(*R*,*R*)-**13:** ($R_a$,*R,R*)-**[Ir]** and (*S*)-Me-BTM

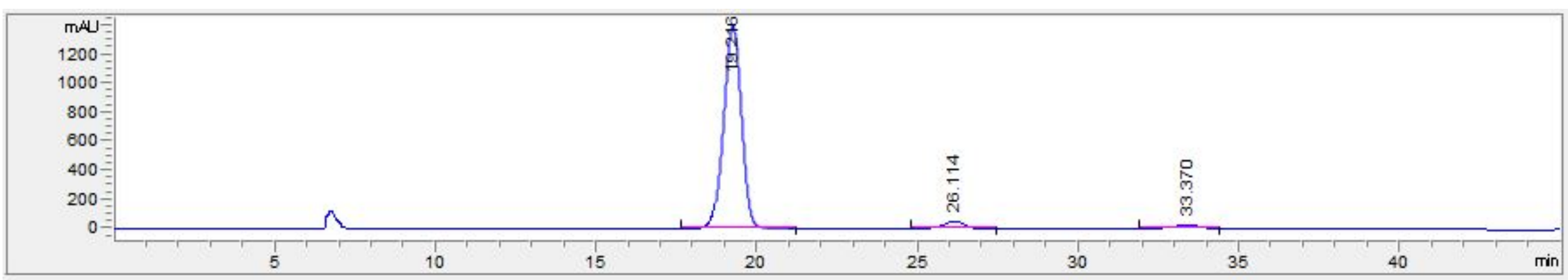


| # | Time | Area | Height | Width | Area% | Symmetry |
|---|---|---|---|---|---|---|
| 1 | 19.216 | 53035.2 | 1382.1 | 0.5997 | 93.742 | 1.007 |
| 2 | 26.114 | 2183 | 50.8 | 0.653 | 3.859 | 1.21 |
| 3 | 33.37 | 1357.7 | 27.3 | 0.7252 | 2.400 | 1.362 |

**Perfluorophenyl (2*R*,3*S*)-2-fluoro-3-phenyl-2-(4-(trifluoromethyl)phenyl)pent-4-enoate ((*R*,*S*)-13):** Prepared according to General Procedure B using pentafluorophenyl ester **1i** (38.8 mg, 0.1 mmol, 1 equiv.), *t*-butyl carbonate **2a** (35.1 mg, 0.15 mmol, 1.5 equiv.) and ($S_a$,*S,S*)-**[Ir]** (4.2 mg, 4 mol%), (*S*)-Me-BTM (3.8 mg, 20 mol%) and DIPEA (22.6 μL, 0.13 mmol, 1.3 equiv.) in 1,4-dioxane (0.3 ml, 0.3 M). The *title compound* was obtained (35.8 mg, 0.071 mmol, 71%) as a white solid following purification by column chromatography [$SiO_2$, 0.25% Ethyl Acetate in Petroleum Ether]. The enantiomeric ratio (>99:1 *er*) of the corresponding primary amide was determined by chiral HPLC in comparison with the racemate. Diastereomeric ratio (88:12) was determined by $^{19}$F-NMR analysis of the crude reaction mixture.

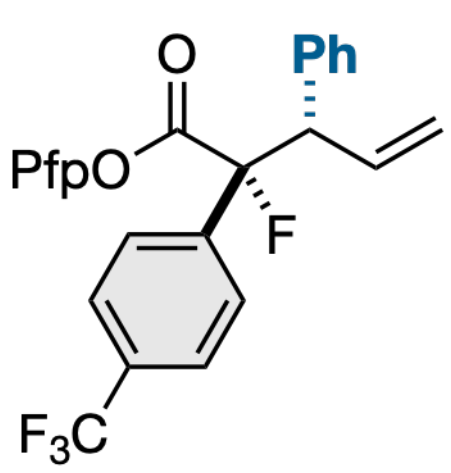

**IR (film):** 3036 (w), 2928 (w), 1801 (w), 1782 (w), 1640 (w), 1620 (w), 1520 (s), 1472 (w), 1326 (s), 1172 (m), 1131 (m), 1011 (s), 999 (s) $cm^{-1}$.

**$^1$H NMR (500 MHz, $CDCl_3$):** δ 7.84 (d, *J* = 8.3 Hz, 2H), 7.74 (d, *J* = 8.3 Hz, 2H), 7.53 – 7.44 (m, 2H), 7.42 – 7.30 (m, 3H), ***6.38 (ddd, J = 17.1, 10.3, 8.7 Hz)**, 5.95 (ddd, *J* = 17.1, 10.3, 8.2 Hz, 1H), ***5.50 – 5.31 (m)**, 5.08 (d, *J* = 10.3 Hz, 1H), 4.92 (d, *J* = 17.1 Hz, 1H), 4.38 (dd, *J* = 33.5, 8.0 Hz, 1H).

**$^{13}$C NMR (126 MHz, $CDCl_3$):** δ 165.6 (d, *J* = 28.2 Hz), 139.6 (d, *J* = 23.0 Hz), 137.0, 133.2 (d, *J* = 5.0 Hz), 131.6 (q, *J* = 32.8 Hz), 129.5 (d, *J* = 2.9 Hz), 129.0, 128.2, 125.9 (d, *J* = 10.6 Hz), 126.0 – 125.6 (m), 123.9 (q, *J* = 272.7 Hz), 120.4, 99.0 (d, *J* = 203.3 Hz), 57.4 (d, *J* = 18.8 Hz).

**$^{19}$F NMR (376 MHz, $CDCl_3$):** δ -62.85 (s, 3F), -151.72 – -151.86 (m, 2F), -156.73 (t, *J* = 21.7 Hz, 1F), -161.65 – -161.89 (m, 2F), -173.71 (d, *J* = 33.5 Hz, 1F).

**HRMS (EI):** m/z calcd for $[M]^+$ $C_{24}H_{13}F_9O_2$: 504.0772. Found: 504.0771.

**(2*R*,3*S*)-2-Fluoro-3-phenyl-2-(4-(trifluoromethyl)phenyl)pent-4-enamide ((*R*,*S*)-13-$NH_2$):**

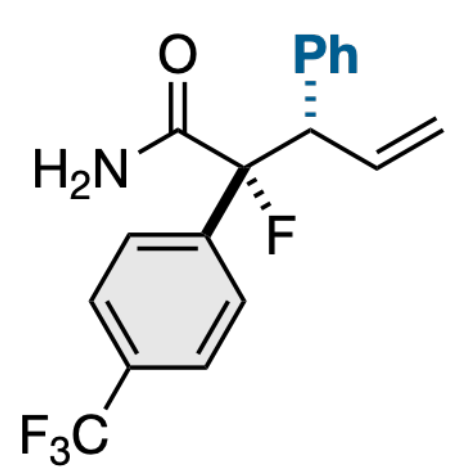


**IR (film):** 3444 (m), 3181 (br), 1673 (s), 1605 (w), 1453 (w), 1413 (w), 1336 (s), 1127 (s) $cm^{-1}$.

**$^1$H NMR (500 MHz, $CDCl_3$):** δ 7.82 (d, *J* = 8.2 Hz, 2H), 7.66 (d, *J* = 8.3 Hz, 2H), 7.42 (dt, *J* = 8.0, 1.6 Hz, 2H), 7.38 – 7.31 (m, 2H), 7.31 – 7.27 (m, 1H), ***6.44 (s)**, ****6.27 (ddd, J = 17.9, 9.9, 8.4 Hz)***, 6.04 (s, 1H), 5.92 (ddd, *J* = 17.1, 10.3, 8.6 Hz, 1H), ****5.56 (s,)***, ****5.37 – 5.22 (m)***, 5.14 (s, 1H), 5.00 (d, *J* = 10.3 Hz, 1H), 4.87 (d, *J* = 17.1 Hz, 1H), 4.35 (dd, *J* = 36.0 , 8.5 Hz, 1H).

**$^{13}$C NMR (126 MHz, CDCl$_3$):** δ 171.3 (d, *J* = 22.9 Hz), 141.1 (d, *J* = 22.4 Hz), 137.9, 133.8 (d, *J* = 4.5 Hz), 130.7 (q, *J* = 32.5 Hz), 129.9 (d, *J* = 2.0 Hz), 128.6, 127.6, 125.7 (d, *J* = 11.2 Hz), 124.1 (q, *J* = 273.0 Hz), 125.5 – 125.2 (m), 119.9, 100.8 (d, *J* = 200.2 Hz), 56.9 (d, *J* = 18.8 Hz).

**$^{19}$F NMR (376 MHz, CDCl$_3$):** δ -62.72 (s, 3F), -173.30 (d, *J* = 36.1 Hz, 1F).

**HRMS (ESI):** m/z calcd for $[M-HF]^+$ $C_{18}H_{15}ONF_3$: 318.1100. Found: 318.1100.

HPLC analysis using a chiral column (Chiralpak IB 3μ column 22 ˚C, 0.5 ml/min, 95:5 hexane:isopropanol, 210 nm, $t_{major}$ = 33.546 min, $t_{minor}$ = 26.175 min, t = 19.391 min, t = 22.039 min).

(±)-**13:** (*rac*)-**[Ir]** and (*rac*)-Bn-BTM

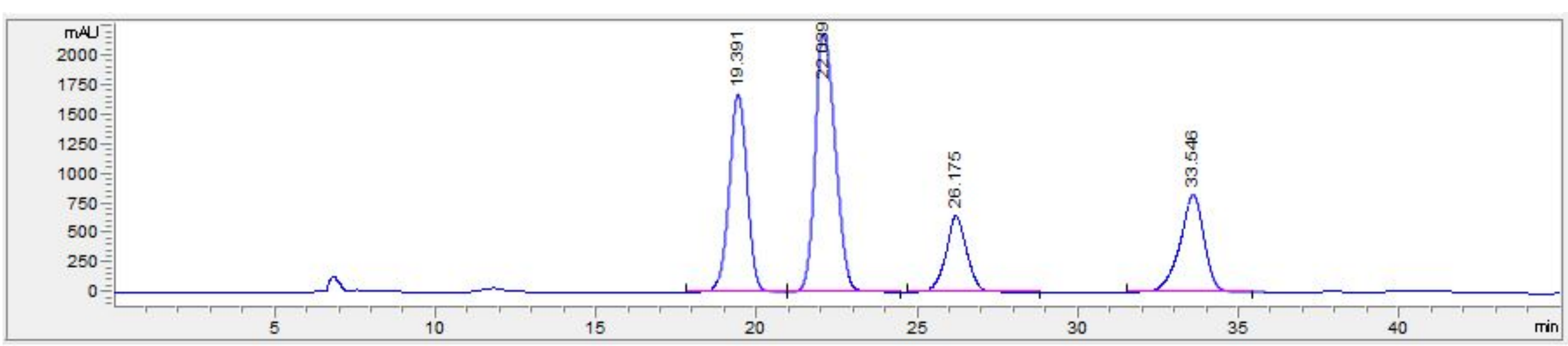


| # | Time | Area | Height | Width | Area% | Symmetry |
|---|---|---|---|---|---|---|
| 1 | 19.391 | 63927 | 1659.9 | 0.5953 | 27.907 | 0.973 |
| 2 | 22.039 | 94728.1 | 2165 | 0.6705 | 41.353 | 0.662 |
| 3 | 26.175 | 28342.8 | 643.4 | 0.6539 | 12.373 | 0.902 |
| 4 | 33.546 | 42074.1 | 818.8 | 0.7695 | 18.367 | 1.187 |

(*R*,*S*)-**13:** ($S_a$,*S*,*S*)-**[Ir]** and (*S*)-Me-BTM

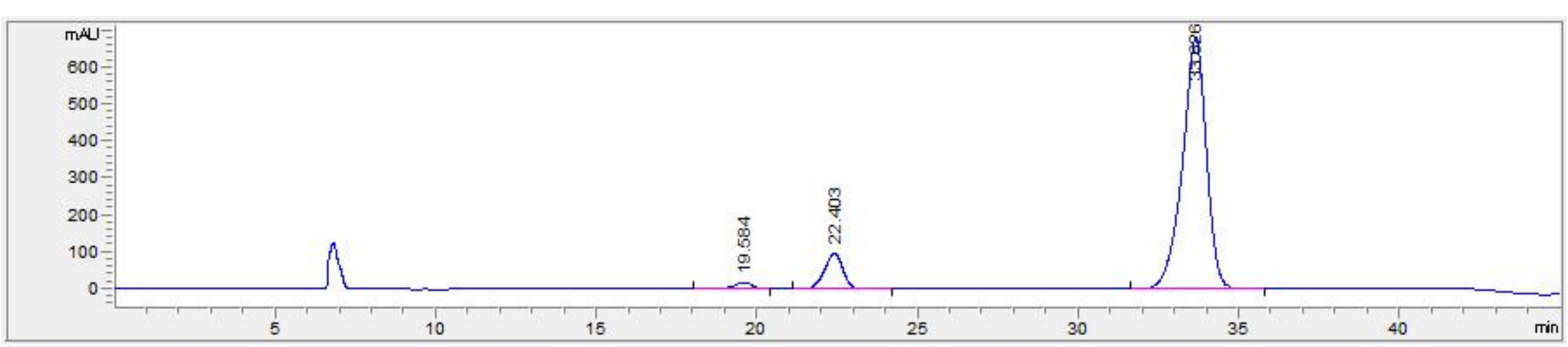


| # | Time | Area | Height | Width | Area% | Symmetry |
|---|---|---|---|---|---|---|
| 1 | 19.584 | 740.6 | 18.1 | 0.6049 | 1.893 | 1.435 |
| 2 | 22.403 | 3784.3 | 97.1 | 0.5968 | 9.672 | 1.14 |
| 3 | 33.626 | 34603 | 679.2 | 0.7564 | 88.436 | 1.207 |

**Perfluorophenyl (2*R*,3*R*)-2-fluoro-2-(naphthalen-2-yl)-3-phenylpent-4-enoate ((*R*,*R*)-4):**

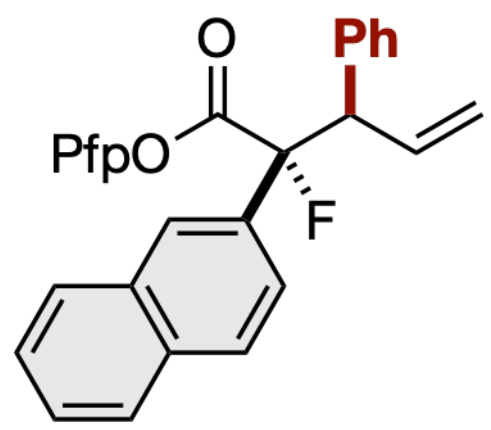


Prepared according to General Procedure B using pentafluorophenyl ester **1j** (37 mg, 0.1 mmol, 1 equiv.), *t*-butyl carbonate **2a** (35.1 mg, 0.15 mmol, 1.5 equiv.) and ($R_a$,*R*,*R*)-**[Ir]** (4.2 mg, 4 mol%), (*S*)-Me-BTM (3.8 mg, 20 mol%) and DIPEA (22.6 μL, 0.13 mmol, 1.3 equiv.) in 1,4-dioxane (0.3 ml, 0.3 M). The *title compound* was obtained (44.2 mg, 0.091 mmol, 91%) as a white solid following purification by column chromatography [$SiO_2$, 0.25% Ethyl Acetate in Petroleum Ether]. The enantiomeric ratio (>99:1 *er*) of the corresponding primary amide was determined by chiral HPLC in comparison with the racemate. Diastereomeric ratio (97:3) was determined by $^{19}$F-NMR analysis of the crude reaction mixture.

**IR (film):** 3063 (w), 3031 (w), 1782 (m), 1639 (w), 1601 (w), 1518 (s), 1203 (m), 1172 (m), 1008 (s), 995 (s), 748 (s), 699 (s) cm$^{-1}$.

**$^{1}$H NMR (500 MHz, $CDCl_3$):** δ 7.93 (d, *J* = 2.0 Hz, 1H), 7.81 – 7.73 (m, 3H), 7.55 (dd, *J* = 8.7, 1.9 Hz, 1H), 7.50 – 7.43 (m, 2H), 7.24 – 7.15 (m, 2H), 7.14 – 7.02 (m, 3H), 6.44 (ddd, *J* = 17.1, 10.2, 8.8 Hz, 1H), 5.44 (d, *J* = 17.1 Hz, 1H), 5.39 (d, *J* = 10.2 Hz, 1H), 4.55 (dd, *J* = 33.0, 9.0 Hz , 1H).

**$^{13}$C NMR (126 MHz, $CDCl_3$):** δ 166.5 (d, *J* = 28.3 Hz), 136.6, 134.6 (d, *J* = 5.1 Hz), 133.2, 132.8 (d, *J* = 1.8 Hz), 132.5 (d, *J* = 22.2 Hz), 129.6 (d, *J* = 2.3 Hz), 128.6, 128.4, 128.3 (d, *J* = 2.0 Hz), 127.7, 127.4, 125.0, 126.6, 125.0 (d, *J* = 11.0 Hz), 122.3 (d, *J* = 8.5 Hz), 120.0, 100.0 (d, *J* = 201.8 Hz), 57.4 (d, *J* = 19.1 Hz).

**$^{19}$F NMR (376 MHz, $CDCl_3$):** δ -151.23 – -151.40 (m, 2F), -156.91 (t, *J* = 21.6 Hz, 1F), -161.67 – -161.93 (m, 2F), -173.50 (d, *J* = 33.0 Hz, 1F).

**HRMS (EI):** m/z calcd for $[M]^+$ $C_{27}H_{16}F_6O_2$: 486.1054. Found: 486.1058.

**(2*R*,3*R*)-2-Fluoro-2-(naphthalen-2-yl)-3-phenylpent-4-enamide ((*R*,*R*)-4-$NH_2$):**

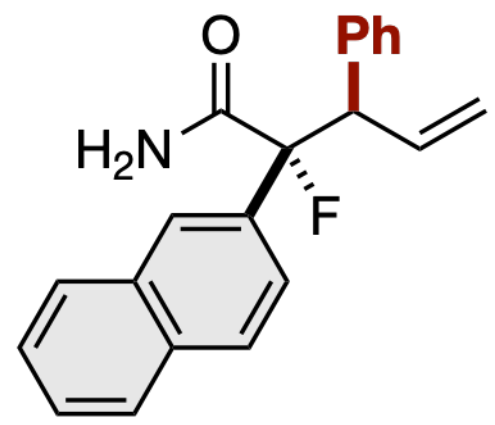


**IR (film):** 3438 (m), 3177 (br), 3061 (w), 1672 (s), 1599 (m), 1492 (w), 1453 (w), 1392 (m) $cm^{-1}$.

**$^1$H NMR (500 MHz, $CDCl_3$):** δ 7.89 (d, *J* = 1.9 Hz, 1H), 7.81 – 7.69 (m, 2H), 7.67 (d, *J* = 8.7 Hz, 1H), 7.55 (dd, *J* = 8.7, 1.9 Hz, 1H), 7.48 – 7.34 (m, 2H), 7.18 – 7.09 (m, 2H), 7.10 – 6.98 (m, 3H), 6.47 (s, 1H), 6.34 (ddd, *J* = 17.0, 10.3, 8.3 Hz, 1H), 5.51 (s, 1H), 5.32 – 5.24 (m, 2H), 4.52 (dd, *J* = 35.0, 10.0 Hz, 1H).

**$^{13}$C NMR (126 MHz, $CDCl_3$):** δ 172.4 (d, *J* = 22.9 Hz), 137.6, 135.3 (d, *J* = 5.3 Hz), 134.4 (d, *J* = 22.0 Hz), 132.9, 132.7 (d, *J* = 1.8 Hz), 129.6 (d, *J* = 2.3 Hz), 128.5, 128.2, 127.8 (d, *J* = 2.1 Hz), 127.6, 127.0, 126.4, 126.2, 124.2 (d, *J* = 11.4 Hz), 122.7 (d, *J* = 9.3 Hz), 119.3, 101.8 (d, *J* = 197.5 Hz), 57.0 (d, *J* = 19.5 Hz).

**$^{19}$F NMR (376 MHz, $CDCl_3$):** δ -170.49 (d, *J* = 34.9 Hz, 1F).

**HRMS (ESI):** m/z calcd for $[M+Na]^+$ $C_{21}H_{18}ONFNa$: 342.1265. Found: 342.1265.

HPLC analysis using a chiral column (Cellulose 3 3μ column 22 ˚C, 1.0 ml/min, 95:5 hexane:isopropanol, 210 nm, $t_{major}$ = 21.097 min, $t_{minor}$ = 41.953 min, t = 34.570 min, t = 60.464 min).

(±)-**4:** (*rac*)-**[Ir]** and (*rac*)-Bn-BTM

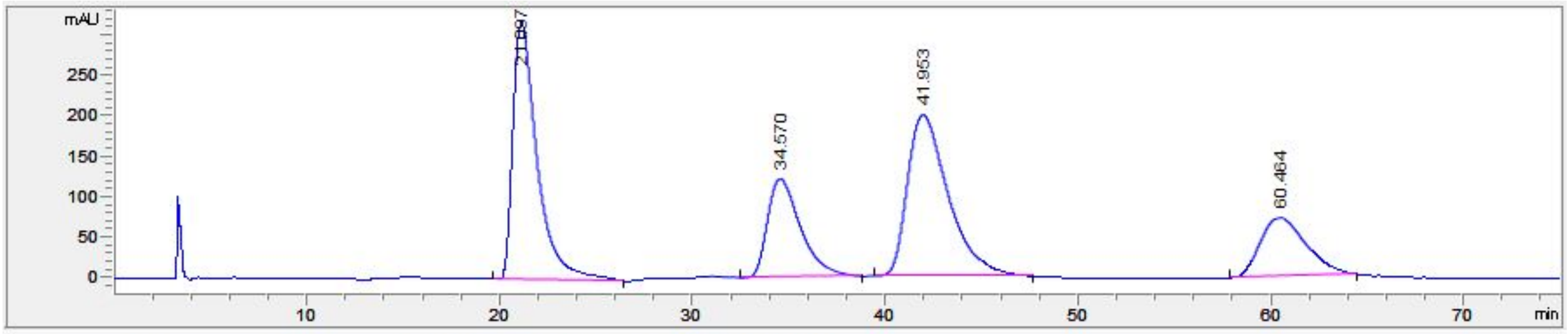


| # | Time | Area | Height | Width | Area% | Symmetry |
|---|---|---|---|---|---|---|
| 1 | 21.097 | 27632.9 | 316.7 | 1.286 | 32.825 | 0.477 |
| 2 | 34.57 | 14657.3 | 122.3 | 1.4787 | 17.411 | 0.65 |
| 3 | 41.953 | 29823.7 | 200.8 | 2.4758 | 35.427 | 0.602 |
| 4 | 60.464 | 12069.7 | 72.9 | 1.9644 | 14.337 | 0.717 |

(*R*,*R*)-**4:** ($R_a$,*R*,*R*)-**[Ir]** and (*S*)-Me-BTM

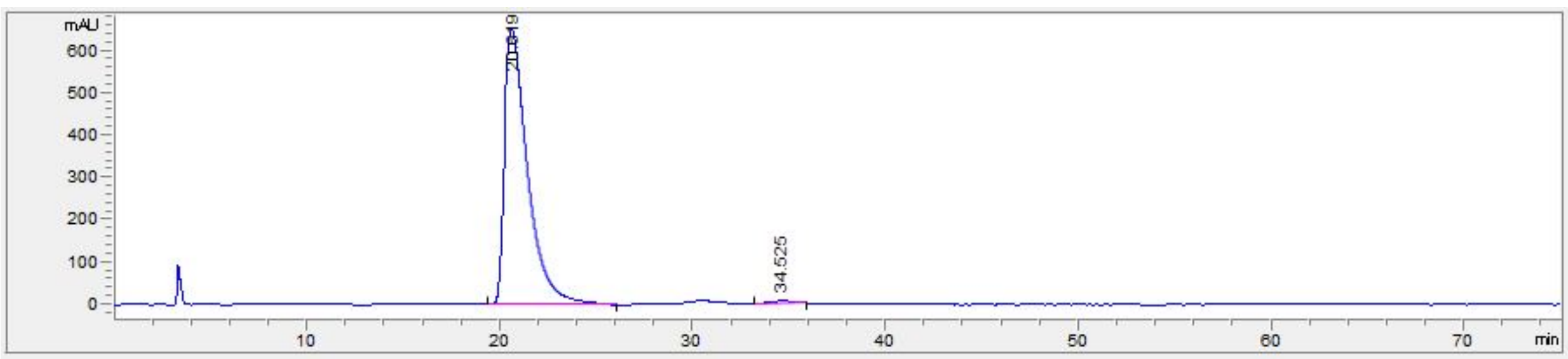


| # | Time | Area | Height | Width | Area% | Symmetry |
|---|---|---|---|---|---|---|
| 1 | 20.619 | 55329.5 | 650.1 | 1.267 | 98.971 | 0.464 |
| 2 | 34.525 | 575.2 | 6.5 | 1.0467 | 1.029 | 0.884 |

**Perfluorophenyl (2*R*,3*S*)-2-fluoro-2-(naphthalen-2-yl)-3-phenylpent-4-enoate ((*R*,*S*)-4):**

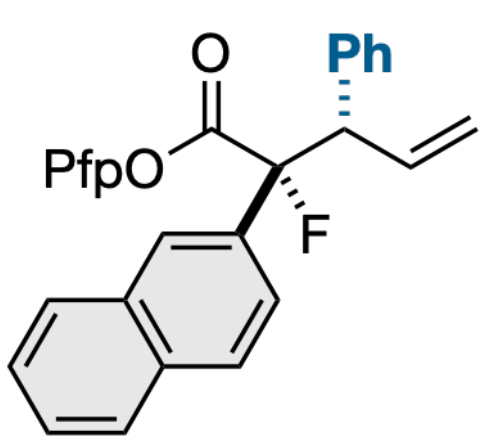


Prepared according to General Procedure B using pentafluorophenyl ester **1j** (37 mg, 0.1 mmol, 1 equiv.), *t*-butyl carbonate **2a** (35.1 mg, 0.15 mmol, 1.5 equiv.) and ($S_a$,*S*,*S*)-**[Ir]** (4.2 mg, 4 mol%), (*S*)-Me-BTM (3.8 mg, 20 mol%) and DIPEA (22.6 μL, 0.13 mmol, 1.3 equiv.) in 1,4-dioxane (0.3 ml, 0.3 M). The *title compound* was obtained (40.3 mg, 0.083 mmol, 83%) as a white solid following purification by column chromatography [$SiO_2$, 0.25% Ethyl Acetate in Petroleum Ether]. The enantiomeric ratio (>99:1 *er*) of the corresponding primary amide was determined by chiral HPLC in comparison with the racemate. Diastereomeric ratio (95:5) was determined by $^{19}$F-NMR analysis of the crude reaction mixture.

**IR (film):** 3063 (w), 3032 (w), 1779 (m), 1640 (w), 1601 (w), 1517 (s), 1471 (w), 1214 (m), 1174 (m), 995 (s), 750 (s), 700 (s) $cm^{-1}$.

**$^{1}$H NMR (400 MHz, $CDCl_3$):** δ 8.20 (d, *J* = 1.9 Hz, 1H), 7.99 – 7.86 (m, 3H), 7.79 (dd, *J* = 8.8, 2.0 Hz, 1H), 7.62 – 7.50 (m, 4H), 7.43 – 7.30 (m, 3H), 6.01 (ddd, *J* = 17.1, 10.3, 7.9 Hz, 1H), 5.03 (d, *J* = 10.3 Hz, 1H), 4.92 (d, *J* = 17.1 Hz, 1H), 4.55 (dd, *J* = 42.0, 9.5 Hz 1H).

**$^{13}$C NMR (126 MHz, $CDCl_3$):** δ 166.0 (d, *J* = 28.6 Hz), 137.6, 133.8 (d, *J* = 4.8 Hz), 133.5, 133.0 (d, *J* = 2.0 Hz), 133.0 (d, *J* = 22.5 Hz), 129.6 (d, *J* = 3.1 Hz), 128.9, 128.8 (d, *J* = 2.1 Hz), 128.7, 128.0, 127.9, 127.2, 126.9, 125.2 (d, *J* = 11.9 Hz), 122.4 (d, *J* = 8.7 Hz), 120.0, 99.4 (d, *J* = 201.9 Hz), 57.0 (d, *J* = 18.8 Hz).

**$^{19}$F NMR (376 MHz, $CDCl_3$):** δ -151.49 – -151.69 (m, 2F), -157.18 (t, *J* = 21.8 Hz, 1F), -161.93 – -162.15 (m, 2F), -172.91 (d, *J* = 33.8 Hz, 1F).

**HRMS (EI):** m/z calcd for $[M]^+$ $C_{27}H_{16}F_6O_2$: 486.1054. Found: 486.1053.

**(2*R*,3*S*)-2-Fluoro-2-(naphthalen-2-yl)-3-phenylpent-4-enamide ((*R*,*S*)-4-$NH_2$):**

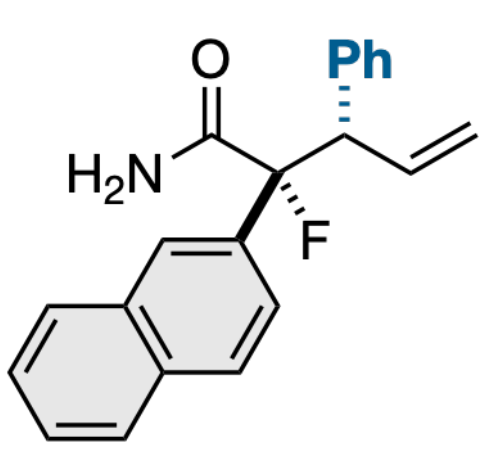


**IR (film):** 3458 (m), 3211 (br), 1671 (s), 1600 (w), 1506 (w), 1453 (w), 1396 (w) $cm^{-1}$.

**$^{1}$H NMR (500 MHz, $CDCl_3$):** δ 8.13 (d, *J* = 1.9 Hz, 1H), 7.97 – 7.80 (m, 4H), 7.55 – 7.45 (m, 4H), 7.39 – 7.32 (m, 2H), 7.32 – 7.27 (m, 1H), 6.10 (s, 1H), 5.98 (ddd, *J* = 17.1, 10.3, 8.3 Hz, 1H), ***5.32 – 5.24 (m)***, 5.05 (d, *J* = 5.8 Hz, 1H), 4.94 (dt, *J* = 10.4, 1.2 Hz, 1H), 4.86 (dt, *J* = 17.2, 1.4 Hz, 1H), 4.52 (dd, *J* = 36.0, 8.5 Hz, 1H).

**$^{13}$C NMR (126 MHz, $CDCl_3$):** δ 172.1 (d, *J* = 23.0 Hz), 138.6, 134.9 (d, *J* = 21.7 Hz), 134.5 (d, *J* = 4.5 Hz), 133.2, 133.1 (d, *J* = 2.2 Hz), 130.2 (d, *J* = 2.3 Hz), 128.7, 128.6, 128.3 (d,

*J* = 2.3 Hz), 127.9, 127.5, 126.7, 126.6, 124.4 (d, *J* = 12.7 Hz), 123.2 (d, *J* = 9.3 Hz), 119.6, 101.4 (d, *J* = 199.0 Hz), 56.6 (d, *J* = 18.8 Hz).

**$^{19}$F NMR (376 MHz, $CDCl_3$):** δ -172.40 (d, *J* = 36.1 Hz, 1F).

**HRMS (ESI):** m/z calcd for $[M+Na]^+$ $C_{21}H_{18}ONFNa$: 342.1265. Found: 342.1265.

HPLC analysis using a chiral column (Cellulose 3 3μ column 22 ˚C, 1.0 ml/min, 95:5 hexane:isopropanol, 210 nm, $t_{major}$ = 34.570 min, $t_{minor}$ = 60.464 min, t = 21.097 min, t = 41.953 min).

(±)-**4:** (*rac*)-**[Ir]** and (*rac*)-Bn-BTM

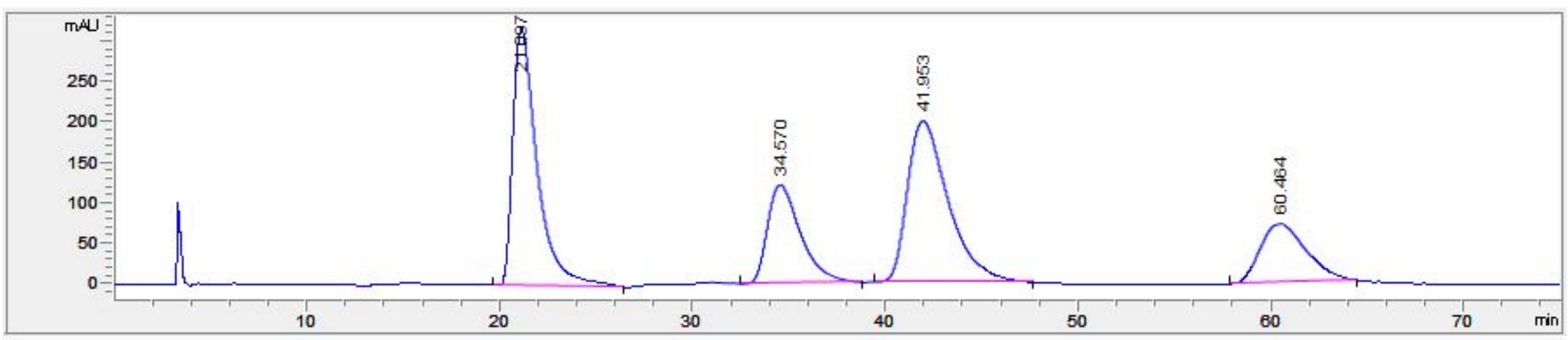


| # | Time | Area | Height | Width | Area% | Symmetry |
|---|---|---|---|---|---|---|
| 1 | 21.097 | 27632.9 | 316.7 | 1.286 | 32.825 | 0.477 |
| 2 | 34.57 | 14657.3 | 122.3 | 1.4787 | 17.411 | 0.65 |
| 3 | 41.953 | 29823.7 | 200.8 | 2.4758 | 35.427 | 0.602 |
| 4 | 60.464 | 12069.7 | 72.9 | 1.9644 | 14.337 | 0.717 |

(*R*,*S*)-**4:** (*S*$_a$,*S*,*S*)-**[Ir]** and (*S*)-Me-BTM

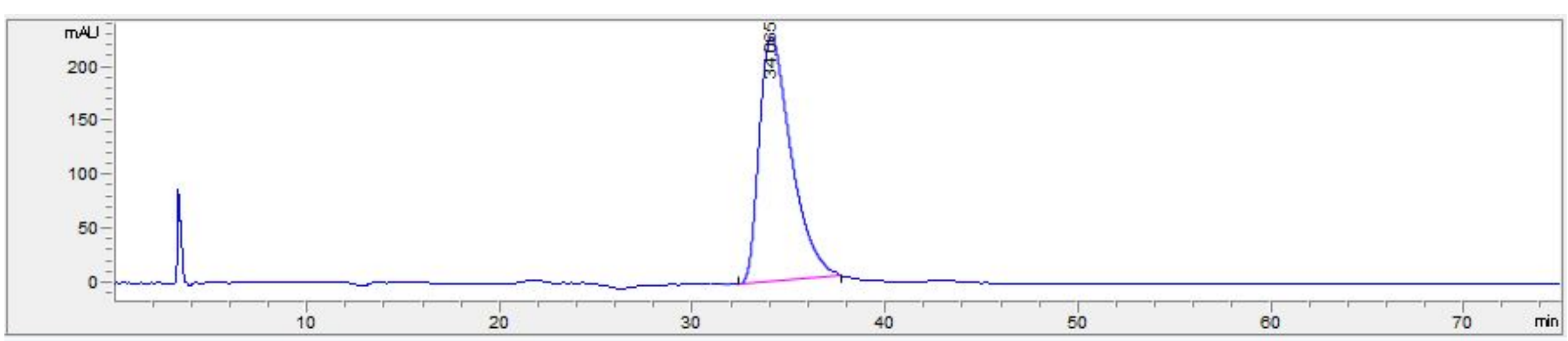


| # | Time | Area | Height | Width | Area% | Symmetry |
|---|---|---|---|---|---|---|
| 1 | 34.065 | 26448.5 | 227.6 | 1.706 | 100.000 | 0.556 |

**(2*R*,3*R*)-2-Fluoro-2-(3-methoxyphenyl)-3-phenylpent-4-enamide ((*R*,*R*)-5):** The *title compound* was obtained (27 mg, 0.090 mmol, 90%) as white solid on treating with $NH_4OH$ after following General Procedure B using pentafluorophenyl ester **1k** (35 mg, 0.1 mmol, 1 equiv.), *t*-butyl

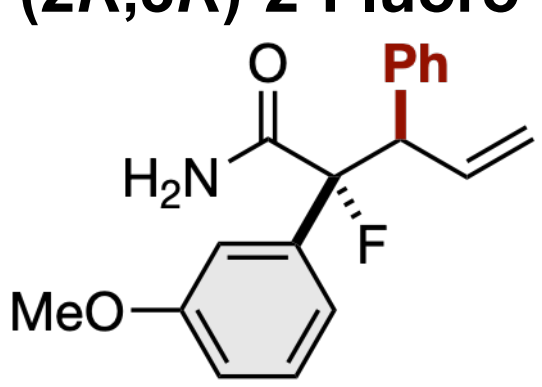

carbonate **2a** (35.1 mg, 0.15 mmol, 1.5 equiv.) and ($R_a$,*R*,*R*)-**[Ir]** catalyst. Purification was done by column chromatography [$SiO_2$, 0.25% Ethyl Acetate in Petroleum Ether]. The enantiomeric ratio (>99:1) was determined by chiral HPLC in comparison with the racemate. Diastereomeric ratio (96:4) was determined by $^{19}$F-NMR analysis of the crude reaction mixture.

**IR (film):** 3450 (m), 3311 br), 3251 br), 2836 (w), 1636 (s), 1602 (m), 1493 (w), 1456 (w), 1386 (w), 1263 (m) cm$^{-1}$.

**$^{1}$H NMR (500 MHz, $CDCl_3$):** δ 7.17 – 7.05 (m, 6H), 7.01 (dt, *J* = 7.8, 1.3 Hz, 1H), 6.93 (t, *J* = 2.2 Hz, 1H), 6.69 (ddd, *J* = 8.1, 2.6, 1.0 Hz, 1H), 6.42 (s, 1H), 6.28 (ddd, *J* = 16.9, 10.5, 8.4 Hz, 1H), 5.57 (d, *J* = 6.2 Hz, 1H), 5.32 – 5.20 (m, 2H), 4.36 (dd, *J* = 34.5, 8.0 Hz 1H), 3.68 (s, 3H).

**$^{13}$C NMR (126 MHz, $CDCl_3$):** δ 172.4 (d, *J* = 22.9 Hz), 159.3 (d, *J* = 2.3 Hz), 138.4 (d, *J* = 22.1 Hz), 137.7, 135.2 (d, *J* = 5.4 Hz), 129.6 (d, *J* = 2.3 Hz), 129.0 (d, *J* = 1.9 Hz), 128.1, 127.0, 119.2, 117.3 (d, *J* = 10.2 Hz), 113.9, 110.6 (d, *J* = 11.3 Hz), 101.4 (d, *J* = 197.9 Hz), 57.0 (d, *J* = 19.4 Hz), 55.4.

**$^{19}$F NMR (376 MHz, $CDCl_3$):** δ -170.55 (d, *J* = 34.8 Hz, 1F).

**HRMS (APCI):** m/z calcd for $[M+H]^+$ $C_{18}H_{19}O_2NF$: 300.1394. Found: 300.1396.

HPLC analysis using a chiral column (Chiralpak IB 3μ column 22 ˚C, 1.0 ml/min, 95:5 hexane:isopropanol, 210 nm, $t_{major}$ = 13.352 min, $t_{minor}$ = 24.663 min, t = 17.729 min, t = 27.706 min).

(±)-**5:** (*rac*)-**[Ir]** and (*rac*)-Bn-BTM

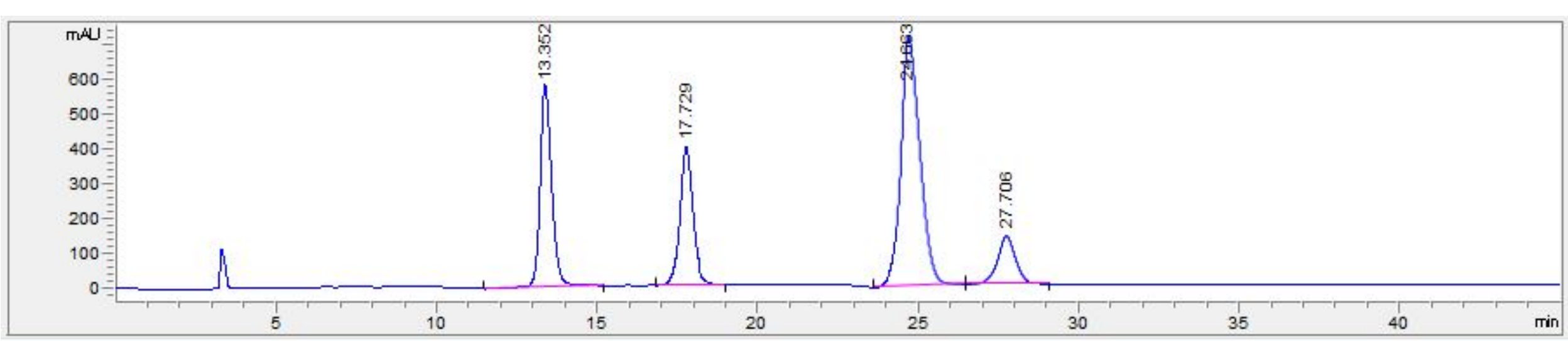


| # | Time | Area | Height | Width | Area% | Symmetry |
|---|---|---|---|---|---|---|
| 1 | 13.352 | 14475.2 | 582.2 | 0.3741 | 23.793 | 0.714 |
| 2 | 17.729 | 11294.1 | 396.2 | 0.4246 | 18.564 | 0.838 |
| 3 | 24.663 | 29516.6 | 715.9 | 0.629 | 48.516 | 0.681 |
| 4 | 27.706 | 5552.5 | 139.8 | 0.5853 | 9.127 | 0.95 |

($R$,$R$)-**5:** ($R_a$,$R$,$R$)-**[Ir]** and ($S$)-Me-BTM

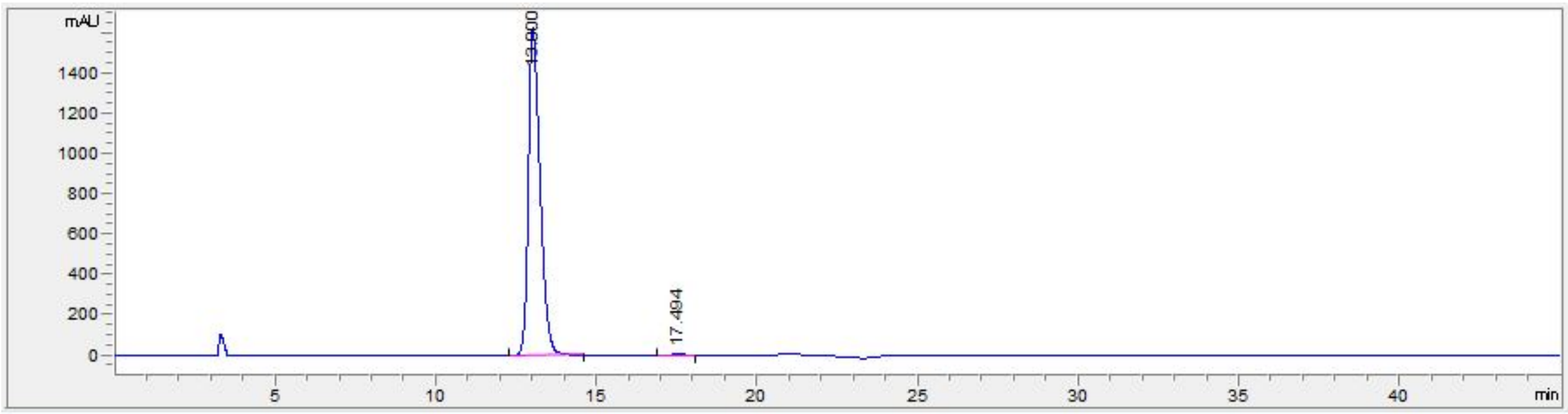


| # | Time | Area | Height | Width | Area% | Symmetry |
|---|---|---|---|---|---|---|
| 1 | 13 | 40383.9 | 1622.5 | 0.3784 | 99.410 | 0.554 |
| 2 | 17.494 | 239.5 | 8.5 | 0.4177 | 0.590 | 0.934 |

**(2*R*,3*S*)-2-Fluoro-2-(3-methoxyphenyl)-3-phenylpent-4-enamide ((*R*,*S*)-5-$NH_2$):** The

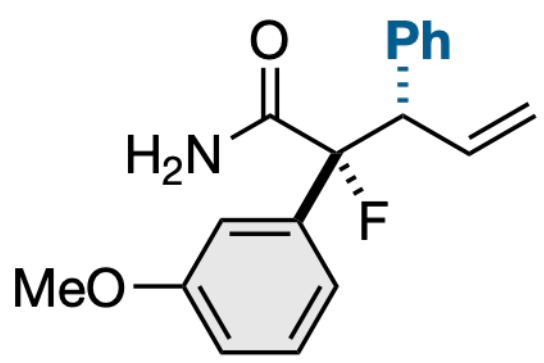


*title compound* was obtained (24 mg, 0.080 mmol, 80%) as white solid on treating with $NH_4OH$ after following General Procedure B using pentafluorophenyl ester **1k** (35 mg, 0.1 mmol, 1 equiv.), *t*-butyl carbonate **2a** (35.1 mg, 0.15 mmol, 1.5 equiv.) and ($S_a$,$S$,$S$)-**[Ir]** catalyst. Purification was done by column chromatography [$SiO_2$, 0.25% Ethyl Acetate in Petroleum Ether]. The enantiomeric ratio (>99:1) was determined by chiral HPLC in comparison with the racemate. Diastereomeric ratio (93:7) was determined by $^{19}$F-NMR analysis of the crude reaction mixture.

**IR (film):** 3455 (w), 3199 (br), 2835 (w), 1649 (s), 1601 (m), 1490 (m), 1453 (w), 1428 (w), 1264 (m) $cm^{-1}$.

**$^1$H NMR (500 MHz, $CDCl_3$):** δ 7.44 – 7.40 (m, 2H), 7.32 (td, *J* = 7.4, 4.7 Hz, 3H), 7.29 – 7.25 (m, 2H), 7.22 (t, *J* = 2.1 Hz, 1H), 6.88 (ddd, *J* = 8.1, 2.6, 1.1 Hz, 1H), 6.04 (s, 1H), 5.96 (ddd, *J* = 17.2, 10.3, 8.2 Hz, 1H), 5.09 (s, 1H), 5.00 (dt, *J* = 10.3, 1.3 Hz, 1H), 4.88 (dt, *J* = 17.2, 1.4 Hz, 1H), 4.37 (dd, *J* = 36.5, 8.5 Hz, 1H), 3.84 (s, 3H).

**$^{13}$C NMR (126 MHz, $CDCl_3$):** δ 171.9 (d, *J* = 23.1 Hz), 159.7 (d, *J* = 2.6 Hz), 138.8 (d, *J* = 22.0 Hz), 138.4, 134.5 (d, *J* = 4.5 Hz), 130.0 (d, *J* = 2.3 Hz), 129.4 (d, *J* = 2.1 Hz), 128.4, 127.4, 119.3, 117.6 (d, *J* = 10.9 Hz), 113.8, 111.0 (d, *J* = 12.0 Hz), 101.0 (d, *J* = 199.1 Hz), 56.5 (d, *J* = 18.9 Hz), 55.5.

**$^{19}$F NMR (376 MHz, $CDCl_3$):** δ -172.13 (d, *J* = 36.1 Hz, 1F).

**HRMS (APCI):** m/z calcd for $[M+H]^+$ $C_{18}H_{19}O_2NF$: 300.1394. Found: 300.1395.

HPLC analysis using a chiral column (Chiralpak IB 3µ column 22 ˚C, 1.0 ml/min, 95:5 hexane:isopropanol, 210 nm, $t_{major}$ = 17.729 min, $t_{minor}$ = 27.706 min, t = 13.352 min, t = 24.663 min).

**(±)5:** (*rac*)-**[Ir]** and (*rac*)-Bn-BTM

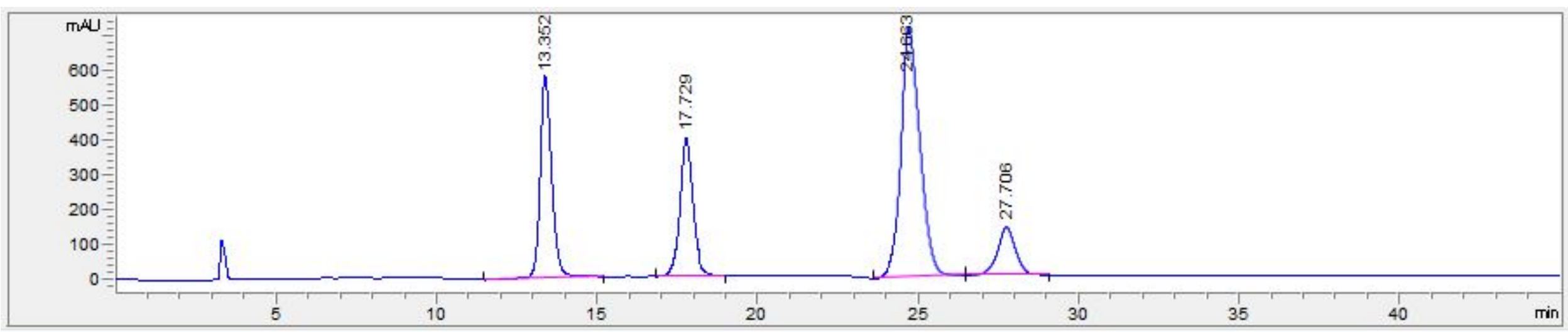


| # | Time | Area | Height | Width | Area% | Symmetry |
|---|---|---|---|---|---|---|
| 1 | 13.352 | 14475.2 | 582.2 | 0.3741 | 23.793 | 0.714 |
| 2 | 17.729 | 11294.1 | 396.2 | 0.4246 | 18.564 | 0.838 |
| 3 | 24.663 | 29516.6 | 715.9 | 0.629 | 48.516 | 0.681 |
| 4 | 27.706 | 5552.5 | 139.8 | 0.5853 | 9.127 | 0.95 |

(*R*,*S*)-**5:** (*S*$_a$,*S*,*S*)-**[Ir]** and (*S*)-Me-BTM

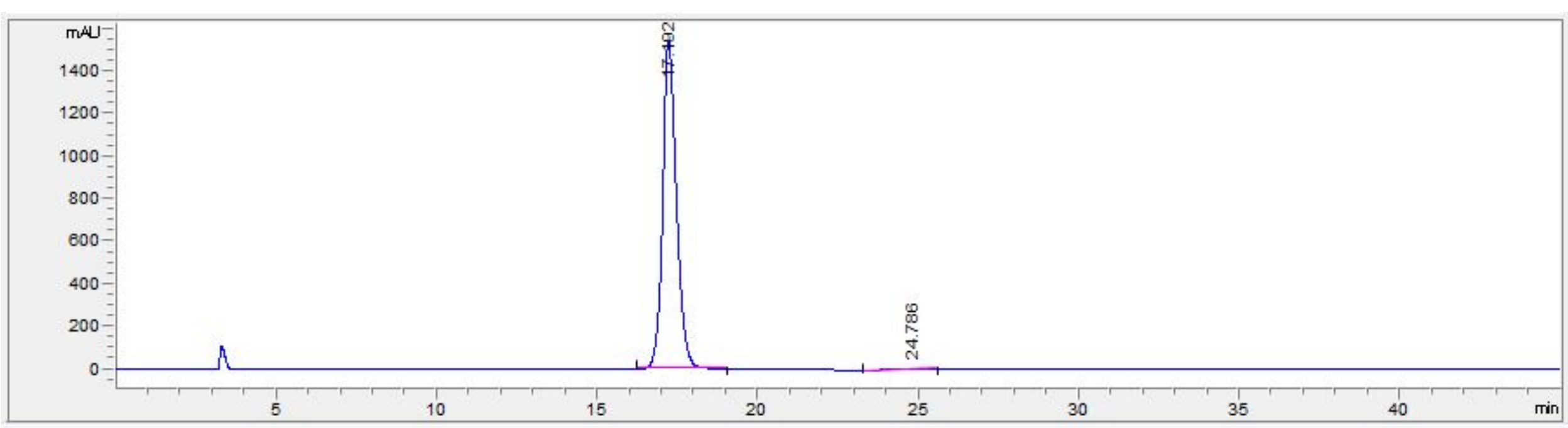


| # | Time | Area | Height | Width | Area% | Symmetry |
|---|---|---|---|---|---|---|
| 1 | 17.192 | 45177 | 1546.9 | 0.4367 | 98.634 | 0.703 |
| 2 | 24.786 | 625.7 | 5.7 | 1.2961 | 1.366 | 3.4 |

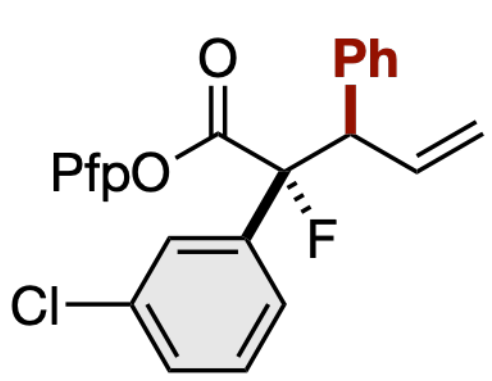


**Perfluorophenyl (2*R*,3*R*)-2-(3-chlorophenyl)-2-fluoro-3-phenylpent-4-enoate ((*R*,*R*)-6):** Prepared according to General Procedure B using pentafluorophenyl ester **1l** (35.4 mg, 0.1 mmol, 1 equiv.), *t*-butyl carbonate **2a** (35.1 mg, 0.15 mmol, 1.5 equiv.), (*R*$_a$,*R*,*R*)-**[Ir]** (4.2 mg, 4 mol%), (*S*)-Me-BTM (3.8 mg, 20 mol%) and DIPEA (22.6 µL, 0.13 mmol, 1.3 equiv.) in 1,4-dioxane (0.3 ml, 0.3 M). The *title compound* was obtained (35.2

mg, 0.075 mmol, 75%) as a colorless oil following purification by column chromatography [$SiO_2$, 0.25% Ethyl Acetate in Petroleum Ether]. The enantiomeric ratio (>99:1 *er*) of the corresponding primary amide was determined by chiral HPLC in comparison with the racemate. Diastereomeric ratio (95:5) was determined by $^{19}$F-NMR analysis of the crude reaction mixture.

**IR (film):** 3069 (w), 3032 (w), 1782 (m), 1640 (m), 1597 (w), 1519 (s), 1477 (w), 1203 (m), 1182 (m), 1010 (s), 997 (s), 701 (m) cm$^{-1}$.

**$^1$H NMR (500 MHz, $CDCl_3$):** δ 7.43 (d, *J* = 2.0 Hz, 1H), 7.31 (dt, *J* = 7.5, 1.7 Hz, 1H), 7.24 – 7.20 (m, 1H), 7.20 – 7.14 (m, 4H), 7.13 – 7.10 (m, 2H), 6.37 (ddd, *J* = 17.1, 10.2, 8.8 Hz, 1H), 5.57 – 5.24 (m, 2H), 4.35 (dd, *J* = 42.0, 9.5 Hz, *J* = 33.0, 9.0 Hz 1H).

**$^{13}$C NMR (126 MHz, $CDCl_3$):** δ 166.1 (d, *J* = 28.0 Hz), 137.1 (d, *J* = 22.9 Hz), 136.1, 134.7 (d, *J* = 2.4 Hz), 134.1 (d, *J* = 5.1 Hz), 129.8 (d, *J* = 1.9 Hz), 129.5 (d, *J* = 2.3 Hz), 129.2, 128.5, 127.6, 125.4 (d, *J* = 10.6 Hz), 123.3 (d, *J* = 9.8 Hz), 120.2, 99.3 (d, *J* = 203.0 Hz), 57.6 (d, *J* = 18.9 Hz).

**$^{19}$F NMR (376 MHz, $CDCl_3$):** δ -151.33 – -151.54 (m, 2F), -156.62 (t, *J* = 21.7 Hz, 1F), -161.51 – -161.72 (m, 2F), -174.27 (d, *J* = 33.1 Hz, 1F).

**HRMS (EI):** m/z calcd for $[M]^+$ $C_{23}H_{13}ClF_6O_2$: 470.0508. Found: 470.0508.

**(2*R*,3*R*)-2-(3-Chlorophenyl)-2-fluoro-3-phenylpent-4-enamide ((*R*,*R*)-6-NH$_2$):**

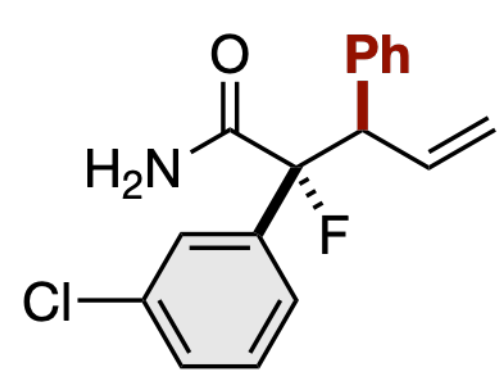


**IR (film):** 3446 (m), 3196 (br), 1637 (s), 1595 (w), 1476 (w), 1455 (w), 1390 (w), 699 (m) cm$^{-1}$.

**$^1$H NMR (500 MHz, $CDCl_3$):** δ 7.42 (t, *J* = 1.9 Hz, 1H), 7.30 (dt, *J* = 7.3, 1.7 Hz, 1H), 7.18 – 7.10 (m, 4H), 7.09 – 7.05 (m, 2H), 6.42 (s, 1H), 6.34 – 6.19 (m, 1H), 5.54 (s, 1H), 5.36 – 5.20 (m, 2H), 4.33 (dd, *J* = 35.0, 8.5 Hz, 1H).

**$^{13}C$ NMR (126 MHz, $CDCl_3$):** δ 171.9 (d, *J* = 22.8 Hz), 138.8 (d, *J* = 22.3 Hz), 137.2, 134.8 (d, *J* = 5.4 Hz), 134.1 (d, *J* = 2.5 Hz), 129.6 (d, *J* = 2.1 Hz), 129.3 (d, *J* = 1.9 Hz), 128.3, 128.3, 127.2, 125.1 (d, *J* = 11.5 Hz), 123.2 (d, *J* = 10.1 Hz), 119.6, 101.0 (d, *J* = 198.7 Hz), 57.2 (d, *J* = 19.2 Hz).

**$^{19}F$ NMR (376 MHz, $CDCl_3$):** δ -171.42 (d, *J* = 34.8 Hz, 1F).

**HRMS (ESI):** m/z calcd for $[M+Na]^+$ $C_{17}H_{15}ONClFNa$: 326.0718. Found: 326.0718.

HPLC analysis using a chiral column (Chiralpak IB 3μ column 22 ˚C, 0.5 ml/min, 95:5 hexane:isopropanol, 210 nm, $t_{major}$ = 20.734 min, $t_{minor}$ = 28.485 min, t = 26.885 min, t = 36.275 min).

(±)-**6:** (*rac*)-**[Ir]** and (*rac*)-Bn-BTM

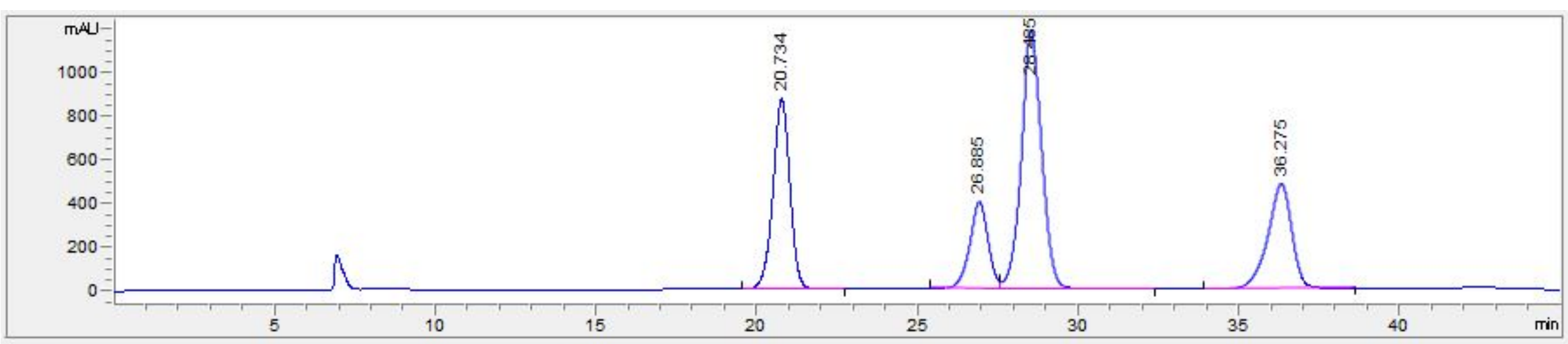


| # | Time | Area | Height | Width | Area% | Symmetry |
|---|---|---|---|---|---|---|
| 1 | 20.734 | 31240 | 870.4 | 0.5467 | 25.288 | 1.017 |
| 2 | 26.885 | 15712.6 | 396.2 | 0.5946 | 12.719 | 1.072 |
| 3 | 28.485 | 51688.6 | 1173.4 | 0.6579 | 41.841 | 0.86 |
| 4 | 36.275 | 24894.7 | 477.4 | 0.7803 | 20.152 | 1.266 |

(*R*,*R*)-**6:** (*R*a,*R*,*R*)-**[Ir]** and (*S*)-Me-BTM

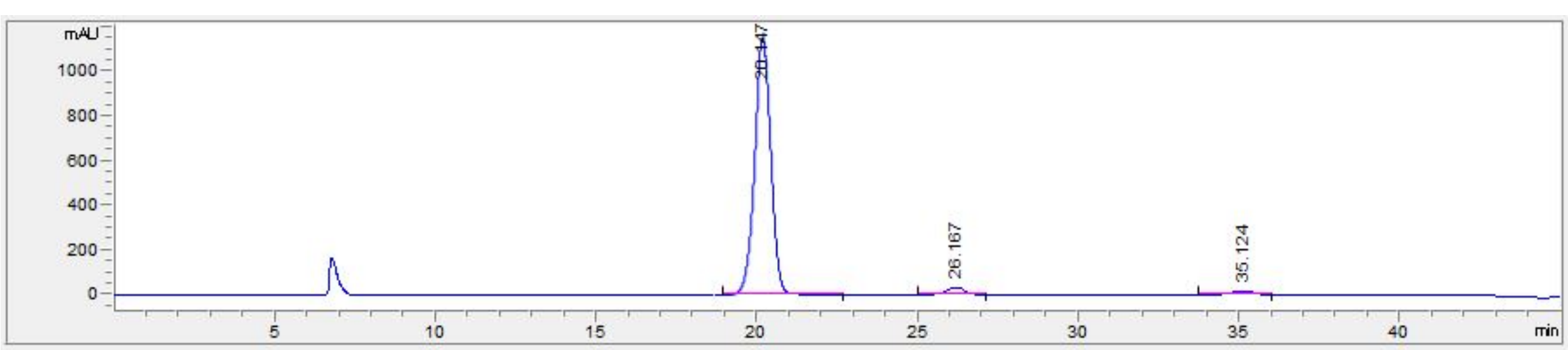


| # | Time | Area | Height | Width | Area% | Symmetry |
|---|---|---|---|---|---|---|
| 1 | 20.147 | 40213.7 | 1151.9 | 0.5353 | 94.926 | 0.955 |
| 2 | 26.167 | 1321.6 | 33 | 0.605 | 3.120 | 1.182 |
| 3 | 35.124 | 827.7 | 17.4 | 0.6825 | 1.954 | 1.335 |

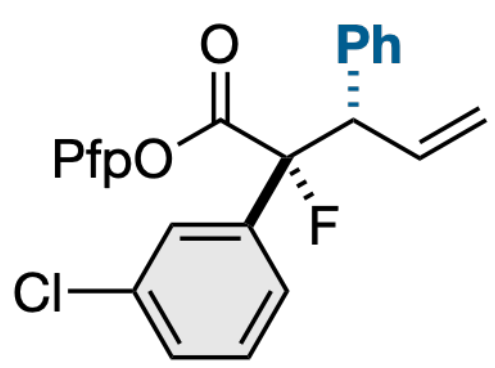


**Perfluorophenyl (2*R*,3*S*)-2-(3-chlorophenyl)-2-fluoro-3-phenylpent-4-enoate ((*R,S*)-6):** Prepared according to General Procedure B using pentafluorophenyl ester **1l** (35.4 mg, 0.1 mmol, 1 equiv.), *t*-butyl carbonate **2a** (35.1 mg, 0.15 mmol, 1.5 equiv.) and ($S_a$*,S,S*)-**[Ir]** (4.2 mg, 4 mol%), (*S*)-Me-BTM (3.8 mg, 20 mol%) and DIPEA (22.6 μL, 0.13 mmol, 1.3 equiv.) in 1,4-dioxane (0.3 ml, 0.3 M). The *title compound* was obtained (33.5 mg, 0.071 mmol, 71%) as a white solid following purification by column chromatography [$SiO_2$, 0.25% Ethyl Acetate in Petroleum Ether]. The enantiomeric ratio (>99:1 *er*) of the corresponding primary amide was determined by chiral HPLC in comparison with the racemate. Diastereomeric ratio (88:12) was determined by $^{19}$F-NMR analysis of the crude reaction mixture.

**IR (film):** 3070 (w), 3032 (w), 1800 (w), 1782 (m), 1640 (w), 1576 (w), 1517 (s), 1476 (w), 1198 (m), 1008 (s), 996 (s), 700 (m) cm$^{-1}$.

**$^1$H NMR (500 MHz, CDCl$_3$):** δ 7.73 – 7.69 (m, 1H), 7.59 (dq, *J* = 5.3, 1.8 Hz, 1H), 7.50 – 7.45 (m, 2H), 7.44 – 7.30 (m, 5H), ****6.37 (ddd, J = 17.1, 10.3, 8.8 Hz)***, 5.96 (ddd, *J* = 17.2, 10.3, 8.1 Hz, 1H), ****5.46 – 5.32 (m)***, 5.09 (dd, *J* = 10.3, 1.1 Hz, 1H), 4.93 (d, *J* = 17.1 Hz, 1H), 4.35 (dd, *J* = 33.5, 8.0 Hz, 1H).

**$^{13}$C NMR (126 MHz, CDCl$_3$):** δ 165.6 (d, *J* = 28.2 Hz), 137.7 (d, *J* = 23.1 Hz), 137.1, 135.1 (d, *J* = 2.6 Hz), 133.4 (d, *J* = 5.0 Hz), 130.2 (d, *J* = 2.0 Hz), 129.6, 129.5 (d, *J* = 3.0 Hz), 129.0, 128.1, 125.7 (d, *J* = 11.4 Hz), 123.6 (d, *J* = 10.0 Hz), 120.3, 98.7 (d, *J* = 203.0 Hz), 57.3 (d, *J* = 18.8 Hz).

**$^{19}$F NMR (376 MHz, CDCl$_3$):** δ -151.60 – -151.82 (m, 2F), -156.89 (t, *J* = 21.8 Hz, 1F), -161.85 (dd, *J* = 21.7, 17.5 Hz, 2F), -173.32 (d, *J* = 33.7 Hz, 1F).

**HRMS (EI):** m/z calcd for $[M]^+$ $C_{23}H_{13}ClF_6O_2$: 470.0508. Found: 470.0505.

**(2*R*,3*S*)-2-(3-Chlorophenyl)-2-fluoro-3-phenylpent-4-enamide ((*R,S*)-6-NH$_2$):**

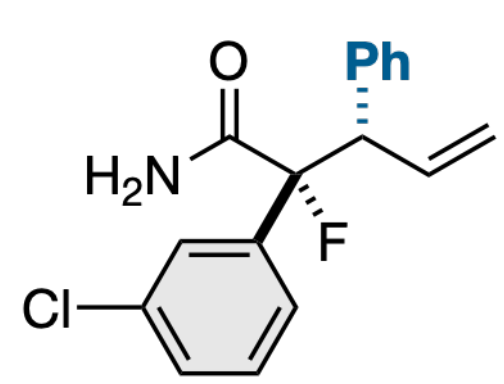

**IR (film):** 3447 (m), 3174 (br), 1671 (s), 1595 (w), 1478 (w), 1453 (w), 1394 (w), 695 (m) $cm^{-1}$.

**$^{1}$H NMR (500 MHz, $CDCl_3$):** δ 7.70 – 7.65 (m, 1H), 7.62 – 7.54 (m, 1H), 7.41 (dt, *J* = 8.0, 1.6 Hz, 2H), 7.37 – 7.31 (m, 4H), 7.31 – 7.27 (m, 1H), ***7.18 – 7.03 (m, 0.9H)**, ***6.41 (s, 0.1H)**, ***6.32 – 6.21 (m, 0.1H)**, 6.03 (s, 1H), 5.94 (ddd, *J* = 17.1, 10.3, 8.5 Hz, 1H), ***5.50 (s, 0.1H)**, ***5.31 – 5.19 (m, 0.3H),** 5.09 (s, 1H), 5.02 (dt, *J* = 10.3, 1.1 Hz, 1H), 4.88 (dd, *J* = 17.1, 1.4 Hz, 1H), 4.32 (dd, *J* = 36.0, 8.5 Hz, 1H).

**$^{13}$C NMR (126 MHz, $CDCl_3$):** δ 171.4 (d, *J* = 23.0 Hz), 139.3 (d, *J* = 22.3 Hz), 138.0, 134.5 (d, *J* = 2.7 Hz), 134.0 (d, *J* = 4.6 Hz), 129.9 (d, *J* = 2.3 Hz), 129.7 (d, *J* = 2.2 Hz), 128.6, 128.5, 127.5, 125.4 (d, *J* = 12.5 Hz), 123.6 (d, *J* = 10.3 Hz), 119.8, 100.5 (d, *J* = 200.2 Hz), 56.8 (d, *J* = 18.8 Hz).

**$^{19}$F NMR (376 MHz, $CDCl_3$):** δ -172.91 (d, *J* = 36.1 Hz, 1F).

**HRMS (ESI):** m/z calcd for $[M+Na]^+$ $C_{17}H_{15}ONClFNa$: 326.0718. Found: 326.0720.

HPLC analysis using a chiral column (Chiralpak IB 3μ column 22 ˚C, 0.5 ml/min, 95:5 hexane:isopropanol, 210 nm, $t_{major}$ = 36.275 min, $t_{minor}$ = 26.885 min, t = 20.734 min, t = 28.485 min).

(+)-**6:** (*rac*)-**[Ir]** and (*rac*)-Bn-BTM

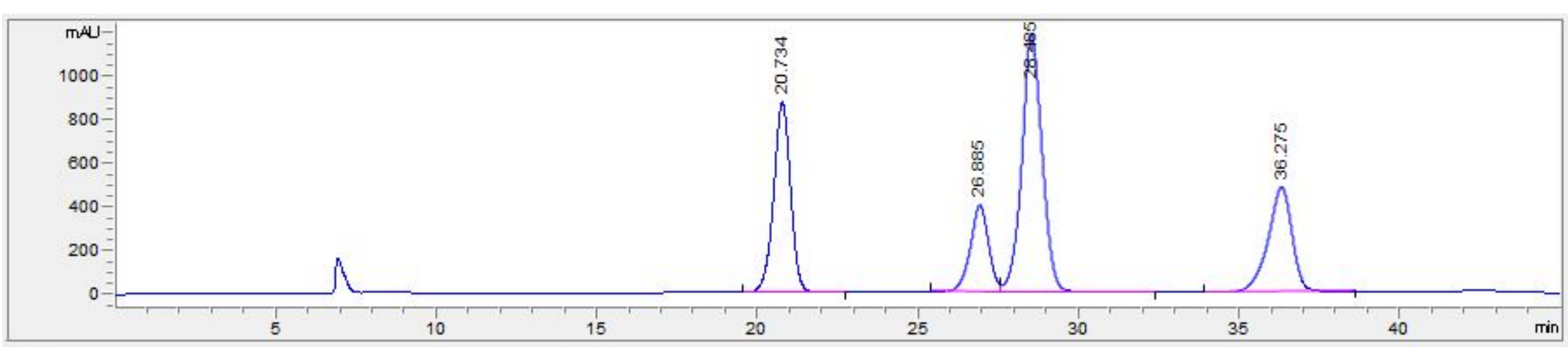


| # | Time | Area | Height | Width | Area% | Symmetry |
|---|---|---|---|---|---|---|
| 1 | 20.734 | 31240 | 870.4 | 0.5467 | 25.288 | 1.017 |
| 2 | 26.885 | 15712.6 | 396.2 | 0.5946 | 12.719 | 1.072 |
| 3 | 28.485 | 51688.6 | 1173.4 | 0.6579 | 41.841 | 0.86 |
| 4 | 36.275 | 24894.7 | 477.4 | 0.7803 | 20.152 | 1.266 |

(*R*,*S*)-**6:** ($S_a$,*S*,*S*)-**[Ir]** and (*S*)-Me-BTM

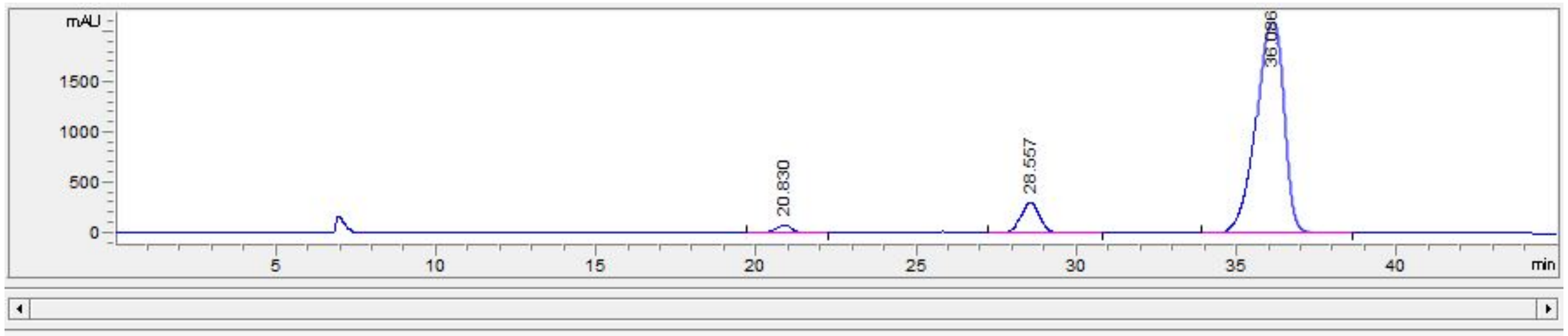


| # | Time | Area | Height | Width | Area% | Symmetry |
|---|---|---|---|---|---|---|
| 1 | 20.83 | 2758.6 | 77.5 | 0.5415 | 1.964 | 1.18 |
| 2 | 28.557 | 12586.5 | 302.5 | 0.6255 | 8.959 | 1.075 |
| 3 | 36.086 | 125144.9 | 2092.3 | 0.9298 | 89.077 | 1.395 |

## ELECTROPHILE SCOPE

**Perfluorophenyl (2*R*,3*R*)-2-fluoro-2-phenyl-3-(p-tolyl)pent-4-enoate ((*R*,*R*)-16):**

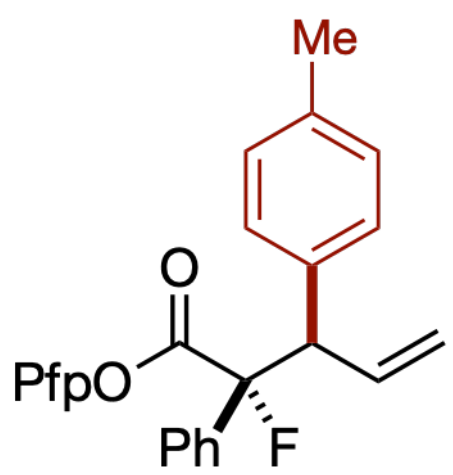


Prepared according to General Procedure B using pentafluorophenyl ester **1a** (32 mg, 0.1 mmol, 1 equiv.), *t*-butyl carbonate **2b** (37.2 mg, 0.15 mmol, 1.5 equiv.), ($R_a$,*R*,*R*)-**[Ir]** (4.2 mg, 4 mol%), (*S*)-Me-BTM (3.8 mg, 20 mol%) and DIPEA (22.6 µL, 0.13 mmol, 1.3 equiv.) in 1,4-dioxane (0.3 ml, 0.3 M). The *title compound* was obtained (38 mg, 0.084 mmol, 84%) as a colorless oil following purification by column chromatography [$SiO_2$, 0.25% Ethyl Acetate in Petroleum Ether]. The enantiomeric ratio (>99:1 *er*) of the corresponding primary amide was determined by chiral HPLC in comparison with the racemate. Diastereomeric ratio (97:3) was determined by $^{19}$F-NMR analysis of the crude reaction mixture.

**IR (film):** 3030 (w), 2926 (w), 1783 (m), 1639 (w), 1519 (s), 1472 (w), 1202 (m), 1180 (m), 1010 (s), 998 (s), 746 (m) cm$^{-1}$.

**$^1$H NMR (500 MHz, CDCl$_3$):** δ 7.47 – 7.41 (m, 2H), 7.30 – 7.23 (m, 3H), 7.04 – 7.00 (m, 2H), 6.95 (d, *J* = 7.9 Hz, 2H), 6.37 (ddd, *J* = 17.1, 10.3, 8.8 Hz, 1H), 5.38 (d, *J* = 17.1 Hz, 1H), 5.33 (dd, *J* = 10.3, 1.2 Hz, 1H), 4.38 (dd, *J* = 33.0, 8.5 Hz, 1H), 2.22 (s, 1H).

**$^{13}$C NMR (126 MHz, CDCl$_3$):** δ 166.6 (d, *J* = 28.4 Hz), 137.0, 135.2 (d, *J* = 22.5 Hz), 134.8 (d, *J* = 5.2 Hz), 133.5, 129.4 (d, *J* = 2.4 Hz), 129.1, 128.9, 128.4 (d, *J* = 2.1 Hz), 125.1 (d, *J* = 9.9 Hz), 119.6, 99.8 (d, *J* = 201.2 Hz), 57.1 (d, *J* = 19.2 Hz), 21.1.

**$^{19}$F NMR (376 MHz, CDCl$_3$):** δ -151.36 – -151.53 (m, 2F), -157.03 (t, *J* = 21.6 Hz, 1F), -161.79 – -162.01 (m, 2F), -174.46 (d, *J* = 33.2 Hz, 1F).

**HRMS (EI):** m/z calcd for $[M]^+$ $C_{24}H_{16}F_6O_2$: 450.1054. Found: 450.1056.

**(2*R*,3*R*)-2-Fluoro-2-phenyl-3-(p-tolyl)pent-4-enamide ((*R*,*R*)-16-$NH_2$):**

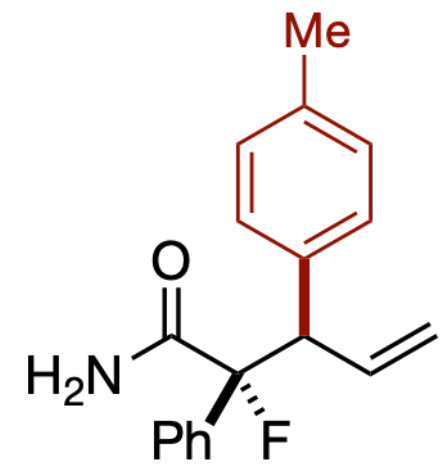

**IR (film):** 3432 (m), 3178 (br), 1670 (s), 1607 (w), 1515 (w), 1448 (m), 1394 (m) $cm^{-1}$.

**$^1$H NMR (500 MHz, $CDCl_3$):** δ 7.48 – 7.38 (m, 2H), 7.23 – 7.13 (m, 3H), 6.97 (dd, *J* = 8.1, 1.4 Hz, 2H), 6.92 (d, *J* = 8.0 Hz, 2H), 6.42 (s, 1H), 6.27 (ddd, *J* = 17.1, 10.3, 8.4 Hz, 1H), 5.50 (s, 1H), 5.32 – 5.13 (m, 2H), 4.35 (dd, *J* = 34.5, 8.5 Hz, 1H), 2.20 (s, 3H).

**$^{13}$C NMR (126 MHz, $CDCl_3$):** δ 172.6 (d, *J* = 23.0 Hz), 137.0 (d, *J* = 22.0 Hz), 136.4, 135.4 (d, *J* = 5.4 Hz), 134.6, 129.5 (d, *J* = 2.2 Hz), 128.9, 128.1, 128.0 (d, *J* = 2.1 Hz), 124.9 (d, *J* = 10.5 Hz), 119.0, 101.6 (d, *J* = 196.9 Hz), 56.7 (d, *J* = 19.5 Hz), 21.1.

**$^{19}$F NMR (376 MHz, $CDCl_3$):** δ -171.33 (d, *J* = 35.0 Hz, 1F).

**HRMS (ESI):** m/z calcd for $[M+Na]^+$ $C_{18}H_{18}ONFNa$: 306.1265. Found: 306.1266.

HPLC analysis using a chiral column (Chiralpak IB 3μ column 22 ˚C, 0.5 ml/min, 95:5 hexane:isopropanol, 210 nm, $t_{major}$ = 21.859 min, $t_{minor}$ = 52.040 min, t = 20.524 min, t = 34.220 min).

(±)-**16:** (*rac*)-**[Ir]** and (*rac*)-Bn-BTM

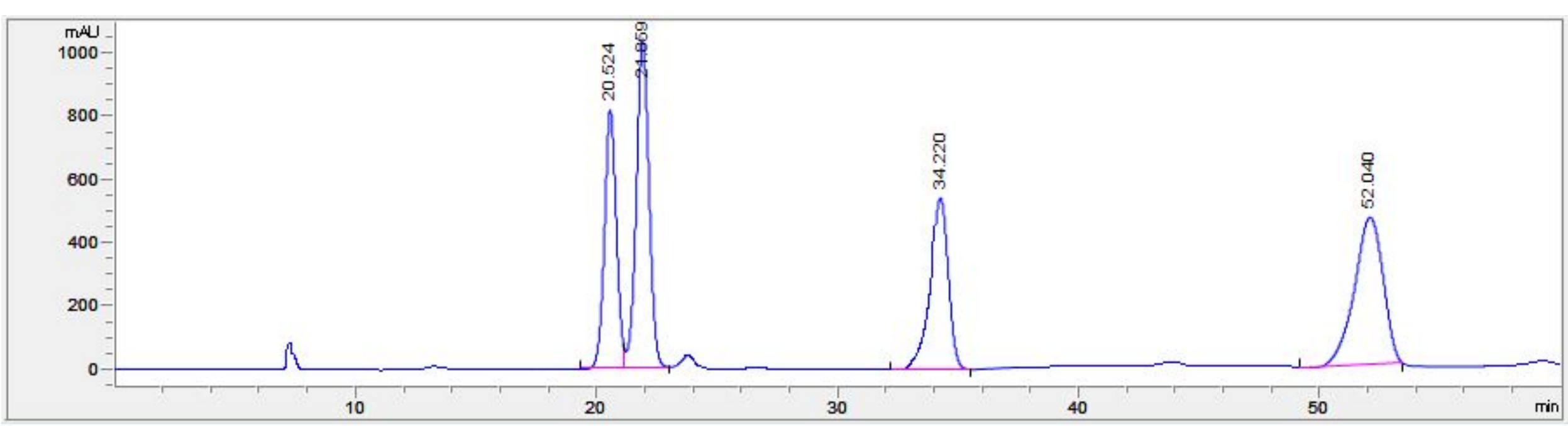

| # | Time | Area | Height | Width | Area% | Symmetry |
|---|---|---|---|---|---|---|
| 1 | 20.524 | 28220.3 | 819.8 | 0.5256 | 21.085 | 0.918 |
| 2 | 21.859 | 38413.1 | 1042.3 | 0.562 | 28.701 | 0.911 |
| 3 | 34.22 | 28062 | 542.1 | 0.7859 | 20.967 | 1.289 |
| 4 | 52.04 | 39144.9 | 466.4 | 1.3988 | 29.248 | 1.218 |

(*R*,*R*)-**16:** (*R*$_a$,*R*,*R*)-**[Ir]** and (*S*)-Me-BTM

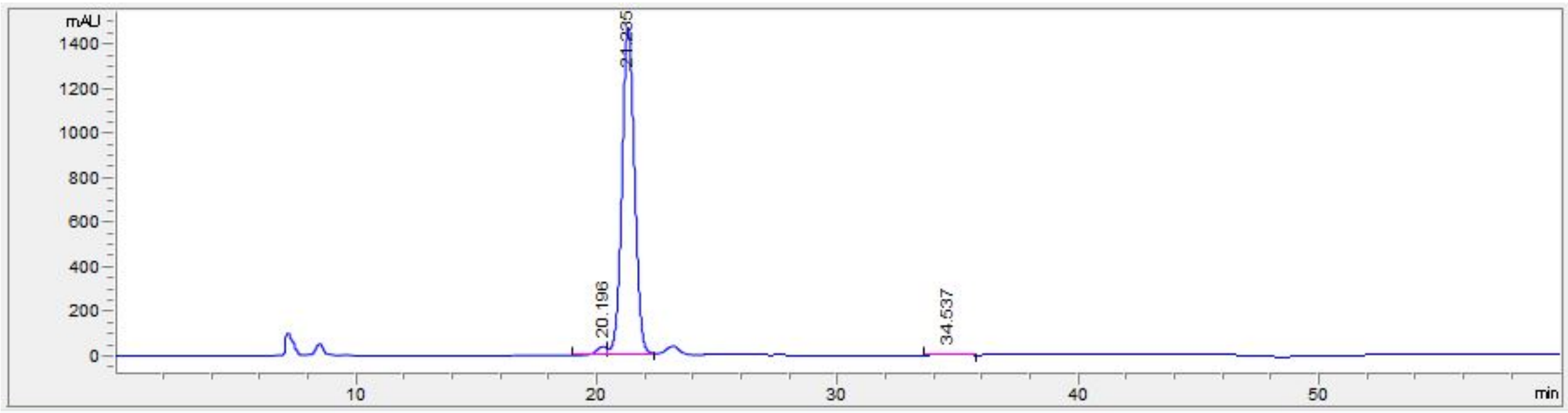


| # | Time | Area | Height | Width | Area% | Symmetry |
|---|---|---|---|---|---|---|
| 1 | 20.196 | 1270.3 | 39.1 | 0.4747 | 2.305 | 1.591 |
| 2 | 21.235 | 53470.2 | 1466.4 | 0.5574 | 97.006 | 0.872 |
| 3 | 34.537 | 380 | 7.6 | 0.5951 | 0.689 | 1.066 |

**Perfluorophenyl (2*R*,3*S*)-2-fluoro-2-phenyl-3-(p-tolyl)pent-4-enoate ((*R*,*S*)-16):**

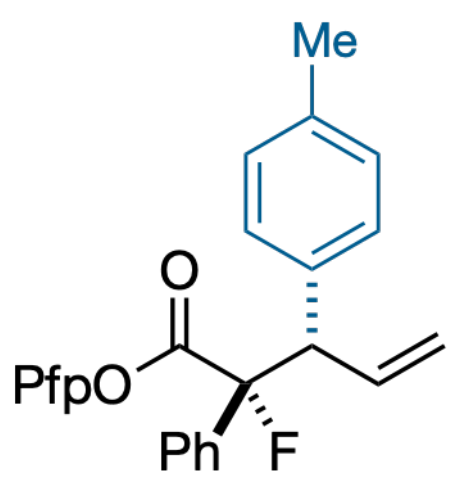


Prepared according to General Procedure B using pentafluorophenyl ester **1a** (32 mg, 0.1 mmol, 1 equiv.), *t*-butyl carbonate **2b** (37.2 mg, 0.15 mmol, 1.5 equiv.) and (*S*$_a$,*S*,*S*)-**[Ir]** catalyst. The diastereomeric ratio was determined to be 1:22. The *title compound* was obtained (37.8 mg, 0.084 mmol, 84%) as a white solid following purification by column chromatography [$SiO_2$, 0.25% Ethyl Acetate in Petroleum Ether]. The enantiomeric ratio (>99:1 *er*) of the corresponding primary amide was determined by chiral HPLC in comparison with the racemate. Diastereomeric ratio (96:4) was determined by $^{19}$F-NMR analysis of the crude reaction mixture.

**IR (film):** 3030 (w), 2926 (w), 1801 (w), 1781 (m), 1639 (w), 1518 (s), 1471 (w), 1195 (m), 1008 (s), 997 (s), 746 (m) cm$^{-1}$.

**$^1$H NMR (500 MHz, $CDCl_3$):** δ 7.72 – 7.67 (m, 2H), 7.47 (dd, *J* = 8.4, 6.6 Hz, 2H), 7.45 – 7.40 (m, 1H), 7.37 (dd, *J* = 8.1, 1.6 Hz, 2H), 7.18 (d, *J* = 7.8 Hz, 2H), 5.97 (ddd, *J* = 17.1, 10.3, 7.9 Hz, 1H), , 5.05 (dt, *J* = 10.4, 1.2 Hz, 1H), 4.91 (dt, *J* = 17.1, 1.4 Hz, 1H), 4.40 (dd, *J* = 34.0, 8.0 Hz,1H), 2.36 (s, 3H), ****2.22 (s, 0.1H)***.

**$^{13}$C NMR (126 MHz, $CDCl_3$):** δ 166.0 (d, *J* = 28.7 Hz), 137.6, 135.8 (d, *J* = 22.6 Hz), 134.5, 134.1 (d, *J* = 4.8 Hz), 129.5, 129.4 (d, *J* = 3.0 Hz), 129.3, 128.8 (d, *J* = 2.2 Hz), 125.4 (d, *J* = 10.2 Hz), 119.7, 99.3 (d, *J* = 200.9 Hz), 56.6 (d, *J* = 18.9 Hz), 21.2.

**$^{19}$F NMR (376 MHz, CDCl$_3$):** δ -151.52 – -151.70 (m, 2F), -157.33 (t, *J* = 21.8 Hz, 1F), -162.05 – -162.28 (m, 2F), -173.47 (d, *J* = 33.8 Hz, 1F).

**HRMS (EI):** m/z calcd for $[M]^+$ $C_{24}H_{16}F_6O_2$: 450.1054. Found: 450.1053.

**(2*R*,3*S*)-2-Fluoro-2-phenyl-3-(p-tolyl)pent-4-enamide ((*R*,*S*)-16-NH$_2$):**

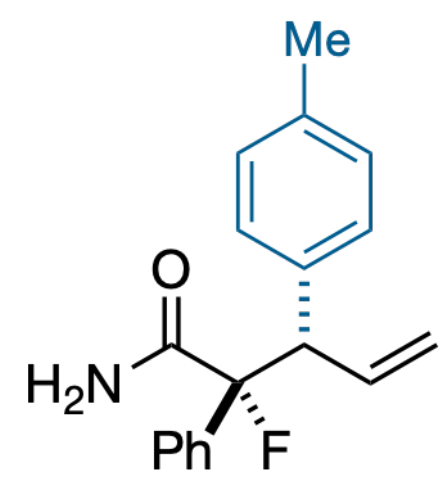


**IR (film):** 3437 (m), 3190 (br), 1667 (s), 1607 (w), 1515 (w), 1447 (w), 1396 (w) cm$^{-1}$.

**$^1$H NMR (500 MHz, CDCl$_3$):** δ 7.72 – 7.63 (m, 2H), 7.43 – 7.37 (m, 2H), 7.37 – 7.29 (m, 3H), 7.14 (d, *J* = 7.8 Hz, 2H), 6.05 (s, 1H), 5.94 (ddd, *J* = 17.1, 10.3, 8.3 Hz, 1H), 5.06 (s, 1H), 4.97 (dt, *J* = 10.4, 1.2 Hz, 1H), 4.85 (dt, *J* = 17.2, 1.5 Hz, 1H), 4.35 (dd, *J* = 36.5, 8.5 Hz, 1H), 2.34 (s, 3H), ****2.20 (s)***.

**$^{13}$C NMR (126 MHz, CDCl$_3$):** δ 172.1 (d, *J* = 23.1 Hz), 137.4 (d, *J* = 22.1 Hz), 137.0, 135.4, 134.7 (d, *J* = 4.6 Hz), 129.8 (d, *J* = 2.3 Hz), 129.2, 128.4 – 128.3 (m), 125.1 (d, *J* = 11.0 Hz), 119.1, 101.2 (d, *J* = 198.3 Hz), 56.2 (d, *J* = 18.9 Hz), 21.3.

**$^{19}$F NMR (376 MHz, CDCl$_3$):** δ -173.14 (d, *J* = 36.5 Hz, 1F).

**HRMS (ESI):** m/z calcd for $[M+Na]^+$ $C_{18}H_{18}ONFNa$: 306.1265. Found: 306.1267.

HPLC analysis using a chiral column (Cellulose 3 3μ column 22 ˚C, 0.5 ml/min, 95:5 hexane:isopropanol, 210 nm, $t_{major}$ = 28.985 min, $t_{minor}$ = 33.549 min, t = 20.668 min, t = 24.417 min).

(±)-**16:** (*rac*)-**[Ir]** and (*rac*)-Bn-BTM

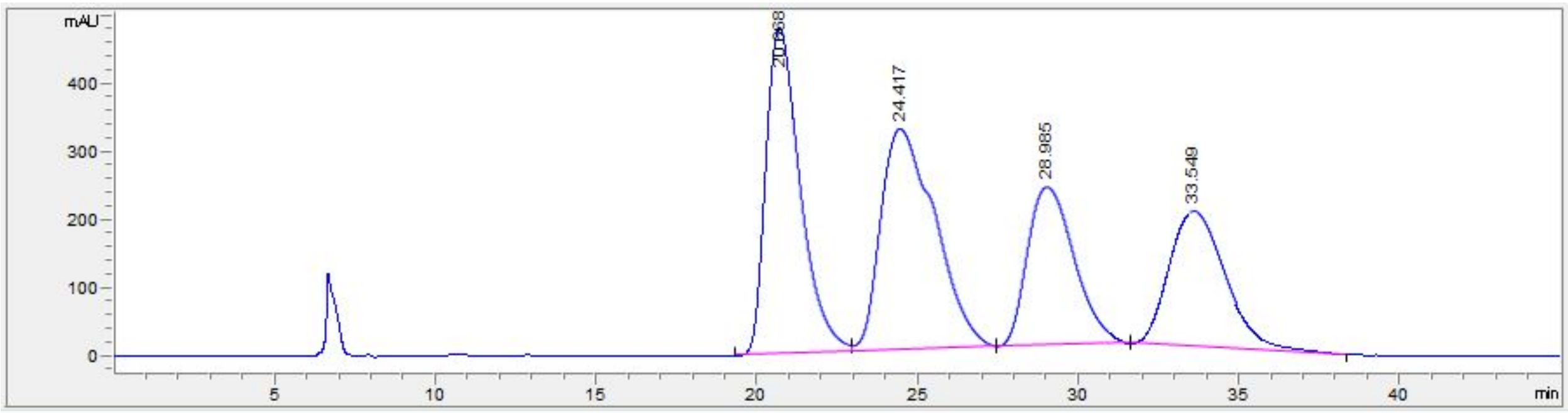

| # | Time | Area | Height | Width | Area% | Symmetry |
|---|---|---|---|---|---|---|
| 1 | 20.668 | 35286.4 | 478.4 | 1.1284 | 29.192 | 0.599 |
| 2 | 24.417 | 37939.2 | 323.3 | 1.7051 | 31.387 | 0.531 |
| 3 | 28.985 | 23550.5 | 231.6 | 1.5844 | 19.483 | 0.68 |
| 4 | 33.549 | 24099.9 | 198.5 | 1.8186 | 19.938 | 0.71 |

(*R*,*S*)-**16:** ($S_a$,*S*,*S*)-**[Ir]** and (*S*)-Me-BTM

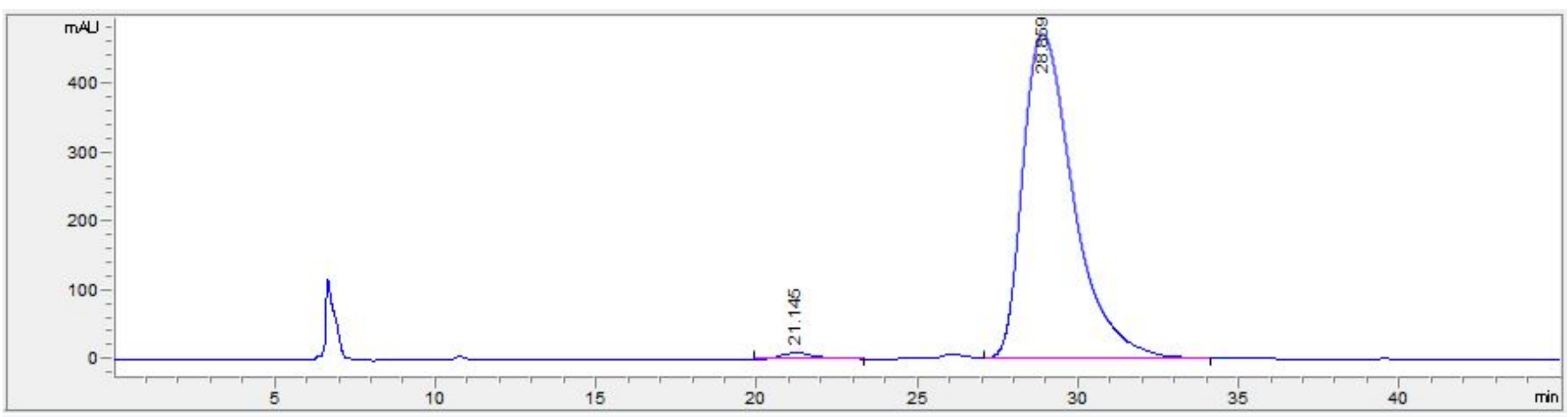

| # | Time | Area | Height | Width | Area% | Symmetry |
|---|---|---|---|---|---|---|
| 1 | 21.145 | 704.8 | 9.1 | 0.931 | 1.348 | 0.7 |
| 2 | 28.859 | 51565.6 | 469.8 | 1.6727 | 98.652 | 0.604 |

**Perfluorophenyl (2*R*,3*R*)-2-fluoro-3-(4-fluorophenyl)-2-phenylpent-4-enoate ((*R*,*R*)-17):** Prepared according to General Procedure B using pentafluorophenyl ester **1a** (32 mg, 0.1 mmol, 1 equiv.), *t*-butyl carbonate **2c** (37.8 mg, 0.15 mmol, 1.5 equiv.), ($R_a$,*R*,*R*)-**[Ir]** (4.2 mg, 4 mol%), (*S*)-Me-BTM (3.8 mg, 20 mol%) and DIPEA (22.6 μL, 0.13 mmol, 1.3 equiv.) in 1,4-dioxane (0.3 ml, 0.3 M). The *title compound* was obtained (39.8 mg, 0.0875 mmol, 87.5%) as a colorless oil following purification by column chromatography [$SiO_2$, 0.25% Ethyl Acetate in Petroleum Ether]. The enantiomeric ratio (>99:1 *er*) of the corresponding primary amide was determined by chiral HPLC in comparison with the racemate. Diastereomeric ratio (97:3) was determined by $^{19}$F-NMR analysis of the crude reaction mixture.

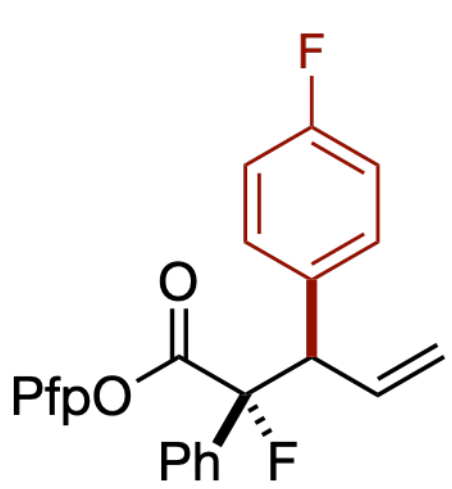

**IR (film):** 3065 (w), 1783 (m), 1640 (w), 1605 (w), 1519 (s), 1510 (s), 1472 (w), 1204 (m), 1181 (m), 1009 (s), 996 (s), 747 (m) cm$^{-1}$.

**$^{1}$H NMR (500 MHz, CDCl$_3$):** δ 7.45 – 7.36 (m, 2H), 7.32 – 7.22 (m, 3H), 7.08 (ddd, *J* = 8.8, 5.3, 1.4 Hz, 2H), 6.83 (t, *J* = 8.7 Hz, 2H), 6.34 (ddd, *J* = 17.1, 10.3, 8.6 Hz, 1H), 5.41 – 5.34 (m, 2H), 4.39 (dd, *J* = 32.5, 8.5 Hz, 1H).

**$^{13}$C NMR (126 MHz, CDCl$_3$):** δ 166.4 (d, *J* = 28.1 Hz), 162.1 (d, *J* = 246.0 Hz), 135.0 (d, *J* = 22.5 Hz), 134.3 (d, *J* = 5.1 Hz), 132.3 (d, *J* = 3.3 Hz), 131.2 (dd, *J* = 8.1, 2.5 Hz), 129.1, 128.6 (d, *J* = 2.2 Hz), 124.9 (d, *J* = 10.1 Hz), 120.1, 115.2 (d, *J* = 21.2 Hz), 99.7 (d, *J* = 201.5 Hz), 56.8 (d, *J* = 19.2 Hz).

**$^{19}$F NMR (376 MHz, CDCl$_3$):** δ -115.15 (ddd, *J* = 14.0, 8.8, 5.3 Hz, 1F), -151.42 – -151.59 (m, 2F), -156.85 (t, *J* = 21.7 Hz, 1F), -161.64 – -161.90 (m, 2F), -175.29 (d, *J* = 32.9 Hz, 1F).

**HRMS (EI):** m/z calcd for $[M]^+$ $C_{23}H_{13}F_7O_2$: 454.0804. Found: 454.0805.

**(2*R*,3*R*)-2-Fluoro-3-(4-fluorophenyl)-2-phenylpent-4-enamide ((*R*,*R*)-17-NH$_2$):**

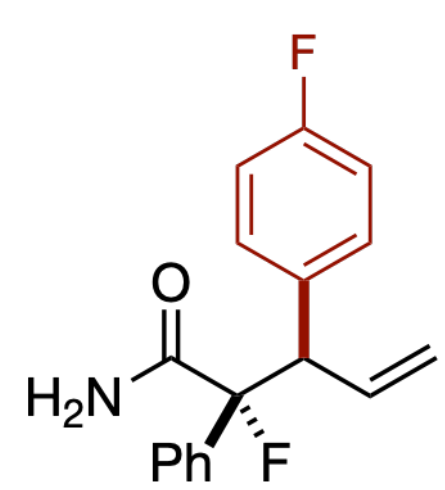


**IR (film):** 3449 (m), 3192 (br), 1670 (m), 1638 (s), 1512 (s), 1441 (w), 1389 (w) cm$^{-1}$.

**$^{1}$H NMR (500 MHz, CDCl$_3$):** δ 7.49 – 7.33 (m, 2H), 7.23 – 7.15 (m, 3H), 7.03 (ddd, *J* = 8.8, 5.3, 1.3 Hz, 2H), 6.86 – 6.73 (m, 2H), 6.42 (s, 1H), 6.25 (ddd, *J* = 17.0, 10.4, 8.2 Hz, 1H), 5.52 (s, 1H), 5.31 – 5.18 (m, 2H), 4.38 (dd, *J* = 35.0, 8.5 Hz, 1H).

**$^{13}$C NMR (126 MHz, CDCl$_3$):** δ 172.3 (d, *J* = 22.9 Hz), 161.8 (d, *J* = 245.2 Hz), 136.7 (d, *J* = 22.0 Hz), 135.0 (d, *J* = 5.3 Hz), 133.4 (d, *J* = 3.4 Hz), 131.1 (dd, *J* = 8.0, 2.4 Hz), 128.2, 128.1 (d, *J* = 2.1 Hz), 124.7 (d, *J* = 10.6 Hz), 119.4, 115.0 (d, *J* = 21.3 Hz), 101.5 (d, *J* = 197.6 Hz), 56.3 (d, *J* = 19.5 Hz).

**$^{19}$F NMR (376 MHz, CDCl$_3$):** δ -115.88 (tt, *J* = 8.7, 5.4 Hz, 1F), -172.37 (d, *J* = 34.7 Hz, 1F).

**HRMS (ESI):** m/z calcd for $[M+Na]^+$ $C_{17}H_{15}ONF_2Na$: 310.1014. Found: 310.1014.

HPLC analysis using a chiral column (Chiralpak IB 3μ column 22 ˚C, 1.0 ml/min, 95:5 hexane:isopropanol, 210 nm, $t_{major}$ = 12.376 min, $t_{minor}$ = 38.844 min, t = 10.598 min, t = 21.206 min).

(±)-**17:** (*rac*)-**[Ir]** and (*rac*)-Bn-BTM

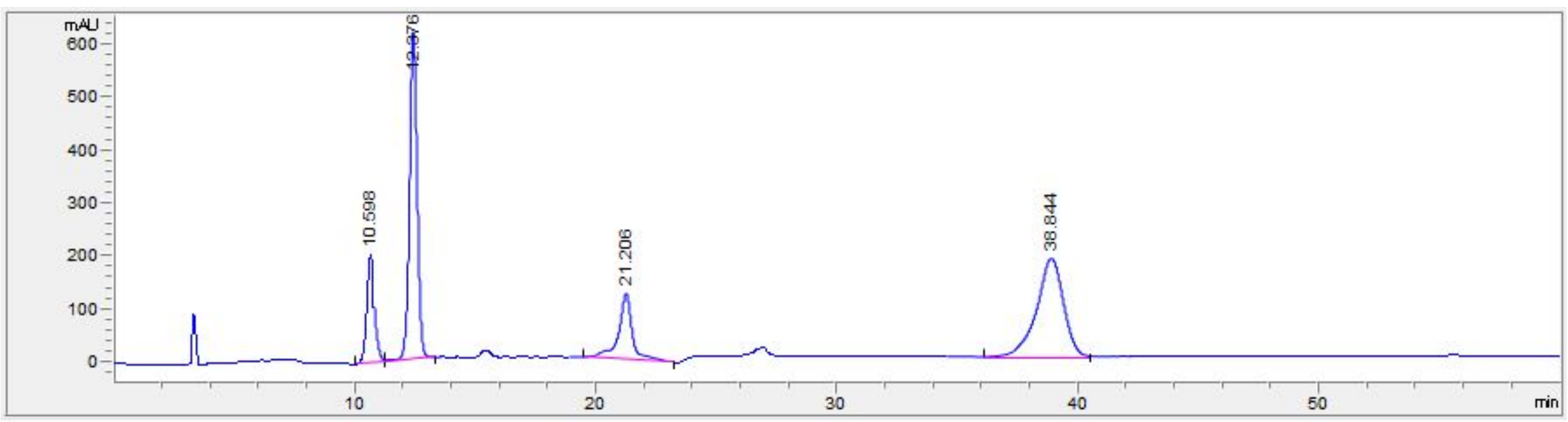


| # | Time | Area | Height | Width | Area% | Symmetry |
|---|---|---|---|---|---|---|
| 1 | 10.598 | 4503.8 | 204 | 0.3276 | 11.764 | 0.8 |
| 2 | 12.376 | 13683.8 | 616.8 | 0.3388 | 35.743 | 0.901 |
| 3 | 21.206 | 5113.3 | 124.8 | 0.5799 | 13.356 | 1.157 |
| 4 | 38.844 | 14983 | 187.3 | 1.333 | 39.137 | 1.228 |

(*R*,*R*)-**17:** ($R_a$,*R,R*)-**[Ir]** and (*S*)-Me-BTM

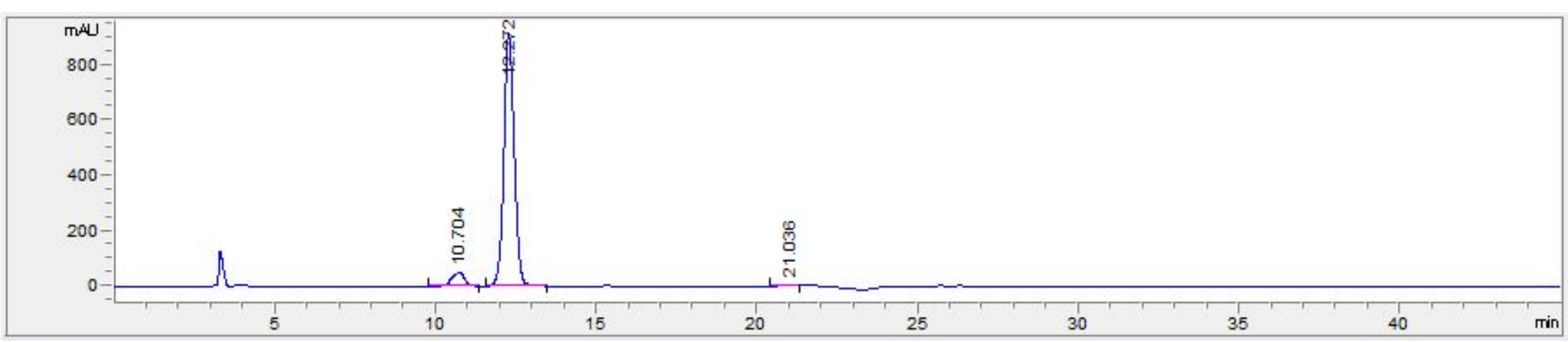


| # | Time | Area | Height | Width | Area% | Symmetry |
|---|---|---|---|---|---|---|
| 1 | 10.704 | 1345.1 | 47.2 | 0.4582 | 6.299 | 1.347 |
| 2 | 12.272 | 19958.3 | 912.3 | 0.3352 | 93.465 | 0.814 |
| 3 | 21.036 | 50.3 | 1.7 | 0.3585 | 0.236 | 2.386 |

**Perfluorophenyl (2*R*,3*S*)-2-fluoro-3-(4-fluorophenyl)-2-phenylpent-4-enoate ((*R,S*)-17):**

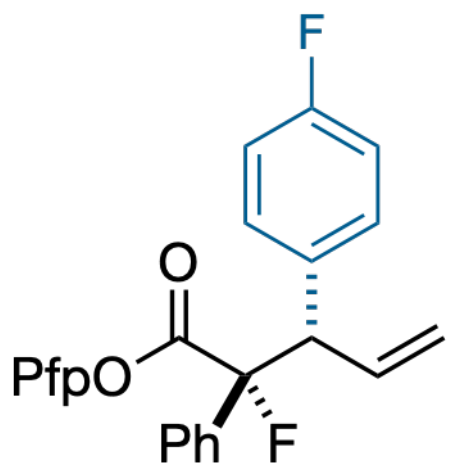


Prepared according to General Procedure B using pentafluorophenyl ester **1a** (32 mg, 0.1 mmol, 1 equiv.), *t*-butyl carbonate **2c** (37.8 mg, 0.15 mmol, 1.5 equiv.), ($S_a$,*S*,*S*)-**[Ir]** (4.2 mg, 4 mol%), (*S*)-Me-BTM (3.8 mg, 20 mol%) and DIPEA (22.6 μL, 0.13 mmol, 1.3 equiv.) in 1,4-dioxane (0.3 ml, 0.3 M). The diastereomeric ratio was determined to be 1:12. The *title compound* was obtained (34.2 mg, 0.075 mmol, 75%) as a white solid following purification by column chromatography [$SiO_2$, 0.25% Ethyl Acetate in Petroleum Ether]. The enantiomeric ratio (>99:1 *er*) of the corresponding primary amide was determined by chiral HPLC in comparison with the racemate. Diastereomeric ratio (92:8) was determined by $^{19}$F-NMR analysis of the crude reaction mixture.

**IR (film):** 3065 (w), 1781 (m), 1640 (w), 1606 (w), 1518 (s), 1510 (s), 1471 (w), 1224 (m), 1198 (m), 1008 (s), 997 (s), 747 (m) $cm^{-1}$.

**$^1$H NMR (500 MHz, CDCl$_3$):** δ 7.71 – 7.63 (m, 2H), 7.52 – 7.39 (m, 5H), 7.07 (t, *J* = 8.7 Hz, 2H), ***6.35 (ddd, J = 17.1, 10.3, 8.6 Hz)**, 5.94 (ddd, *J* = 17.5, 10.4, 7.7 Hz, 1H), ***5.47 – 5.31 (m,)**, 5.08 (dt, *J* = 10.2, 1.2 Hz, 1H), 4.90 (d, *J* = 17.1 Hz, 1H), 4.43 (dd, *J* = 33.5, 7.5 Hz, 1H).

**$^{13}$C NMR (126 MHz, CDCl$_3$):** δ 166.0 (d, *J* = 28.5 Hz), 162.6 (d, *J* = 246.5 Hz), 135.4 (d, *J* = 22.6 Hz), 133.7 (d, *J* = 5.0 Hz), 133.3 (d, *J* = 3.4 Hz), 131.3 (dd, *J* = 8.1, 3.3 Hz), 129.5, 128.9 (d, *J* = 2.1 Hz), 125.3 (d, *J* = 10.2 Hz), 120.1, 115.7 (d, *J* = 21.3 Hz), 99.2 (d, *J* = 201.3 Hz), 56.1 (d, *J* = 19.0 Hz).

**$^{19}$F NMR (376 MHz, CDCl$_3$):** δ -114.62 (ddd, *J* = 14.0, 8.6, 5.2 Hz, 1F), -151.84 – -152.07 (m, 2F), -157.00 (t, *J* = 21.7 Hz, 1F), -161.78 – -161.97 (m, 2F), -174.04 (d, *J* = 33.6 Hz, 1F).

**HRMS (EI):** m/z calcd for $[M]^+$ $C_{23}H_{13}F_7O_2$: 454.0804. Found: 454.0814.

**(2*R*,3*S*)-2-Fluoro-3-(4-fluorophenyl)-2-phenylpent-4-enamide ((*R*,*S*)-17-$NH_2$):**

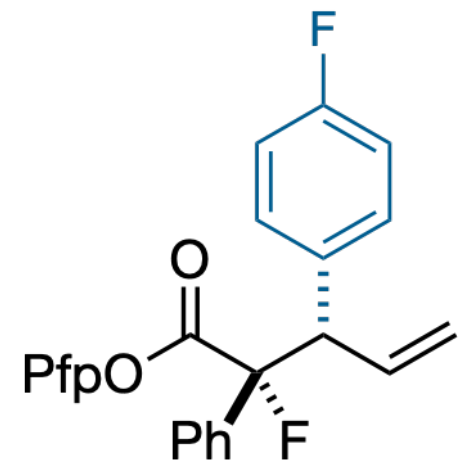


**IR (film):** 3444 (m), 3198 (br), 1669 (s), 1602 (w), 1509 (m), 1448 (w), 1398 (w) $cm^{-1}$.

**$^{1}H$ NMR (500 MHz, $CDCl_3$):** δ 7.71 – 7.60 (m, 2H), 7.44 – 7.37 (m, 4H), 7.37 – 7.31 (m, 1H), 7.06 – 6.97 (m, 2H), , ***6.41 (s)**, ***6.25 (ddd, J = 17.0, 10.4, 8.2 Hz)**, 5.91 (ddd, *J* = 17.1, 10.4, 8.1 Hz, 1H), ***5.46 (s)**, ***5.35 – 5.19 (m)**, 5.08 (s, 1H), 5.00 (dt, *J* = 10.4, 1.2 Hz, 1H), 4.85 (dd, *J* = 17.1, 1.5 Hz, 1H), 4.39 (dd, *J* = 36.0, 8.0 Hz, 1H).

**$^{13}C$ NMR (126 MHz, $CDCl_3$):** δ 171.9 (d, *J* = 23.0 Hz), 162.2 (d, *J* = 245.7 Hz), 137.1 (d, *J* = 22.0 Hz), 134.3 (d, *J* = 4.4 Hz), 134.2 (d, *J* = 3.4 Hz), 131.6 (dd, *J* = 7.9, 2.4 Hz), 128.5, 128.4 (d, *J* = 2.3 Hz), 125.1 (d, *J* = 10.9 Hz), 119.5, 115.3 (d, *J* = 21.1 Hz), 101.1 (d, *J* = 198.2 Hz), 55.7 (d, *J* = 19.0 Hz).

**$^{19}F$ NMR (376 MHz, $CDCl_3$):** δ -115.46 (ddd, *J* = 14.0, 8.7, 5.3 Hz, 1F), -173.59 (d, *J* = 36.5 Hz, 1F).

**HRMS (ESI):** m/z calcd for $[M+Na]^+$ $C_{17}H_{15}ONF_2Na$: 310.1014. Found: 310.1014.

HPLC analysis using a chiral column (Chiralpak IB 3µ column 22 ˚C, 1.0 ml/min, 95:5 hexane:isopropanol, 210 nm, $t_{major}$ = 10.598 min, $t_{minor}$ = 21.206 min, t = 12.376 min, t = 38.844 min).

(±)-**17:** (*rac*)-**[Ir]** and (*rac*)-Bn-BTM

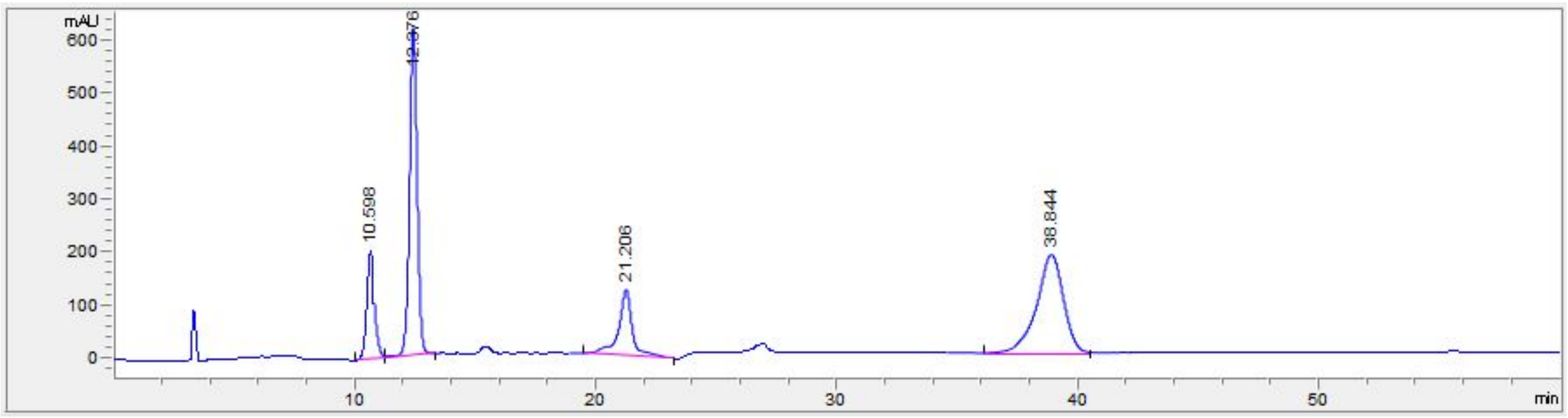


| # | Time | Area | Height | Width | Area% | Symmetry |
|---|---|---|---|---|---|---|
| 1 | 10.598 | 4503.8 | 204 | 0.3276 | 11.764 | 0.8 |
| 2 | 12.376 | 13683.8 | 616.8 | 0.3388 | 35.743 | 0.901 |
| 3 | 21.206 | 5113.3 | 124.8 | 0.5799 | 13.356 | 1.157 |
| 4 | 38.844 | 14983 | 187.3 | 1.333 | 39.137 | 1.228 |

(*R*,*S*)-**17:** ($S_a$,*S*,*S*)-**[Ir]** and (*S*)-Me-BTM

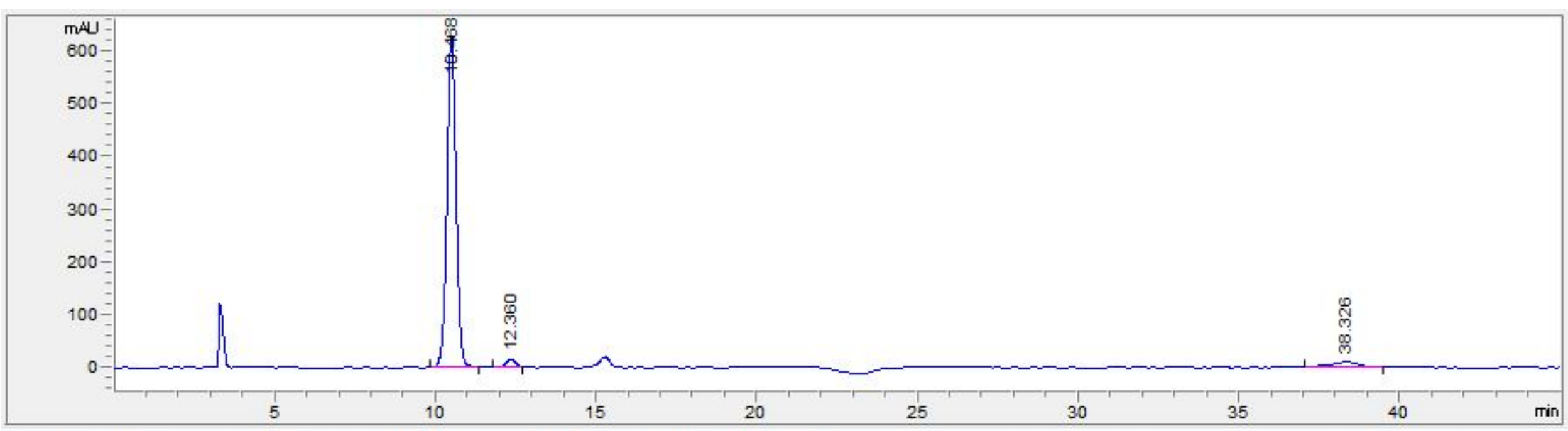


| # | Time | Area | Height | Width | Area% | Symmetry |
|---|---|---|---|---|---|---|
| 1 | 10.468 | 12170.7 | 628.8 | 0.2958 | 92.858 | 0.888 |
| 2 | 12.36 | 316.3 | 15.1 | 0.316 | 2.413 | 1.195 |
| 3 | 38.326 | 619.9 | 10.7 | 0.7577 | 4.729 | 1.254 |

**Perfluorophenyl (2*R*,3*R*)-3-(4-chlorophenyl)-2-fluoro-2-phenylpent-4-enoate ((*R*,*R*)-18):**

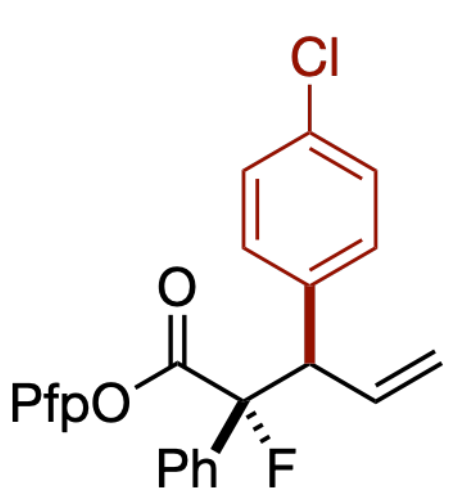


Prepared according to General Procedure B using pentafluorophenyl ester **1a** (32 mg, 0.1 mmol, 1 equiv.), *t*-butyl carbonate **2d** (40.3 mg, 0.15 mmol, 1.5 equiv.), ($R_a$,*R*,*R*)-**[Ir]** (4.2 mg, 4 mol%), (*S*)-Me-BTM (3.8 mg, 20 mol%) and DIPEA (22.6 μL, 0.13 mmol, 1.3 equiv.) in 1,4-dioxane (0.3 ml, 0.3 M). The diastereomeric ratio was determined to be 23:1. The *title compound* was obtained (40.6 mg, 0.086 mmol, 86%) as a colorless oil following purification by column chromatography [$SiO_2$, 0.25% Ethyl Acetate in Petroleum Ether]. The enantiomeric ratio (>99:1 *er*) of the corresponding

primary amide was determined by chiral HPLC in comparison with the racemate. Diastereomeric ratio (96:4) was determined by $^{19}$F-NMR analysis of the crude reaction mixture.

**IR (film):** 3065 (w), 1783 (m), 1640 (w), 1597 (w), 1519 (s), 1493 (m), 1204 (m), 1181 (m), 1010 (s), 997 (s), 748 (m) cm$^{-1}$.

**$^{1}$H NMR (500 MHz, $CDCl_3$):** δ 7.38 – 7.30 (m, 2H), 7.26 – 7.14 (m, 3H), 7.04 (d, *J* = 8.5 Hz, 2H), 6.98 (dd, *J* = 8.5, 1.3 Hz, 2H), 6.26 (ddd, *J* = 17.0, 10.3, 8.7 Hz, 1H), 5.37 – 5.25 (m, 2H), 4.32 (dd, *J* = 33.0, 8.5 Hz, 1H).

**$^{13}$C NMR (126 MHz, $CDCl_3$):** δ 166.3 (d, *J* = 28.1 Hz), 135.1, 134.8 (d, *J* = 22.5 Hz), 134.1 (d, *J* = 5.0 Hz), 133.3, 130.9 (d, *J* = 2.4 Hz), 129.2, 128.6 (d, *J* = 2.2 Hz), 128.5, 124.9 (d, *J* = 10.0 Hz), 120.3, 99.6 (d, *J* = 201.5 Hz), 56.9 (d, *J* = 19.0 Hz).

**$^{19}$F NMR (376 MHz, $CDCl_3$):** δ -151.39 – -151.64 (m, 2F), -156.80 (t, *J* = 21.7 Hz, 1F), -161.66 – -161.89 (m, 2F), -175.12 (d, *J* = 32.8 Hz, 1F).

**HRMS (EI):** m/z calcd for $[M]^+$ $C_{23}H_{13}ClF_6O_2$: 470.0508. Found: 470.0510.

**(2*R*,3*R*)-3-(4-Chlorophenyl)-2-fluoro-2-phenylpent-4-enamide ((*R,R*)-18-$NH_2$):**

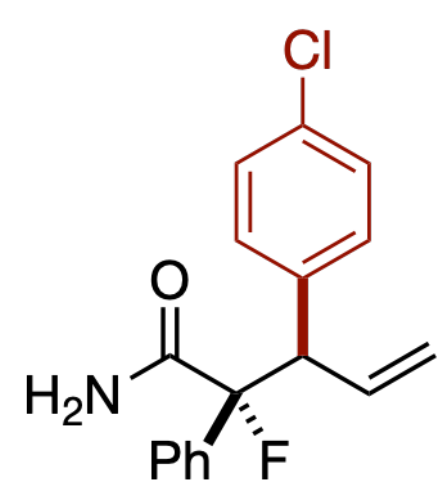


**IR (film):** 3429 (m), 3178 (br), 1670 (s), 1607 (w), 1493 (w), 1448 (w), 1396 (w), 787 (w) cm$^{-1}$.

**$^{1}$H NMR (500 MHz, $CDCl_3$):** δ 7.44 – 7.35 (m, 2H), 7.24 – 7.15 (m, 3H), 7.11 – 7.06 (m, 2H), 7.03 – 6.98 (m, 2H), 6.41 (s, 1H), 6.24 (ddd, *J* = 17.0, 10.4, 8.2 Hz, 1H), 5.50 (s, 1H), 5.34 – 5.17 (m, 2H), 4.38 (dd, *J* = 34.5, 8.5 Hz, 1H).

**$^{13}$C NMR (126 MHz, $CDCl_3$):** δ 172.2 (d, *J* = 22.9 Hz), 136.6 (d, *J* = 22.0 Hz), 136.2, 134.8 (d, *J* = 5.4 Hz), 132.8, 131.0 (d, *J* = 2.4 Hz), 128.3, 128.2 (d, *J* = 2.1 Hz), 124.7 (d, *J* = 10.5 Hz), 119.6, 101.4 (d, *J* = 197.6 Hz), 56.4 (d, *J* = 19.4 Hz).

**$^{19}$F NMR (376 MHz, $CDCl_3$):** δ -172.24 (d, *J* = 34.8 Hz, 1F).

**HRMS (ESI):** m/z calcd for $[M+Na]^+$ $C_{17}H_{15}ONClFNa$: 326.0718. Found: 326.0718.

HPLC analysis using a chiral column (Chiralpak IB 3μ column 22 ˚C, 1.0 ml/min, 95:5 hexane:isopropanol, 210 nm, $t_{major}$ = 13.169 min, $t_{minor}$ = 42.912 min, t = 10.828 min, t = 22.333 min).

(+)-**18:** (*rac*)-**[Ir]** and (*rac*)-Bn-BTM

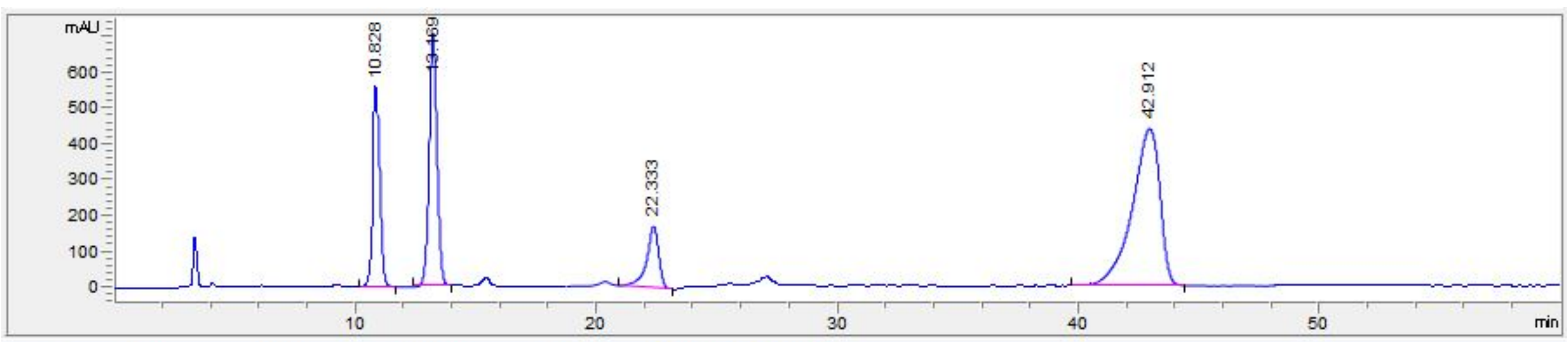


| # | Time | Area | Height | Width | Area% | Symmetry |
|---|---|---|---|---|---|---|
| 1 | 10.828 | 11287.1 | 558.2 | 0.3079 | 16.378 | 0.896 |
| 2 | 13.169 | 16127 | 711.2 | 0.3445 | 23.400 | 0.856 |
| 3 | 22.333 | 6178.9 | 174.5 | 0.5211 | 8.966 | 1.449 |
| 4 | 42.912 | 35324.6 | 441.8 | 1.1728 | 51.256 | 1.732 |

(*R*,*R*)-**18:** (*R$_a$*,*R*,*R*)-**[Ir]** and (*S*)-Me-BTM

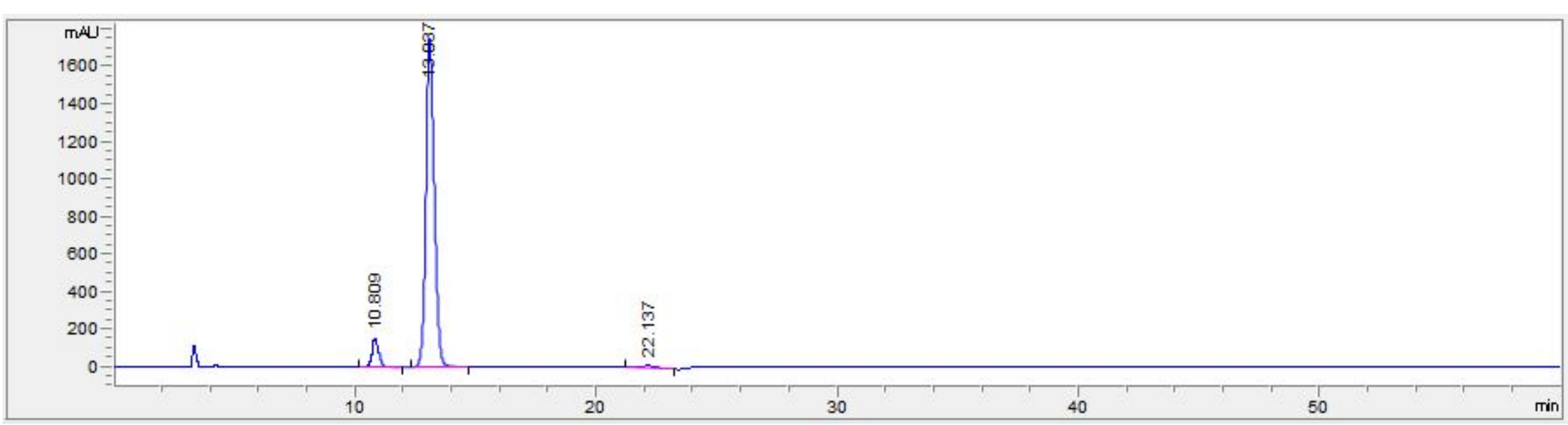


| # | Time | Area | Height | Width | Area% | Symmetry |
|---|---|---|---|---|---|---|
| 1 | 10.809 | 3149.4 | 150.8 | 0.3217 | 6.923 | 0.988 |
| 2 | 13.037 | 41773.5 | 1744.3 | 0.3634 | 91.826 | 0.686 |
| 3 | 22.137 | 568.9 | 11.8 | 0.6414 | 1.251 | 1.196 |

**Perfluorophenyl (2*R*,3*S*)-3-(4-chlorophenyl)-2-fluoro-2-phenylpent-4-enoate ((*R,S*)-18):** Prepared according to General Procedure B using pentafluorophenyl ester **1a** (32 mg, 0.1 mmol, 1 equiv.), *t*-butyl carbonate **2d** (40.3 mg, 0.15 mmol, 1.5 equiv.), ($S_a$,*S,S*)-**[Ir]** (4.2 mg, 4 mol%), (*S*)-Me-BTM (3.8 mg, 20 mol%) and DIPEA (22.6 µL, 0.13 mmol, 1.3 equiv.) in 1,4-dioxane (0.3 ml, 0.3 M). The diastereomeric ratio was determined to be 1:13. The *title compound* was obtained (38.6 mg, 0.082 mmol, 82%) as a white solid following purification by column chromatography [$SiO_2$, 0.25% Ethyl Acetate in Petroleum Ether]. The enantiomeric ratio (>99:1 *er*) of the corresponding primary amide was determined by chiral HPLC in comparison with the racemate. Diastereomeric ratio (93:7) was determined by $^{19}$F-NMR analysis of the crude reaction mixture.

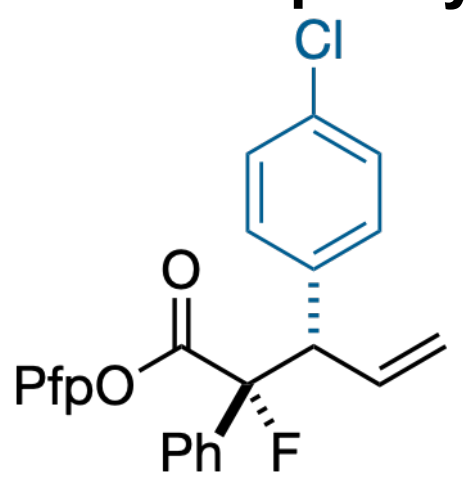


**IR (film):** 3065 (w), 1799 (w), 1780 (m), 1640 (w), 1596 (w), 1517 (s), 1493 (m), 1197 (m), 1007 (s), 996 (s), 749 (m) cm$^{-1}$.

**$^1$H NMR (500 MHz, $CDCl_3$):** δ 7.68 (dt, *J* = 6.7, 1.3 Hz, 2H), 7.48 (t, *J* = 7.3 Hz, 2H), 7.45 – 7.40 (m, 3H), 7.37 – 7.34 (m, 2H), ***6.33 (ddd, J = 17.0, 10.3, 8.6 Hz)***, 5.93 (ddd, *J* = 17.6, 10.3, 7.8 Hz, 1H), ***5.52 – 5.31 (m)***, 5.09 (d, *J* = 10.3 Hz, 1H), 4.91 (d, *J* = 17.1 Hz, 1H), 4.41 (dd, *J* = 33.5, 8 Hz, 1H).

**$^{13}$C NMR (126 MHz, $CDCl_3$):** δ 165.9 (d, *J* = 28.5 Hz), 136.1, 135.4 (d, *J* = 22.8 Hz), 134.0, 133.4 (d, *J* = 4.7 Hz), 131.0 (d, *J* = 3.3 Hz), 129.5, 129.0, 128.9 (d, *J* = 2.2 Hz), 125.3 (d, *J* = 10.1 Hz), 120.3, 99.1 (d, *J* = 201.4 Hz), 56.3 (d, *J* = 19.0 Hz).

**$^{19}$F NMR (376 MHz, $CDCl_3$):** δ -151.81 – -151.96 (m, 2F), -156.94 (t, *J* = 21.8 Hz, 1F), -160.84 – -162.06 (m, 2F), -173.79 (d, *J* = 33.5 Hz, 1F).

**HRMS (EI):** m/z calcd for $[M]^+$ $C_{23}H_{13}ClF_6O_2$: 470.0508. Found: 470.0514.

**(2*R*,3*S*)-3-(4-Chlorophenyl)-2-fluoro-2-phenylpent-4-enamide ((*R*,*S*)-18-$NH_2$):**

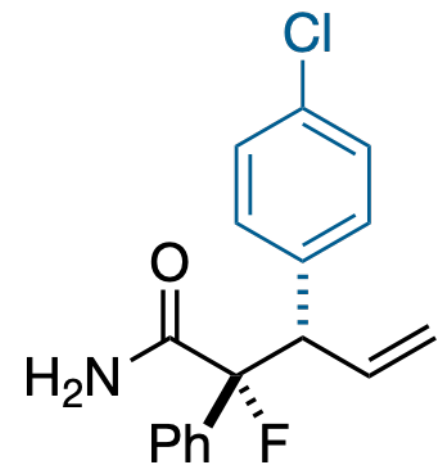


**IR (film):** 3438 (m), 3176 (br), 1668 (s), 1606 (w), 1491 (w), 1448 (w), 1405 (w), 598 (m) $cm^{-1}$.

**$^1$H NMR (500 MHz, $CDCl_3$):** δ 7.65 (dd, *J* = 7.3, 1.9 Hz, 2H), 7.44 – 7.32 (m, 5H), 7.30 (d, *J* = 8.6 Hz, 2H), ***6.23 (ddd, J = 16.7, 10.4, 8.3 Hz)**, 6.06 (s, 1H), 5.90 (ddd, *J* = 17.1, 10.4, 8.1 Hz, 1H), ***5.30 – 5.19 (m)**, 5.09 (s, 1H), 5.00 (d, *J* = 10.3 Hz, 1H), 4.85 (d, *J* = 17.1 Hz, 1H), 4.37 (dd, *J* = 36.0, 8.0 Hz, 1H).

**$^{13}$C NMR (126 MHz, $CDCl_3$):** δ 171.8 (d, *J* = 23.1 Hz), 137.0, 137.0 (d, *J* = 22.0 Hz), 134.1 (d, *J* = 4.4 Hz), 133.4, 131.4 (d, *J* = 2.3 Hz), 128.6, 128.6, 128.5 (d, *J* = 2.3 Hz), 125.0 (d, *J* = 11.0 Hz), 119.7, 101.0 (d, *J* = 198.7 Hz), 55.8 (d, *J* = 19.1 Hz).

**$^{19}$F NMR (376 MHz, $CDCl_3$):** δ -173.47 (d, *J* = 35.8 Hz, 1F).

**HRMS (ESI):** m/z calcd for $[M+Na]^+$ $C_{17}H_{15}ONClFNa$: 326.0718. Found: 326.0719.
HPLC analysis using a chiral column (Chiralpak IB 3µ column 22 ˚C, 1.0 ml/min, 95:5 hexane:isopropanol, 210 nm, $t_{major}$ = 10.828 min, $t_{minor}$ = 22.333 min, t = 13.169 min, t = 42.912 min).

(±)-**18:** (*rac*)-**[Ir]** and (*rac*)-Bn-BTM

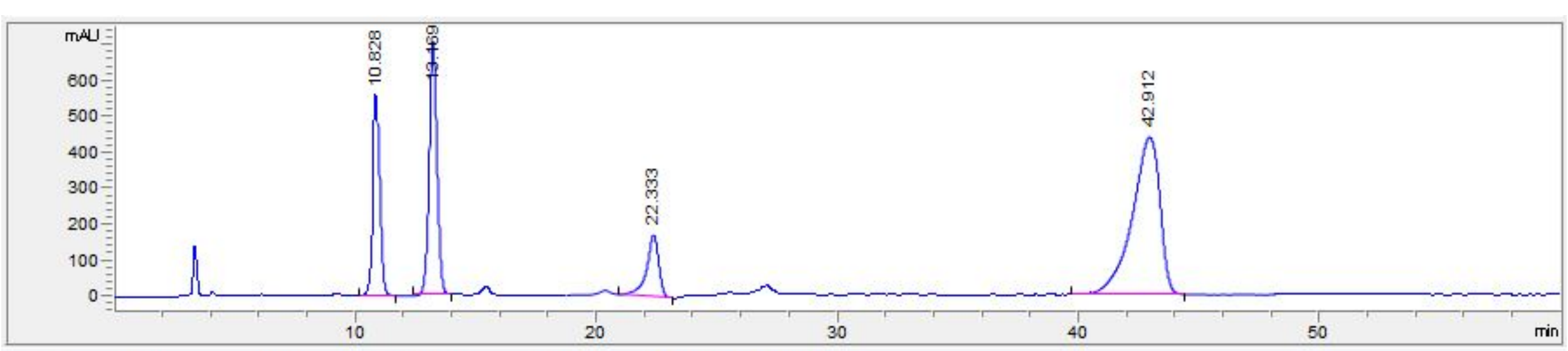


| # | Time | Area | Height | Width | Area% | Symmetry |
|---|---|---|---|---|---|---|
| 1 | 10.828 | 11287.1 | 558.2 | 0.3079 | 16.378 | 0.896 |
| 2 | 13.169 | 16127 | 711.2 | 0.3445 | 23.400 | 0.856 |
| 3 | 22.333 | 6178.9 | 174.5 | 0.5211 | 8.966 | 1.449 |
| 4 | 42.912 | 35324.6 | 441.8 | 1.1728 | 51.256 | 1.732 |

($R$,$S$)-**18:** ($S_a$,$S$,$S$)-**[Ir]** and ($S$)-Me-BTM

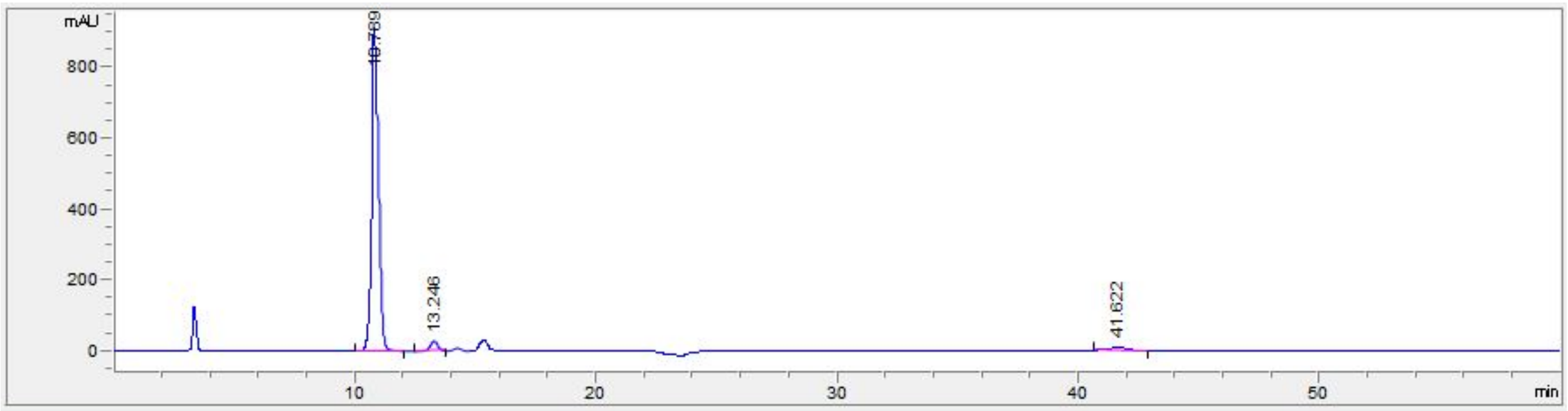


| # | Time | Area | Height | Width | Area% | Symmetry |
|---|---|---|---|---|---|---|
| 1 | 10.789 | 18461 | 911.4 | 0.3063 | 93.767 | 0.822 |
| 2 | 13.246 | 647 | 28.6 | 0.3495 | 3.286 | 1.173 |
| 3 | 41.622 | 580.2 | 10.9 | 0.7146 | 2.947 | 0.976 |

**Perfluorophenyl (2*R*,3*R*)-3-(4-bromophenyl)-2-fluoro-2-phenylpent-4-enoate ((*R*,*R*)-19):**

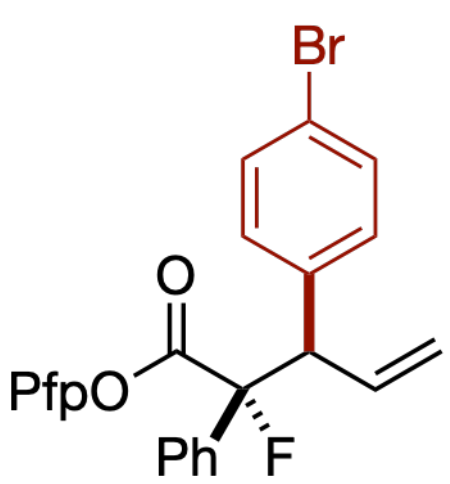


Prepared according to General Procedure B using pentafluorophenyl ester **1a** (32 mg, 0.1 mmol, 1 equiv.), *t*-butyl carbonate **2e** (47 mg, 0.15 mmol, 1.5 equiv.), ($R_a$,$R$,$R$)-**[Ir]** (4.2 mg, 4 mol%), ($S$)-Me-BTM (3.8 mg, 20 mol%) and DIPEA (22.6 µL, 0.13 mmol, 1.3 equiv.) in 1,4-dioxane (0.3 ml, 0.3 M). The diastereomeric ratio was determined to be 20.5:1. The *title compound* was obtained (42.8 mg, 0.083 mmol, 83%) as a colorless oil following purification by column chromatography [$SiO_2$, 0.25% Ethyl Acetate in Petroleum Ether]. The enantiomeric ratio (>99:1 *er*) of the corresponding primary amide was determined by chiral HPLC in comparison with the racemate. Diastereomeric ratio (95:5) was determined by $^{19}$F-NMR analysis of the crude reaction mixture.

**IR (film):** 3066 (w), 1783 (m), 1640 (w), 1592 (w), 1519 (s), 1490 (m), 1203 (m), 1180 (m), 1009 (s), 997 (s), 747 (m) cm$^{-1}$.

**$^1$H NMR (500 MHz, $CDCl_3$):** δ 7.42 – 7.37 (m, 2H), 7.31 – 7.19 (m, 5H), 6.99 (dd, $J$ = 8.5, 1.4 Hz, 2H), 6.32 (ddd, $J$ = 17.1, 10.3, 8.7 Hz, 1H), 5.41 – 5.32 (m, 2H), 4.37 (dd, $J$ = 32.5, 8.5 Hz, 1H).

**$^{13}$C NMR (126 MHz, $CDCl_3$):** δ 166.3 (d, $J$ = 28.1 Hz), 135.7, 134.8 (d, $J$ = 22.5 Hz), 134.0 (d, $J$ = 5.1 Hz), 131.5, 131.3 (d, $J$ = 2.5 Hz), 129.2, 128.7 (d, $J$ = 2.1 Hz), 124.9 (d, $J$ = 9.9 Hz), 121.5, 120.4, 99.5 (d, $J$ = 201.7 Hz), 56.9 (d, $J$ = 19.1 Hz).

**$^{19}$F NMR (376 MHz, CDCl$_3$):** δ -151.36 – -151.70 (m, 2F), -156.79 (t, *J* = 21.7 Hz, 1F), -161.50 – -161.94 (m, 2F), -175.09 (d, *J* = 32.8 Hz, 1F).

**HRMS (EI):** m/z calcd for $[M]^+$ $C_{23}H_{13}BrF_6O_2$: 514.0003. Found: 514.0003.

**(2*R*,3*R*)-3-(4-Bromophenyl)-2-fluoro-2-phenylpent-4-enamide ((*R*,*R*)-19-NH$_2$):**

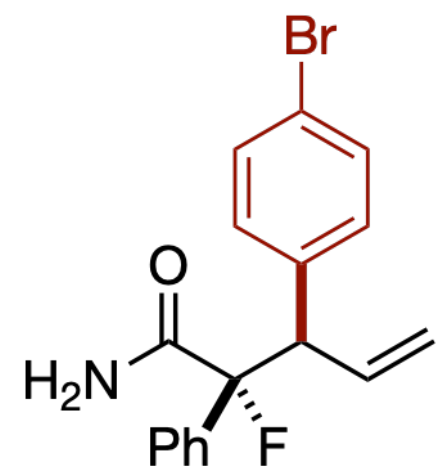

**IR (film):** 3428 (m), 3176 (br), 1670 (s), 1609 (w), 1489 (w), 1448 (w), 1398 (m), 615 (m) cm$^{-1}$.

**$^1$H NMR (500 MHz, CDCl$_3$):** δ 7.42 – 7.37 (m, 2H), 7.25 – 7.16 (m, 5H), 6.95 (dd, *J* = 8.4, 1.3 Hz, 2H), 6.41 (s, 1H), 6.23 (ddd, *J* = 17.0, 10.4, 8.2 Hz, 1H), 5.50 (s, 1H), 5.33 – 5.16 (m, 2H), 4.36 (dd, *J* = 35.0, 8.5 Hz, 1H).

**$^{13}$C NMR (126 MHz, CDCl$_3$):** δ 172.2 (d, *J* = 22.9 Hz), 136.8, 136.6 (d, *J* = 22.0 Hz), 134.7 (d, *J* = 5.2 Hz), 131.3 (d, *J* = 2.3 Hz), 131.3, 128.3, 128.2 (d, *J* = 2.2 Hz), 124.7 (d, *J* = 10.5 Hz), 121.0, 119.7, 101.3 (d, *J* = 197.5 Hz), 56.5 (d, *J* = 19.6 Hz).

**$^{19}$F NMR (376 MHz, CDCl$_3$):** δ -172.19 (d, *J* = 34.6 Hz, 1F).

**HRMS (ESI):** m/z calcd for $[M+Na]^+$ $C_{17}H_{15}ONBrFNa$:370.0213. Found: 370.0213.

HPLC analysis using a chiral column (Chiralpak IB 3μ column 22 ˚C, 1.0 ml/min, 95:5 hexane:isopropanol, 210 nm, $t_{major}$ = 13.795 min, $t_{minor}$ = 44.300 min, t = 11.369 min, t = 23.413 min).

(±)-**19:** (*rac*)-**[Ir]** and (*rac*)-Bn-BTM

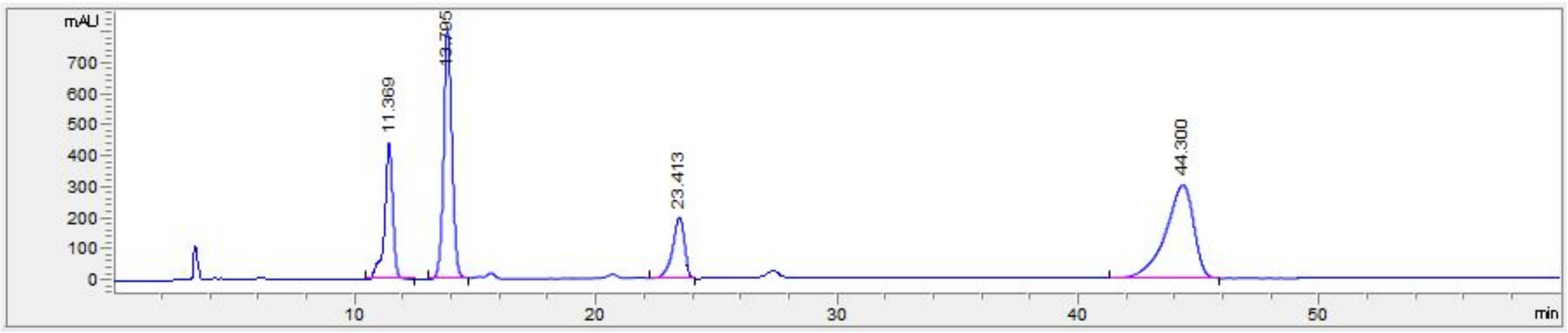


| # | Time | Area | Height | Width | Area% | Symmetry |
|---|---|---|---|---|---|---|
| 1 | 11.369 | 10111 | 443.1 | 0.3382 | 16.578 | 1.125 |
| 2 | 13.795 | 19338.1 | 817.3 | 0.3581 | 31.708 | 0.842 |
| 3 | 23.413 | 6904.4 | 199.1 | 0.5247 | 11.321 | 1.31 |
| 4 | 44.3 | 24635.3 | 303.8 | 1.2199 | 40.393 | 1.654 |

(*R*,*R*)-**19:** ($R_a$,*R*,*R*)-**[Ir]** and (*S*)-Me-BTM

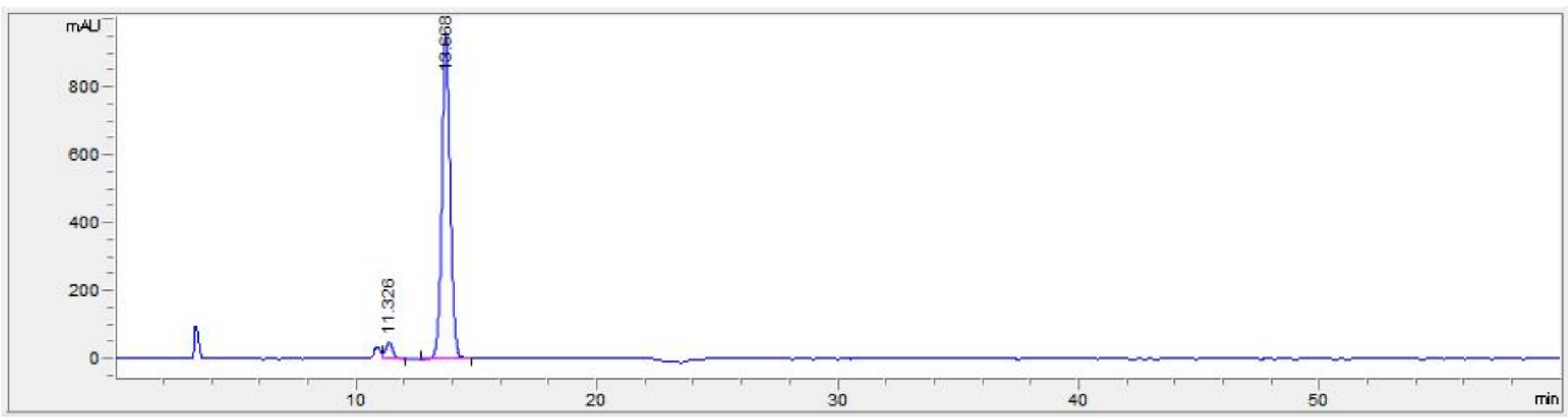


| # | Time | Area | Height | Width | Area% | Symmetry |
|---|---|---|---|---|---|---|
| 1 | 11.326 | 980.7 | 48.2 | 0.3094 | 4.116 | 0.983 |
| 2 | 13.668 | 22847.3 | 958.5 | 0.3601 | 95.884 | 0.808 |

**Perfluorophenyl (2*R*,3*S*)-3-(4-bromophenyl)-2-fluoro-2-phenylpent-4-enoate ((*R*,*S*)-19):**

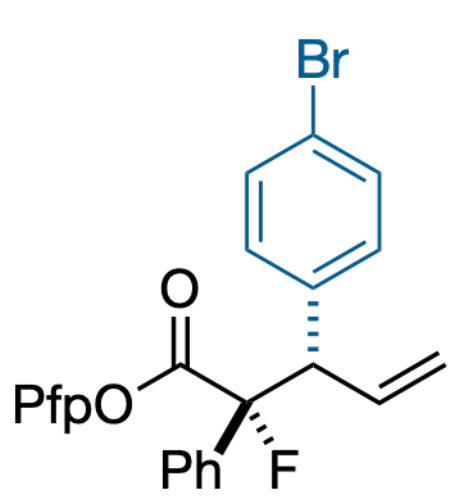


Prepared according to General Procedure B using pentafluorophenyl ester **1a** (32 mg, 0.1 mmol, 1 equiv.), *t*-butyl carbonate **2e** (47 mg, 0.15 mmol, 1.5 equiv.), ($S_a$,*S*,*S*)-**[Ir]** (4.2 mg, 4 mol%), (*S*)-Me-BTM (3.8 mg, 20 mol%) and DIPEA (22.6 µL, 0.13 mmol, 1.3 equiv.) in 1,4-dioxane (0.3 ml, 0.3 M). The diastereomeric ratio was determined to be 1:12. The *title compound* was obtained (41.1 mg, 0.080 mmol, 80%) as a white solid following purification by column chromatography [$SiO_2$, 0.25% Ethyl Acetate in Petroleum Ether]. The enantiomeric ratio (>99:1 *er*) of the corresponding primary amide was determined by chiral HPLC in comparison with the racemate.

Diastereomeric ratio (93:7) was determined by $^{19}$F-NMR analysis of the crude reaction mixture.

**IR (film):** 3066 (w), 1799 (w), 1780 (m), 1640 (w), 1592 (w), 1518 (s), 1490 (m), 1197 (m), 1008 (s), 997 (s), 748 (m) cm$^{-1}$.

**$^{1}$H NMR (500 MHz, $CDCl_3$):** δ 7.71 – 7.64 (m, 2H), 7.56 – 7.40 (m, 5H), 7.39 – 7.32 (m, 2H), ***6.33 (ddd, J = 17.1, 10.3, 8.7 Hz)***, 5.92 (ddd, *J* = 17.5, 10.4, 7.8 Hz, 1H), ***5.47 – 5.33 (m)***, 5.08 (dt, *J* = 10.4, 1.2 Hz, 1H), 4.91 (dt, *J* = 17.1, 1.3 Hz, 1H), 4.40 (dd, *J* = 33.5, 7.5 Hz, 1H).

**$^{13}$C NMR (126 MHz, $CDCl_3$):** δ 165.9 (d, *J* = 28.6 Hz), 136.6, 135.3 (d, *J* = 22.6 Hz), 133.4 (d, *J* = 4.8 Hz), 132.0, 131.3 (d, *J* = 3.2 Hz), 129.5, 128.9 (d, *J* = 2.2 Hz), 125.2 (d, *J* = 10.3 Hz), 122.2, 120.3, 99.0 (d, *J* = 201.5 Hz), 56.3 (d, *J* = 18.9 Hz).

**$^{19}$F NMR (376 MHz, $CDCl_3$):** δ -151.78 – -151.94 (m, 2F), -156.92 (t, *J* = 21.6 Hz, 1F), -161.52 – -161.93 (m, 2F), -173.75 (d, *J* = 33.6 Hz, 1F).

**HRMS (EI):** m/z calcd for $[M]^+$ $C_{23}H_{13}BrF_6O_2$: 514.0003. Found: 514.0005.

**(2*R*,3*S*)-3-(4-Bromophenyl)-2-fluoro-2-phenylpent-4-enamide ((*R*,*S*)-19-$NH_2$):**

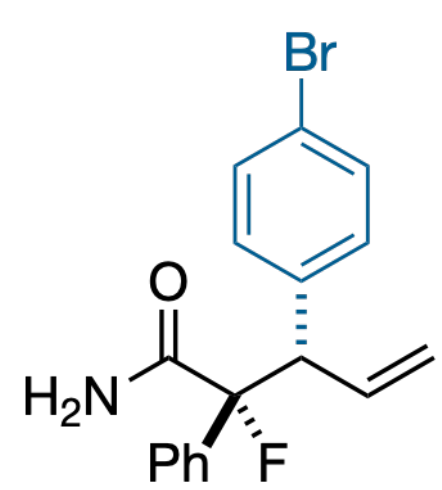


**IR (film):** 3437 (m), 3181 (br), 1668 (s), 1605 (w), 1488 (w), 1448 (w), 1402 (w), 598 (m) cm$^{-1}$.

**$^{1}$H NMR (500 MHz, $CDCl_3$):** δ 7.67 – 7.62 (m, 2H), 7.49 – 7.43 (m, 2H), 7.43 – 7.37 (m, 2H), 7.37 – 7.32 (m, 1H), 7.30 (dd, *J* = 8.4, 1.6 Hz, 2H), , ***6.40 (s)***, ***6.22 (ddd, J = 17.1, 10.4, 8.2 Hz)***, 6.06 (s, 1H), 5.89 (ddd, *J* = 17.1, 10.4, 8.1 Hz, 1H), ***5.51 (s)***, ***5.34 – 5.19 (m)***, 5.13 (s, 1H), 5.00 (dt, *J* = 10.4, 1.2 Hz, 1H), 4.85 (dd, *J* = 17.1, 1.4 Hz, 1H), 4.36 (dd, 1H).

**$^{13}$C NMR (126 MHz, CDCl$_3$):** δ 171.8 (d, $J$ = 23.1 Hz), 137.5, 136.9 (d, $J$ = 21.9 Hz), 134.0 (d, $J$ = 4.3 Hz), 131.8 (d, $J$ = 2.3 Hz), 131.6, 128.6, 128.5 (d, $J$ = 2.3 Hz), 125.0 (d, $J$ = 11.1 Hz), 121.5, 119.7, 100.9 (d, $J$ = 198.6 Hz), 55.9 (d, $J$ = 18.9 Hz).

**$^{19}$F NMR (376 MHz,CDCl$_3$):** δ -173.46 (d, $J$ = 35.5 Hz, 1F).

**HRMS (ESI):** m/z calcd for $[M+Na]^+$ $C_{17}H_{15}ONBrFNa$: 370.0213. Found: 370.0214.

HPLC analysis using a chiral column (Chiralpak IB 3μ column 22 ˚C, 1.0 ml/min, 95:5 hexane:isopropanol, 210 nm, $t_{major}$ = 11.369 min, $t_{minor}$ = 23.413 min, t = 13.795 min, t = 44.300 min).

(+)-**19:** (*rac*)-**[Ir]** and (*rac*)-Bn-BTM

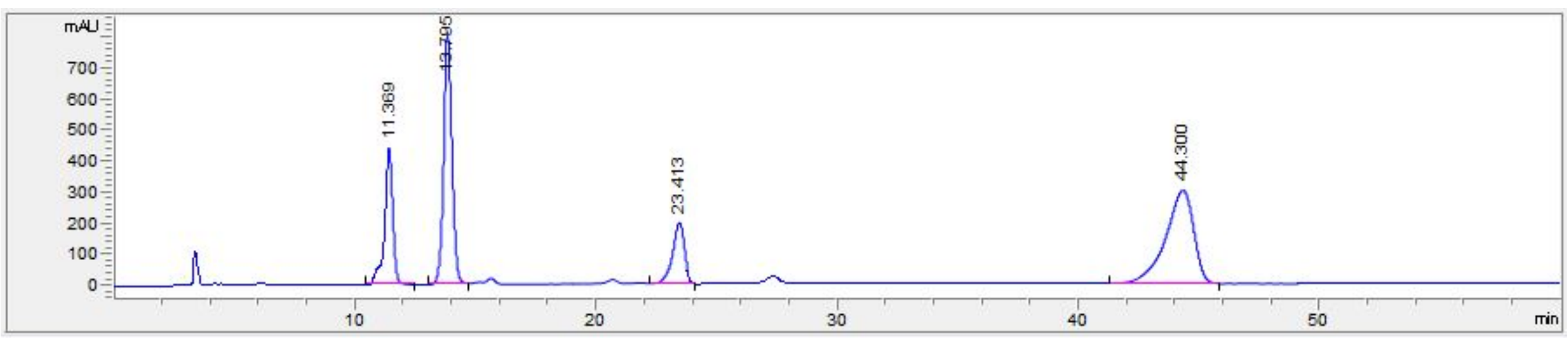


| # | Time | Area | Height | Width | Area% | Symmetry |
|---|---|---|---|---|---|---|
| 1 | 11.369 | 10111 | 443.1 | 0.3382 | 16.578 | 1.125 |
| 2 | 13.795 | 19338.1 | 817.3 | 0.3581 | 31.708 | 0.842 |
| 3 | 23.413 | 6904.4 | 199.1 | 0.5247 | 11.321 | 1.31 |
| 4 | 44.3 | 24635.3 | 303.8 | 1.2199 | 40.393 | 1.654 |

(*R*,*S*)-**19:** (*S$_a$*,*S*,*S*)-**[Ir]** and (*S*)-Me-BTM

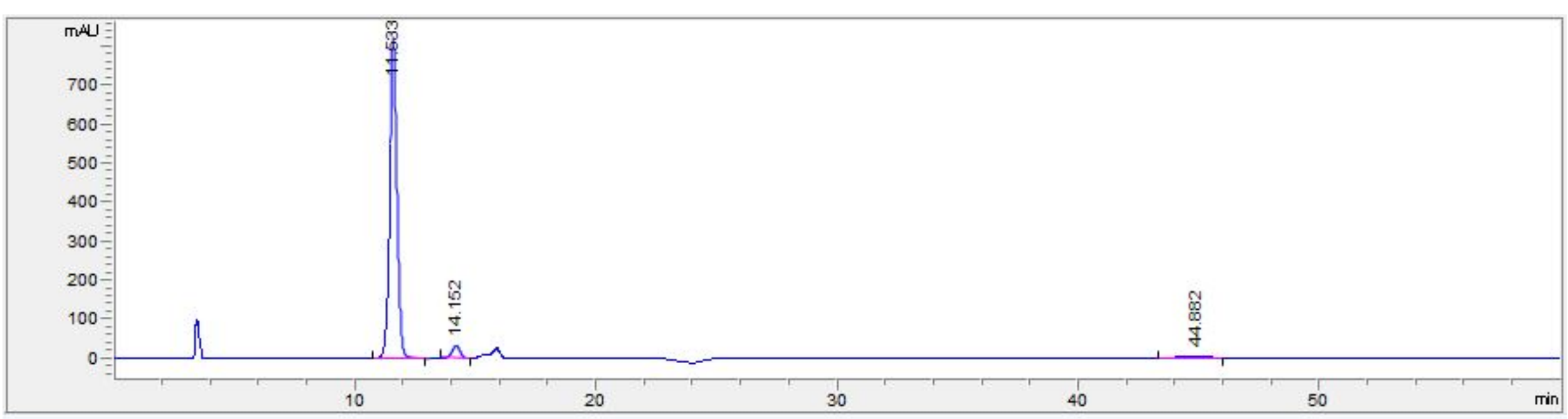


| # | Time | Area | Height | Width | Area% | Symmetry |
|---|---|---|---|---|---|---|
| 1 | 11.533 | 17163 | 822.3 | 0.3155 | 92.909 | 0.821 |
| 2 | 14.152 | 766.6 | 32.9 | 0.358 | 4.150 | 1.053 |
| 3 | 44.882 | 543.4 | 6.7 | 0.9566 | 2.942 | 1.376 |

**Perfluorophenyl (2*R*,3*R*)-2-fluoro-3-(4-methoxyphenyl)-2-phenylpent-4-enoate**

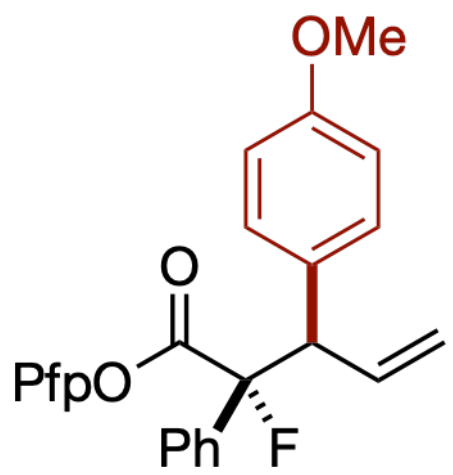


**((*R*,*R*)-20):** Prepared according to General Procedure B using pentafluorophenyl ester **1a** (32 mg, 0.1 mmol, 1 equiv.), *t*-butyl carbonate **2f** (39.6 mg, 0.15 mmol, 1.5 equiv.), ($R_a$,*R*,*R*)-**[Ir]** (4.2 mg, 4 mol%), (*S*)-Me-BTM (3.8 mg, 20 mol%) and DIPEA (22.6 μL, 0.13 mmol, 1.3 equiv.) in 1,4-dioxane (0.3 ml, 0.3 M). The diastereomeric ratio was determined to be 45:1. The *title compound* was obtained (35.3 mg, 0.076 mmol, 76%) as a colorless oil following purification by column chromatography [$SiO_2$, 0.25% Ethyl Acetate in Petroleum Ether]. The enantiomeric ratio (>99:1 *er*) of the corresponding primary amide was determined by chiral HPLC in comparison with the racemate. Diastereomeric ratio (98:2) was determined by $^{19}$F-NMR analysis of the crude reaction mixture.

**IR (film):** 2938 (w), 1782 (m), 1639 (w), 1612 (w), 1514 (s), 1469 (w), 1179 (s), 1008 (s), 996 (s), 747 (m) cm$^{-1}$.

**$^1$H NMR (500 MHz, CDCl$_3$):** δ 7.49 – 7.38 (m, 2H), 7.33 – 7.19 (m, 3H), 7.04 (dd, *J* = 8.7, 1.5 Hz, 2H), 6.68 (d, *J* = 8.7 Hz, 2H), 6.36 (ddd, *J* = 17.1, 10.3, 8.7 Hz, 1H), 5.37 (d, *J* = 17.0 Hz, 1H), 5.33 (d, *J* = 10.3 Hz, 1H), 4.36 (dd, *J* = 33.5, 9 Hz, 1H), 3.71 (s, 3H).

**$^{13}$C NMR (126 MHz, CDCl$_3$):** δ 166.6 (d, *J* = 28.4 Hz), 158.7, 135.2 (d, *J* = 22.4 Hz), 134.8 (d, *J* = 5.1 Hz), 130.6 (d, *J* = 2.4 Hz), 128.9, 128.6, 128.5 (d, *J* = 2.1 Hz), 125.0 (d, *J* = 9.9 Hz), 119.5, 113.7, 99.9 (d, *J* = 200.9 Hz), 56.7 (d, *J* = 19.1 Hz), 55.2.

**$^{19}$F NMR (376 MHz, CDCl$_3$):** δ -151.34 – -151.53 (m, 2F), -157.02 (t, *J* = 21.7 Hz, 1F), -161.75 – -162.02 (m, 2F), -174.77 (d, *J* = 33.1 Hz, 1F).

**HRMS (EI):** m/z calcd for $[M]^+$ $C_{24}H_{16}F_6O_3$: 466.1004. Found: 466.1012.

**(2*R*,3*R*)-2-Fluoro-3-(4-methoxyphenyl)-2-phenylpent-4-enamide ((*R*,*R*)-20-NH$_2$):**

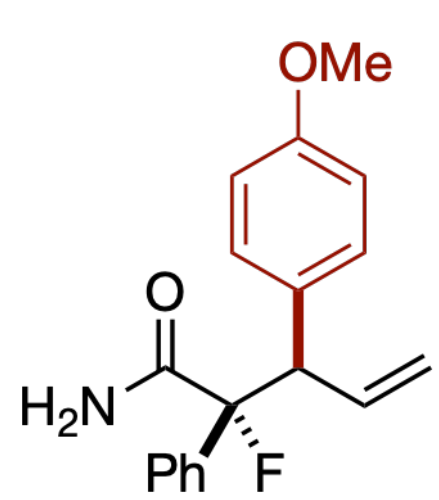

**IR (film):** 3433 (m), 3180 (br), 2930 (w), 1670 (s), 1610 (w), 1514 (m), 1448 (w), 1395 (w), 1252 (m) $cm^{-1}$.

**$^{1}$H NMR (500 MHz, $CDCl_3$):** δ 7.51 – 7.34 (m, 2H), 7.25 – 7.13 (m, 3H), 7.11 – 6.90 (m, 2H), 6.80 – 6.53 (m, 2H), 6.41 (s, 1H), 6.34 – 6.19 (m, 1H), 5.46 (s, 1H), 5.30 – 5.16 (m, 2H), 4.33 (dd, *J* = 35.0, 8.5 Hz, 1H), 3.70 (s, 3H).

**$^{13}$C NMR (126 MHz, $CDCl_3$):** δ 172.6 (d, *J* = 23.0 Hz), 158.4, 137.0 (d, *J* = 22.0 Hz), 135.5 (d, *J* = 5.4 Hz), 130.6 (d, *J* = 2.2 Hz), 129.8, 128.1, 128.0 (d, *J* = 2.1 Hz), 124.9 (d, *J* = 10.4 Hz), 118.9, 113.5, 101.7 (d, *J* = 196.7 Hz), 56.3 (d, *J* = 19.6 Hz), 55.2.

**$^{19}$F NMR (376 MHz, $CDCl_3$):** δ -171.63 (d, *J* = 35.2 Hz, 1F).

**HRMS (ESI):** m/z calcd for $[M+Na]^+$ $C_{18}H_{18}O_2NFNa$: 322.1214. Found: 322.1215.

HPLC analysis using a chiral column (Chiralpak IB 3μ column 22 ˚C, 1.0 ml/min, 95:5 hexane:isopropanol, 210 nm, $t_{major}$ = 13.091 min, $t_{minor}$ = 30.948 min, t = 15.295 min, t = 22.911 min).

(±)-**20:** (*rac*)-**[Ir]** and (*rac*)-Bn-BTM

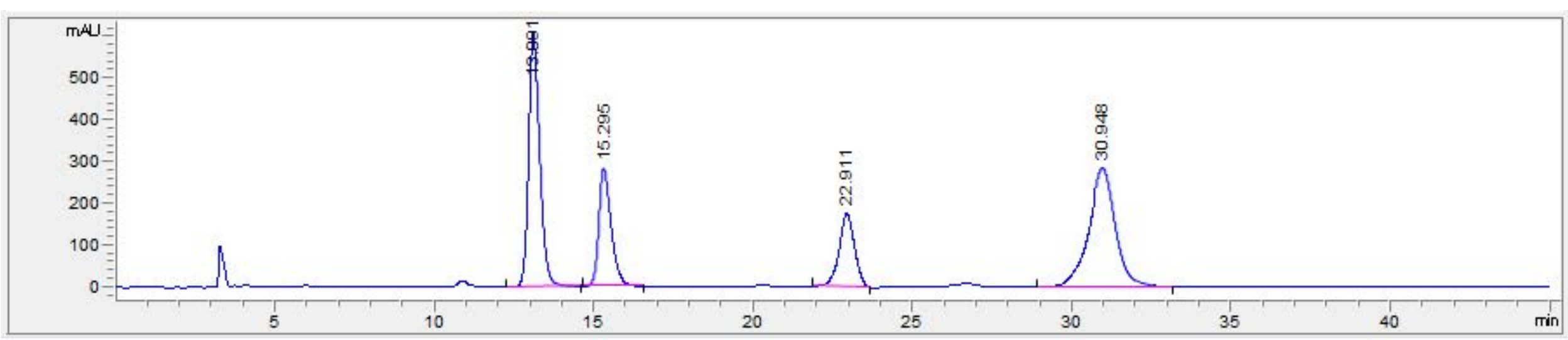


| # | Time | Area | Height | Width | Area% | Symmetry |
|---|---|---|---|---|---|---|
| 1 | 13.091 | 14285.7 | 605.4 | 0.3553 | 32.174 | 0.669 |
| 2 | 15.295 | 7638.9 | 282.7 | 0.4053 | 17.204 | 0.611 |
| 3 | 22.911 | 5829.5 | 175.6 | 0.5013 | 13.129 | 0.962 |
| 4 | 30.948 | 16646.9 | 284.5 | 0.8647 | 37.492 | 1.07 |

(*R*,*R*)-**20:** (*R*$_a$,*R*,*R*)-**[Ir]** and (*S*)-Me-BTM

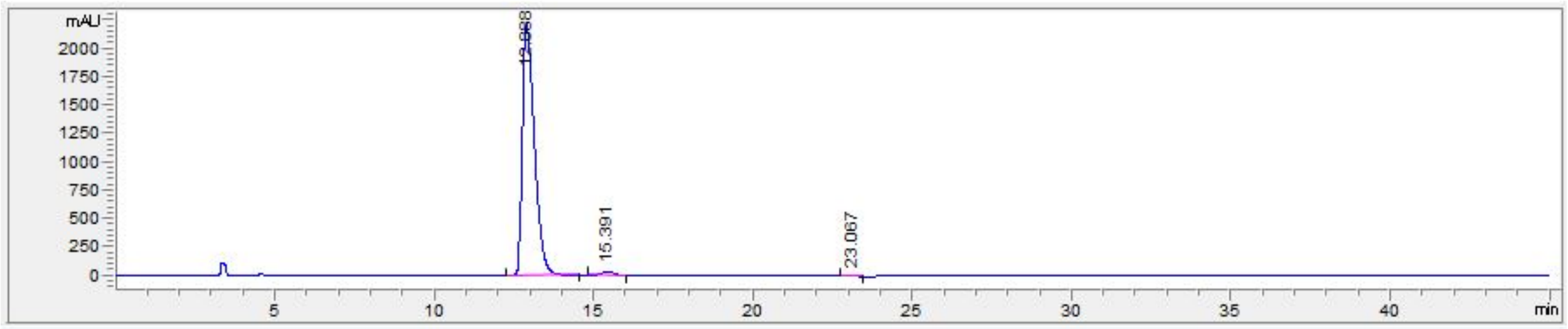


| # | Time | Area | Height | Width | Area% | Symmetry |
|---|---|---|---|---|---|---|
| 1 | 12.888 | 57394.1 | 2212 | 0.3927 | 98.124 | 0.48 |
| 2 | 15.391 | 995.9 | 38.4 | 0.3904 | 1.703 | 0.794 |
| 3 | 23.067 | 101.5 | 4.7 | 0.3385 | 0.173 | 0.77 |

**Perfluorophenyl (2*R*,3*S*)-2-fluoro-3-(4-methoxyphenyl)-2-phenylpent-4-enoate**

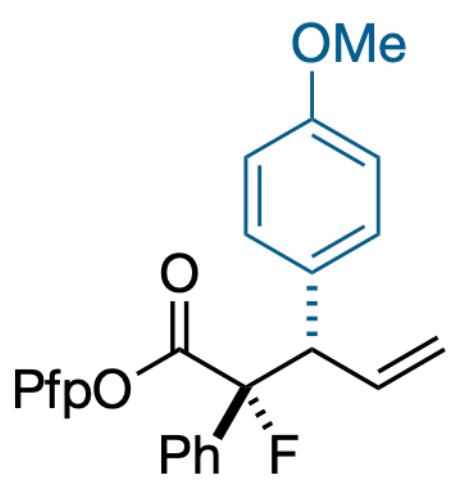


**((*R*,*S*)-20):** Prepared according to General Procedure B using pentafluorophenyl ester **1a** (32 mg, 0.1 mmol, 1 equiv.), *t*-butyl carbonate **2f** (39.6 mg, 0.15 mmol, 1.5 equiv.), (*S*$_a$,*S*,*S*)-**[Ir]** (4.2 mg, 4 mol%), (*S*)-Me-BTM (3.8 mg, 20 mol%) and DIPEA (22.6 µL, 0.13 mmol, 1.3 equiv.) in 1,4-dioxane (0.3 ml, 0.3 M). The diastereomeric ratio was determined to be 1:13. The *title compound* was obtained (33.4 mg, 0.072 mmol, 72%) as a white solid following purification by column chromatography [$SiO_2$, 0.25% Ethyl Acetate in Petroleum Ether]. The enantiomeric ratio (>99:1 *er*) of the corresponding primary amide was determined by chiral HPLC in comparison with the racemate. Diastereomeric ratio (93:7) was determined by $^{19}$F-NMR analysis of the crude reaction mixture.

**IR (film):** 2938 (w),1798 (w), 1781 (w), 1639 (w), 1612 (w), 1519 (s), 1468 (w), 1252 (m), 1182 (m), 1008 (s), 997 (s), 748 (m) cm$^{-1}$.

**$^1$H NMR (500 MHz, $CDCl_3$):** δ 7.69 (dt, *J* = 6.4, 1.3 Hz, 2H), 7.47 (dd, *J* = 8.4, 6.6 Hz, 2H), 7.42 (tt, *J* = 8.6, 1.4 Hz, 3H), 6.94 – 6.88 (m, 2H), 6.02 – 5.90 (m, 1H), **5.37 (d, *J* = 17.1 Hz)**, **5.33 (d, *J* = 10.4 Hz)**, 5.05 (dt, *J* = 10.3, 1.2 Hz, 1H), 4.91 (dt, *J* = 17.1, 1.4 Hz, 1H), 4.40 (dd, *J* = 34, 8 Hz, 1H), 3.83 (s, 3H), ***3.71 (s)**.

**$^{13}$C NMR (126 MHz, CDCl$_3$):** δ 166.1 (d, *J* = 28.9 Hz), 159.4, 135.8 (d, *J* = 22.6 Hz), 134.1 (d, *J* = 5.0 Hz), 130.7 (d, *J* = 3.2 Hz), 129.5, 129.3, 128.8 (d, *J* = 2.2 Hz), 125.4 (d, *J* = 10.4 Hz), 119.6, 114.3, 99.4 (d, *J* = 200.8 Hz), 56.1 (d, *J* = 19.0 Hz), 55.4.

**$^{19}$F NMR (376 MHz, CDCl$_3$):** δ -151.54 – -151.70 (m, 2F), -157.28 (t, *J* = 21.6 Hz, 1F), -161.94 – -162.19 (m, 2F), -173.74 (d, *J* = 33.9 Hz, 1F).

**HRMS (EI):** m/z calcd for $[M]^+$ $C_{24}H_{16}F_6O_3$: 466.1004. Found: 466.1003.

**(2*R*,3*S*)-2-fluoro-3-(4-methoxyphenyl)-2-phenylpent-4-enamide ((*R*,*S*)-20-NH$_2$):**

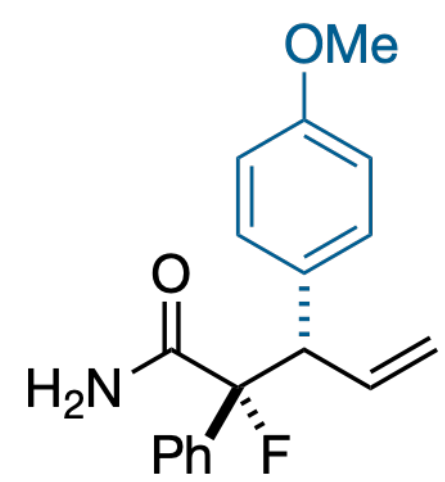


**IR (film):** 3437 (m), 3177 (br), 1666 (s), 1612 (w), 1515 (m), 1447 (w), 1399 (w), 1179 (m) cm$^{-1}$.

**$^{1}$H NMR (500 MHz, CDCl$_3$):** δ 7.74 – 7.60 (m, 2H), 7.49 – 7.37 (m, 2H), 7.37 – 7.30 (m, 3H), 6.94 – 6.81 (m, 2H), 6.05 (s, 1H), 5.92 (ddd, *J* = 17.1, 10.3, 8.1 Hz, 1H), ****5.28 – 5.19 (m)***, 5.07 (s, 1H), 4.97 (dt, *J* = 10.4, 1.3 Hz, 1H), 4.85 (dt, *J* = 17.1, 1.4 Hz, 1H), 4.35 (dd, *J* = 36, 8 Hz, 1H), 3.80 (s, 3H), ****3.70 (s)***.

**$^{13}$C NMR (126 MHz, CDCl$_3$):** δ 172.2 (d, *J* = 23.1 Hz), 158.9, 137.3 (d, *J* = 22.1 Hz), 134.7 (d, *J* = 4.5 Hz), 131.0 (d, *J* = 2.3 Hz), 130.4, 128.4 (d, *J* = 2.6 Hz), 125.1 (d, *J* = 11.0 Hz), 119.0, 113.8, 101.2 (d, *J* = 198.0 Hz), 55.7 (d, *J* = 19.1 Hz), 55.3.

**$^{19}$F NMR (376 MHz, CDCl$_3$):** δ -173.36 (d, *J* = 36.1 Hz).

**HRMS (ESI):** m/z calcd for $[M+Na]^+$ $C_{18}H_{18}O_2NFNa$: 322.1214. Found: 322.1215.

HPLC analysis using a chiral column (Chiralpak IB 3μ column 22 ˚C, 1.0 ml/min, 95:5 hexane:isopropanol, 210 nm, $t_{major}$ = 15.295 min, $t_{minor}$ = 22.911 min, t = 13.091 min, t = 30.948 min).

**(±)-20:** (*rac*)-**[Ir]** and (*rac*)-Bn-BTM

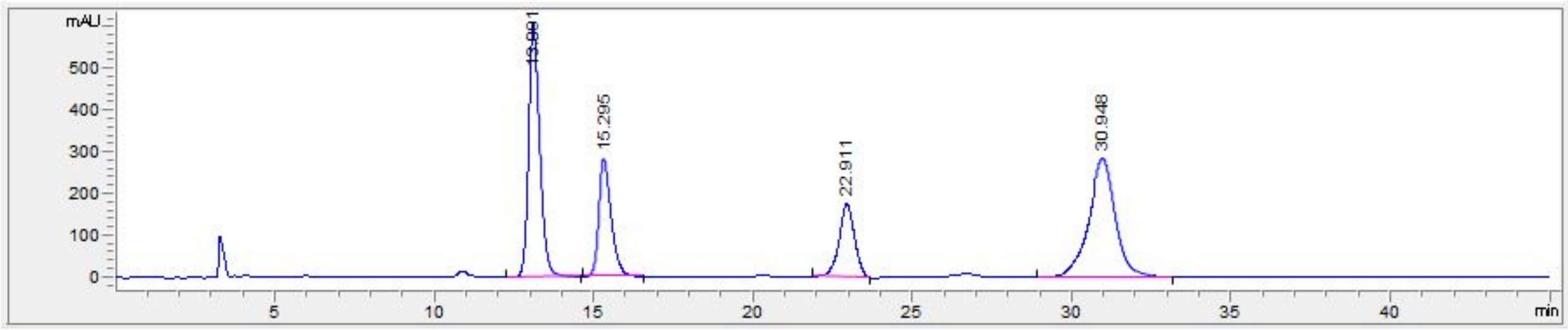


| # | Time | Area | Height | Width | Area% | Symmetry |
|---|---|---|---|---|---|---|
| 1 | 13.091 | 14285.7 | 605.4 | 0.3553 | 32.174 | 0.669 |
| 2 | 15.295 | 7638.9 | 282.7 | 0.4053 | 17.204 | 0.611 |
| 3 | 22.911 | 5829.5 | 175.6 | 0.5013 | 13.129 | 0.962 |
| 4 | 30.948 | 16646.9 | 284.5 | 0.8647 | 37.492 | 1.07 |

**(*R*,*S*)-20:** ($S_a$,*S*,*S*)-**[Ir]** and (*S*)-Me-BTM

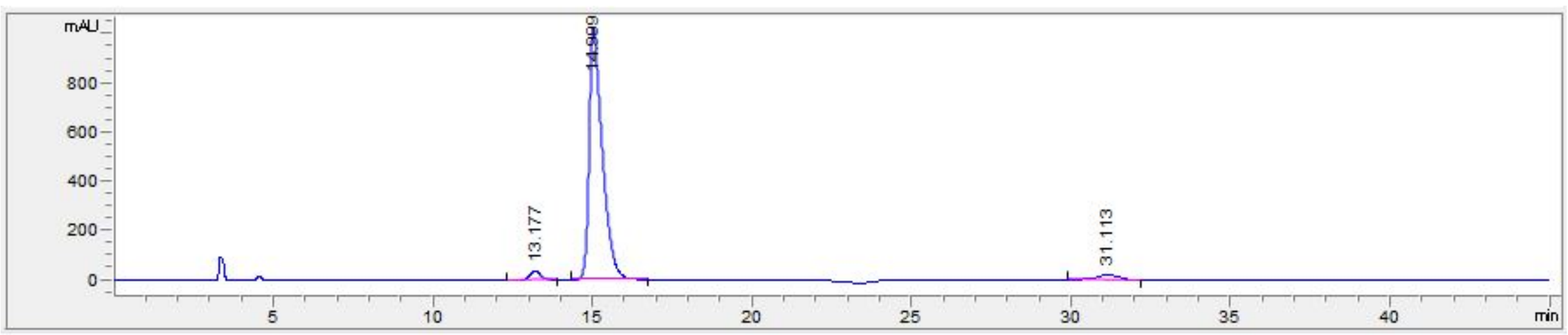


| # | Time | Area | Height | Width | Area% | Symmetry |
|---|---|---|---|---|---|---|
| 1 | 13.177 | 884.5 | 37.5 | 0.3591 | 2.823 | 0.918 |
| 2 | 14.999 | 29324.5 | 1018.2 | 0.42 | 93.585 | 0.427 |
| 3 | 31.113 | 1125.5 | 21.2 | 0.7509 | 3.592 | 1.098 |

**Perfluorophenyl (2*R*,3*R*)-2-fluoro-3-(4-nitrophenyl)-2-phenylpent-4-enoate ((*R*,*R*)-21):** Prepared according to General Procedure B using pentafluorophenyl ester **1a** (32 mg, 0.1 mmol, 1 equiv.), *t*-butyl carbonate **2g** (42 mg, 0.15 mmol, 1.5 equiv.), ($R_a$,*R*,*R*)-**[Ir]** (4.2 mg, 4 mol%), (*S*)-Me-BTM (3.8 mg, 20 mol%) and DIPEA (22.6 μL, 0.13 mmol, 1.3 equiv.) in 1,4-dioxane (0.3 ml, 0.3 M). The diastereomeric ratio was determined to be 31:1. The *title compound* was obtained (31.7 mg, 0.066 mmol, 66%) as a colorless oil following purification by column chromatography [$SiO_2$, 0.25% Ethyl Acetate in Petroleum Ether]. The enantiomeric ratio (>99:1 *er*) of the corresponding primary amide was determined by chiral HPLC in comparison with the racemate. Diastereomeric ratio (97:3) was determined by $^{19}$F-NMR analysis of the crude reaction mixture.

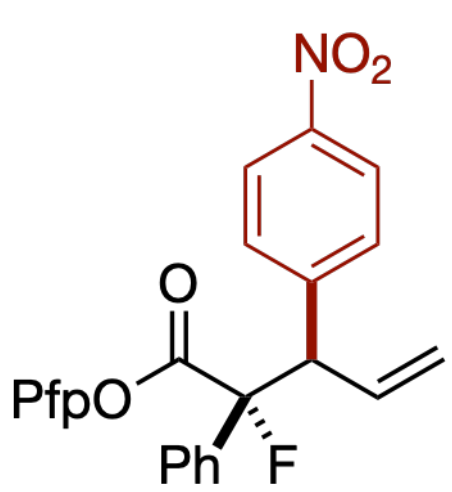

**IR (film):** 3083 (w), 1788 (w), 1640 (w), 1607 (w), 1519 (s), 1347 (m), 1182 (m), 1011 (m), 997 (m) $cm^{-1}$.

**$^1$H NMR (500 MHz, $CDCl_3$):** δ 8.01 (d, *J* = 8.8 Hz, 2H), 7.48 – 7.38 (m, 2H), 7.33 – 7.27 (m, 5H), 6.35 (ddd, *J* = 17.2, 10.1, 8.7 Hz, 1H), 5.49 – 5.40 (m, 2H), 4.55 (dd, *J* = 32.5, 9 Hz, 1H).

**$^{13}$C NMR (126 MHz, $CDCl_3$):** δ 165.9 (d, *J* = 27.9 Hz), 147.2, 144.2, 134.4 (d, *J* = 22.5 Hz), 133.1 (d, *J* = 5.1 Hz), 130.5 (d, *J* = 2.7 Hz), 129.5, 128.8 (d, *J* = 2.2 Hz), 124.7 (d, *J* = 10.1 Hz), 123.5, 121.4, 99.4 (d, *J* = 202.5 Hz), 57.3 (d, *J* = 19.1 Hz).

**$^{19}$F NMR (376 MHz, $CDCl_3$):** δ -151.51 – -151.67 (m, 2F), -156.50 (t, *J* = 21.7 Hz, 1F), -161.43 – -161.70 (m, 2F), -175.23 (d, *J* = 32.3 Hz, 1F).

**HRMS (EI):** m/z calcd for $[M]^+$ $C_{23}H_{13}F_6NO_4$: 481.0749. Found: 481.0753.

**(2*R*,3*R*)-2-Fluoro-3-(4-nitrophenyl)-2-phenylpent-4-enamide ((*R*,*R*)-21-$NH_2$):**

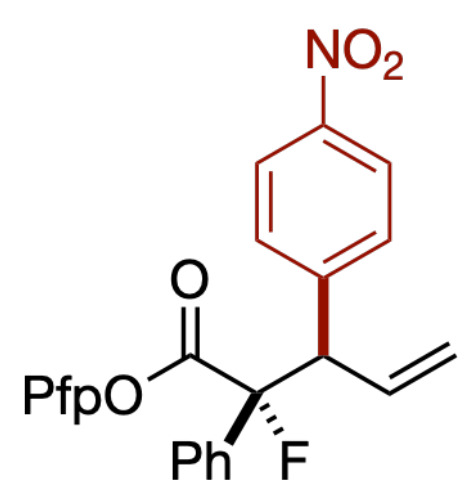


**IR (film):** 3487 (w), 3322 (br), 3083 (w), 1693 (m), 1604 (m), 1518 (s), 1495 (w), 1389 (w), 1346 (s) $cm^{-1}$.

**$^1$H NMR (500 MHz, $CDCl_3$):** δ 8.01 – 7.93 (m, 2H), 7.44 – 7.35 (m, 2H), 7.25 (d, *J* = 7.9 Hz, 2H), 7.19 (dd, *J* = 5.0, 2.6 Hz, 3H), 6.42 (s, 1H), 6.25 (ddd, *J* = 17.1, 10.4, 8.2 Hz, 1H), 5.53 (s, 1H), 5.37 – 5.21 (m, 2H), 4.55 (dd, *J* = 34.5, 8.5 Hz, 1H).

**$^{13}$C NMR (126 MHz, $CDCl_3$):** δ 171.7 (d, *J* = 22.7 Hz), 146.9, 145.5, 136.1 (d, *J* = 22.0 Hz), 133.8 (d, *J* = 5.4 Hz), 130.5 (d, *J* = 2.5 Hz), 128.6, 128.4 (d, *J* = 2.3 Hz), 124.5 (d, *J* = 10.6 Hz), 123.3, 120.7, 101.1 (d, *J* = 198.5 Hz), 56.8 (d, *J* = 19.3 Hz).

**$^{19}$F NMR (376 MHz, $CDCl_3$):** δ -172.75 (d, *J* = 34.0 Hz, 1F).

**HRMS (EI):** m/z calcd for $[M]^+$ $C_{17}H_{15}FN_2O_3$: 314.1067. Found: 314.1074.

HPLC analysis using a chiral column (Chiralpak IB 3μ column 22 ˚C, 1.0 ml/min, 93:7 hexane:isopropanol, 210 nm, $t_{major}$ = 19.577 min, $t_{minor}$ = 59.994 min, t = 15.945 min, t = 27.643 min).

(±)-**21:** (*rac*)-**[Ir]** and (*rac*)-Bn-BTM

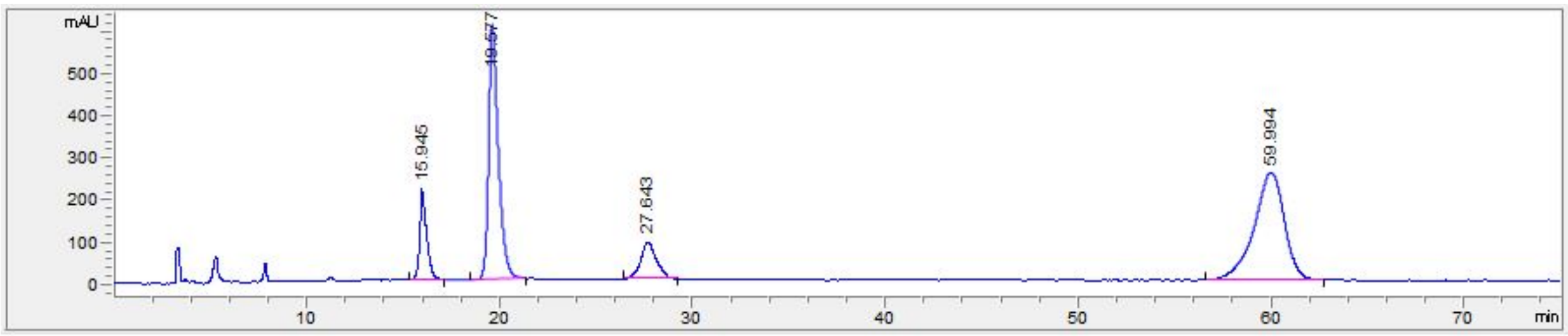


| # | Time | Area | Height | Width | Area% | Symmetry |
|---|---|---|---|---|---|---|
| 1 | 15.945 | 6077.6 | 214.5 | 0.4246 | 10.192 | 0.585 |
| 2 | 19.577 | 21052.3 | 601.1 | 0.5106 | 35.305 | 0.53 |
| 3 | 27.643 | 4824.8 | 87 | 0.8066 | 8.091 | 0.788 |
| 4 | 59.994 | 27675.1 | 252.1 | 1.571 | 46.412 | 1.255 |

(*R*,*R*)-**21:** (*R*$_a$*,R,R*)-**[Ir]** and (*S*)-Me-BTM

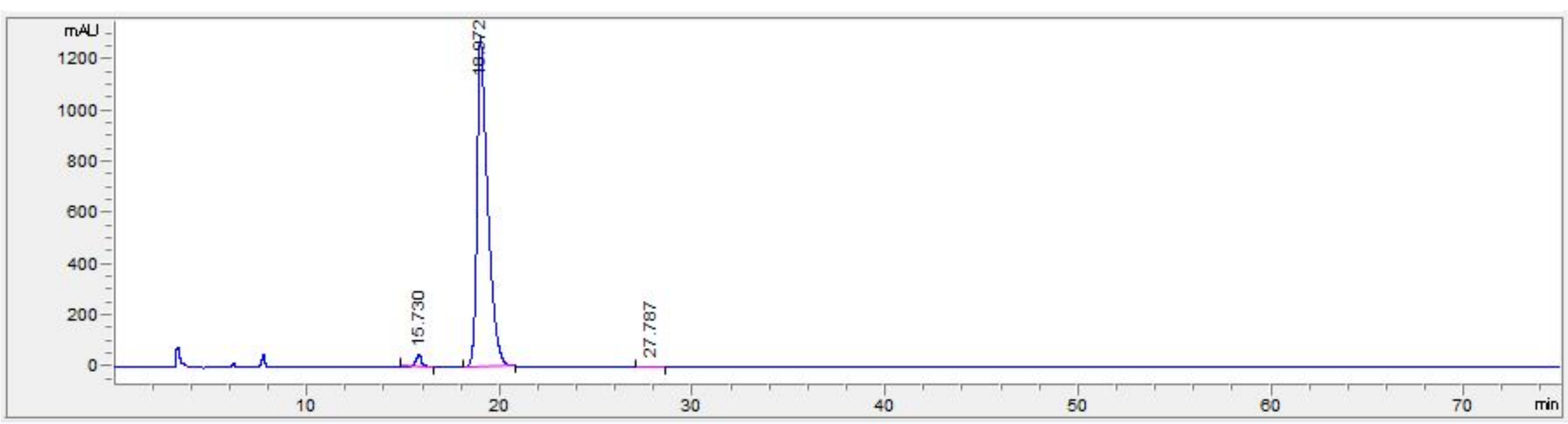


| # | Time | Area | Height | Width | Area% | Symmetry |
|---|---|---|---|---|---|---|
| 1 | 15.73 | 1167.5 | 45.6 | 0.3889 | 2.405 | 0.786 |
| 2 | 18.972 | 47227.3 | 1283.3 | 0.5234 | 97.269 | 0.44 |
| 3 | 27.787 | 158.3 | 3.4 | 0.5887 | 0.326 | 1.103 |

**Perfluorophenyl (2*R*,3*S*)-2-fluoro-3-(4-nitrophenyl)-2-phenylpent-4-enoate ((*R*,*S*)-21):**

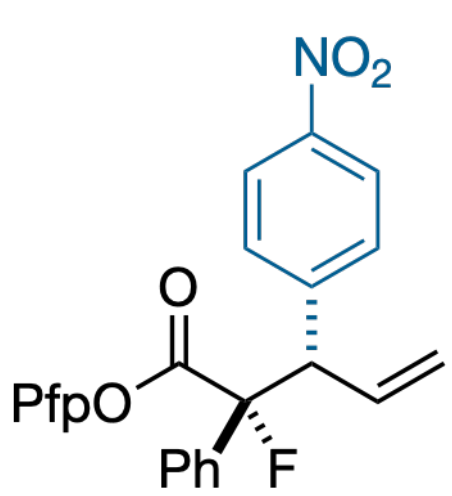


Prepared according to General Procedure B using pentafluorophenyl ester **1a** (32 mg, 0.1 mmol, 1 equiv.), *t*-butyl carbonate **2g** (42 mg, 0.15 mmol, 1.5 equiv.), (*S*$_a$*,S,S*)-**[Ir]** (4.2 mg, 4 mol%), (*S*)-Me-BTM (3.8 mg, 20 mol%) and DIPEA (22.6 μL, 0.13 mmol, 1.3 equiv.) in 1,4-dioxane (0.3 ml, 0.3 M). The diastereomeric ratio was determined to be 1:23. The *title compound* was obtained (30.2 mg, 0.063 mmol, 63%) as a colorless oil following purification by column chromatography [$SiO_2$, 0.25% Ethyl Acetate in Petroleum Ether]. The enantiomeric ratio (>99:1 *er*) of the corresponding

primary amide was determined by chiral HPLC in comparison with the racemate. Diastereomeric ratio (91:9) was determined by $^{19}$F-NMR analysis of the crude reaction mixture.

**IR (film):** 3086 (m), 1798 (w), 1782 (w), 1654 (w), 1606 (w), 1518 (s), 1347 (s), 1198 (m), 1009 (s), 997 (s) cm$^{-1}$.

**$^{1}$H NMR (500 MHz, CDCl$_3$):** δ 8.36 – 8.17 (m, 2H), 7.68 (dt, *J* = 8.1, 1.5 Hz, 4H), 7.55 – 7.43 (m, 3H), ***6.35 (ddd, J = 17.3, 10.2, 8.7 Hz)**, 5.99 – 5.90 (m, 1H), ***5.53 – 5.37 (m)**, 5.14 (d, *J* = 10.3 Hz, 1H), 4.93 (d, *J* = 17.1 Hz, 1H), 4.54 (dd, *J* = 32.5, 7.5 Hz, 1H).

**$^{13}$C NMR (126 MHz, CDCl$_3$):** δ 165.7 (d, *J* = 28.3 Hz), 147.7, 145.1, 134.8 (d, *J* = 22.5 Hz), 132.6 (d, *J* = 4.7 Hz), 130.6 (d, *J* = 3.5 Hz), 129.7, 129.1 (d, *J* = 2.1 Hz), 125.1 (d, *J* = 10.2 Hz), 124.0, 121.2, 98.8 (d, *J* = 202.3 Hz), 56.7 (d, *J* = 19.0 Hz).

**$^{19}$F NMR (376 MHz, CDCl$_3$):** δ -152.19 – -152.38 (m, 2F), -156.44 (t, *J* = 21.8 Hz, 1F), -161.30 – -161.51 (m, 2F), -173.65 (d, *J* = 32.7 Hz, 1F).

**HRMS (EI):** m/z calcd for $[M]^+$ $C_{23}H_{13}F_6NO_4$: 481.0749. Found: 481.0760.

**(2*R*,3*S*)-2-Fluoro-3-(4-nitrophenyl)-2-phenylpent-4-enamide ((*R*,*S*)-21-NH2):**

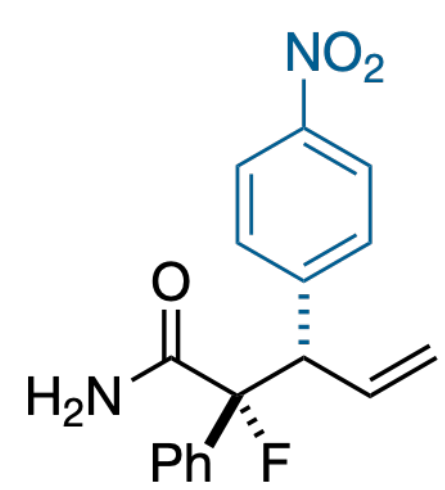


**IR (film):** 3442 (m), 3222 (br), 1671 (s), 1599 (m), 1518 (s), 1448 (w), 1396 (w), 1350 (s) cm$^{-1}$.

**$^{1}$H NMR (500 MHz, CDCl$_3$):** δ 8.26 – 8.12 (m, 2H), 7.68 – 7.63 (m, 2H), 7.63 – 7.58 (m, 2H), 7.46 – 7.39 (m, 2H), 7.39 – 7.35 (m, 1H), ***6.42 (s)**, ***6.24 (ddd, J = 17.7, 10.3, 8.2 Hz)**, 6.08 (s, 1H), 5.92 (ddd, *J* = 17.1, 10.3, 8.2 Hz, 1H), ***5.52 (s)**, ***5.36 – 5.23 (m)**, 5.12 (s, 1H), 5.06 (dt, *J* = 10.3, 1.1 Hz, 1H), 4.87 (d, *J* = 17.1 Hz, 1H), 4.51 (dd, *J* = 35.0, 8.5 Hz, 1H).

**$^{13}$C NMR (126 MHz, CDCl$_3$):** δ 171.4 (d, *J* = 23.0 Hz), 147.4, 146.1, 136.5 (d, *J* = 21.8 Hz), 133.2 (d, *J* = 4.3 Hz), 131.0 (d, *J* = 2.6 Hz), 128.8, 128.6 (d, *J* = 2.3 Hz), 124.9 (d, *J* = 11.0 Hz), 123.6, 120.6, 100.8 (d, *J* = 199.4 Hz), 56.2 (d, *J* = 19.1 Hz).

**$^{19}$F NMR (376 MHz, CDCl$_3$):** δ -173.23 (d, *J* = 35.2 Hz, 1F).

**HRMS (ESI):** m/z calcd for [M+Na]$^+$ $C_{17}H_{15}O_3N_2FNa$: 337.0959. Found: 337.0960.

HPLC analysis using a chiral column (Chiralpak IB 3μ column 22 ˚C, 1.0 ml/min, 93:7 hexane:isopropanol, 210 nm, $t_{major}$ = 15.945 min, $t_{minor}$ = 27.643 min, t = 19.577 min, t = 59.994 min).

(±)-**21:** (*rac*)-**[Ir]** and (*rac*)-Bn-BTM

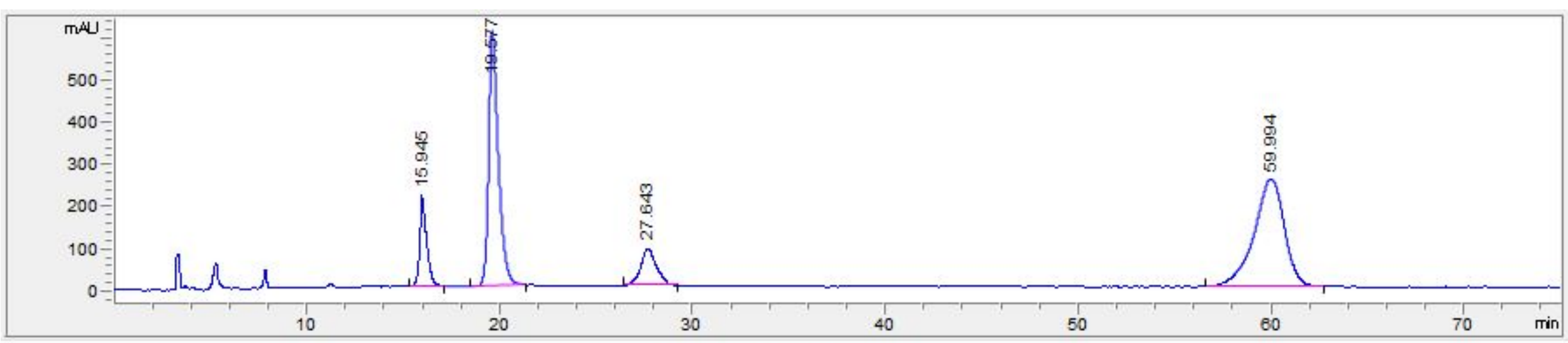


| # | Time | Area | Height | Width | Area% | Symmetry |
|---|---|---|---|---|---|---|
| 1 | 15.945 | 6077.6 | 214.5 | 0.4246 | 10.192 | 0.585 |
| 2 | 19.577 | 21052.3 | 601.1 | 0.5106 | 35.305 | 0.53 |
| 3 | 27.643 | 4824.8 | 87 | 0.8066 | 8.091 | 0.788 |
| 4 | 59.994 | 27675.1 | 252.1 | 1.571 | 46.412 | 1.255 |

(*R*,*S*)-**21:** (*S*$_a$,*S*,*S*)-**[Ir]** and (*S*)-Me-BTM

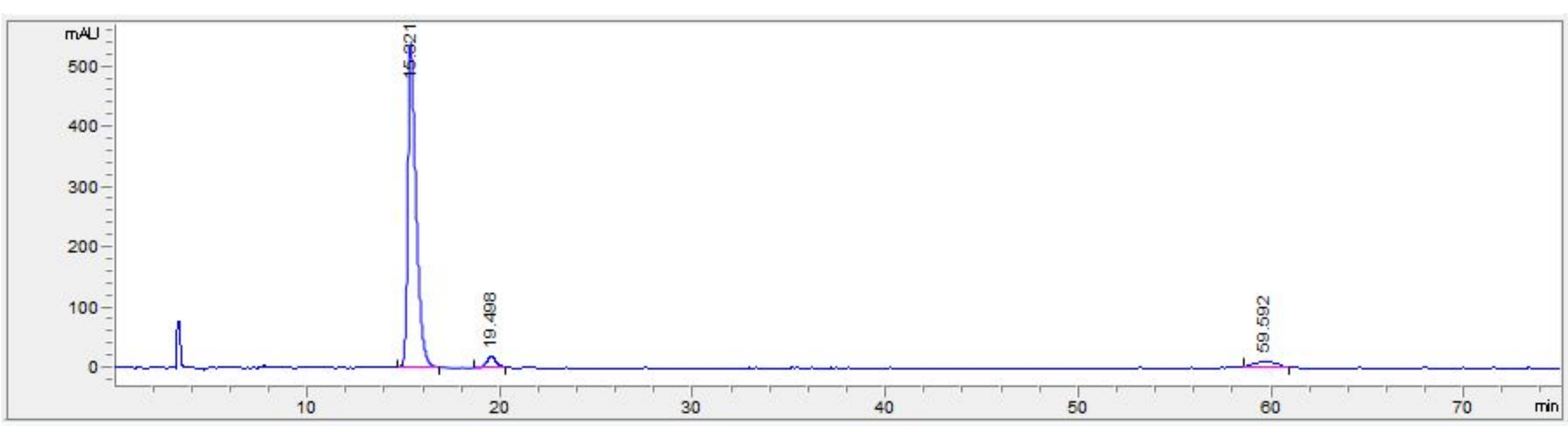


| # | Time | Area | Height | Width | Area% | Symmetry |
|---|---|---|---|---|---|---|
| 1 | 15.321 | 16028.8 | 542.1 | 0.433 | 91.543 | 0.461 |
| 2 | 19.498 | 625.6 | 18.5 | 0.4979 | 3.573 | 0.937 |
| 3 | 59.592 | 855.2 | 10.8 | 0.9686 | 4.884 | 0.862 |

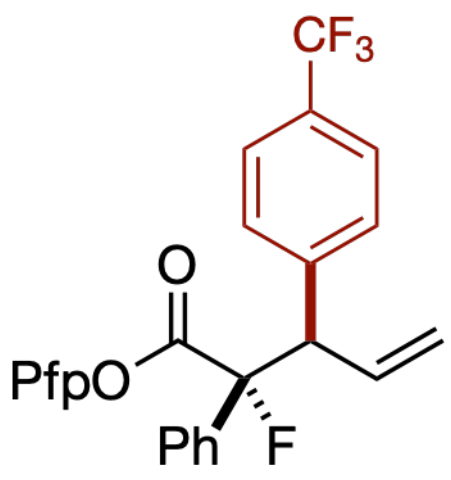


**Perfluorophenyl (2*R*,3*R*)-2-fluoro-2-phenyl-3-(4-(trifluoromethyl)phenyl)pent-4-enoate ((*R*,*R*)-22):** Prepared according to General Procedure B using pentafluorophenyl ester **1a** (32 mg, 0.1 mmol, 1 equiv.), *t*-butyl carbonate **2h** (45.3 mg, 0.15 mmol, 1.5 equiv.), ($R_a$,*R*,*R*)-**[Ir]** (4.2 mg, 4 mol%), (*S*)-Me-BTM (3.8 mg, 20 mol%) and DIPEA (22.6 µL, 0.13 mmol, 1.3 equiv.) in 1,4-dioxane (0.3 ml, 0.3 M). The diastereomeric ratio was determined to be 34.5:1. The *title compound* was obtained (37.1 mg, 0.0735 mmol, 73.5%) as a colorless oil following purification by column chromatography [$SiO_2$, 0.25% Ethyl Acetate in Petroleum Ether]. The enantiomeric ratio (>99:1 *er*) of the corresponding primary amide was determined by chiral HPLC in comparison with the racemate. Diastereomeric ratio (93:7) was determined by $^{19}$F-NMR analysis of the crude reaction mixture.

**IR (film):** 3067 (w), 1784 (w), 1640 (w), 1619 (w), 1519 (s), 1472 (w), 1326 (s), 1126 (s), 1010 (s), 997 (s) cm$^{-1}$.

**$^{1}$H NMR (500 MHz, $CDCl_3$):** δ 7.41 (td, *J* = 5.7, 3.4 Hz, 4H), 7.31 – 7.20 (m, 5H), 6.35 (ddd, *J* = 17.1, 10.2, 8.7 Hz, 1H), 5.46 – 5.35 (m, 2H), 4.48 (dd, *J* = 32.5, 8.5 Hz, 1H).

**$^{13}$C NMR (126 MHz, $CDCl_3$):** δ 166.2 (d, *J* = 28.0 Hz), 140.7, 134.7 (d, *J* = 22.5 Hz), 133.7 (d, *J* = 5.0 Hz), 130.0 (d, *J* = 2.5 Hz), 129.6 (q, *J* = 32.6 Hz), 129.3, 128.7 (d, *J* = 2.1 Hz), 125.3 (q, *J* = 3.7 Hz), 124.8 (d, *J* = 10.0 Hz), 124.1 (q, *J* = 272.1 Hz), 120.8, 99.5 (d, *J* = 201.9 Hz), 57.2 (d, *J* = 19.1 Hz).

**$^{19}$F NMR (376 MHz, $CDCl_3$):** δ -62.73 (s, 3F), -151.50 – -151.64 (m, 2F), -156.72 (t, *J* = 21.7 Hz, 1F), -161.61 – -161.81 (m, 2F), -174.88 (d, *J* = 32.6 Hz, 1F).

**HRMS (EI):** m/z calcd for $[M]^+$ $C_{24}H_{13}F_9O_2$: 504.0772. Found: 504.0779.

**(2*R*,3*R*)-2-Fluoro-2-phenyl-3-(4-(trifluoromethyl)phenyl)pent-4-enamide ((*R*,*R*)-22-NH$_2$):**

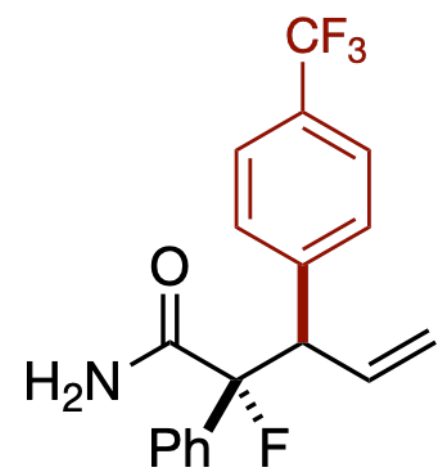


**IR (film):** 3432 (m), 3183 (br), 1671 (s), 1618 (w), 1495 (w), 1449 (w), 1389 (w), 1328 (s), 1130 (s) cm$^{-1}$.

**$^1$H NMR (500 MHz, CDCl$_3$):** δ 7.46 – 7.32 (m, 4H), 7.23 – 7.14 (m, 5H), 6.42 (s, 1H), 6.26 (ddd, *J* = 17.0, 10.3, 8.3 Hz, 1H), 5.52 (s, 1H), 5.41 – 5.17 (m, 2H), 4.47 (dd, *J* = 34.5, 8.5 Hz, 1H).

**$^{13}$C NMR (126 MHz, CDCl$_3$):** δ 172.0 (d, *J* = 22.8 Hz), 141.9, 136.4 (d, *J* = 21.9 Hz), 134.4 (d, *J* = 5.3 Hz), 130.0 (d, *J* = 2.3 Hz), 129.2 (q, *J* = 32.2 Hz), 128.4, 128.2 (d, *J* = 2.1 Hz), 125.0 (q, *J* = 3.8 Hz), 124.7 (d, *J* = 10.5 Hz), 124.2 (d, *J* = 272.1 Hz), 120.1, 101.3 (d, *J* = 198.0 Hz), 56.8 (d, *J* = 19.5 Hz).

**$^{19}$F NMR (376 MHz, CDCl$_3$):** δ -62.64 (s, 3F), -172.09 (d, *J* = 34.6 Hz, 1F).

**HRMS (ESI):** m/z calcd for $[M+Na]^+$ $C_{18}H_{15}ONF_4Na$: 360.0982. Found: 360.0982.

HPLC analysis using a chiral column (Chiralpak IB 3μ column 22 ˚C, 1.0 ml/min, 95:5 hexane:isopropanol, 210 nm, $t_{major}$ = 14.680 min, $t_{minor}$ = 40.069 min, t = 9.532 min, t = 24.513 min).

**(±)22:** (*rac*)-**[Ir]** and (*rac*)-Bn-BTM

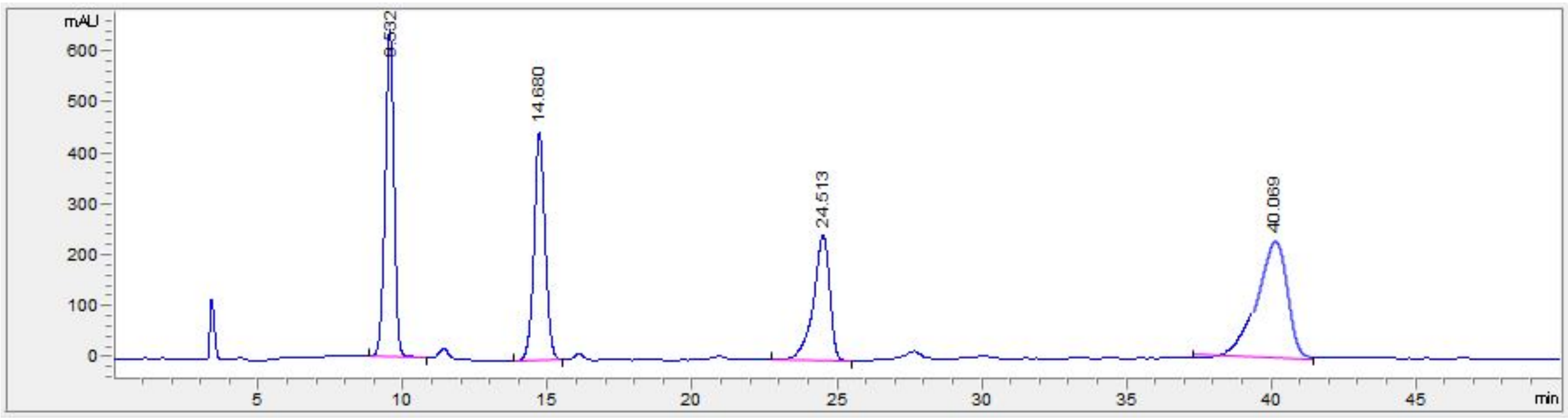

| # | Time | Area | Height | Width | Area% | Symmetry |
|---|---|---|---|---|---|---|
| 1 | 9.532 | 13625.2 | 644.2 | 0.3207 | 26.191 | 1.077 |
| 2 | 14.68 | 11824.2 | 448.5 | 0.3996 | 22.729 | 0.89 |
| 3 | 24.513 | 9619.9 | 245.5 | 0.5932 | 18.492 | 1.468 |
| 4 | 40.069 | 16952.8 | 231.5 | 1.2205 | 32.588 | 1.409 |

***(R,R)*-22:** ($R_a$,*R,R*)-**[Ir]** and (*S*)-Me-BTM

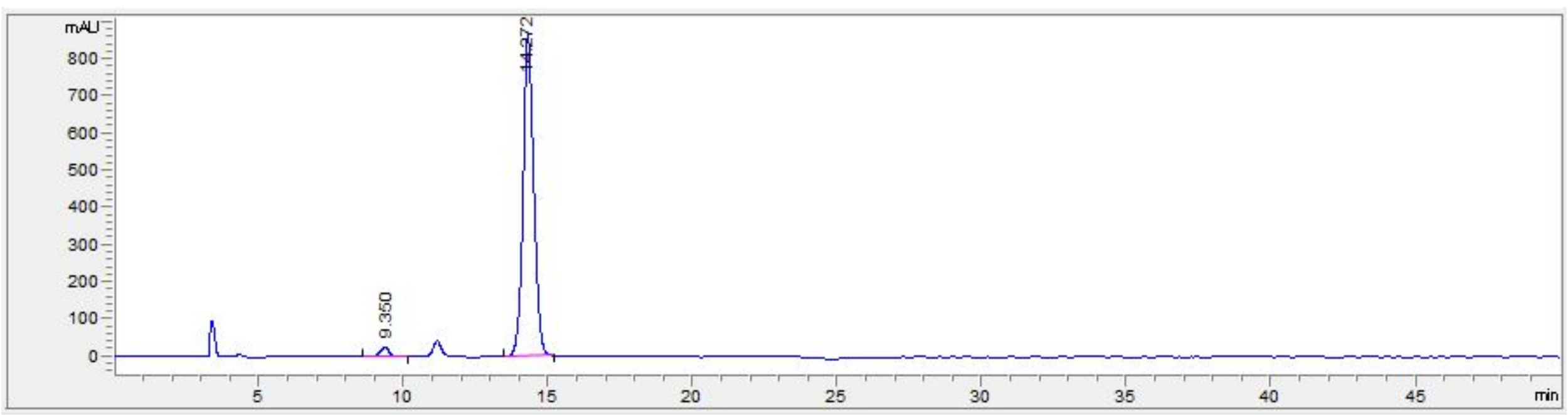

| # | Time | Area | Height | Width | Area% | Symmetry |
|---|---|---|---|---|---|---|
| 1 | 9.35 | 560.2 | 25.9 | 0.3244 | 2.469 | 1.264 |
| 2 | 14.272 | 22128.9 | 866.4 | 0.384 | 97.531 | 0.779 |

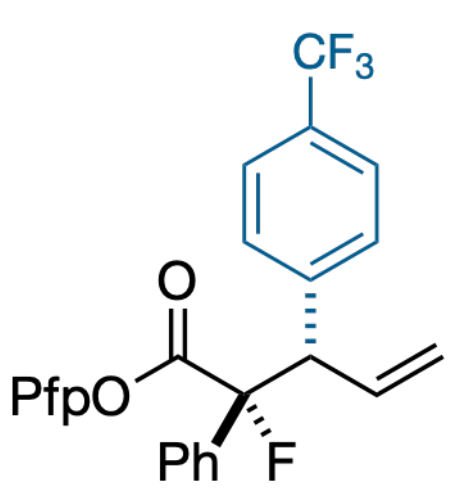

**Perfluorophenyl (2*R*,3*S*)-2-fluoro-2-phenyl-3-(4-(trifluoromethyl)phenyl)pent-4-enoate ((*R*,*S*)-22):** Prepared according to General Procedure B using pentafluorophenyl ester **1a** (32 mg, 0.1 mmol, 1 equiv.), *t*-butyl carbonate **2h** (45.3 mg, 0.15 mmol, 1.5 equiv.), ($S_a$,*S,S*)-**[Ir]** (4.2 mg, 4 mol%), (*S*)-Me-BTM (3.8 mg, 20 mol%) and DIPEA (22.6 μL, 0.13 mmol, 1.3 equiv.) in 1,4-dioxane (0.3 ml, 0.3 M). The diastereomeric ratio was determined to be 1:15. The *title compound* was obtained (36 mg, 0.071 mmol, 71%) as a white solid following purification by column chromatography [$SiO_2$, 0.25% Ethyl Acetate in Petroleum Ether]. The enantiomeric ratio (>99:1 *er*) of the corresponding primary amide was determined by chiral HPLC in comparison with the

racemate. Diastereomeric ratio (94:6) was determined by $^{19}$F-NMR analysis of the crude reaction mixture.

**IR (film):** 3069 (w), 1781 (w), 1640 (w), 1620 (w), 1519 (s), 1326 (s), 1126 (s), 1009 (s), 998 (s) m$^{-1}$.

**$^{1}$H NMR (500 MHz, CDCl$_3$):** δ 7.69 (dd, *J* = 7.5, 1.9 Hz, 2H), 7.63 (q, *J* = 8.4 Hz, 4H), 7.56 – 7.42 (m, 3H), ***6.36 (ddd, J = 17.0, 10.4, 8.9 Hz)***, 5.96 (ddd, *J* = 17.6, 10.3, 7.8 Hz, 1H), ***5.47 – 5.36 (m)***, 5.11 (d, *J* = 10.3 Hz, 1H), 4.92 (d, *J* = 17.1 Hz, 1H), 4.49 (dd, *J* = 33.0, 8.0 Hz, 1H).

**$^{13}$C NMR (126 MHz, CDCl$_3$):** δ 165.9 (d, *J* = 28.5 Hz), 141.6, 135.2 (d, *J* = 22.8 Hz), 133.1 (d, *J* = 4.9 Hz), 130.3 (q, *J* = 32.6 Hz), 130.0 (d, *J* = 3.3 Hz), 129.6, 129.0 (d, *J* = 2.1 Hz), 125.8 (q, *J* = 3.8 Hz), 125.2 (d, *J* = 10.4 Hz), 124.2 (q, *J* = 272.1 Hz), 120.7, 99.0 (d, *J* = 202.2 Hz), 56.8 (d, *J* = 18.9 Hz).

**$^{19}$F NMR (376 MHz, CDCl$_3$):** δ -62.78 (s, 3F), -152.11 – -152.31 (m, 2F), -156.81 (t, *J* = 21.6 Hz, 1F), -161.63 – -161.94 (m, 2F), -173.74 (d, *J* = 33.1 Hz, 1F).

**HRMS (EI):** m/z calcd for [M]$^+$ $C_{24}H_{13}F_9O_2$: 504.0772. Found: 504.0779.

**(2*R*,3*S*)-2-fluoro-2-phenyl-3-(4-(trifluoromethyl)phenyl)pent-4-enamide ((*R*,*S*)-22-NH$_2$):**

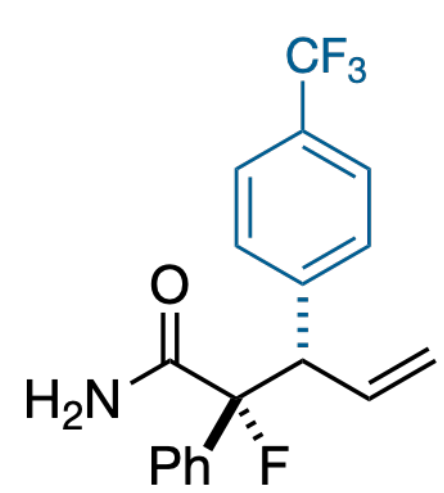


**IR (film):** 3444 (m), 3227 (br), 1672 (s), 1619 (w), 1495 (w), 1448 (w), 1394 (w), 1336 (s), 1125 (s) cm$^{-1}$.

**$^{1}$H NMR (500 MHz, CDCl$_3$):** δ 7.66 (dt, *J* = 6.6, 1.3 Hz, 2H), 7.59 (d, *J* = 8.3 Hz, 2H), 7.55 (d, *J* = 8.2 Hz, 2H), 7.47 – 7.39 (m, 2H), 7.39 – 7.33 (m, 1H), 6.06 (s, 1H), 5.93 (ddd, *J* = 17.1, 10.4, 8.2 Hz, 1H), ***5.33 – 5.22 (m)***, 5.09 (s, 1H), 5.03 (dt, *J* = 10.4, 1.1 Hz, 1H), 4.86 (dt, *J* = 17.1, 1.5 Hz, 1H), 4.46 (dd, *J* = 36.0, 8.5 Hz, 1H).

**$^{13}$C NMR (126 MHz, CDCl$_3$):** δ 171.6 (d, *J* = 23.2 Hz), 142.6, 136.8 (d, *J* = 22.0 Hz), 133.7 (d, *J* = 4.4 Hz), 130.4 (d, *J* = 2.5 Hz), 129.6 (q, *J* = 32.2 Hz), 128.7, 128.5 (d, *J* = 2.2 Hz), 125.3 (q, *J* = 4.2 Hz), 125.0 (d, *J* = 11.0 Hz), 124.3 (q, *J* = 272.6 Hz), 120.1, 100.9 (d, *J* = 199.1 Hz), 56.3 (d, *J* = 18.9 Hz).

**$^{19}$F NMR (376 MHz, CDCl$_3$):** δ -62.58 (s, 3F), -173.31 (d, *J* = 35.5 Hz, 1F).

**HRMS (ESI):** m/z calcd for $[M+Na]^+$ $C_{18}H_{15}ONF_4Na$: 360.0982. Found: 360.0982.

HPLC analysis using a chiral column (Chiralpak IB 3μ column 22 ˚C, 1.0 ml/min, 95:5 hexane:isopropanol, 210 nm, $t_{major}$ = 9.532 min, $t_{minor}$ = 24.513 min, t = 14.680 min, t = 40.069 min).

(±)-**22:** (*rac*)-**[Ir]** and (*rac*)-Bn-BTM

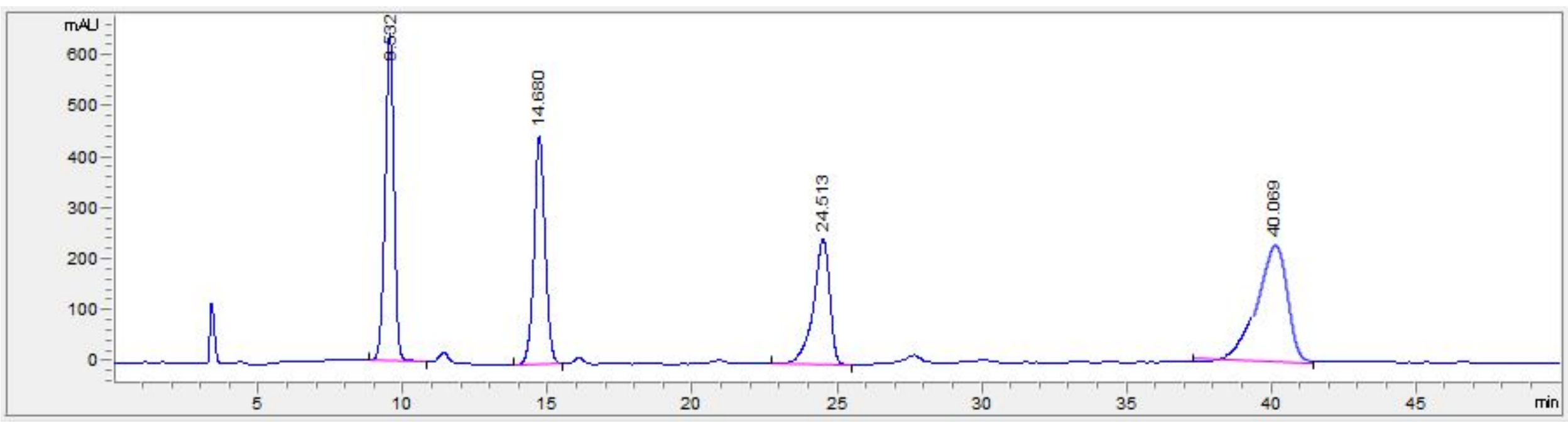


| # | Time | Area | Height | Width | Area% | Symmetry |
|---|---|---|---|---|---|---|
| 1 | 9.532 | 13625.2 | 644.2 | 0.3207 | 26.191 | 1.077 |
| 2 | 14.68 | 11824.2 | 448.5 | 0.3996 | 22.729 | 0.89 |
| 3 | 24.513 | 9619.9 | 245.5 | 0.5932 | 18.492 | 1.468 |
| 4 | 40.069 | 16952.8 | 231.5 | 1.2205 | 32.588 | 1.409 |

(*R*,*S*)-**22:** (*S$_a$*,*S*,*S*)-**[Ir]** and (*S*)-Me-BTM

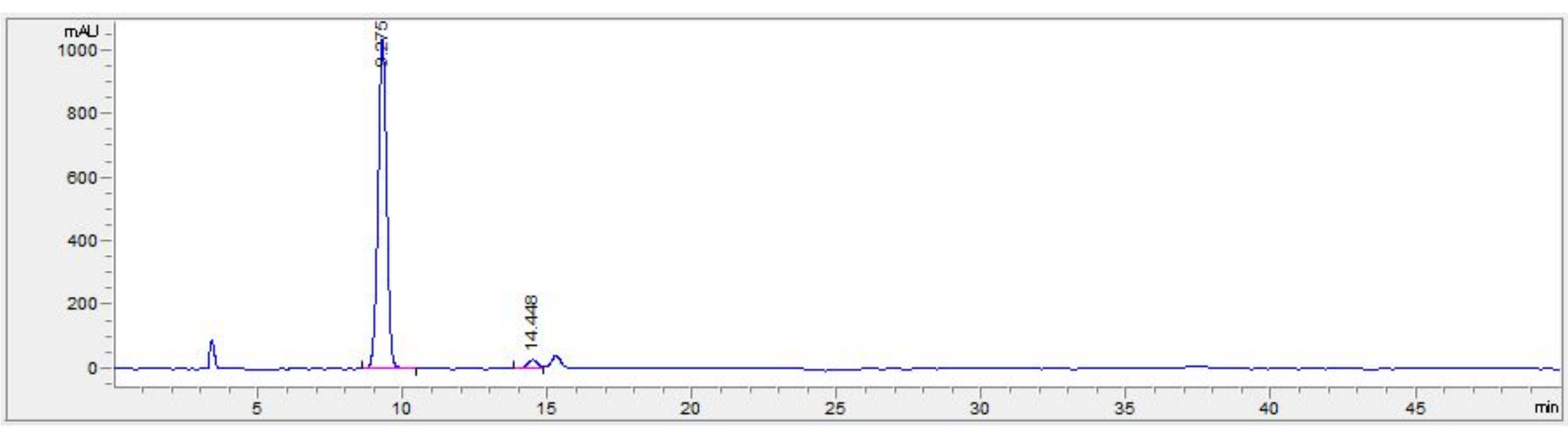


| # | Time | Area | Height | Width | Area% | Symmetry |
|---|---|---|---|---|---|---|
| 1 | 9.275 | 21351.2 | 1037.8 | 0.316 | 96.567 | 0.988 |
| 2 | 14.448 | 759.1 | 28 | 0.4128 | 3.433 | 1.073 |

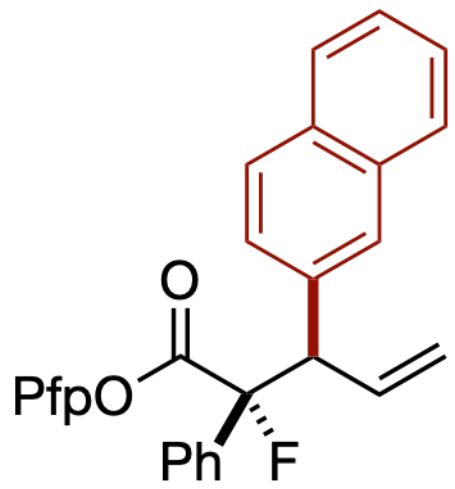

**Perfluorophenyl (2*R*,3*R*)-2-fluoro-3-(naphthalen-2-yl)-2-phenylpent-4-enoate ((*R*,*R*)-15):** Prepared according to General Procedure B using pentafluorophenyl ester **1a** (32 mg, 0.1 mmol, 1 equiv.), *t*-butyl carbonate **2i** (42.6 mg, 0.15 mmol, 1.5 equiv.), ($R_a$,*R,R*)-**[Ir]** (4.2 mg, 4 mol%), (*S*)-Me-BTM (3.8 mg, 20 mol%) and DIPEA (22.6 µL, 0.13 mmol, 1.3 equiv.) in 1,4-dioxane (0.3 ml, 0.3 M). The diastereomeric ratio was determined to be 55:1. The *title compound* was obtained (41.3 mg, 0.085 mmol, 85%) as a white solid following purification by column chromatography [$SiO_2$, 0.25% Ethyl Acetate in Petroleum Ether]. The enantiomeric ratio (>99:1 *er*) of the corresponding primary amide was determined by chiral HPLC in comparison with the racemate. Diastereomeric ratio (98:2) was determined by $^{19}$F-NMR analysis of the crude reaction mixture.

**IR (film):** 3062 (w), 1782 (m), 1638 (w), 1600 (w), 1518 (s), 1471 (w), 1193 (m), 1008 (s), 996 (s), 748 (s) cm$^{-1}$.

**$^1$H NMR (500 MHz, CDCl$_3$):** δ 7.70 (ddd, *J* = 10.3, 6.5, 3.3 Hz, 2H), 7.66 – 7.57 (m, 2H), 7.53 – 7.43 (m, 2H), 7.43 – 7.35 (m, 2H), 7.28 (dt, *J* = 8.5, 1.7 Hz, 1H), 7.24 – 7.16 (m, 3H), 6.47 (ddd, *J* = 17.0, 10.3, 8.6 Hz, 1H), 5.43 (d, *J* = 17.1 Hz, 1H), 5.38 (d, *J* = 10.3 Hz, 1H), 4.60 (dd, *J* = 33.5, 9.0 Hz, 1H).

**$^{13}$C NMR (126 MHz, CDCl$_3$):** δ 166.6 (d, *J* = 28.2 Hz), 135.1 (d, *J* = 22.5 Hz), 134.6 (d, *J* = 5.1 Hz), 134.2, 133.3, 132.6, 129.0, 128.9 (d, *J* = 2.1 Hz), 128.5 (d, *J* = 2.2 Hz), 128.0, 128.0, 127.6, 127.4 (d, *J* = 2.8 Hz), 126.1, 126.0, 125.0 (d, *J* = 9.7 Hz), 120.1, 99.9 (d, *J* = 201.4 Hz), 57.4 (d, *J* = 18.9 Hz).

**$^{19}$F NMR (376 MHz, CDCl$_3$):** δ -151.32 – -151.55 (m, 2F), -156.92 (t, *J* = 21.7 Hz, 1F), -161.66 – -161.92 (m, 2F), -174.06 (d, *J* = 33.1 Hz, 1F).

**HRMS (EI):** m/z calcd for $[M]^+$ $C_{27}H_{16}F_6O_2$: 486.1054. Found: 486.1059.

**(2*R*,3*R*)-2-Fluoro-3-(naphthalen-2-yl)-2-phenylpent-4-enamide ((*R*,*R*)-15-NH$_2$):**

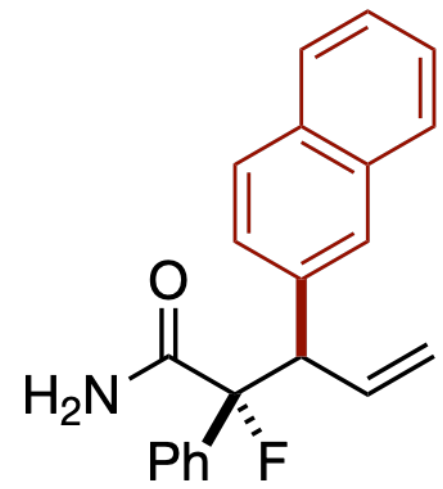

**IR (film):** 3439 (m), 3175 (br), 3061 (w), 2921 (w), 1671 (s), 1600 (w), 1509 (w), 1448 (w), 1392 (m) $cm^{-1}$.

**$^1$H NMR (500 MHz, $CDCl_3$):** δ 7.77 – 7.64 (m, 2H), 7.60 (d, *J* = 8.5 Hz, 1H), 7.55 (s, 1H), 7.49 – 7.43 (m, 2H), 7.41 – 7.35 (m, 2H), 7.29 – 7.22 (m, 1H), 7.18 – 7.07 (m, 3H), 6.46 (s, 1H), 6.43 – 6.31 (m, 1H), 5.60 (s, 1H), 5.40 – 5.14 (m, 2H), 4.58 (dd, *J* = 34.5, 8.0 Hz, 1H).

**$^{13}$C NMR (126 MHz, $CDCl_3$):** δ 172.6 (d, *J* = 22.9 Hz), 136.8 (d, *J* = 22.0 Hz), 135.3, 135.2 (d, *J* = 5.4 Hz), 133.3, 132.4, 128.8 (d, *J* = 1.9 Hz), 128.2, 128.0 (d, *J* = 2.0 Hz), 127.9, 127.6, 127.63 (d, *J* = 2.6 Hz), 127.6, 125.9, 125.7, 124.8 (d, *J* = 10.4 Hz), 119.4, 101.7 (d, *J* = 197.5 Hz), 57.0 (d, *J* = 19.4 Hz).

**$^{19}$F NMR (376 MHz, $CDCl_3$):** δ -170.96 (d, *J* = 34.8 Hz, 1F).

**HRMS (ESI):** m/z calcd for $[M+Na]^+$ $C_{21}H_{18}ONFNa$: 342.1265. Found: 342.1265.

HPLC analysis using a chiral column (Chiralpak IB 3μ column 22 ˚C, 1.0 ml/min, 95:5 hexane:isopropanol, 210 nm, $t_{major}$ = 14.057 min, $t_{minor}$ = 40.095 min, t = 16.998 min, t = 22.909 min).

(±)-**15** (*rac*)-**[Ir]** and (*rac*)-Bn-BTM

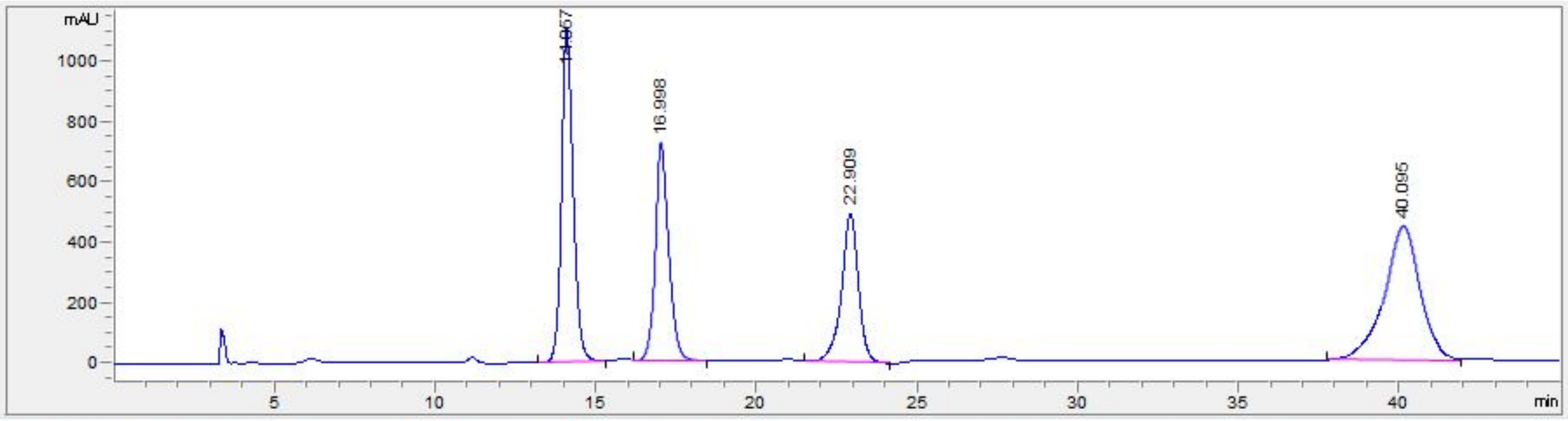


| # | Time | Area | Height | Width | Area% | Symmetry |
|---|---|---|---|---|---|---|
| 1 | 14.057 | 27326.1 | 1109.4 | 0.3694 | 27.250 | 0.737 |
| 2 | 16.998 | 20667.3 | 718.8 | 0.4235 | 20.610 | 0.715 |
| 3 | 22.909 | 17766.5 | 493.5 | 0.536 | 17.717 | 1.109 |
| 4 | 40.095 | 34518.6 | 444.2 | 1.295 | 34.423 | 1.083 |

(*R*,*R*)-**15:** (*R$_a$,R,R*)-**[Ir]** and (*S*)-Me-BTM

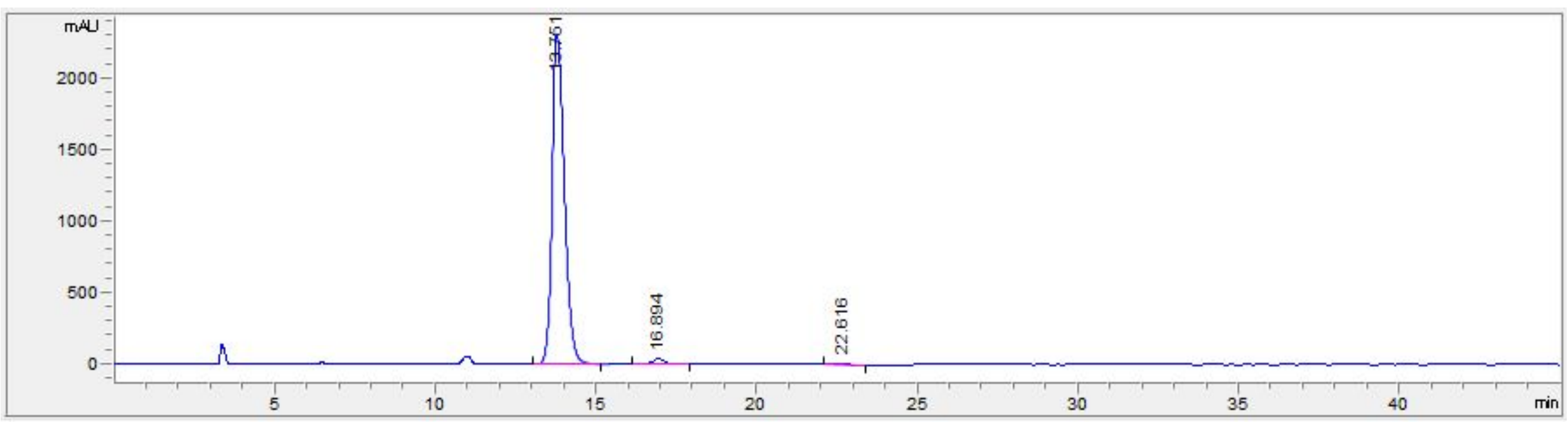


| # | Time | Area | Height | Width | Area% | Symmetry |
|---|---|---|---|---|---|---|
| 1 | 13.751 | 61234.6 | 2314.8 | 0.4046 | 97.825 | 0.606 |
| 2 | 16.894 | 1088.2 | 37.6 | 0.4357 | 1.738 | 0.893 |
| 3 | 22.616 | 273.4 | 8.2 | 0.477 | 0.437 | 0.822 |

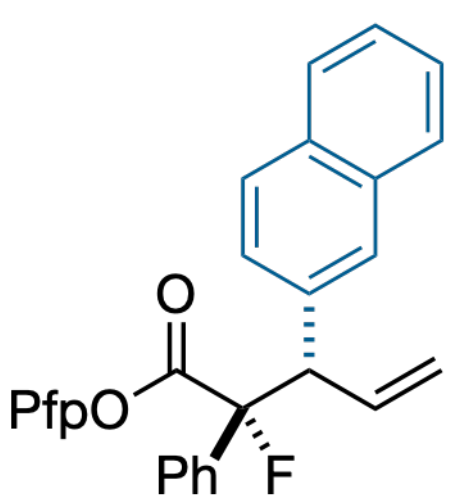


**Perfluorophenyl (2*R*,3*S*)-2-fluoro-3-(naphthalen-2-yl)-2-phenylpent-4-enoate ((*R*,*S*)-15):** Prepared according to General Procedure B using pentafluorophenyl ester **1a** (32 mg, 0.1 mmol, 1 equiv.), *t*-butyl carbonate **2i** (42.6 mg, 0.15 mmol, 1.5 equiv.), (*S$_a$,S,S*)-**[Ir]** (4.2 mg, 4 mol%), (*S*)-Me-BTM (3.8 mg, 20 mol%) and DIPEA (22.6 μL, 0.13 mmol, 1.3 equiv.) in 1,4-dioxane (0.3 ml, 0.3 M). The diastereomeric ratio was determined to be 1:32. The *title compound* was obtained (38.7 mg, 0.0795 mmol, 79.5%) as a white solid following purification by column chromatography [$SiO_2$, 0.25% Ethyl Acetate in Petroleum Ether]. The enantiomeric ratio (>99:1 *er*) of the corresponding primary amide was determined by chiral HPLC in comparison with the racemate.

Diastereomeric ratio (96:4) was determined by $^{19}$F-NMR analysis of the crude reaction mixture.

**IR (film):** 3062 (w), 1798 (w), 1780 (m), 1637 (w), 1600 (w), 1518 (s), 1471 (w), 1194 (m), 1007 (s), 997 (s), 749 (s) $cm^{-1}$.

**$^1$H NMR (500 MHz, $CDCl_3$):** δ 7.99 (s, 1H), 7.85 (td, *J* = 6.3, 3.1 Hz, 3H), 7.78 – 7.71 (m, 2H), 7.60 (dt, *J* = 8.6, 1.8 Hz, 1H), 7.50 (td, *J* = 6.4, 2.9 Hz, 3H), 7.47 – 7.41 (m, 1H), 6.10 – 6.01 (m, 1H), 5.11 (dd, *J* = 10.4, 1.3 Hz, 1H), 4.96 (d, *J* = 17.1 Hz, 1H), 4.62 (dd, *J* = 34.0, 8.0 Hz,1H).

**$^{13}$C NMR (126 MHz, $CDCl_3$):** δ 166.0 (d, *J* = 28.8 Hz), 135.7 (d, *J* = 22.7 Hz), 135.1, 133.9 (d, *J* = 4.8 Hz), 133.6, 133.0, 129.4, 128.9 (d, *J* = 2.1 Hz), 128.6 (d, *J* = 2.9 Hz), 128.5, 128.2, 127.7, 127.4 (d, *J* = 3.3 Hz), 126.3, 126.2, 125.4 (d, *J* = 10.4 Hz), 120.2, 99.4 (d, *J* = 201.2 Hz), 56.9 (d, *J* = 18.8 Hz).

**$^{19}$F NMR (376 MHz, $CDCl_3$):** δ -151.59 – -151.78 (m, 2F), -157.25 (t, *J* = 21.6 Hz, 1F), -161.82 – -162.18 (m, 2F), -172.86 (d, *J* = 33.7 Hz, 1F).

**HRMS (EI):** m/z calcd for $[M]^+$ $C_{27}H_{16}F_6O_2$: 486.1054. Found: 486.1061.

**(2*R*,3*S*)-2-Fluoro-3-(naphthalen-2-yl)-2-phenylpent-4-enamide ((*R*,*S*)-15-$NH_2$):**

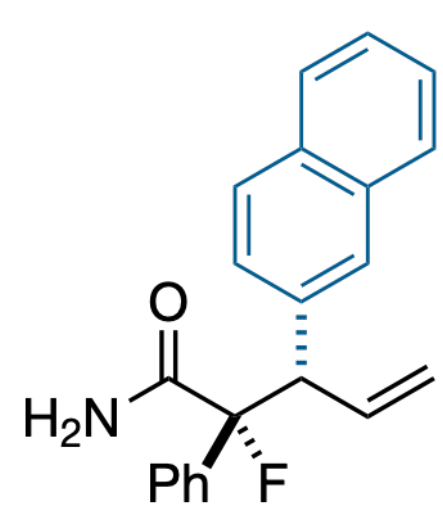


**IR (film):** 3449 (m), 3170 (br), 1671 (s), 1599 (w), 1490 (w), 1447 (w), 1393 (w) $cm^{-1}$.

**$^1$H NMR (500 MHz, $CDCl_3$):** δ 7.90 (s, 1H), 7.87 – 7.79 (m, 3H), 7.77 – 7.69 (m, 2H), 7.57 (dt, *J* = 8.5, 1.9 Hz, 1H), 7.52 – 7.40 (m, 4H), 7.39 – 7.34 (m, 1H), 6.14 – 5.97 (m, 3H), 5.28 – 5.24 (m, 0.1H), 5.03 (dt, *J* = 10.4, 1.3 Hz, 1H), 4.98 (s, 1H), 4.90 (dt, *J* = 17.2, 1.4 Hz, 1H), 4.57 (dd, *J* = 36.0, 8.0 Hz, 1H).

**$^{13}$C NMR (126 MHz, $CDCl_3$):** δ 172.0 (d, *J* = 23.3 Hz), 137.3 (d, *J* = 22.0 Hz), 136.0, 134.5 (d, *J* = 4.3 Hz), 133.5, 132.8, 129.1 (d, *J* = 1.9 Hz), 128.5, 128.4 (d, *J* = 2.2 Hz), 128.2, 128.0 (d, *J* = 2.8 Hz), 127.9, 127.7, 126.1, 126.0, 125.2 (d, *J* = 11.1 Hz), 119.6, 101.2 (d, *J* = 198.7 Hz), 56.6 (d, *J* = 18.9 Hz).

**$^{19}$F NMR (376 MHz, $CDCl_3$):** δ -172.78 (d, *J* = 36.4 Hz, 1F).

**HRMS (ESI):** m/z calcd for $[M+Na]^+$ $C_{21}H_{18}ONFNa$: 342.1265. Found: 342.1266.

HPLC analysis using a chiral column (Chiralpak IB 3μ column 22 ˚C, 1.0 ml/min, 95:5 hexane:isopropanol, 210 nm, $t_{major}$ = 16.998 min, $t_{minor}$ = 22.909 min, t = 14.057 min, t = 40.095 min).

(±)-**15:** (*rac*)-**[Ir]** and (*rac*)-Bn-BTM

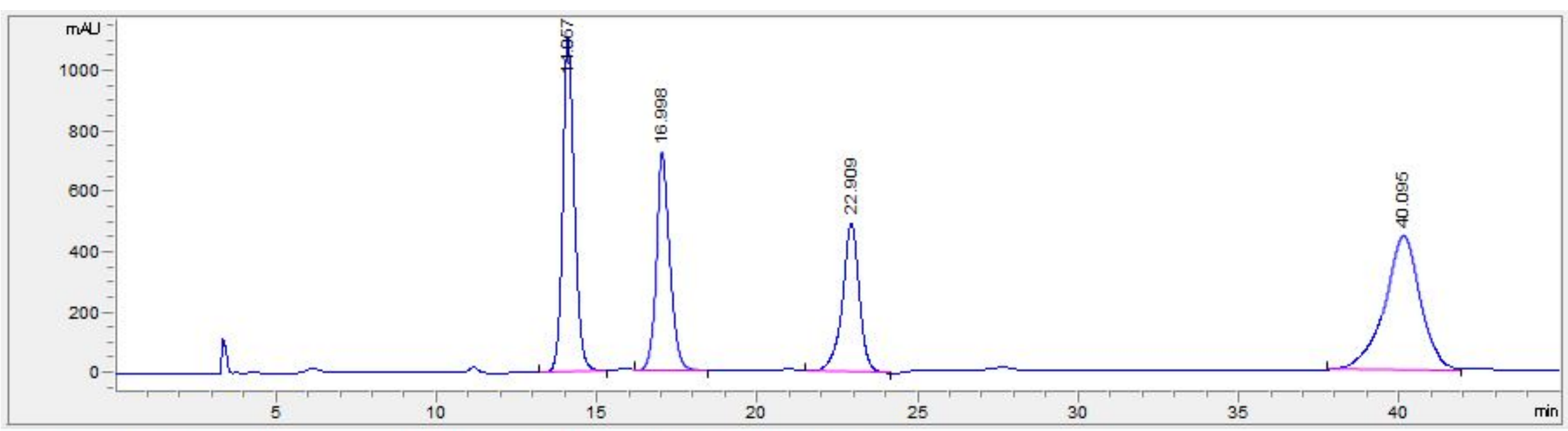


| # | Time | Area | Height | Width | Area% | Symmetry |
|---|---|---|---|---|---|---|
| 1 | 14.057 | 27326.1 | 1109.4 | 0.3694 | 27.250 | 0.737 |
| 2 | 16.998 | 20667.3 | 718.8 | 0.4235 | 20.610 | 0.715 |
| 3 | 22.909 | 17766.5 | 493.5 | 0.536 | 17.717 | 1.109 |
| 4 | 40.095 | 34518.6 | 444.2 | 1.295 | 34.423 | 1.083 |

(*R*,*S*)-**15:** (*S$_a$*,*S*,*S*)-**[Ir]** and (*S*)-Me-BTM

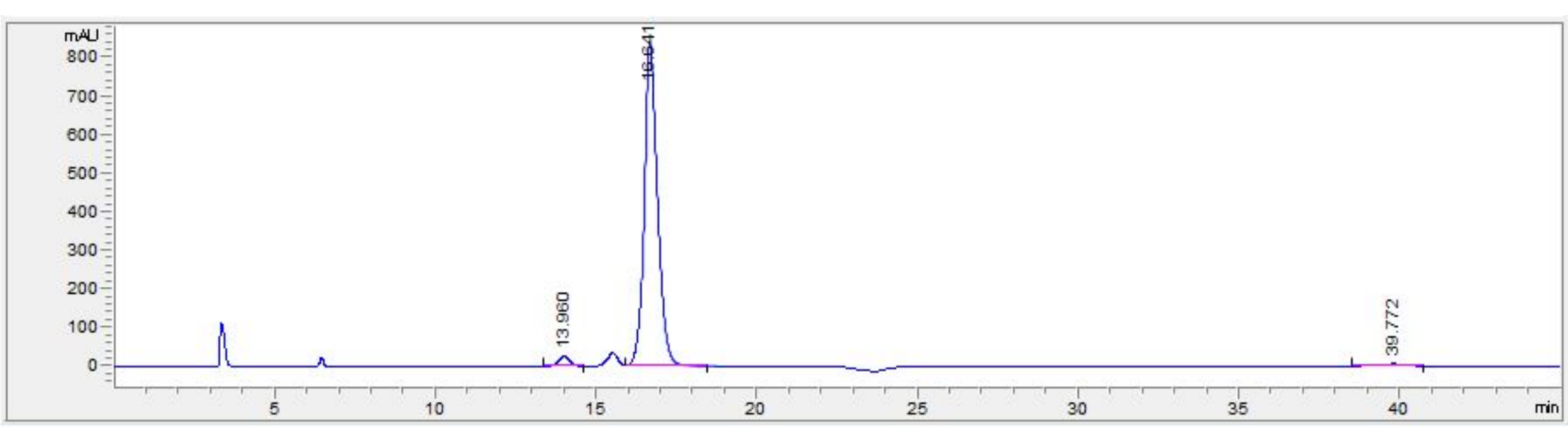


| # | Time | Area | Height | Width | Area% | Symmetry |
|---|---|---|---|---|---|---|
| 1 | 13.96 | 609.3 | 25.9 | 0.3582 | 2.462 | 0.955 |
| 2 | 16.641 | 23773.2 | 838.4 | 0.4208 | 96.058 | 0.694 |
| 3 | 39.772 | 366.1 | 6 | 0.7439 | 1.479 | 1.301 |

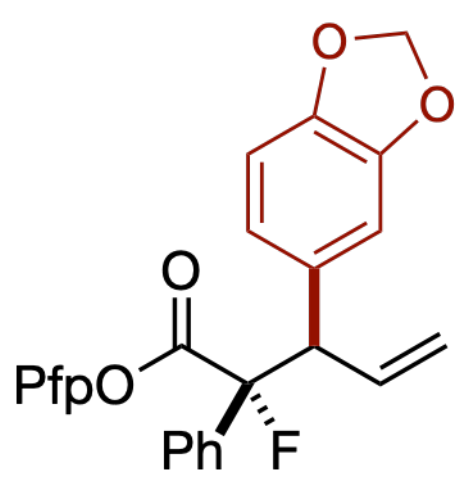


**Perfluorophenyl (2*R*,3*R*)-3-(benzo[d][1,3]dioxol-5-yl)-2-fluoro-2-phenylpent-4-enoate ((*R*,*R*)-23):** Prepared according to General Procedure B using pentafluorophenyl ester **1a** (32 mg, 0.1 mmol, 1 equiv.), *t*-butyl carbonate **2j** (41.7 mg, 0.15 mmol, 1.5 equiv.) and ($R_a$,*R*,*R*)-**[Ir]** (4.2 mg, 4 mol%), (*S*)-Me-BTM (3.8 mg, 20 mol%) and DIPEA (22.6 µL, 0.13 mmol, 1.3 equiv.) in 1,4-dioxane (0.3 ml, 0.3 M). The diastereomeric ratio was determined to be 56:1. The *title compound* was obtained (38.1 mg, 0.079 mmol, 79%) as a colorless oil following purification by column chromatography [$SiO_2$, 0.25% Ethyl Acetate in Petroleum Ether]. The enantiomeric ratio (>99:1 *er*) of the corresponding primary amide was determined by chiral HPLC in comparison with the racemate. Diastereomeric ratio (98:2) was determined by $^{19}$F-NMR analysis of the crude reaction mixture.

**IR (film):** 2897 (w), 1782 (m), 1639 (w), 1610 (w), 1519 (s), 1506 (s), 1491 (m), 1249 (m), 1197 (m), 1008 (s), 996 (s), 747 (m) $cm^{-1}$.

**$^1$H NMR (500 MHz, $CDCl_3$):** δ 7.48 – 7.41 (m, 2H), 7.32 – 7.26 (m, 3H), 6.70 (d, *J* = 1.7 Hz, 1H), 6.62 – 6.48 (m, 2H), 6.31 (ddd, *J* = 17.1, 10.3, 8.7 Hz, 1H), 5.37 (d, *J* = 17.3 Hz, 1H), 5.33 (d, *J* = 10.6 Hz, 1H), 4.33 (dd, *J* = 33.0, 8.5 Hz, 1H).

**$^{13}$C NMR (126 MHz, $CDCl_3$):** δ 166.0 (d, *J* = 28.7 Hz), 148.0, 147.3, 135.6 (d, *J* = 22.8 Hz), 133.9 (d, *J* = 4.9 Hz), 131.2, 129.4, 128.9 (d, *J* = 2.1 Hz), 125.3 (d, *J* = 10.2 Hz), 123.1 (d, *J* = 2.8 Hz), 119.8, 109.9 (d, *J* = 4.1 Hz), 108.5, 101.2, 99.3 (d, *J* = 201.0 Hz), 56.6 (d, *J* = 18.8 Hz).

**$^{19}$F NMR (376 MHz, $CDCl_3$):** δ -151.39 – -151.58 (m, 2F), -156.95 (t, *J* = 21.7 Hz, 1F), -161.68 – -162.03 (m, 2F), -174.77 (d, *J* = 32.9 Hz, 1F).

**HRMS (EI):** m/z calcd for $[M]^+$ $C_{24}H_{14}F_6O_4$: 480.0796. Found: 480.0801.

**(2*R*,3*R*)-3-(benzo[d][1,3]dioxol-5-yl)-2-fluoro-2-phenylpent-4-enamide ((*R*,*R*)-23-$NH_2$):**

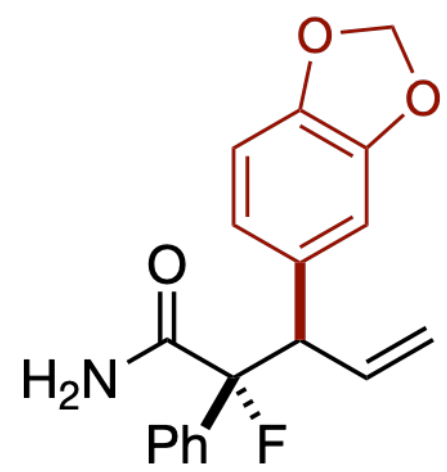


**IR (film):** 3434 (m), 3180 (br), 1669 (s), 1608 (w), 1504 (m), 1491 (m), 1394 (w), 1249 (m) $cm^{-1}$.

**$^1$H NMR (500 MHz, $CDCl_3$):** δ 7.47 – 7.39 (m, 2H), 7.24 – 7.15 (m, 3H), 6.66 (d, *J* = 1.6 Hz, 1H), 6.54 (d, *J* = 8.0 Hz, 1H), 6.49 (dt, *J* = 8.1, 1.3 Hz, 1H), 6.41 (s, 1H), 6.22 (ddd, *J* = 17.7, 10.6, 8.3 Hz, 1H), 5.91 – 5.75 (m, 2H), 5.46 (s, 1H), 5.32 – 5.18 (m, 2H), 4.32 (dd, *J* = 34.5, 8.5 Hz, 1H).

**$^{13}$C NMR (126 MHz, $CDCl_3$):** δ 172.4 (d, *J* = 22.9 Hz), 147.4, 146.4, 136.8 (d, *J* = 22.0 Hz), 135.3 (d, *J* = 5.4 Hz), 131.4, 128.16, 128.1 (d, *J* = 2.2 Hz), 124.8 (d, *J* = 10.5 Hz), 123.2 (d, *J* = 1.8 Hz), 119.1, 109.7 (d, *J* = 3.2 Hz), 108.0, 101.7 (d, *J* = 197.2 Hz), 100.9, 56.6 (d, *J* = 19.4 Hz).

**$^{19}$F NMR (376 MHz, $CDCl_3$):** δ -171.95 (d, *J* = 34.7 Hz, 1F).

**HRMS (ESI):** m/z calcd for $[M+Na]^+$ $C_{18}H_{16}O_3NFNa$: 336.1006. Found: 336.1007.

HPLC analysis using a chiral column (Chiralpak IB 3µ column 22 ˚C, 1.0 ml/min, 95:5 hexane:isopropanol, 210 nm, $t_{major}$ = 15.826 min, $t_{minor}$ = 45.539 min, t = 19.688 min, t = 28.505 min).

(±)-**23:** (*rac*)-**[Ir]** and (*rac*)-Bn-BTM

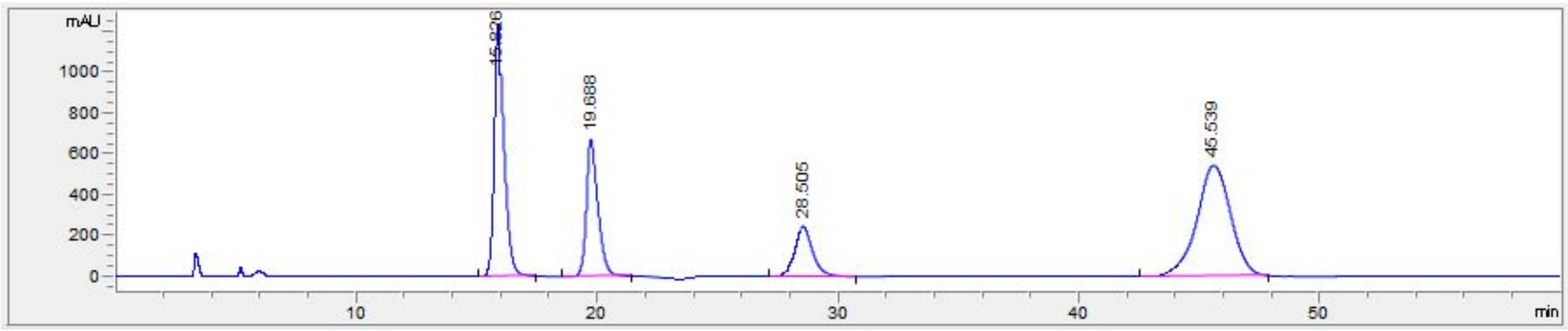


| # | Time | Area | Height | Width | Area% | Symmetry |
|---|---|---|---|---|---|---|
| 1 | 15.826 | 35442.9 | 1235.4 | 0.4267 | 28.780 | 0.548 |
| 2 | 19.688 | 22172.1 | 668.2 | 0.4851 | 18.004 | 0.592 |
| 3 | 28.505 | 12402.3 | 245.2 | 0.746 | 10.071 | 0.806 |
| 4 | 45.539 | 53134.3 | 539.1 | 1.4918 | 43.145 | 0.979 |

(*R*,*R*)-**23:** (*R*$_a$,*R*,*R*)-**[Ir]** and (*S*)-Me-BTM

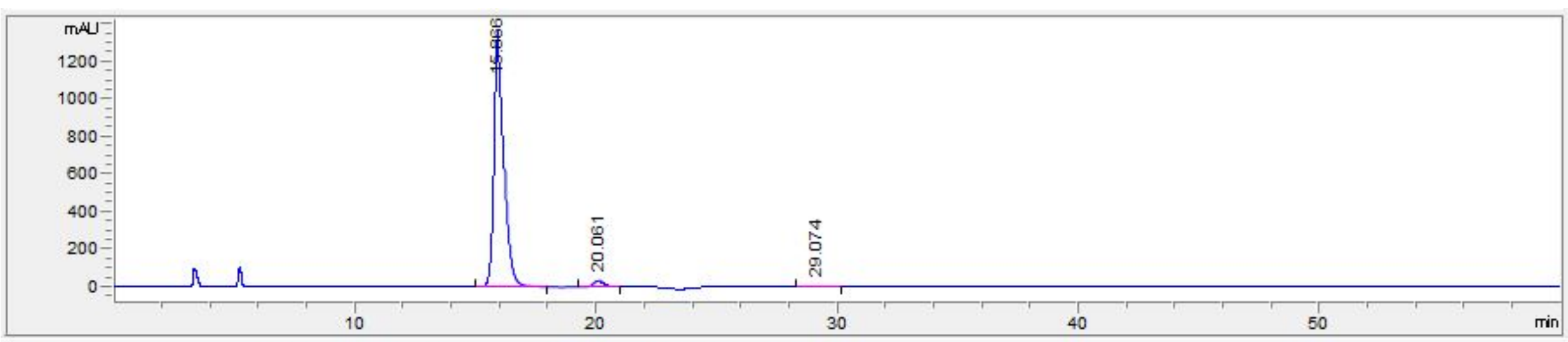


| # | Time | Area | Height | Width | Area% | Symmetry |
|---|---|---|---|---|---|---|
| 1 | 15.866 | 39777.9 | 1357 | 0.438 | 96.717 | 0.533 |
| 2 | 20.061 | 1092.9 | 34.5 | 0.482 | 2.657 | 0.851 |
| 3 | 29.074 | 257.4 | 5 | 0.6084 | 0.626 | 1.071 |

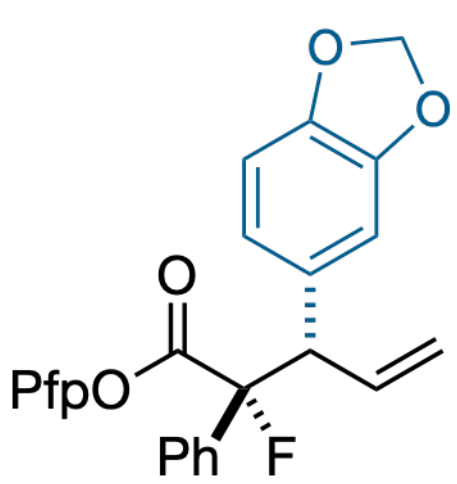


**Perfluorophenyl (2*R*,3*S*)-3-(benzo[d][1,3]dioxol-5-yl)-2-fluoro-2-phenylpent-4-enoate ((*R*,*S*)-23):** Prepared according to General Procedure B using pentafluorophenyl **1a** (32 mg, 0.1 mmol, 1 equiv.), *t*-butyl carbonate **2j** (41.7 mg, 0.15 mmol, 1.5 equiv.) and (*S*$_a$,*S*,*S*)-**[Ir]** (4.2 mg, 4 mol%), (*S*)-Me-BTM (3.8 mg, 20 mol%) and DIPEA (22.6 μL, 0.13 mmol, 1.3 equiv.) in 1,4-dioxane (0.3 ml, 0.3 M). The diastereomeric ratio was determined to be 1:15. The *title compound* was obtained (38.4 mg, 0.080 mmol, 80%) as a white solid following purification by column chromatography [$SiO_2$, 0.25% Ethyl Acetate in Petroleum Ether]. The enantiomeric ratio (>99:1 *er*) of the corresponding primary amide was determined by chiral HPLC in comparison with the racemate. Diastereomeric ratio (94:6) was determined by $^{19}$F-NMR analysis of the crude reaction mixture.

**IR (film):** 2894 (w), 1797 (w), 1781 (w), 1640 (w), 1610 (w), 1520 (s), 1505 (m), 1489 (m), 1249 (m), 1195 (m), 1009 (s), 998 (s), 748 (m) cm$^{-1}$.

**$^1$H NMR (500 MHz, $CDCl_3$):** δ 7.79 – 7.62 (m, 2H), 7.47 (dd, *J* = 8.4, 6.5 Hz, 2H), 7.44 – 7.40 (m, 1H), 7.01 (t, *J* = 1.7 Hz, 1H), 6.95 (dt, *J* = 8.0, 1.3 Hz, 1H), 6.81 (d, *J* = 8.0 Hz, 1H), 5.97 (s, 2H), 5.92 (ddd, *J* = 17.1, 10.4, 7.8 Hz, 1H), ****5.37 (d, J = 17.0 Hz), *5.34 (d, J = 10.3 Hz)***, 5.06 (dt, *J* = 10.4, 1.2 Hz, 1H), 4.92 (dt, *J* = 17.1, 1.4 Hz, 1H), 4.35 (dd, *J* = 33.5, 7.5 Hz, 1H).

**$^{13}$C NMR (126 MHz, $CDCl_3$):** δ 166.0 (d, *J* = 28.7 Hz), 148.0, 147.3, 135.6 (d, *J* = 22.8 Hz), 133.9 (d, *J* = 4.9 Hz), 131.2, 129.4, 128.9 (d, *J* = 2.2 Hz), 125.3 (d, *J* = 10.2 Hz), 123.1 (d, *J* = 2.8 Hz), 119.8, 109.9 (d, *J* = 4.1 Hz), 108.5, 101.2, 99.3 (d, *J* = 201.0 Hz), 56.6 (d, *J* = 18.8 Hz).

**$^{19}$F NMR (376 MHz, $CDCl_3$):** δ -151.62 – -151.90 (m, 2F), -157.17 (t, *J* = 21.6 Hz, 1F), -161.87 – -162.08 (m, 2F), -173.70 (d, *J* = 33.5 Hz, 1F).

**HRMS (EI):** m/z calcd for $[M]^+$ $C_{24}H_{14}F_6O_4$: 480.0796. Found: 480.0798.

**(2*R*,3*S*)-3-(Benzo[d][1,3]dioxol-5-yl)-2-fluoro-2-phenylpent-4-enamide ((*R*,*S*)-23-$NH_2$):**

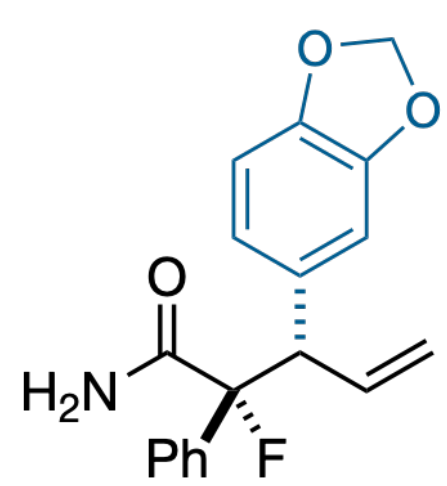


**IR (film):** 3436 (w), 3224 (br), 2910 (w), 1666 (s), 1596 (w), 1504 (m), 1488 (m), 1395 (w), 1250 (m) $cm^{-1}$.

**$^1$H NMR (500 MHz, $CDCl_3$):** δ 7.68 – 7.61 (m, 2H), 7.44 – 7.36 (m, 2H), 7.36 – 7.30 (m, 1H), 6.94 (t, *J* = 1.8 Hz, 1H), 6.89 (dt, *J* = 8.1, 1.4 Hz, 1H), 6.77 (d, *J* = 8.0 Hz, 1H), 6.10 (s, 1H), 5.98 – 5.93 (m, 2H), 5.93 – 5.83 (m, 1H), ****5.27 – 5.20 (m)***, 5.12 (s, 1H), 4.98 (dt, *J* = 10.4, 1.2 Hz, 1H), 4.85 (dt, *J* = 17.1, 1.4 Hz, 1H), 4.31 (dd, *J* = 36.0, 8.0 Hz, 1H).

**$^{13}$C NMR (126 MHz, $CDCl_3$):** δ 172.0 (d, *J* = 23.3 Hz), 147.6, 146.8, 137.2 (d, *J* = 22.1 Hz), 134.6 (d, *J* = 4.5 Hz), 132.1, 128.4, 128.4 (d, *J* = 2.2 Hz), 125.1 (d, *J* = 11.1 Hz),

123.5 (d, $J$ = 1.9 Hz), 119.2, 110.2 (d, $J$ = 3.2 Hz), 108.3, 101.1, 101.2 (d, $J$ = 198.5 Hz), 56.1 (d, $J$ = 18.9 Hz).

**$^{19}$F NMR (376 MHz, CDCl$_3$):** δ -173.25 (d, $J$ = 36.1 Hz, 1F).

**HRMS (ESI):** m/z calcd for $[M+Na]^+$ $C_{18}H_{16}O_3NFNa$: 336.1006. Found: 336.1007.

HPLC analysis using a chiral column (Chiralpak IB 3µ column 22 ˚C, 1.0 ml/min, 95:5 hexane:isopropanol, 210 nm, $t_{major}$ = 19.688 min, $t_{minor}$ = 28.505 min, t = 15.826 min, t = 45.539 min).

(±)-**23:** (*rac*)-**[Ir]** and (*rac*)-Bn-BTM

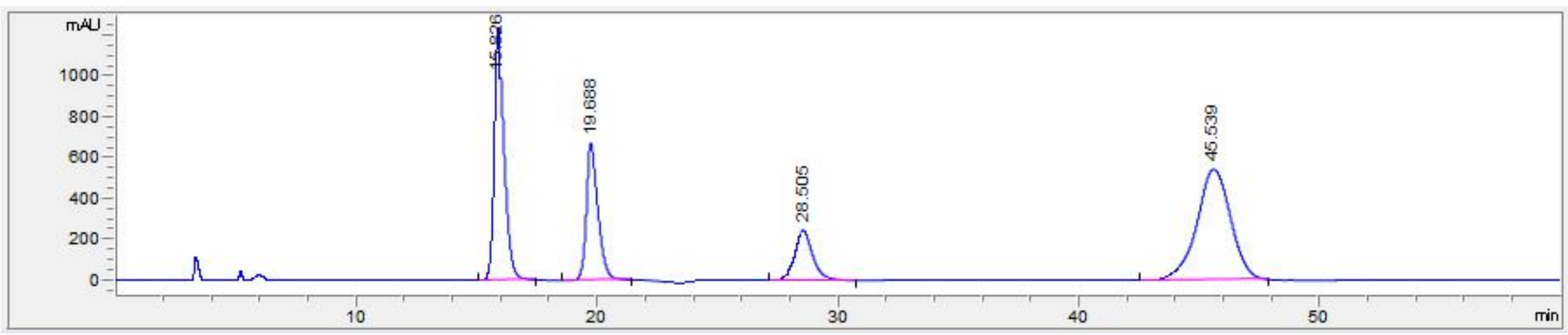


| # | Time | Area | Height | Width | Area% | Symmetry |
|---|---|---|---|---|---|---|
| 1 | 15.826 | 35442.9 | 1235.4 | 0.4267 | 28.780 | 0.548 |
| 2 | 19.688 | 22172.1 | 668.2 | 0.4851 | 18.004 | 0.592 |
| 3 | 28.505 | 12402.3 | 245.2 | 0.746 | 10.071 | 0.806 |
| 4 | 45.539 | 53134.3 | 539.1 | 1.4918 | 43.145 | 0.979 |

(*R*,*S*)-**23:** (*S*$_a$*,S,S*)-**[Ir]** and (*S*)-Me-BTM

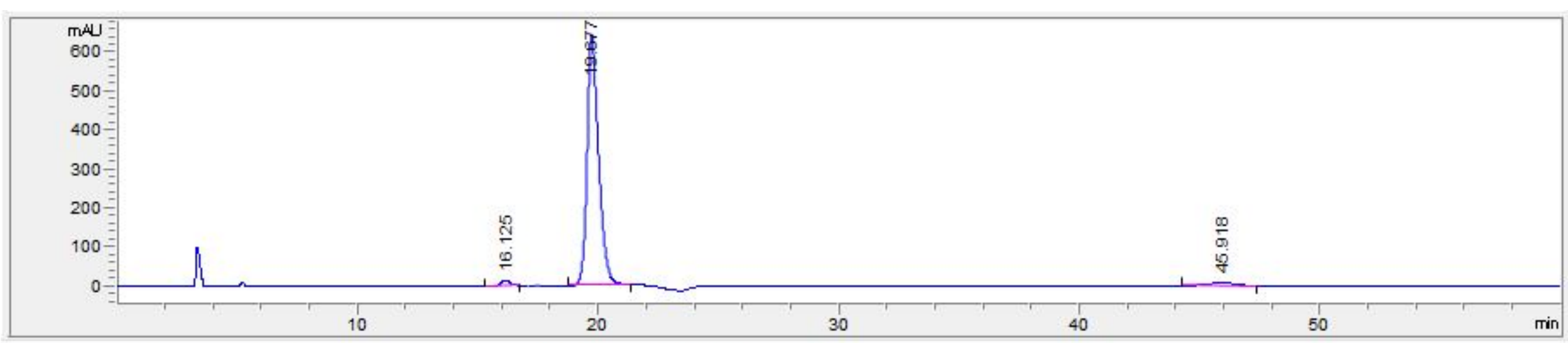


| # | Time | Area | Height | Width | Area% | Symmetry |
|---|---|---|---|---|---|---|
| 1 | 16.125 | 404.5 | 13.9 | 0.4427 | 1.786 | 0.989 |
| 2 | 19.677 | 21448.9 | 646.1 | 0.4893 | 94.678 | 0.612 |
| 3 | 45.918 | 801.2 | 9.8 | 1.0155 | 3.537 | 1.216 |

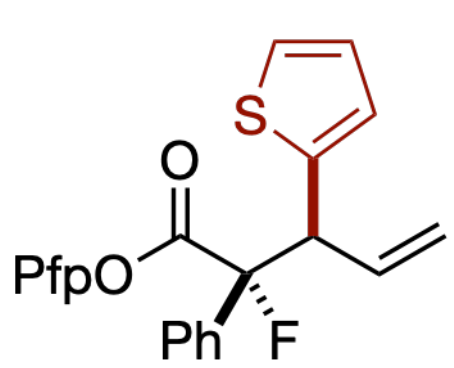


**Perfluorophenyl (2*R*,3*S*)-2-fluoro-2-phenyl-3-(thiophen-2-yl)pent-4-enoate ((*R*,*S*)-25):** Prepared according to General Procedure B using pentafluorophenyl ester **1a** (32 mg, 0.1 mmol, 1 equiv.), *t*-butyl carbonate **2k** (36.1 mg, 0.15 mmol, 1.5 equiv.), (*R*$_a$*,R,R*)-**[Ir]** (4.2 mg, 4

mol%), (*S*)-Me-BTM (3.8 mg, 20 mol%) and DIPEA (22.6 µL, 0.13 mmol, 1.3 equiv.) in 1,4-dioxane (0.3 ml, 0.3 M). The *title compound* was obtained (34.8 mg, 0.079 mmol, 79%) as a colorless oil following purification by column chromatography [$SiO_2$, 0.25% Ethyl Acetate in Petroleum Ether]. The enantiomeric ratio (>99:1 *er*) of the corresponding primary amide was determined by chiral HPLC in comparison with the racemate. Diastereomeric ratio (99:1) was determined by $^{19}$F-NMR analysis of the crude reaction mixture.

**IR (film):** 3072 (w), 1782 (m), 1640 (w), 1517 (s), 1472 (w), 1187 (m), 1007 (s), 995 (s), 745 (m), 693 (s) cm$^{-1}$.

**$^1$H NMR (500 MHz, $CDCl_3$):** δ 7.56 – 7.48 (m, 2H), 7.37 – 7.31 (m, 3H), 7.09 (dd, *J* = 5.2, 1.2 Hz, 1H), 6.81 (dd, *J* = 5.1, 3.5 Hz, 1H), 6.76 (d, *J* = 3.5 Hz, 1H), 6.33 (dt, *J* = 17.0, 9.5 Hz, 1H), 5.43 (d, *J* = 17.0 Hz, 1H), 5.38 (d, *J* = 10.2 Hz, 1H), 4.74 (dd, *J* = 32.0, 9.0 Hz, 1H).

**$^{13}$C NMR (126 MHz, $CDCl_3$):** δ 165.8 (d, *J* = 28.5 Hz), 138.9, 135.1 (d, *J* = 22.5 Hz), 133.5 (d, *J* = 4.5 Hz), 129.5, 128.9 (d, *J* = 2.0 Hz), 127.3 (d, *J* = 2.4 Hz), 127.0, 125.8, 125.2 (d, *J* = 10.2 Hz), 120.1, 99.0 (d, *J* = 201.4 Hz), 52.2 (d, *J* = 20.1 Hz).

**$^{19}$F NMR (376 MHz, $CDCl_3$):** δ -151.35 – -151.58 (m, 2F), -156.85 (t, *J* = 21.7 Hz, 1F), -161.66 – -161.91 (m, 2F), -173.81 (d, *J* = 31.6 Hz, 1F).

**HRMS (EI):** m/z calcd for $[M]^+$ $C_{21}H_{12}F_6O_2S$: 442.0462. Found: 442.0467.

**(2*R*,3*S*)-2-Fluoro-2-phenyl-3-(thiophen-2-yl)pent-4-enamide ((*R*,*S*)-25-$NH_2$):**

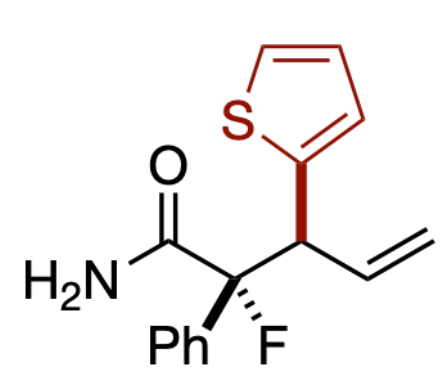


**IR (film):** 3431 (m), 3179 (br), 1670 (s), 1607 (w), 1448 (w), 1395 (w) cm$^{-1}$.

**$^1$H NMR (500 MHz, $CDCl_3$):** δ 7.55 – 7.42 (m, 2H), 7.30 – 7.21 (m, 3H), 7.05 (dd, *J* = 5.2, 1.2 Hz, 1H), 6.76 (dd, *J* = 5.1, 3.5 Hz, 1H), 6.67 (d, *J* = 3.5 Hz, 1H), 6.41 (s, 1H), 6.22

(ddd, $J$ = 17.0, 10.3, 8.4 Hz, 1H), 5.53 (s, 1H), 5.32 (d, $J$ = 16.9 Hz, 1H), 5.27 (d, $J$ = 10.2 Hz, 1H), 4.71 (dd, $J$ = 32.0, 9.0 Hz, 1H).

**$^{13}$C NMR (126 MHz, CDCl$_3$):** δ 172.1 (d, $J$ = 22.7 Hz), 139.4, 136.6 (d, $J$ = 22.0 Hz), 134.8 (d, $J$ = 5.2 Hz), 128.4, 128.1 (d, $J$ = 2.1 Hz), 126.8, 126.4, 124.9 (d, $J$ = 10.5 Hz), 124.8, 119.6, 101.0 (d, $J$ = 197.8 Hz), 52.5 (d, $J$ = 20.5 Hz).

**$^{19}$F NMR (376 MHz, CDCl$_3$):** δ -171.63 (d, $J$ = 34.0 Hz, 1F).

**HRMS (ESI):** m/z calcd for $[M+Na]^+$ $C_{15}H_{14}ONFNaS$: 298.0672. Found: 298.0673.

HPLC analysis using a chiral column (Chiralpak IB 3μ column 22 ˚C, 1.0 ml/min, 95:5 hexane:isopropanol, 210 nm, $t_{major}$ = 11.403 min, $t_{minor}$ = 25.033 min, t = 17.913 min, t = 29.955 min).

(±)-**25:** (*rac*)-**[Ir]** and (*rac*)-Bn-BTM

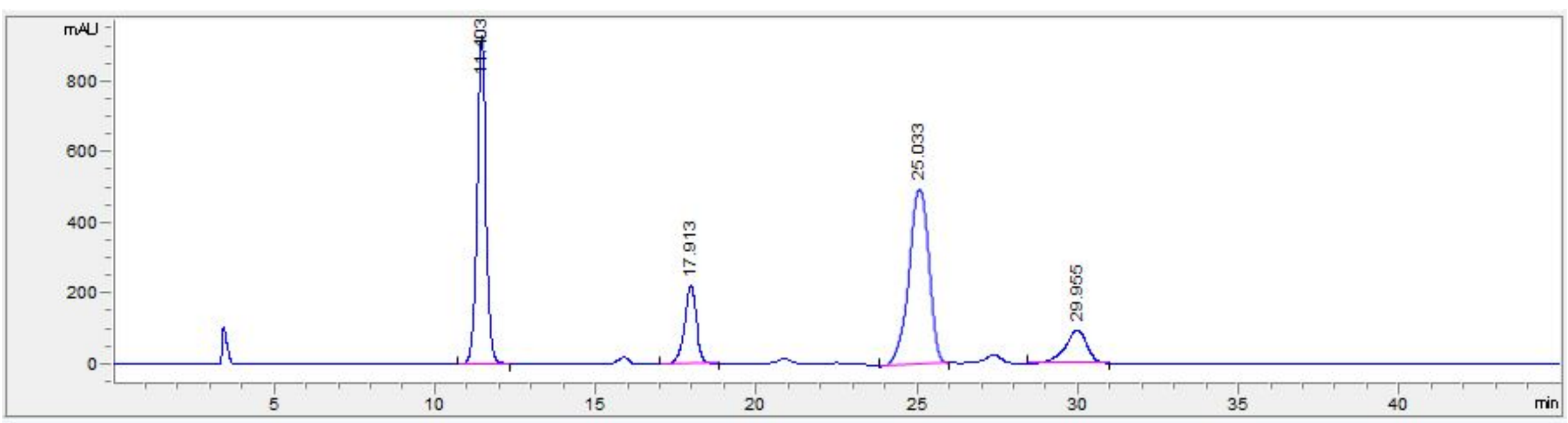


| # | Time | Area | Height | Width | Area% | Symmetry |
|---|---|---|---|---|---|---|
| 1 | 11.403 | 19204.3 | 927.2 | 0.3116 | 37.397 | 0.798 |
| 2 | 17.913 | 5919.8 | 222.2 | 0.3988 | 11.528 | 0.995 |
| 3 | 25.033 | 21698.6 | 497.2 | 0.6812 | 42.255 | 1.122 |
| 4 | 29.955 | 4529.2 | 95.3 | 0.7082 | 8.820 | 1.284 |

(*R*,*S*)-**25:** (*R*$_a$,*R*,*R*)-**[Ir]** and (*S*)-Me-BTM

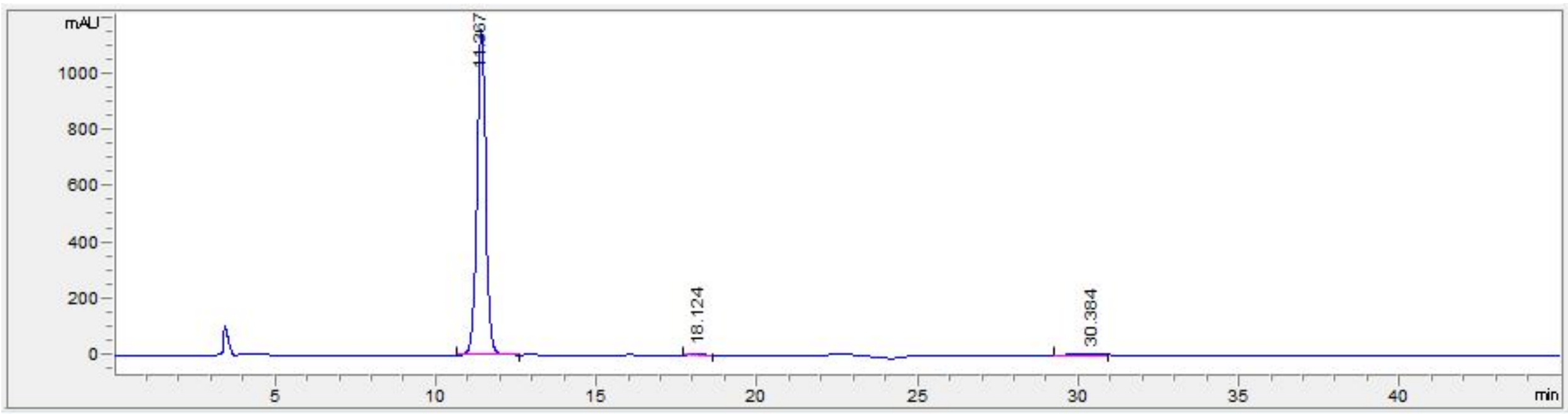


| # | Time | Area | Height | Width | Area% | Symmetry |
|---|---|---|---|---|---|---|
| 1 | 11.367 | 23552 | 1156.1 | 0.3057 | 94.226 | 0.773 |
| 2 | 18.124 | 420.1 | 10.6 | 0.6591 | 1.681 | 0.498 |
| 3 | 30.384 | 1023.1 | 12 | 1.425 | 4.093 | 1.77 |

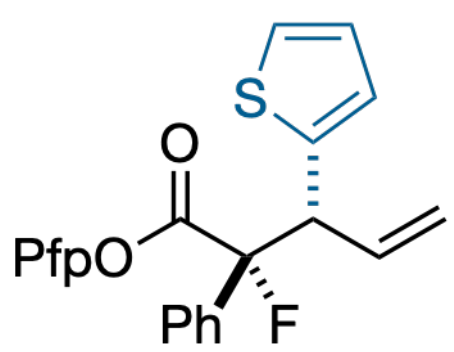


**Perfluorophenyl (2*R*,3*R*)-2-fluoro-2-phenyl-3-(thiophen-2-yl)pent-4-enoate ((*R*,*R*)-25):** Prepared according to General Procedure B using pentafluorophenyl ester **1a** (32 mg, 0.1 mmol, 1 equiv.), *t*-butyl carbonate **2k** (36.1 mg, 0.15 mmol, 1.5 equiv.), ($S_a$,*S*,*S*)-**[Ir]** (4.2 mg, 4 mol%), (*S*)-Me-BTM (3.8 mg, 20 mol%) and DIPEA (22.6 µL, 0.13 mmol, 1.3 equiv.) in 1,4-dioxane (0.3 ml, 0.3 M). The *title compound* was obtained (35.3 mg, 0.080 mmol, 80%) as a colorless oil following purification by column chromatography [$SiO_2$, 0.25% Ethyl Acetate in Petroleum Ether]. The enantiomeric ratio (>99:1 *er*) of the corresponding primary amide was determined by chiral HPLC in comparison with the racemate. Diastereomeric ratio (93:7) was determined by $^{19}$F-NMR analysis of the crude reaction mixture.

**IR (film):** 3073 (w), 1799 (w), 1781 (m), 1640 (w), 1517 (s), 1471 (w), 1195 (m), 1008 (s), 996 (s), 746 (m), 695 (s) cm$^{-1}$.

**$^1$H NMR (500 MHz, CDCl$_3$):** δ 7.73 – 7.62 (m, 2H), 7.51 – 7.40 (m, 3H), 7.31 (dd, *J* = 5.2, 1.2 Hz, 1H), 7.18 (dd, *J* = 3.3, 1.7 Hz, 1H), 7.03 (dd, *J* = 5.2, 3.5 Hz, 1H), ***6.32 (dt, J = 17.0, 9.5 Hz)**, 5.88 (ddd, *J* = 17.5, 10.3, 7.9 Hz, 1H), ***5.43 (d, J = 17.0 Hz)**, ***5.38 (d, J = 10.2 Hz)**, 5.08 (d, *J* = 10.2 Hz, 1H), 4.98 (d, *J* = 17.0 Hz, 1H), 4.79 (dd, *J* = 33.0, 7.5 Hz, 1H).

**$^{13}$C NMR (126 MHz, CDCl$_3$):** δ 165.8 (d, *J* = 28.5 Hz), 138.9, 135.1 (d, *J* = 22.5 Hz), 133.5 (d, *J* = 4.5 Hz), 129.5, 128.9 (d, *J* = 2.0 Hz), 127.3 (d, *J* = 2.4 Hz), 127.0, 125.8, 125.2 (d, *J* = 10.2 Hz), 120.1, 99.0 (d, *J* = 201.4 Hz), 52.2 (d, *J* = 20.1 Hz).

**$^{19}$F NMR (376 MHz, CDCl$_3$):** δ -151.70 – -151.89 (m, 2F), -157.10 (t, *J* = 21.7 Hz, 1F), -161.88 – -162.08 (m, 2F), -173.37 (d, *J* = 33.3 Hz, 1F).

**HRMS (EI):** m/z calcd for $[M]^+$ $C_{21}H_{12}F_6O_2S$: 442.0462. Found: 442.0466.

**(2*R*,3*R*)-2-Fluoro-2-phenyl-3-(thiophen-2-yl)pent-4-enamide ((*R*,*R*)-25-NH$_2$):**

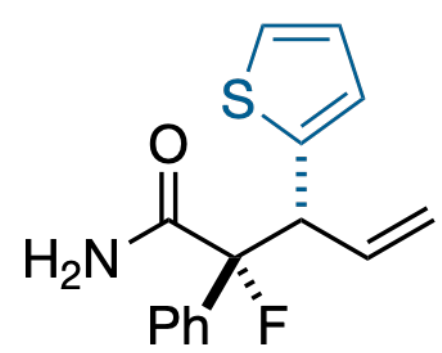


**IR (film):** 3435 (m), 3180 (br), 2923 (w), 1668 (s), 1603 (w), 1494 (w), 1448 (w), 1398 (w) cm$^{-1}$.

**$^1$H NMR (500 MHz, CDCl$_3$):** δ 7.71 – 7.60 (m, 2H), 7.40 (dd, *J* = 8.4, 6.7 Hz, 2H), 7.37 – 7.31 (m, 1H), 7.26 (d, *J* = 4.1 Hz, 1H), 7.08 (d, *J* = 3.4 Hz, 1H), 6.99 (dd, *J* = 5.1, 3.5 Hz, 1H), 6.17 (d, *J* = 5.1 Hz, 1H), 5.84 (ddd, *J* = 16.9, 10.3, 8.2 Hz, 1H), ***5.31 (d, J = 17.0 Hz)**, ***5.27 (d, J = 10.2 Hz)**, 5.20 (s, 1H), 5.00 (dt, *J* = 10.3, 1.1 Hz, 1H), 4.91 (d, *J* = 17.0 Hz, 1H), 4.76 (dd, *J* = 35.5, 8.5 Hz 1H).
**$^{13}$C NMR (126 MHz, CDCl$_3$):** δ 172.0 (d, *J* = 23.0 Hz), 140.0, 136.7 (d, *J* = 22.0 Hz), 134.2 (d, *J* = 4.4 Hz), 128.6, 128.5 (d, *J* = 2.3 Hz), 127.3, 126.7, 125.2, 125.0 (d, *J* = 10.8 Hz), 119.5, 100.7 (d, *J* = 198.6 Hz), 52.0 (d, *J* = 19.8 Hz).

**$^{19}$F NMR (376 MHz, CDCl$_3$):** δ -172.39 (d, *J* = 35.1 Hz).
**HRMS (ESI):** m/z calcd for $[M+Na]^+$ $C_{15}H_{14}ONFNaS$: 298.0672. Found: 298.0673.

HPLC analysis using a chiral column (Chiralpak IB 3μ column 22 ˚C, 1.0 ml/min, 95:5 hexane:isopropanol, 210 nm, $t_{major}$ = 17.913 min, $t_{minor}$ = 29.955 min, t = 11.403 min, t = 25.033 min).

(±)-**25:** (*rac*)-**[Ir]** and (*rac*)-Bn-BTM

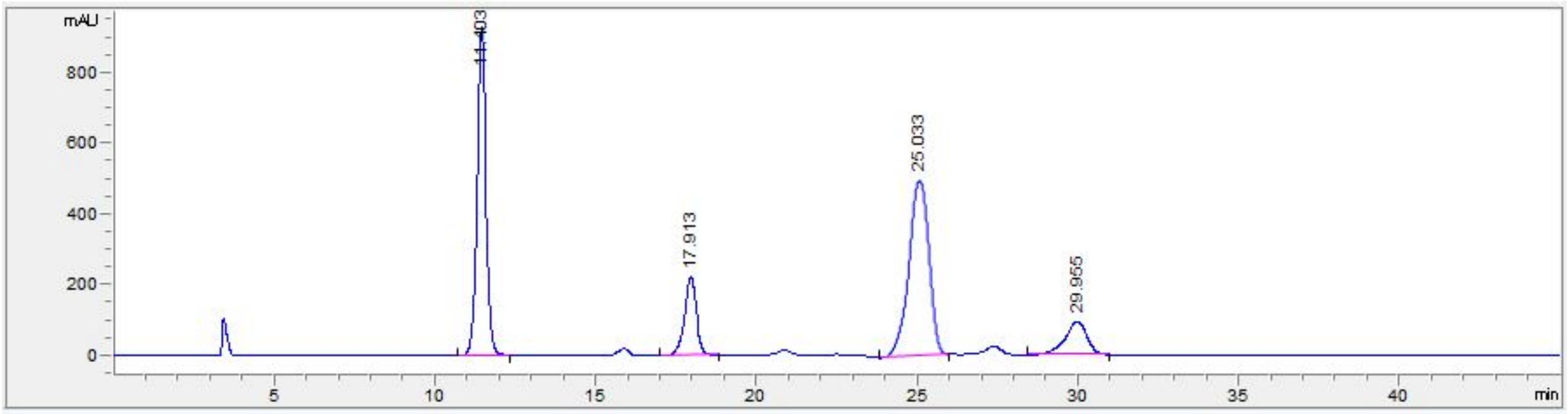


| # | Time | Area | Height | Width | Area% | Symmetry |
|---|---|---|---|---|---|---|
| 1 | 11.403 | 19204.3 | 927.2 | 0.3116 | 37.397 | 0.798 |
| 2 | 17.913 | 5919.8 | 222.2 | 0.3988 | 11.528 | 0.995 |
| 3 | 25.033 | 21698.6 | 497.2 | 0.6812 | 42.255 | 1.122 |
| 4 | 29.955 | 4529.2 | 95.3 | 0.7082 | 8.820 | 1.284 |

(*R*,*R*)-**25:** ($S_a$,*S*,*S*)-**[Ir]** and (*S*)-Me-BTM

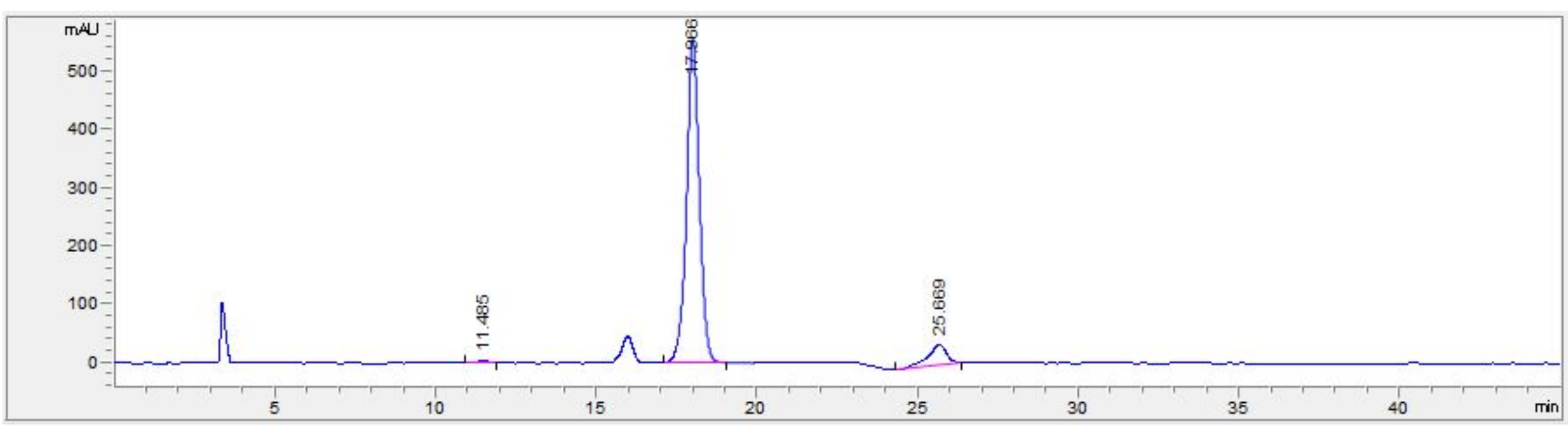


| # | Time | Area | Height | Width | Area% | Symmetry |
|---|---|---|---|---|---|---|
| 1 | 11.485 | 144.2 | 5.8 | 0.3632 | 0.837 | 1.565 |
| 2 | 17.966 | 15569.3 | 558.9 | 0.417 | 90.358 | 0.919 |
| 3 | 25.669 | 1517.2 | 35.2 | 0.617 | 8.805 | 1.797 |

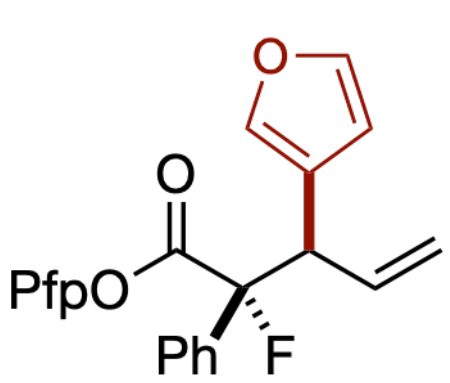


**Perfluorophenyl (2*R*,3*R*)-2-fluoro-3-(furan-3-yl)-2-phenylpent-4-enoate ((*R*,*R*)-24):** Prepared according to General Procedure B using pentafluorophenyl ester **1a** (32 mg, 0.1 mmol, 1 equiv.), *t*-butyl carbonate **2l** (33.6 mg, 0.15 mmol, 1.5 equiv.), ($R_a$,*R*,*R*)-**[Ir]** (4.2 mg, 4 mol%), (*S*)-Me-BTM (3.8 mg, 20 mol%) and DIPEA (22.6 μL, 0.13 mmol, 1.3 equiv.) in 1,4-dioxane (0.3 ml, 0.3 M). The *title compound* was obtained (32 mg, 0.075 mmol, 75%) as a colorless oil following purification by column chromatography [$SiO_2$, 0.25% Ethyl Acetate in Petroleum Ether]. The enantiomeric ratio (>99:1 *er*) was determined by chiral

HPLC in comparison with the racemate. Diastereomeric ratio (98:2) was determined by $^{19}$F-NMR analysis of the crude reaction mixture.

**IR (film):** 3066 (w), 1783 (m), 1641 (w), 1518 (s), 1225 (m), 1192 (m), 1009 (s), 996 (s), 854 (m), 744 (m), 695 (m), 602 (m) cm$^{-1}$.

**$^1$H NMR (500 MHz, CDCl$_3$):** δ 7.54 – 7.49 (m, 2H), 7.39 – 7.32 (m, 3H), 7.21 (d, *J* = 1.8 Hz, 1H), 7.09 (s, 1H), 6.23 (ddd, *J* = 17.1, 10.2, 8.7 Hz, 1H), 6.15 (d, *J* = 1.9 Hz, 1H), 5.42 – 5.33 (m, 2H), 4.40 (dd, *J* = 32.2, 8.7 Hz, 1H).

**$^{13}$C NMR (126 MHz, CDCl$_3$):** δ 166.26 (d, *J* = 28.6 Hz), 142.75, 141.01 (d, *J* = 1.7 Hz), 135.28 (d, *J* = 22.4 Hz), 133.78 (d, *J* = 4.7 Hz), 129.26, 128.65 (d, *J* = 2.0 Hz), 125.10 (d, *J* = 9.7 Hz), 120.28, 120.12, 110.84 (d, *J* = 1.9 Hz), 99.35 (d, *J* = 200.3 Hz), 48.66 (d, *J* = 20.2 Hz).

**$^{19}$F NMR (376 MHz, CDCl$_3$):** δ -151.45 – -151.60 (m, 2F), -156.91 (t, *J* = 21.6 Hz, 1F), -161.62 – -162.00 (m, 2F), -173.78 (d, *J* = 32.2 Hz, 1F).

**HRMS (EI):** m/z calcd for $[M]^+$ $C_{21}H_{12}F_6O_3$: 426.0691. Found: 426.0698.

**(2*R*,3*R*)-2-fluoro-3-(furan-3-yl)-2-phenylpent-4-enamide ((*R*,*R*)-24-NH$_2$):**

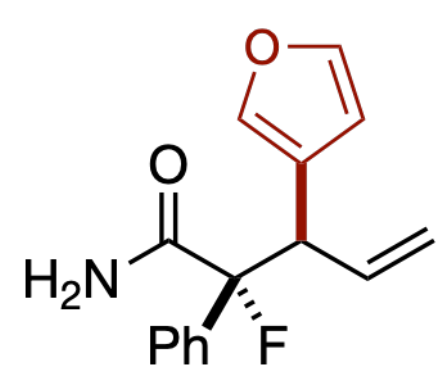


**IR (film):** 3427 (m), 3180 (br), 1669 (s), 1609 (w), 1503 (m), 1448 (m), 1397 (m) cm$^{-1}$.

**$^1$H NMR (500 MHz, CDCl$_3$):** δ 7.52 – 7.45 (m, 2H), 7.31 – 7.23 (m, 3H), 7.18 (t, *J* = 1.7 Hz, 1H), 7.02 (s, 1H), 6.40 (s, 1H), 6.20 – 6.08 (m, 2H), 5.50 (s, 1H), 5.31 – 5.21 (m, 2H), 4.35 (dd, *J* = 34.5, 8.1 Hz, 1H).

**$^{13}$C NMR (126 MHz, CDCl$_3$):** δ 172.33 (d, *J* = 23.0 Hz), 142.45, 140.91, 137.05 (d, *J* = 22.0 Hz), 134.50 (d, *J* = 4.6 Hz), 128.37, 128.18 (d, *J* = 2.1 Hz), 124.82 (d, *J* = 10.5 Hz), 120.93, 119.32, 111.02 (d, *J* = 1.8 Hz), 101.12 (d, *J* = 196.8 Hz), 48.19 (d, *J* = 20.6 Hz).

**$^{19}$F NMR (376 MHz, CDCl$_3$):** δ -171.88 (d, *J* = 34.4 Hz).

**HRMS (ESI):** m/z calcd for $[M+Na]^+$ $C_{15}H_{14}FNO_2Na$: 282.0901. Found: 282.0893.

HPLC analysis using a chiral column (Chiralpak IB 3μ column 22 ˚C, 1.0 ml/min, 95:5 hexane:isopropanol, 210 nm, $t_{major}$ = 12.03 min, $t_{minor}$ = 34.401 min, t = 19.20 min, t = 28.019 min).

(±)-**24:** (*rac*)-**[Ir]** and (*rac*)-Bn-BTM

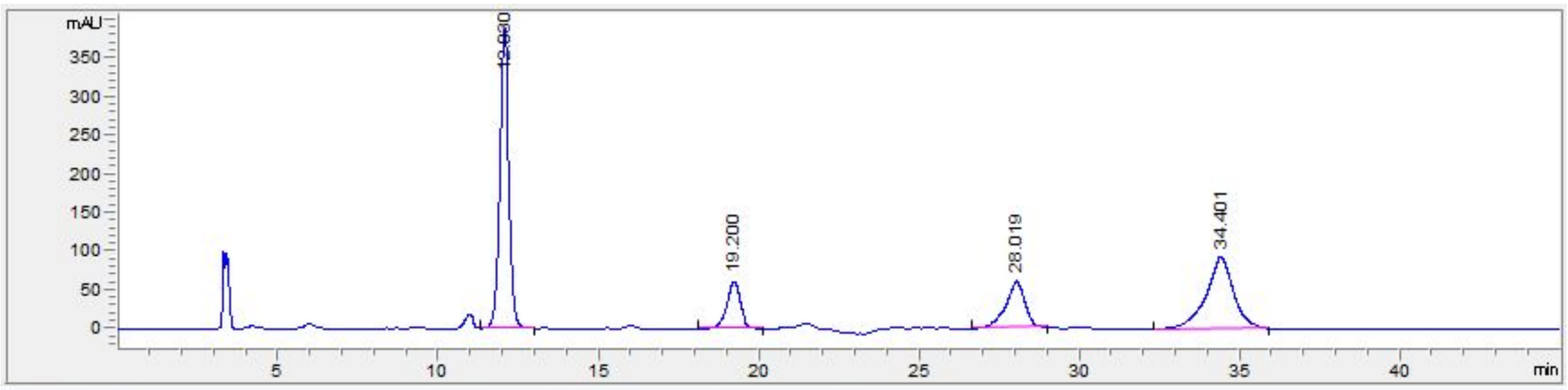


| # | Time | Area | Height | Width | Area% | Symmetry |
|---|---|---|---|---|---|---|
| 1 | 12.03 | 8128.9 | 387.6 | 0.3227 | 44.991 | 0.871 |
| 2 | 19.2 | 1796.2 | 62 | 0.434 | 9.942 | 1.134 |
| 3 | 28.019 | 2571.7 | 60.3 | 0.6416 | 14.234 | 1.339 |
| 4 | 34.401 | 5570.9 | 92.1 | 0.8775 | 30.833 | 1.206 |

(*R*,*R*)-**24:** (*R<sub></sub>a*,*R*,*R*)-**[Ir]** and (*S*)-Me-BTM

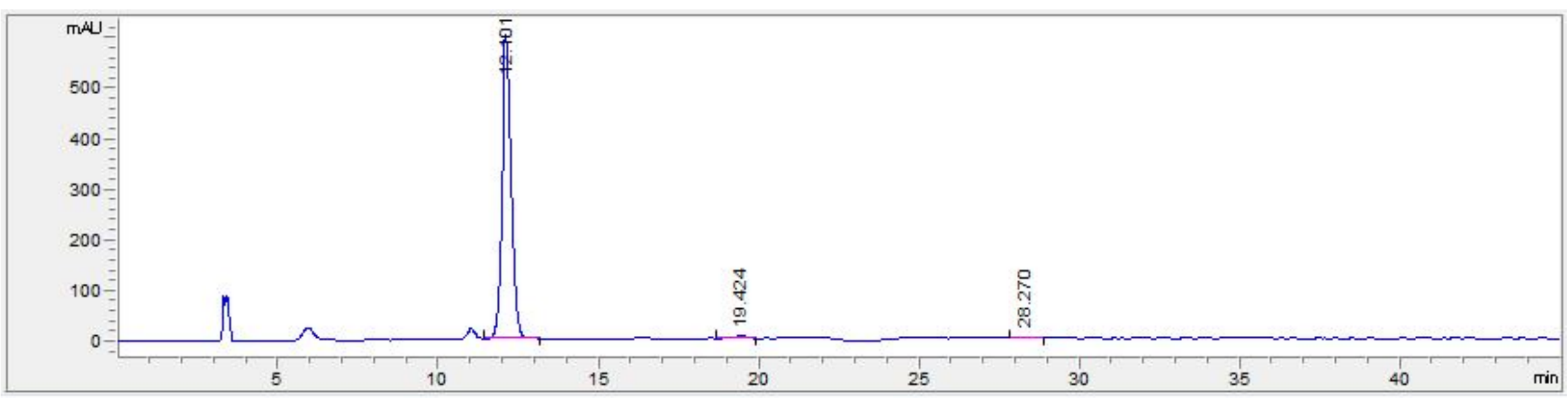


| # | Time | Area | Height | Width | Area% | Symmetry |
|---|---|---|---|---|---|---|
| 1 | 12.101 | 12754.8 | 602.6 | 0.323 | 98.163 | 0.812 |
| 2 | 19.424 | 168.5 | 5.6 | 0.4486 | 1.296 | 1.418 |
| 3 | 28.27 | 70.3 | 2.3 | 0.4002 | 0.541 | 0.634 |

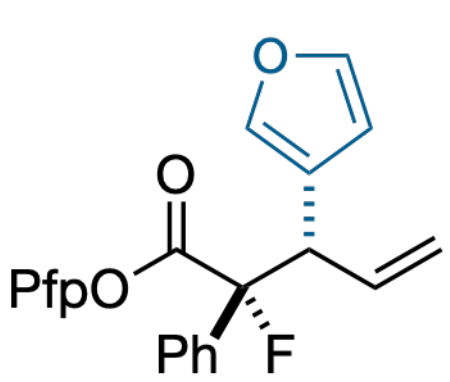


**Perfluorophenyl (2*R*,3*S*)-2-fluoro-3-(furan-3-yl)-2-phenylpent-4-enoate ((*R*,*S*)-24):** Prepared according to General Procedure B using pentafluorophenyl ester **1a** (32 mg, 0.1 mmol, 1 equiv.), *t*-butyl carbonate **2l** (33.6 mg, 0.15 mmol, 1.5 equiv.), ($S_a$,*S*,*S*)-**[Ir]** (4.2 mg, 4 mol%), (*S*)-Me-BTM (3.8 mg, 20 mol%) and DIPEA (22.6 μL, 0.13 mmol, 1.3 equiv.) in

1,4-dioxane (0.3 ml, 0.3 M). The *title compound* was obtained (35 mg, 0.082 mmol, 82%) as a colorless oil following purification by column chromatography [$SiO_2$, 0.25% Ethyl Acetate in Petroleum Ether]. The enantiomeric ratio (>99:1 *er*) was determined by chiral HPLC in comparison with the racemate. Diastereomeric ratio (92:8) was determined by $^{19}$F-NMR analysis of the crude reaction mixture.

**IR (film):** 3069 (w), 1799 (m), 1781 (m), 1641 (w), 1517 (s), 1211 (m), 1193 (m), 1025 (m), 1008 (s), 995 (s), 929 (m), 874 (m), 742 (m), 695 (m), 602 (m) cm$^{-1}$.

**$^1$H NMR (500 MHz, CDCl$_3$):** δ 7.65 (dd, *J* = 7.4, 1.9 Hz, 2H), 7.54 (s, 1H), 7.49 – 7.41 (m, 4H), **7.09 (s, 0.1H)*, 6.50 (d, *J* = 1.9 Hz, 1H), ****6.23 (dt, J = 17.2, 9.5 Hz)***, 5.80 (ddd, *J* = 17.4, 10.3, 7.4 Hz, 1H), ****5.45 – 5.31 (m)***, 5.08 (d, *J* = 10.3 Hz, 1H), 4.99 (d, *J* = 17.1 Hz, 1H), 4.45 (dd, *J* = 34.0, 7.4 Hz, 1H).

**$^{13}$C NMR (126 MHz, CDCl$_3$):** δ 166.14 (d, *J* = 28.5 Hz), 143.29, 141.16 (d, *J* = 3.0 Hz), 135.18 (d, *J* = 22.5 Hz), 133.12 (d, *J* = 4.0 Hz), 129.42, 128.91 (d, *J* = 2.2 Hz), 125.21 (d, *J* = 10.1 Hz), 121.02, 119.84, 111.11 (d, *J* = 2.3 Hz), 99.20 (d, *J* = 200.4 Hz), 47.87 (d, *J* = 20.2 Hz).

**$^{19}$F NMR (376 MHz, CDCl$_3$):** δ -151.98 – -152.13 (m, 2F), -157.05 (t, *J* = 21.7 Hz, 1F), -161.79 – -162.02 (m, 2F), -173.93 (d, *J* = 33.9 Hz, 1F).

**HRMS (EI):** m/z calcd for $[M]^+$ $C_{21}H_{12}F_6O_3$: 426.0691. Found: 426.0691.

**(2*R*,3*S*)-2-Fluoro-3-(furan-3-yl)-2-phenylpent-4-enamide ((*R*,*S*)-24-NH$_2$):**

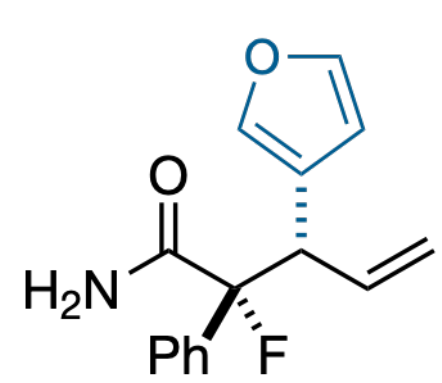


**IR (film):** 3436 (m), 3180 (br), 1666 (s), 1604 (w), 1502 (w), 1448 (w), 1400 (w) cm$^{-1}$.

**$^1$H NMR (500 MHz, CDCl$_3$):** δ 7.65 – 7.58 (m, 2H), 7.45 – 7.42 (m, 1H), 7.41 – 7.36 (m, 3H), 7.36 – 7.31 (m, 1H), 6.46 (d, *J* = 2.0 Hz, 1H), 6.18 (s, 1H), 5.75 (ddd, *J* = 17.0, 10.3, 7.8 Hz, 1H), 5.32 – 5.26 (m, 1H), 4.99 (dt, *J* = 10.3, 1.3 Hz, 1H), 4.91 (dt, *J* = 17.2, 1.5 Hz, 1H), 4.39 (dd, *J* = 36.0, 7.9 Hz, 1H).

**$^{13}$C NMR (126 MHz, $CDCl_3$):** δ 172.35 (d, *J* = 23.1 Hz), 142.71, 141.21 (d, *J* = 1.9 Hz), 136.94 (d, *J* = 21.8 Hz), 133.82 (d, *J* = 3.8 Hz), 128.50, 128.44 (d, *J* = 2.3 Hz), 124.95 (d, *J* = 10.8 Hz), 121.60, 119.16, 111.42 (d, *J* = 1.9 Hz), 100.98 (d, *J* = 197.6 Hz), 47.50 (d, *J* = 20.2 Hz).

**$^{19}$F NMR (376 MHz, $CDCl_3$):** δ -173.23 (d, *J* = 35.9 Hz, 1F).

**HRMS (ESI):** m/z calcd for $[M+Na]^+$ $C_{15}H_{14}FNO_2Na$: 282.0901. Found: 282.0892.

HPLC analysis using a chiral column (Chiralpak IB 3μ column 22 ˚C, 1.0 ml/min, 95:5 hexane:isopropanol, 210 nm, $t_{major}$ = 19.2 min, $t_{minor}$ = 28.019 min, t = 12.03 min, t = 34.401 min).

(±)-**24:** (*rac*)-**[Ir]** and (*rac*)-Bn-BTM

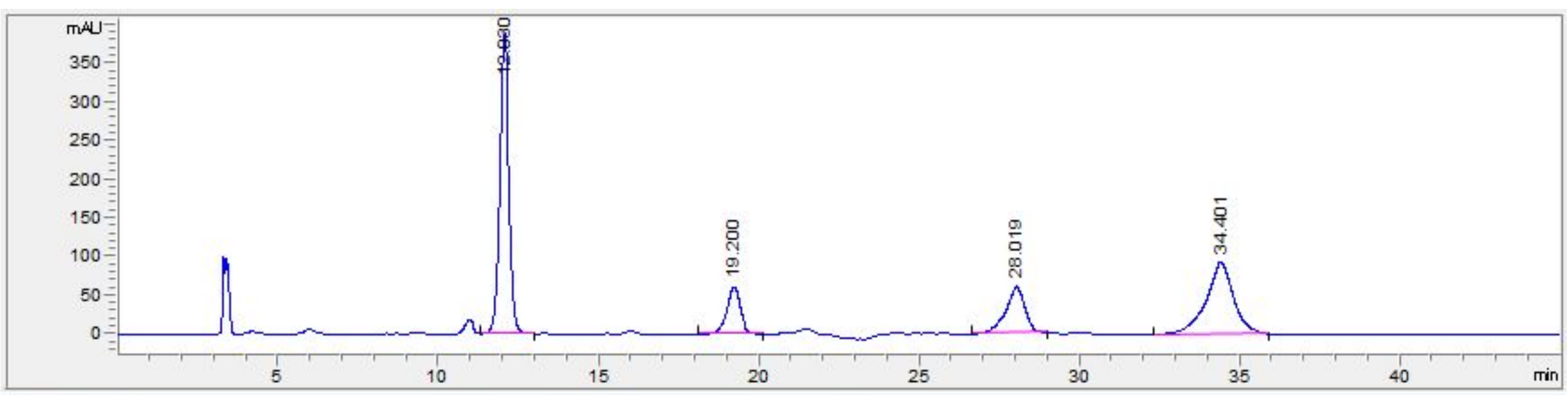


| # | Time | Area | Height | Width | Area% | Symmetry |
|---|---|---|---|---|---|---|
| 1 | 12.03 | 8128.9 | 387.6 | 0.3227 | 44.991 | 0.871 |
| 2 | 19.2 | 1796.2 | 62 | 0.434 | 9.942 | 1.134 |
| 3 | 28.019 | 2571.7 | 60.3 | 0.6416 | 14.234 | 1.339 |
| 4 | 34.401 | 5570.9 | 92.1 | 0.8775 | 30.833 | 1.206 |

(*R*,*S*)-**24:** (*S$_a$,S,S*)-**[Ir]** and (*S*)-Me-BTM

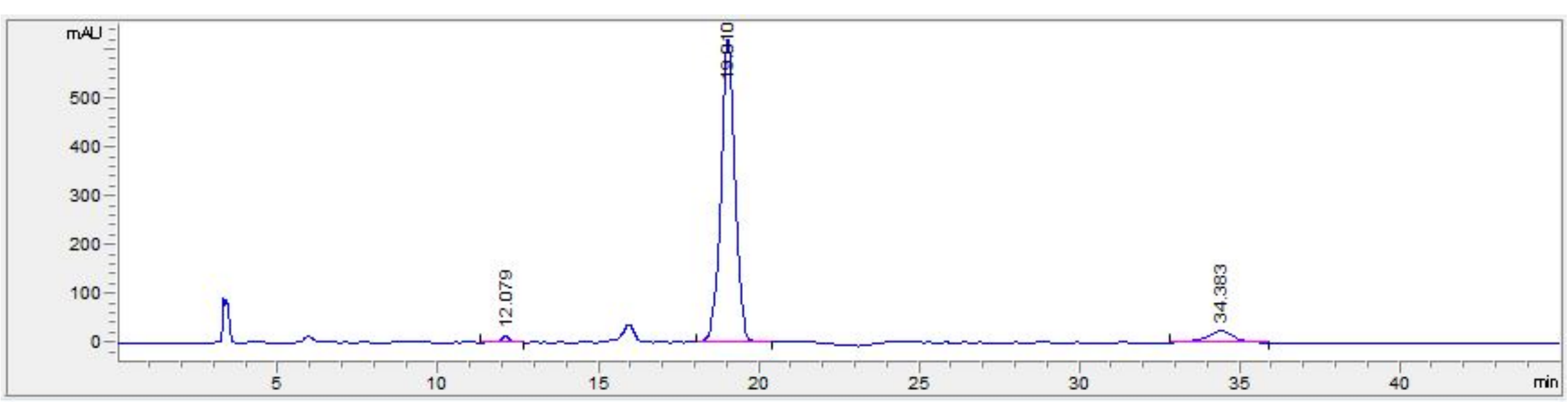


| # | Time | Area | Height | Width | Area% | Symmetry |
|---|---|---|---|---|---|---|
| 1 | 12.079 | 290.9 | 13.2 | 0.3322 | 1.462 | 1.114 |
| 2 | 19.01 | 18139.1 | 621 | 0.4348 | 91.180 | 0.927 |
| 3 | 34.383 | 1463.8 | 24.6 | 0.8309 | 7.358 | 1.11 |

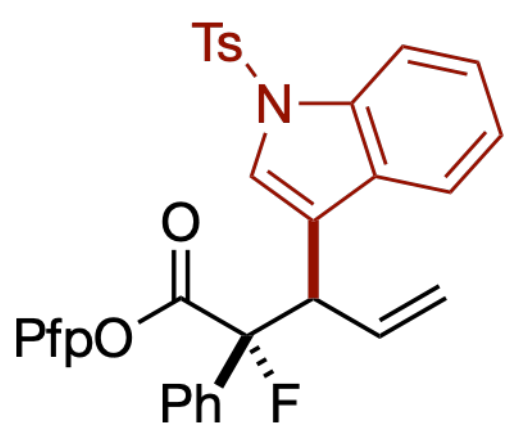


**Perfluorophenyl (2*R*,3*R*)-2-fluoro-2-phenyl-3-(1-tosyl-1H-indol-3-yl)pent-4-enoate ((*R*,*R*)-26):** Prepared according to General Procedure B using pentafluorophenyl ester **1a** (32 mg, 0.1 mmol, 1 equiv.), *t*-butyl carbonate **2m** (64.1 mg, 0.15 mmol, 1.5 equiv.), ($R_a$,*R*,*R*)-**[Ir]** (4.2 mg, 4 mol%), (*S*)-Me-BTM (3.8 mg, 20 mol%) and DIPEA (22.6 µL, 0.13 mmol, 1.3 equiv.) in 1,4-dioxane (0.3 ml, 0.3 M). The *title compound* was obtained (45.05 mg, 0.0715 mmol, 71.5%) as a colorless oil following purification by column chromatography [$SiO_2$, 0.25% Ethyl Acetate in Petroleum Ether]. The enantiomeric ratio (>99:1 *er*) was determined by chiral HPLC in comparison with the racemate. Diastereomeric ratio (94:6) was determined by $^{19}$F-NMR analysis of the crude reaction mixture.

**IR (film):** 3062 (w), 1792 (m), 1640 (w), 1598 (w), 1519 (s), 1370 (m), 1174 (s), 1011 (s), 996 (s) cm$^{-1}$.

**$^1$H NMR (500 MHz, $CDCl_3$):** δ 7.84 (d, *J* = 8.2 Hz, 1H), 7.47 (d, *J* = 7.8 Hz, 1H), 7.45 – 7.36 (m, 5H), 7.26 – 7.22 (m, 2H), 7.18 (td, *J* = 7.3, 5.2 Hz, 3H), 7.09 (d, *J* = 8.1 Hz, 2H), 6.25 (ddd, *J* = 17.1, 10.1, 8.4 Hz, 1H), 5.41 (d, *J* = 17.0 Hz, 1H), 5.36 (d, *J* = 10.2 Hz, 1H), 4.74 (dd, *J* = 34.0, 8.5 Hz, 1H), 2.33 (s, 3H).

**$^{13}$C NMR (126 MHz, $CDCl_3$):** δ 166.20 (d, *J* = 28.9 Hz), 144.76, 135.16, 135.08 (d, *J* = 22.6 Hz), 134.73, 133.06 (d, *J* = 4.5 Hz), 130.28, 129.89, 129.09, 128.66 (d, *J* = 1.8 Hz), 126.70, 125.65 (d, *J* = 4.5 Hz), 125.01 (d, *J* = 9.3 Hz), 124.85, 123.29, 120.67, 119.69 (d, *J* = 2.4 Hz), 117.46, 113.72, 99.70 (d, *J* = 200.4 Hz), 48.10 (d, *J* = 19.9 Hz), 21.71.

**$^{19}$F NMR (376 MHz, $CDCl_3$):** δ -151.50 – -151.66 (m, 2F), -156.70 (t, *J* = 21.7 Hz, 1F), -161.54 – -161.83 (m, 2F), -171.69 (d, *J* = 33.8 Hz, 1F).

**HRMS (APCI):** m/z calcd for $[M+H]^+$ $C_{32}H_{22}O_4NF_6S$: 630.1168. Found: 630.1170.

HPLC analysis using a chiral column (Cellulose 1 3µ column 22 ˚C, 0.5 ml/min, 99:1 hexane:isopropanol, 210 nm, $t_{major}$ = 31.660 min, $t_{minor}$ = 29..063 min, t = 23.998 min, t = 35.289 min).

**(±)26:** (*rac*)-**[Ir]** and (*rac*)-Bn-BTM

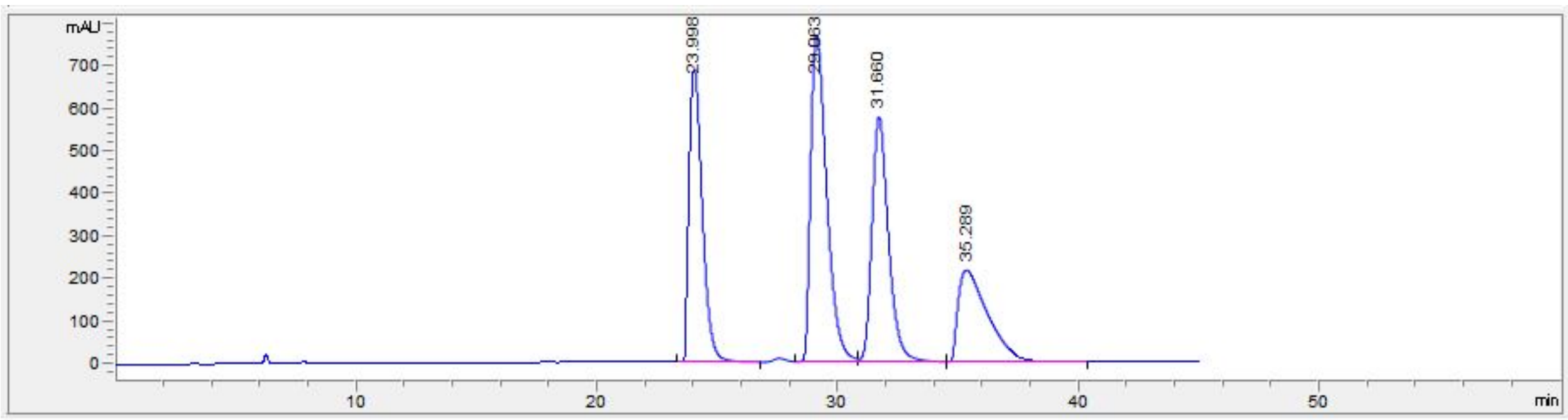


| # | Time | Area | Height | Width | Area% | Symmetry |
|---|---|---|---|---|---|---|
| 1 | 23.998 | 25123.2 | 688.1 | 0.568 | 23.521 | 0.557 |
| 2 | 29.063 | 35704.8 | 769.4 | 0.7234 | 33.427 | 0.529 |
| 3 | 31.66 | 27479.8 | 576.7 | 0.7279 | 25.727 | 0.703 |
| 4 | 35.289 | 18505.7 | 216.3 | 1.2162 | 17.325 | 0.368 |

**(*R*,*R*)-26:** ($R_a$,*R*,*R*)-**[Ir]** and (*S*)-Me-BTM

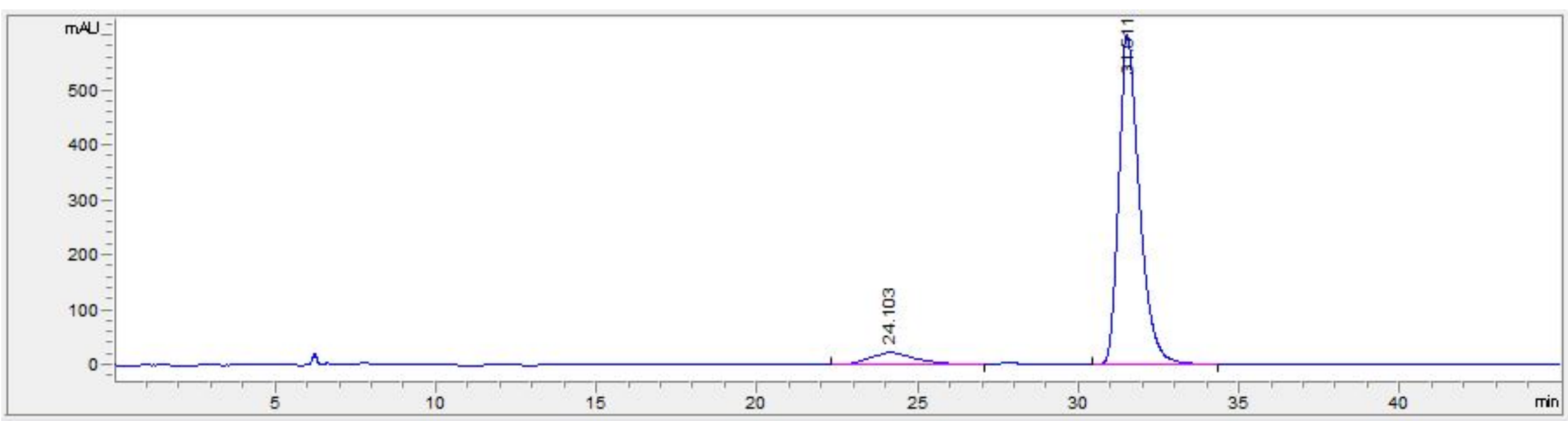


| # | Time | Area | Height | Width | Area% | Symmetry |
|---|---|---|---|---|---|---|
| 1 | 24.103 | 2265.9 | 22.6 | 1.2672 | 7.441 | 0.784 |
| 2 | 31.511 | 28184.6 | 599.3 | 0.7147 | 92.559 | 0.705 |

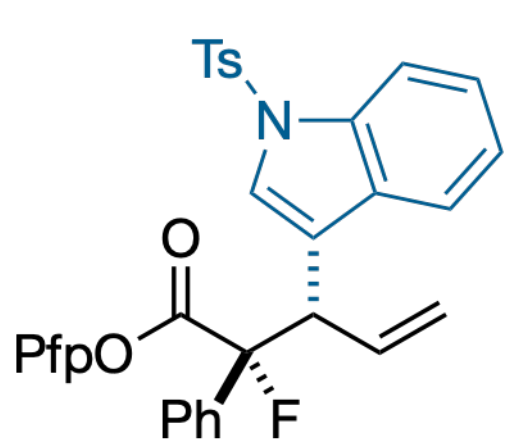


**Perfluorophenyl (2*R*,3*S*)-2-fluoro-2-phenyl-3-(1-tosyl-1H-indol-3-yl)pent-4-enoate ((*R*,*S*)-26):** Prepared according to General Procedure B using pentafluorophenyl ester **1a** (32 mg, 0.1 mmol, 1 equiv.), *t*-butyl carbonate **2m** (64.1 mg, 0.15 mmol, 1.5 equiv.), ($S_a$,*S*,*S*)-**[Ir]** (4.2 mg, 4 mol%), (*S*)-Me-BTM (3.8 mg, 20 mol%) and DIPEA (22.6 µL, 0.13 mmol, 1.3 equiv.) in 1,4-dioxane (0.3 ml, 0.3 M). The *title compound* was obtained (43.3 mg, 0.069 mmol, 69%) as a colorless oil following purification by column chromatography [$SiO_2$, 0.25% Ethyl Acetate in Petroleum Ether]. The enantiomeric ratio (>99:1 *er*) was

determined by chiral HPLC in comparison with the racemate. Diastereomeric ratio (97:3) was determined by $^{19}$F-NMR analysis of the crude reaction mixture.

**IR (film):** 3065 (w), 1796 (m), 1640 (w), 1598 (w), 1519 (s), 1370 (m), 1175 (m), 1010 (m), 997 (m) $cm^{-1}$.

**$^{1}$H NMR (500 MHz, $CDCl_3$):** δ 8.03 (d, *J* = 8.3 Hz, 1H), 7.88 (d, *J* = 2.9 Hz, 1H), 7.83 – 7.76 (m, 2H), 7.75 – 7.69 (m, 2H), 7.56 (d, *J* = 7.9 Hz, 1H), 7.50 (dd, *J* = 8.3, 6.4 Hz, 2H), 7.47 – 7.43 (m, 1H), 7.35 (ddd, *J* = 8.4, 7.2, 1.2 Hz, 1H), 7.28 – 7.22 (m, 1H), 7.16 (d, *J* = 8.2 Hz, 2H), 5.80 (ddd, *J* = 17.3, 10.2, 7.4 Hz, 1H), 5.05 (dd, *J* = 10.2, 1.1 Hz, 1H), 4.95 (dt, *J* = 17.0, 1.3 Hz, 1H), 4.73 (dd, *J* = 34.5, 7.5 Hz, 1H), 2.29 (s, 3H).

**$^{13}$C NMR (126 MHz, $CDCl_3$):** δ 165.6 (d, *J* = 29.0 Hz), 144.9, 135.3, 135.0 (d, *J* = 22.7 Hz), 134.9, 132.3 (d, *J* = 3.9 Hz), 130.3, 129.8, 129.6, 129.0 (d, *J* = 2.1 Hz), 127.2, 125.4 (d, *J* = 7.1 Hz), 125.2 (d, *J* = 9.5 Hz), 125.2, 123.4, 120.2, 119.3, 118.4, 113.8, 99.2 (d, *J* = 198.8 Hz), 47.3 (d, *J* = 20.3 Hz), 21.5.

**$^{19}$F NMR (376 MHz, $CDCl_3$):** δ -152.02 – -152.17 (m, 2F), -157.20 (t, *J* = 21.7 Hz, 1F), -161.81 – -162.04 (m, 2F), -171.85 (d, *J* = 34.8 Hz, 1F).

**HRMS (APCI):** m/z calcd for $[M+H]^+$ $C_{32}H_{22}O_4NF_6S$: 630.1168. Found: 630.1169.

HPLC analysis using a chiral column (Cellulose 1 3μ column 22 ˚C, 0.5 ml/min, 99:1 hexane:isopropanol, 210 nm, $t_{major}$ = 23.998 min, $t_{minor}$ = 35.289 min, t = 29..063 min, t = 31.660 min).

(±)-**26:** (*rac*)-**[Ir]** and (*rac*)-Bn-BTM

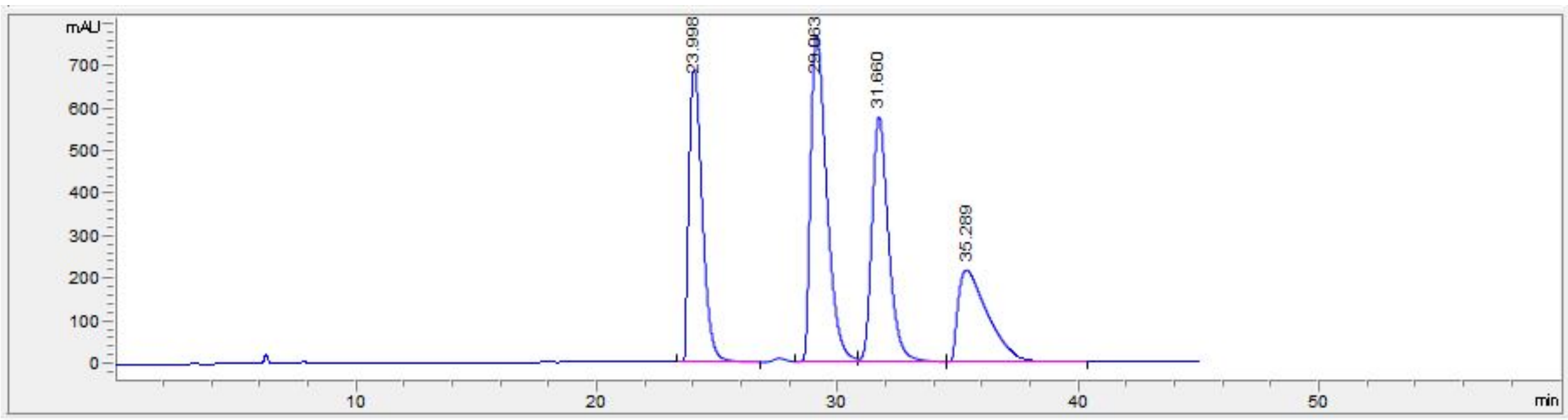


| # | Time | Area | Height | Width | Area% | Symmetry |
|---|---|---|---|---|---|---|
| 1 | 23.998 | 25123.2 | 688.1 | 0.568 | 23.521 | 0.557 |
| 2 | 29.063 | 35704.8 | 769.4 | 0.7234 | 33.427 | 0.529 |
| 3 | 31.66 | 27479.8 | 576.7 | 0.7279 | 25.727 | 0.703 |
| 4 | 35.289 | 18505.7 | 216.3 | 1.2162 | 17.325 | 0.368 |

(*R*,*S*)-**26:** ($S_a$,*S*,*S*)-**[Ir]** and (*S*)-Me-BTM

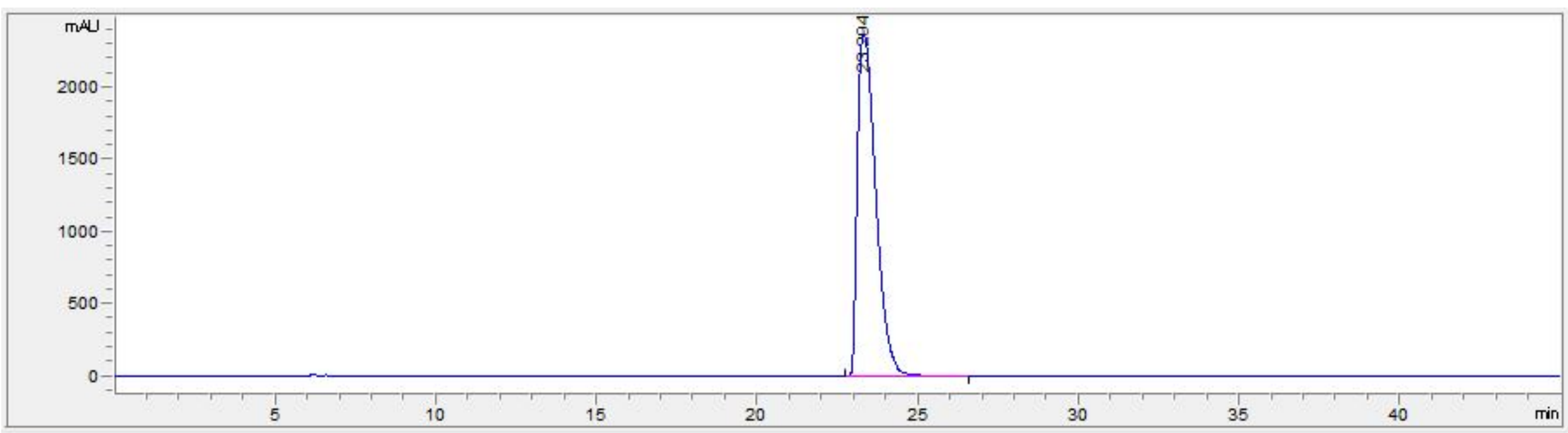


| # | Time | Area | Height | Width | Area% | Symmetry |
|---|---|---|---|---|---|---|
| 1 | 23.294 | 93210.1 | 2369.8 | 0.6189 | 100.000 | 0.493 |

## Product transformations

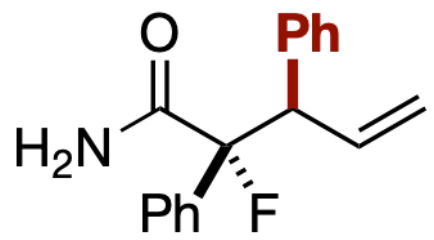

**(2*R*,3*R*)-2-fluoro-2,3-diphenylpent-4-enamide ((*R*,*R*)-29):** Pentafluorophenyl ester (*R*,*R*)-**3** (57.2 mg, 0.13 mmol, 1 equiv.) was dissolved in THF (1.3 ml, 0.1 M) in a 1-dram vial equipped with stirrer bar. $NH_4OH$ (0.26 ml) was added and the reaction mixture was stirred at room temperature. After 1 hour, the reaction mixture was diluted with EtOAc and transferred to a separatory funnel. The organic layer was washed sequentially with $Na_2CO_3$ (2M aq.) (3 x 10mL) and brine (10 ml). The organic layer was dried with anhydrous $Na_2SO_4$, filtered and the solvent was removed under reduced pressure. The title compound was obtained (34.7 mg, 0.13 mmol, 99%) as white solid following purification by column chromatography [$SiO_2$, 8% to 25% Ethyl Acetate in Petroleum Ether]. The enantiomeric ratio (>99:1 *er*) was determined by chiral HPLC in comparison with the racemate.

**IR (film):** 3436 (m), 3178 (br), 1672 (s), 1602 (w), 1493 (w), 1448 (w), 1393 (w) $cm^{-1}$.

**$^1$H NMR (500 MHz, $CDCl_3$):** δ 7.49 – 7.36 (m, 2H), 7.23 – 7.14 (m, 3H), 7.14 – 7.03 (m, 5H), 6.43 (s, 1H), 6.30 (ddd, *J* = 16.9, 10.6, 8.4 Hz, 1H), 5.56 (s, 1H), 5.41 – 5.17 (m, 2H), 4.39 (dd, *J* = 34.5, 8.0 Hz, 1H).

**$^{13}$C NMR (126 MHz, $CDCl_3$):** δ 172.5 (d, *J* = 23.0 Hz), 137.7, 136.9 (d, *J* = 22.1 Hz), 135.2 (d, *J* = 5.4 Hz), 129.6 (d, *J* = 2.3 Hz), 128.1, 128.0 (d, *J* = 2.1 Hz), 126.9, 124.8 (d, *J* = 10.5 Hz), 119.2, 101.6 (d, *J* = 197.4 Hz), 57.1 (d, *J* = 19.4 Hz).

**$^{19}$F NMR (376 MHz, $CDCl_3$):** δ -171.59 (d, *J* = 34.8 Hz, 1F).

**HRMS (ESI):** m/z calcd for $[M+Na]^+$ $C_{17}H_{16}ONFNa$: 292.1108. Found: 292.1109.

HPLC analysis using a chiral column (Chiralpak IB 3μ column 22 ˚C, 1.0 ml/min, 95:5 hexane:isopropanol, 210 nm, $t_{major}$ = 10.794 min, $t_{minor}$ = 28.363 min, t = 15.735 min, t = 21.053 min).

(±)-**29:** (*rac*)-**[Ir]** and (*rac*)-Bn-BTM

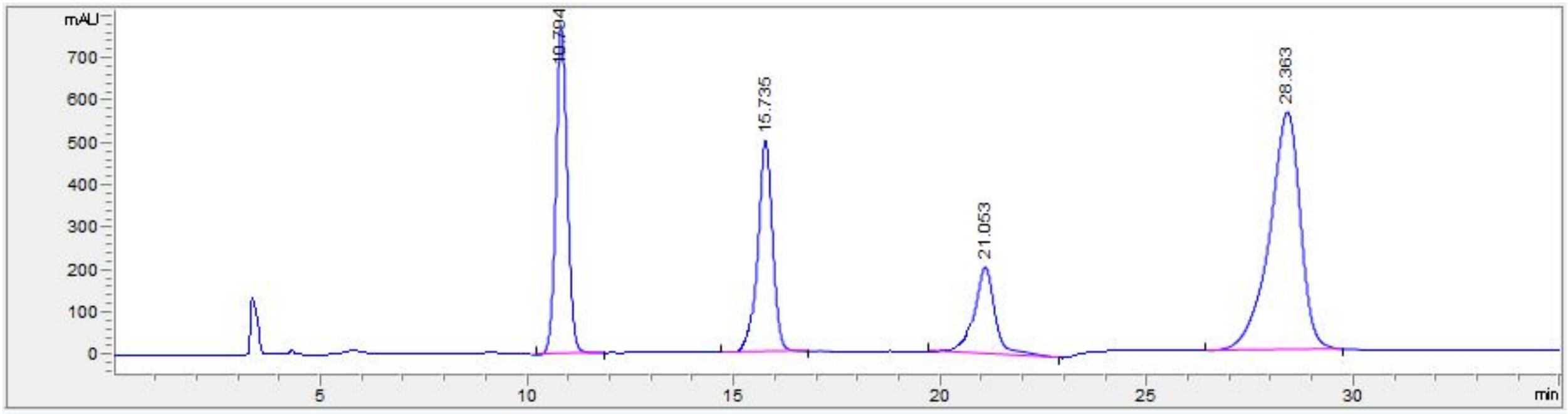


| # | Time | Area | Height | Width | Area% | Symmetry |
|---|---|---|---|---|---|---|
| 1 | 10.794 | 14739.8 | 772.4 | 0.2906 | 23.254 | 0.842 |
| 2 | 15.735 | 12193.8 | 496.6 | 0.3705 | 19.237 | 1.041 |
| 3 | 21.053 | 7369.3 | 204.8 | 0.5197 | 11.626 | 1.05 |
| 4 | 28.363 | 29083 | 559.5 | 0.7844 | 45.882 | 1.389 |

(*R*,*R*)-**29:** ($R_a$,*R*,*R*)-**[Ir]** and (*S*)-Me-BTM

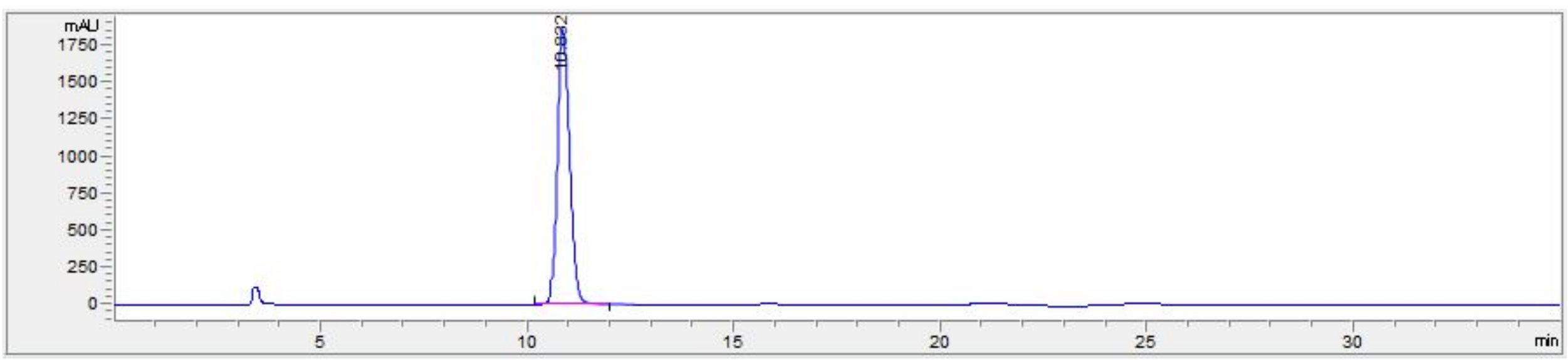


| # | Time | Area | Height | Width | Area% | Symmetry |
|---|---|---|---|---|---|---|
| 1 | 10.832 | 38095.2 | 1852.4 | 0.3199 | 100.000 | 0.709 |

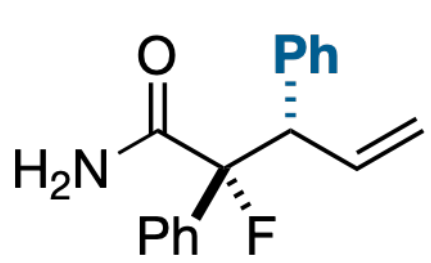


**(2*R*,3*S*)-2-fluoro-2,3-diphenylpent-4-enamide ((*R*,*S*)-29):** Pentafluorophenyl ester (*R*,*S*)-**3** (48.7 mg, 0.11 mmol, 1 equiv.) was dissolved in THF (1.1 ml, 0.1 M) in a 1-dram vial equipped with stirrer bar. $NH_4OH$ (0.22 ml) was added and the reaction mixture was stirred at room temperature. After 1 hour, the reaction mixture was diluted with EtOAc and transferred to a separatory funnel. The organic layer was washed sequentially with $Na_2CO_3$ (2M aq.) (3 x 10mL) and brine (10 ml). The organic layer was dried with anhydrous $Na_2SO_4$, filtered and the solvent was removed under reduced pressure. The title compound was obtained (24.2 mg, 0.09 mmol, 82%) as white solid following purification by column chromatography [$SiO_2$, 8% to 50% Ethyl Acetate in Petroleum Ether]. The enantiomeric ratio (>99:1 *er*) was determined by chiral HPLC in comparison with the racemate.

**IR (film):** 3436 (m), 3178 (br), 2922 (w), 1666 (s), 1602 (w), 1492 (w), 1447 (w), 1398 (w) $cm^{-1}$.

**$^{1}$H NMR (500 MHz, DMSO):** δ 7.65 – 7.56 (m, 2H), 7.49 (s, 1H), 7.44 – 7.36 (m, 4H), 7.36 – 7.27 (m, 3H), 7.27 – 7.20 (m, 1H), 7.13 (d, *J* = 4.5 Hz, 1H), 5.88 (ddd, *J* = 17.1, 10.3, 8.7 Hz, 1H), 4.93 – 4.88 (m, 1H), 4.87 – 4.81 (m, 1H), 4.45 (dd, *J* = 35.5, 8.5 Hz, 1H).

**$^{13}$C NMR (126 MHz, DMSO):** δ 170.9 (d, *J* = 23.5 Hz), 139.0, 138.3 (d, *J* = 22.5 Hz), 135.6 (d, *J* = 4.6 Hz), 129.6 (d, *J* = 2.6 Hz), 128.0, 127.8, 126.7, 124.9 (d, *J* = 10.6 Hz), 118.5, 99.9 (d, *J* = 199.9 Hz), 55.1 (d, *J* = 18.6 Hz).

**$^{19}$F NMR (376 MHz, DMSO):** δ -172.15 – -172.29 (m, 1F).

**HRMS (ESI):** m/z calcd for $[M+Na]^+$ $C_{17}H_{16}ONFNa$: 292.1108. Found: 292.1109.

HPLC analysis using a chiral column (Chiralpak IB 3μ column 22 ˚C, 1.0 ml/min, 95:5 hexane:isopropanol, 210 nm, $t_{major}$ = 15.735 min, $t_{minor}$ = 21.053 min, t = 10.794 min, t = 28.363 min).

(+)-**29:** (*rac*)-**[Ir]** and (*rac*)-Bn-BTM

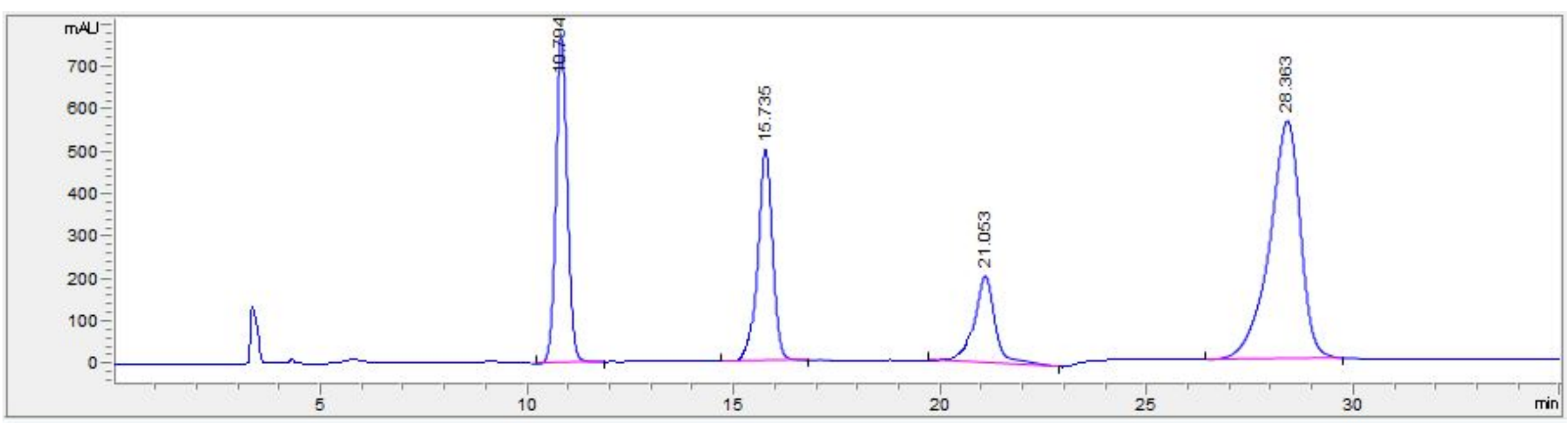


| # | Time | Area | Height | Width | Area% | Symmetry |
|---|---|---|---|---|---|---|
| 1 | 10.794 | 14739.8 | 772.4 | 0.2906 | 23.254 | 0.842 |
| 2 | 15.735 | 12193.8 | 496.6 | 0.3705 | 19.237 | 1.041 |
| 3 | 21.053 | 7369.3 | 204.8 | 0.5197 | 11.626 | 1.05 |
| 4 | 28.363 | 29083 | 559.5 | 0.7844 | 45.882 | 1.389 |

(*R*,*S*)-**29:** (*S*a,*S,S*)-**[Ir]** and (*S*)-Me-BTM

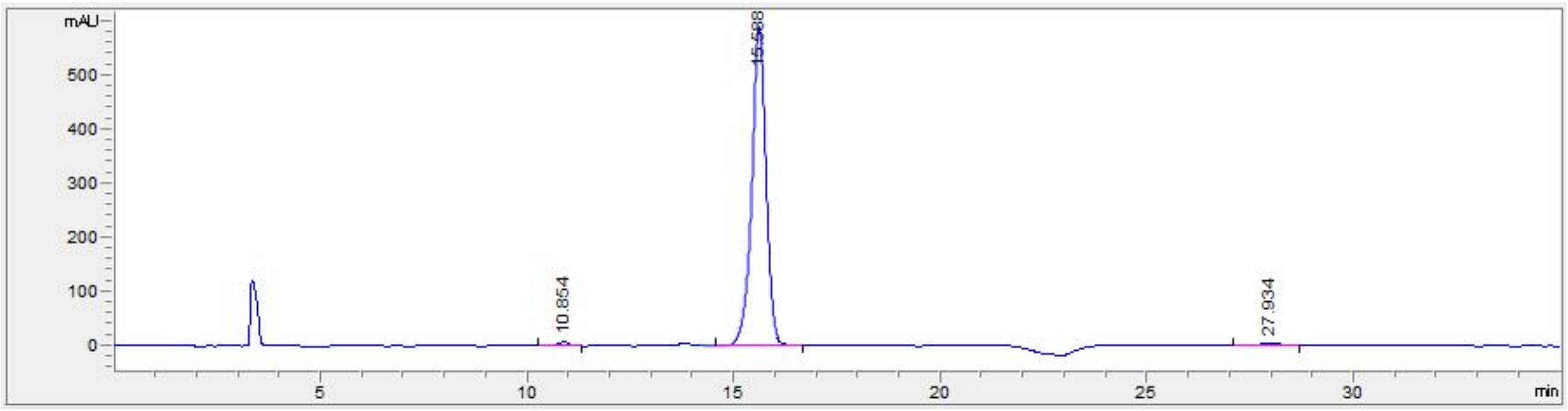


| # | Time | Area | Height | Width | Area% | Symmetry |
|---|---|---|---|---|---|---|
| 1 | 10.854 | 148.2 | 7.1 | 0.3187 | 1.027 | 0.987 |
| 2 | 15.588 | 14094.7 | 586.8 | 0.3602 | 97.653 | 1.036 |
| 3 | 27.934 | 190.5 | 4.3 | 0.5662 | 1.320 | 1.074 |

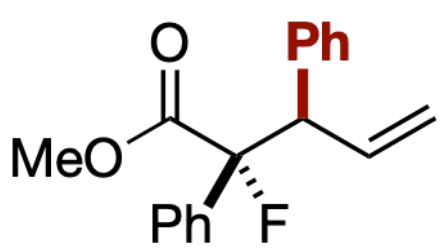


**Methyl (2*R*,3*R*)-2-fluoro-2,3-diphenylpent-4-enoate ((*R*,*R*)-28):** Pentafluorophenyl ester (*R*,*R*)-**3** (55.2 mg, 0.13 mmol, 1 equiv.) was dissolved in THF (1.3 ml, 0.1 M) in a 1-dram vial equipped with stirrer bar. MeOH (25.5 µl, 0.63 mmol, 5 equiv.), DMAP (3.1 mg, 0.025 mmol, 20 mol%) and diisopropylethylamine (0.11 ml, 0.63 mmol, 5 equiv.) were added sequentially and the reaction mixture was stirred overnight. Upon solvent removal under reduced pressure, the crude reaction mixture was redissolved with EtOAc and transferred to a separatory funnel. The organic layer was washed sequentially with $Na_2CO_3$ (2M aq.) (3 x 10mL) and brine (10 ml). The organic layer was dried with anhydrous $Na_2SO_4$, filtered and the solvent was removed under reduced pressure. The title compound was obtained (37 mg, 0.13 mmol, 100%) as white solid following purification by column chromatography [$SiO_2$, 2% Ethyl Acetate in Petroleum Ether]. The enantiomeric ratio (>99:1 *er*) was determined by chiral HPLC in comparison with the racemate.

**IR (film):** 3063 (w), 3030 (w), 2953 (w), 2922 (w), 1761 (m), 1739 (s), 1637 (w), 1602 (w) 1450 (m), 1269 (s) cm$^{-1}$.

**$^1$H NMR (500 MHz, $CDCl_3$):** δ 7.42 – 7.36 (m, 2H), 7.24 – 7.16 (m, 3H), 7.14 – 7.04 (m, 5H), 6.30 (ddd, *J* = 17.1, 10.3, 8.8 Hz, 1H), 5.32 – 5.20 (m, 2H), 4.31 (dd, *J* = 33.5, 9.0 Hz, 1H), 3.80 (s, 3H), ****3.49 (s)***.

**$^{13}$C NMR (126 MHz, $CDCl_3$):** δ 170.5 (d, *J* = 25.7 Hz), 137.3, 136.5 (d, *J* = 22.6 Hz), 135.6 (d, *J* = 5.1 Hz), 129.6 (d, *J* = 2.4 Hz), 128.3, 128.1, 127.0, 125.0 (d, *J* = 10.1 Hz), 118.8, 99.6 (d, *J* = 198.9 Hz), 57.8 (d, *J* = 19.3 Hz), 53.0.

**$^{19}$F NMR (376 MHz, $CDCl_3$):** δ -175.81 (d, *J* = 33.1 Hz, 1F).

**HRMS (ESI):** m/z calcd for $[M+Na]^+$ $C_{18}H_{17}O_2FNa$: 307.1105. Found: 307.1106.

HPLC analysis using a chiral column (Chiralpak IA 3μ column 22 ˚C, 0.5 ml/min, 400:1 hexane:isopropanol, 210 nm, $t_{major}$ = 20.740 min, $t_{minor}$ = 23.230 min, t = 25.710 min, t = 29.462 min).

(±)-**28:** (*rac*)-**[Ir]** and (*rac*)-Bn-BTM

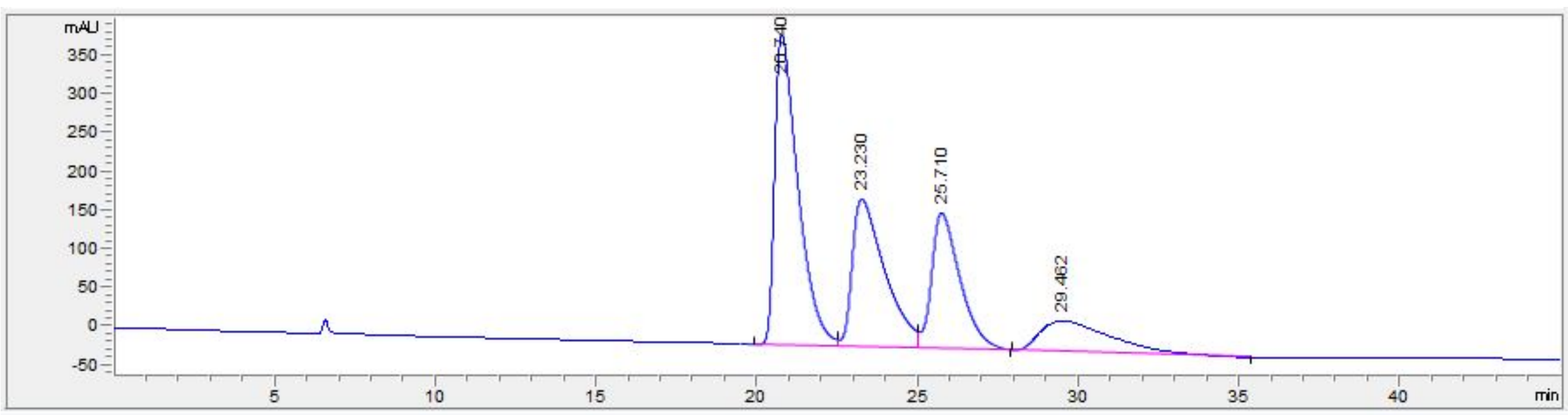


| # | Time | Area | Height | Width | Area% | Symmetry |
|---|---|---|---|---|---|---|
| 1 | 20.74 | 20214 | 400.9 | 0.7443 | 39.923 | 0.401 |
| 2 | 23.23 | 13492.4 | 190.8 | 1.0211 | 26.648 | 0.389 |
| 3 | 25.71 | 10796.3 | 175.4 | 0.9121 | 21.323 | 0.515 |
| 4 | 29.462 | 6129.3 | 39.5 | 1.8293 | 12.106 | 0.417 |

(*R*,*R*)-**28:** (*R<sub>a</sub>*,*R*,*R*)-**[Ir]** and (*S*)-Me-BTM

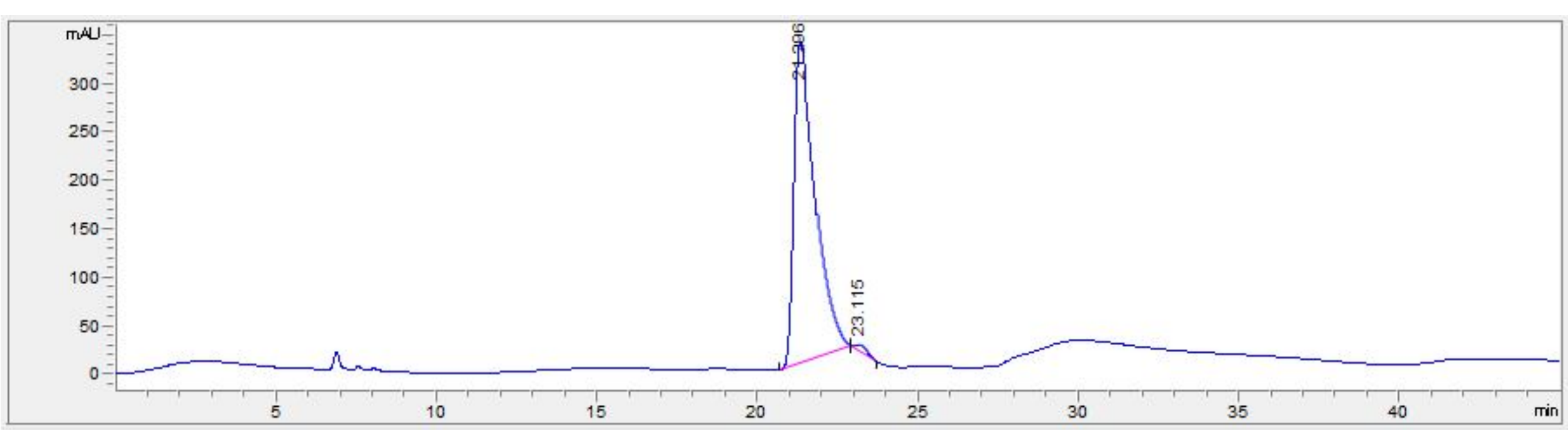


| # | Time | Area | Height | Width | Area% | Symmetry |
|---|---|---|---|---|---|---|
| 1 | 21.296 | 15658.2 | 332.6 | 0.6893 | 99.065 | 0.385 |
| 2 | 23.115 | 147.7 | 4.9 | 0.464 | 0.935 | 0.221 |

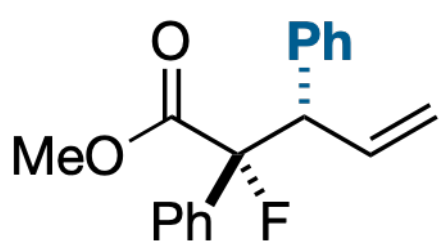

**Methyl (2*R*,3*S*)-2-fluoro-2,3-diphenylpent-4-enoate ((*R*,*S*)-28):** Pentafluorophenyl ester (*R*,*S*)-**3** (54 mg, 0.12 mmol, 1 equiv.) was dissolved in THF (1.2 ml, 0.1 M) in a 1-dram vial equipped with stirrer bar. MeOH (25 µl, 0.62 mmol, 5 equiv.), DMAP (3 mg, 0.025 mmol, 20 mol%) and diisopropylethylamine (0.11 ml, 0.62 mmol, 5 equiv.) were added sequentially and the reaction mixture was stirred overnight. Upon solvent removal under reduced pressure, the crude reaction mixture was redissolved with EtOAc and transferred to a separatory funnel. The organic layer was washed sequentially with $Na_2CO_3$ (2M aq.) (3 x 10mL) and brine (10 ml). The organic layer was dried with anhydrous $Na_2SO_4$, filtered and the solvent was removed under reduced pressure. The title compound was obtained (32.7 mg, 0.11 mmol, 93%) as white solid following purification by column chromatography [$SiO_2$, 2% Ethyl Acetate in Petroleum Ether]. The enantiomeric ratio (>99:1 *er*) was determined by chiral HPLC in comparison with the racemate.

**IR (film):** 3062 (w), 3029 (w), 2959 (w), 1759 (m), 1739 (s), 1639 (w), 1601 (w) 1448 (m), 1265 (s) $cm^{-1}$.

**$^1$H NMR (500 MHz, $CDCl_3$):** δ 7.69 – 7.62 (m, 2H), 7.44 – 7.39 (m, 4H), 7.38 – 7.31 (m, 3H), 7.30 – 7.25 (m, 1H), 5.95 (ddd, *J* = 17.1, 10.4, 8.1 Hz, 1H), 5.00 (dt, *J* = 10.3, 1.2 Hz, 1H), 4.85 (dt, *J* = 17.2, 1.4 Hz, 1H), 4.30 (dd, *J* = 34.0, 8.5 Hz, 1H), ****3.80 (s)***, 3.49 (s, 3H).

**$^{13}$C NMR (126 MHz, $CDCl_3$):** δ 169.8 (d, *J* = 26.4 Hz), 138.3, 136.9 (d, *J* = 22.9 Hz), 134.3 (d, *J* = 4.8 Hz), 129.5 (d, *J* = 2.8 Hz), 128.5, 128.4, 128.4 (d, *J* = 2.3 Hz), 127.4, 125.2 (d, *J* = 10.7 Hz), 119.2, 99.0 (d, *J* = 198.9 Hz), 57.2 (d, *J* = 19.2 Hz), 52.6.

**$^{19}$F NMR (376 MHz, $CDCl_3$):** δ -175.35 (d, *J* = 33.7 Hz, 1F).

**HRMS (ESI):** m/z calcd for $[M+Na]^+$ $C_{18}H_{17}O_2FNa$: 307.1105. Found: 307.1106.

HPLC analysis using a chiral column (Chiralpak IA 3µ column 22 ˚C, 0.5 ml/min, 400:1 hexane:isopropanol, 210 nm, $t_{major}$ = 21.503 min, $t_{minor}$ = 22.696 min, t = 18.856 min, t = 20.072 min).

(±)-**28:** (*rac*)-**[Ir]** and (*rac*)-Bn-BTM

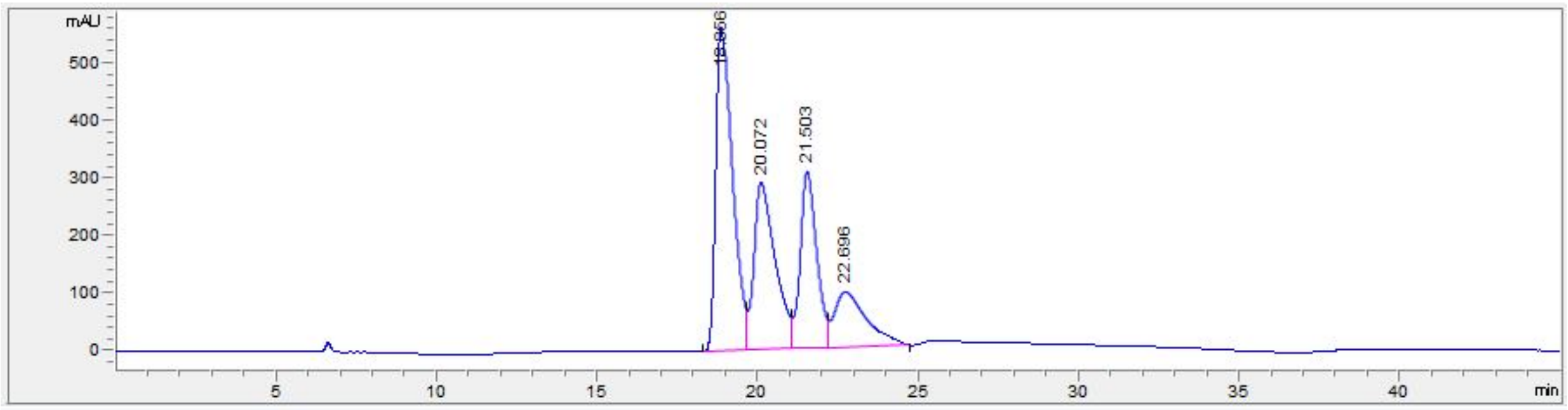


| # | Time | Area | Height | Width | Area% | Symmetry |
|---|---|---|---|---|---|---|
| 1 | 18.856 | 19590.7 | 561.3 | 0.5232 | 38.569 | 0.454 |
| 2 | 20.072 | 13416.1 | 290.8 | 0.6682 | 26.413 | 0.468 |
| 3 | 21.503 | 11099 | 307.1 | 0.5376 | 21.851 | 0.676 |
| 4 | 22.696 | 6687.5 | 96.4 | 0.9994 | 13.166 | 0.455 |

(*R*,*S*)-**28:** ($S_a$,*S*,*S*)-**[Ir]** and (*S*)-Me-BTM

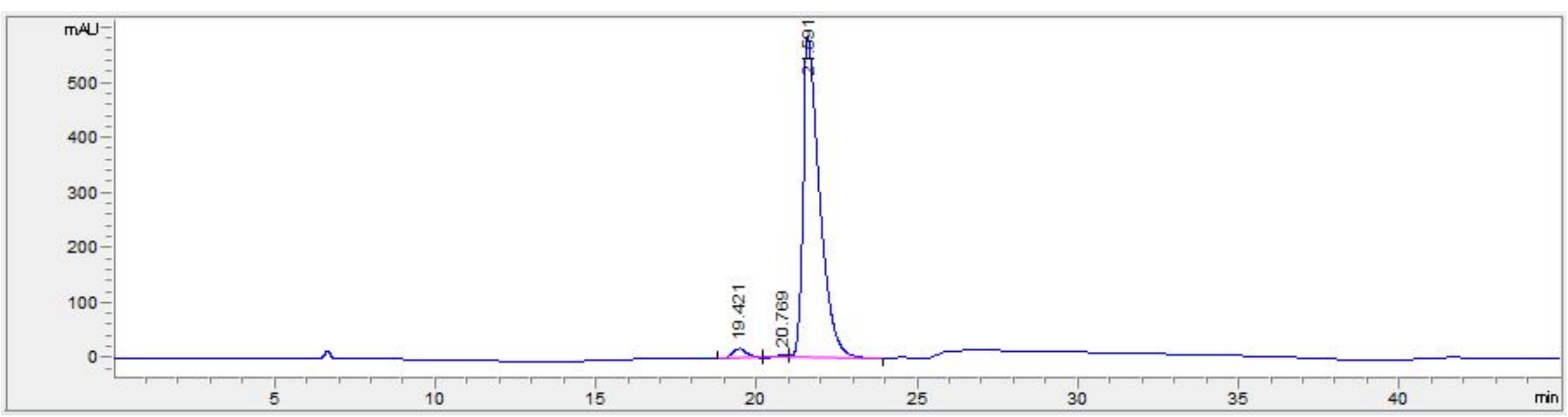


| # | Time | Area | Height | Width | Area% | Symmetry |
|---|---|---|---|---|---|---|
| 1 | 19.421 | 591.6 | 18 | 0.5071 | 2.588 | 0.788 |
| 2 | 20.769 | 168.7 | 5.7 | 0.4508 | 0.738 | 1.305 |
| 3 | 21.591 | 22100.9 | 584.8 | 0.5569 | 96.674 | 0.46 |

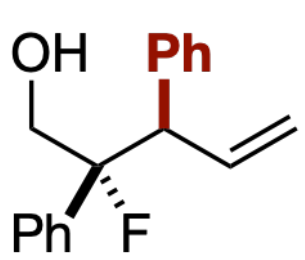


**(2*R*,3*R*)-2-fluoro-2,3-diphenylpent-4-en-1-ol ((*R*,*R*)-27):** An oven-dried 1-dram vial equipped with a stirrer bar was charged with pentafluorophenyl ester (*R*,*R*)-**3aa** (52 mg, 0.12 mmol, 1 equiv.). The vial was evacuated and backfilled with $N_2$ (3 times) before adding THF (2.4 ml, 0.05 M). The resulting solution was cooled to 0 °C using an ice bath and a solution of $LiAlH_4$ (1 M in THF, 0.26 ml, 0.26 mmol, 2.2 equiv.) was added dropwise via syringe. After complete addition, the reaction mixture was allowed to warm to room temperature and stirred for 2 hours. Thereafter, aq. HCl (1 N) was added dropwise at 0 °C until gas evolution stopped. The reaction mixture was diluted with EtOAc and transferred to a separatory funnel. The aqueous phase was extracted with EtOAc (3 times). The combined organic layer was washed sequentially with $Na_2CO_3$ (2M aq.) (3 x 10mL) and brine (10 ml). The organic layer was dried with

anhydrous $Na_2SO_4$ filtered and the solvent was removed under reduced pressure. The title compound was obtained (29.7 mg, 0.12 mmol, 96.5%) as white solid following purification by column chromatography [$SiO_2$, 8% to 10% Ethyl Acetate in Petroleum Ether]. The enantiomeric ratio (>99:1 *er*) was determined by chiral HPLC in comparison with the racemate.

**IR (film):** 3318 (br), 3063 (w), 3030 (w), 2943 (w), 1637 (w), 1602 (w) 1450 (m) $cm^{-1}$.

**$^1$H NMR (500 MHz, $CDCl_3$):** δ 7.23 – 7.11 (m, 3H), 7.09 – 7.00 (m, 5H), 6.98 – 6.86 (m, 2H), 6.34 – 6.09 (m, 1H), 5.26 – 4.97 (m, 2H), 4.00 (ddd, *J* = 17.1, 12.3, 5.3 Hz, 1H), 3.95 – 3.87 (m, 1H), 3.83 (dd, *J* = 24.5, 9.5 Hz, 1H), 1.59 (t, *J* = 6.5 Hz, 1H).

**$^{13}$C NMR (126 MHz, $CDCl_3$):** δ 138.9 (d, *J* = 3.2 Hz), 138.4 (d, *J* = 21.8 Hz), 136.1 (d, *J* = 4.1 Hz), 129.5 (d, *J* = 1.9 Hz), 128.1, 128.0 (d, *J* = 1.8 Hz), 127.8, 126.9, 125.7 (d, *J* = 10.1 Hz), 118.2, 100.4 (d, *J* = 182.6 Hz), 67.0 (d, *J* = 24.7 Hz), 56.6 (d, *J* = 21.7 Hz).

**$^{19}$F NMR (376 MHz, $CDCl_3$):** δ -171.59 (td, *J* = 24.4, 16.7 Hz, 1F).
**HRMS (ESI):** m/z calcd for $[M+Na]^+$ $C_{17}H_{17}OFNa$: 279.1156. Found: 279.1154.

HPLC analysis using a chiral column (Cellulose 1 3μ column 22 ˚C, 1.0 ml/min, 97:3 hexane:isopropanol, 210 nm, $t_{major}$ = 18.672 min, $t_{minor}$ = 17.099 min, t = 24.336 min, t = 25.536 min).

(+)-**27:** (*rac*)-**[Ir]** and (*rac*)-Bn-BTM

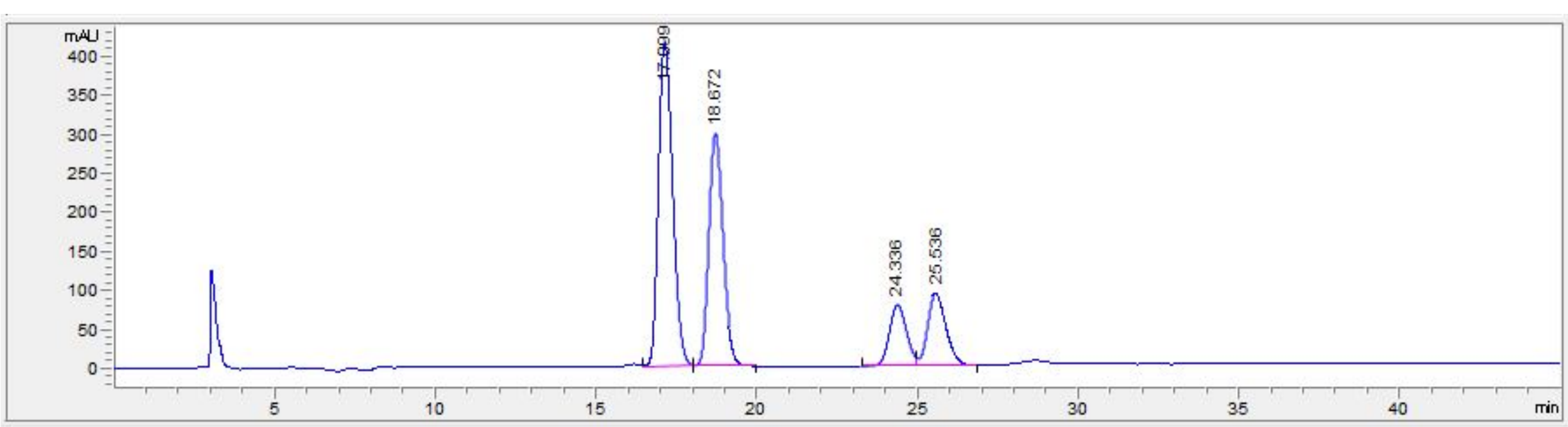


| # | Time | Area | Height | Width | Area% | Symmetry |
|---|---|---|---|---|---|---|
| 1 | 17.099 | 12673.3 | 414.5 | 0.4807 | 44.116 | 0.733 |
| 2 | 18.672 | 9339.7 | 298.6 | 0.489 | 32.512 | 0.791 |
| 3 | 24.336 | 2928 | 78.5 | 0.5734 | 10.193 | 0.863 |
| 4 | 25.536 | 3786.1 | 93.6 | 0.6339 | 13.180 | 0.751 |

(*R*,*R*)-**27:** (*R*$_a$,*R*,*R*)-**[Ir]** and (*S*)-Me-BTM

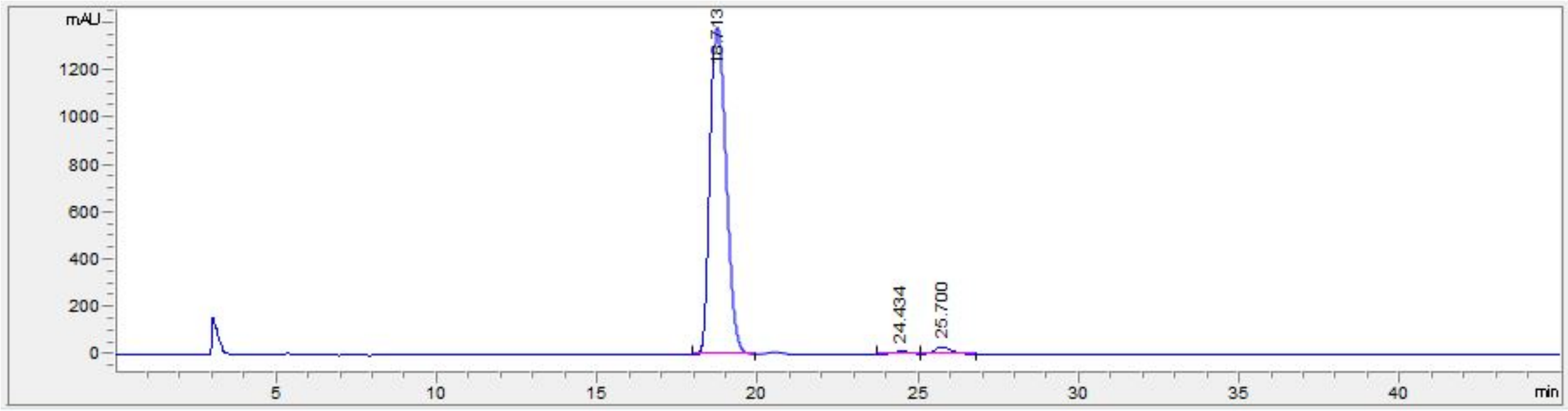


| # | Time | Area | Height | Width | Area% | Symmetry |
|---|---|---|---|---|---|---|
| 1 | 18.713 | 45853.3 | 1383.7 | 0.5266 | 96.553 | 0.721 |
| 2 | 24.434 | 495.3 | 13.2 | 0.5458 | 1.043 | 0.899 |
| 3 | 25.7 | 1141.5 | 28.7 | 0.6107 | 2.404 | 0.748 |

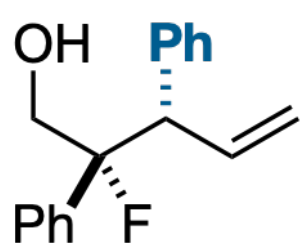


**(2*R*,3*S*)-2-fluoro-2,3-diphenylpent-4-en-1-ol ((*R*,*S*)-27):** An oven-dried 1-dram vial equipped with a stirrer bar was charged with pentafluorophenyl ester (*R*,*S*)-**3** (62.2 mg, 0.14 mmol, 1 equiv.). The vial was evacuated and backfilled with $N_2$ (3 times) before adding THF (2.8 ml, 0.05 M). The resulting solution was cooled to 0 °C using an ice bath and a solution of $LiAlH_4$ (1 M in THF, 0.31 ml, 0.31 mmol, 2.2 equiv.) was added dropwise via syringe. After complete addition, the reaction mixture was allowed to warm to room temperature and stirred for 2 hours. Thereafter, aq. HCl (1 N) was added dropwise at 0 °C until gas evolution stopped. The reaction mixture was diluted with EtOAc and transferred to a separatory funnel. The aqueous phase was extracted with EtOAc (3 times). The combined organic layer was washed sequentially with $Na_2CO_3$ (2M aq.) (3 x 10mL) and brine (10 ml). The organic layer was dried with anhydrous $Na_2SO_4$, filtered and the solvent was removed under reduced pressure. The title compound was obtained (30.6 mg, 0.12 mmol, 85%) as white solid following purification by column chromatography [$SiO_2$, 5% to 8% Ethyl Acetate in Petroleum Ether]. The enantiomeric ratio (>99:1 *er*) was determined by chiral HPLC in comparison with the racemate.

**IR (film):** 3396 (br), 3062 (w), 3029 (w), 2923 (w), 1637 (w), 1601(w) 1448 (w) cm$^{-1}$.

**$^1$H NMR (500 MHz, $CDCl_3$):** δ 7.41 – 7.35 (m, 2H), 7.35 – 7.31 (m, 1H), 7.31 – 7.24 (m, 5H), 7.20 – 7.13 (m, 2H), 6.05 (ddd, *J* = 17.0, 10.3, 8.6 Hz, 1H), 5.05 (dd, *J* = 10.1, 1.6

Hz, 1H), 4.97 (dt, *J* = 17.1, 1.3 Hz, 1H), 3.92 (dd, *J* = 24.5, 8.5 Hz, 1H), 3.88 – 3.83 (m, 1H), 3.83 – 3.78 (m, 1H), 1.49 (td, *J* = 6.9, 1.1 Hz, 1H).

**$^{13}$C NMR (126 MHz, $CDCl_3$):** δ 139.0 (d, *J* = 22.0 Hz), 138.8, 135.9 (d, *J* = 6.8 Hz), 129.7 (d, *J* = 2.0 Hz), 128.4, 128.4 (d, *J* = 2.1 Hz), 128.0, 127.3, 125.6 (d, *J* = 10.6 Hz), 118.5, 100.6 (d, *J* = 183.2 Hz), 67.2 (d, *J* = 25.0 Hz), 56.5 (d, *J* = 21.5 Hz).

**$^{19}$F NMR (376 MHz, $CDCl_3$):** δ -172.62 (td, *J* = 23.8, 17.2 Hz, 1F).

**HRMS (EI):** m/z calcd for $[M]^+$ $C_{17}H_{17}FO$: 256.1263. Found: 256.1263.

HPLC analysis using a chiral column (Cellulose 1 3μ column 22 ˚C, 1.0 ml/min, 97:3 hexane:isopropanol, 210 nm, $t_{major}$ = 25.536 min, $t_{minor}$ = 24.336 min, t = 17.099 min, t = 18.672 min).

(±)-**27:** (*rac*)-**[Ir]** and (*rac*)-Bn-BTM

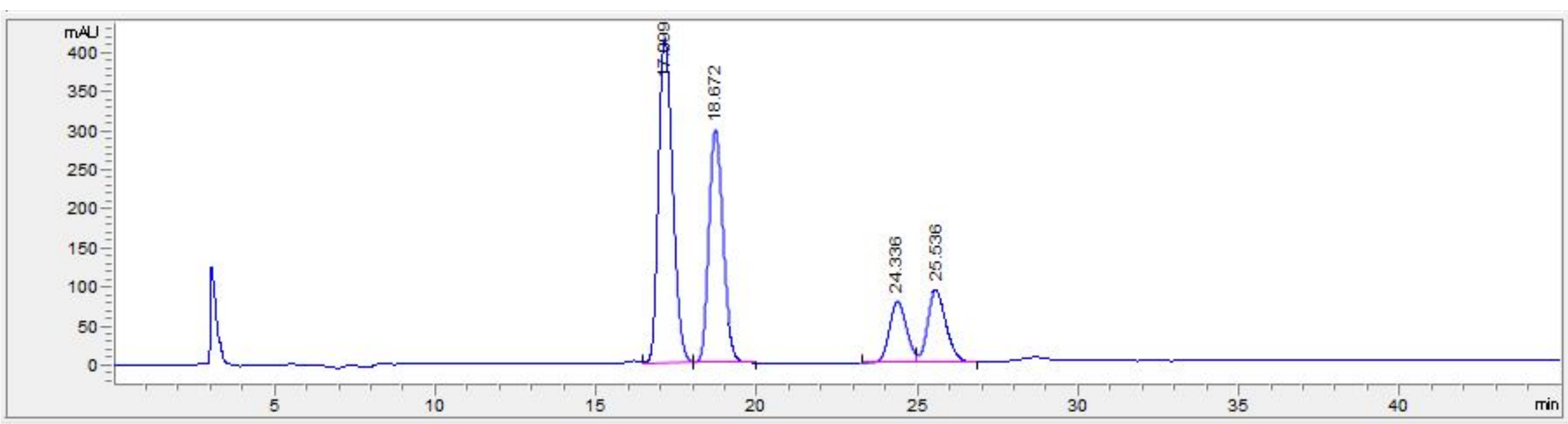


| # | Time | Area | Height | Width | Area% | Symmetry |
|---|---|---|---|---|---|---|
| 1 | 17.099 | 12673.3 | 414.5 | 0.4807 | 44.116 | 0.733 |
| 2 | 18.672 | 9339.7 | 298.6 | 0.489 | 32.512 | 0.791 |
| 3 | 24.336 | 2928 | 78.5 | 0.5734 | 10.193 | 0.863 |
| 4 | 25.536 | 3786.1 | 93.6 | 0.6339 | 13.180 | 0.751 |

(*R*,*S*)-**27:** (*S*$_a$*,S,S*)-**[Ir]** and (*S*)-Me-BTM

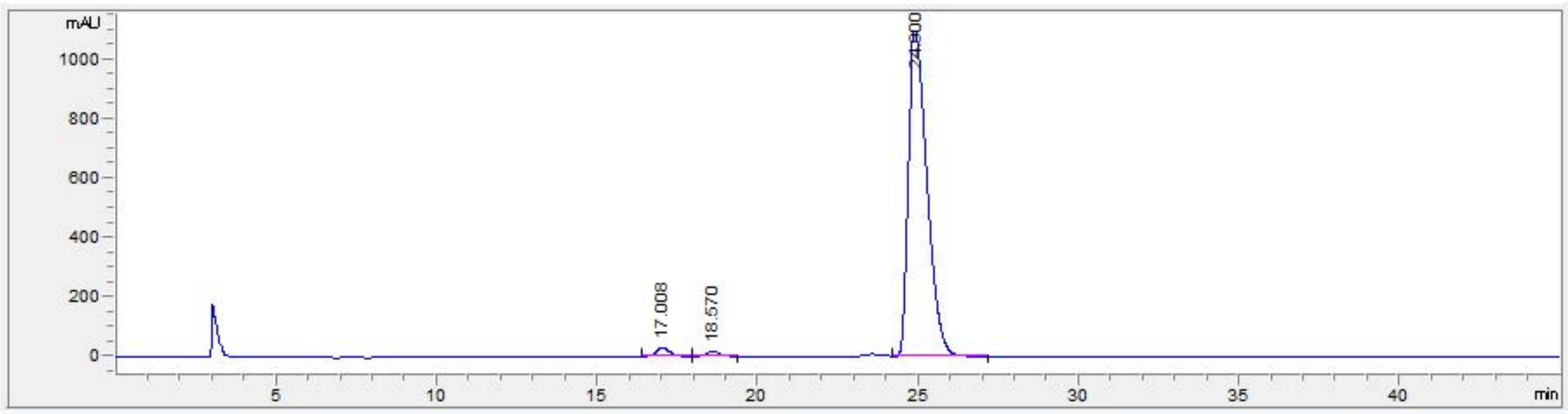


| # | Time | Area | Height | Width | Area% | Symmetry |
|---|---|---|---|---|---|---|
| 1 | 17.008 | 841.7 | 29 | 0.4451 | 1.761 | 0.867 |
| 2 | 18.57 | 508 | 16.5 | 0.4765 | 1.063 | 0.858 |
| 3 | 24.9 | 46446.4 | 1097.4 | 0.6718 | 97.176 | 0.589 |

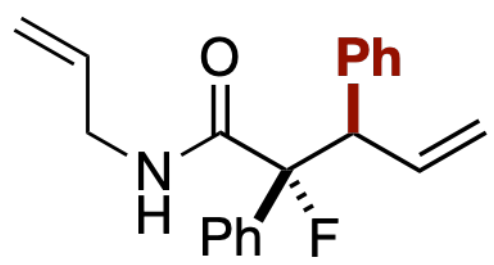


**(2*R*,3*R*)-N-allyl-2-fluoro-2,3-diphenylpent-4-enamide ((*R*,*R*)-30):** Pentafluorophenyl ester **(*R*,*R*)-3** (93.7 mg, 0.21 mmol, 1 equiv.) was dissolved in THF (2.1 ml, 0.1 M) in a 1-dram vial equipped with stirrer bar. Allyl amine (0.16 ml, 2.14 mmol, 10 equiv.) was added and the reaction mixture was stirred at room temperature for 15 minutes. Upon solvent removal under reduced pressure, the crude reaction mixture was redissolved with EtOAc and transferred to a separatory funnel. The organic layer was washed sequentially with $Na_2CO_3$ (2M aq.) (3 x 10mL) and brine (10 ml). The organic layer was dried with anhydrous $Na_2SO_4$, filtered and the solvent was removed under reduced pressure. The title compound was obtained (64.4 mg, 0.21 mmol, 99%) as white solid following purification by column chromatography [$SiO_2$, 3% to 5% Ethyl Acetate in Petroleum Ether]. The enantiomeric ratio (>99:1 *er*) was determined by chiral HPLC in comparison with the racemate.

**IR (film):** 3383 (br), 3080 (w), 1671 (s), 1645 (w), 1525 (s), 1493 (w), 1450 (w) cm$^{-1}$.

**$^1$H NMR (500 MHz, $CDCl_3$):** δ 7.47 – 7.38 (m, 2H), 7.21 – 7.14 (m, 3H), 7.13 – 7.05 (m, 5H), 6.57 (q, *J* = 6.0 Hz, 1H), 6.27 (ddd, *J* = 17.0, 10.3, 8.4 Hz, 1H), 5.79 (ddt, *J* = 17.1, 10.3, 5.6 Hz, 1H), 5.29 – 5.19 (m, 2H), 5.19 – 5.06 (m, 2H), 4.40 (dd, *J* = 34.5, 8.0 Hz, 1H), 3.99 (dddd, *J* = 15.9, 6.6, 5.2, 1.4 Hz, 1H), 3.88 – 3.75 (m, 1H).

**$^{13}$C NMR (126 MHz, $CDCl_3$):** δ 169.8 (d, *J* = 21.1 Hz), 137.9, 137.2 (d, *J* = 22.1 Hz), 135.4 (d, *J* = 5.6 Hz), 133.8, 129.7 (d, *J* = 2.2 Hz), 128.1, 128.0, 127.9 (d, *J* = 2.1 Hz), 126.9, 124.9 (d, *J* = 10.5 Hz), 119.1, 116.8, 101.7 (d, *J* = 197.0 Hz), 57.3 (d, *J* = 19.4 Hz), 41.8.

**$^{19}$F NMR (376 MHz, $CDCl_3$):** δ -173.61 (d, $J$ = 34.9 Hz, 1F).

**HRMS (ESI):** m/z calcd for $[M+Na]^+$ $C_{20}H_{20}ONFNa$: 332.1421. Found: 332.1423.

HPLC analysis using a chiral column (Cellulose 1 3μ column 22 ˚C, 0.5 ml/min, 97:3 hexane:isopropanol, 210 nm, $t_{major}$ = 17.830 min, $t_{minor}$ = 14.897 min, t = 13.440 min, t = 15.915 min).

(±)-**30:** (*rac*)-**[Ir]** and (*rac*)-Bn-BTM

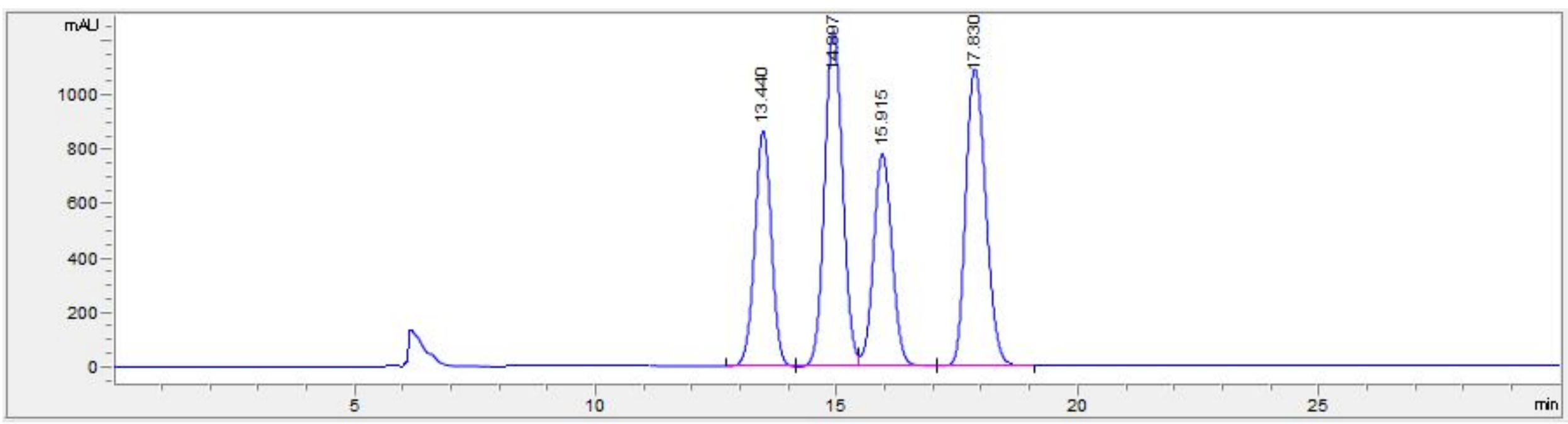


| # | Time | Area | Height | Width | Area% | Symmetry |
|---|---|---|---|---|---|---|
| 1 | 13.44 | 20734.7 | 864.6 | 0.3718 | 19.774 | 0.993 |
| 2 | 14.897 | 32141.1 | 1228.3 | 0.4093 | 30.651 | 0.909 |
| 3 | 15.915 | 20704 | 778.8 | 0.4181 | 19.744 | 0.908 |
| 4 | 17.83 | 31280.2 | 1089.8 | 0.4529 | 29.830 | 0.782 |

(*R*,*R*)-**30:** (*R*$_a$*,R,R*)-**[Ir]** and (*S*)-Me-BTM

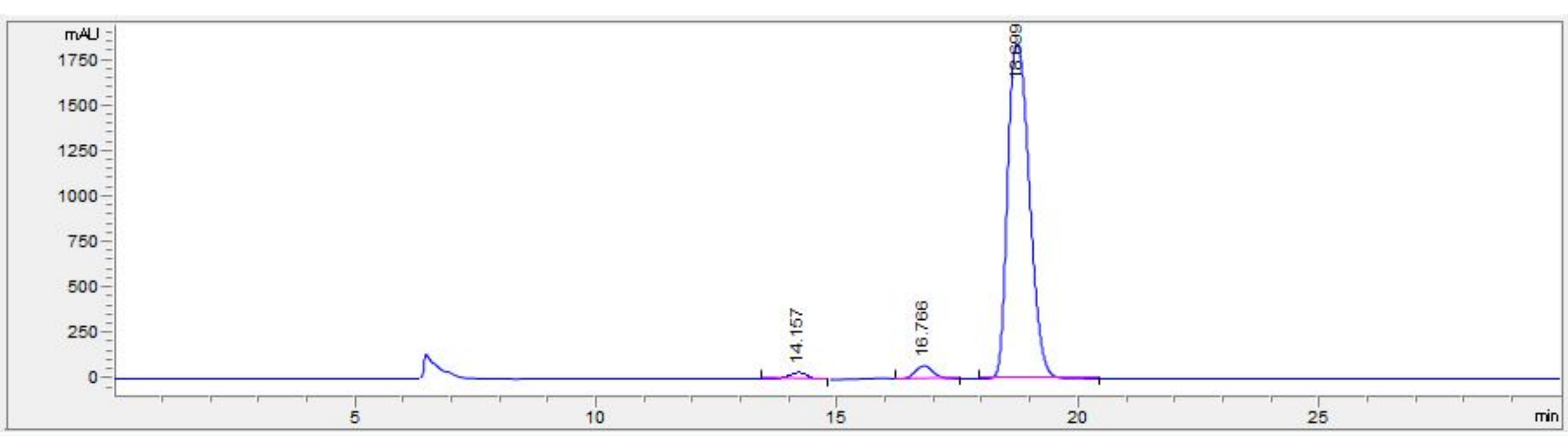


| # | Time | Area | Height | Width | Area% | Symmetry |
|---|---|---|---|---|---|---|
| 1 | 14.157 | 882.5 | 35.1 | 0.3857 | 1.454 | 1.045 |
| 2 | 16.766 | 1905.4 | 72.5 | 0.4084 | 3.140 | 0.981 |
| 3 | 18.699 | 57886.3 | 1849.8 | 0.5011 | 95.405 | 0.716 |

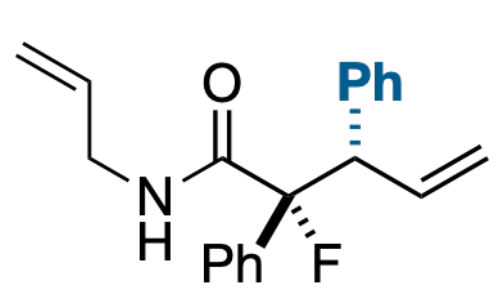


**(2*R*,3*S*)-N-allyl-2-fluoro-2,3-diphenylpent-4-enamide ((*R*,*S*)-30):** Pentafluorophenyl ester (*R*,*S*)-**3** (56 mg, 0.13 mmol, 1 equiv.) was dissolved in THF (1.3 ml, 0.1 M) in a 1-dram vial equipped with stirrer bar. Allyl amine (96 μl, 1.28 mmol, 10 equiv.) was added and the reaction mixture was

stirred at room temperature for 15 minutes. Upon solvent removal under reduced pressure, the crude reaction mixture was redissolved with EtOAc and transferred to a separatory funnel. The organic layer was washed sequentially with $Na_2CO_3$ (2M aq.) (3 x 10mL) and brine (10 ml). The organic layer was dried with anhydrous $Na_2SO_4$, filtered and the solvent was removed under reduced pressure. The title compound was obtained (39.9 mg, 0.13 mmol, 100%) as white solid following purification by column chromatography [$SiO_2$, 3% to 5% Ethyl Acetate in Petroleum Ether]. The enantiomeric ratio (>99:1 *er*) was determined by chiral HPLC in comparison with the racemate.

**IR (film):** 3388 (br), 3063 (w), 3031 (w), 2981 (w), 1663 (s), 1645 (m), 1601 (w), 1521 (s), 1495 (m), 1449 (w) $cm^{-1}$.

**$^1$H NMR (500 MHz, $CDCl_3$):** δ 7.67 – 7.60 (m, 2H), 7.34 (ddt, *J* = 10.2, 7.9, 3.9 Hz, 4H), 7.26 (td, *J* = 8.0, 6.6 Hz, 3H), 7.22 – 7.16 (m, 1H), 6.11 (q, *J* = 5.4 Hz, 1H), 5.91 (ddd, *J* = 17.1, 10.3, 8.3 Hz, 1H), 5.36 – 5.22 (m, 1H), 4.93 (dt, *J* = 10.4, 1.3 Hz, 1H), 4.81 (dt, *J* = 17.2, 1.5 Hz, 1H), 4.77 (dq, *J* = 10.3, 1.6 Hz, 1H), 4.54 (dq, *J* = 17.2, 1.6 Hz, 1H), 4.34 (dd, *J* = 36.5, 8.5 Hz, 1H), 3.73 – 3.58 (m, 1H), 3.36 (dt, *J* = 16.0, 5.4 Hz, 1H).

**$^{13}$C NMR (126 MHz, $CDCl_3$):** δ 169.4 (d, *J* = 21.5 Hz), 138.5, 137.5 (d, *J* = 22.0 Hz), 134.6 (d, *J* = 4.6 Hz), 133.4, 130.1 (d, *J* = 2.2 Hz), 128.4, 128.3 (d, *J* = 2.3 Hz), 128.3, 127.3, 125.2 (d, *J* = 11.1 Hz), 119.3, 116.1, 101.2 (d, *J* = 198.2 Hz), 56.8 (d, *J* = 18.8 Hz), 41.3.

**$^{19}$F NMR (376 MHz,$CDCl_3$):** δ -175.78 (dd, *J* = 36.4, 5.8 Hz, 1H).

**HRMS (ESI):** m/z calcd for $[M+Na]^+$ $C_{20}H_{20}ONFNa$: 332.1421. Found: 332.1423.

HPLC analysis using a chiral column (Cellulose 1 3μ column 22 ˚C, 0.5 ml/min, 97:3 hexane:isopropanol, 210 nm, $t_{major}$ = 15.915 min, $t_{minor}$ = 13.440 min, t = 14.897 min, t = 17.830 min).

(±)-**30:** (*rac*)-**[Ir]** and (*rac*)-Bn-BTM

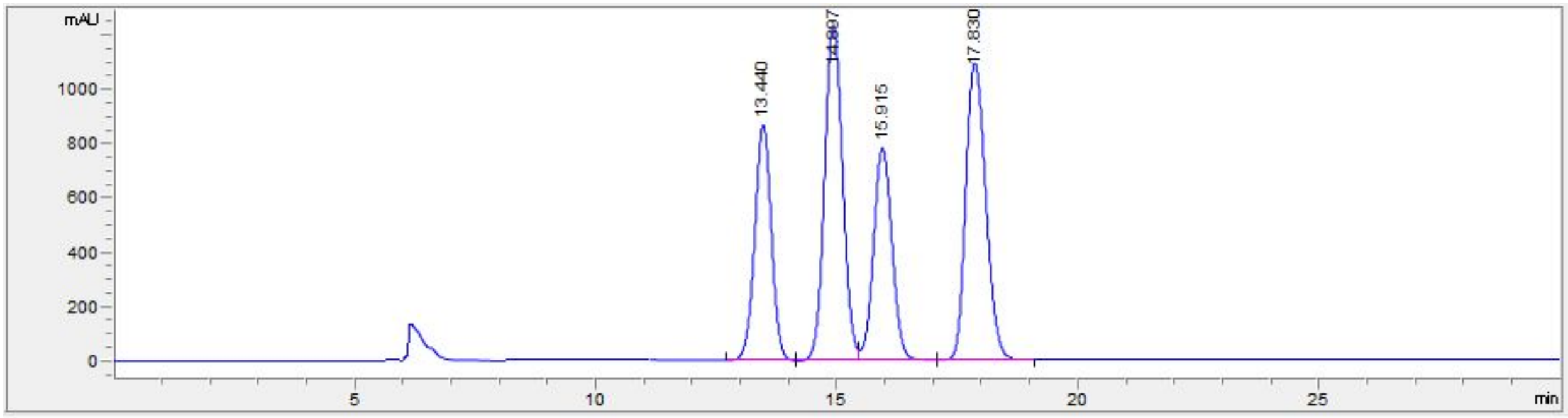

| # | Time | Area | Height | Width | Area% | Symmetry |
|---|---|---|---|---|---|---|
| 1 | 13.44 | 20734.7 | 864.6 | 0.3718 | 19.774 | 0.993 |
| 2 | 14.897 | 32141.1 | 1228.3 | 0.4093 | 30.651 | 0.909 |
| 3 | 15.915 | 20704 | 778.8 | 0.4181 | 19.744 | 0.908 |
| 4 | 17.83 | 31280.2 | 1089.8 | 0.4529 | 29.830 | 0.782 |

(*R*,*S*)-**30:** ($S_a$,*S*,*S*)-**[Ir]** and (*S*)-Me-BTM

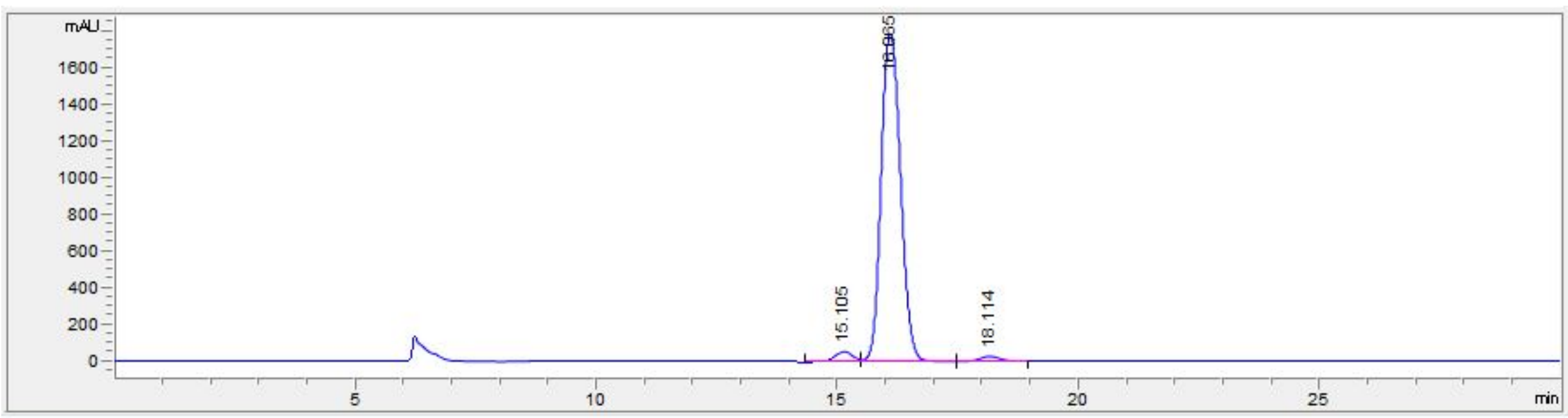

| # | Time | Area | Height | Width | Area% | Symmetry |
|---|---|---|---|---|---|---|
| 1 | 15.105 | 1477.1 | 56.5 | 0.4031 | 2.830 | 1.039 |
| 2 | 16.065 | 49935.2 | 1789.1 | 0.4456 | 95.686 | 0.829 |
| 3 | 18.114 | 774 | 27.1 | 0.4488 | 1.483 | 0.873 |

**Confirmation of absolute stereochemistry via X-ray analysis (Scheme 2, manuscript)**

**(2*R*,3*R*)-N-(4-bromobenzyl)-2-fluoro-2,3-diphenylpent-4-enamide: (*R*,*R*)-3-amide:**

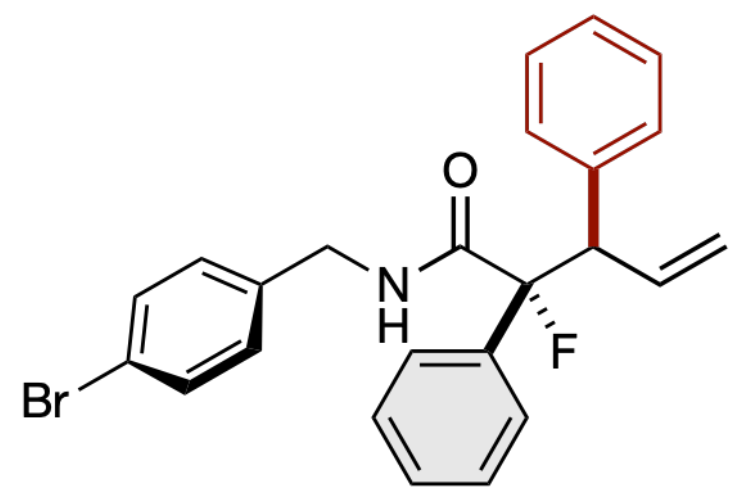


X-ray Single crystals of sufficient quality were obtained by slow evaporation of a saturated solution of **(*R*,*R*)-3-amide** in methanol. Single crystal analysis confirmed the absolute stereochemistry as (*R,R*) and is derived from ($R_a$,*R*,*R*)-[Ir] catalyst and (*S*)-Me-BTM. Thus, all stereochemistry was assigned in analogy to this.

This structure has been deposited at the Cambridge Crystallographic Data Center and can be found under the deposition number: CCDC 2349145.

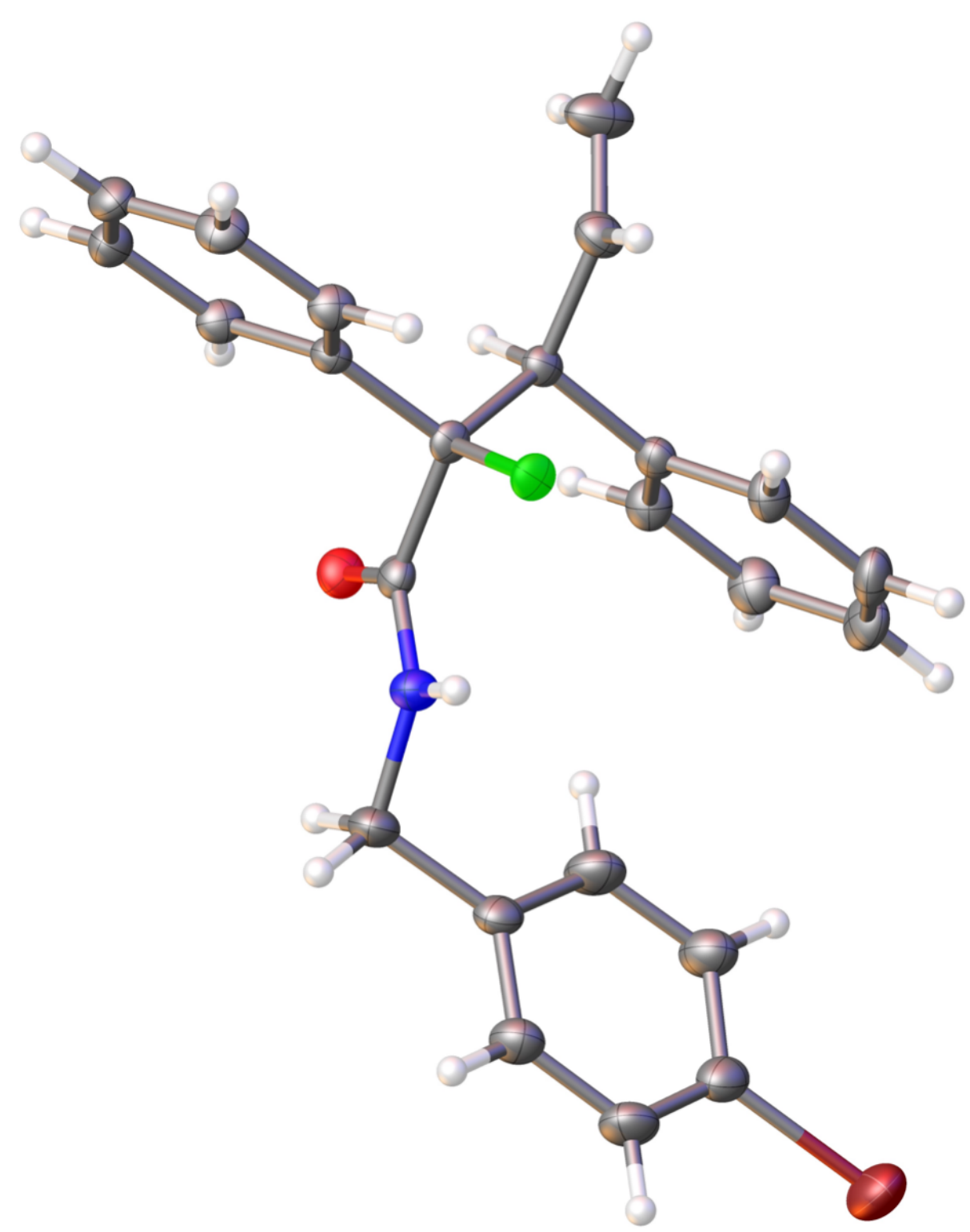

**(2*R*,3*S*)-N-(4-bromobenzyl)-2-fluoro-2,3-diphenylpent-4-enamide: (R,S)-3-amide**

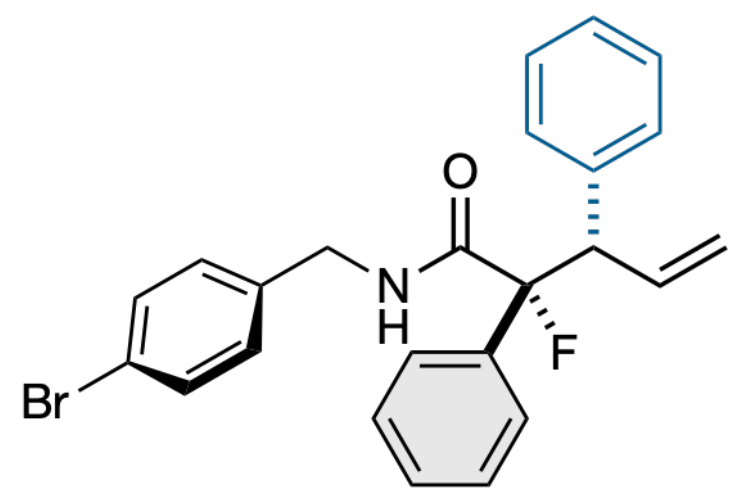


X-ray Single crystals of sufficient quality were obtained by slow evaporation of a solution of **(*R*,*S*)-3-amide** in chloroform-d. Single crystal analysis confirmed the absolute stereochemistry as (*R,S*) and is derived from ($S_a$*,S,S*)-[Ir] catalyst and (*S*)-Me-BTM. Thus, all stereochemistry was assigned in analogy to this.

This structure has been deposited at the Cambridge Crystallographic Data Center and can be found under the deposition number: CCDC 2349144.

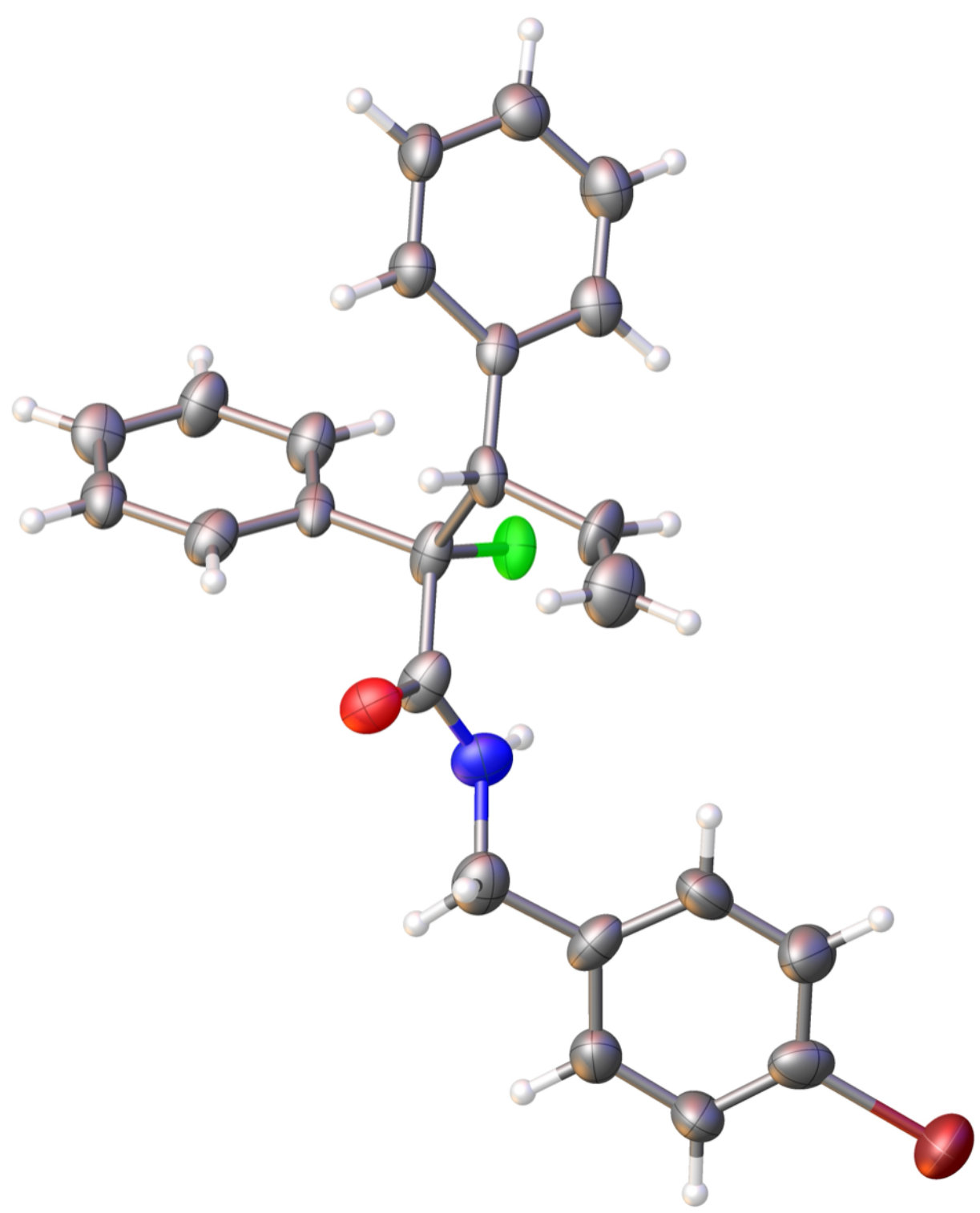

**(2*S*,3*R*)-N-(4-bromobenzyl)-2-fluoro-2,3-diphenylpent-4-enamide: (*S*,*R*)-3-amide**

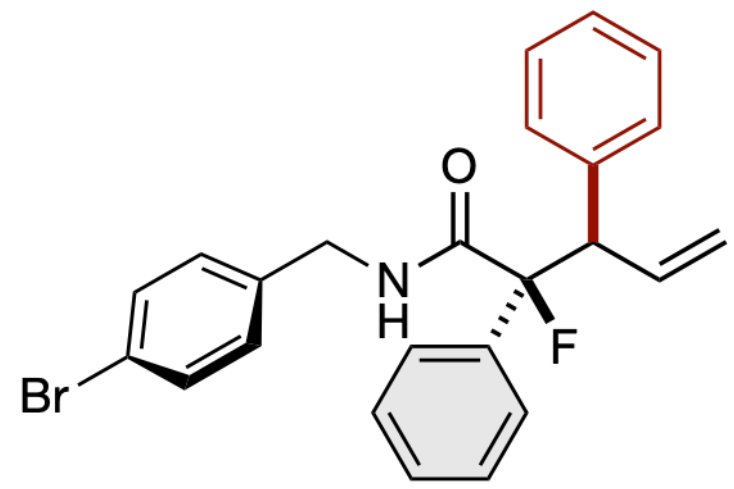


X-ray Single crystals of sufficient quality were obtained by slow evaporation of a saturated solution of **(*S*,*R*)-3-amide** in ethanol. Single crystal analysis confirmed the absolute stereochemistry as (*S,R*) and is derived from ($R_a$,*R,R*)-[Ir] catalyst and (*R*)-Me-BTM.

This structure has been deposited at the Cambridge Crystallographic Data Center and can be found under the deposition number: CCDC 2349147.

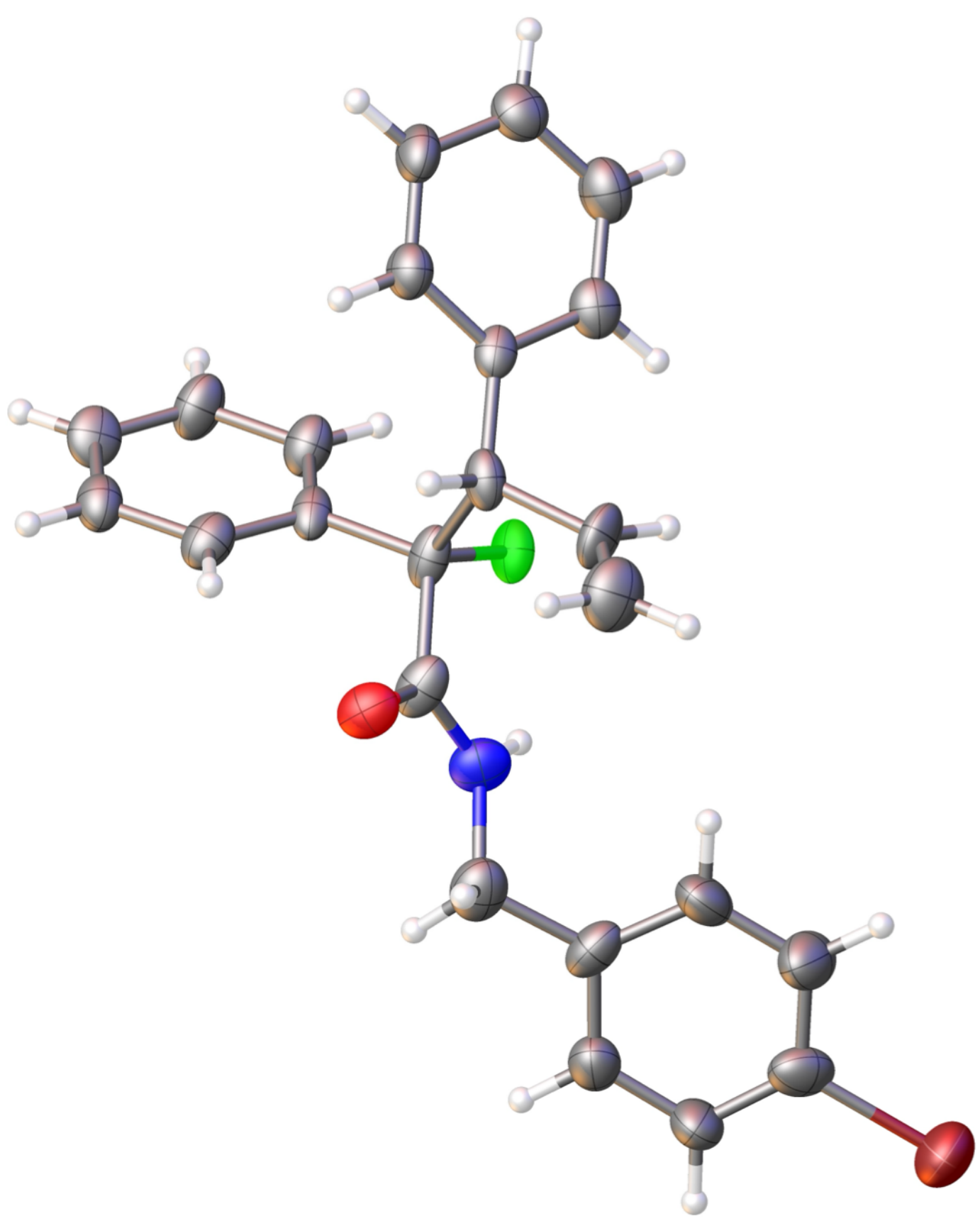

**(2*S*,3*S*)-N-(4-bromobenzyl)-2-fluoro-2,3-diphenylpent-4-enamide: (*S*,*S*)-3-amide**

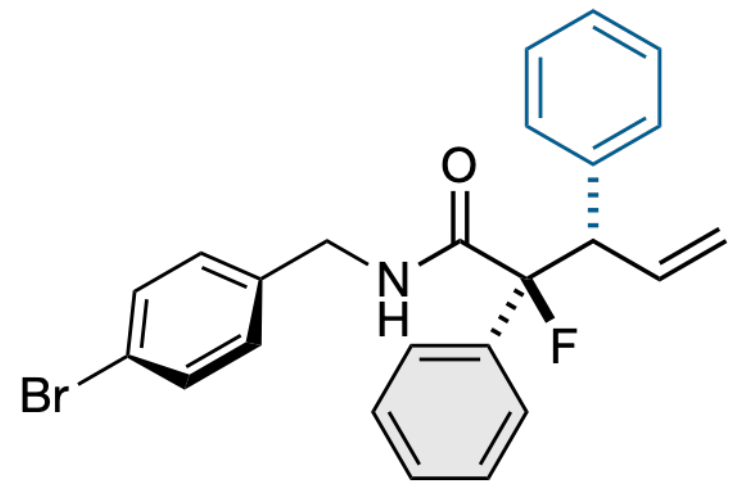


X-ray Single crystals of sufficient quality were obtained by freezing of a saturated solution of **(*S*,*S*)-3-amide** in ether. Single crystal analysis confirmed the absolute stereochemistry as (*S,S*)- and is derived from ($S_a$,*S,S*)-[Ir] catalyst and (*R*)-Me-BTM.

This structure has been deposited at the Cambridge Crystallographic Data Center and can be found under the deposition number: CCDC 2349148.

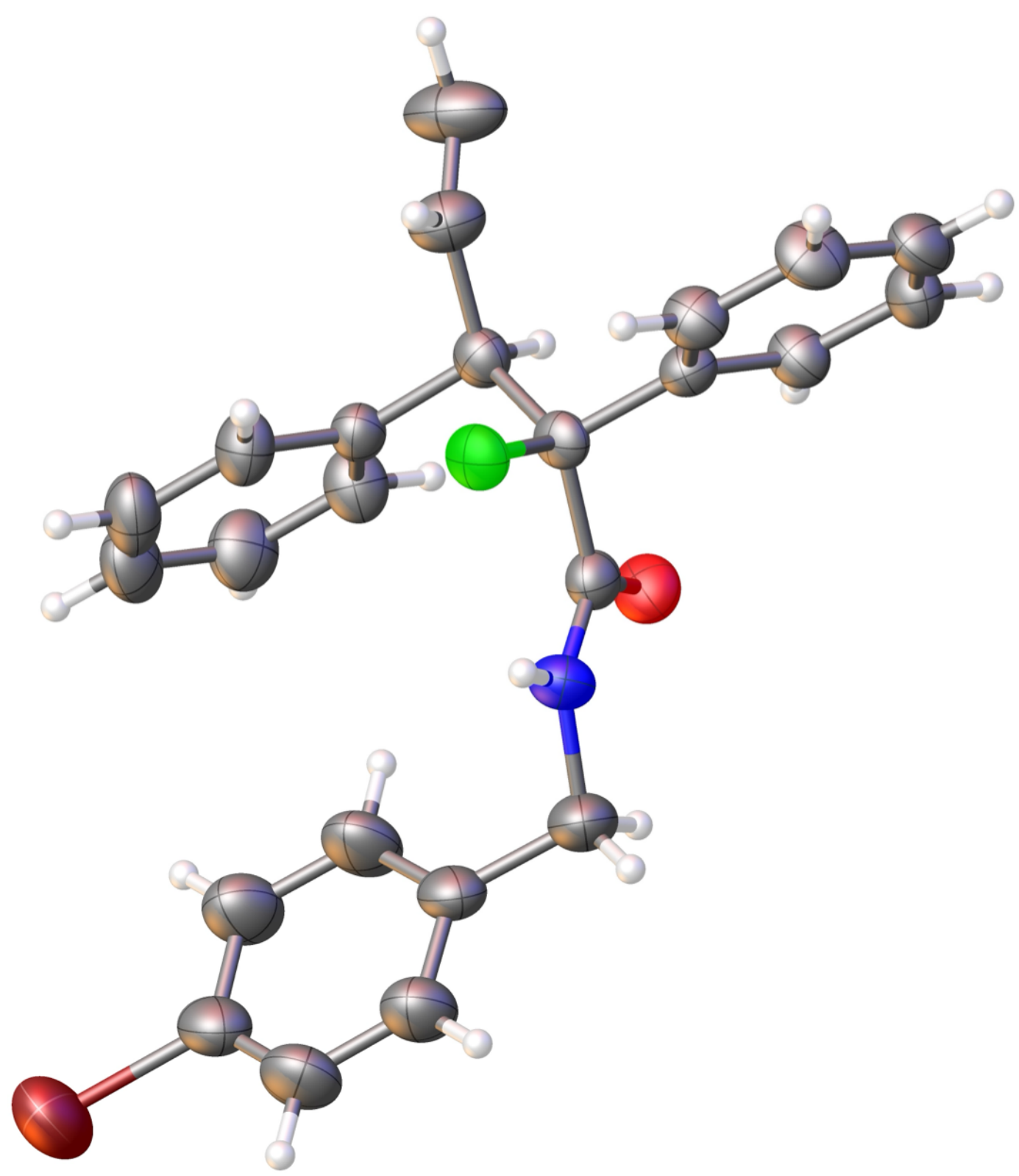

## References


1. D. D. Perrin, W. L. F. Amarego, Purification of Laboratory Chemicals, Pergamon Press, Oxford, Ed. 3, **1988**.
2. Birman, V. B.; Li, X. *Org. Lett.* **2006**, *8* (7), 1351-1354.
3. Lu, X.; Ge, L.; Cheng, C.; Chen, J.; Cao, W.; Wu, X. *Chem. Eur. J*. **2017**, *23*, 7689-7693.
4. Pearson, C. M.; Fyfe, J. W. B.; Snaddon, T. N. *Angew. Chem. Int. Ed.* **2019**, *58*, 10521-10527.
5. Zhang, F.; Song, J. Z. *Tetrahedron Lett.* **2006**, *47*, 7641-7644.
6. Liu, R.; Zhang, Q.; Lin, Z.; Snaddon, T. N.; Zi, W. *Angew. Chem. Int. Ed.* **2025**, *64*, e202504121.
7. Blanchette, M. A.; Choy, W.; Davis, J. T.; Essenfeld, A. P.; Masamune, S.; Roush, W. R.; Sakai, T. *Tetrahedron Lett.* **1984**, *25* (21), 2183-2186.
8. Li, J.-Q.; Peters, B.; Anderson, P. G. *Chem. Eur. J.* **2011**, *17*, 11143-11145.
9. Weix, D. J.; Marković , D.; Ueda, M.; Hartwig, J. F. *Org. Lett.* **2009**, *11* (13), 2944-2947.
10. Panne, P.; Fox, J. M. *J. Am. Chem. Soc.* **2007**, *129* (1), 22-23.

**Perfluorophenyl (2*R*,3*R*)-2-fluoro-2,3-diphenylpent-4-enoate (*R*,*R*)-3):**

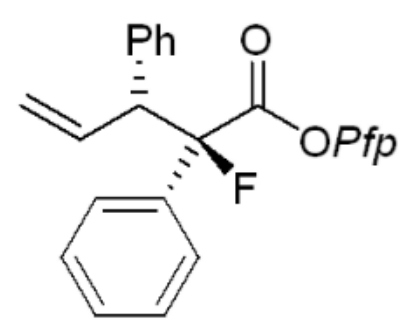


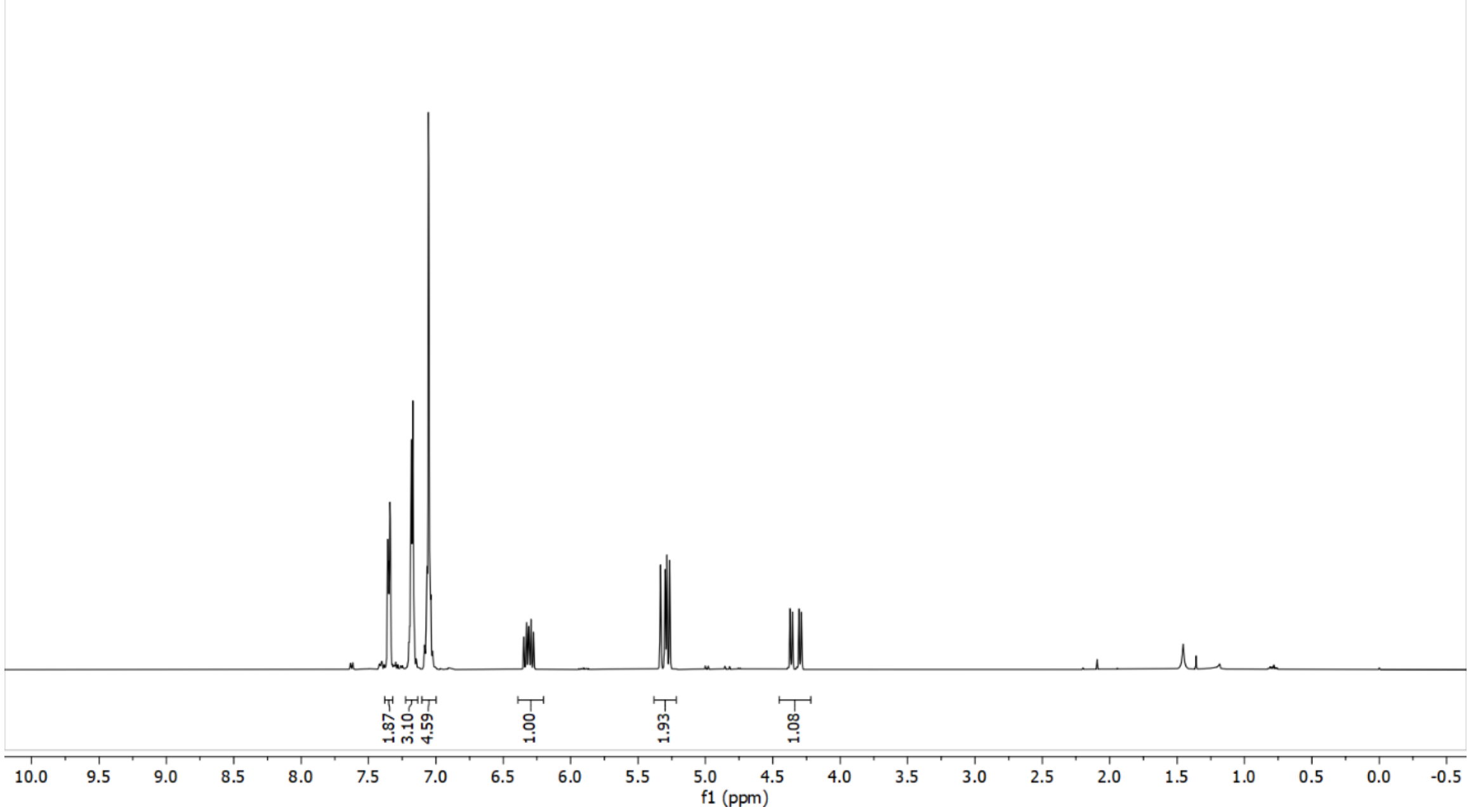


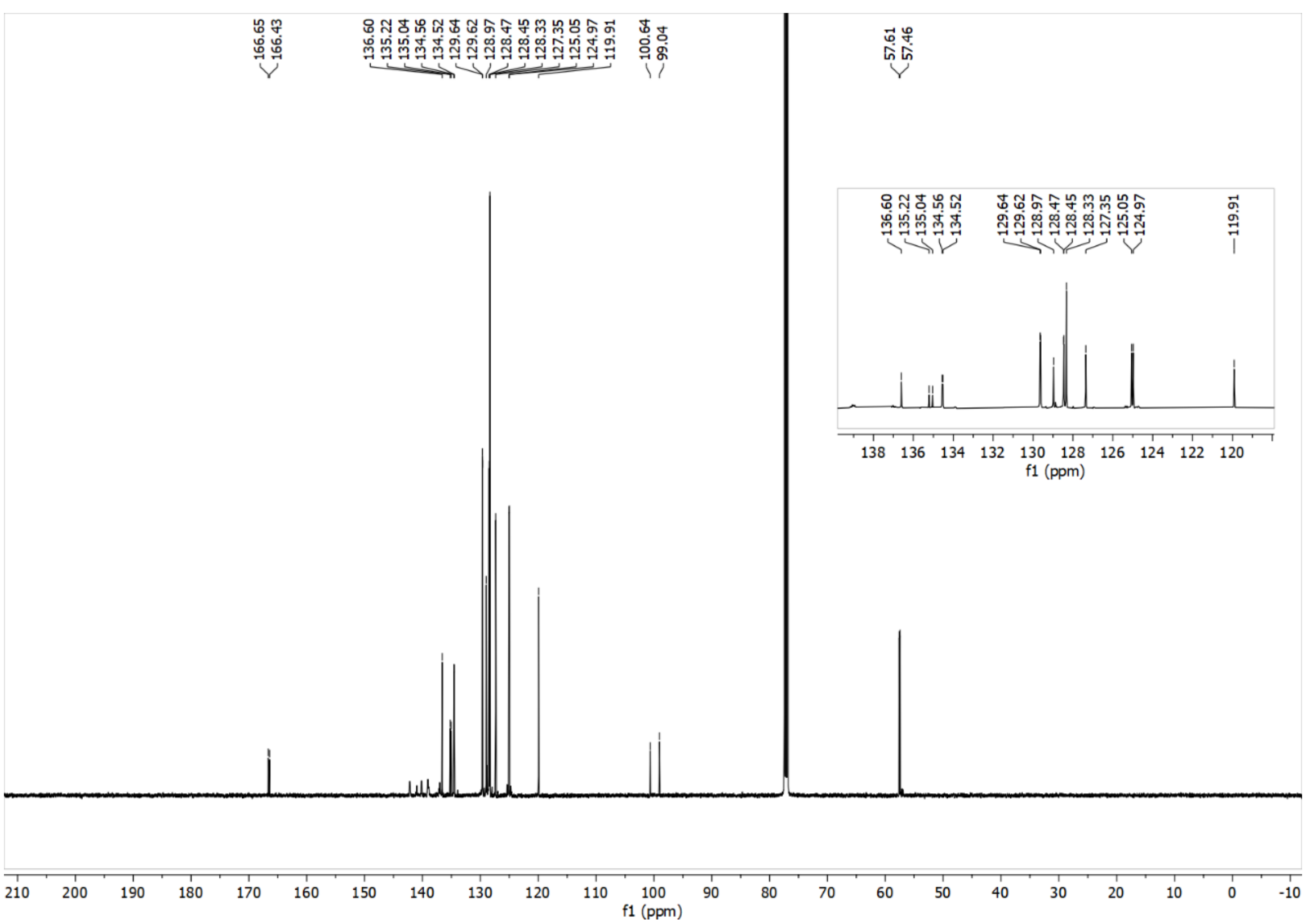

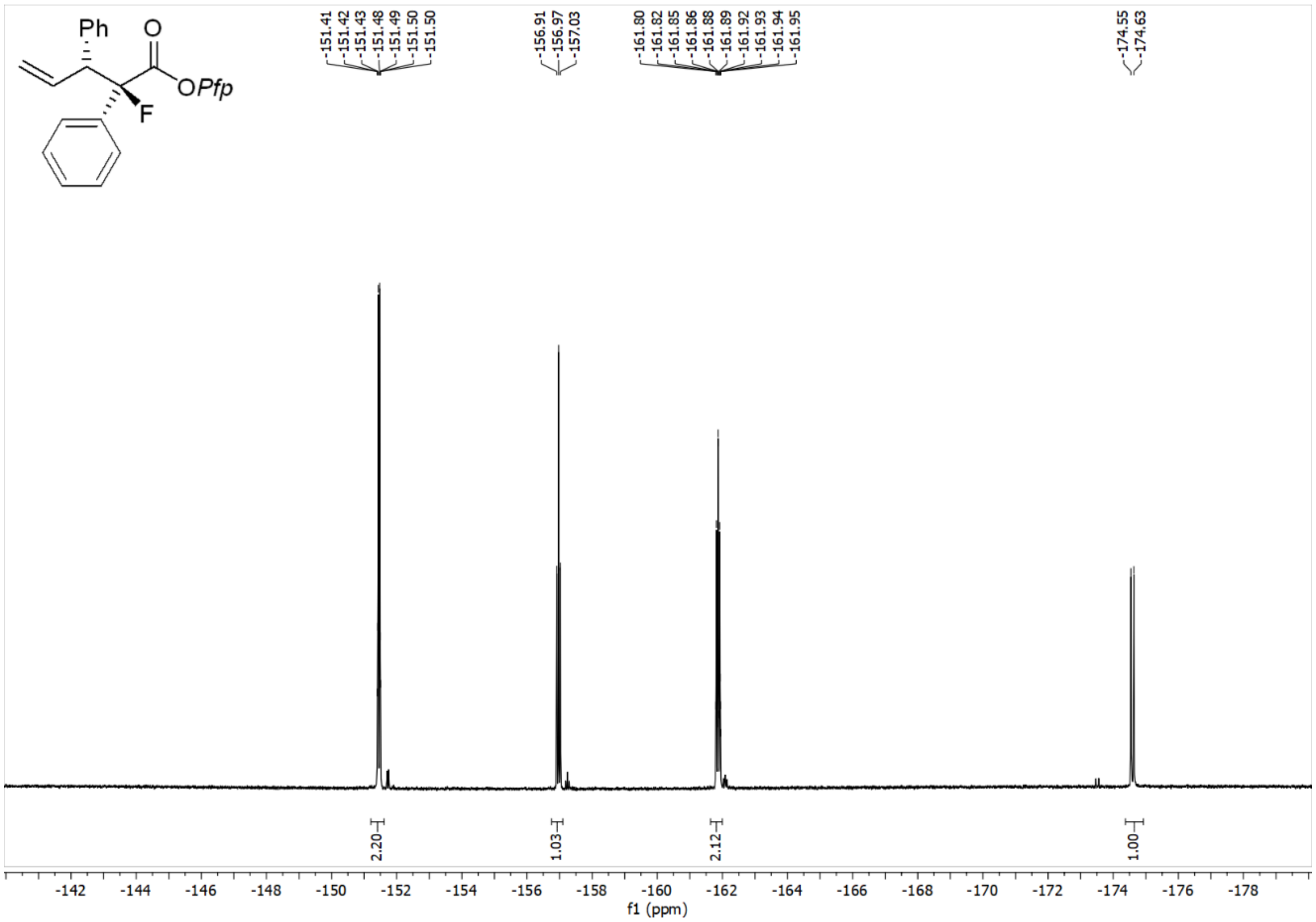
Ph
O
OPfp
F
-151.41
-151.42
-151.43
-151.48
-151.49
-151.50
-151.50
-156.91
-156.97
-157.03
-161.80
-161.82
-161.85
-161.86
-161.88
-161.89
-161.92
-161.93
-161.94
-161.95
-174.55
-174.63
2.20
1.03
2.12
1.00
-142 -144 -146 -148 -150 -152 -154 -156 -158 -160 -162 -164 -166 -168 -170 -172 -174 -176 -178
f1 (ppm)

**(2*R*,3*R*)-N-(4-bromobenzyl)-2-fluoro-2,3-diphenylpent-4-enamide (*R*,*R*)-3-amide):**

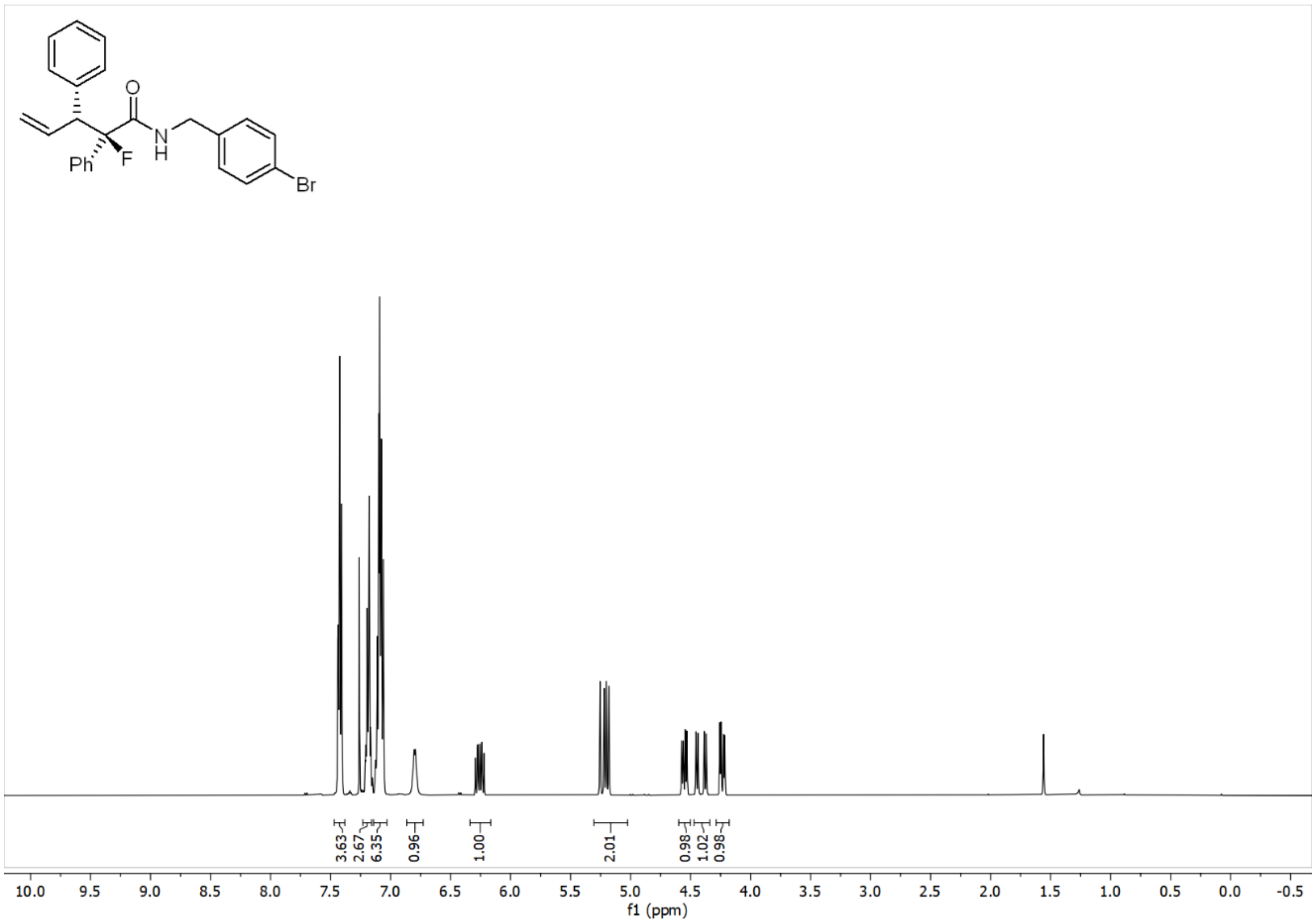

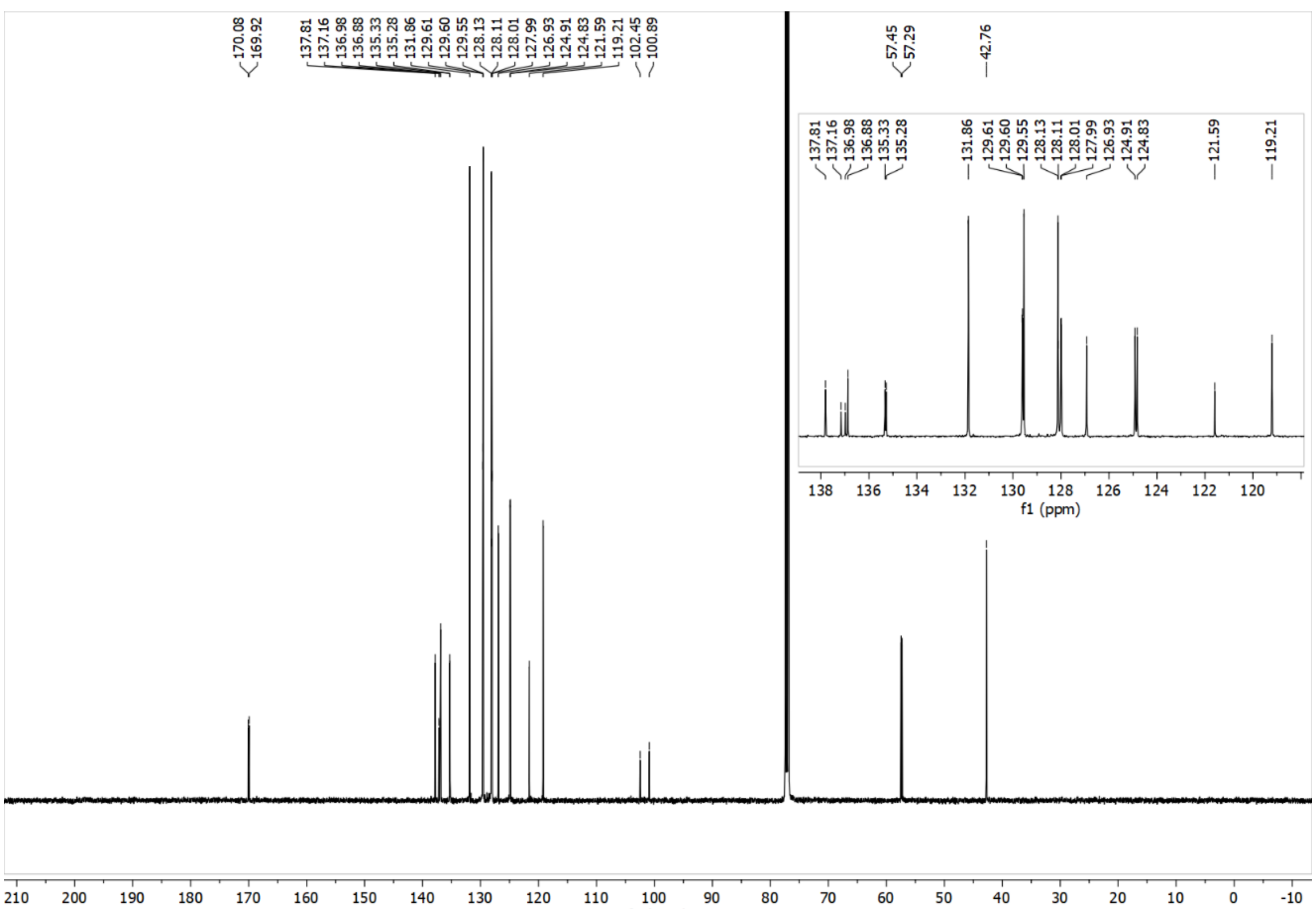

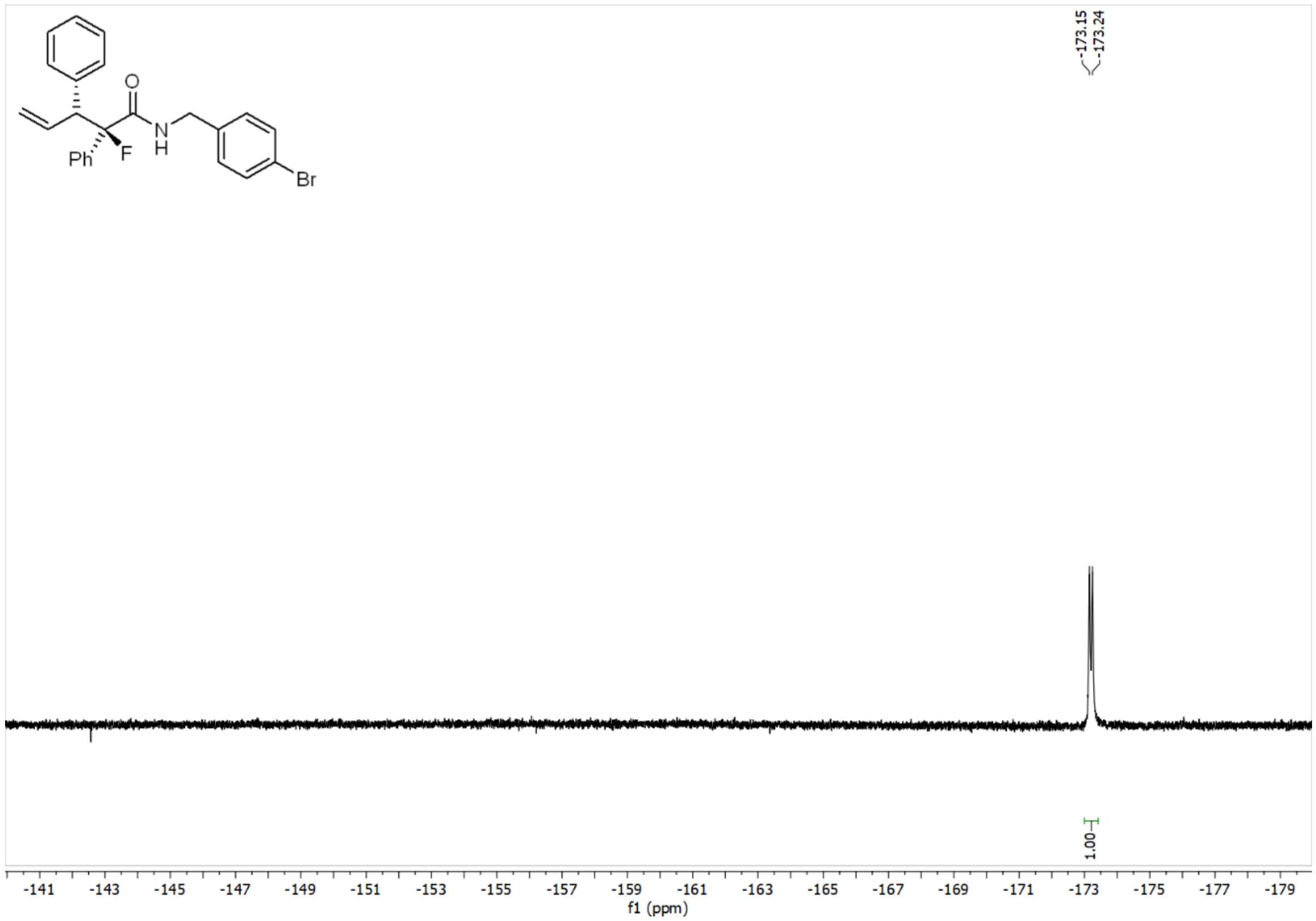

Ph
F
O
N
H
Br
-173.15
-173.24
1.00
-141
-143
-145
-147
-149
-151
-153
-155
-157
-159
-161
-163
-165
-167
-169
-171
-173
-175
-177
-179
f1 (ppm)

**Perfluorophenyl (2*R*,3*S*)-2-fluoro-2,3-diphenylpent-4-enoate (*R*,*S*)-3):**

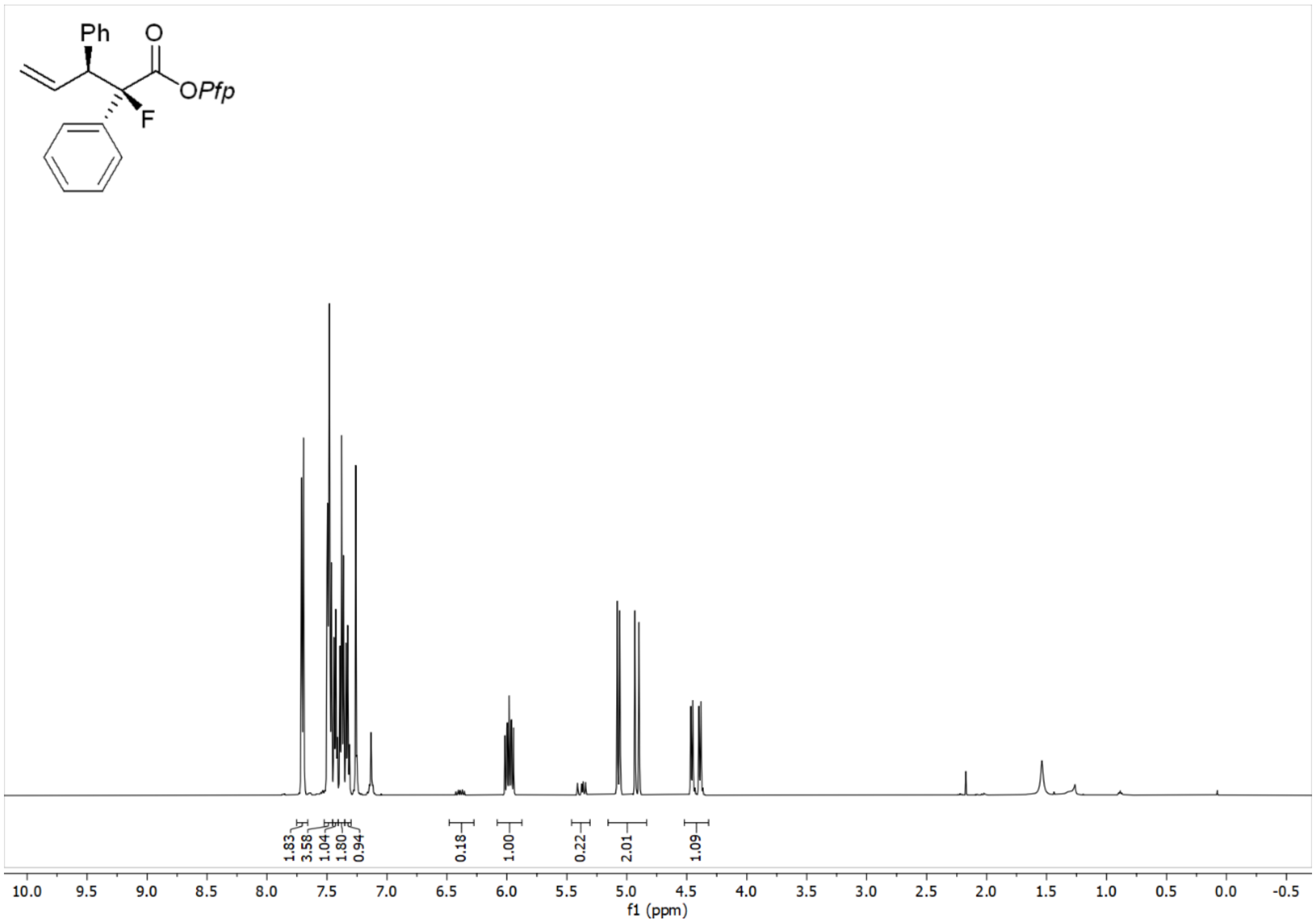


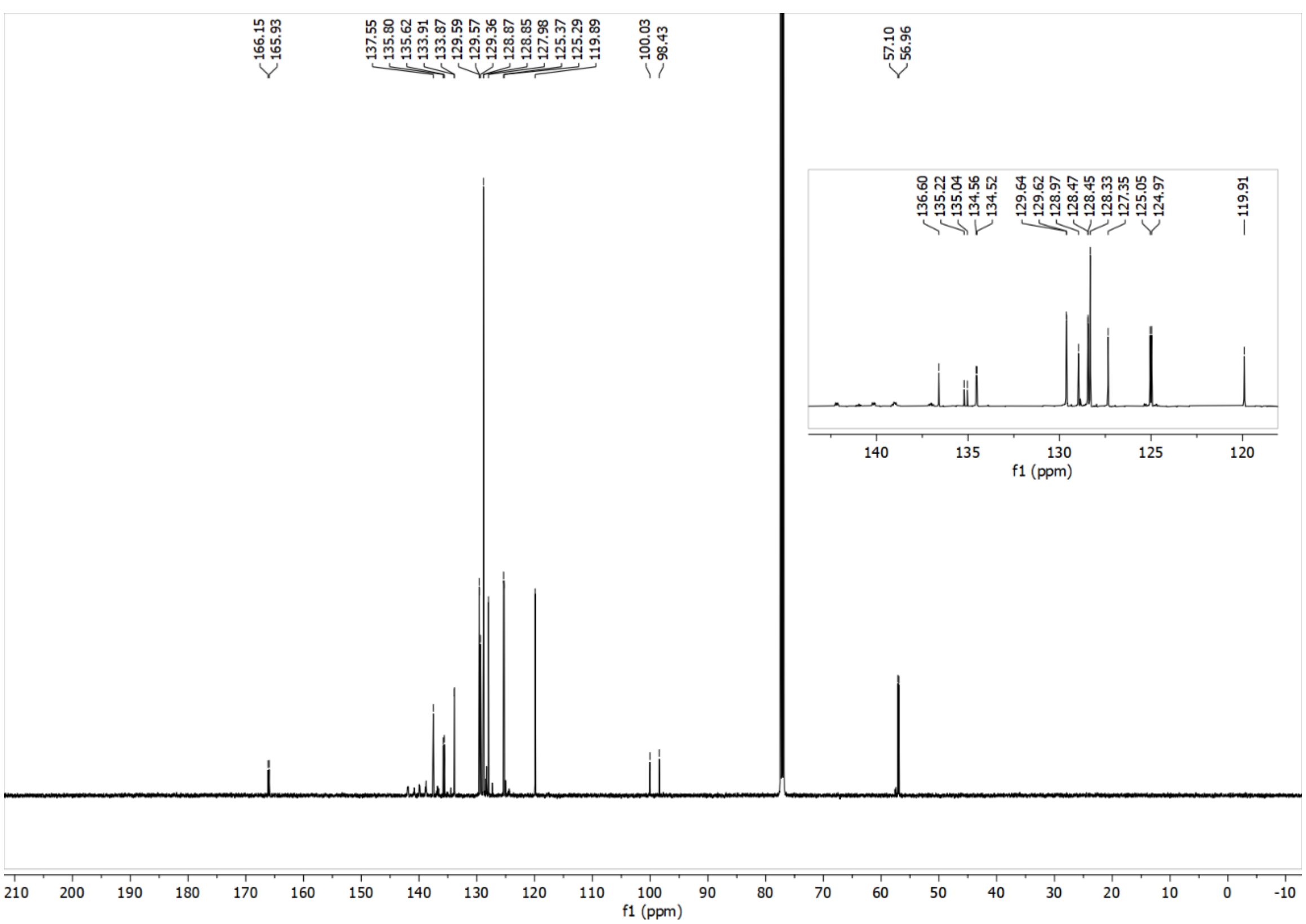

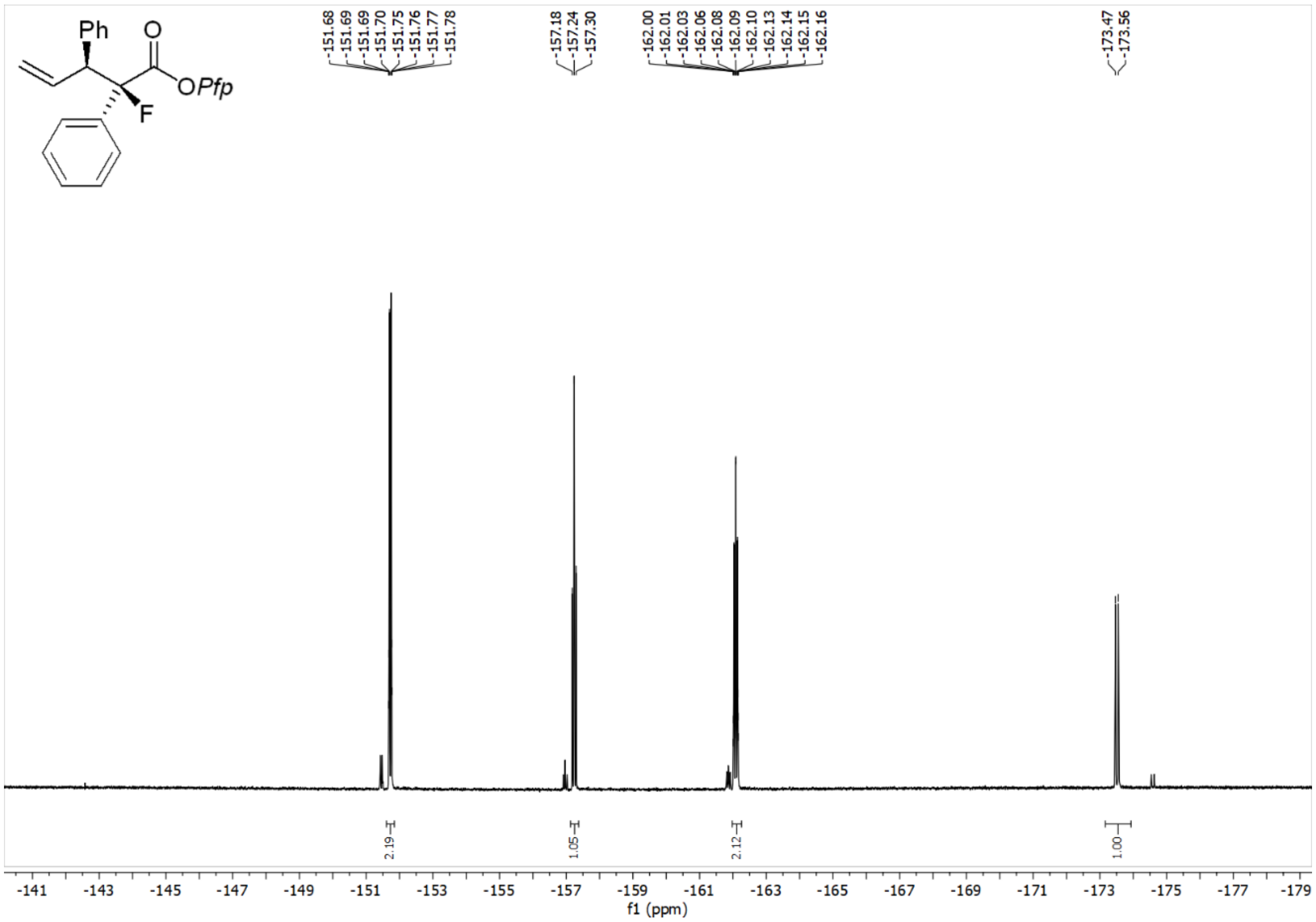
Ph
O
OPfp
F
-151.68
-151.69
-151.69
-151.70
-151.75
-151.76
-151.77
-151.78
-157.18
-157.24
-157.30
-162.00
-162.01
-162.03
-162.06
-162.08
-162.09
-162.10
-162.13
-162.14
-162.15
-162.16
-173.47
-173.56
2.19
1.05
2.12
1.00
-141 -143 -145 -147 -149 -151 -153 -155 -157 -159 -161 -163 -165 -167 -169 -171 -173 -175 -177 -179
f1 (ppm)

**(2*R*,3*S*)-N-(4-bromobenzyl)-2-fluoro-2,3-diphenylpent-4-enamide (*R*,*S*)-3-amide):**

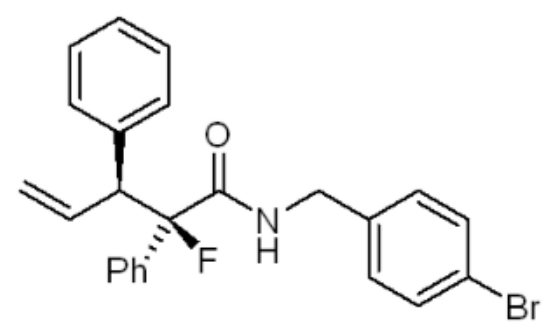


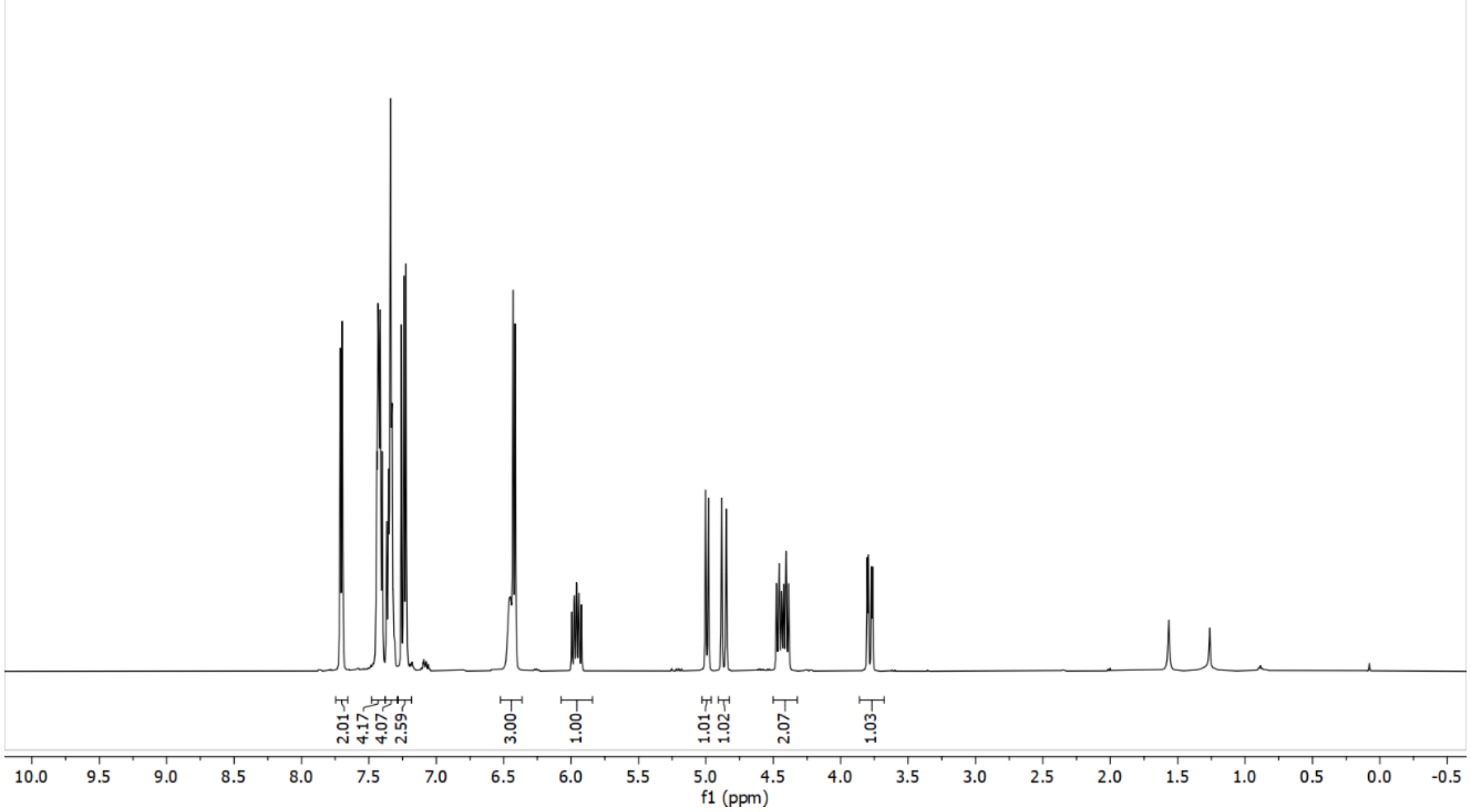

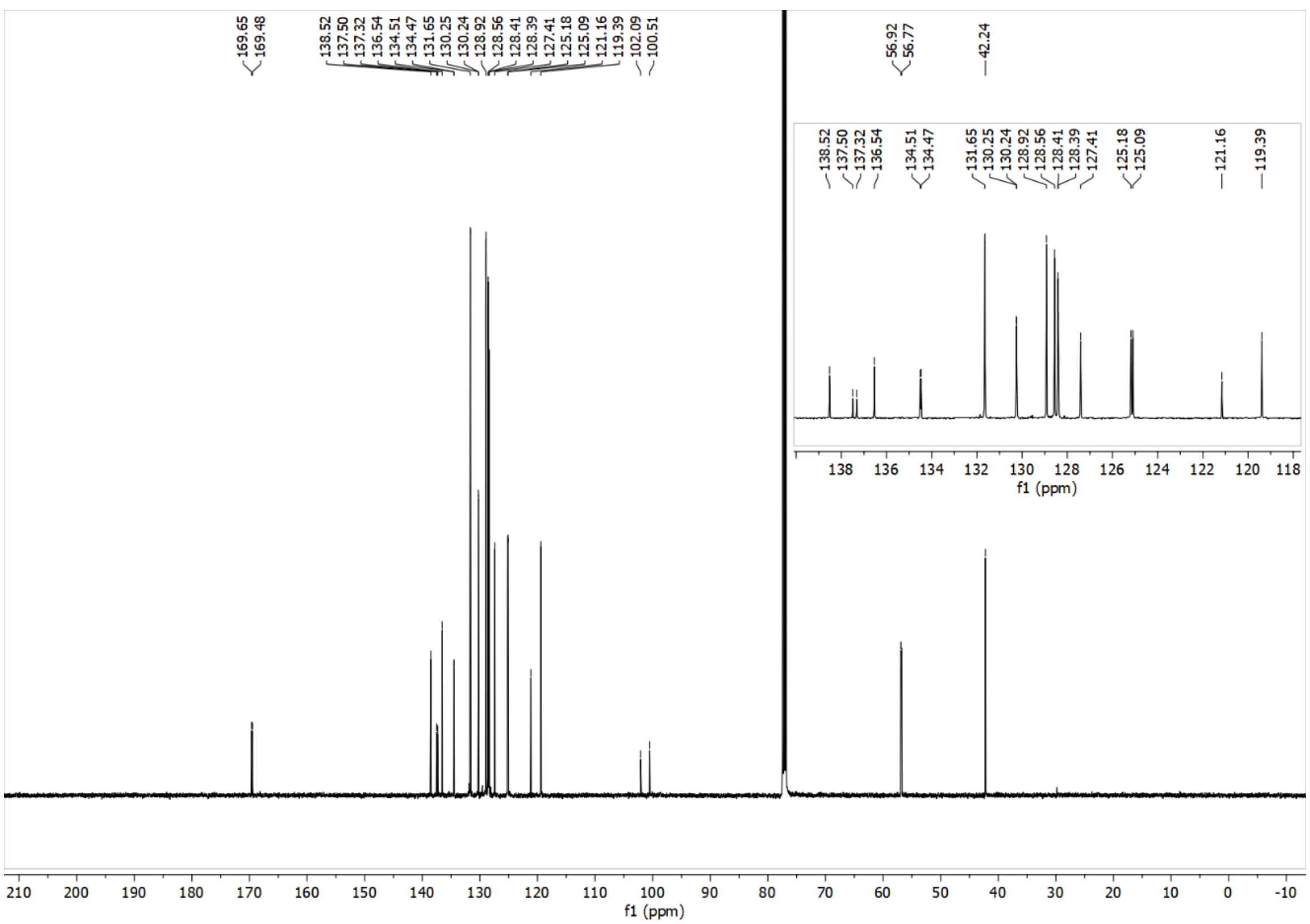

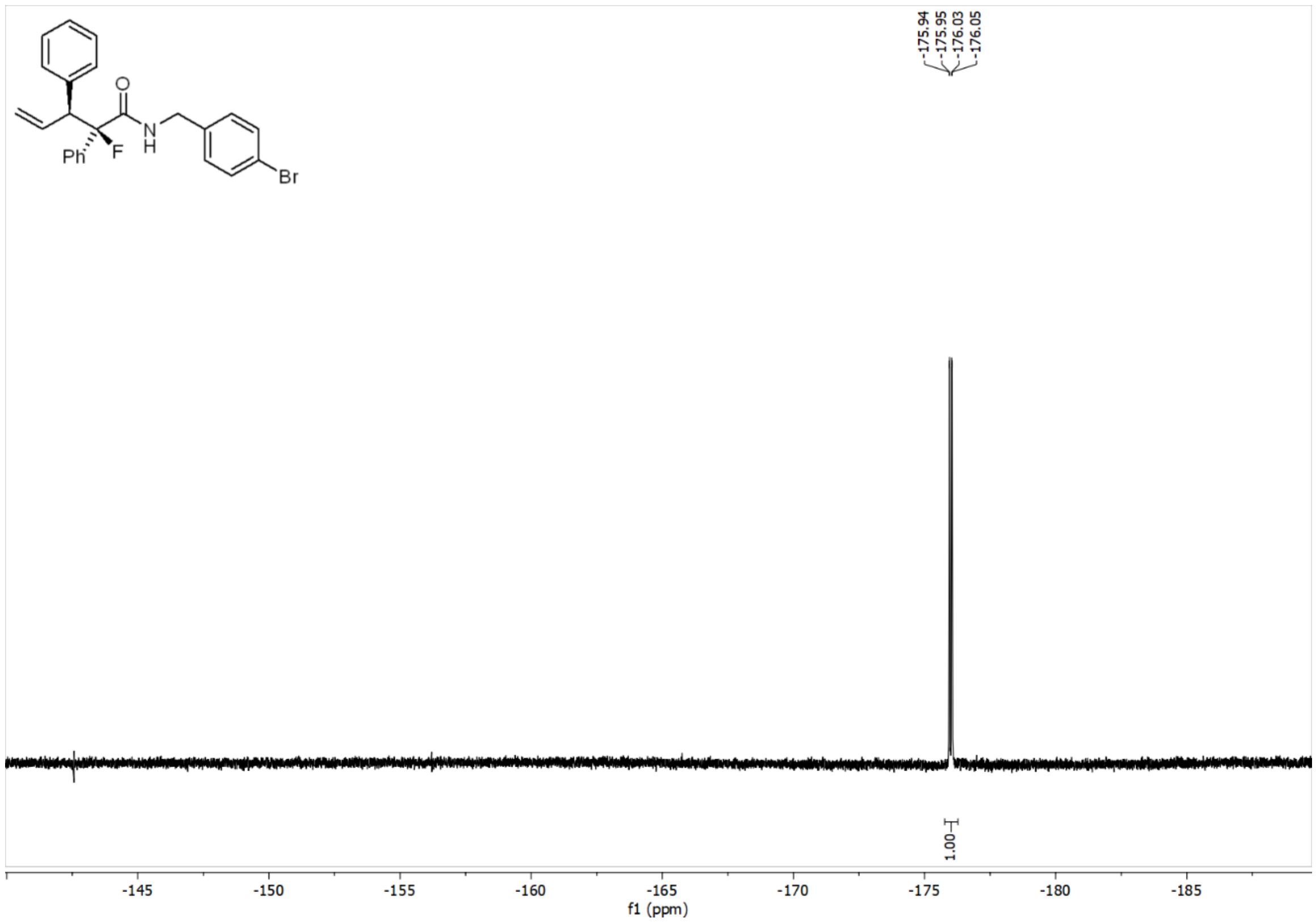

-175.94
-175.95
-176.03
-176.05
O
N
H
Ph
F
Br
1.00
-145
-150
-155
-160
-165
-170
-175
-180
-185
f1 (ppm)

**Perfluorophenyl (2*S*,3*R*)-2-fluoro-2,3-diphenylpent-4-enoate (*S*,*R*)-3):**

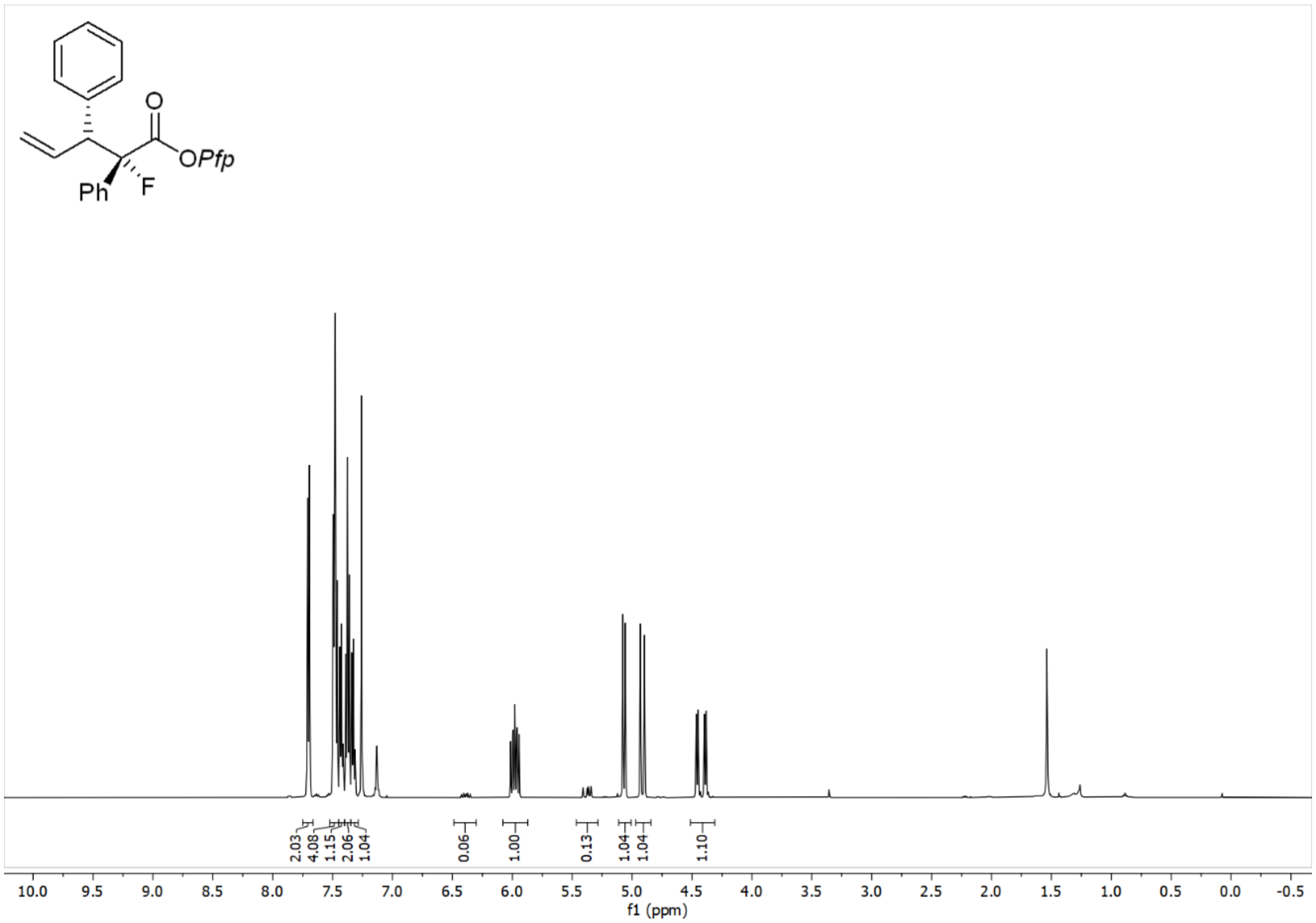


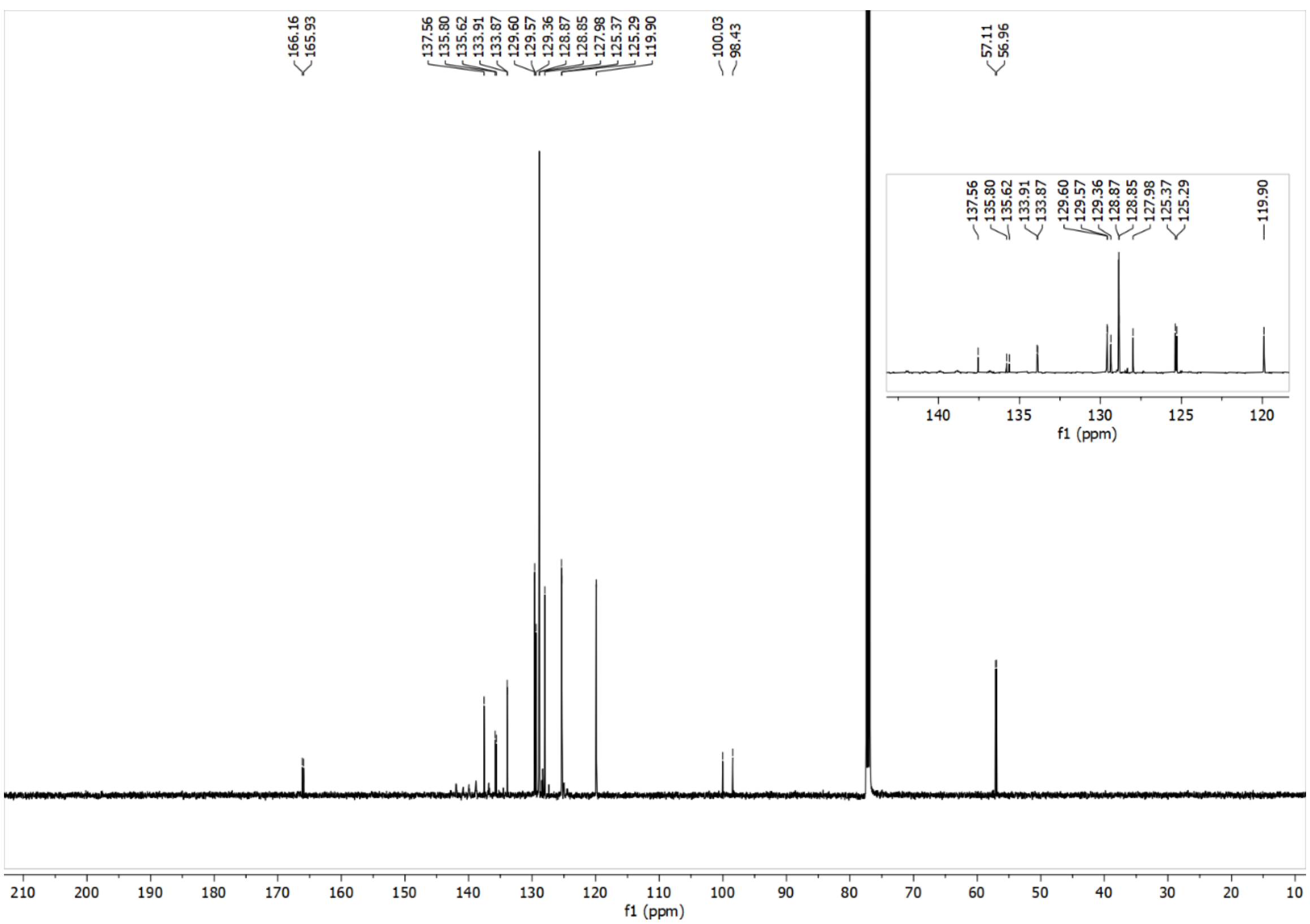

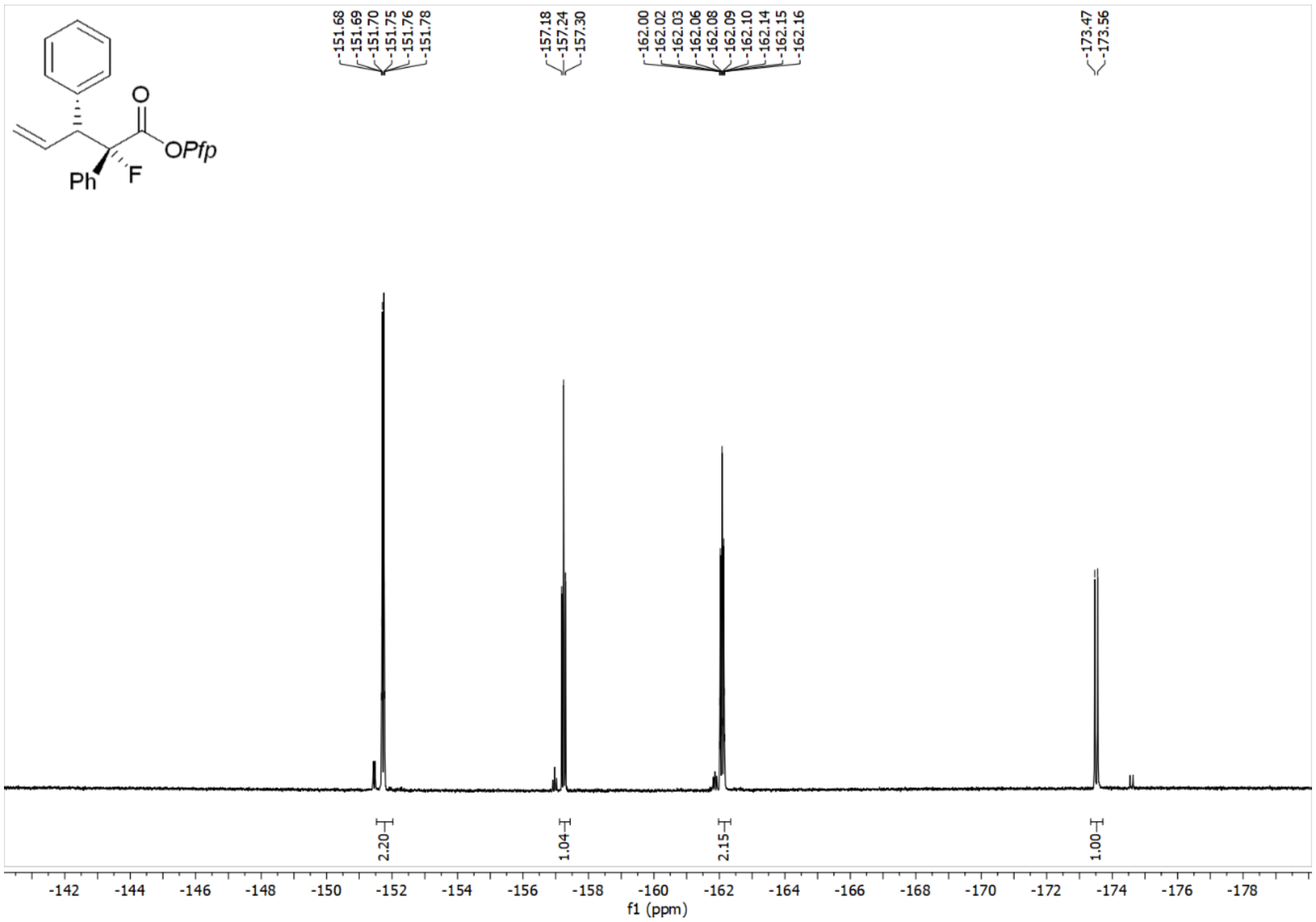

OPfp
Ph
F
-151.68
-151.69
-151.70
-151.75
-151.76
-151.78
-157.18
-157.24
-157.30
-162.00
-162.02
-162.03
-162.06
-162.08
-162.09
-162.10
-162.14
-162.15
-162.16
-173.47
-173.56
2.20
1.04
2.15
1.00
-142
-144
-146
-148
-150
-152
-154
-156
-158
-160
-162
-164
-166
-168
-170
-172
-174
-176
-178
f1 (ppm)

**(2*S*,3*R*)-N-(4-bromobenzyl)-2-fluoro-2,3-diphenylpent-4-enamide (*S*,*R*)-3-amide):**

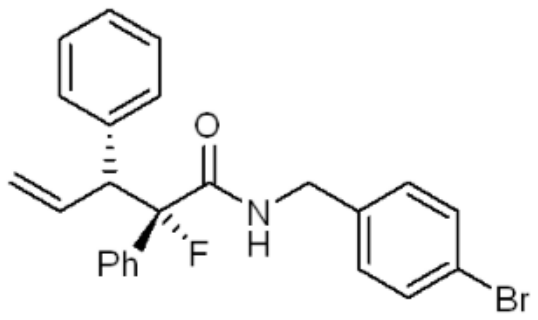


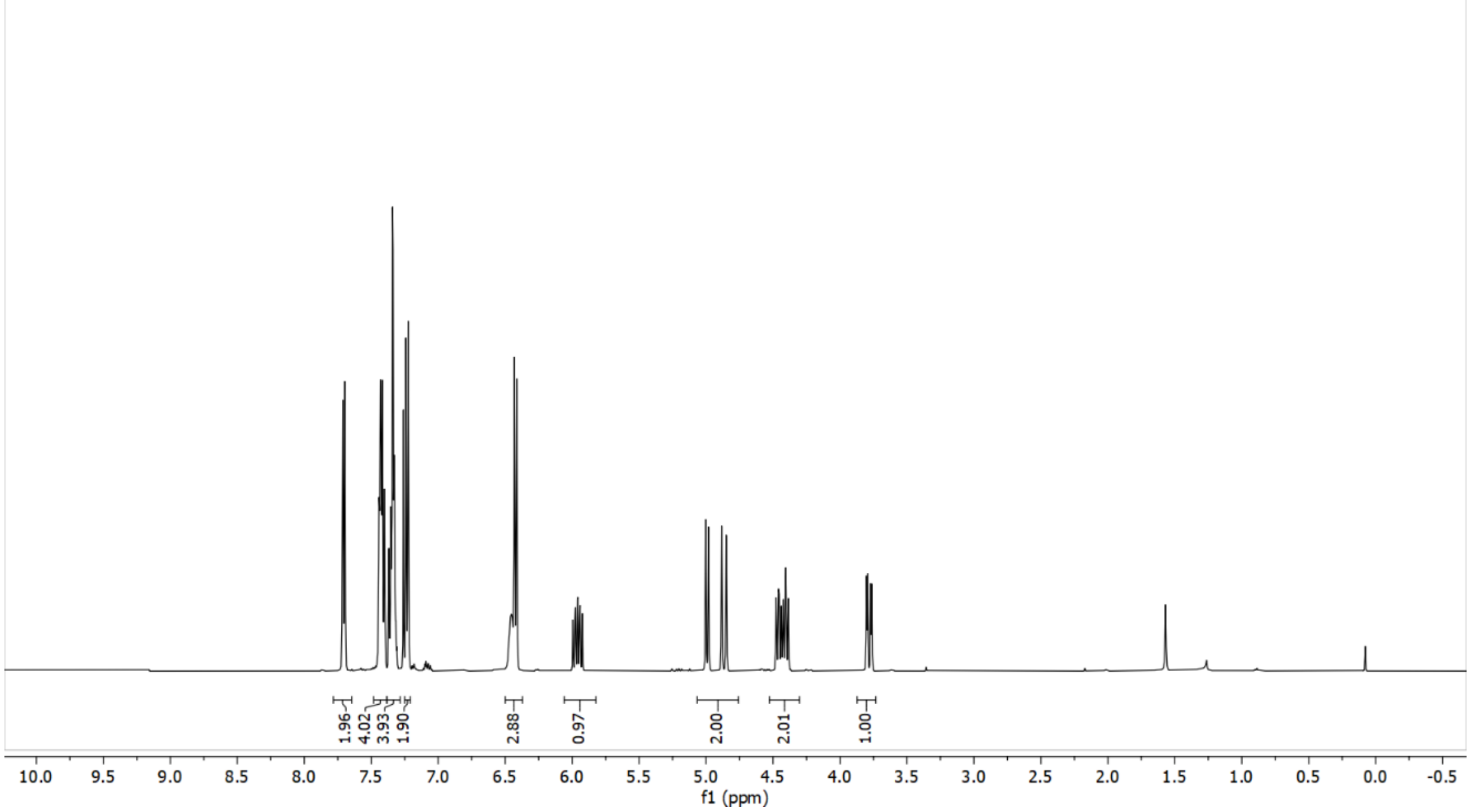

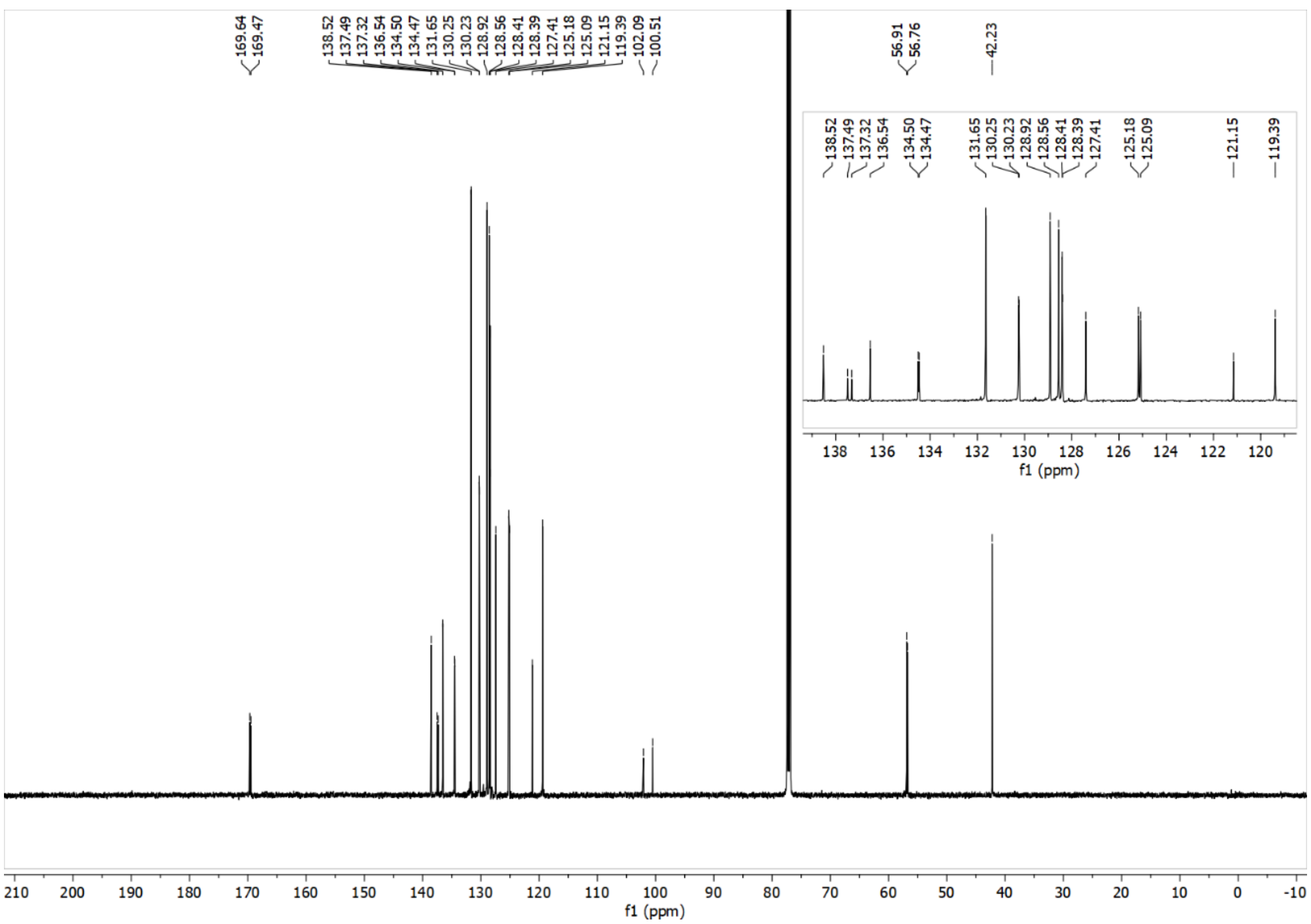

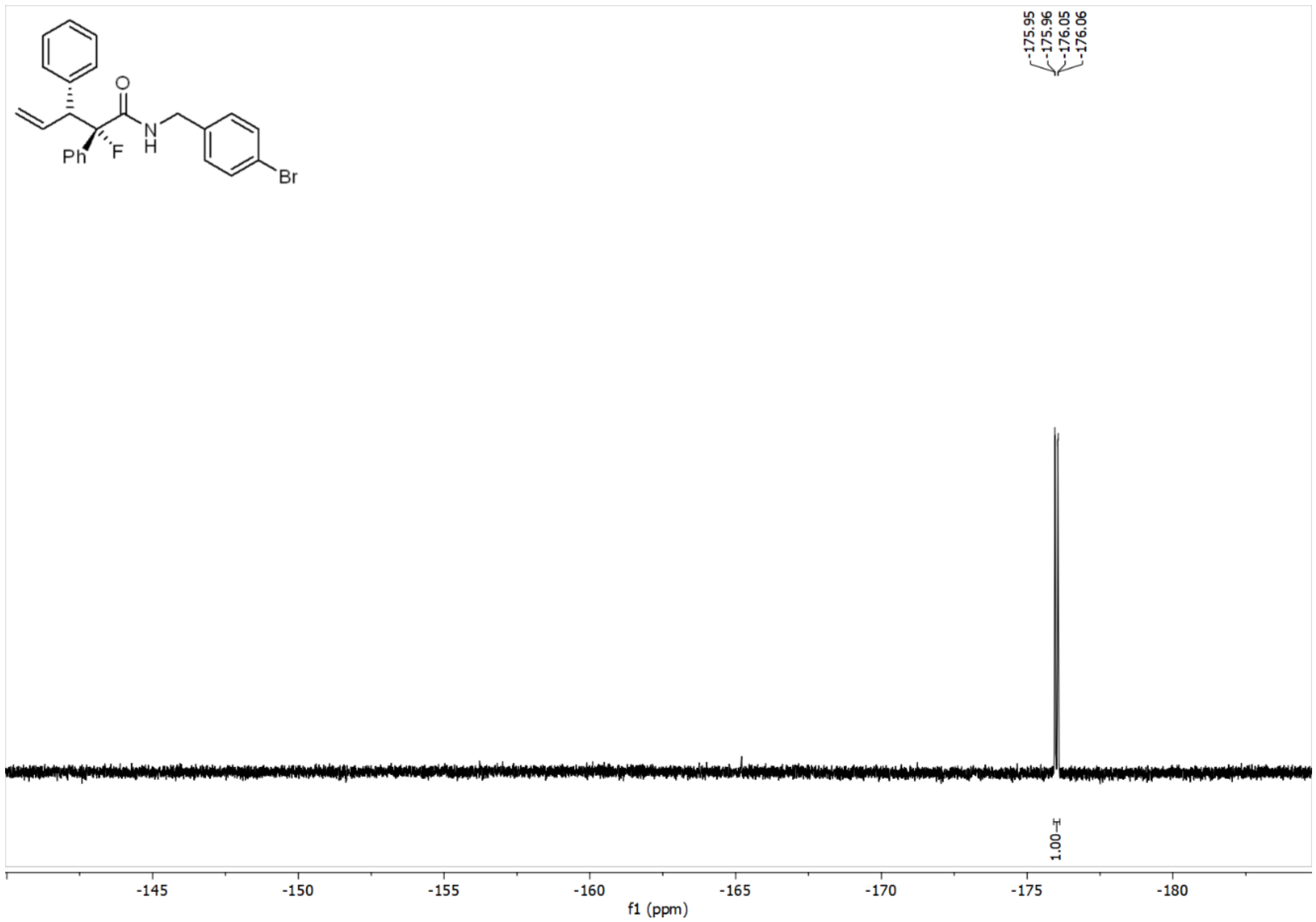

-175.95
-175.96
-176.05
-176.06
1.00
-145
-150
-155
-160
-165
-170
-175
-180
f1 (ppm)
O
N
H
Ph
F
Br

**Perfluorophenyl (2*S*,3*S*)-2-fluoro-2,3-diphenylpent-4-enoate (*S*,*S*)-3):**

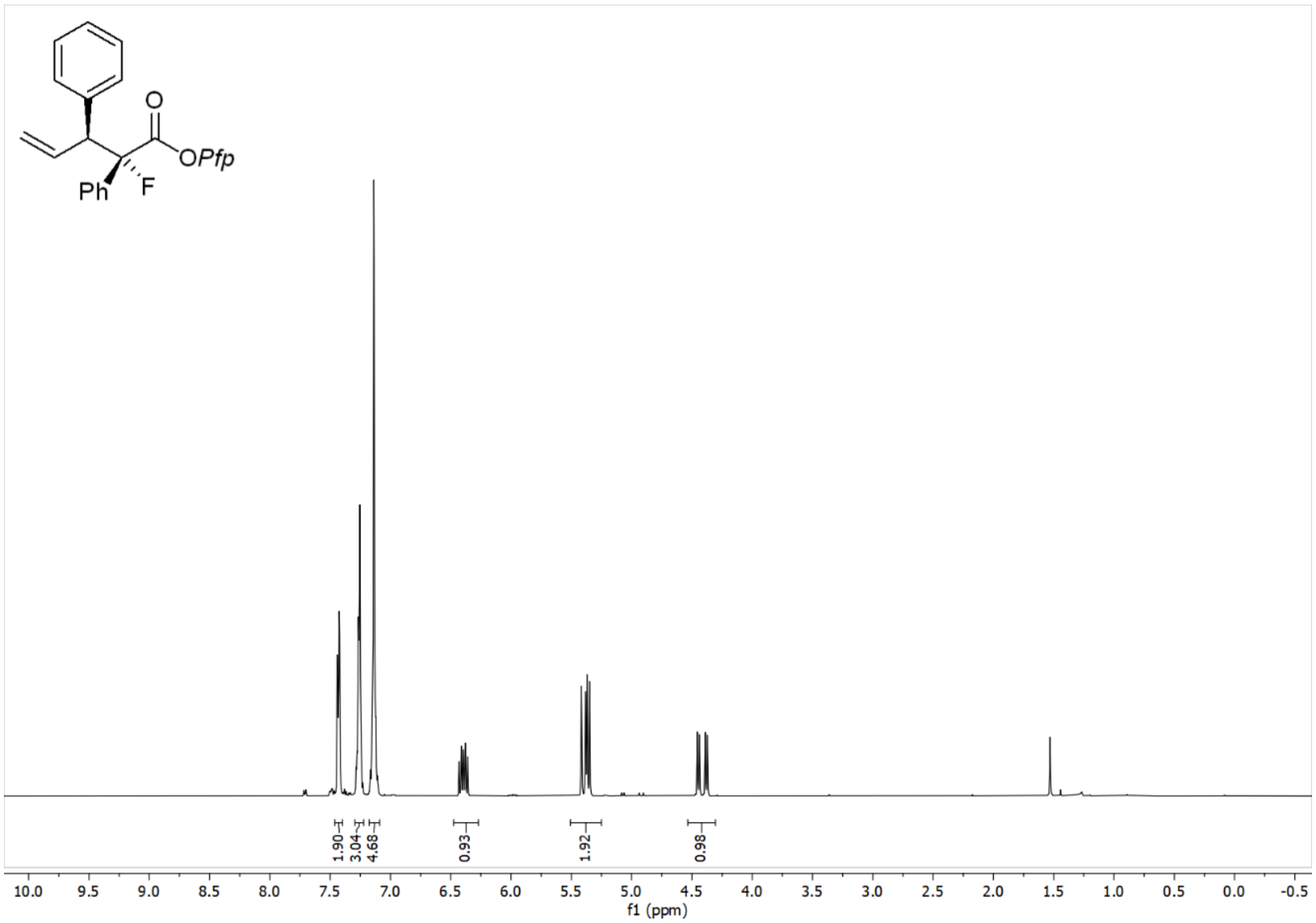


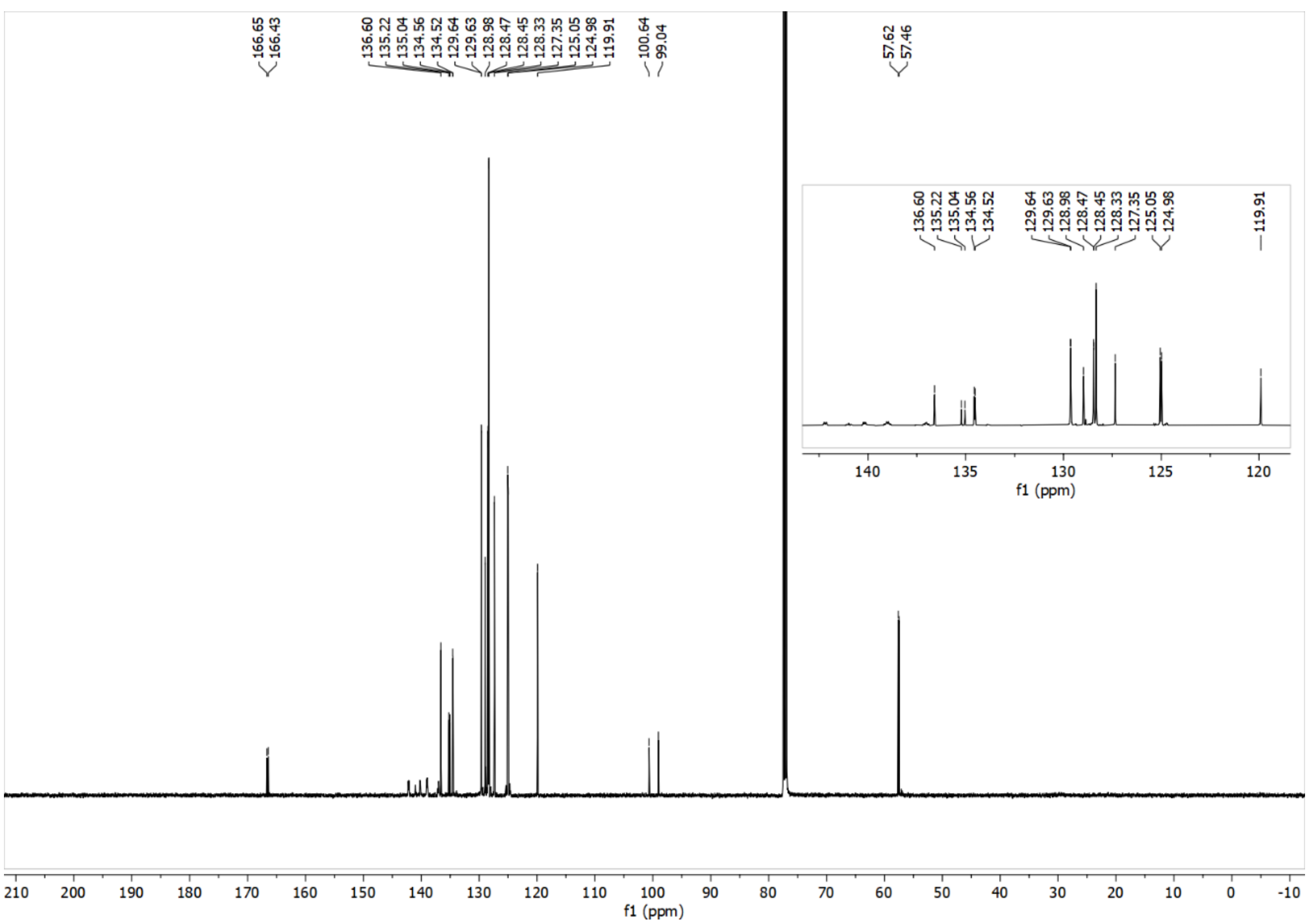

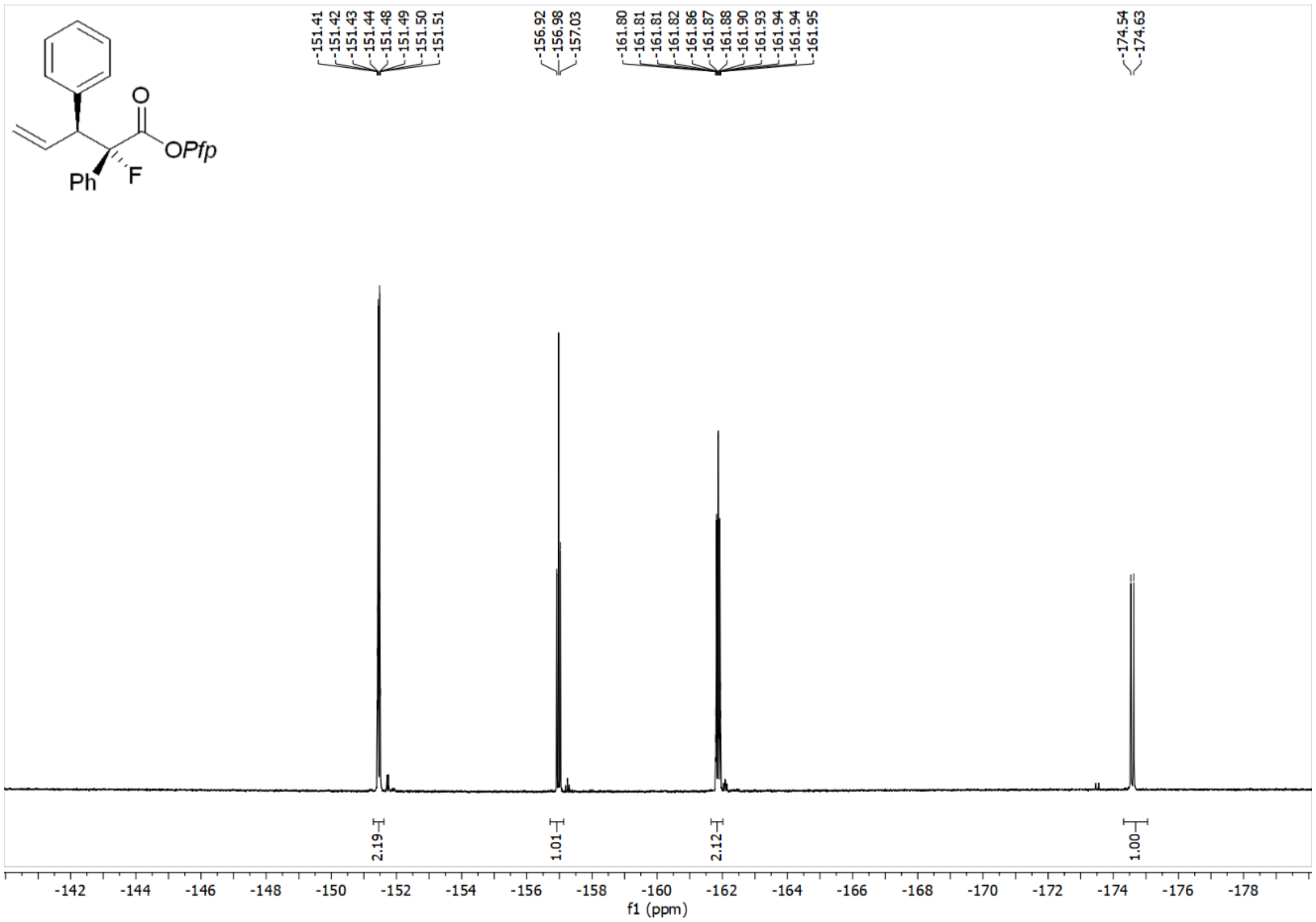

OPfp
Ph
F
-151.41
-151.42
-151.43
-151.44
-151.48
-151.49
-151.50
-151.51
-156.92
-156.98
-157.03
-161.80
-161.81
-161.81
-161.82
-161.86
-161.87
-161.88
-161.90
-161.93
-161.94
-161.94
-161.95
-174.54
-174.63
2.19
1.01
2.12
1.00
-142
-144
-146
-148
-150
-152
-154
-156
-158
-160
-162
-164
-166
-168
-170
-172
-174
-176
-178
f1 (ppm)

**(2*S*,3*S*)-N-(4-bromobenzyl)-2-fluoro-2,3-diphenylpent-4-enamide (*S*,*S*)-3-amide)**

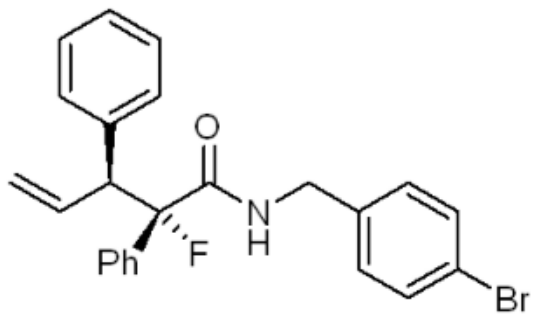


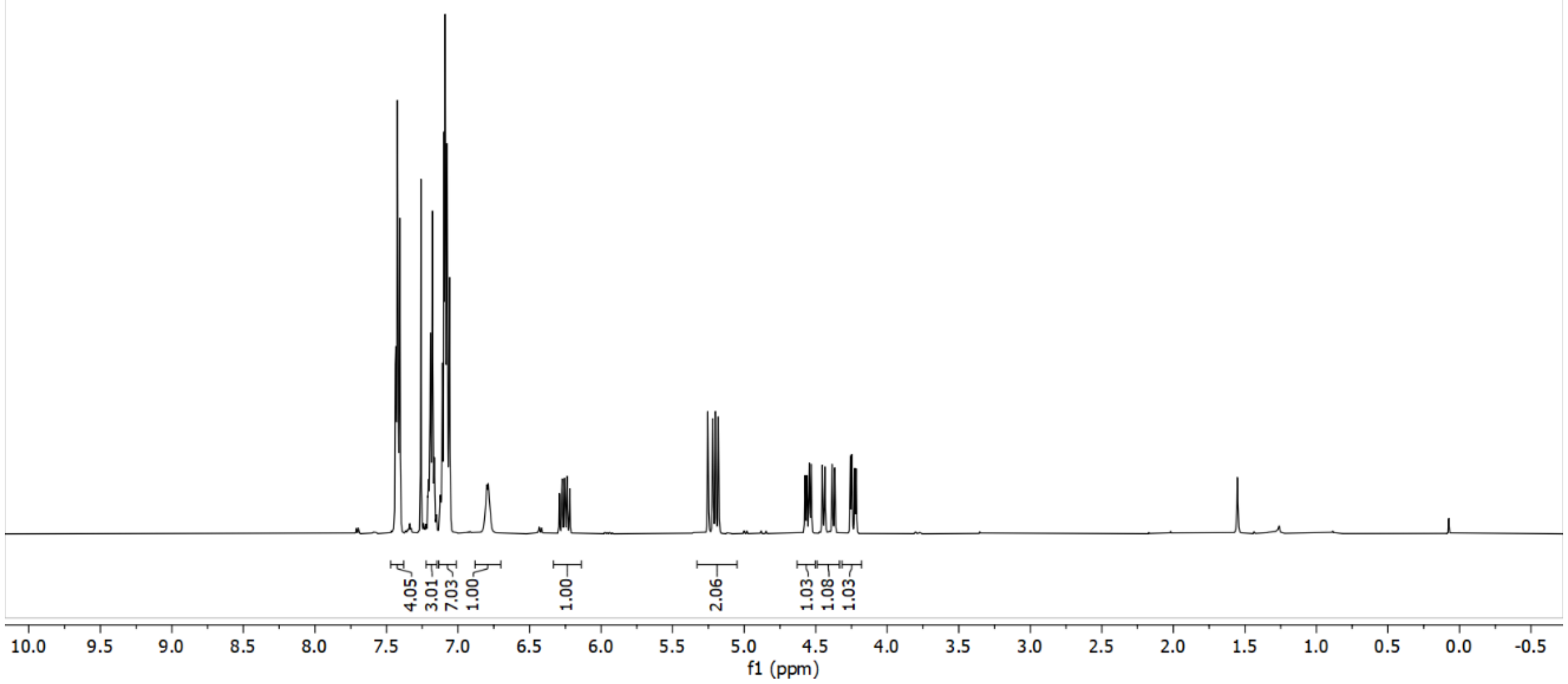

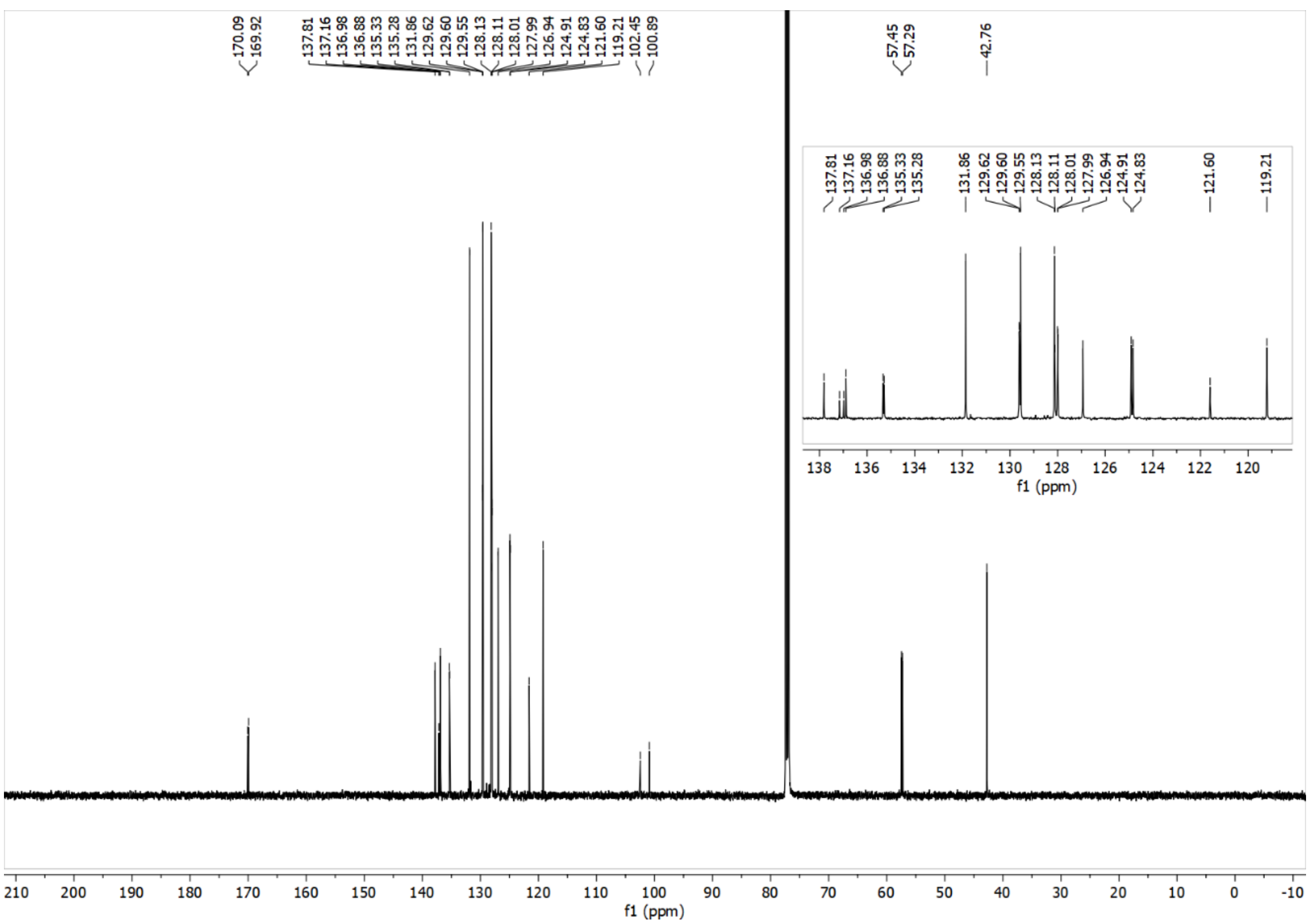

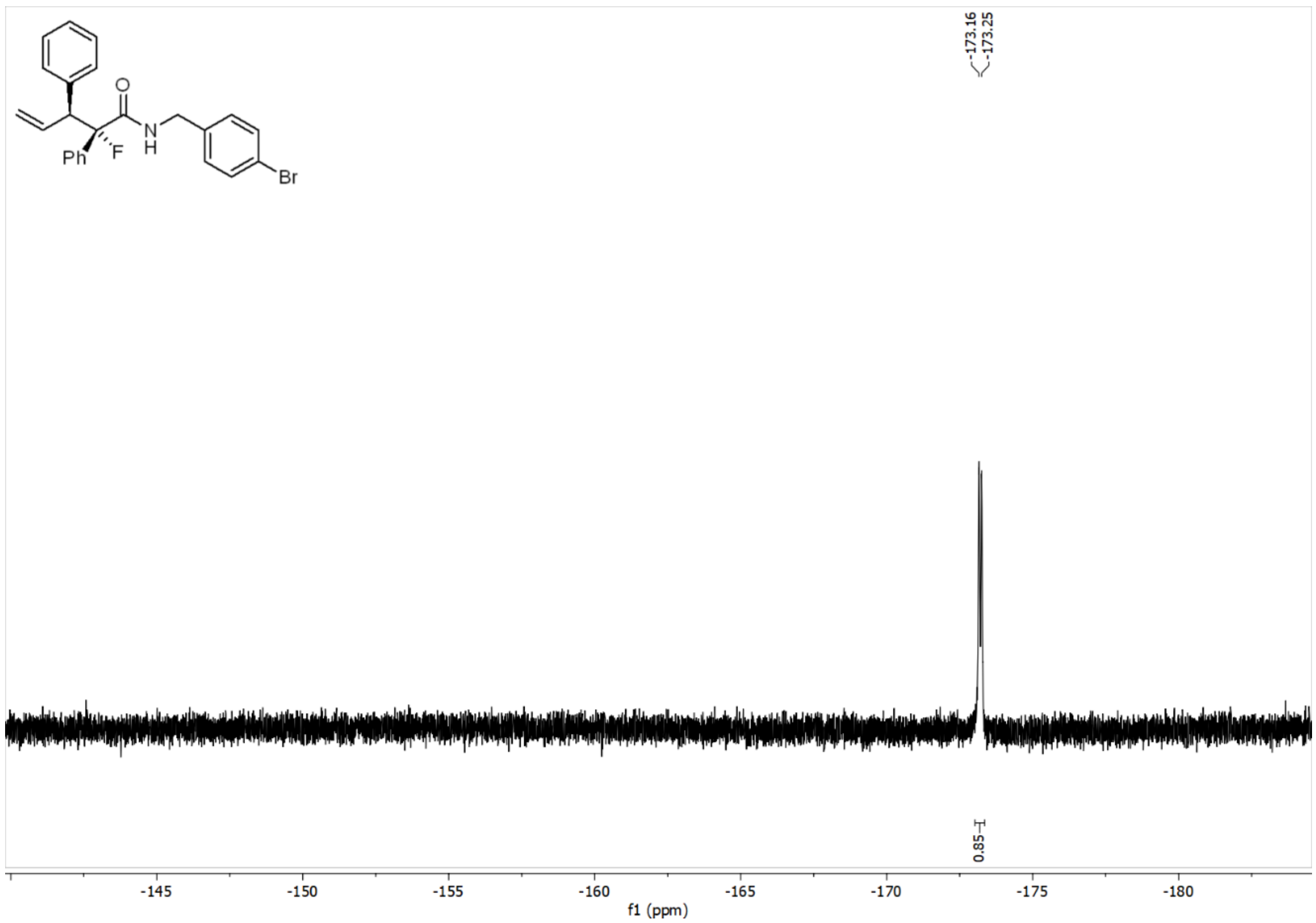

-173.16
-173.25
0.85
-145
-150
-155
-160
-165
-170
-175
-180
f1 (ppm)
Ph
F
O
N
H
Br

**Perfluorophenyl (2*R*,3*R*)-2-fluoro-3-phenyl-2-(p-tolyl)pent-4-enoate (*R*,*R*)-7):**

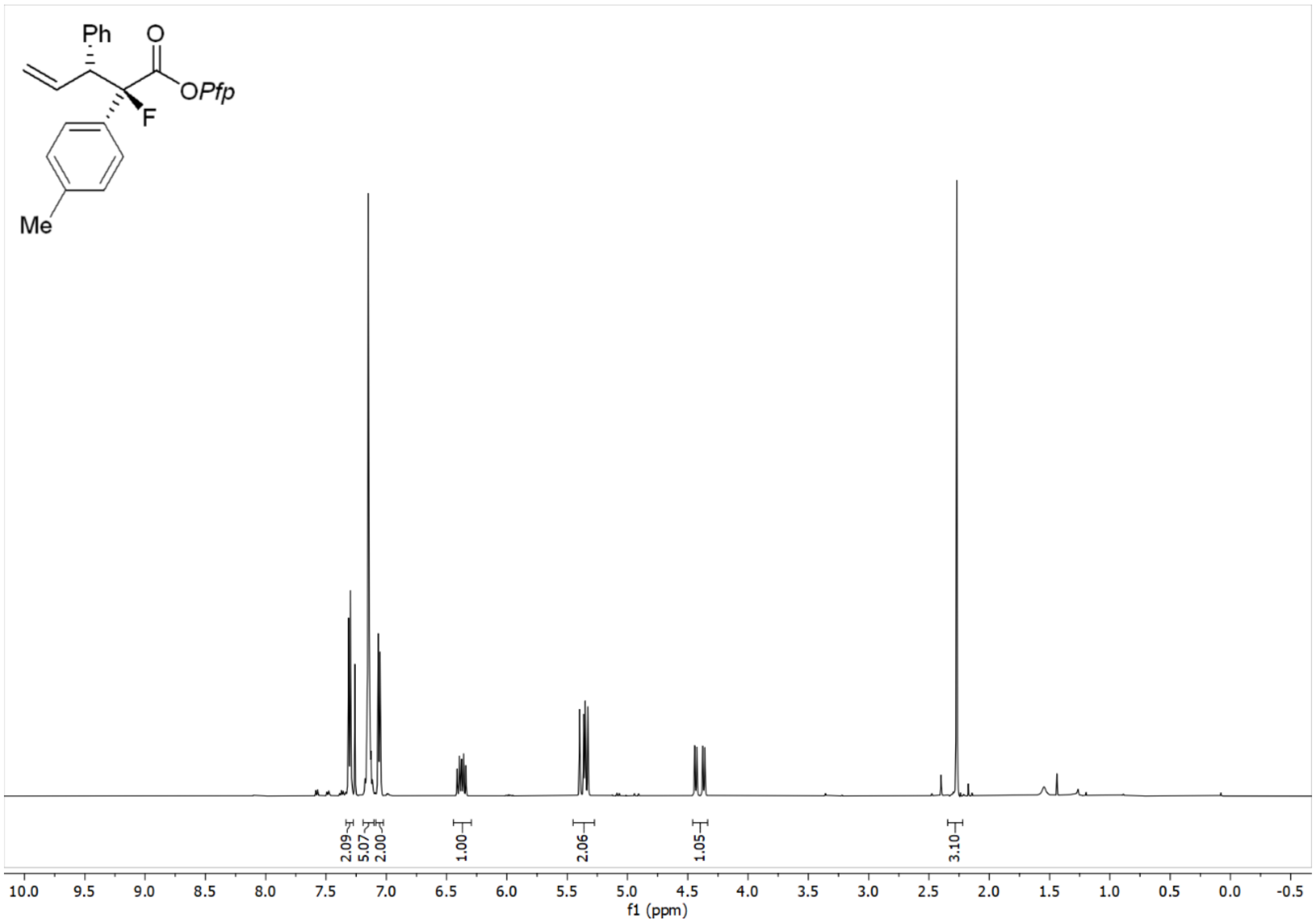


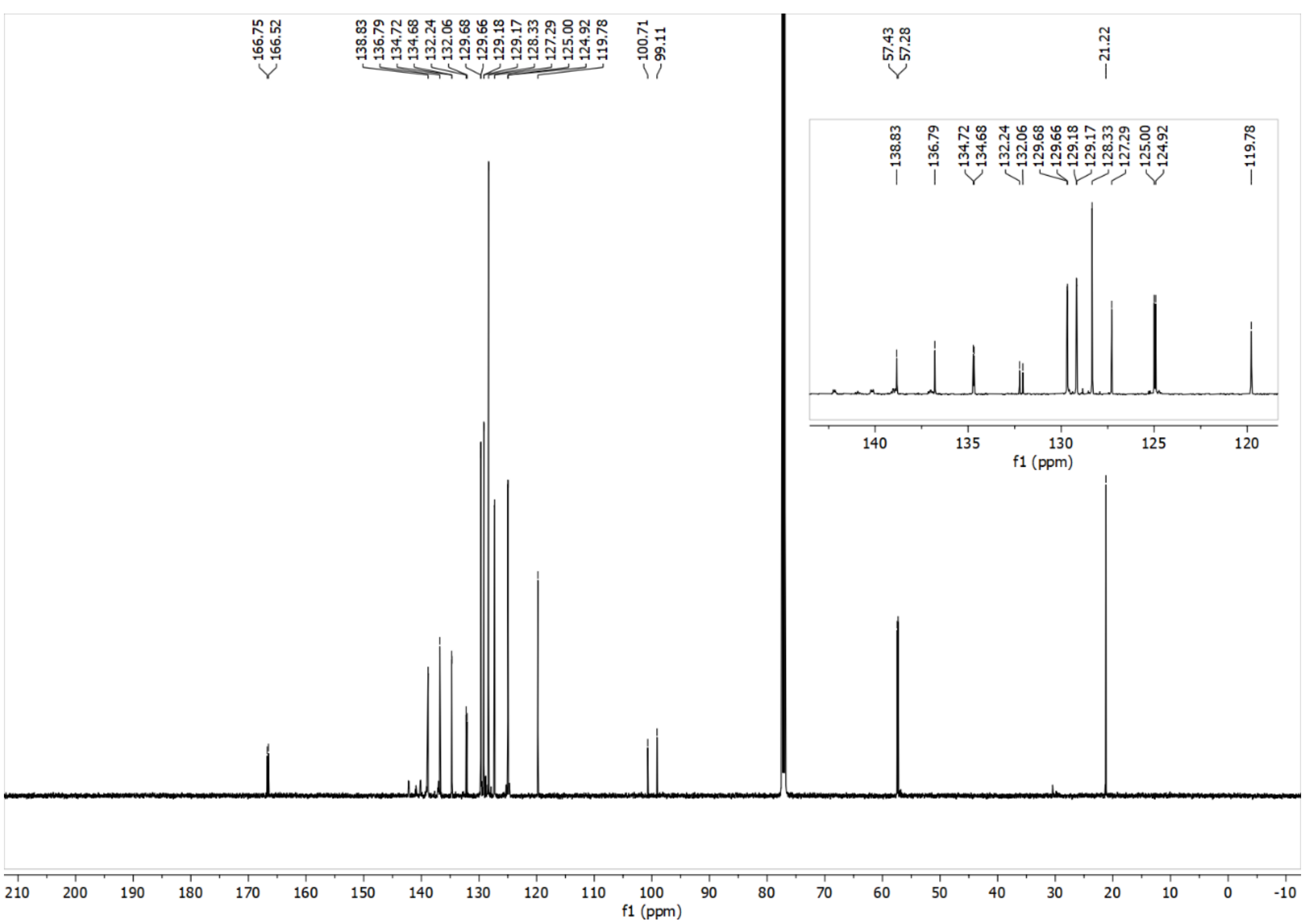

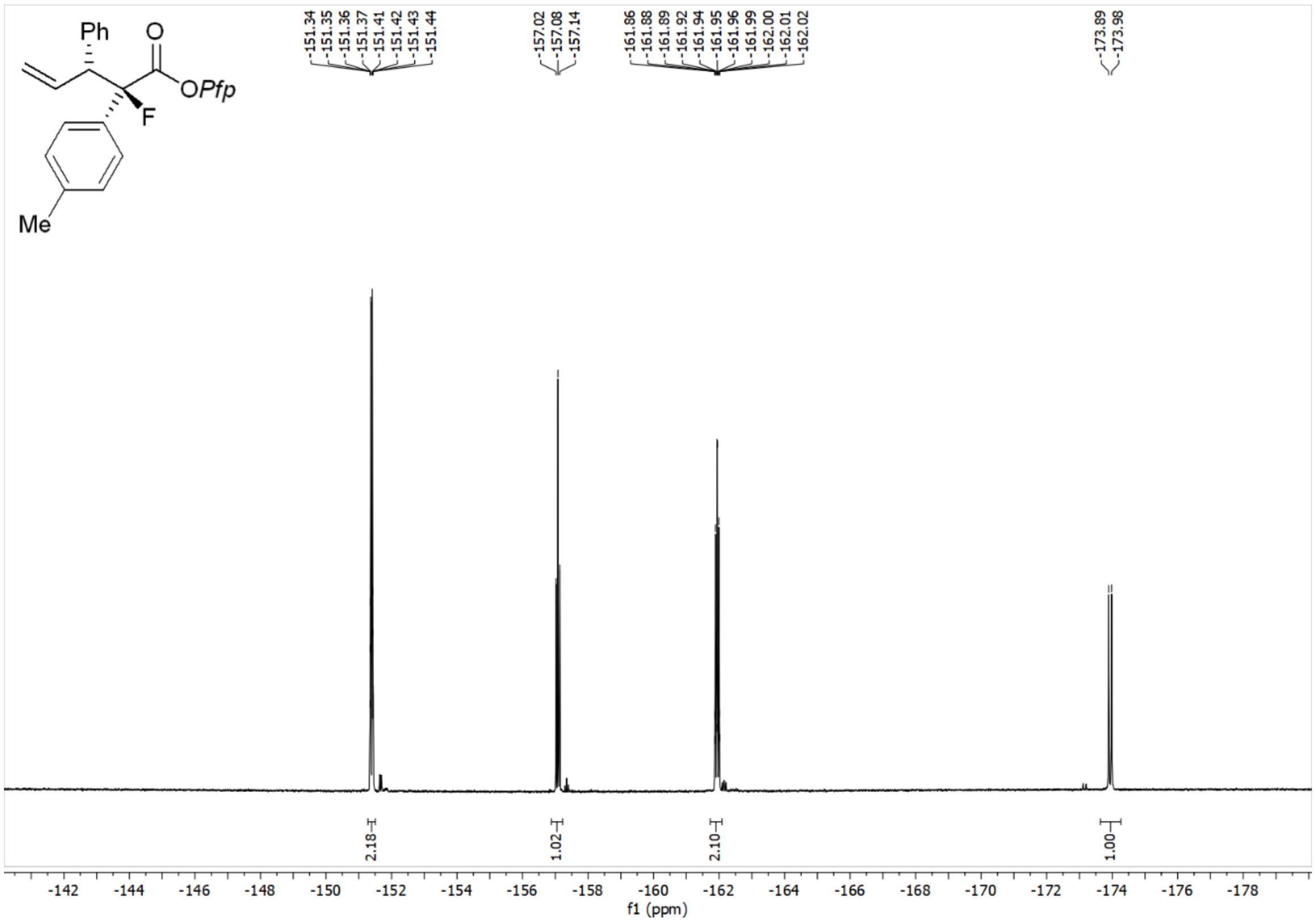

Ph
O
OPfp
F
Me
-151.34
-151.35
-151.36
-151.37
-151.41
-151.42
-151.43
-151.44
-157.02
-157.08
-157.14
-161.86
-161.88
-161.89
-161.92
-161.94
-161.95
-161.96
-161.99
-162.00
-162.01
-162.02
-173.89
-173.98
2.18
1.02
2.10
1.00
-142
-144
-146
-148
-150
-152
-154
-156
-158
-160
-162
-164
-166
-168
-170
-172
-174
-176
-178
f1 (ppm)

**Perfluorophenyl (2*R*,3*S*)-2-fluoro-3-phenyl-2-(p-tolyl)pent-4-enoate (*R*,*S*)-7):**

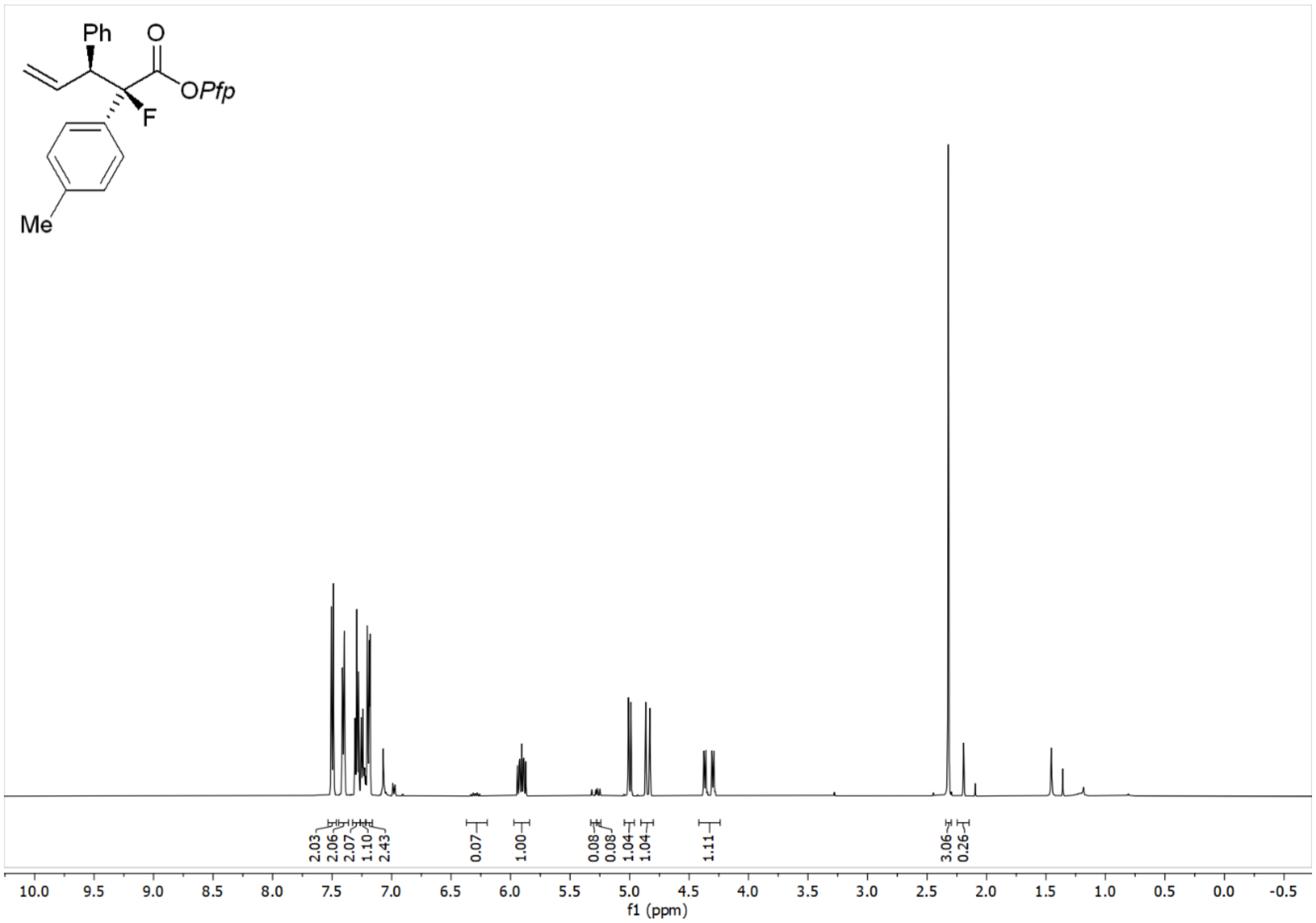


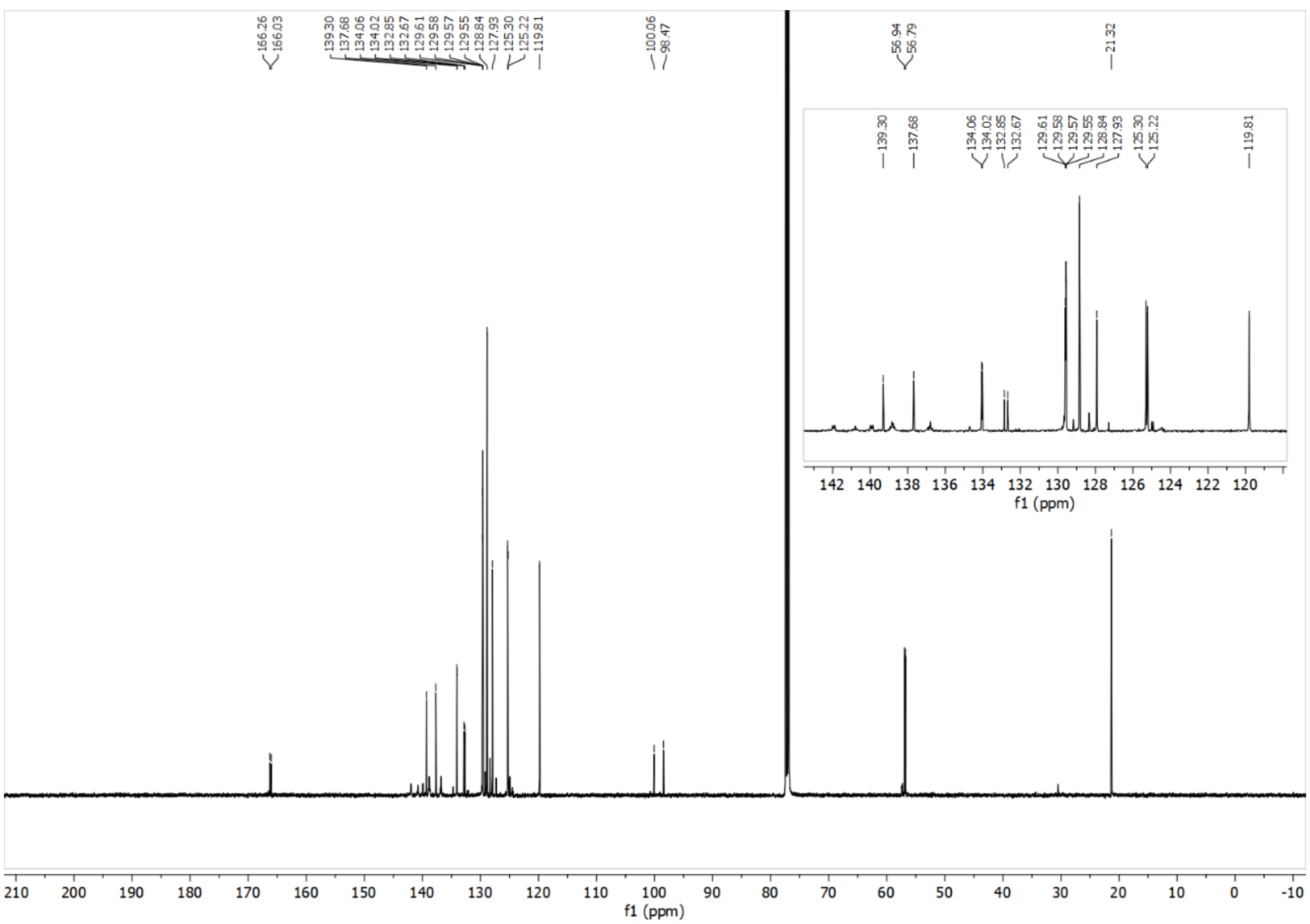

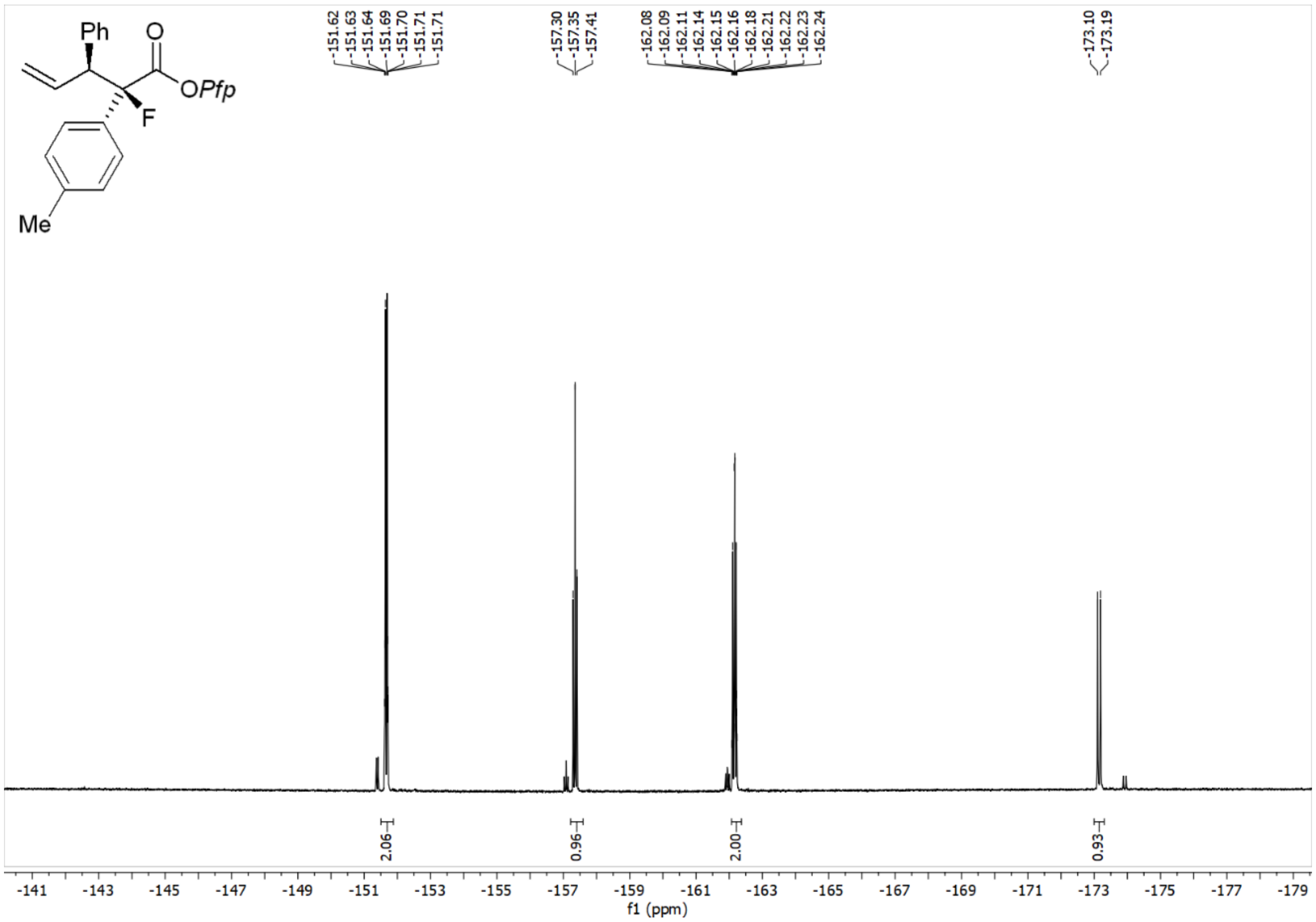
Ph
O
OPfp
F
Me
-151.62
-151.63
-151.64
-151.69
-151.70
-151.71
-151.71
-157.30
-157.35
-157.41
-162.08
-162.09
-162.11
-162.14
-162.15
-162.16
-162.18
-162.21
-162.22
-162.23
-162.24
-173.10
-173.19
2.06
0.96
2.00
0.93
-141
-143
-145
-147
-149
-151
-153
-155
-157
-159
-161
-163
-165
-167
-169
-171
-173
-175
-177
-179
f1 (ppm)

**Perfluorophenyl (2*R*,3*R*)-2-fluoro-2-(4-fluorophenyl)-3-phenylpent-4-enoate (*R*,*R*)-8:**

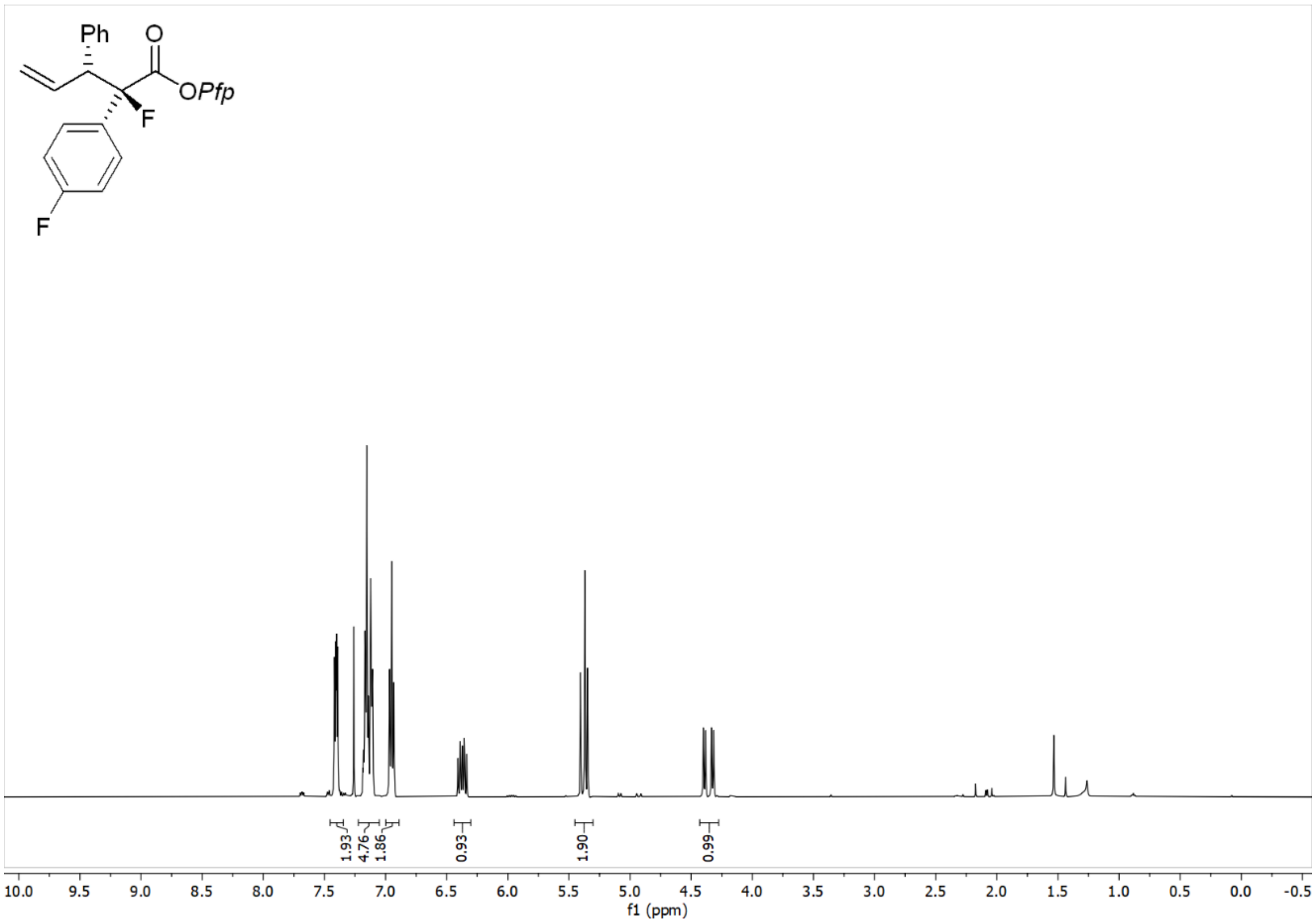


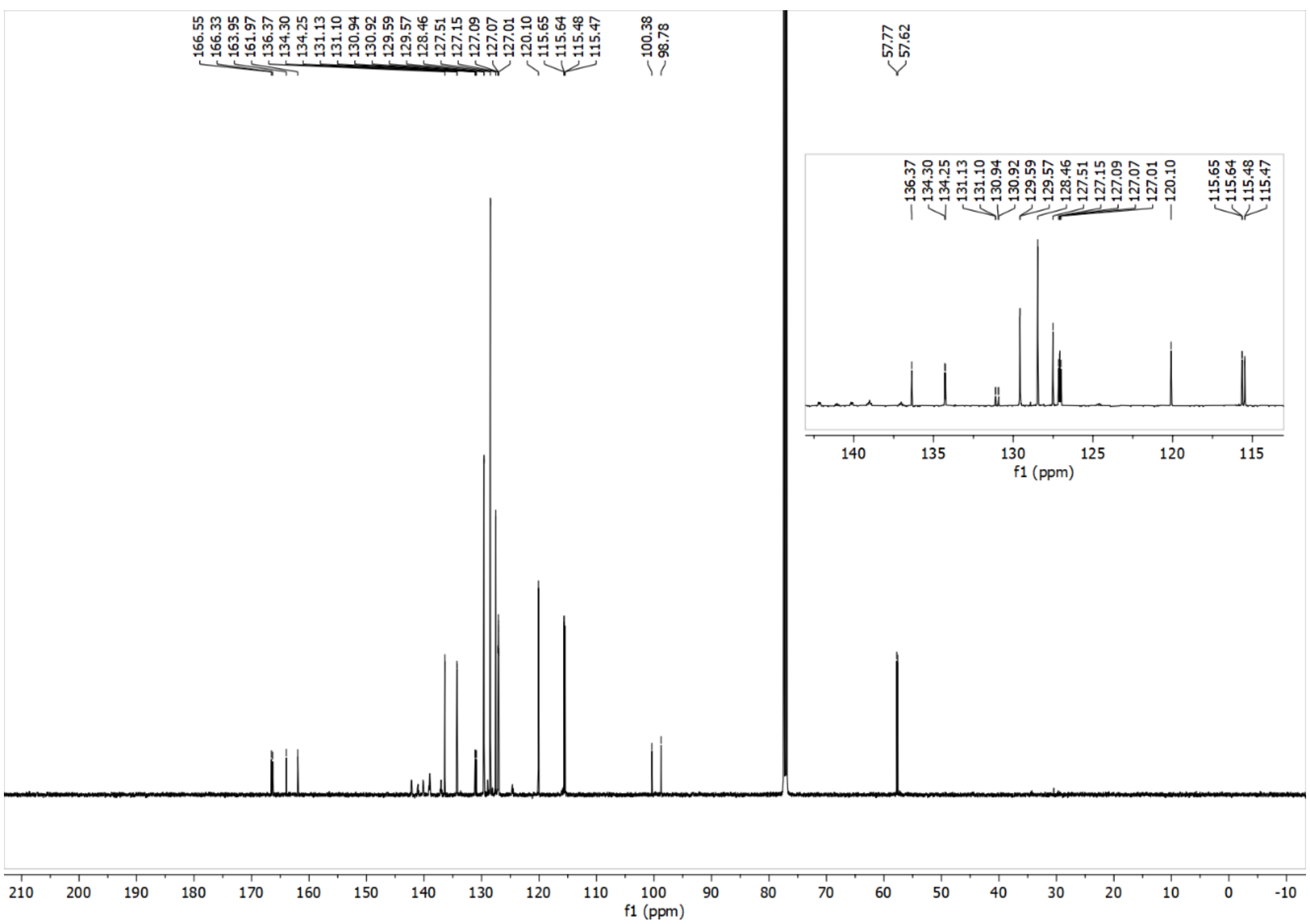

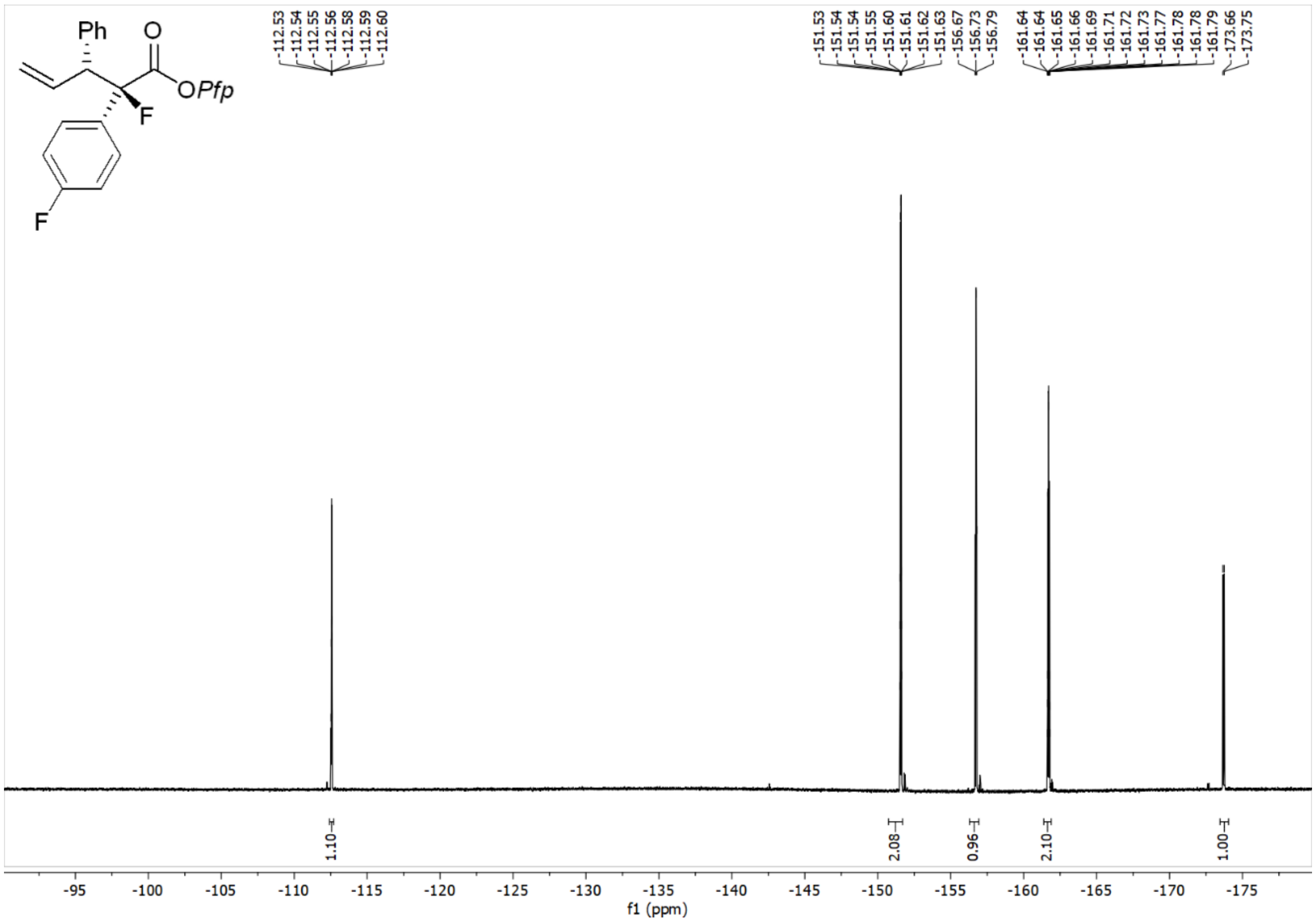
Ph
O
OPfp
F
F
-112.53
-112.54
-112.55
-112.56
-112.58
-112.59
-112.60
-151.53
-151.54
-151.54
-151.55
-151.60
-151.61
-151.62
-151.63
-156.67
-156.73
-156.79
-161.64
-161.64
-161.65
-161.66
-161.69
-161.71
-161.72
-161.73
-161.77
-161.78
-161.79
-173.66
-173.75
1.10
2.08
0.96
2.10
1.00
-95
-100
-105
-110
-115
-120
-125
-130
-135
-140
-145
-150
-155
-160
-165
-170
-175
f1 (ppm)

**Perfluorophenyl (2*R*,3*S*)-2-fluoro-2-(4-fluorophenyl)-3-phenylpent-4-enoate (*R*,*S*)-8:**

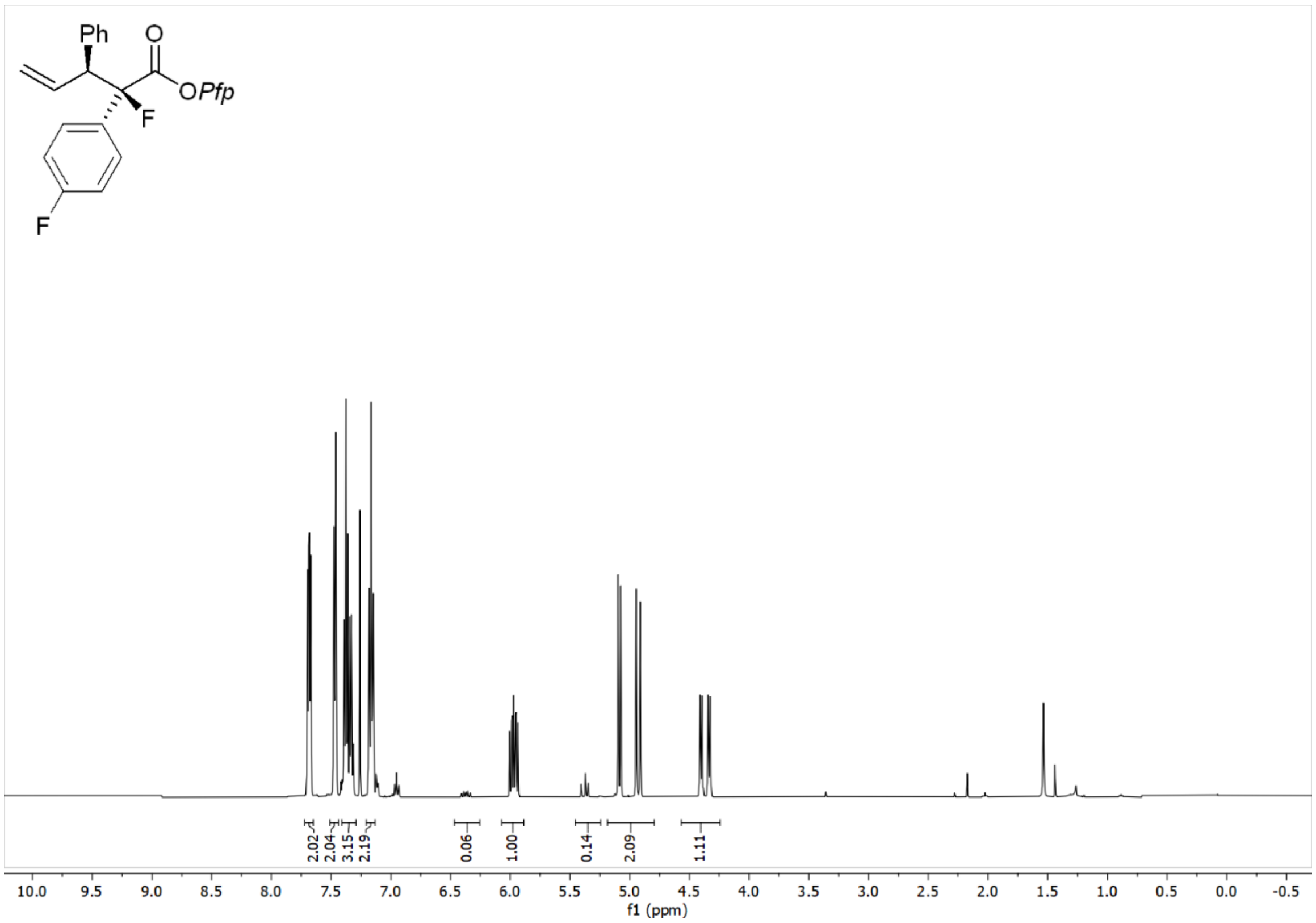


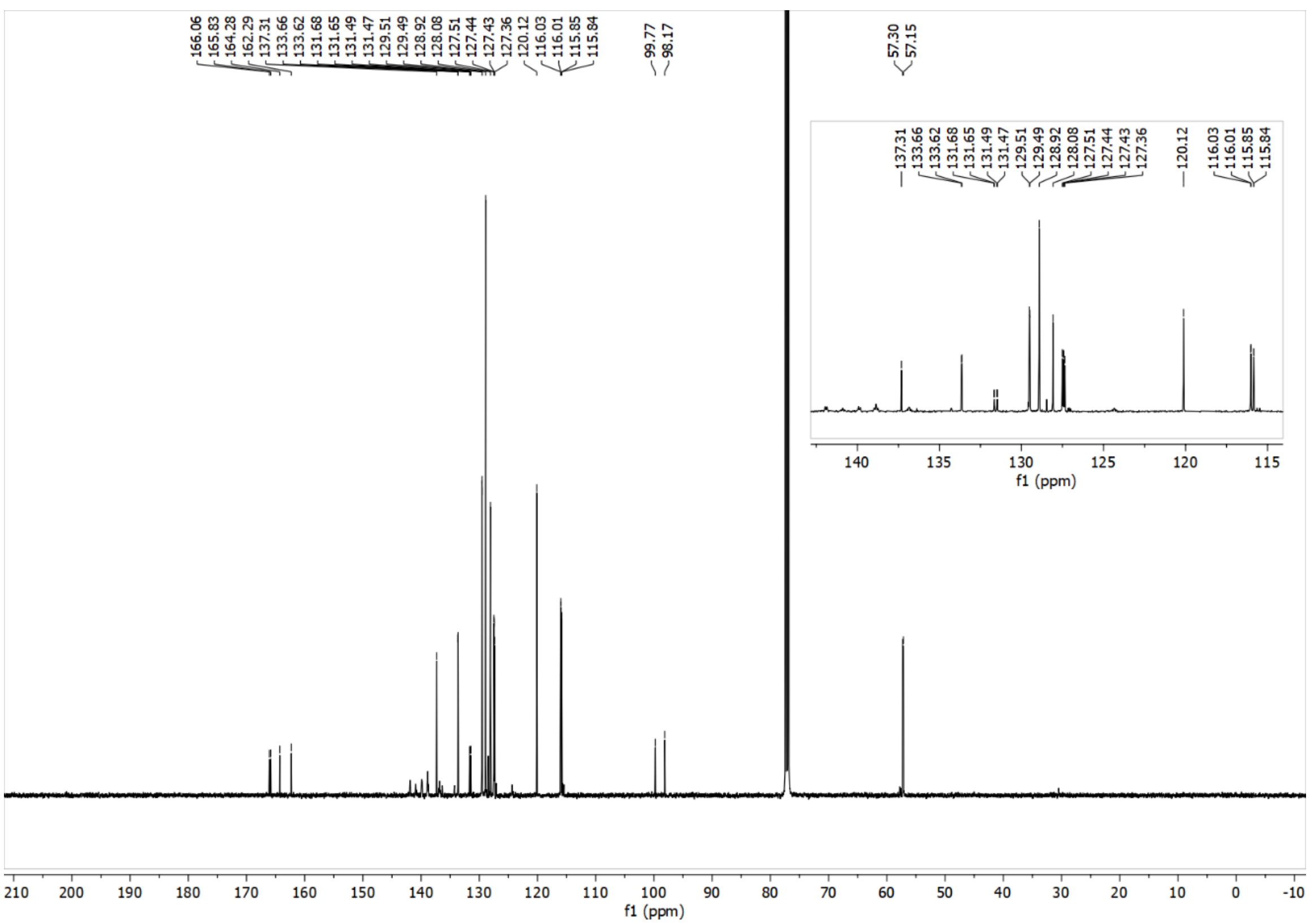

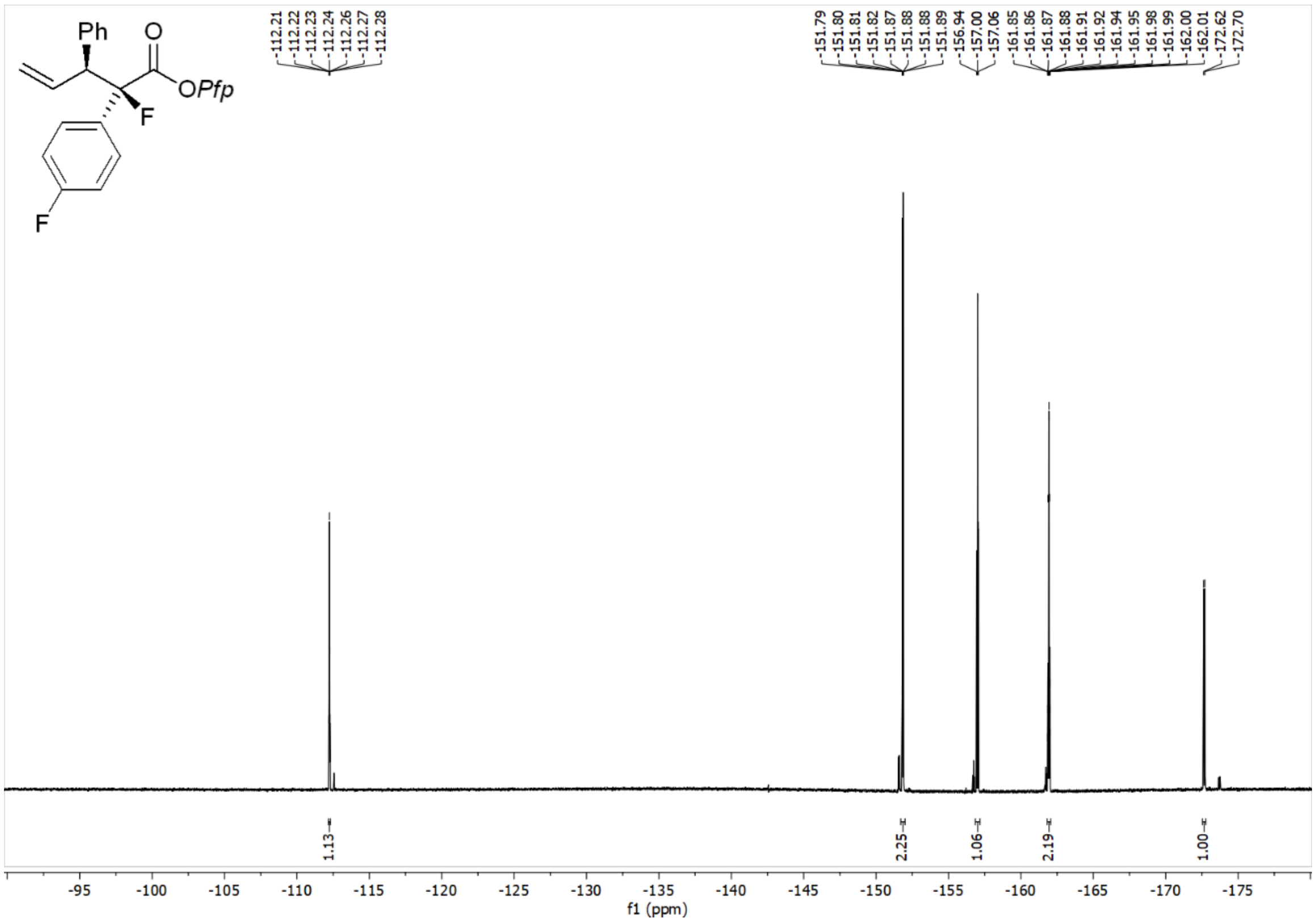

**Perfluorophenyl (2*R*,3*R*)-2-(4-chlorophenyl)-2-fluoro-3-phenylpent-4-enoate (*R*,*R*)-10:**

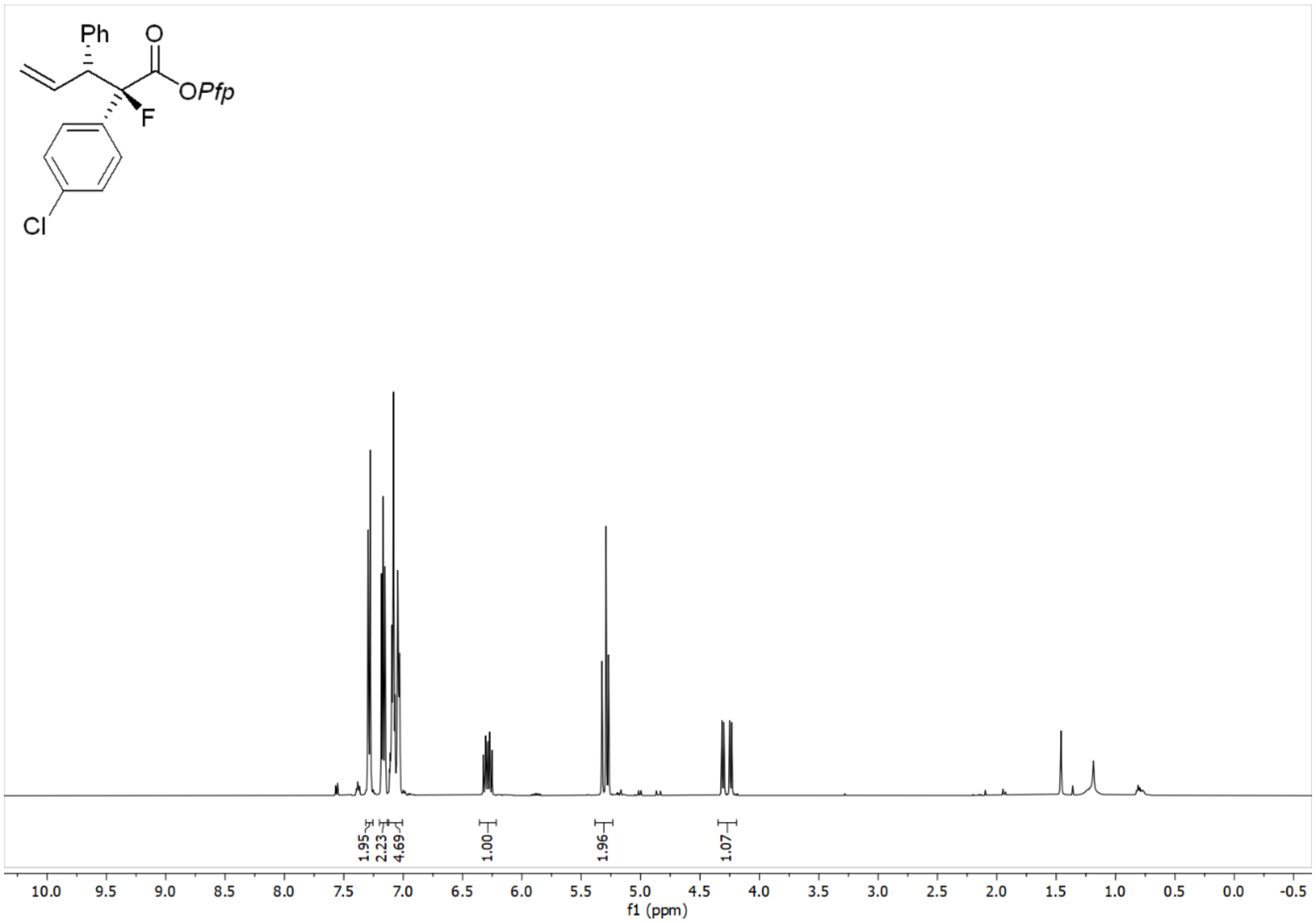


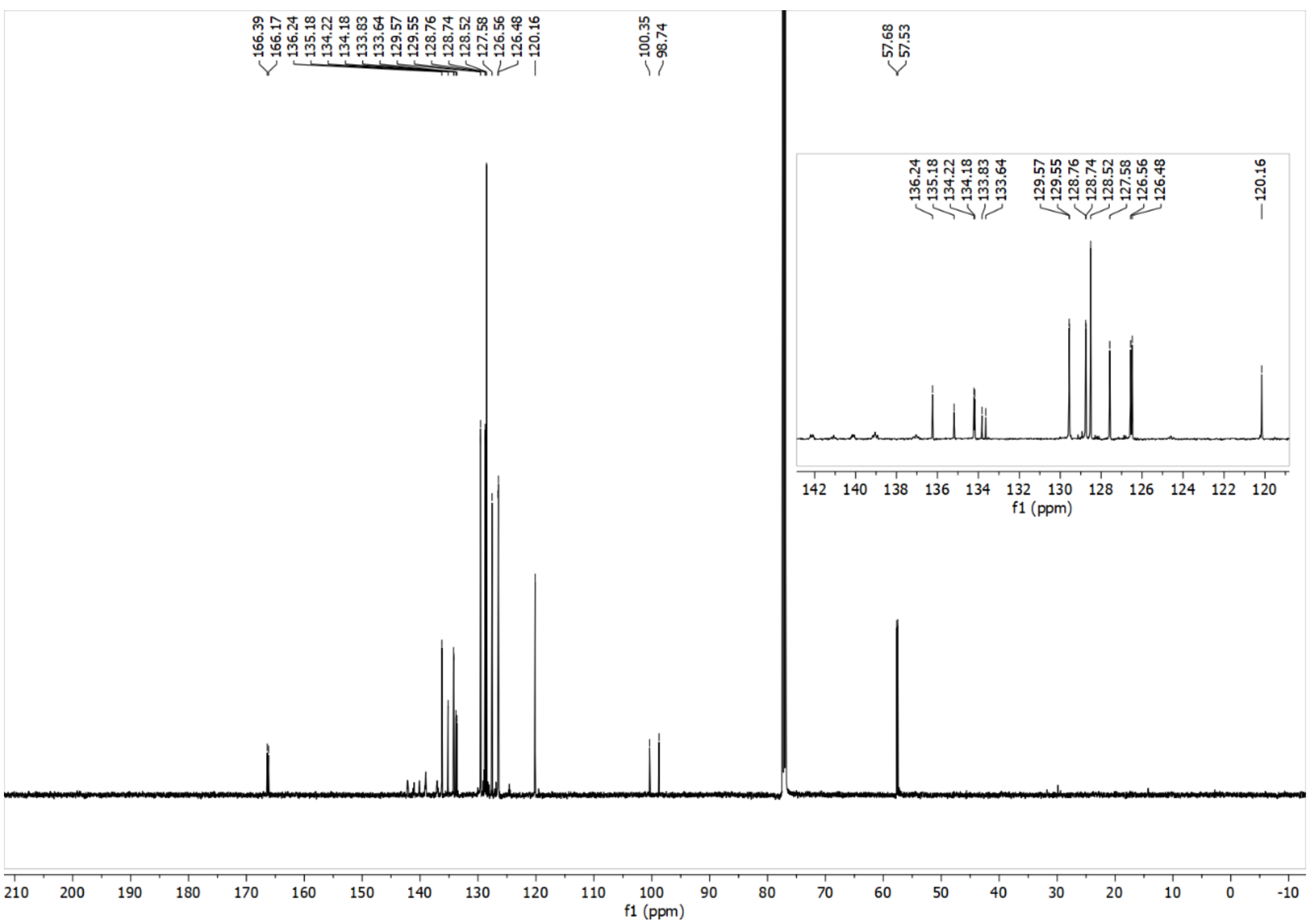

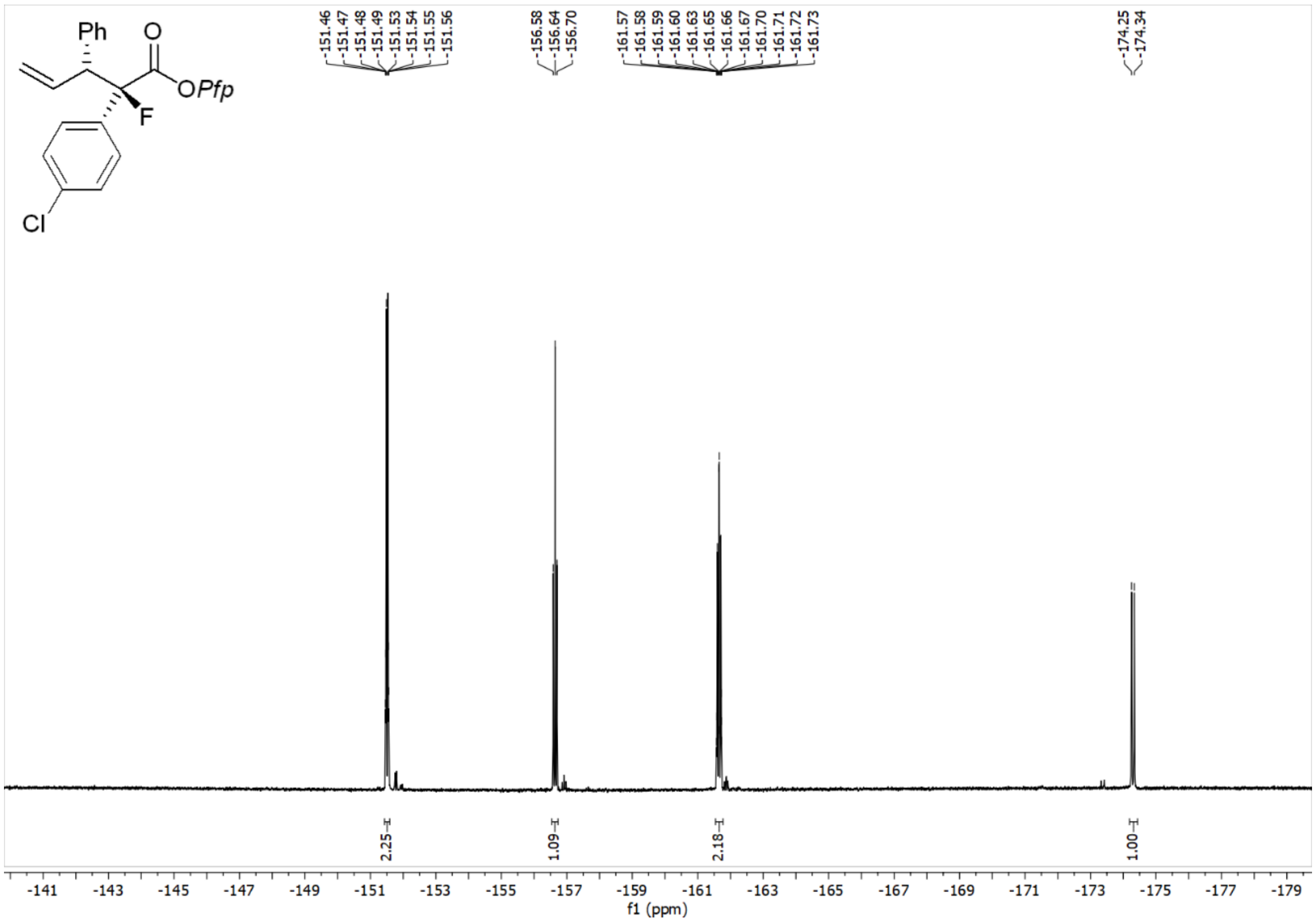

Ph
O
OPfp
F
Cl
-151.46
-151.47
-151.48
-151.49
-151.53
-151.54
-151.55
-151.56
-156.58
-156.64
-156.70
-161.57
-161.58
-161.59
-161.60
-161.63
-161.65
-161.66
-161.67
-161.70
-161.71
-161.72
-161.73
-174.25
-174.34
2.25
1.09
2.18
1.00
-141 -143 -145 -147 -149 -151 -153 -155 -157 -159 -161 -163 -165 -167 -169 -171 -173 -175 -177 -179
f1 (ppm)

**Perfluorophenyl (2*R*,3*S*)-2-(4-chlorophenyl)-2-fluoro-3-phenylpent-4-enoate (*R*,*S*)-10:**

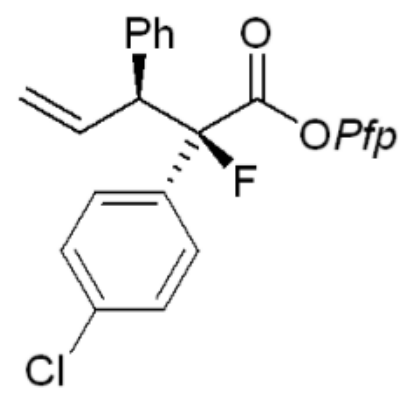


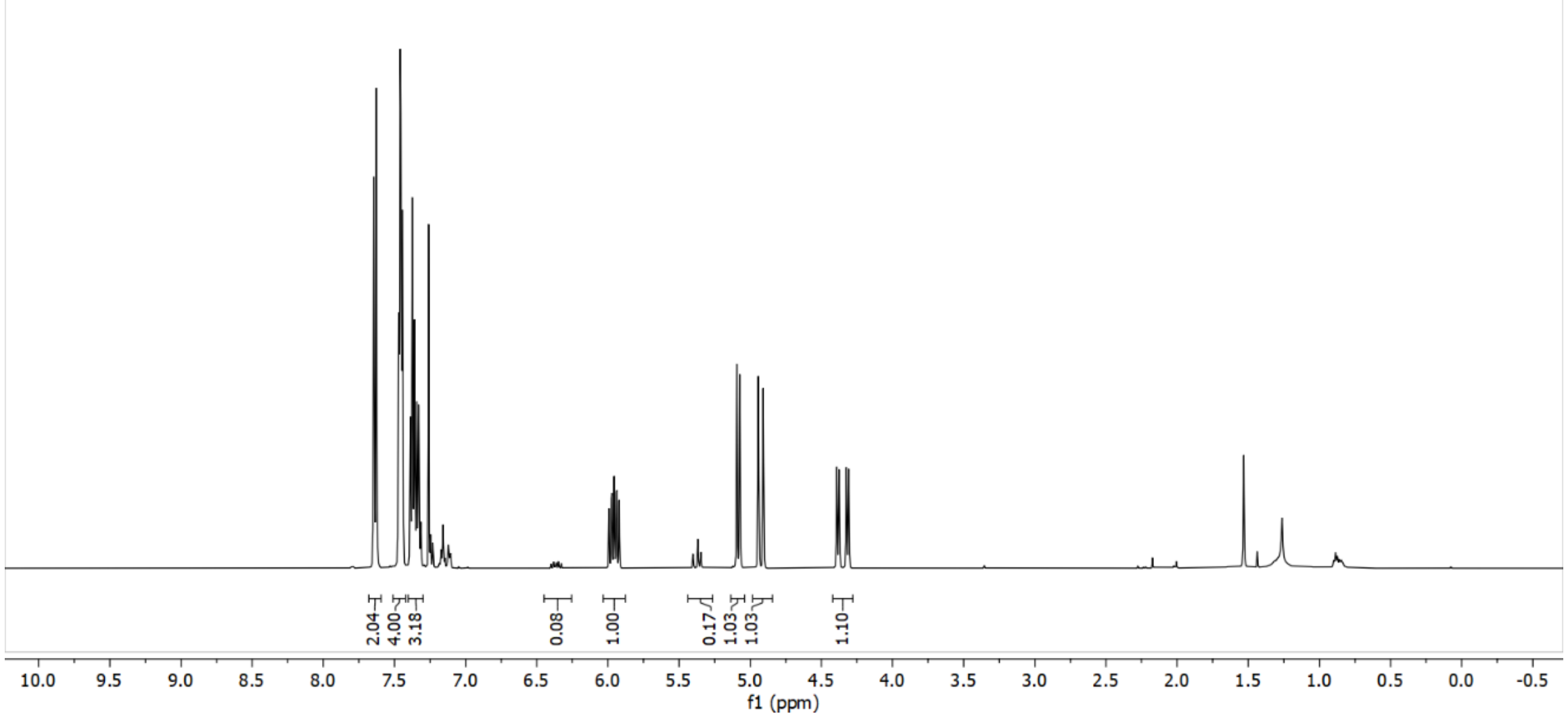


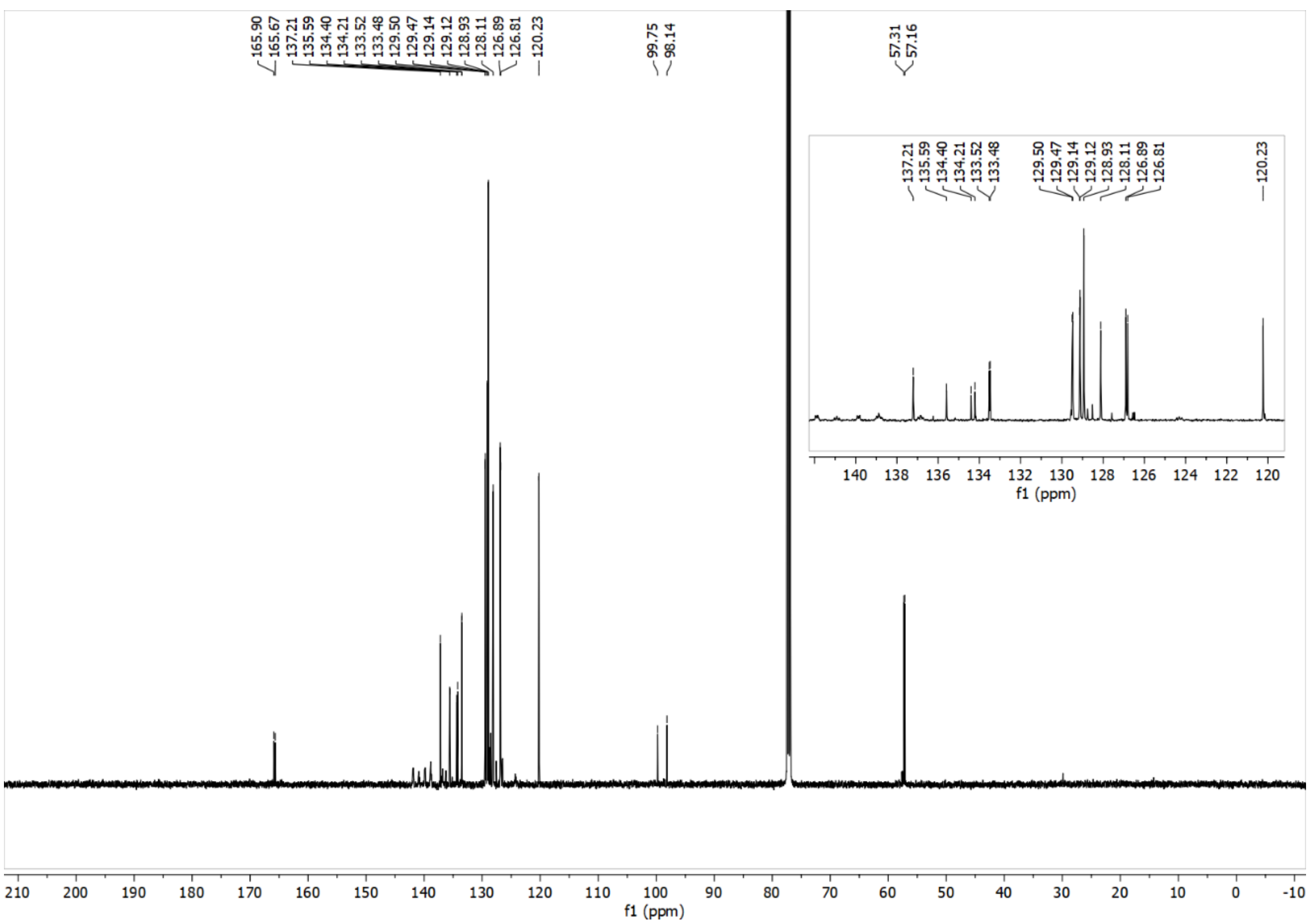

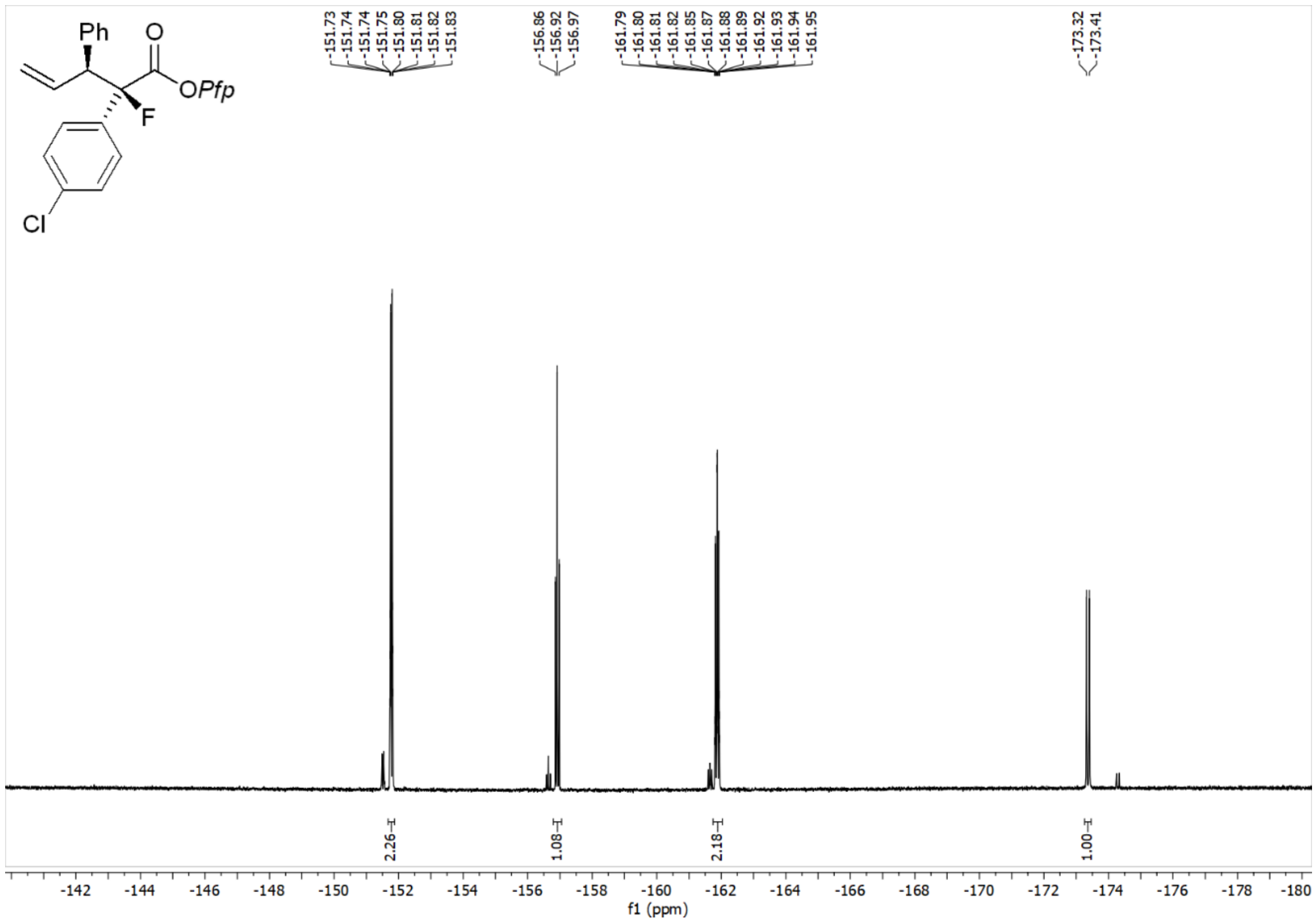
Ph
O
OPfp
F
Cl
-151.73
-151.74
-151.74
-151.75
-151.80
-151.81
-151.82
-151.83
-156.86
-156.92
-156.97
-161.79
-161.80
-161.81
-161.82
-161.85
-161.87
-161.88
-161.89
-161.92
-161.93
-161.94
-161.95
-173.32
-173.41
2.26
1.08
2.18
1.00
-142 -144 -146 -148 -150 -152 -154 -156 -158 -160 -162 -164 -166 -168 -170 -172 -174 -176 -178 -180
f1 (ppm)

**Perfluorophenyl (2*R*,3*R*)-2-(4-bromophenyl)-2-fluoro-3-phenylpent-4-enoate (*R*,*R*)-11):**

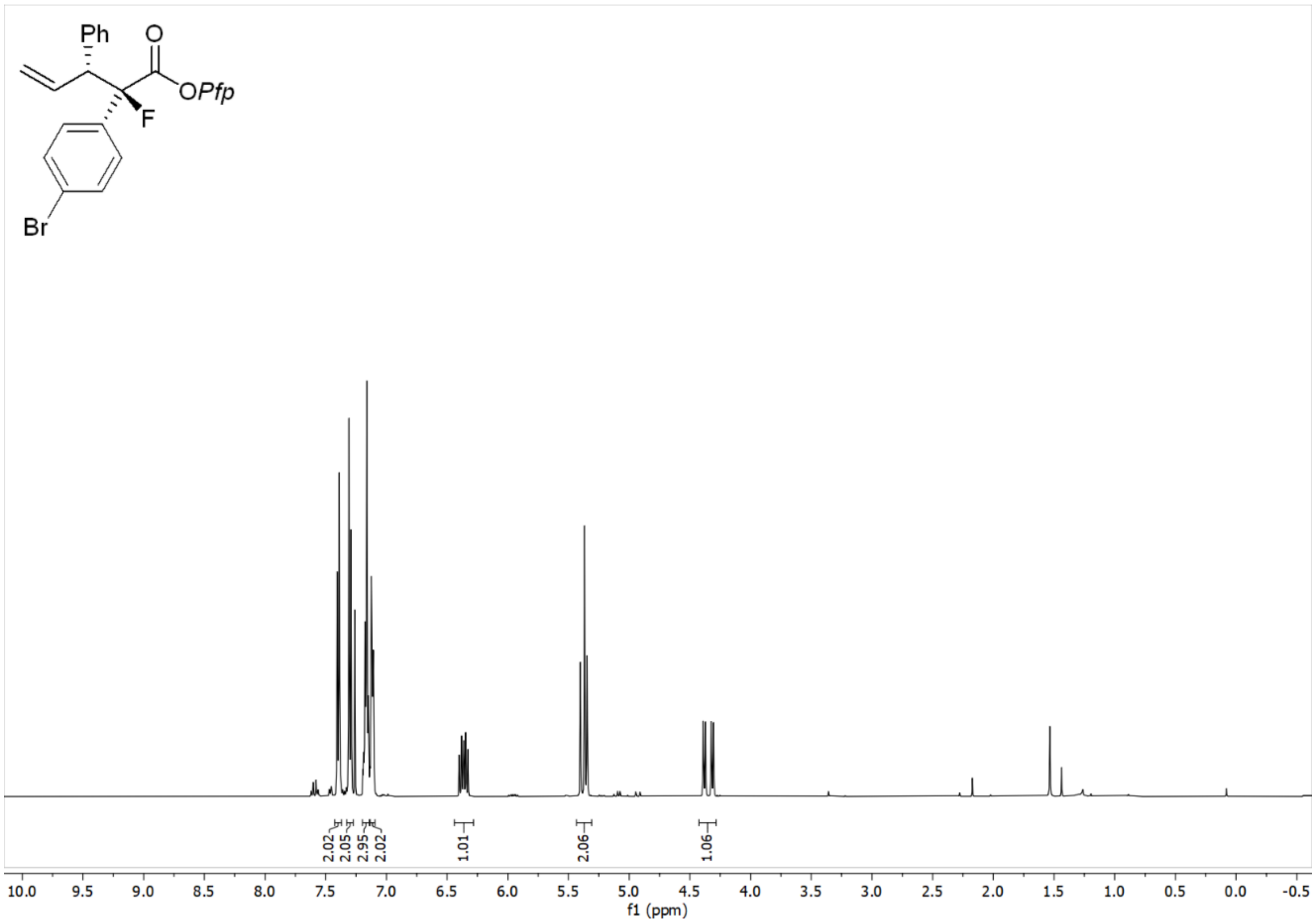


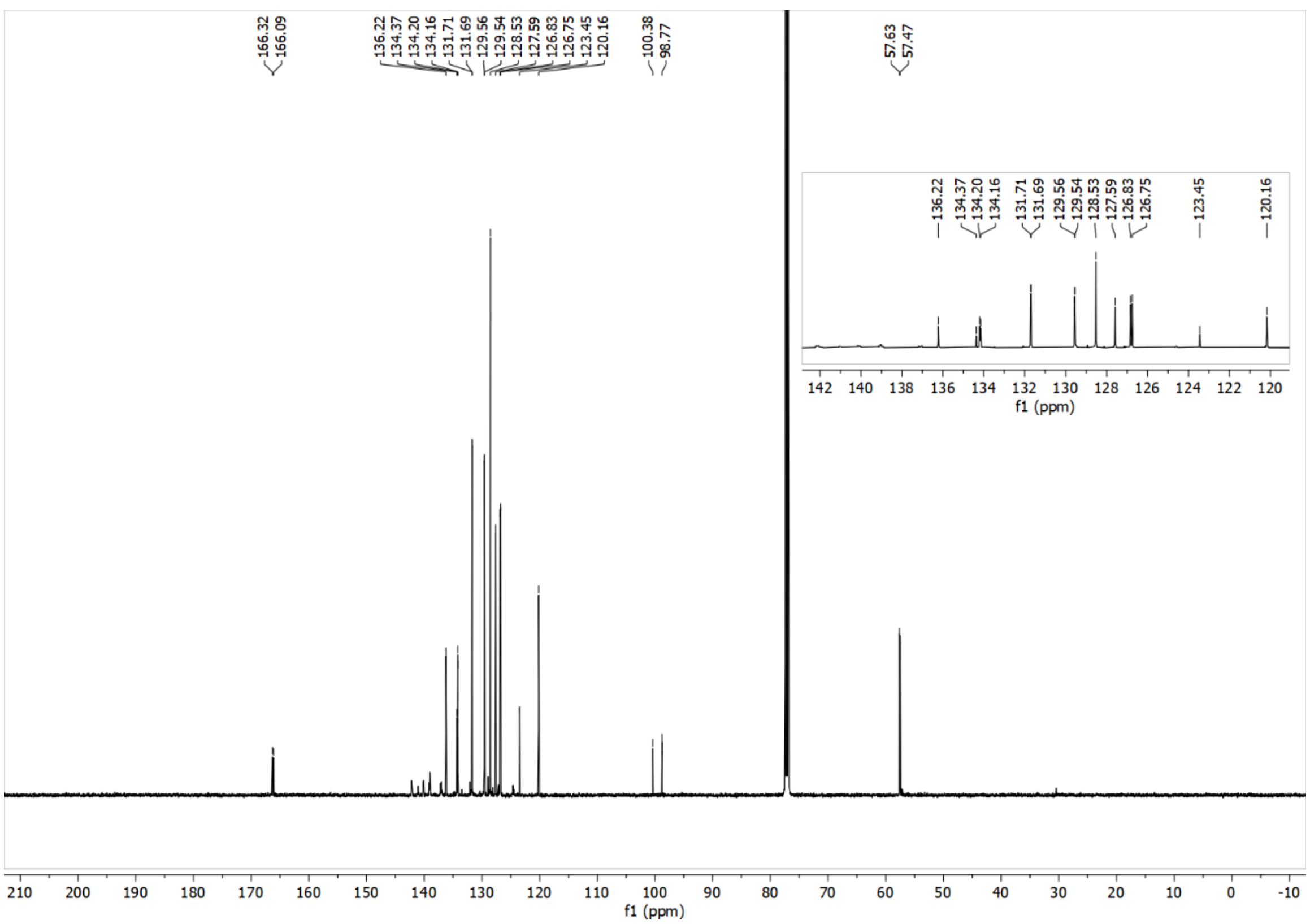

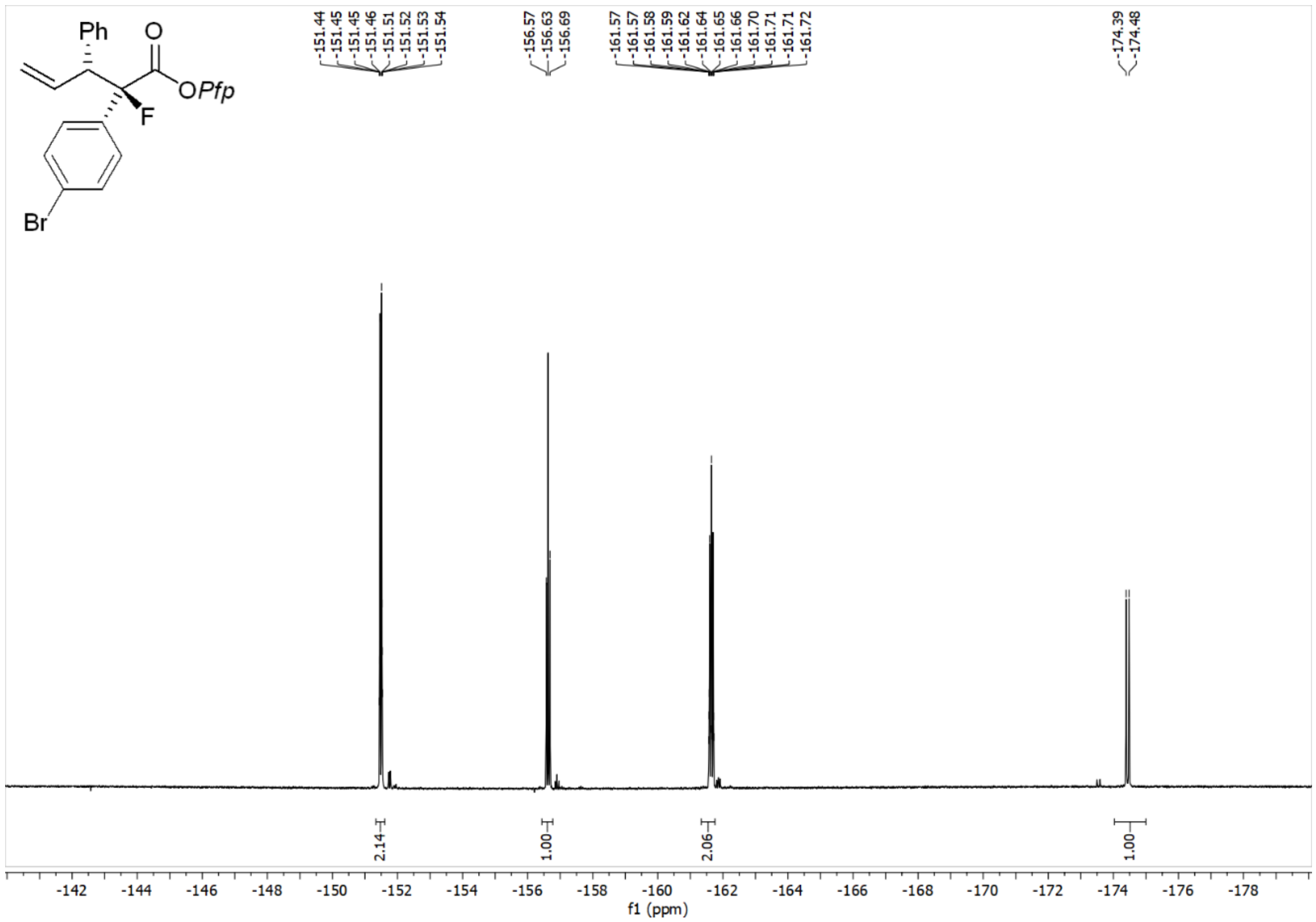
Ph
O
OPfp
F
Br
-151.44
-151.45
-151.45
-151.46
-151.51
-151.52
-151.53
-151.54
-156.57
-156.63
-156.69
-161.57
-161.57
-161.58
-161.59
-161.62
-161.64
-161.65
-161.66
-161.70
-161.71
-161.71
-161.72
-174.39
-174.48
2.14
1.00
2.06
1.00
-142
-144
-146
-148
-150
-152
-154
-156
-158
-160
-162
-164
-166
-168
-170
-172
-174
-176
-178
f1 (ppm)

**Perfluorophenyl (2*R*,3*S*)-2-(4-bromophenyl)-2-fluoro-3-phenylpent-4-enoate (*R*,*S*)-11:**

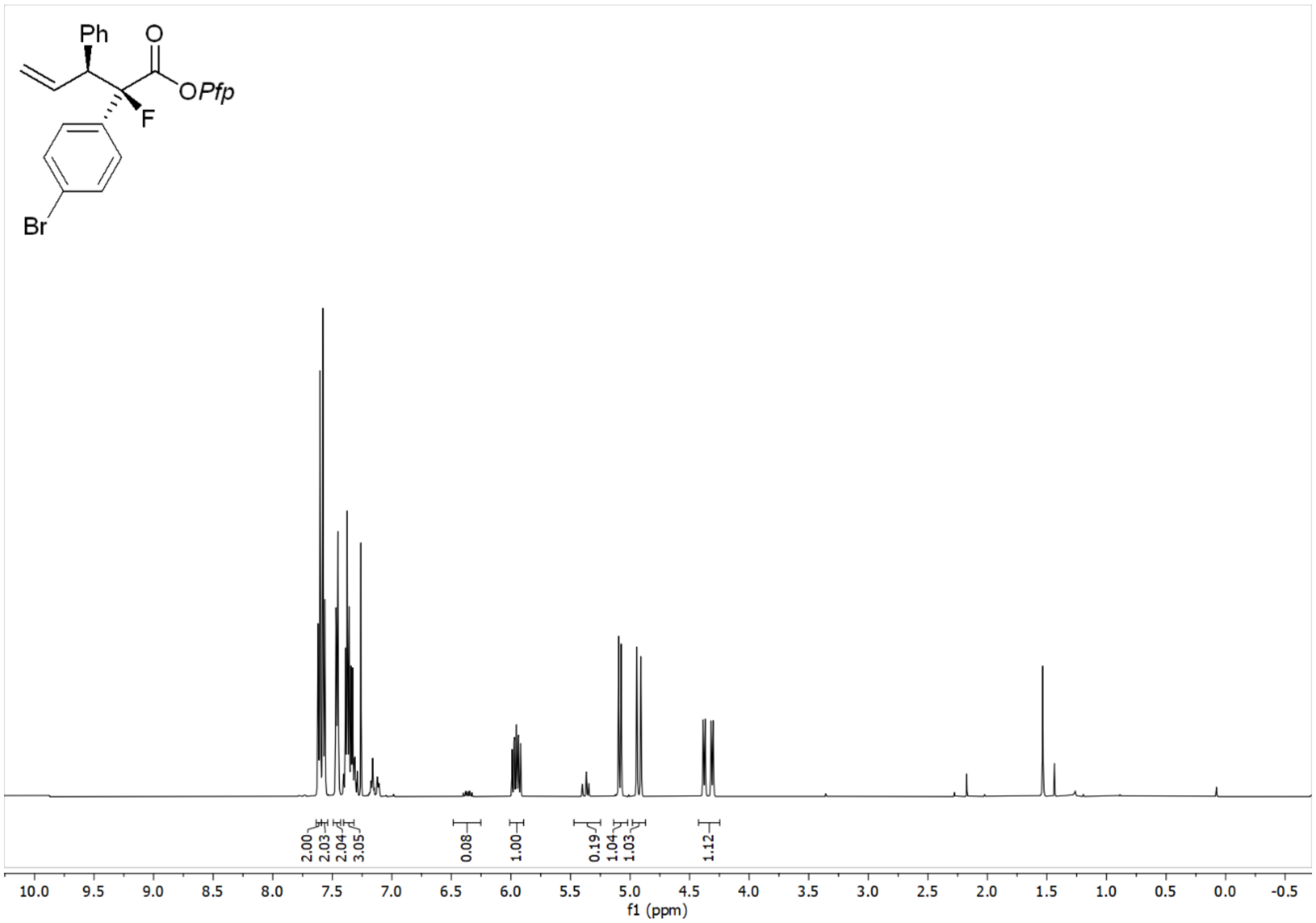


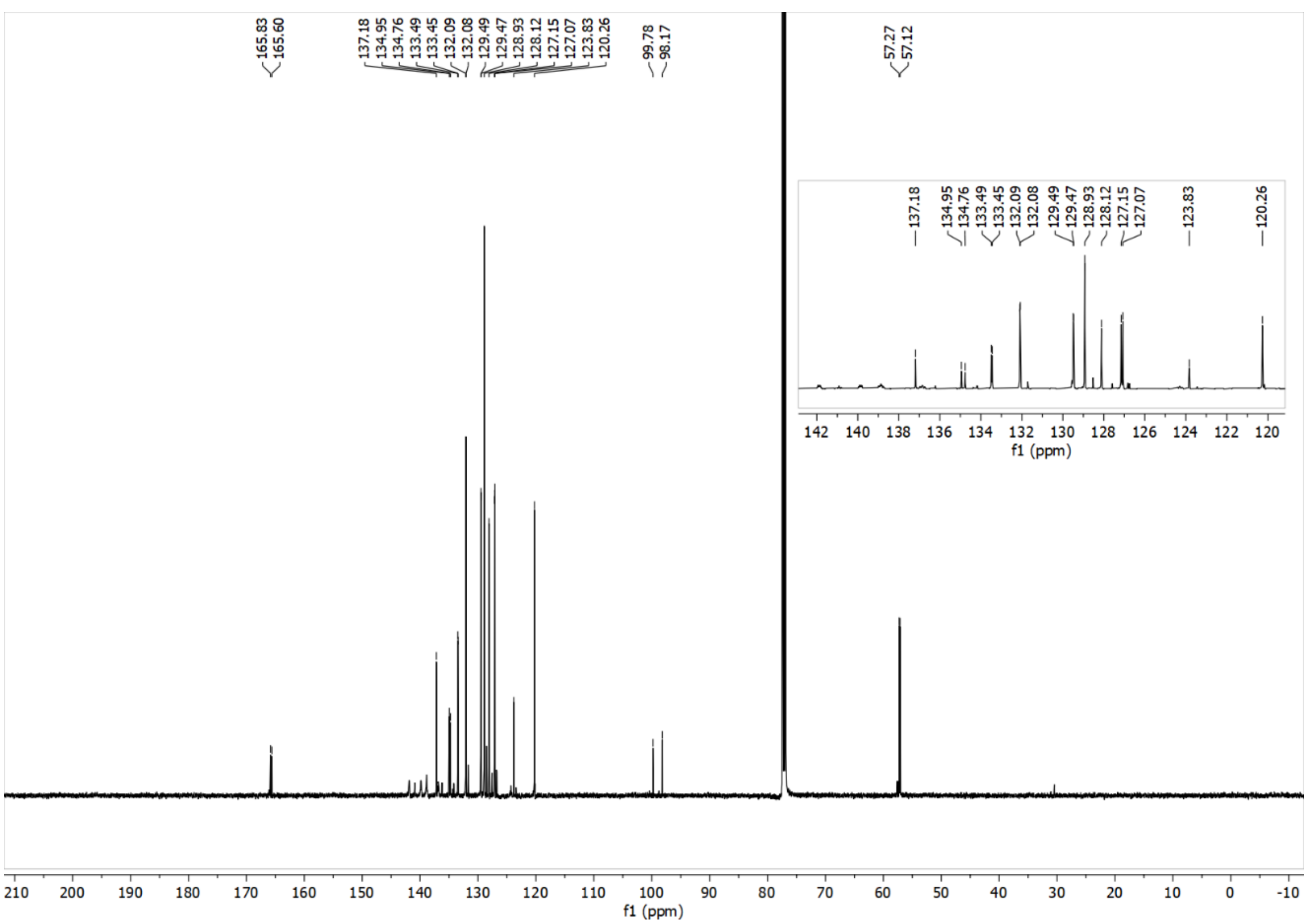

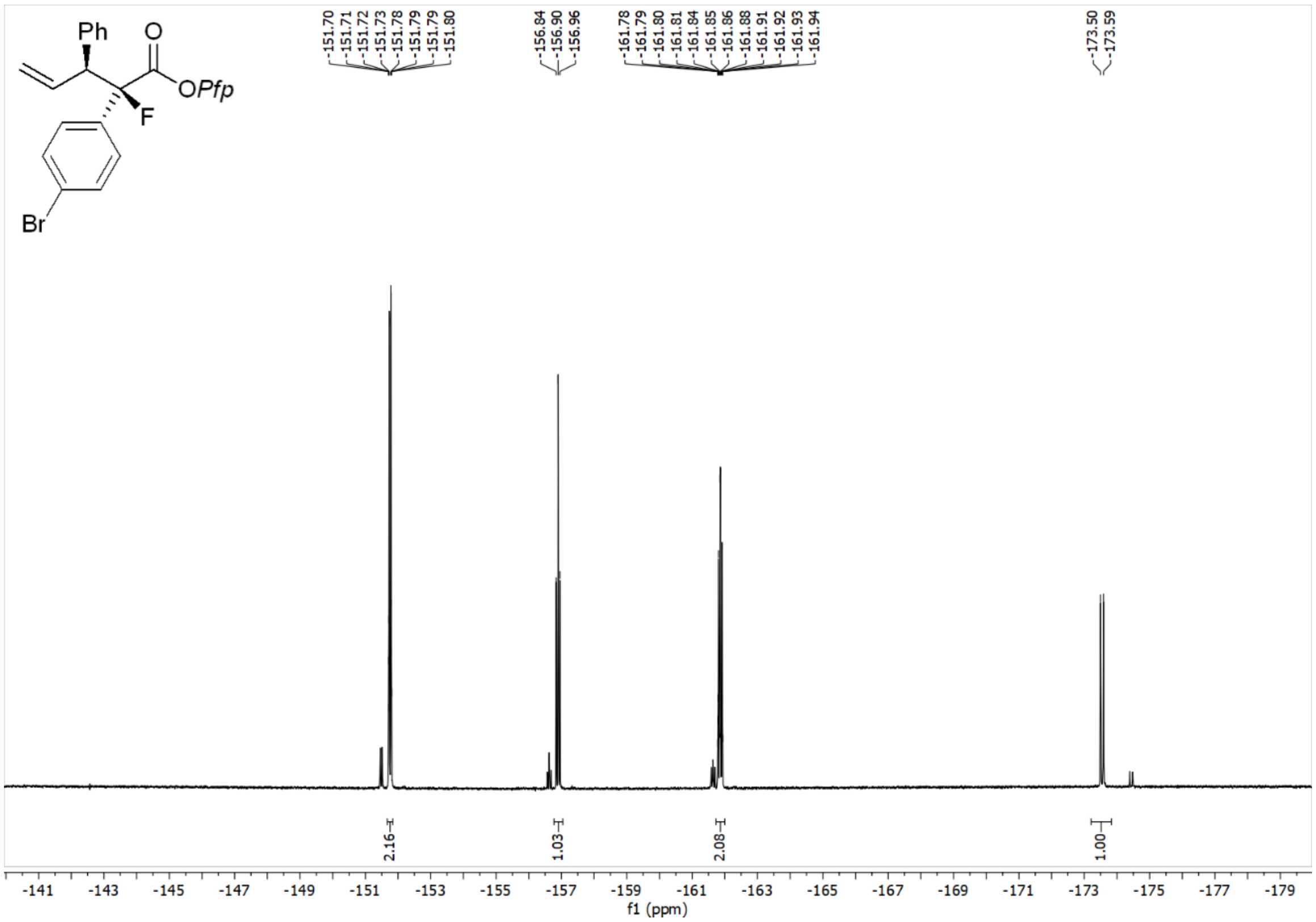
Ph
O
OPfp
F
Br
-151.70
-151.71
-151.72
-151.73
-151.78
-151.79
-151.79
-151.80
-156.84
-156.90
-156.96
-161.78
-161.79
-161.80
-161.81
-161.84
-161.85
-161.86
-161.88
-161.91
-161.92
-161.93
-161.94
-173.50
-173.59
2.16
1.03
2.08
1.00
-141
-143
-145
-147
-149
-151
-153
-155
-157
-159
-161
-163
-165
-167
-169
-171
-173
-175
-177
-179
f1 (ppm)

**Perfluorophenyl (2*R*,3*R*)-2-fluoro-2-(4-iodophenyl)-3-phenylpent-4-enoate (*R*,*R*)-12:**

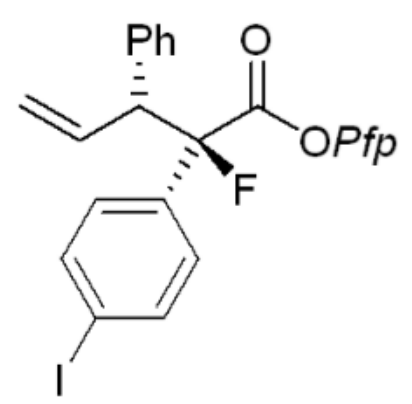


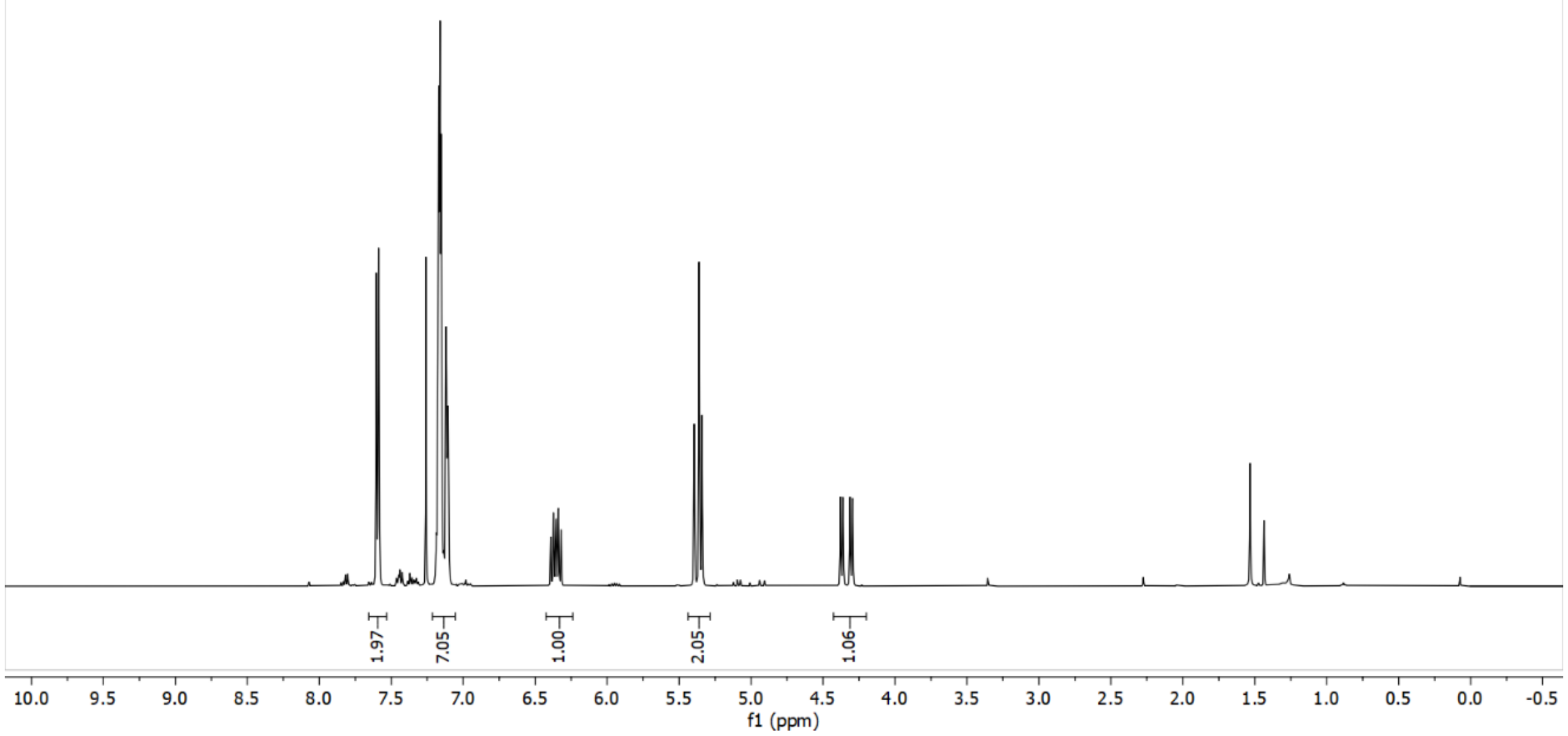


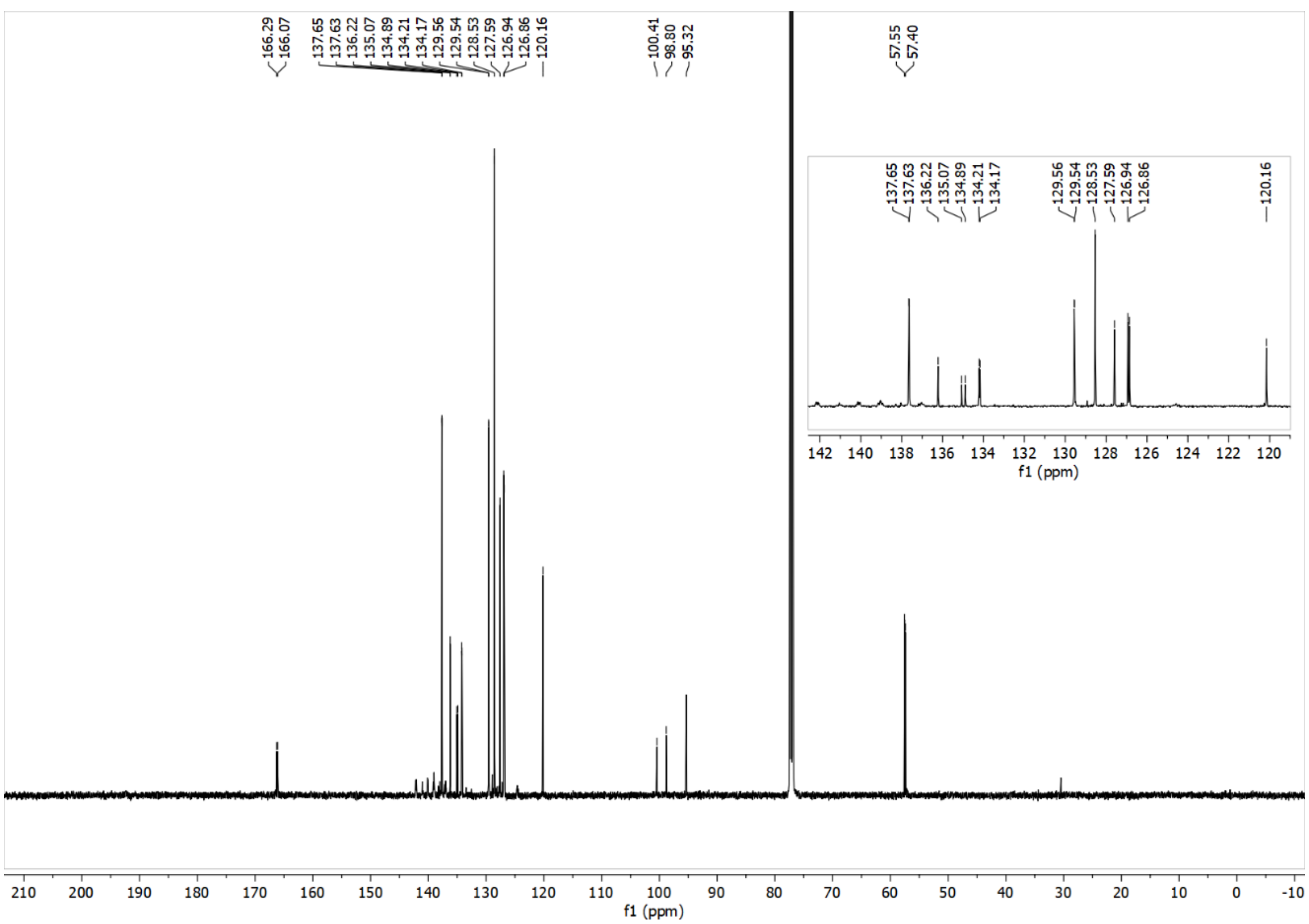

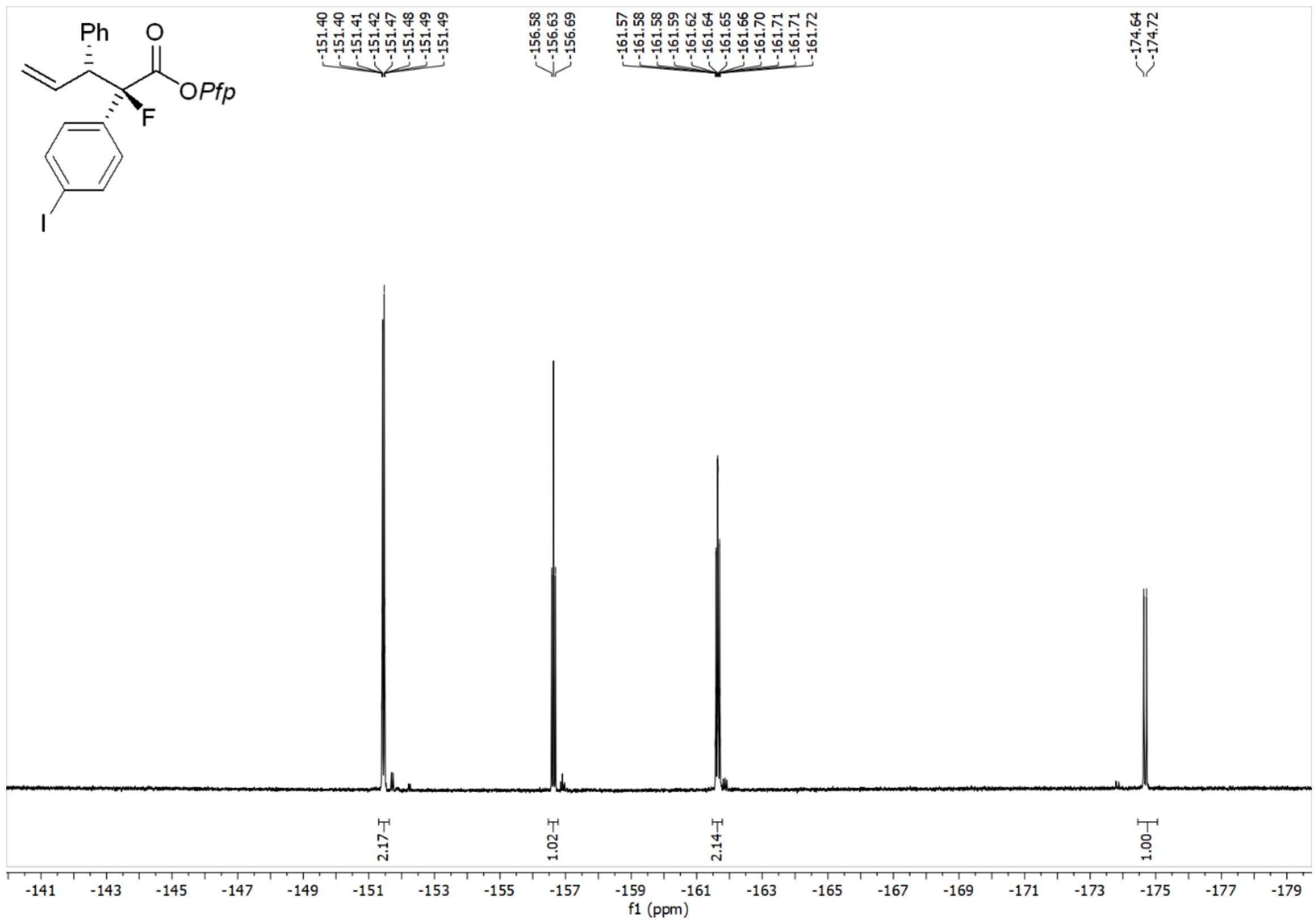
Ph
O
OPfp
F
I
-151.40
-151.40
-151.41
-151.42
-151.47
-151.48
-151.49
-151.49
-156.58
-156.63
-156.69
-161.57
-161.58
-161.58
-161.59
-161.62
-161.64
-161.65
-161.66
-161.70
-161.71
-161.71
-161.72
-174.64
-174.72
2.17
1.02
2.14
1.00
-141
-143
-145
-147
-149
-151
-153
-155
-157
-159
-161
-163
-165
-167
-169
-171
-173
-175
-177
-179
f1 (ppm)

**Perfluorophenyl (2*R*,3*S*)-2-fluoro-2-(4-iodophenyl)-3-phenylpent-4-enoate (*R*,*S*)-12:**

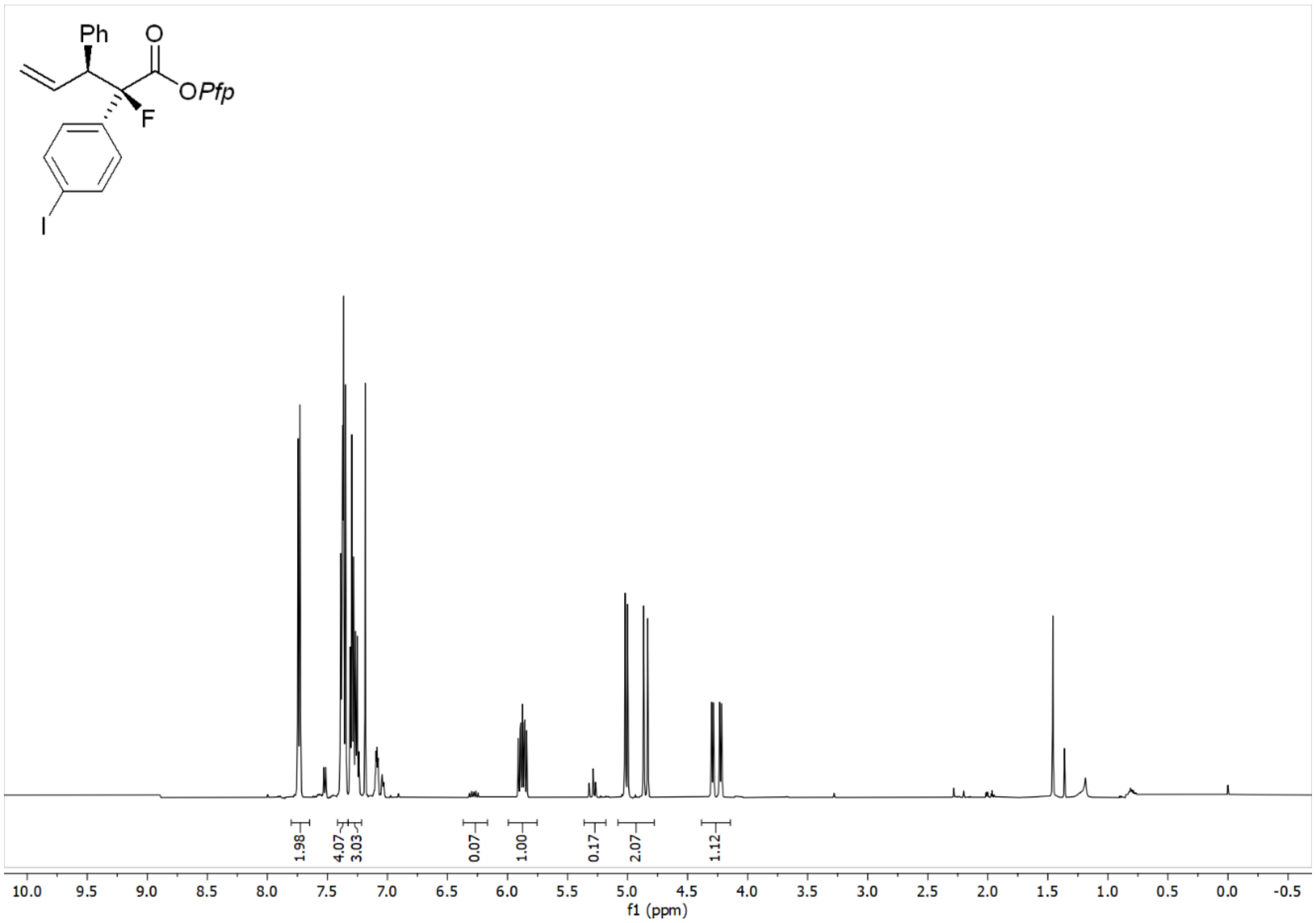


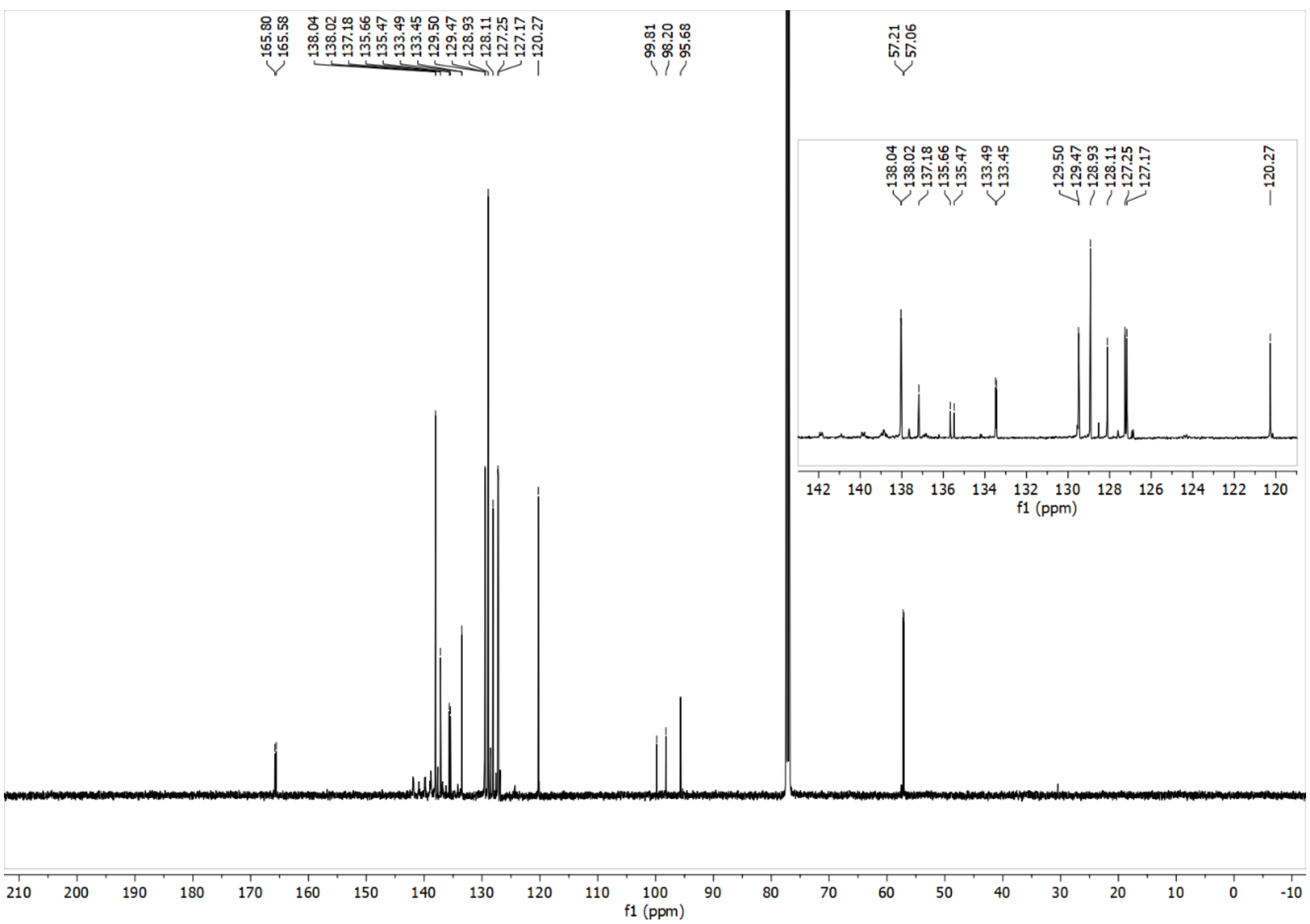

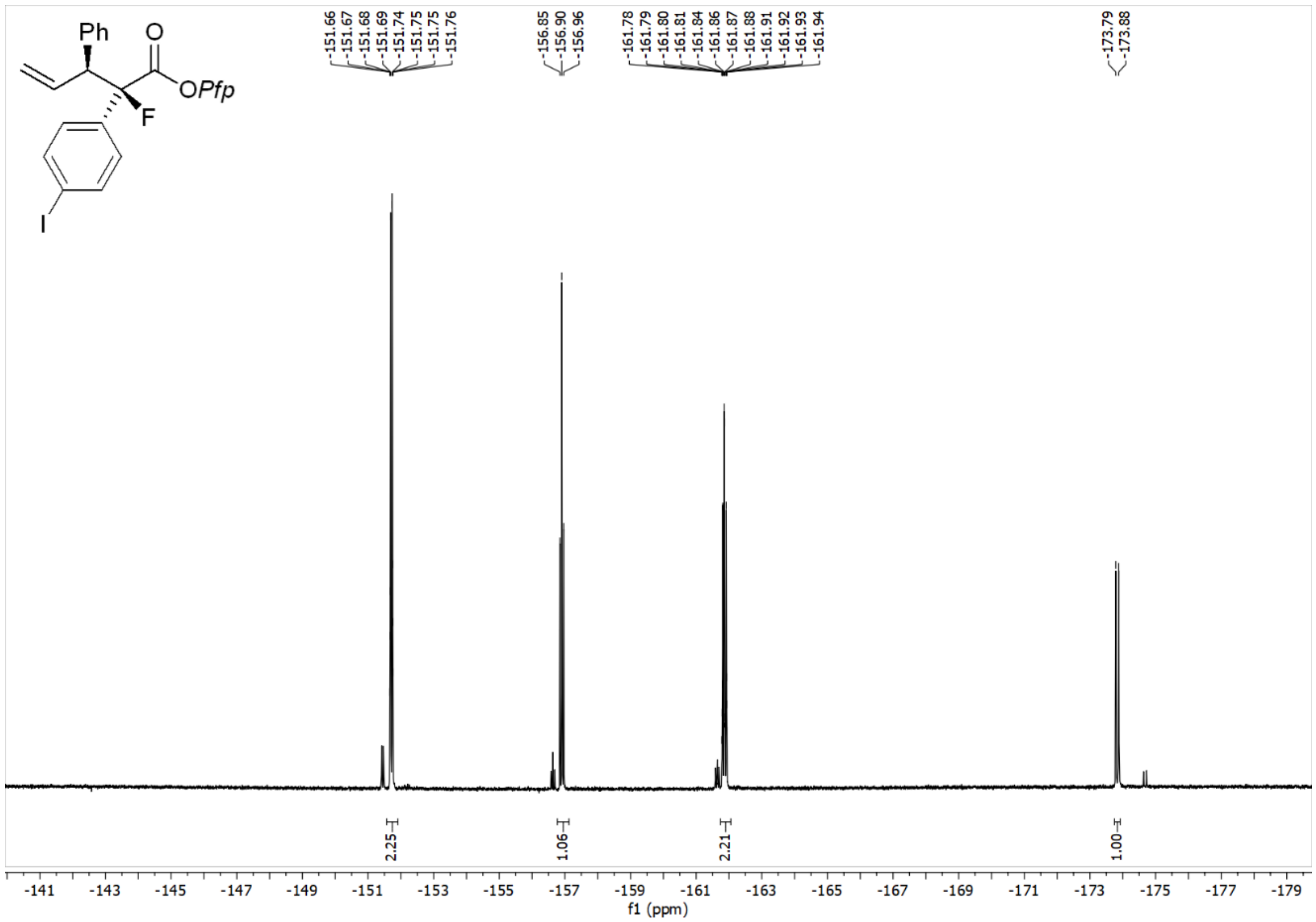

Ph
O
OPfp
F
I
-151.66
-151.67
-151.68
-151.69
-151.74
-151.75
-151.75
-151.76
-156.85
-156.90
-156.96
-161.78
-161.79
-161.80
-161.81
-161.84
-161.86
-161.87
-161.88
-161.91
-161.92
-161.93
-161.94
-173.79
-173.88
2.25
1.06
2.21
1.00
-141 -143 -145 -147 -149 -151 -153 -155 -157 -159 -161 -163 -165 -167 -169 -171 -173 -175 -177 -179
f1 (ppm)

**Perfluorophenyl (2*R*,3*R*)-2-([1,1'-biphenyl]-4-yl)-2-fluoro-3-phenylpent-4-enoate (*R*,*R*)-8**

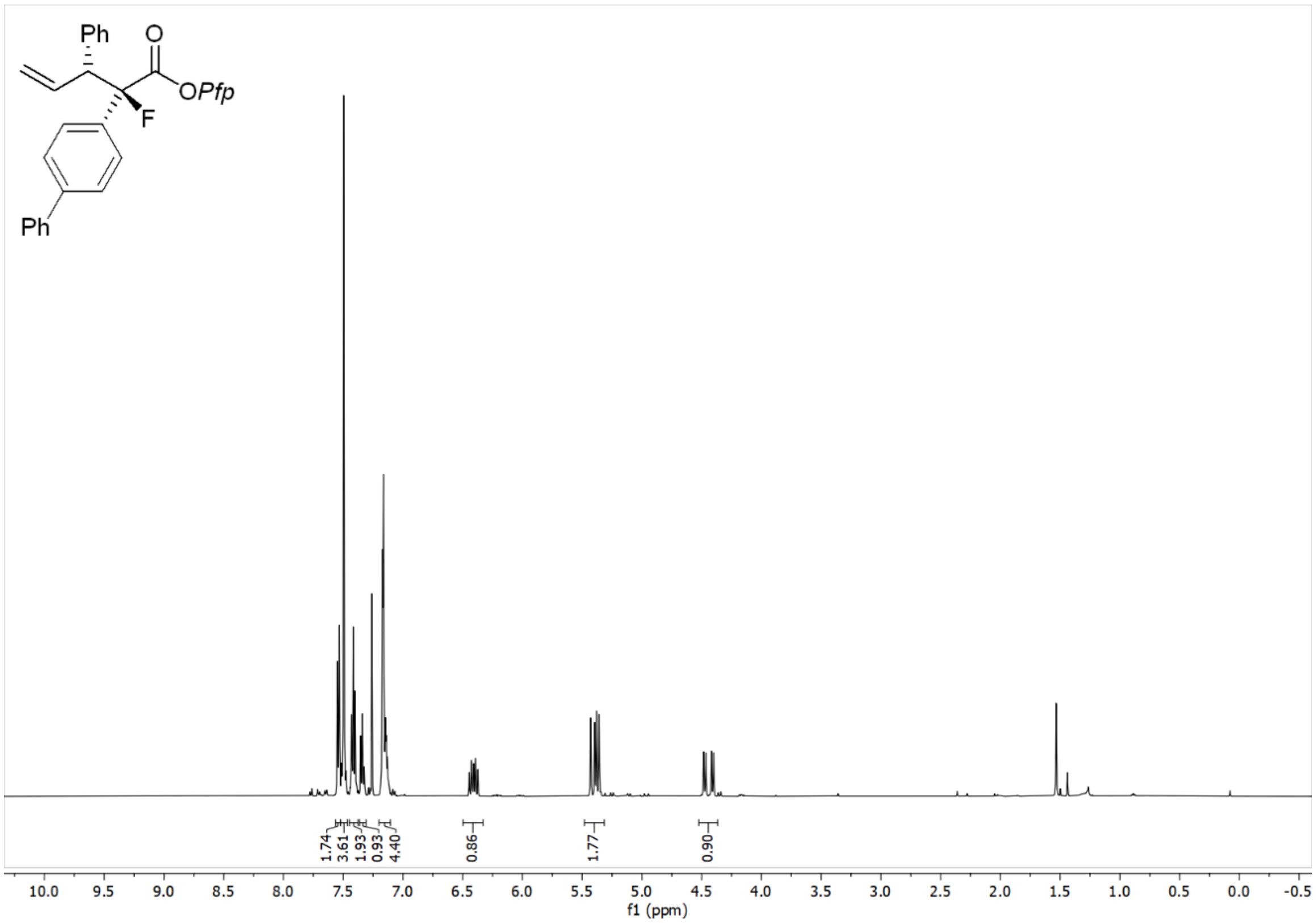

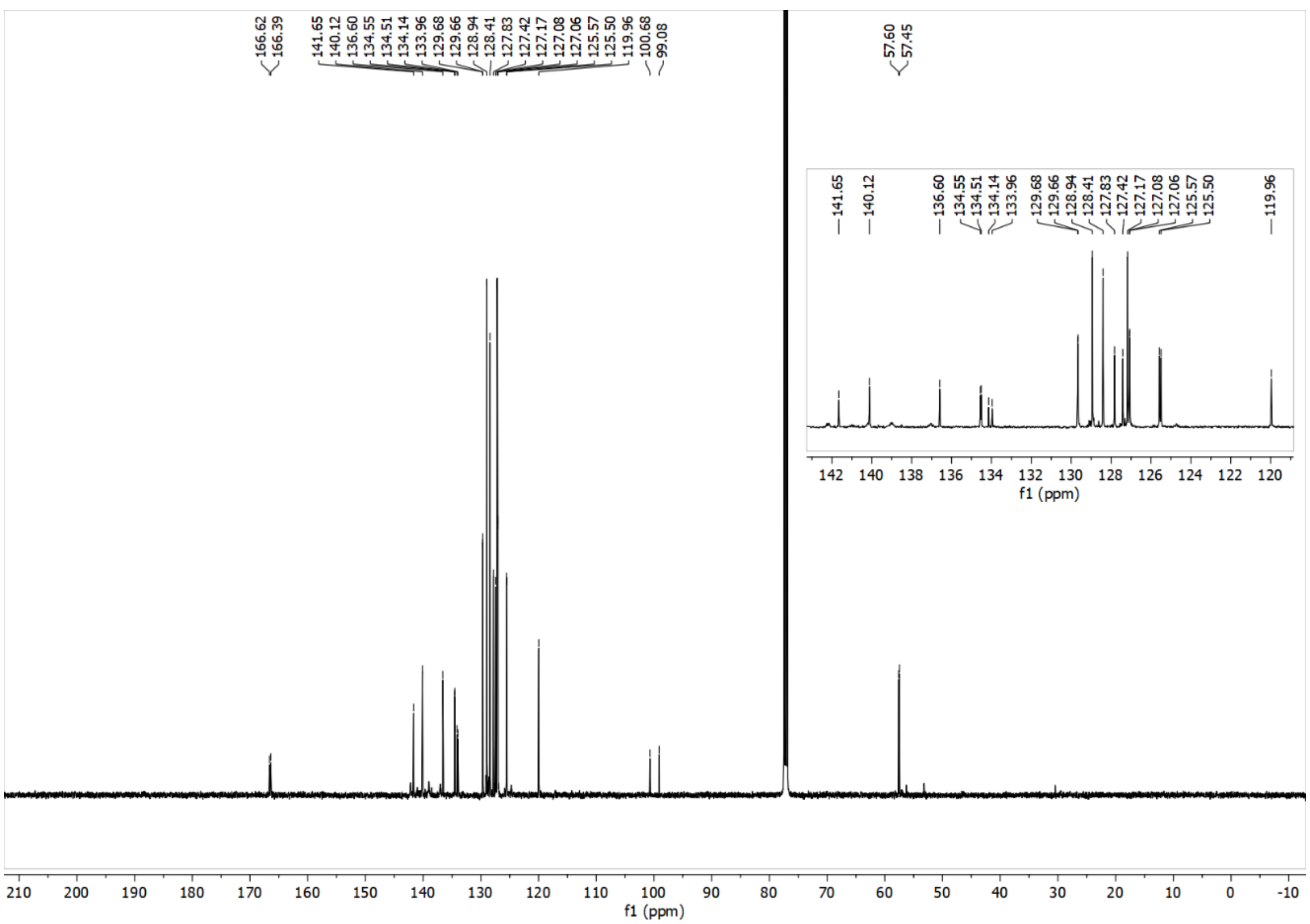

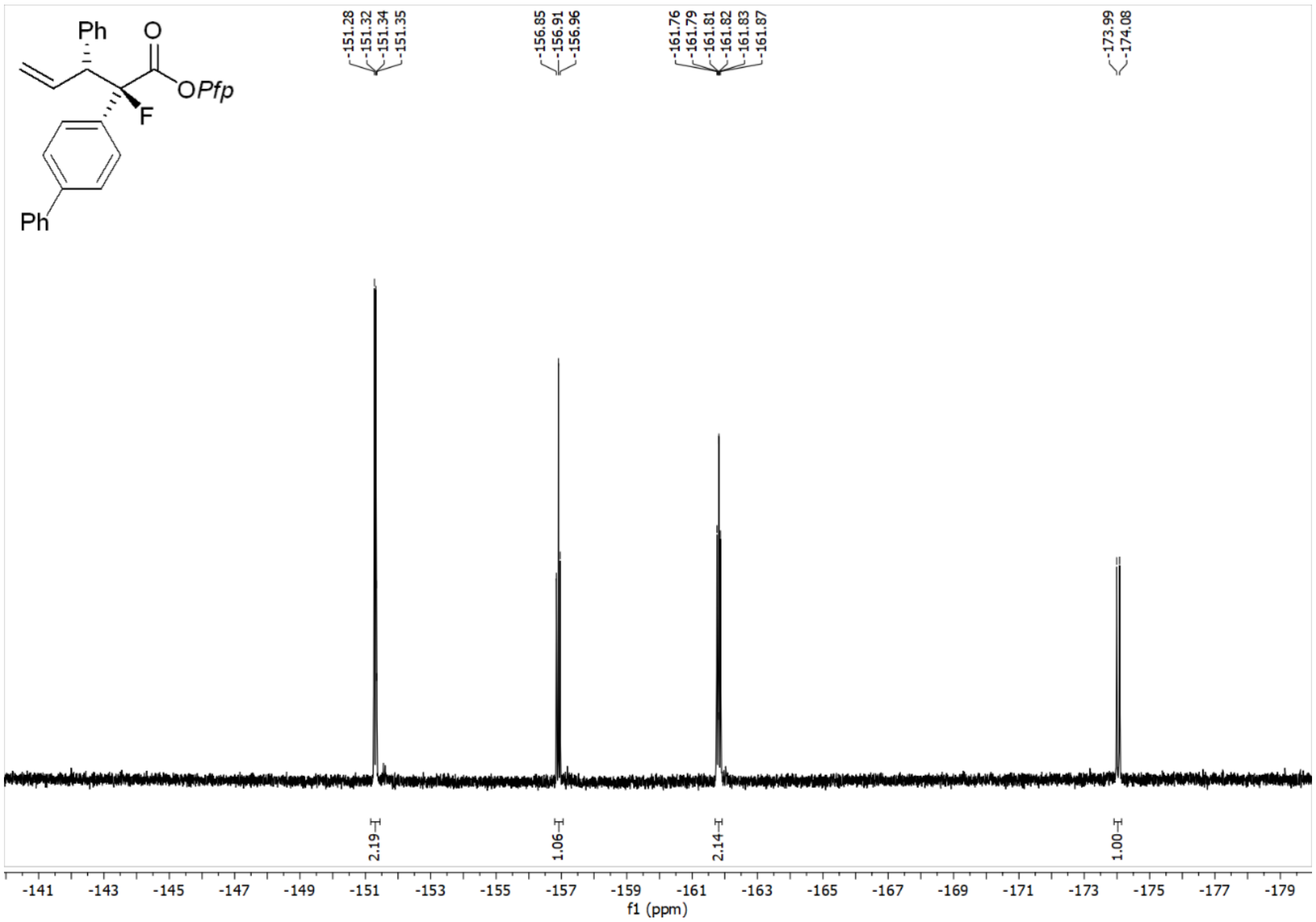
Ph
O
OPfp
F
Ph
-151.28
-151.32
-151.34
-151.35
-156.85
-156.91
-156.96
-161.76
-161.79
-161.81
-161.82
-161.83
-161.87
-173.99
-174.08
2.19
1.06
2.14
1.00
-141 -143 -145 -147 -149 -151 -153 -155 -157 -159 -161 -163 -165 -167 -169 -171 -173 -175 -177 -179
f1 (ppm)

**Perfluorophenyl (2*R*,3*S*)-2-([1,1'-biphenyl]-4-yl)-2-fluoro-3-phenylpent-4-enoate (*R*,*S*)-8:**

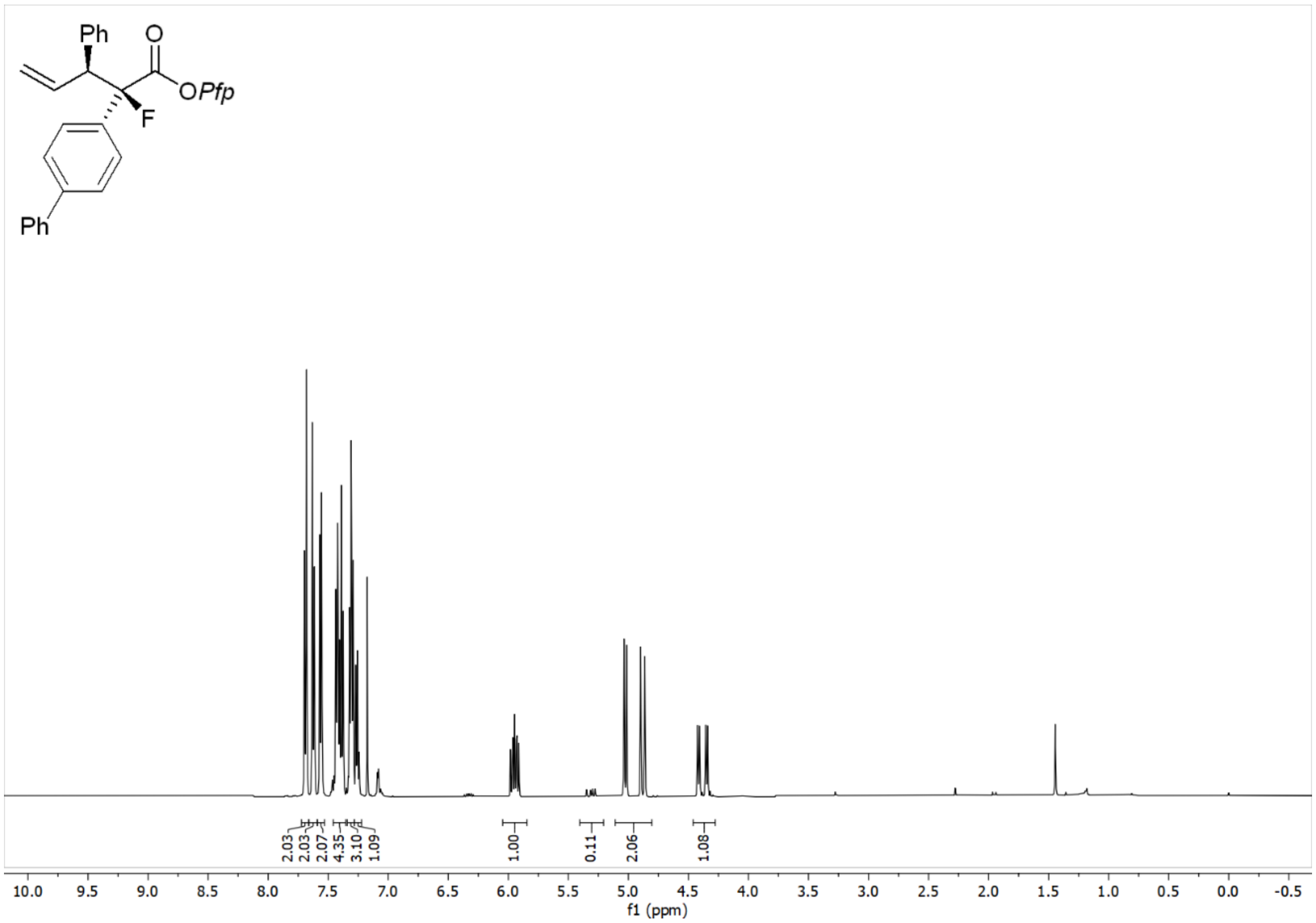


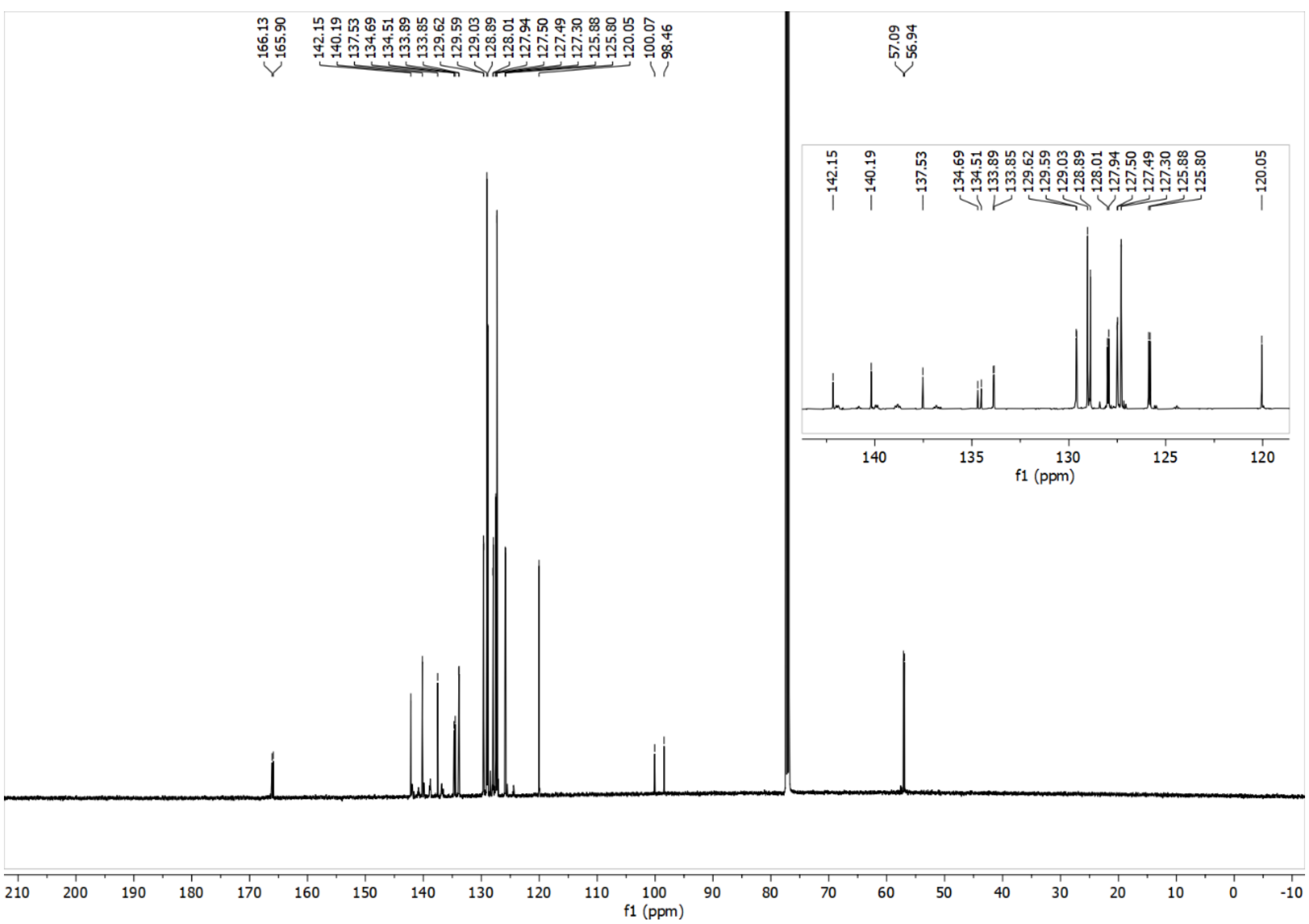

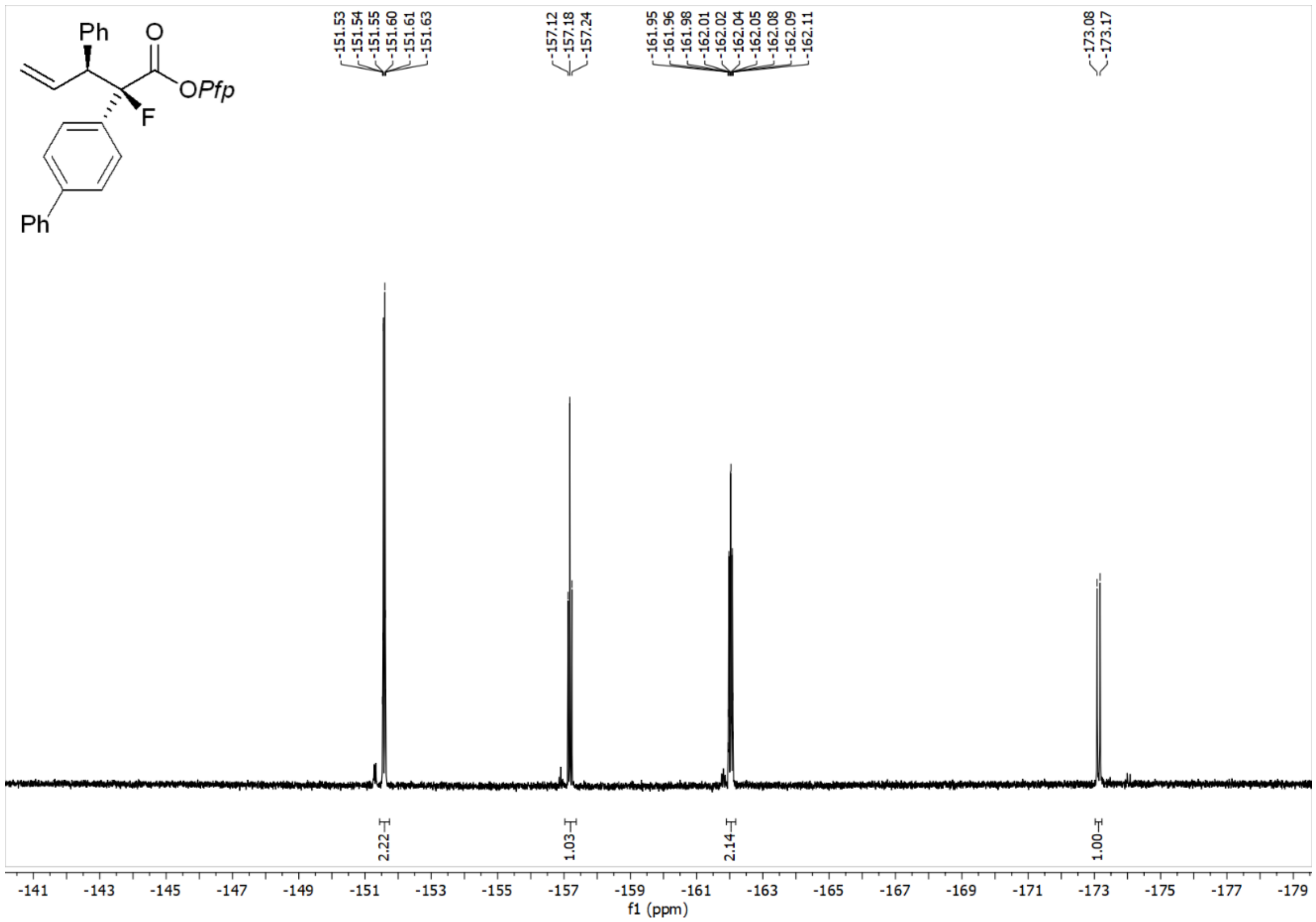
Ph
O
OPfp
F
Ph
-151.53
-151.54
-151.55
-151.60
-151.61
-151.63
-157.12
-157.18
-157.24
-161.95
-161.96
-161.98
-162.01
-162.02
-162.04
-162.05
-162.08
-162.09
-162.11
-173.08
-173.17
2.22
1.03
2.14
1.00
-141
-143
-145
-147
-149
-151
-153
-155
-157
-159
-161
-163
-165
-167
-169
-171
-173
-175
-177
-179
f1 (ppm)

**Perfluorophenyl (2*R*,3*R*)-2-fluoro-3-phenyl-2-(4-(trifluoromethoxy)phenyl)pent-4-enoate (*R*,*R*)-14):**

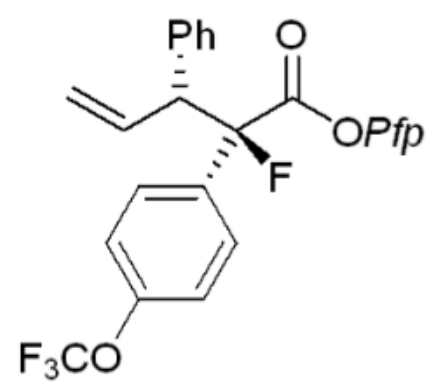


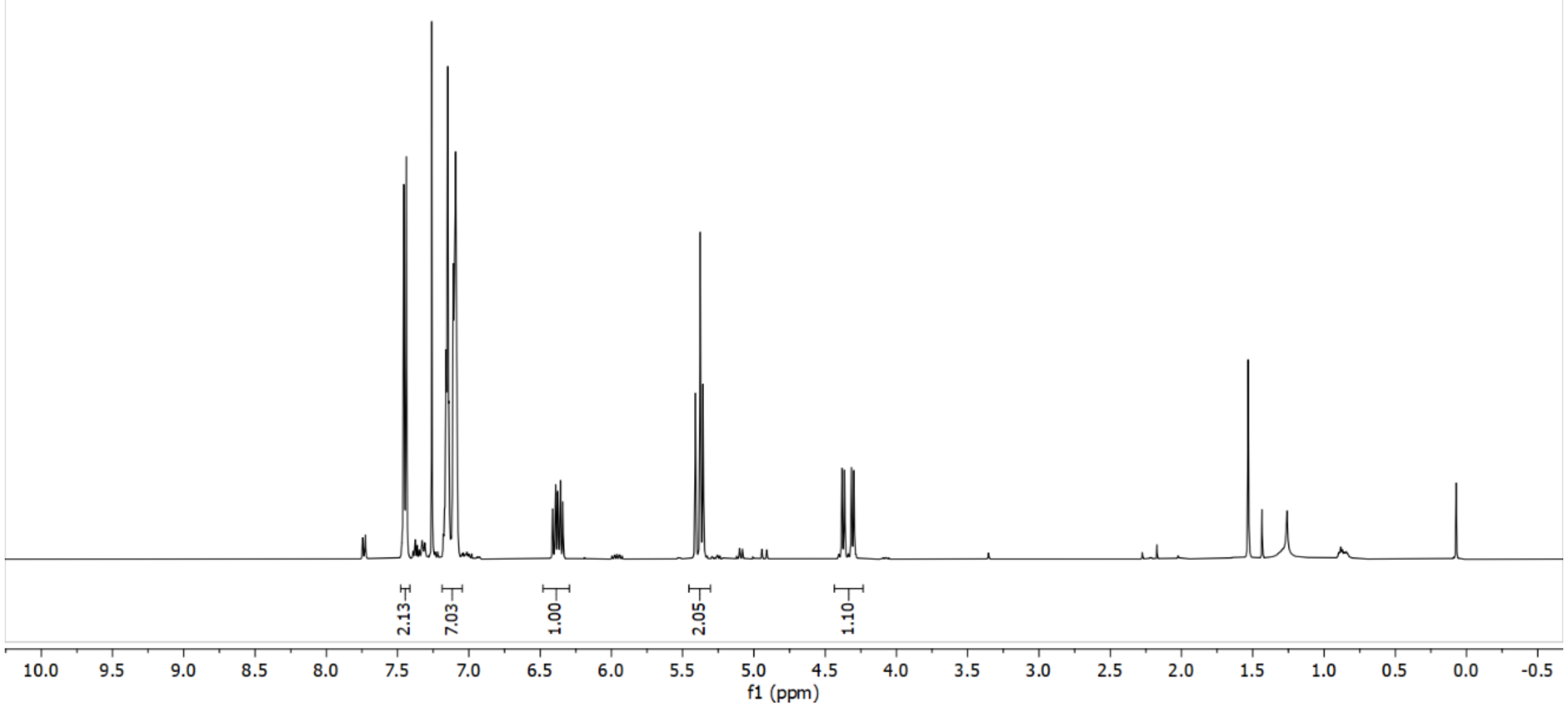

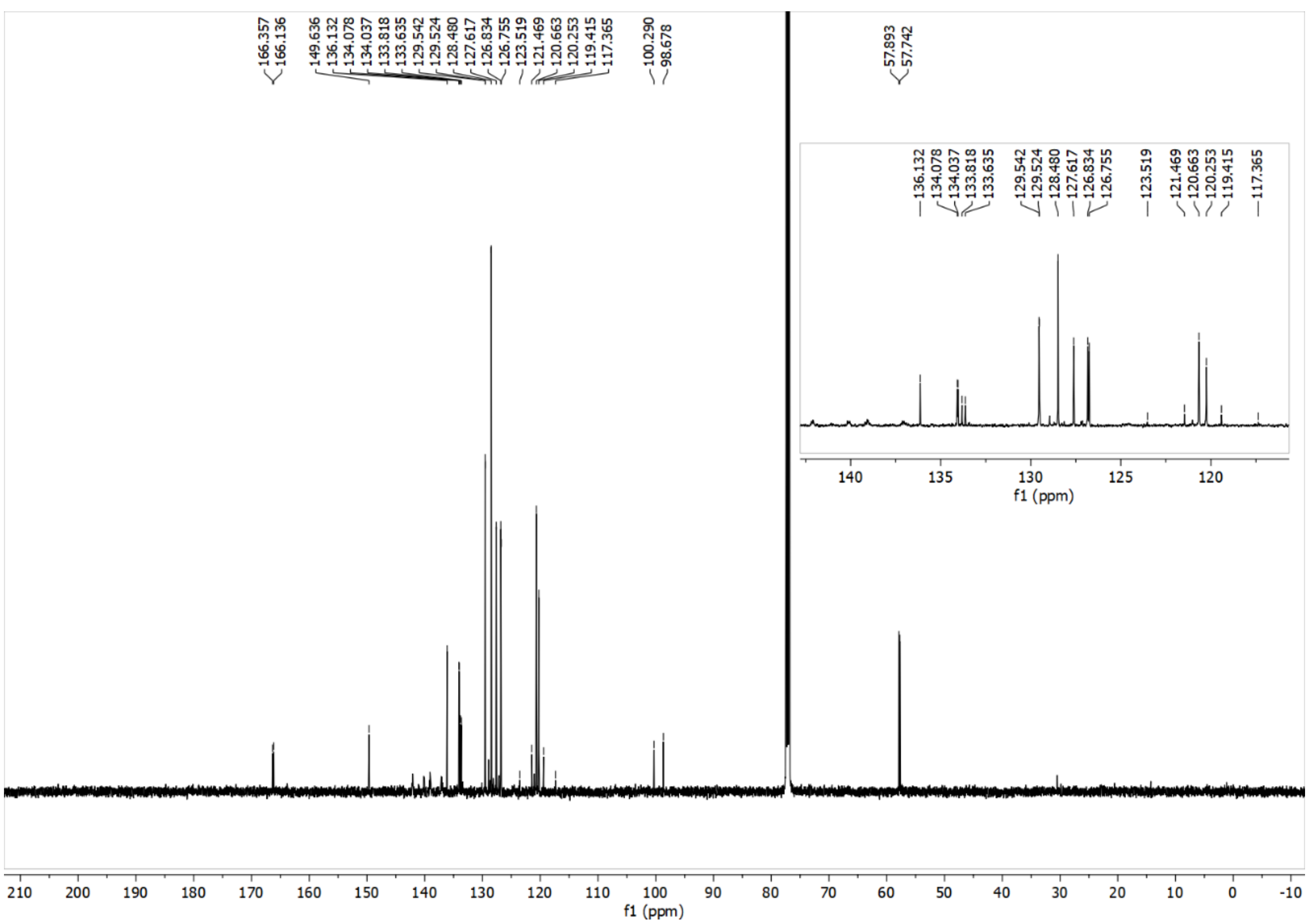

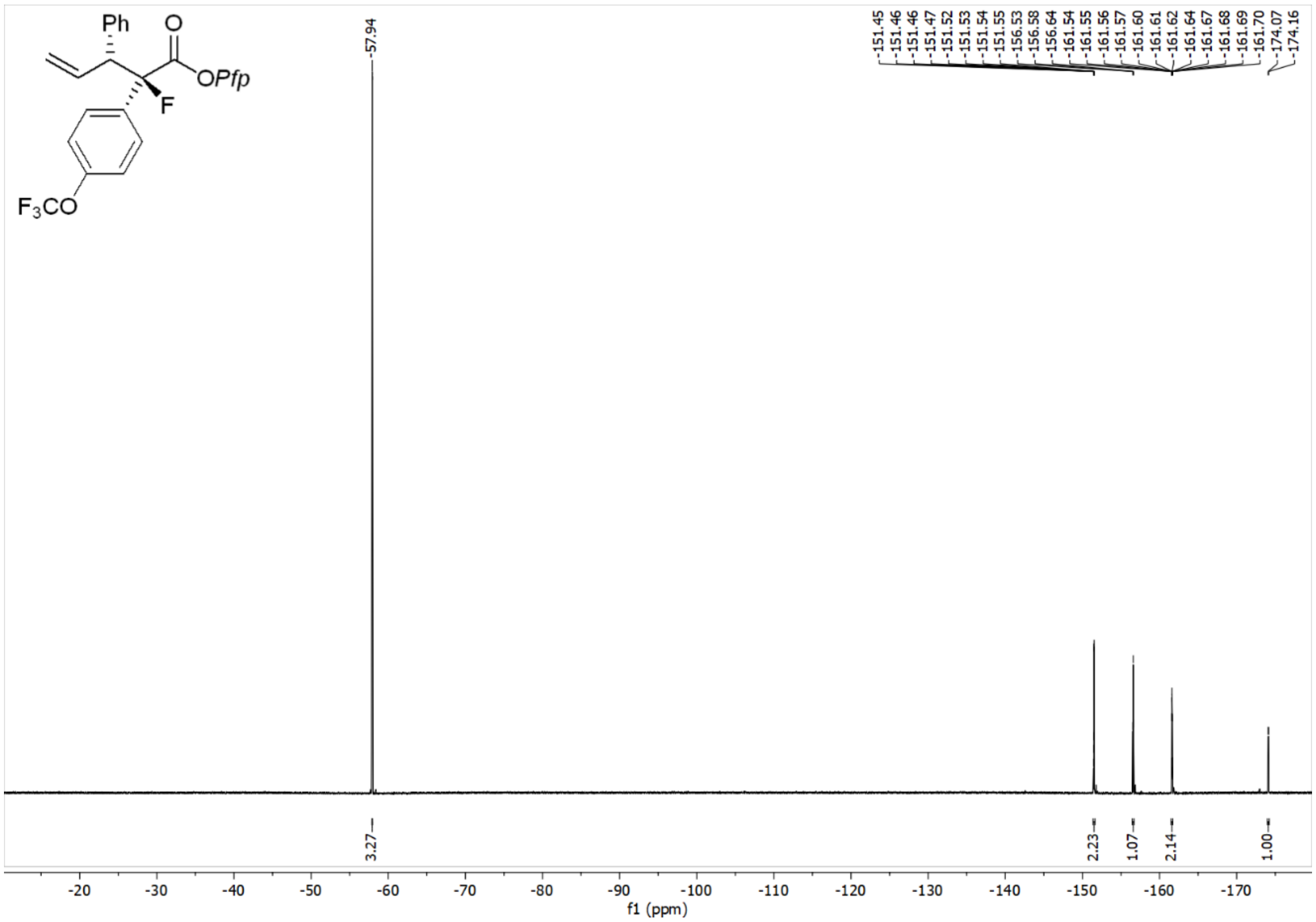

Ph
O
OPfp
F
F3CO
-57.94
-151.45
-151.46
-151.46
-151.47
-151.52
-151.53
-151.54
-151.55
-156.53
-156.58
-156.64
-161.54
-161.55
-161.56
-161.57
-161.60
-161.61
-161.62
-161.64
-161.67
-161.68
-161.69
-161.70
-174.07
-174.16
3.27
2.23
1.07
2.14
1.00
-20
-30
-40
-50
-60
-70
-80
-90
-100
-110
-120
-130
-140
-150
-160
-170
f1 (ppm)

**Perfluorophenyl (2*R*,3*S*)-2-fluoro-3-phenyl-2-(4-(trifluoromethoxy)phenyl)pent-4-enoate (*R*,*S*)-14:**

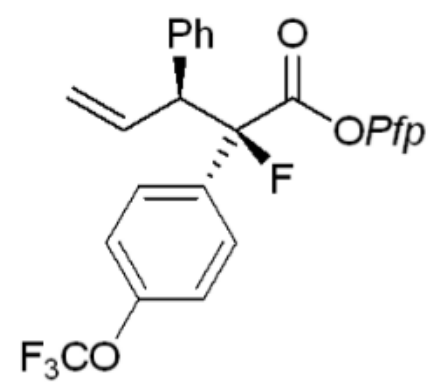


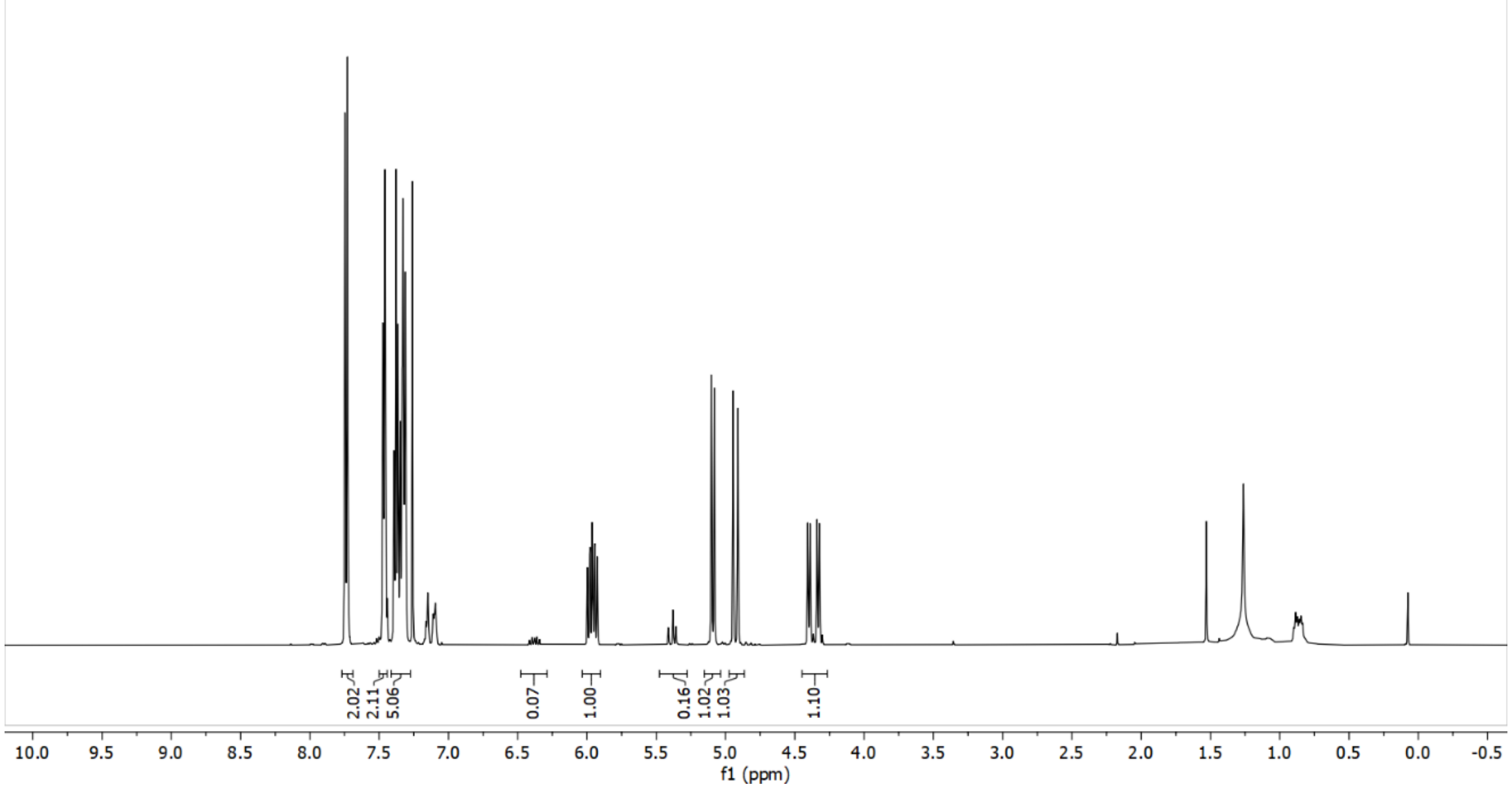


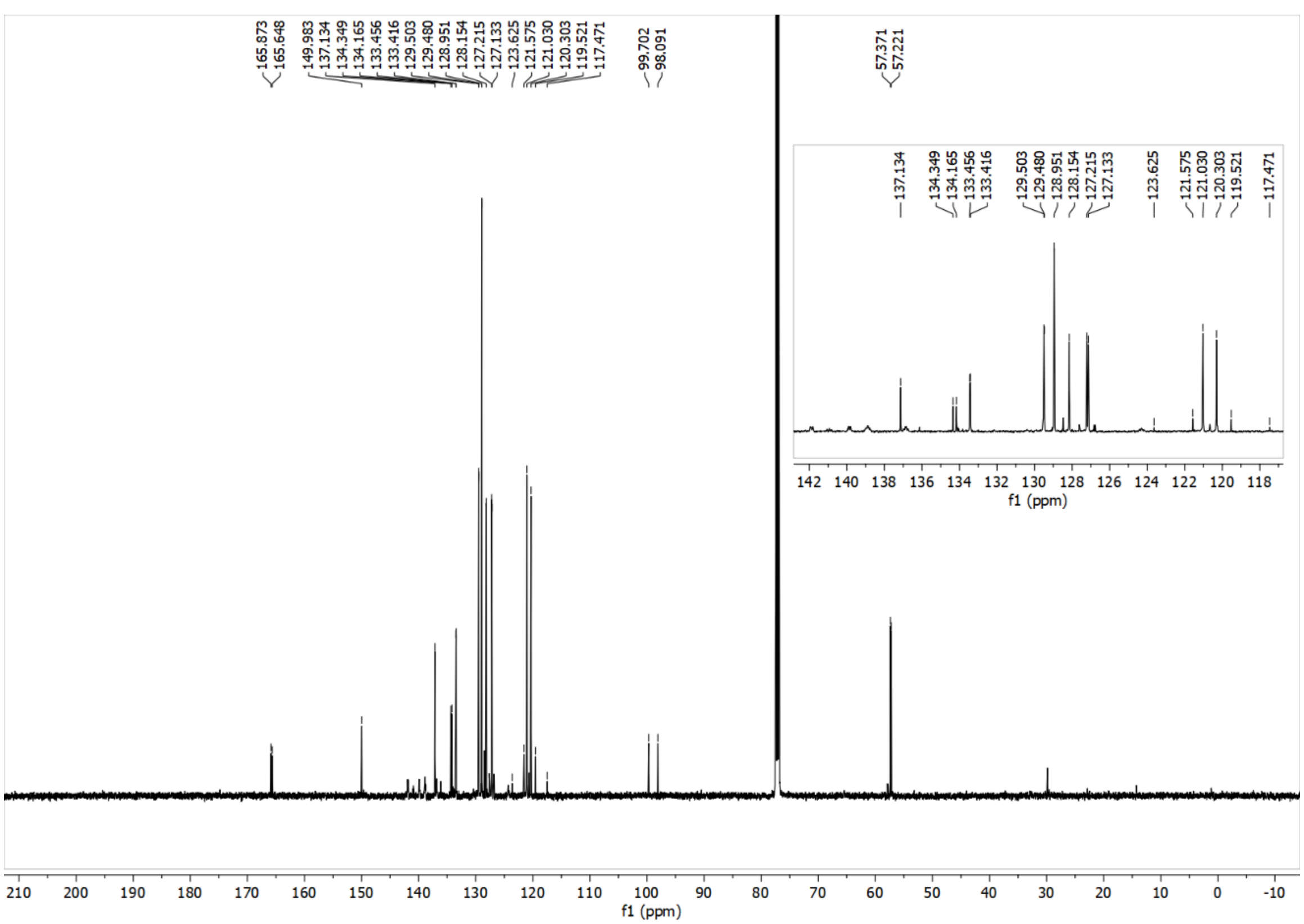

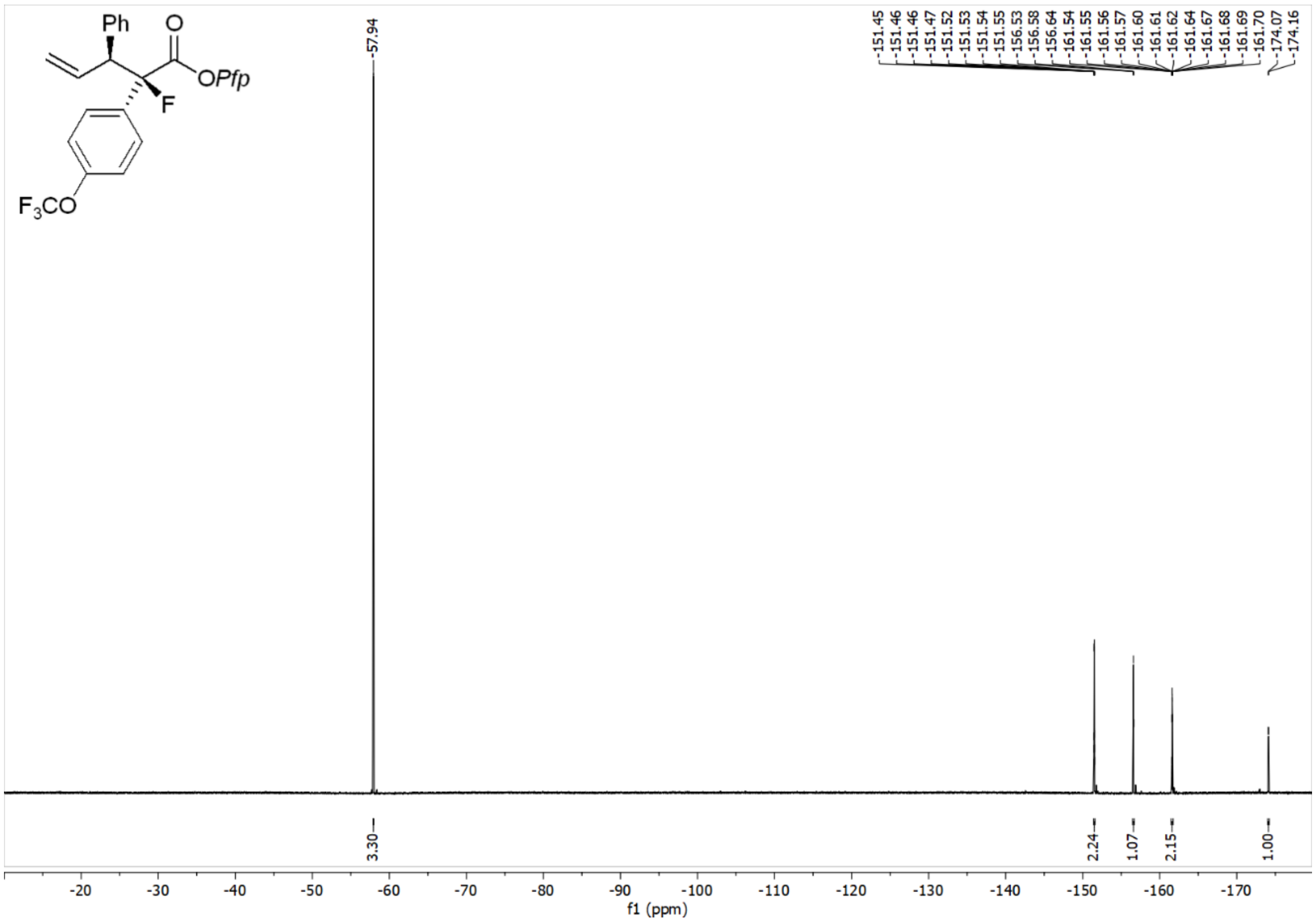
Ph
O
OPfp
F
F3CO
-57.94
-151.45
-151.46
-151.46
-151.47
-151.52
-151.53
-151.54
-151.55
-156.53
-156.58
-156.64
-161.54
-161.55
-161.56
-161.57
-161.60
-161.61
-161.62
-161.64
-161.67
-161.68
-161.69
-161.70
-174.07
-174.16
3.30
2.24
1.07
2.15
1.00
-20
-30
-40
-50
-60
-70
-80
-90
-100
-110
-120
-130
-140
-150
-160
-170
f1 (ppm)

**Perfluorophenyl (2*R*,3*R*)-2-fluoro-3-phenyl-2-(4-(trifluoromethyl)phenyl)pent-4-enoate (*R*,*R*)-13:**

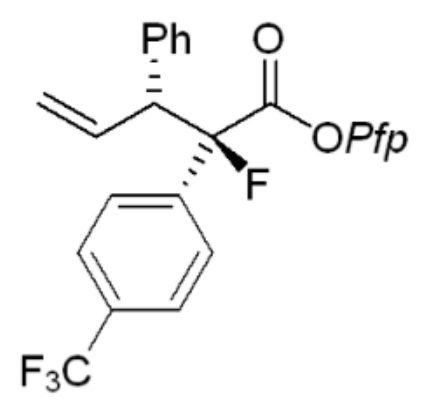


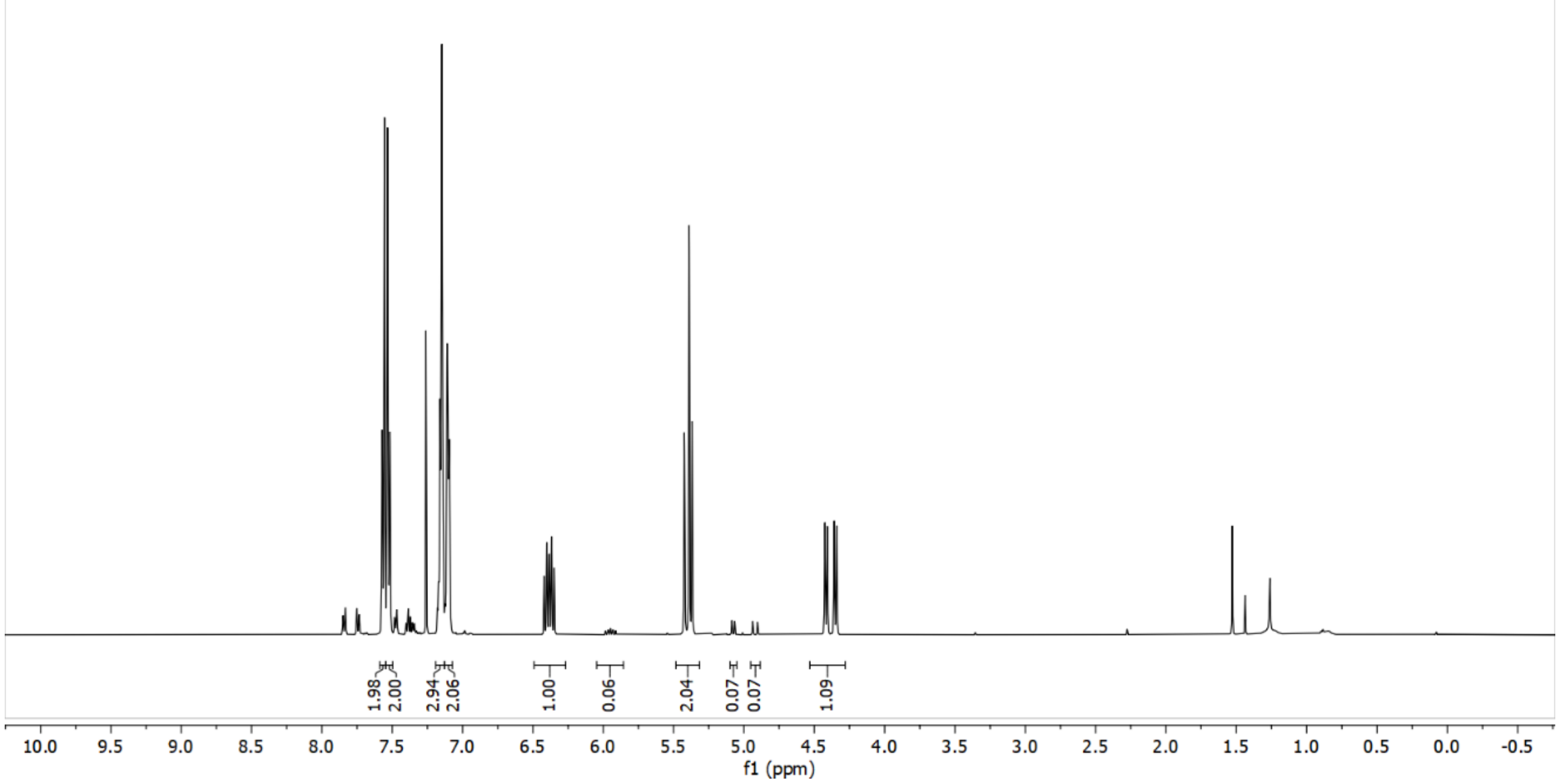


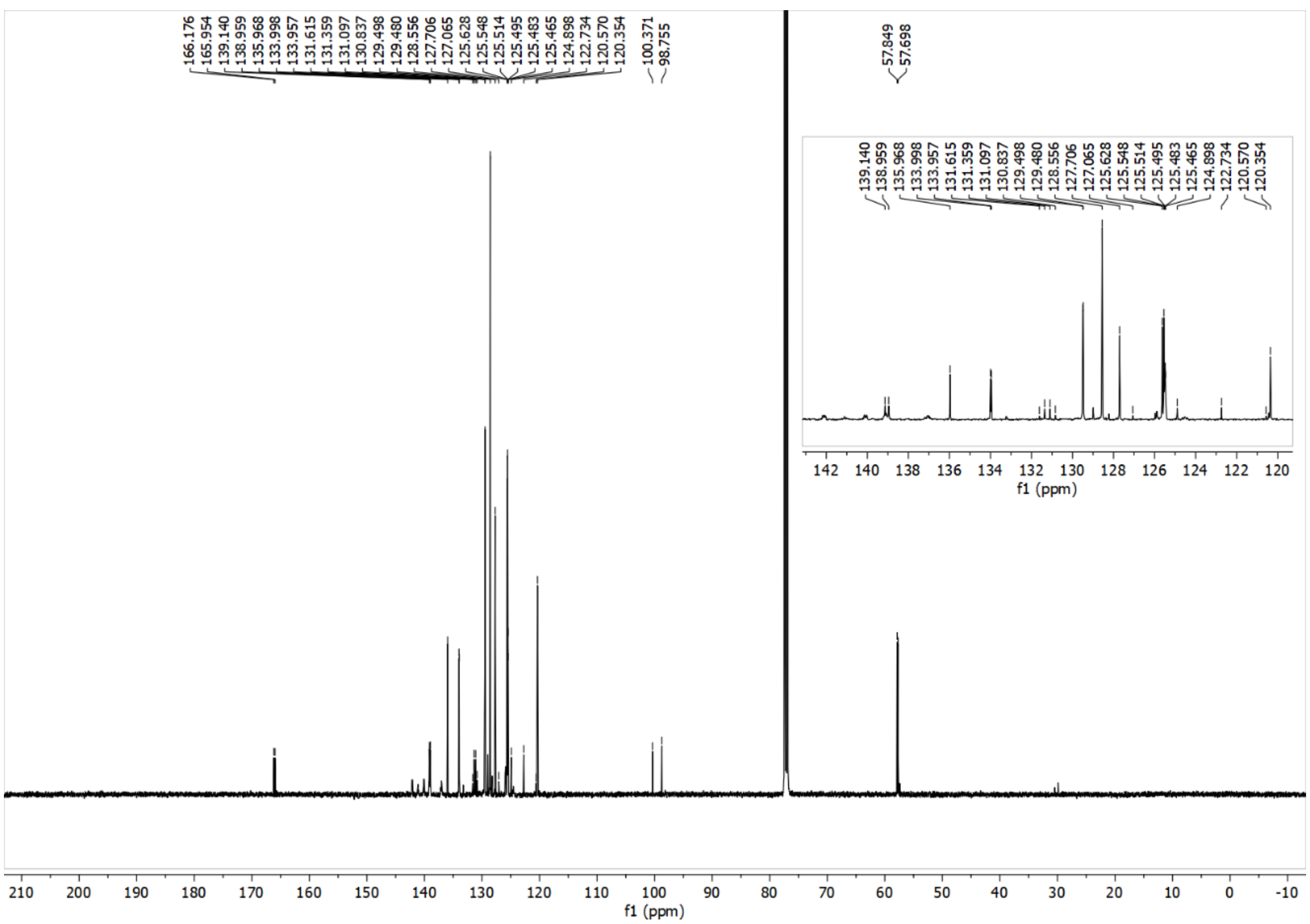

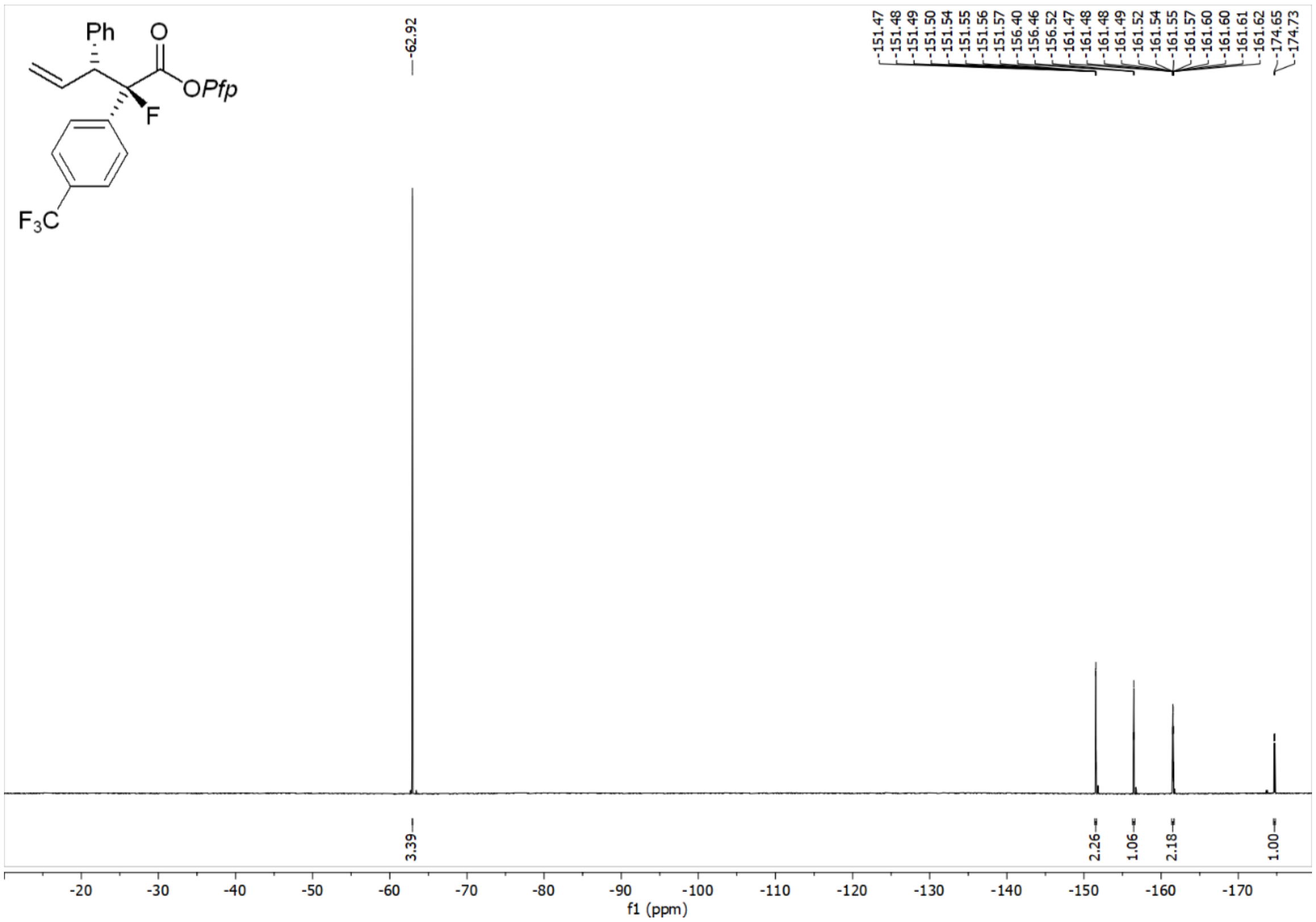

Ph
O
OPfp
F
F3C
-62.92
-151.47
-151.48
-151.49
-151.50
-151.54
-151.55
-151.56
-151.57
-156.40
-156.46
-156.52
-161.47
-161.48
-161.49
-161.52
-161.54
-161.55
-161.57
-161.60
-161.60
-161.61
-161.62
-174.65
-174.73
3.39
2.26
1.06
2.18
1.00
-20
-30
-40
-50
-60
-70
-80
-90
-100
-110
-120
-130
-140
-150
-160
-170
f1 (ppm)

**Perfluorophenyl (2*R*,3*S*)-2-fluoro-3-phenyl-2-(4-(trifluoromethyl)phenyl)pent-4-enoate (*R*,*S*)-13:**

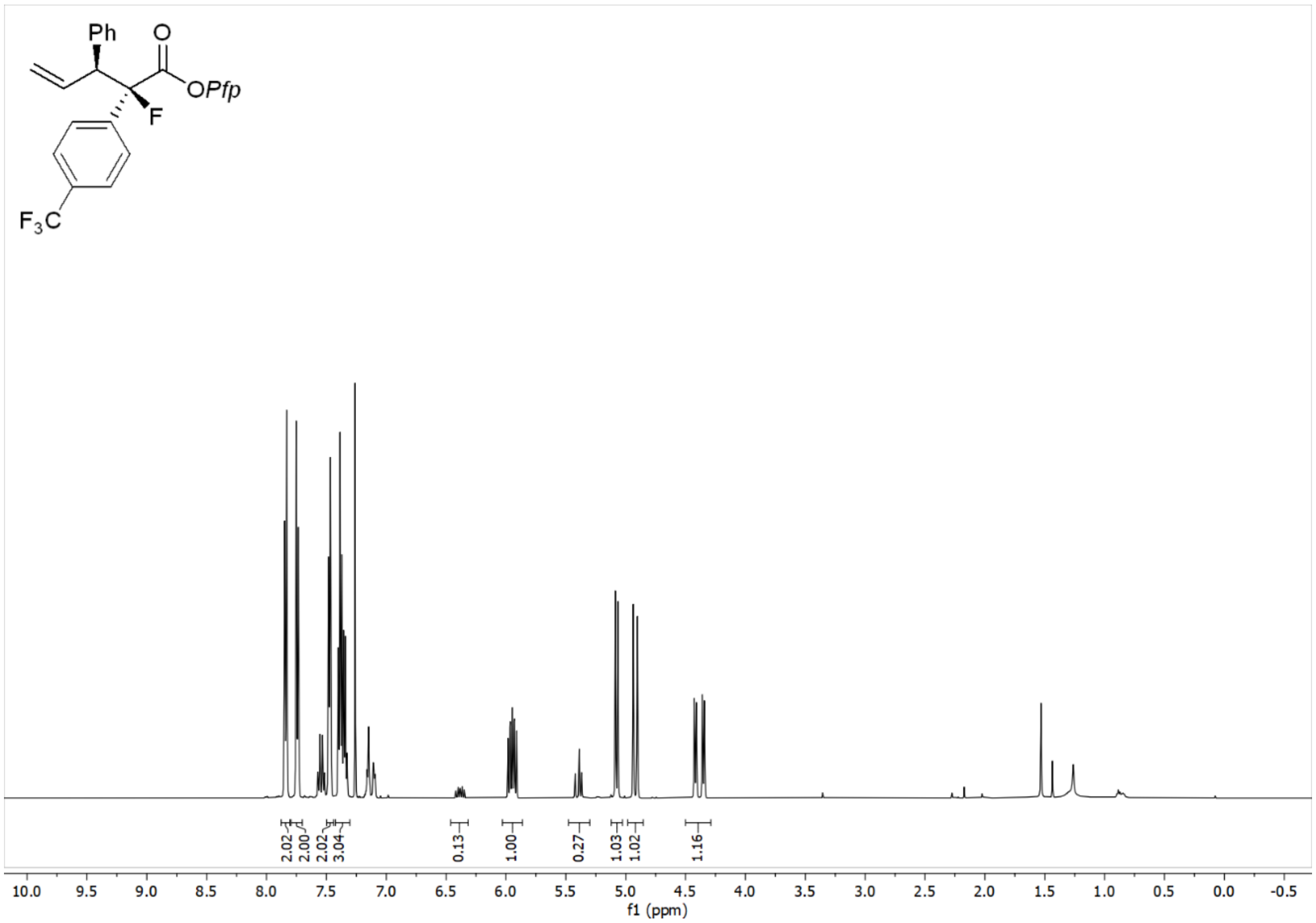


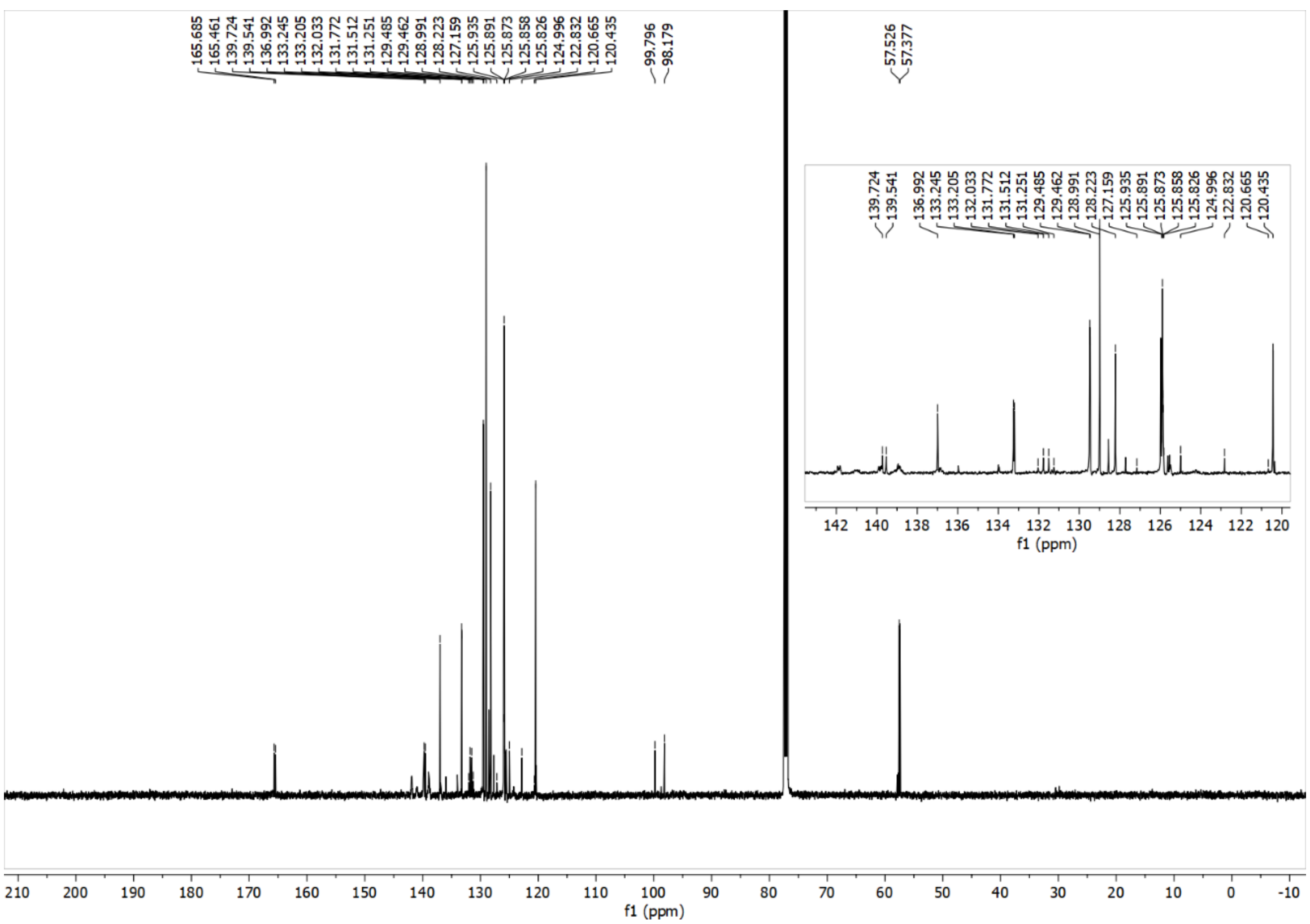

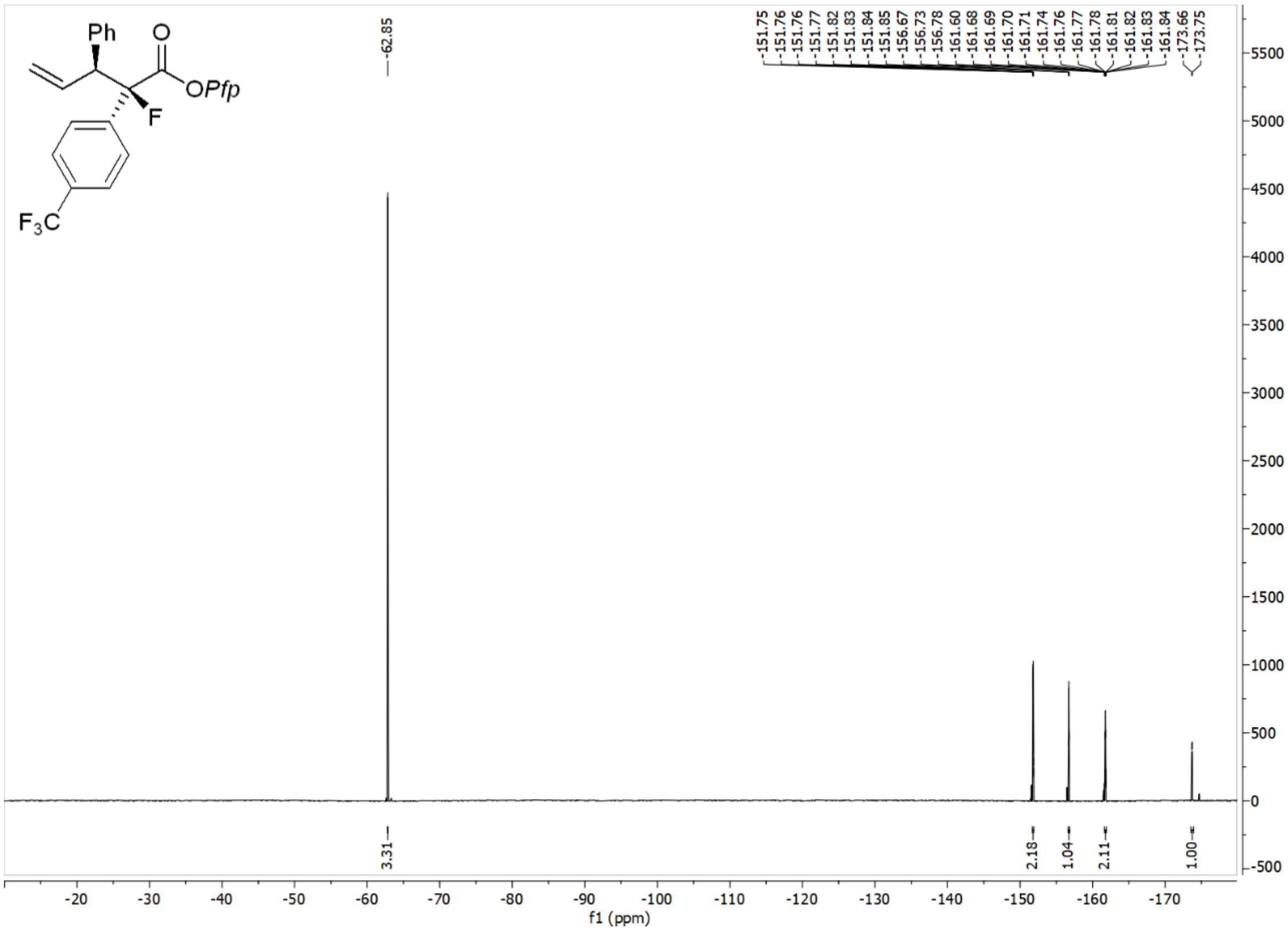

**Perfluorophenyl (2*R*,3*R*)-2-fluoro-2-(naphthalen-2-yl)-3-phenylpent-4-enoate (*R*,*R*)-4:**

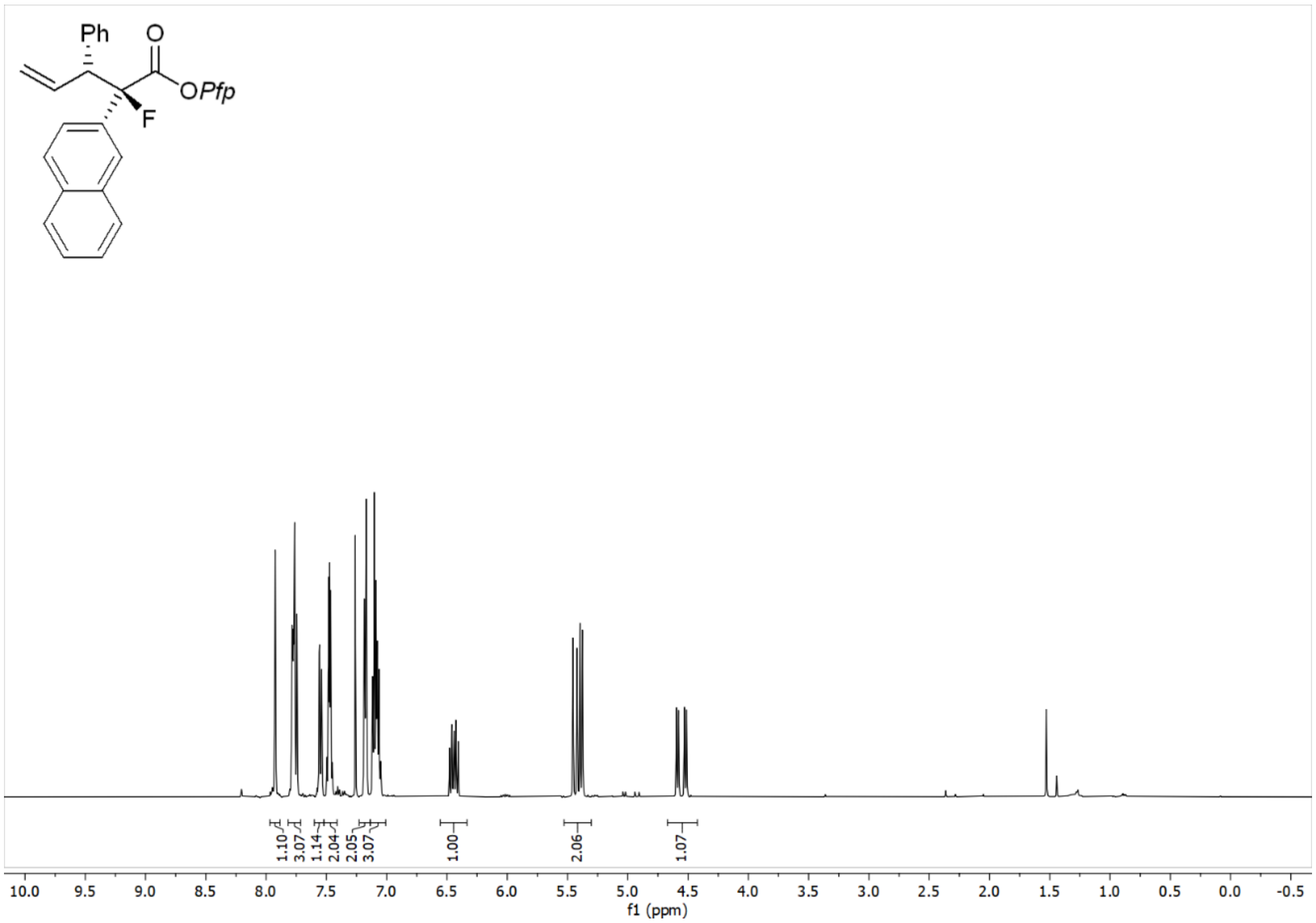


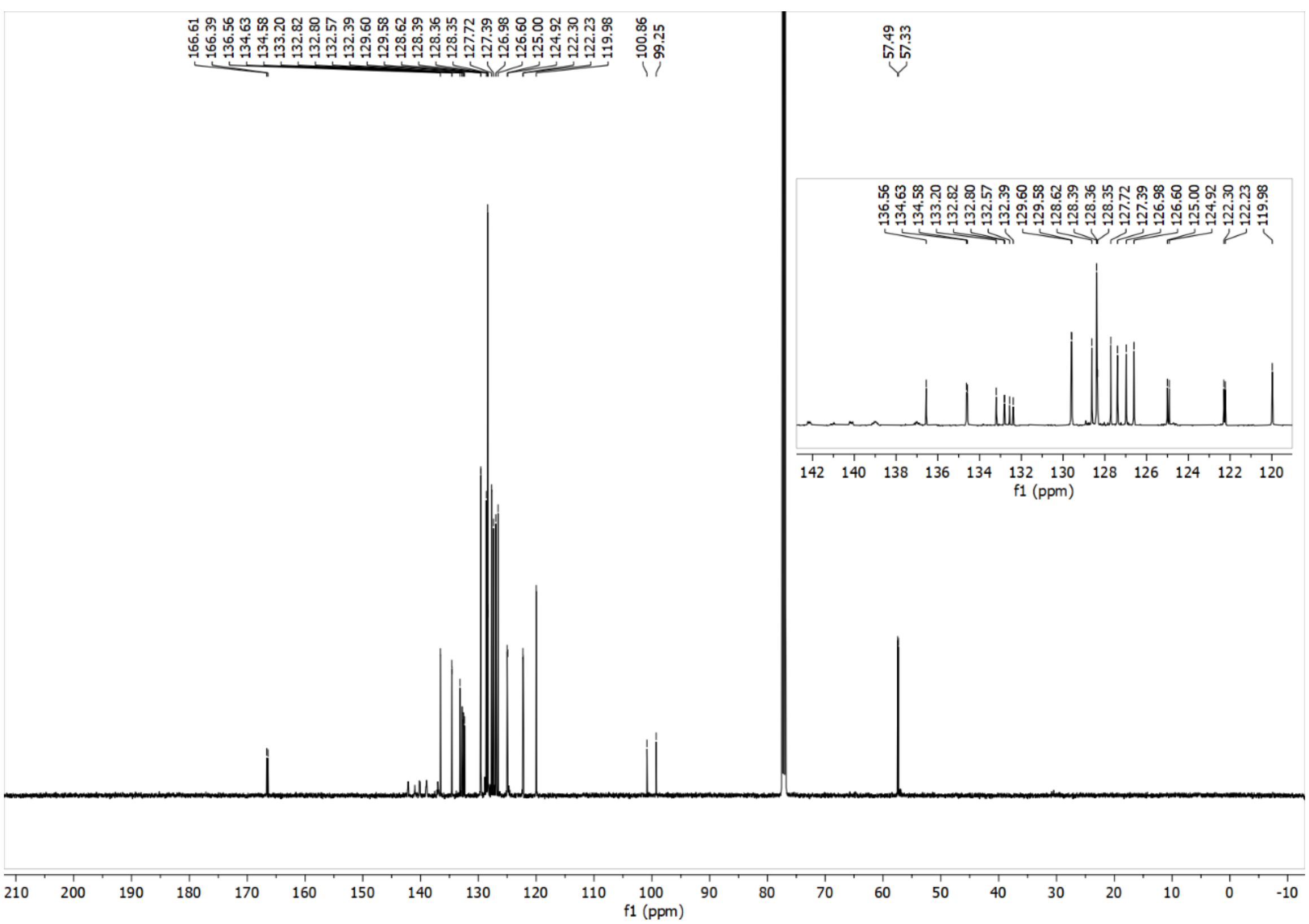

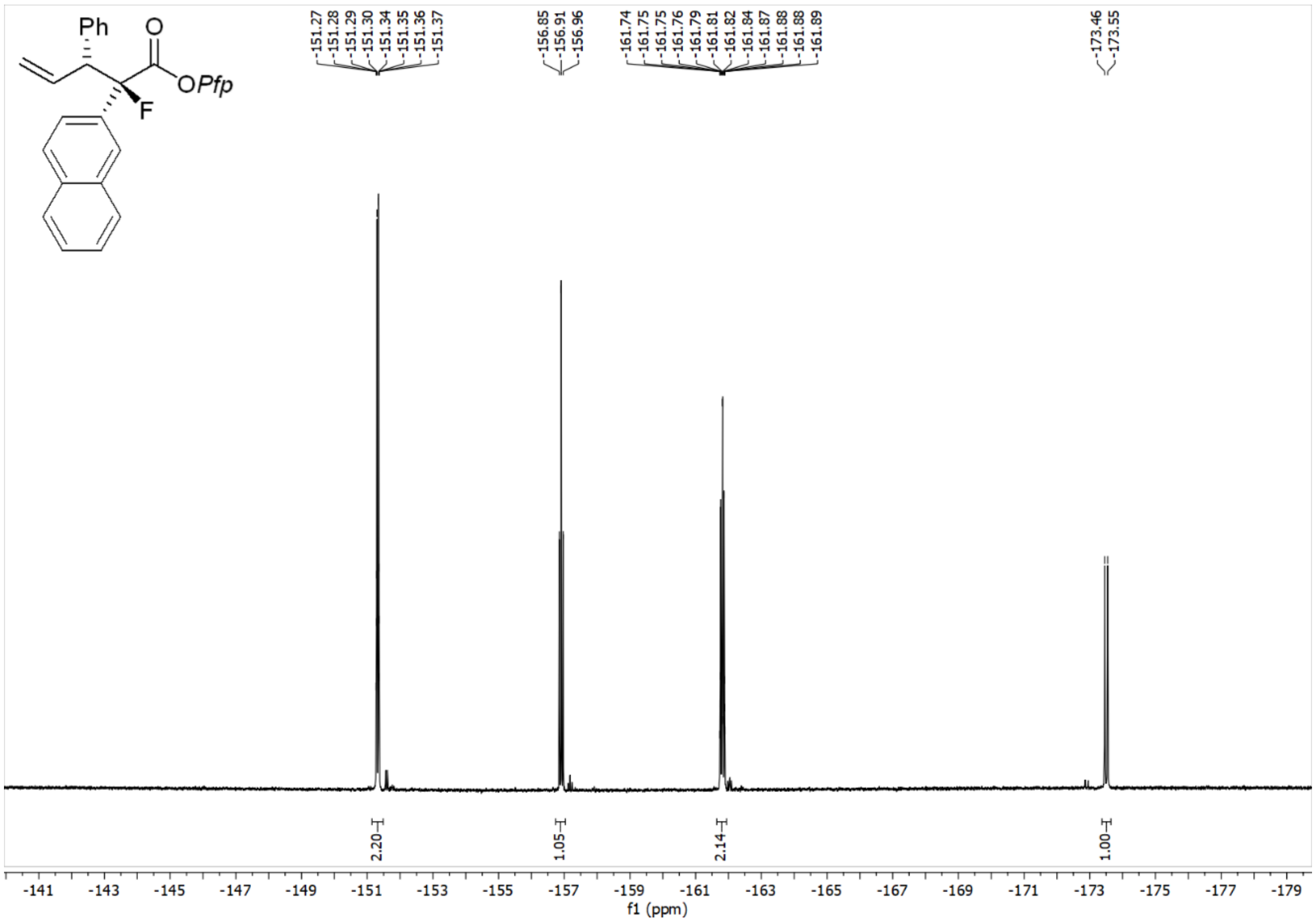

Ph
O
OPfp
F
-151.27
-151.28
-151.29
-151.30
-151.34
-151.35
-151.36
-151.37
-156.85
-156.91
-156.96
-161.74
-161.75
-161.75
-161.76
-161.79
-161.81
-161.82
-161.84
-161.87
-161.88
-161.88
-161.89
-173.46
-173.55
2.20
1.05
2.14
1.00
-141
-143
-145
-147
-149
-151
-153
-155
-157
-159
-161
-163
-165
-167
-169
-171
-173
-175
-177
-179
f1 (ppm)

**Perfluorophenyl (2*R*,3*S*)-2-fluoro-2-(naphthalen-2-yl)-3-phenylpent-4-enoate (*R*,*S*)-4:**

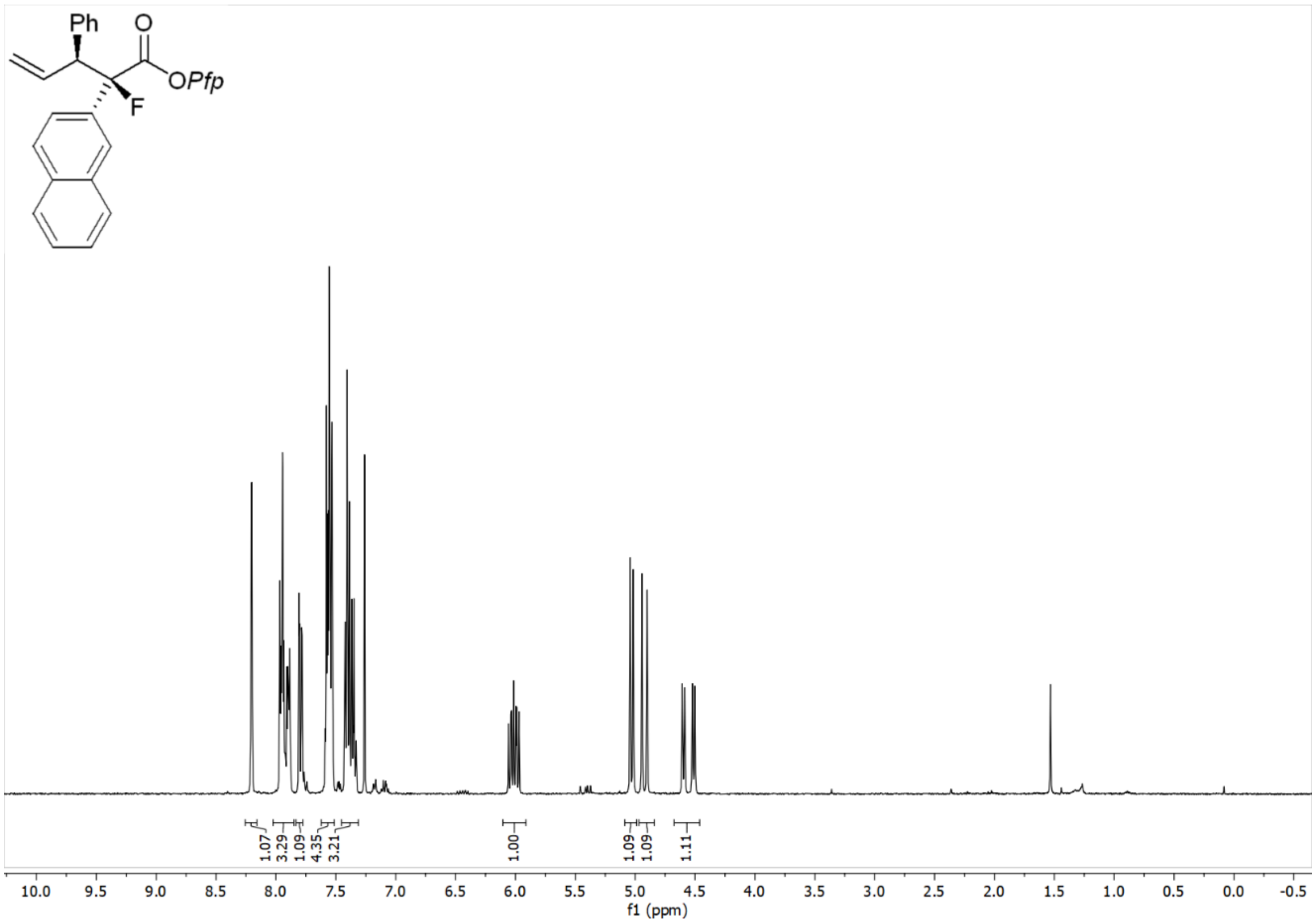


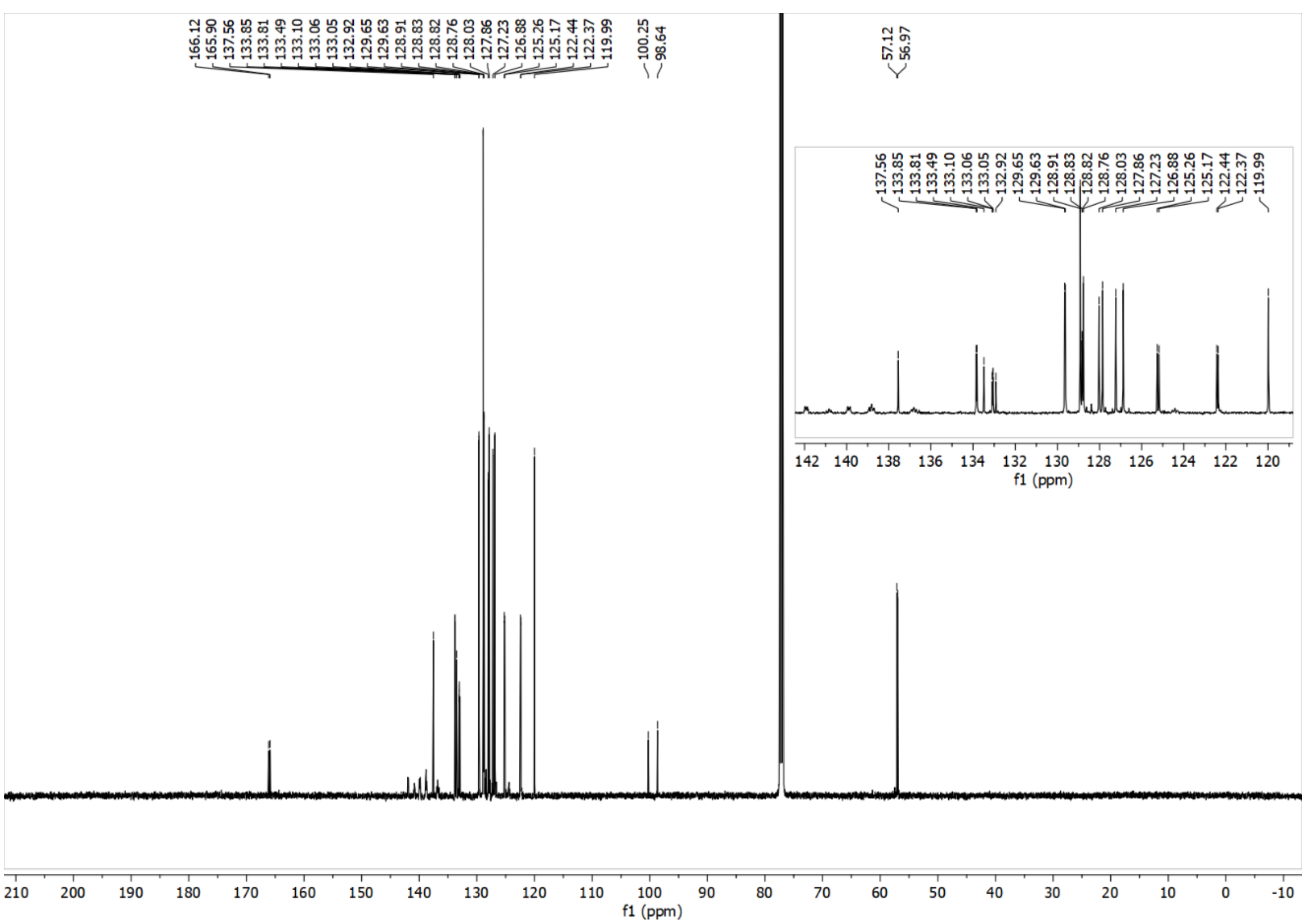

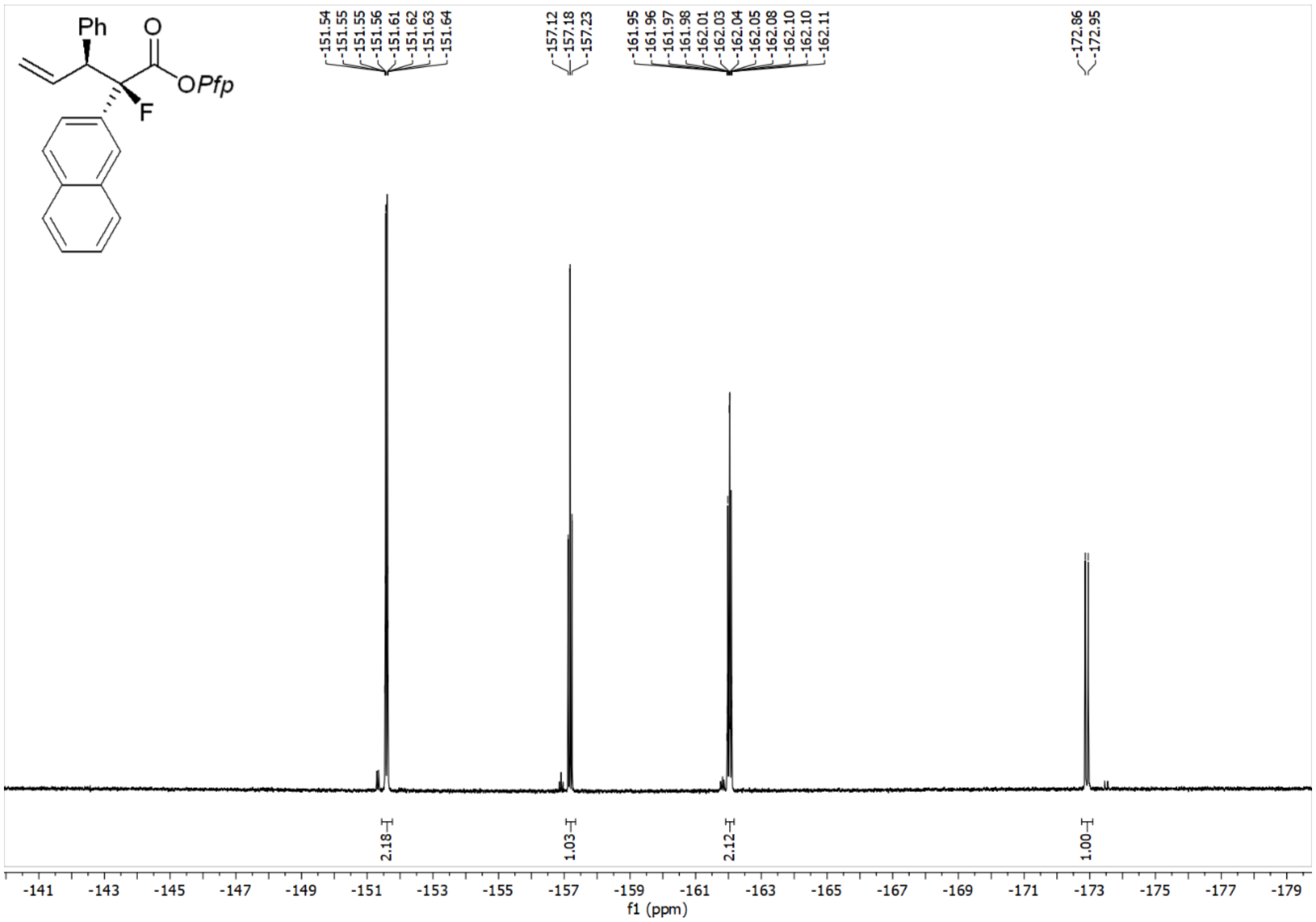

Ph
O
OPfp
F
-151.54
-151.55
-151.55
-151.56
-151.61
-151.62
-151.63
-151.64
-157.12
-157.18
-157.23
-161.95
-161.96
-161.97
-161.98
-162.01
-162.03
-162.04
-162.05
-162.08
-162.10
-162.10
-162.11
-172.86
-172.95
2.18
1.03
2.12
1.00
-141 -143 -145 -147 -149 -151 -153 -155 -157 -159 -161 -163 -165 -167 -169 -171 -173 -175 -177 -179
f1 (ppm)

**(2*R*,3*R*)-2-Fluoro-2-(3-methoxyphenyl)-3-phenylpent-4-enamide (*R*,*R*)-5:**

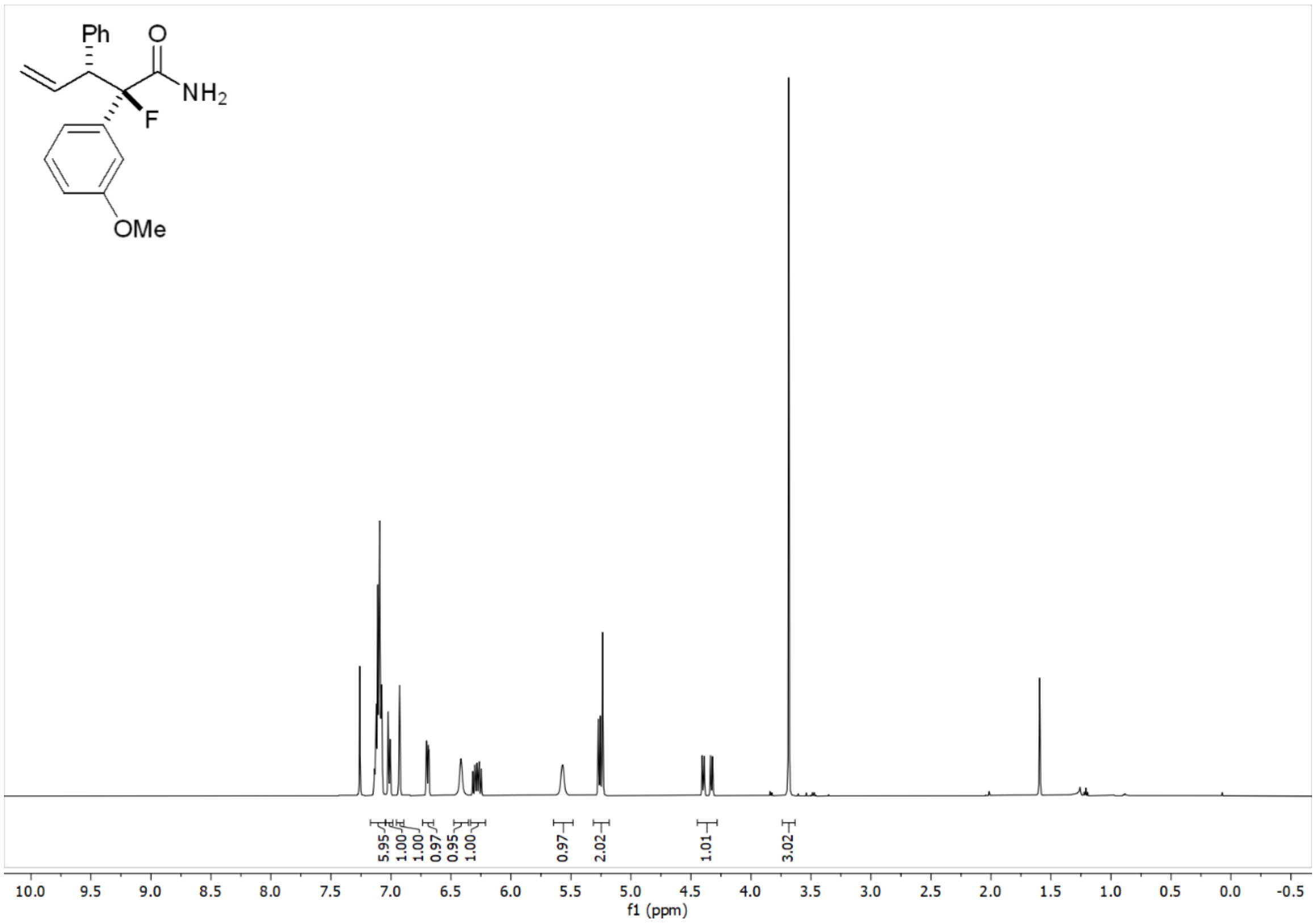

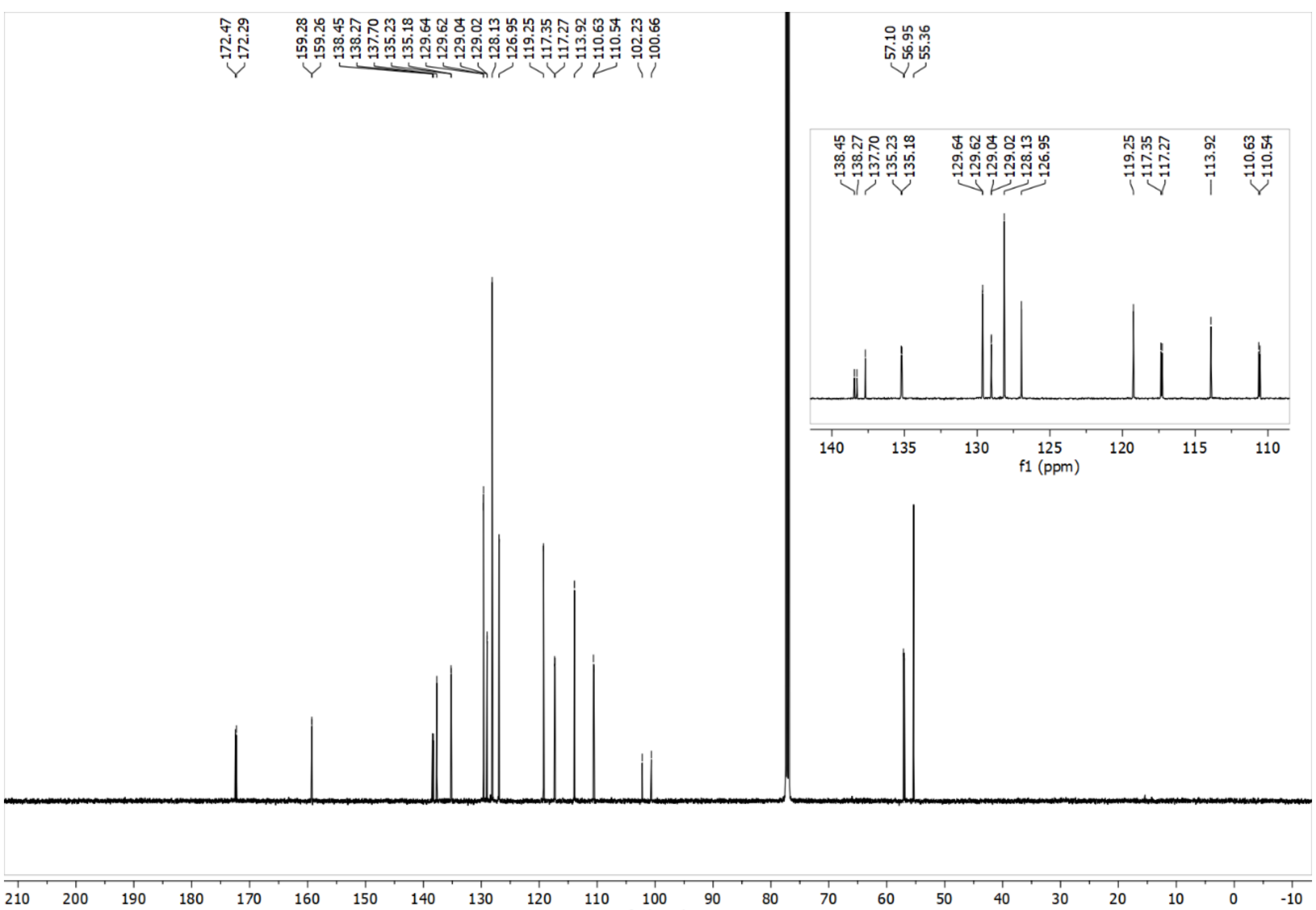

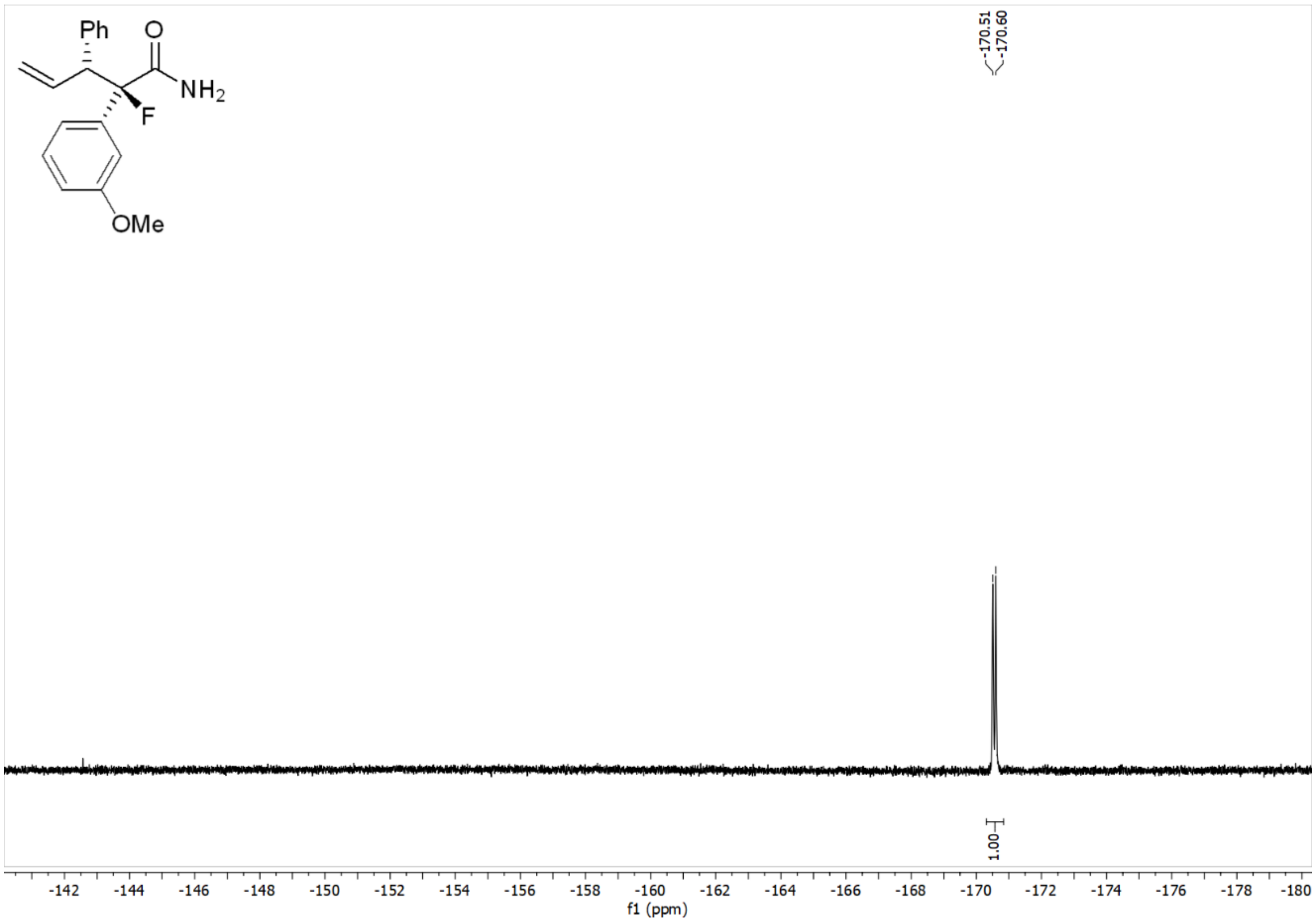

Ph
O
NH2
F
OMe
-170.51
-170.60
1.00
-142
-144
-146
-148
-150
-152
-154
-156
-158
-160
-162
-164
-166
-168
-170
-172
-174
-176
-178
-180
f1 (ppm)

**(2*R*,3*S*)-2-Fluoro-2-(3-methoxyphenyl)-3-phenylpent-4-enamide (*R*,*S*)-5:**

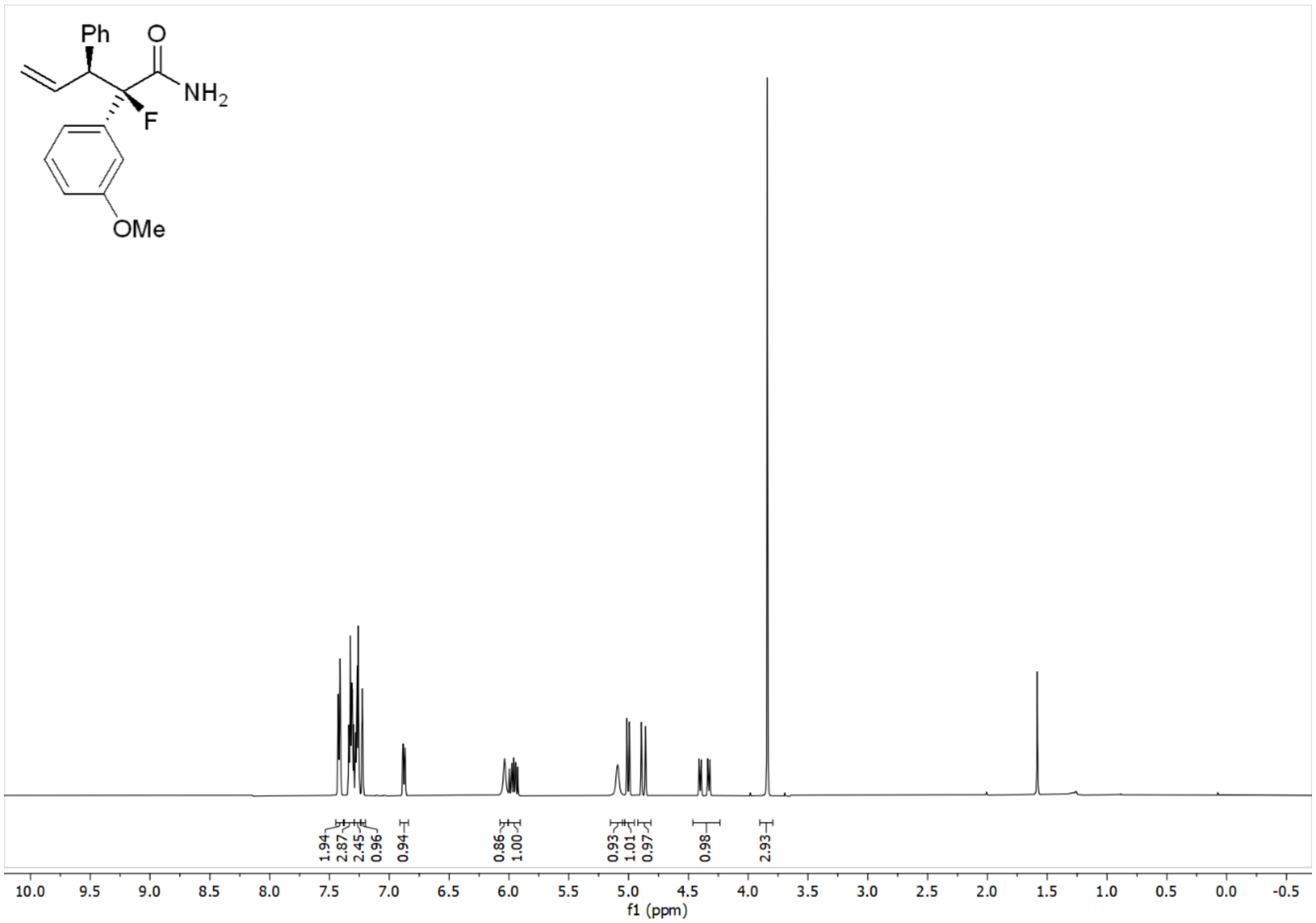


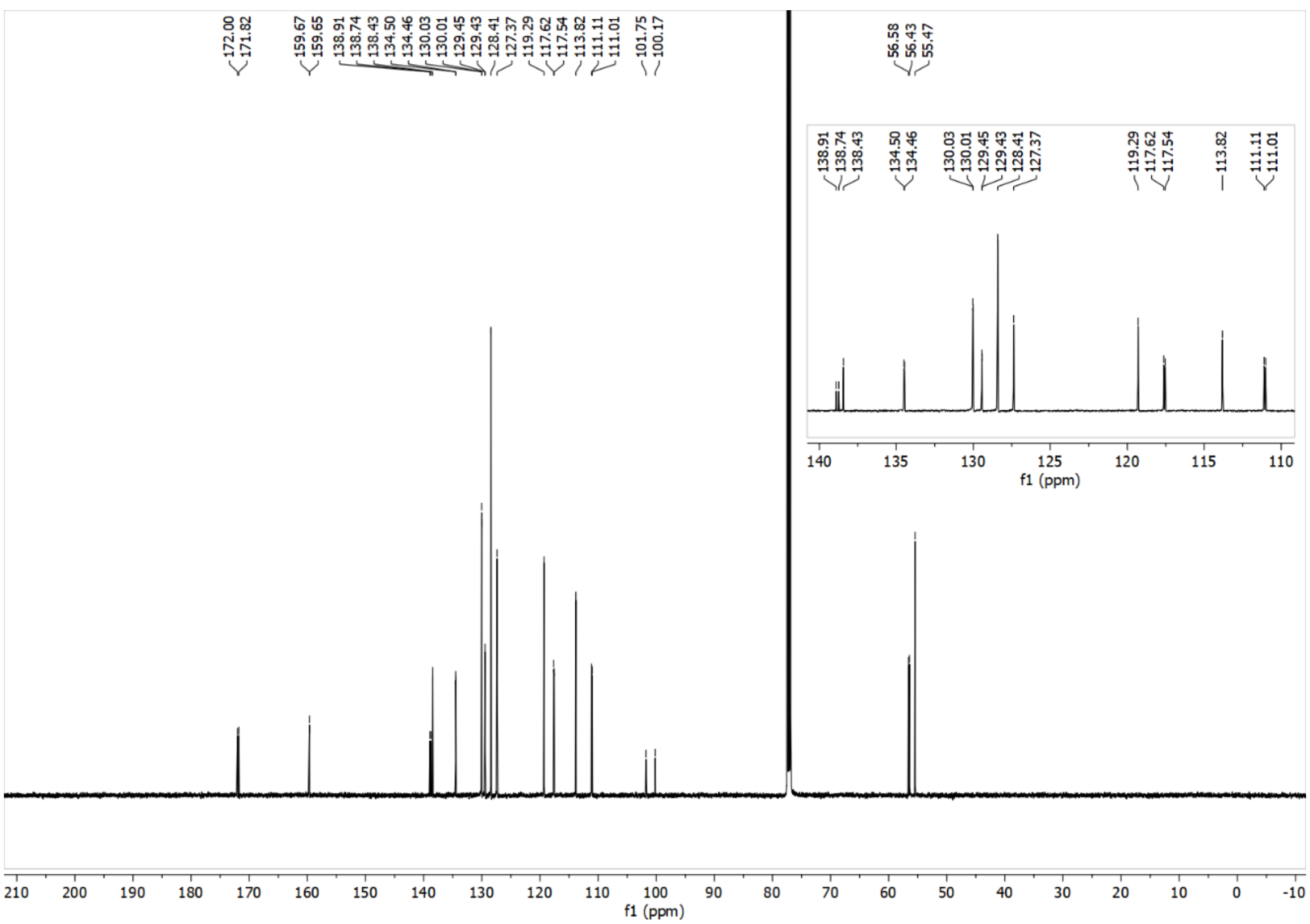

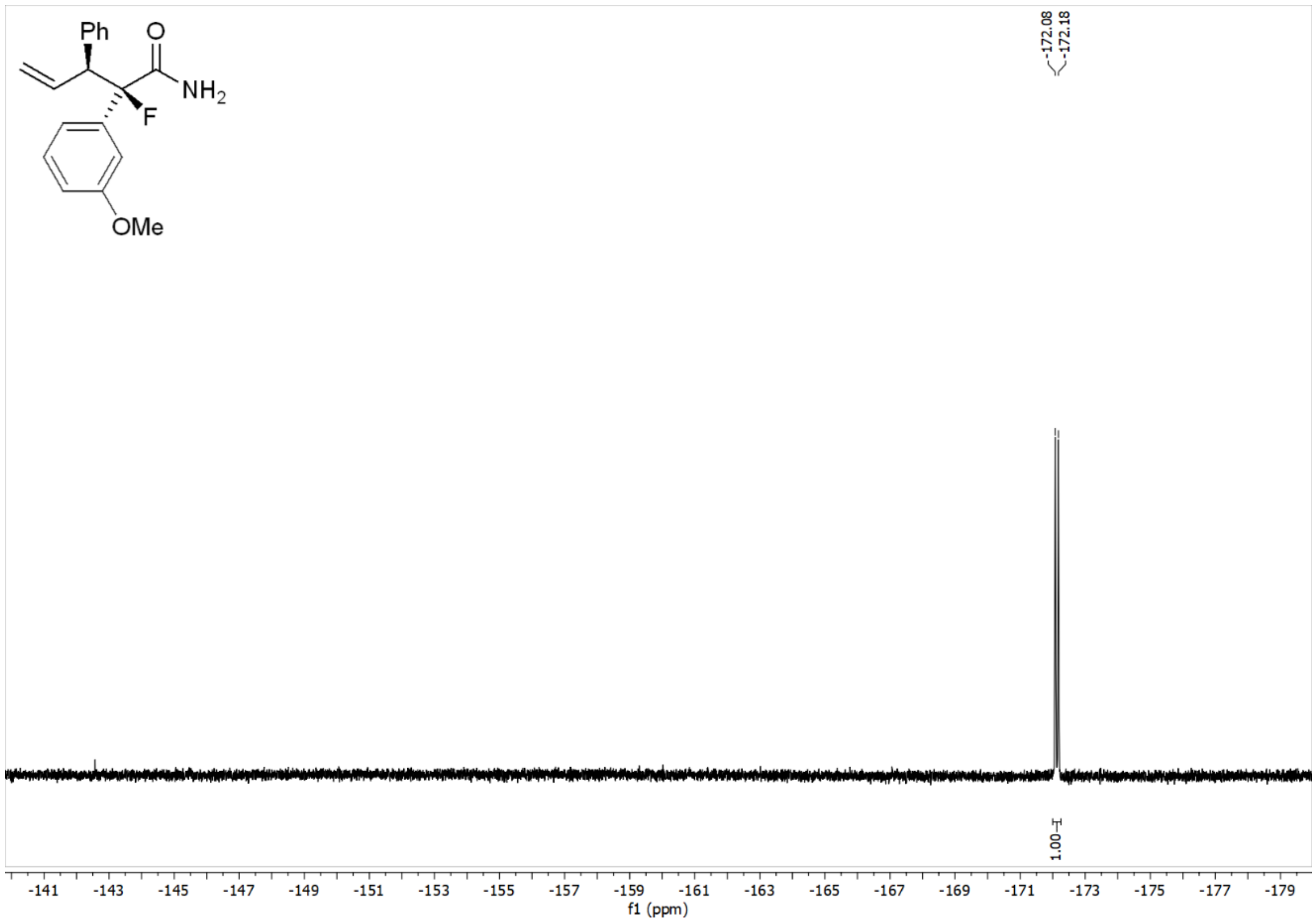

Ph
O
NH2
F
OMe
-172.08
-172.18
1.00
-141
-143
-145
-147
-149
-151
-153
-155
-157
-159
-161
-163
-165
-167
-169
-171
-173
-175
-177
-179
f1 (ppm)

**Perfluorophenyl (2*R*,3*R*)-2-(3-chlorophenyl)-2-fluoro-3-phenylpent-4-enoate (*R*,*R*)-6:**

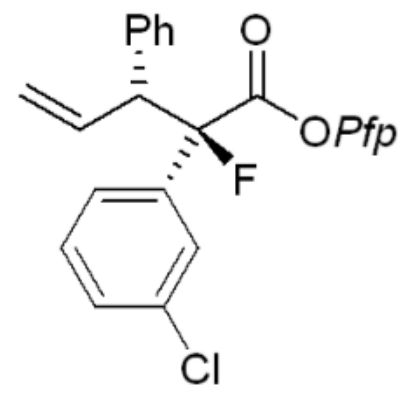


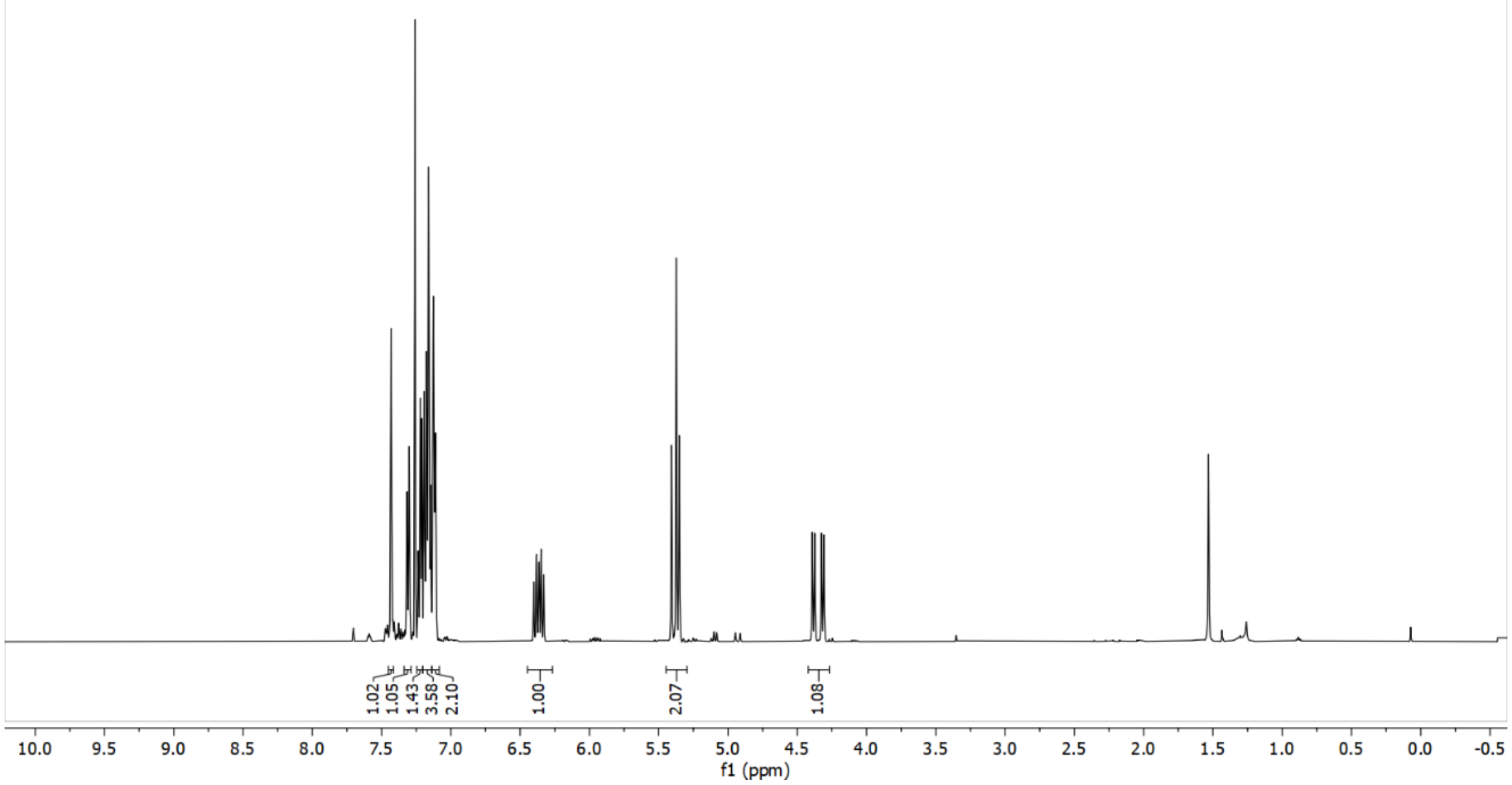


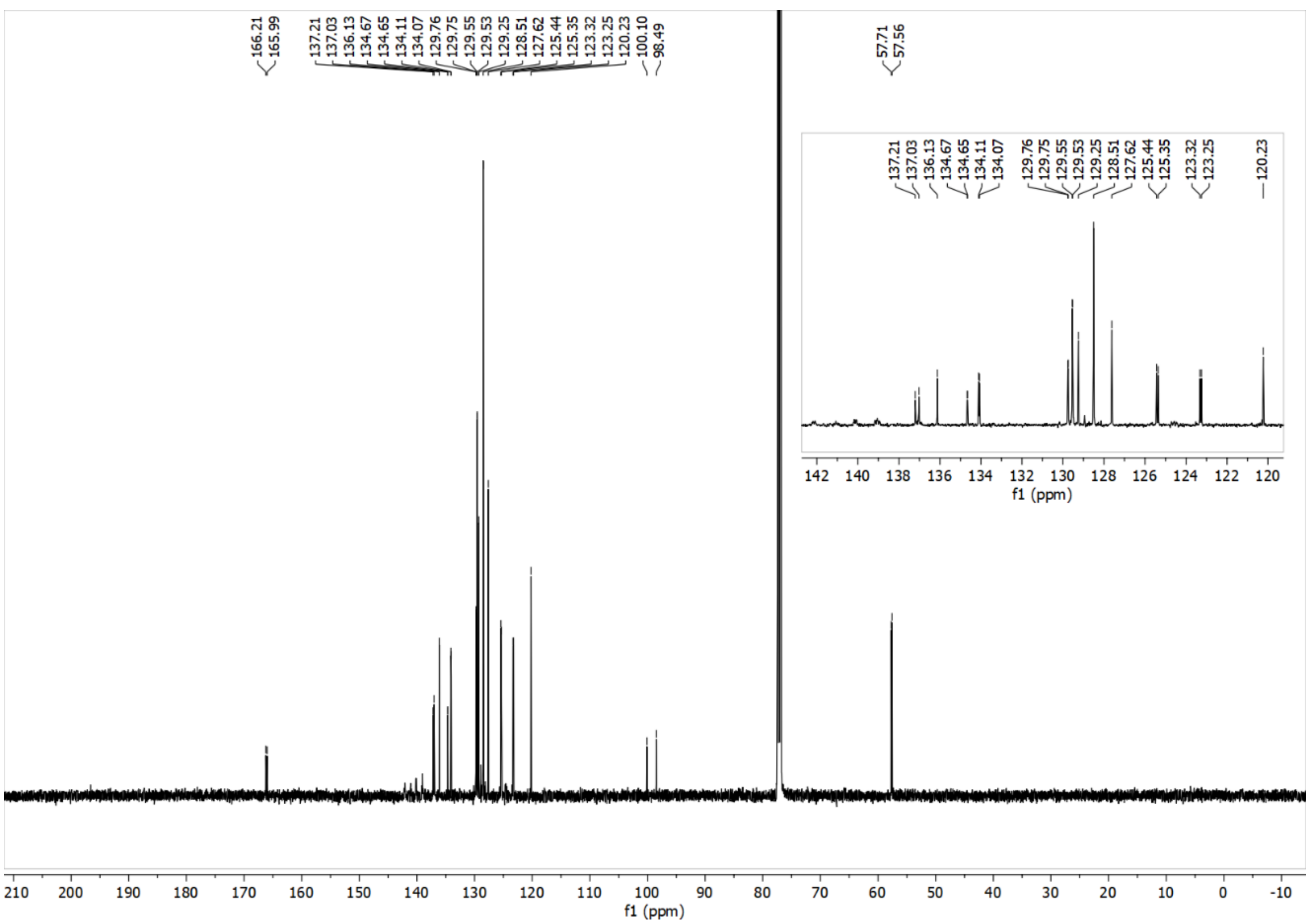

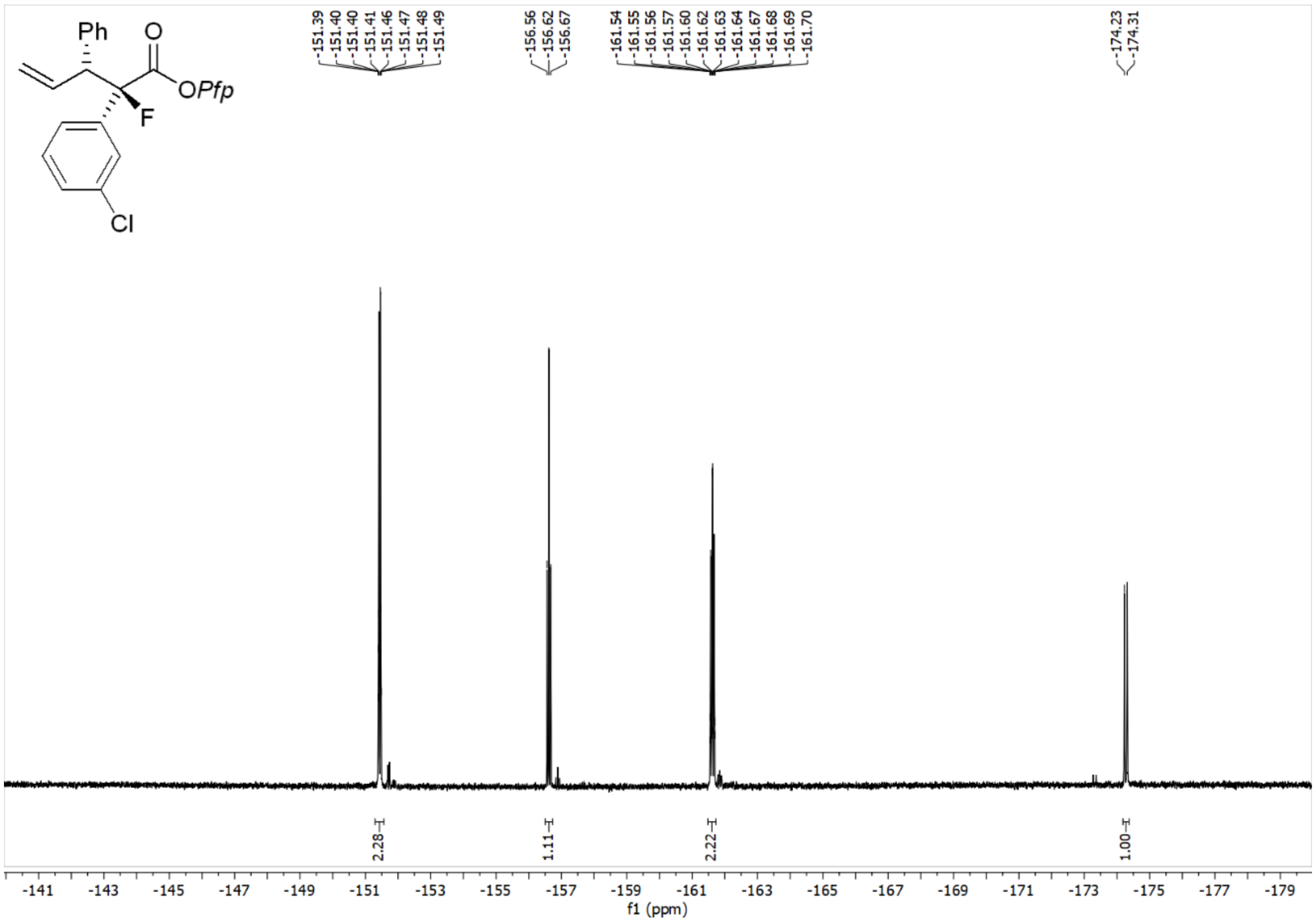
Ph
O
OPfp
F
Cl
-151.39
-151.40
-151.40
-151.41
-151.46
-151.47
-151.48
-151.49
-156.56
-156.62
-156.67
-161.54
-161.55
-161.56
-161.57
-161.60
-161.62
-161.63
-161.64
-161.67
-161.68
-161.69
-161.70
-174.23
-174.31
2.28
1.11
2.22
1.00
-141 -143 -145 -147 -149 -151 -153 -155 -157 -159 -161 -163 -165 -167 -169 -171 -173 -175 -177 -179
f1 (ppm)

**Perfluorophenyl (2*R*,3*S*)-2-(3-chlorophenyl)-2-fluoro-3-phenylpent-4-enoate (*R*,*S*)-6:**

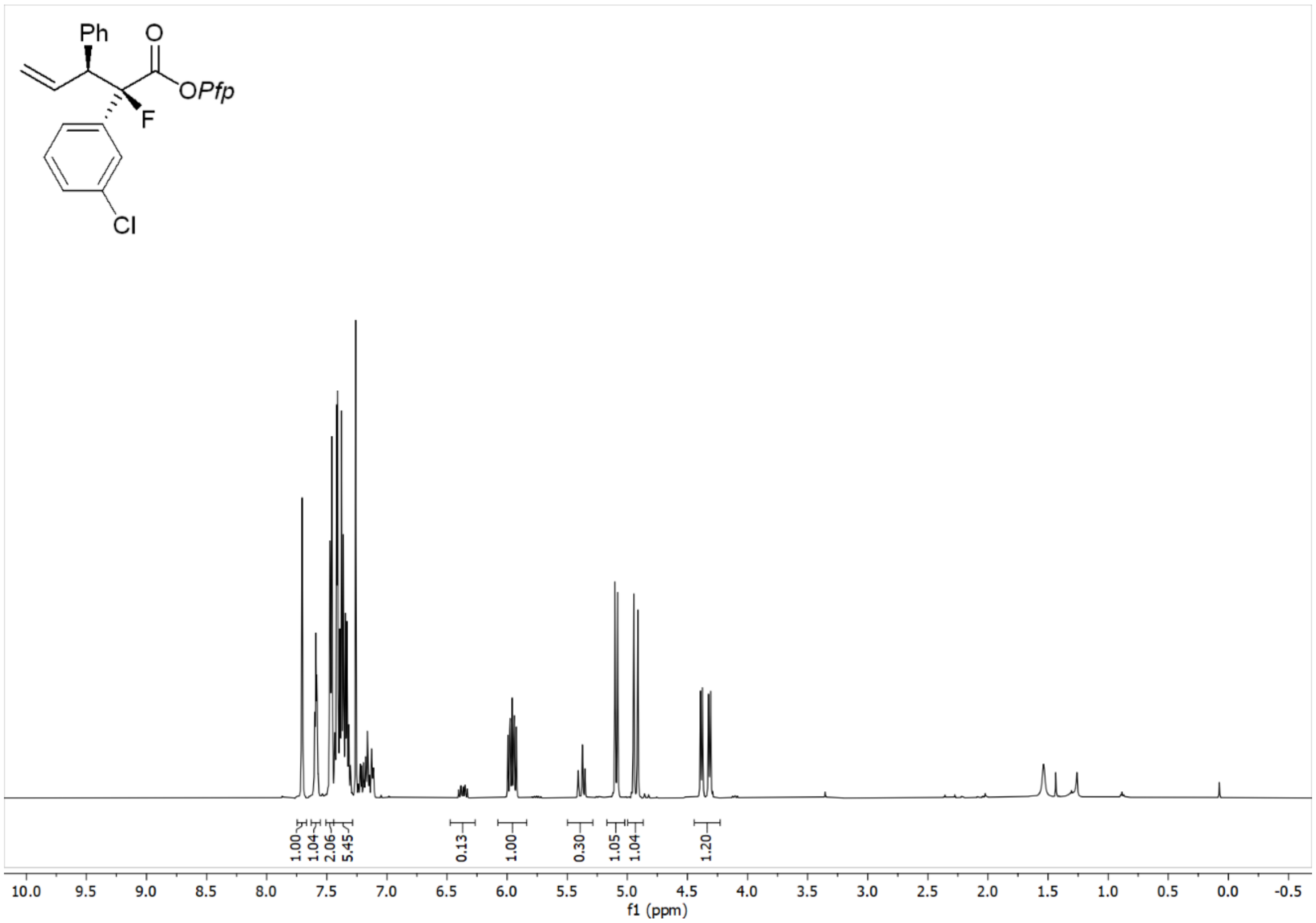


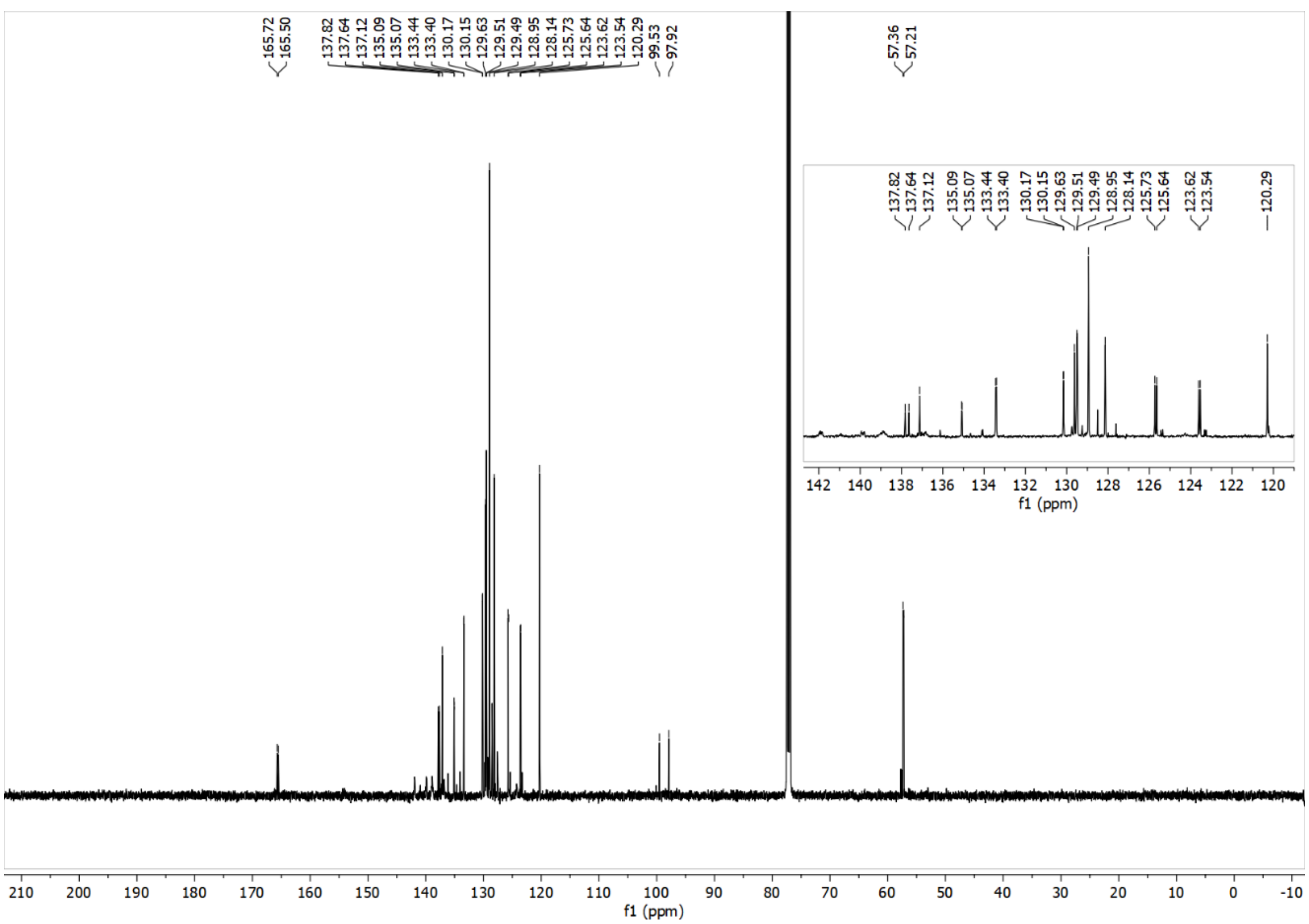

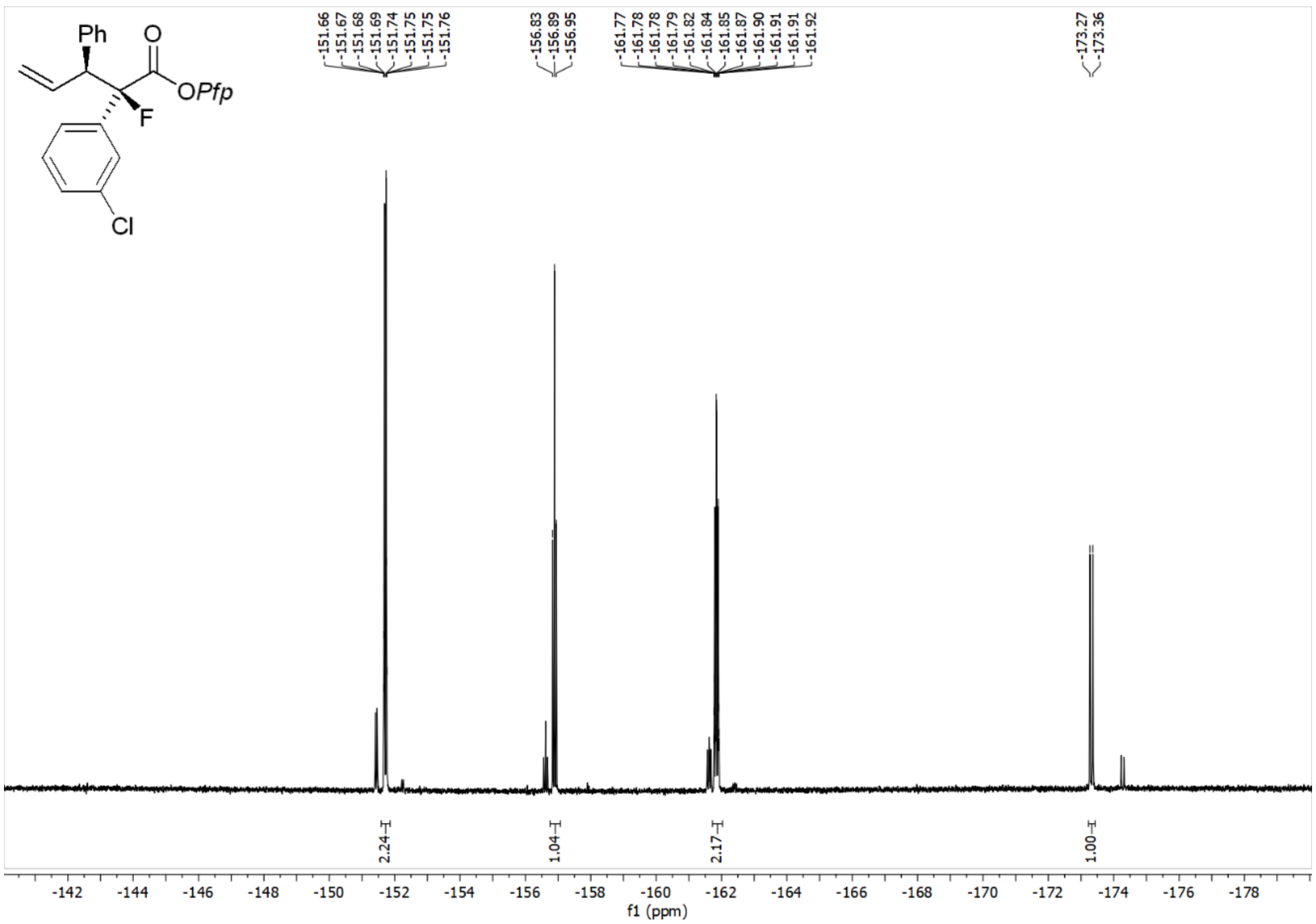
Ph
O
OPfp
F
Cl
-151.66
-151.67
-151.68
-151.69
-151.74
-151.75
-151.75
-151.76
-156.83
-156.89
-156.95
-161.77
-161.78
-161.78
-161.79
-161.82
-161.84
-161.85
-161.87
-161.90
-161.91
-161.91
-161.92
-173.27
-173.36
2.24
1.04
2.17
1.00
-142
-144
-146
-148
-150
-152
-154
-156
-158
-160
-162
-164
-166
-168
-170
-172
-174
-176
-178
f1 (ppm)

**Perfluorophenyl (2*R*,3*R*)-2-fluoro-2-phenyl-3-(p-tolyl)pent-4-enoate (*R*,*R*)-16:**

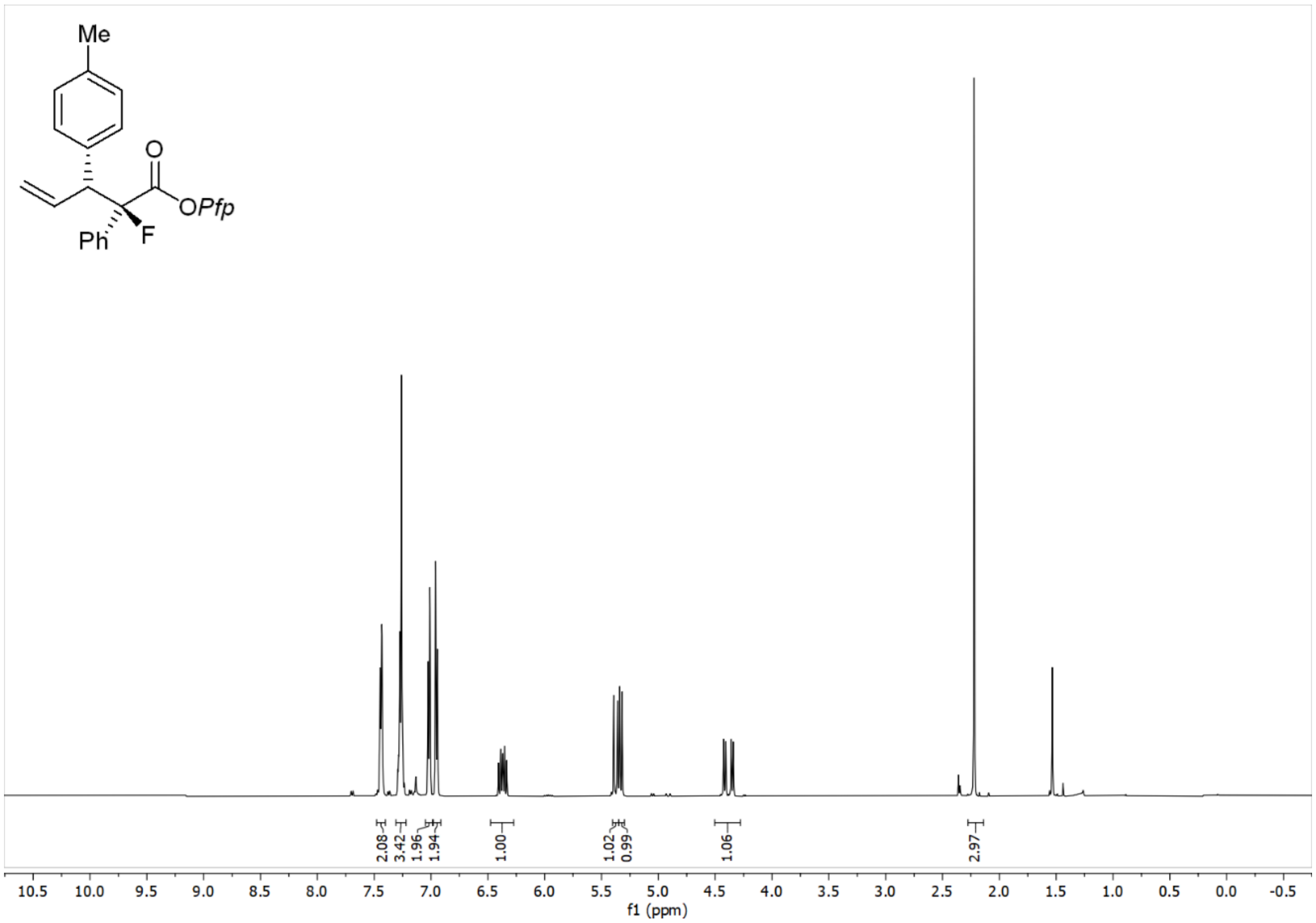


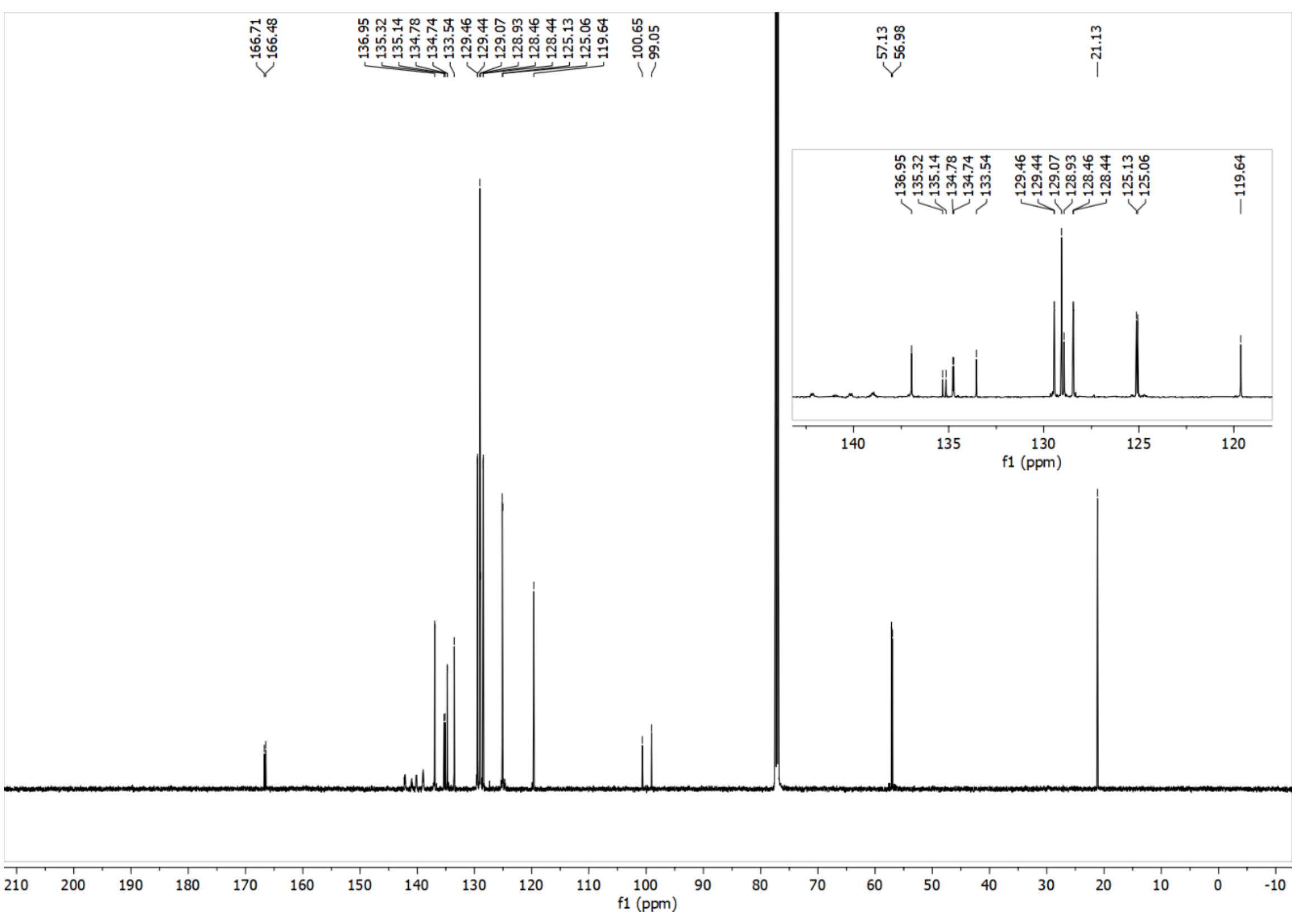

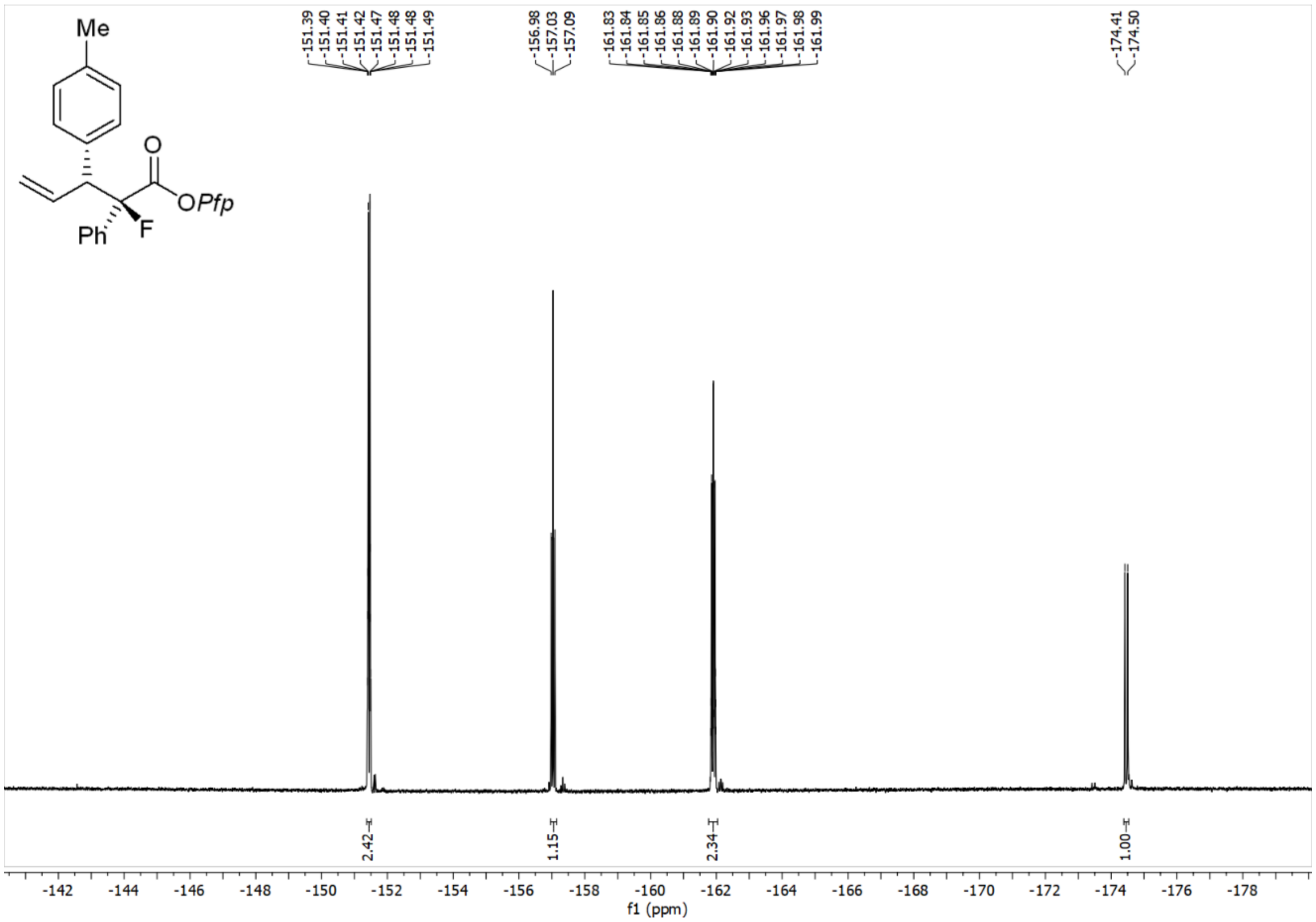

Me
O
OPfp
Ph
F
-151.39
-151.40
-151.41
-151.42
-151.47
-151.48
-151.48
-151.49
-156.98
-157.03
-157.09
-161.83
-161.84
-161.85
-161.86
-161.88
-161.89
-161.90
-161.92
-161.93
-161.96
-161.97
-161.98
-161.99
-174.41
-174.50
2.42
1.15
2.34
1.00
-142 -144 -146 -148 -150 -152 -154 -156 -158 -160 -162 -164 -166 -168 -170 -172 -174 -176 -178
f1 (ppm)

**Perfluorophenyl (2*R*,3*S*)-2-fluoro-2-phenyl-3-(p-tolyl)pent-4-enoate (*R*,*S*)-16:**

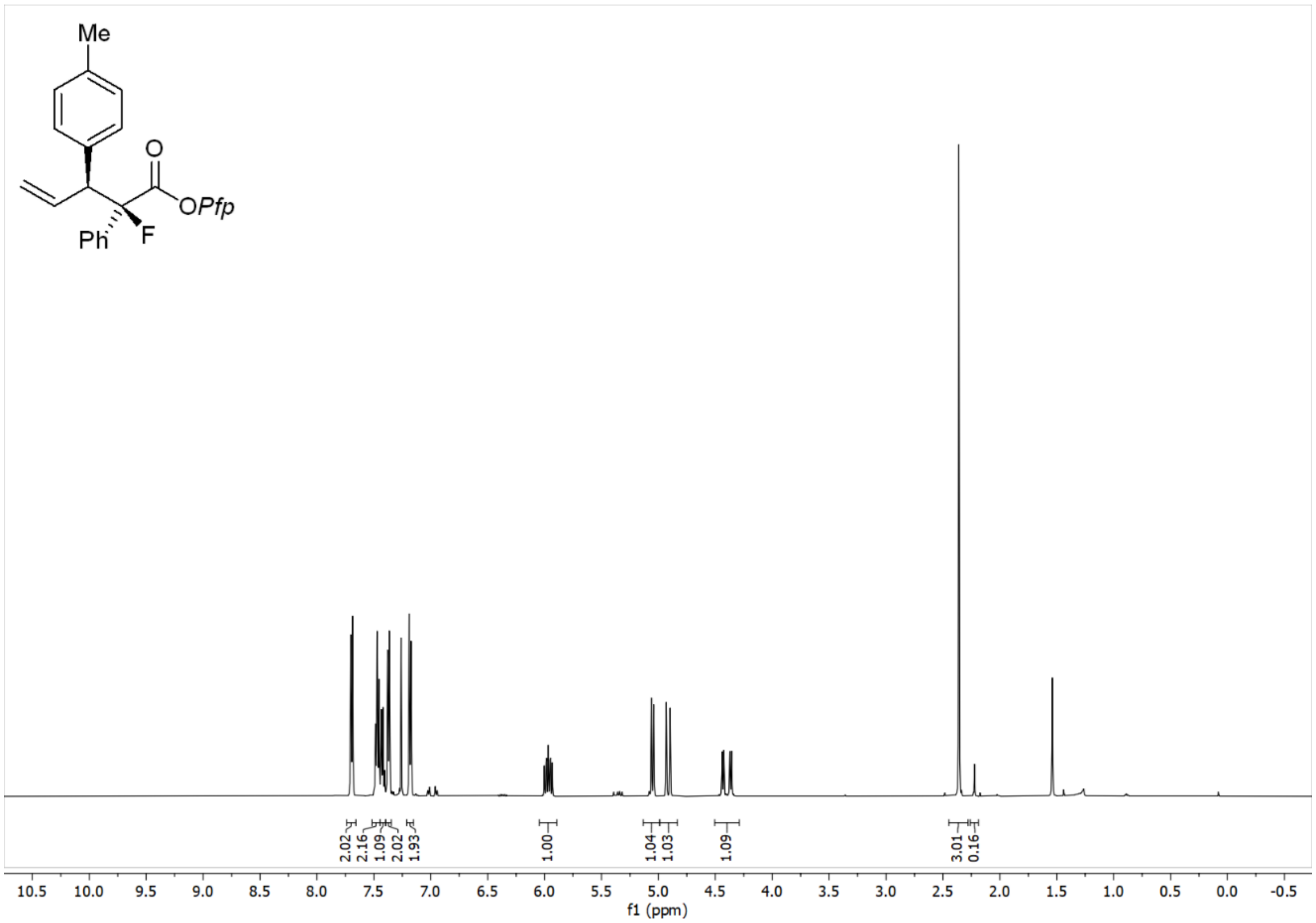


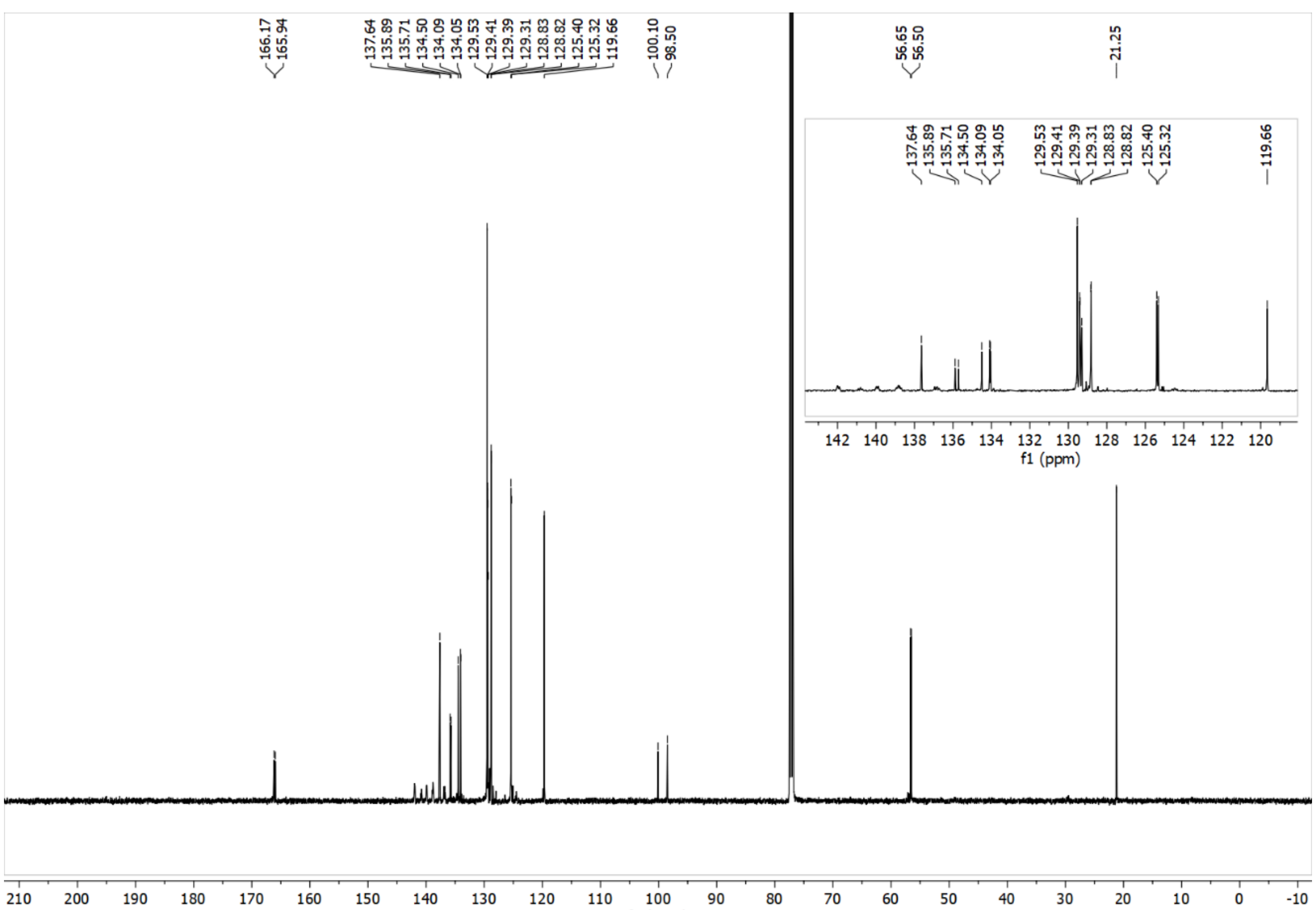

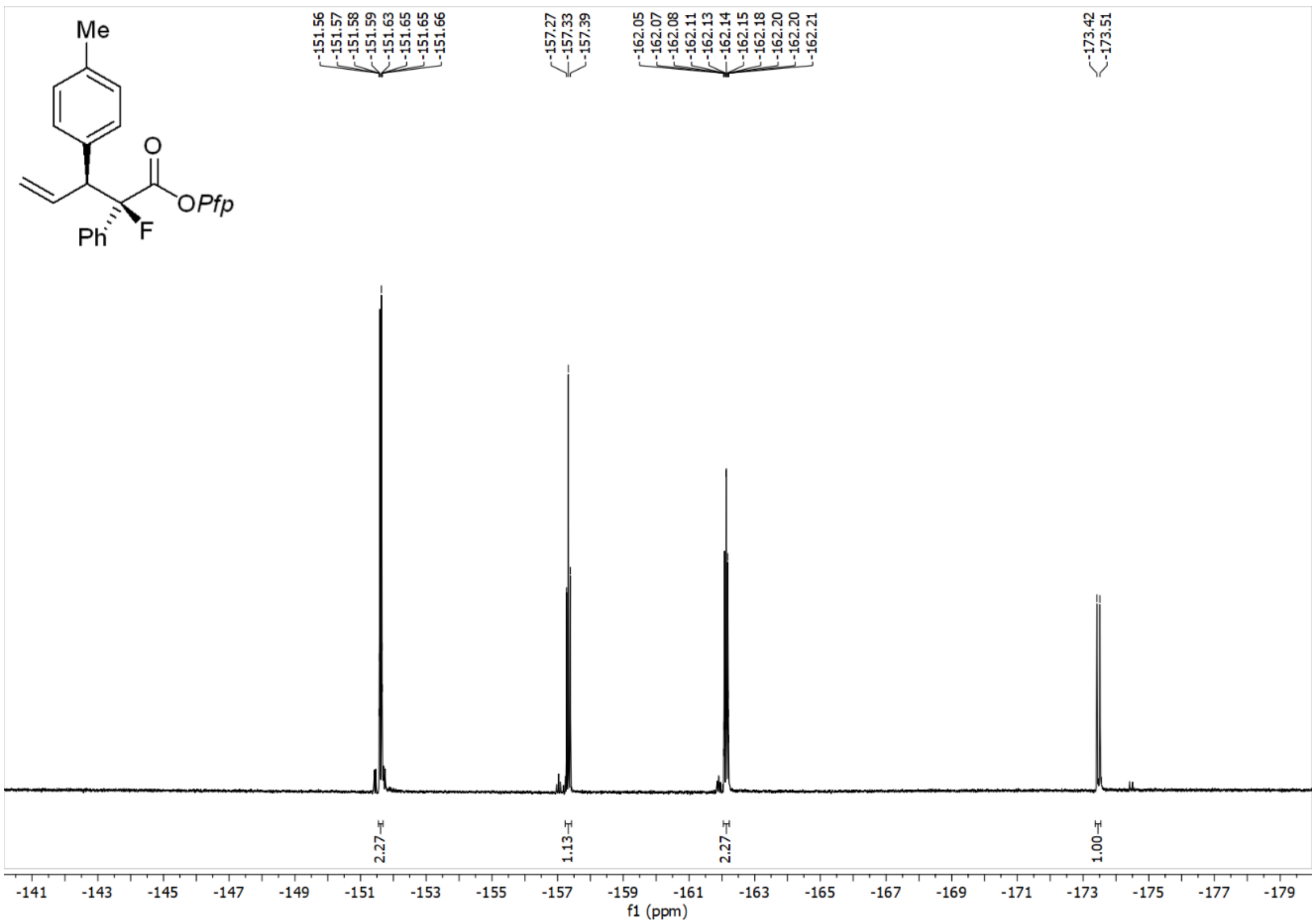

Me
O
OPfp
Ph
F
-151.56
-151.57
-151.58
-151.59
-151.63
-151.65
-151.65
-151.66
-157.27
-157.33
-157.39
-162.05
-162.07
-162.08
-162.11
-162.13
-162.14
-162.15
-162.18
-162.20
-162.20
-162.21
-173.42
-173.51
2.27
1.13
2.27
1.00
-141 -143 -145 -147 -149 -151 -153 -155 -157 -159 -161 -163 -165 -167 -169 -171 -173 -175 -177 -179
f1 (ppm)

**Perfluorophenyl (2*R*,3*R*)-2-fluoro-3-(4-fluorophenyl)-2-phenylpent-4-enoate (*R*,*R*)-17:**

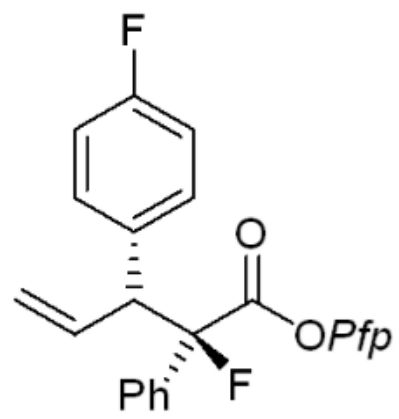


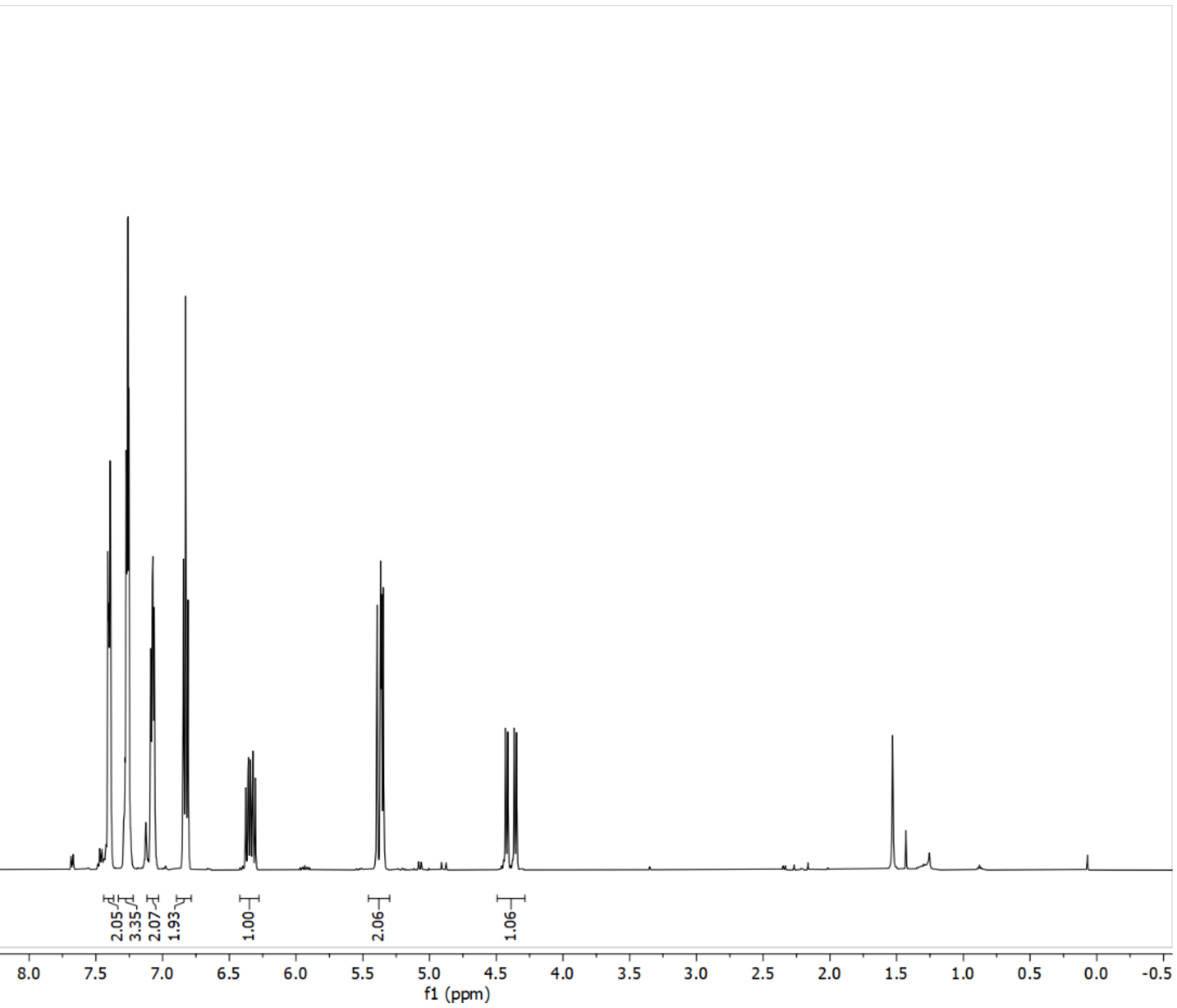


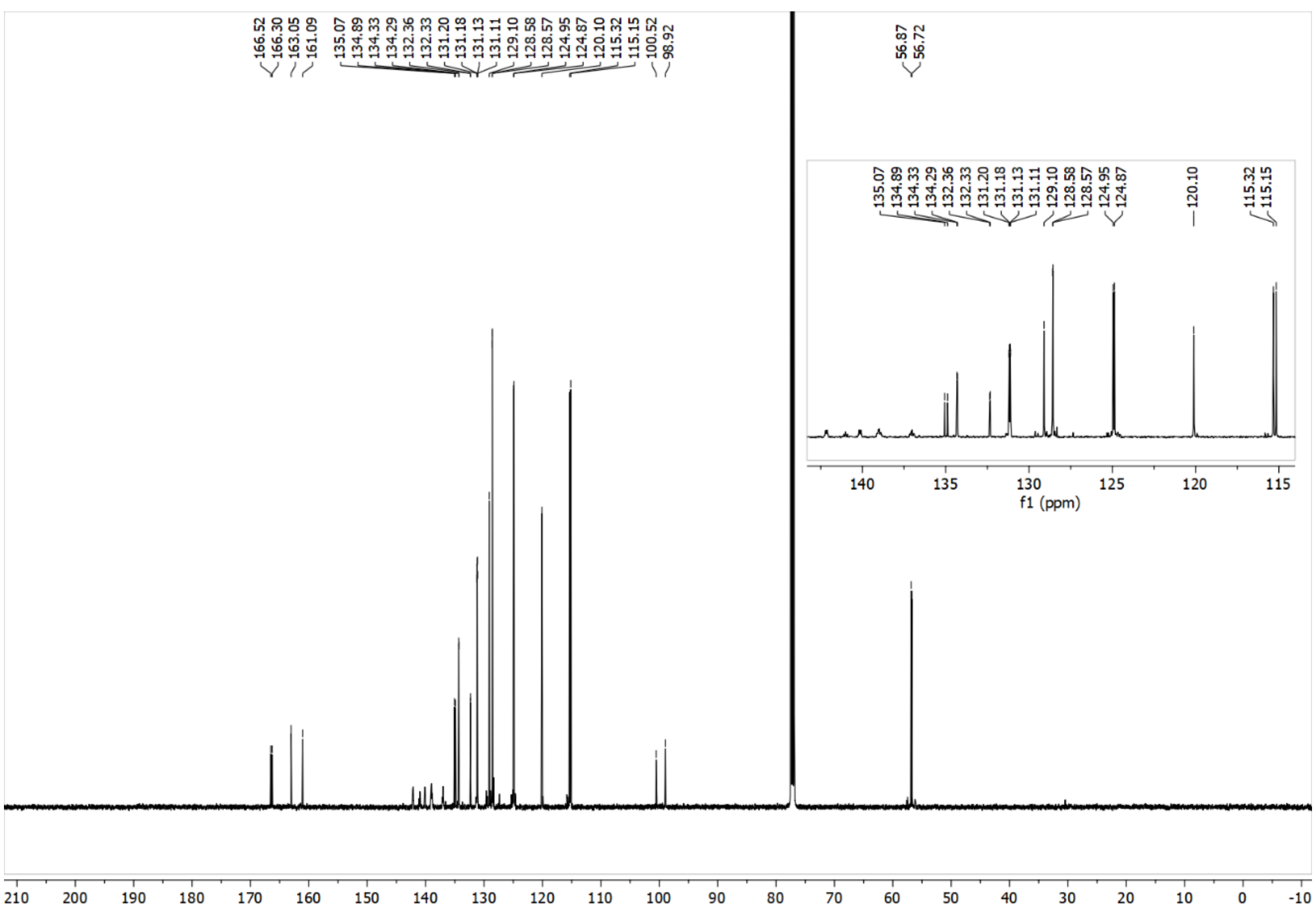

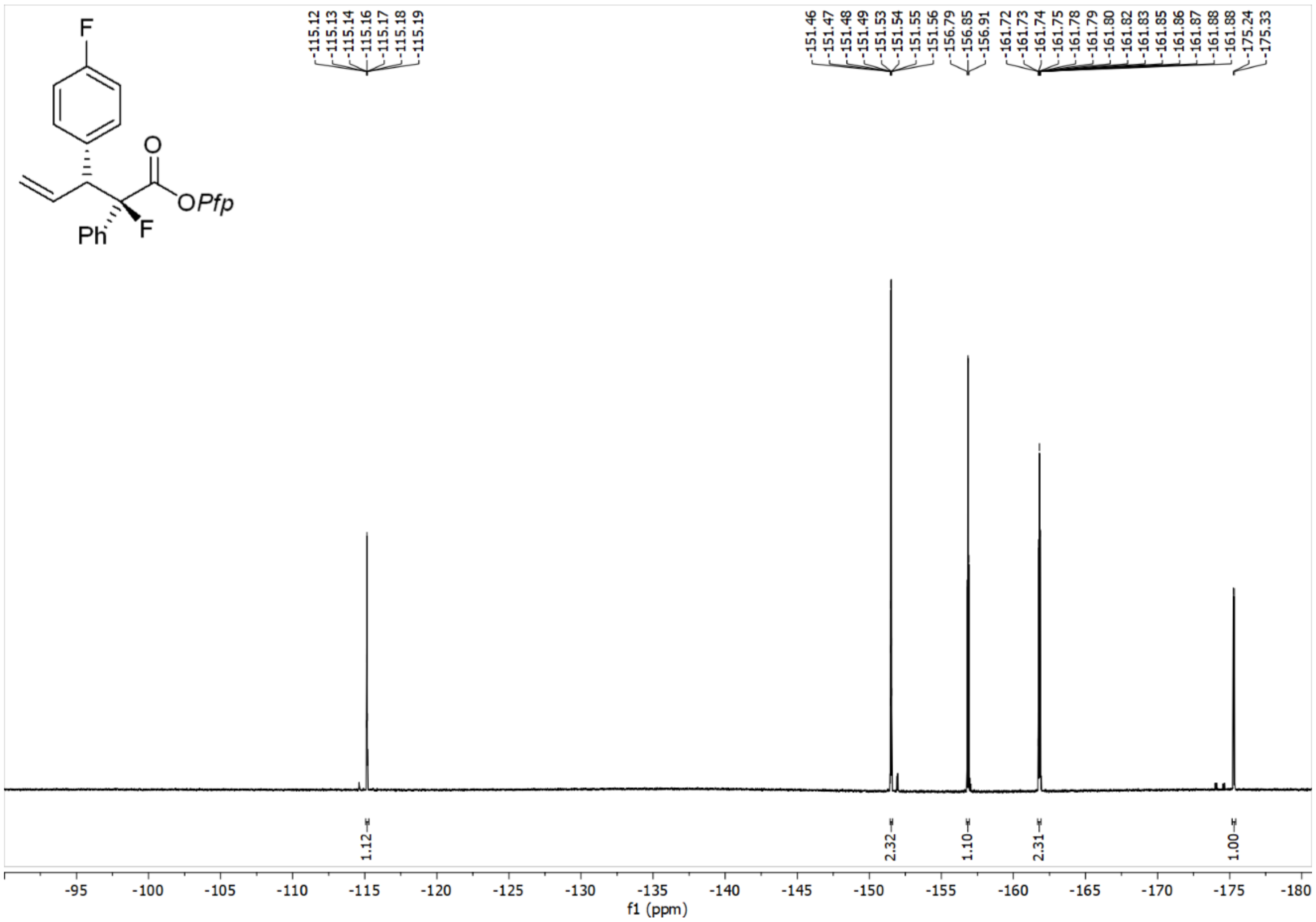

F
O
OPfp
Ph
F
-115.12
-115.13
-115.14
-115.16
-115.17
-115.18
-115.19
-151.46
-151.47
-151.48
-151.49
-151.53
-151.54
-151.55
-151.56
-156.79
-156.85
-156.91
-161.72
-161.73
-161.74
-161.75
-161.78
-161.79
-161.80
-161.82
-161.83
-161.85
-161.86
-161.87
-161.88
-161.88
-175.24
-175.33
1.12
2.32
1.10
2.31
1.00
-95 -100 -105 -110 -115 -120 -125 -130 -135 -140 -145 -150 -155 -160 -165 -170 -175 -180
f1 (ppm)

**Perfluorophenyl (2*R*,3*S*)-2-fluoro-3-(4-fluorophenyl)-2-phenylpent-4-enoate (*R*,*S*)-17:**

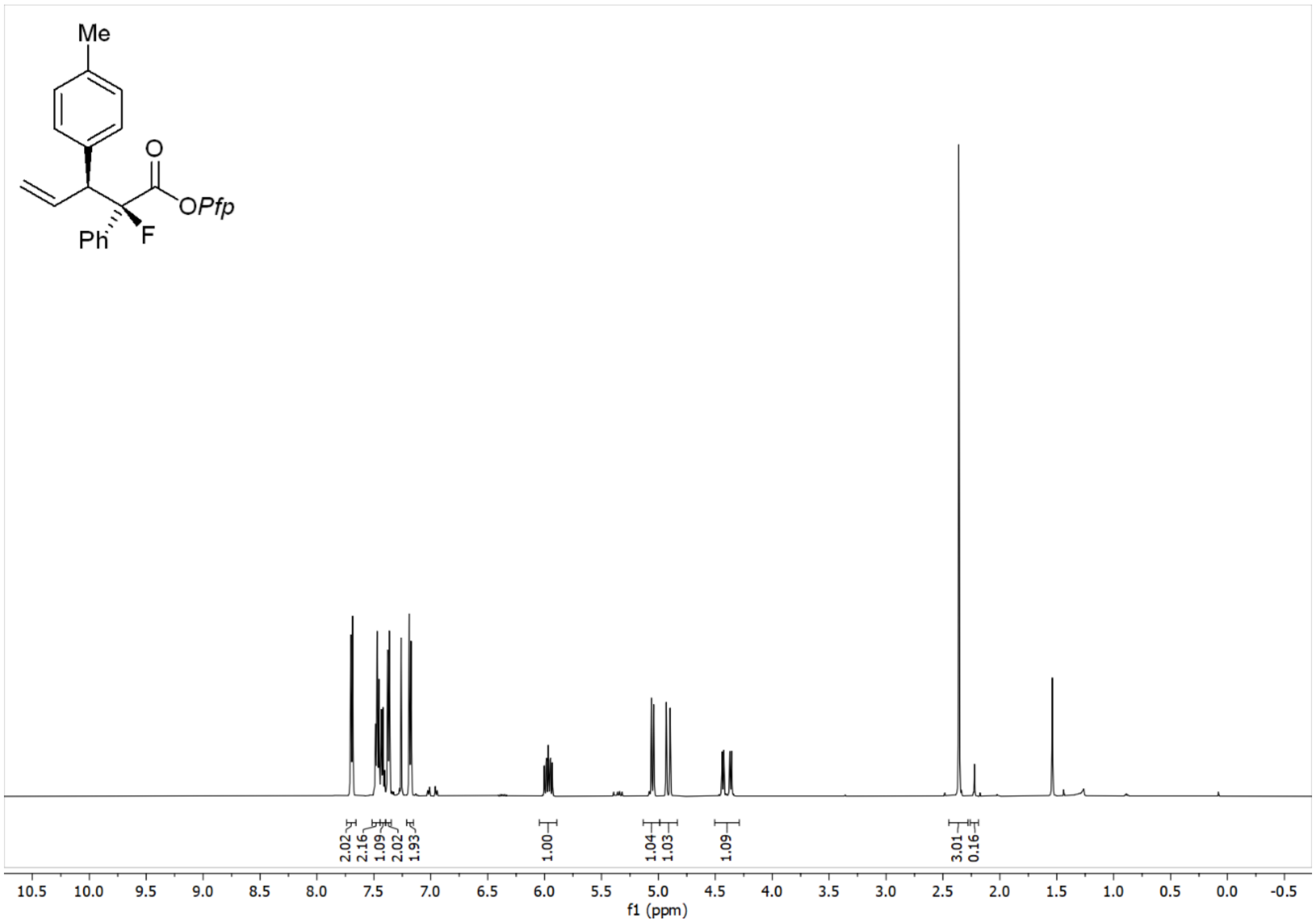


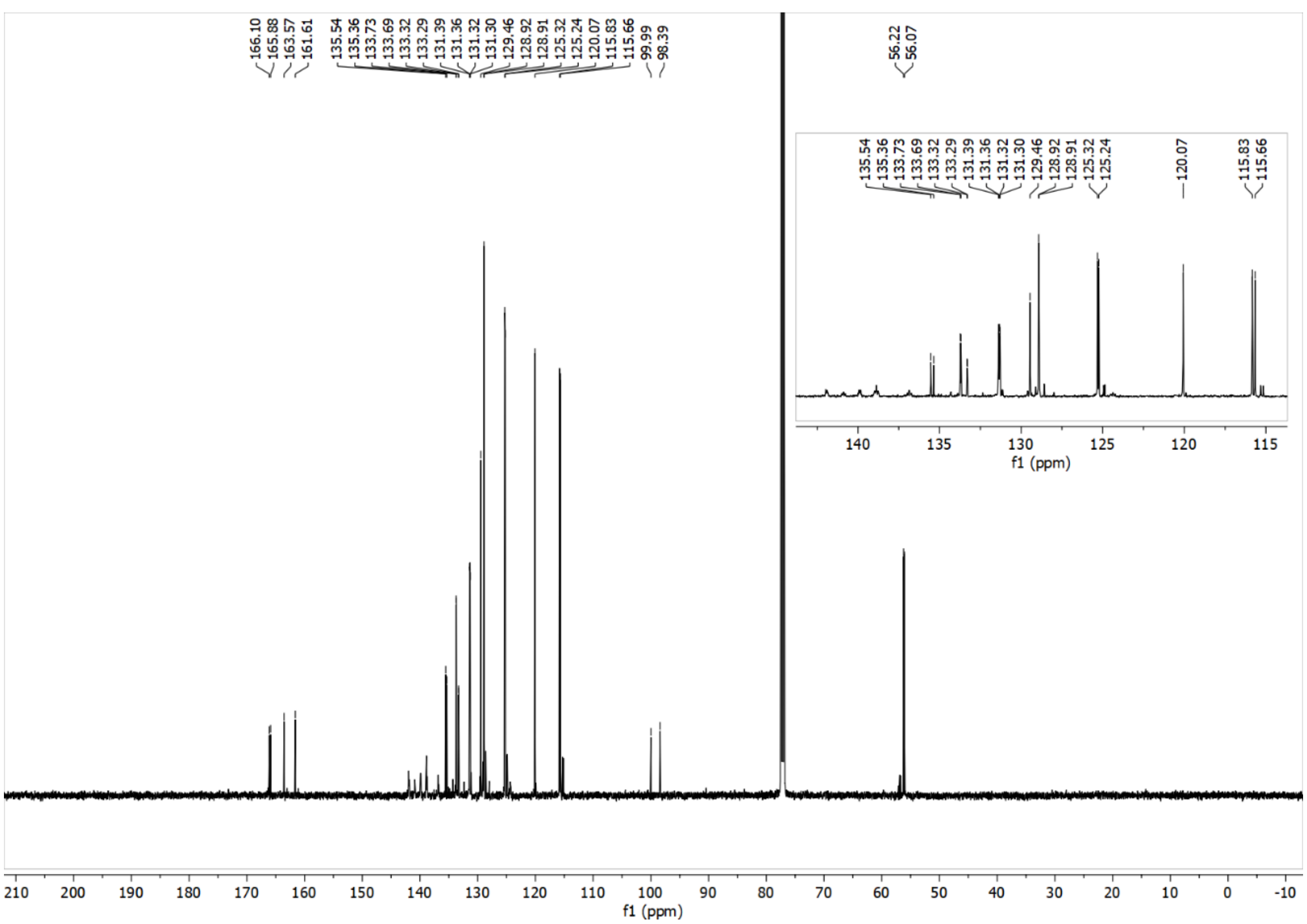

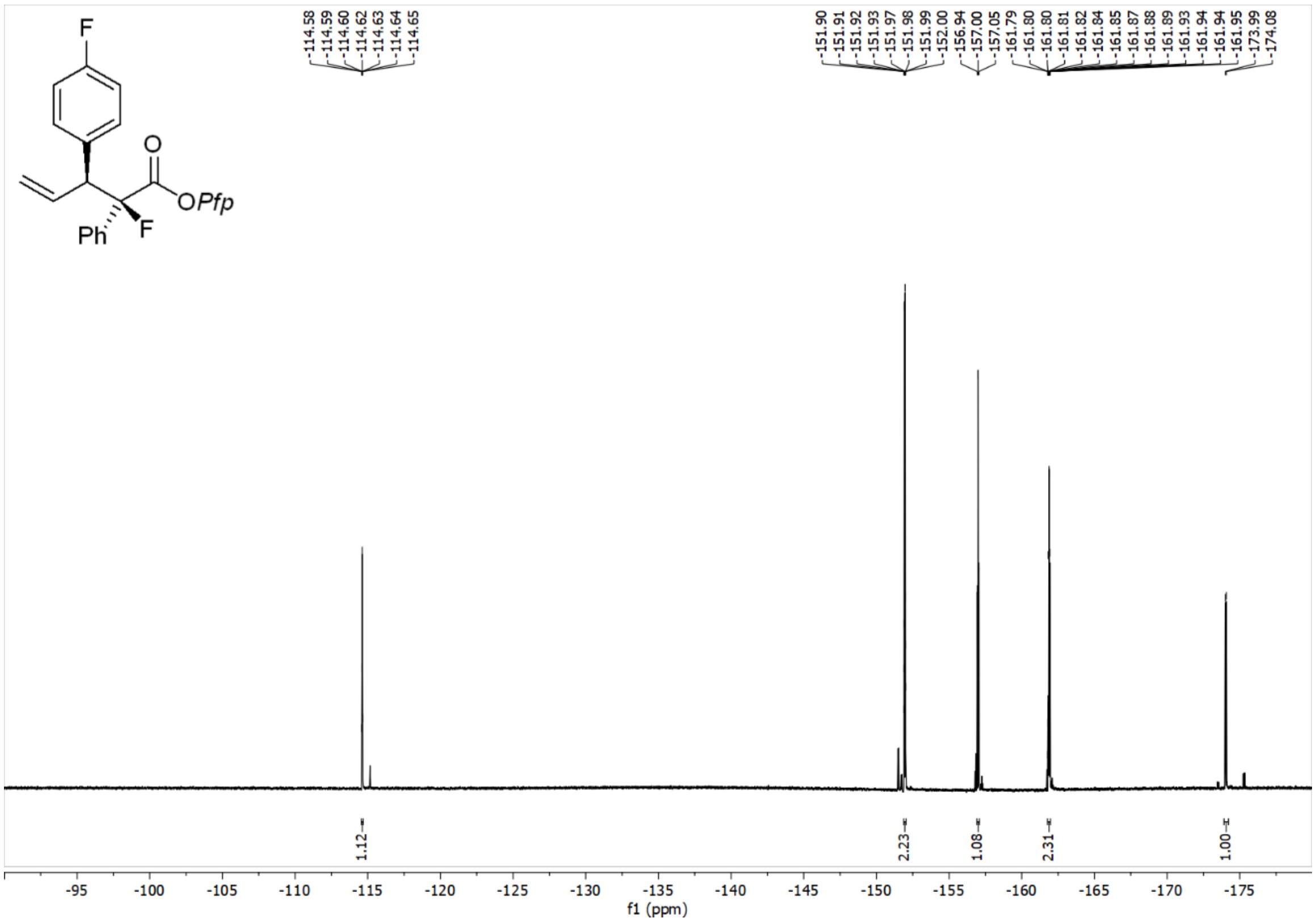

F
O
OPfp
Ph
F
-114.58
-114.59
-114.60
-114.62
-114.63
-114.64
-114.65
-151.90
-151.91
-151.92
-151.93
-151.97
-151.98
-151.99
-152.00
-156.94
-157.00
-157.05
-161.79
-161.80
-161.80
-161.81
-161.82
-161.84
-161.85
-161.87
-161.88
-161.89
-161.93
-161.94
-161.94
-161.95
-173.99
-174.08
1.12
2.23
1.08
2.31
1.00
-95
-100
-105
-110
-115
-120
-125
-130
-135
-140
-145
-150
-155
-160
-165
-170
-175
f1 (ppm)

**Perfluorophenyl (2*R*,3*R*)-3-(4-chlorophenyl)-2-fluoro-2-phenylpent-4-enoate (*R*,*R*)-18:**

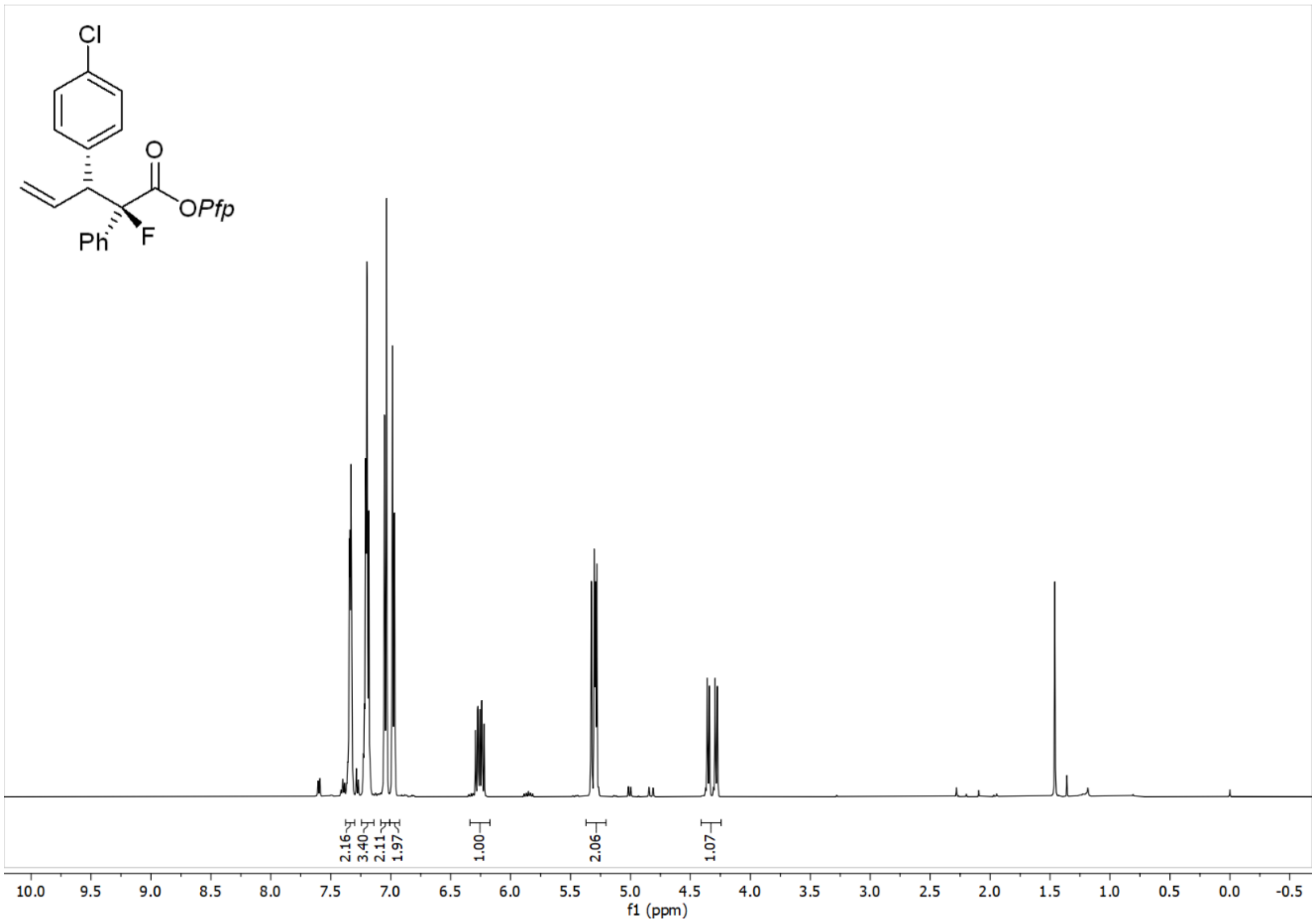


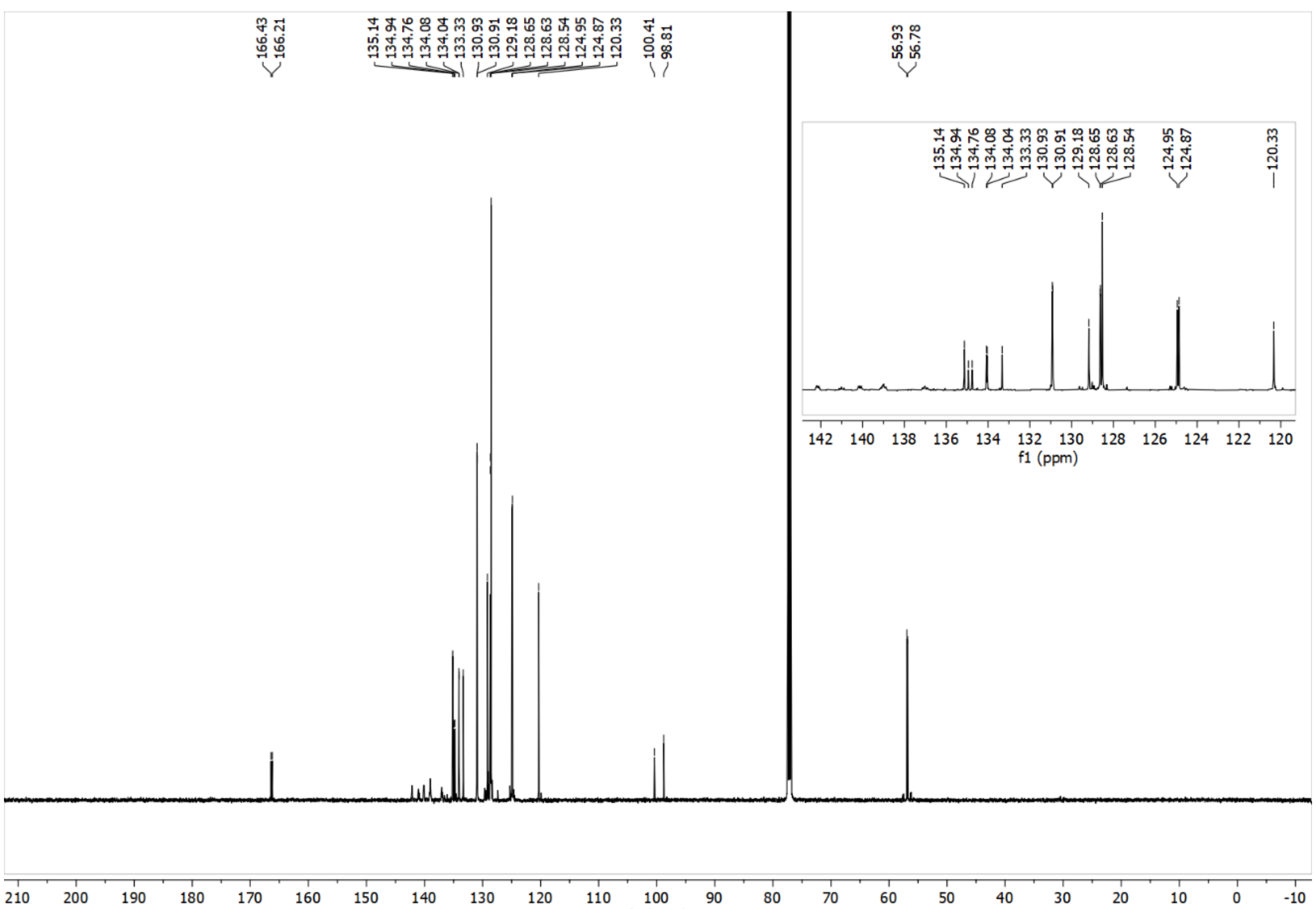

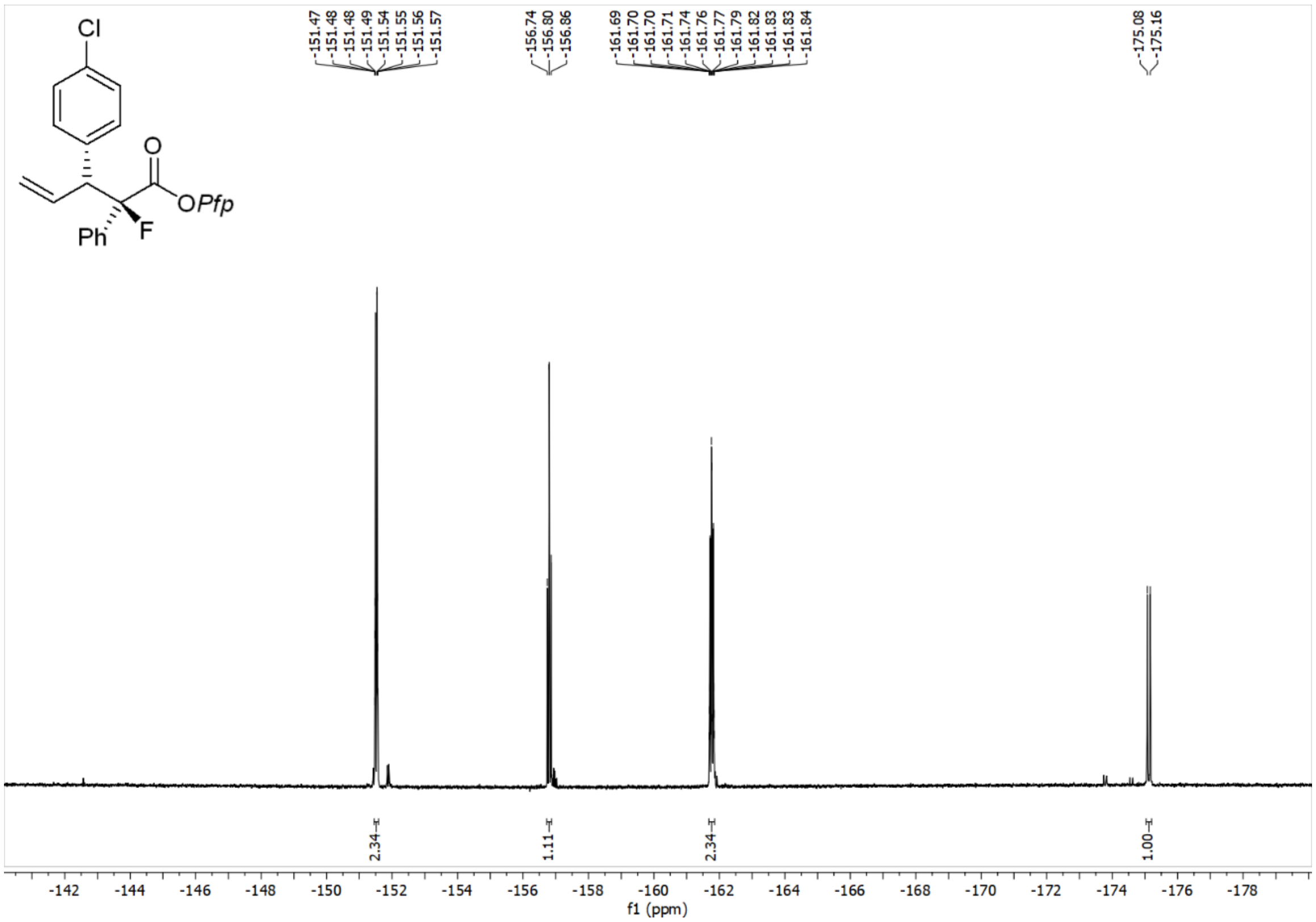

Cl
O
OPfp
Ph
F
-151.47
-151.48
-151.48
-151.49
-151.54
-151.55
-151.56
-151.57
-156.74
-156.80
-156.86
-161.69
-161.70
-161.70
-161.71
-161.74
-161.76
-161.77
-161.79
-161.82
-161.83
-161.83
-161.84
-175.08
-175.16
2.34
1.11
2.34
1.00
-142
-144
-146
-148
-150
-152
-154
-156
-158
-160
-162
-164
-166
-168
-170
-172
-174
-176
-178
f1 (ppm)

**Perfluorophenyl (2*R*,3*S*)-3-(4-chlorophenyl)-2-fluoro-2-phenylpent-4-enoate (*R*,*S*)-18:**

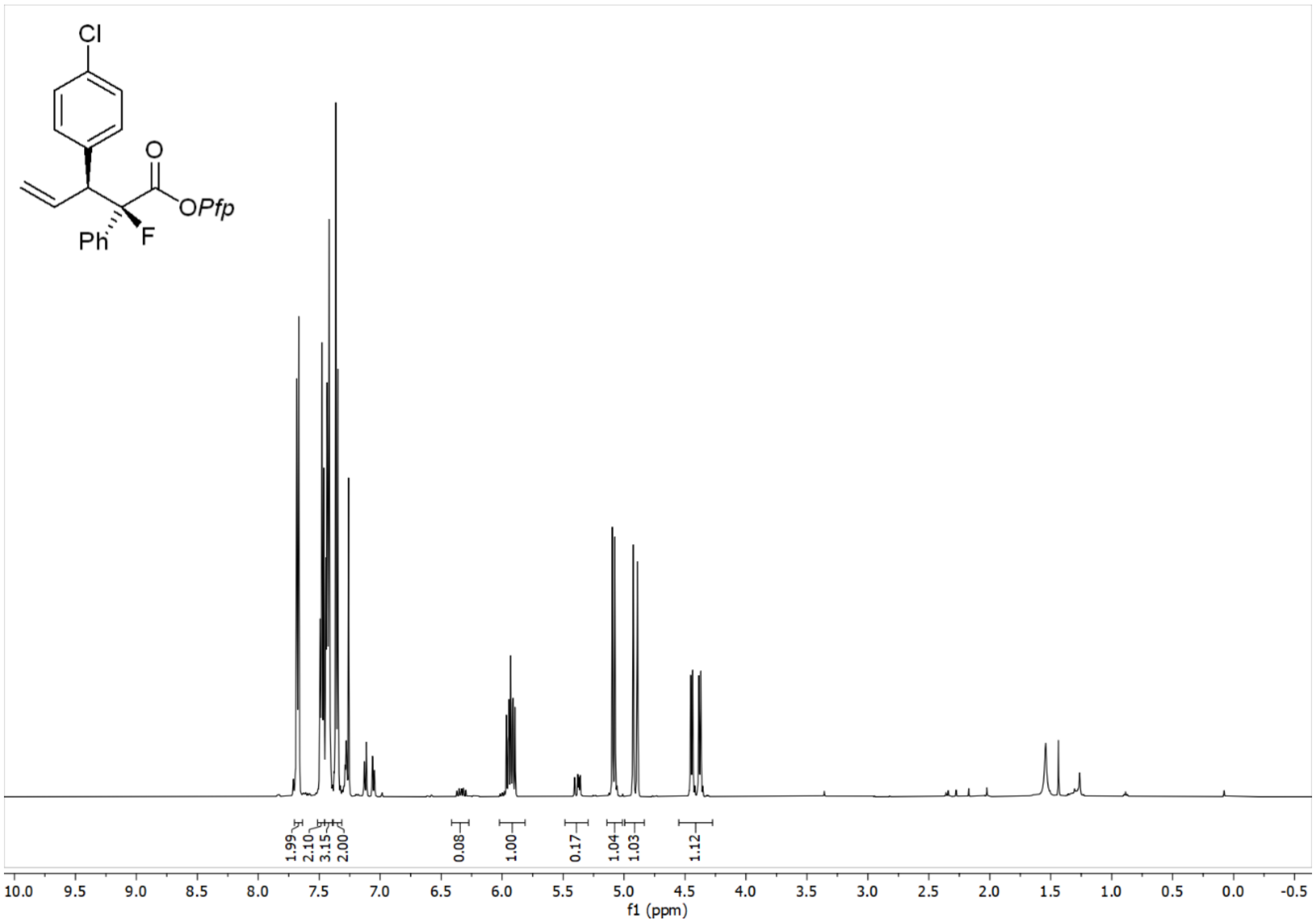


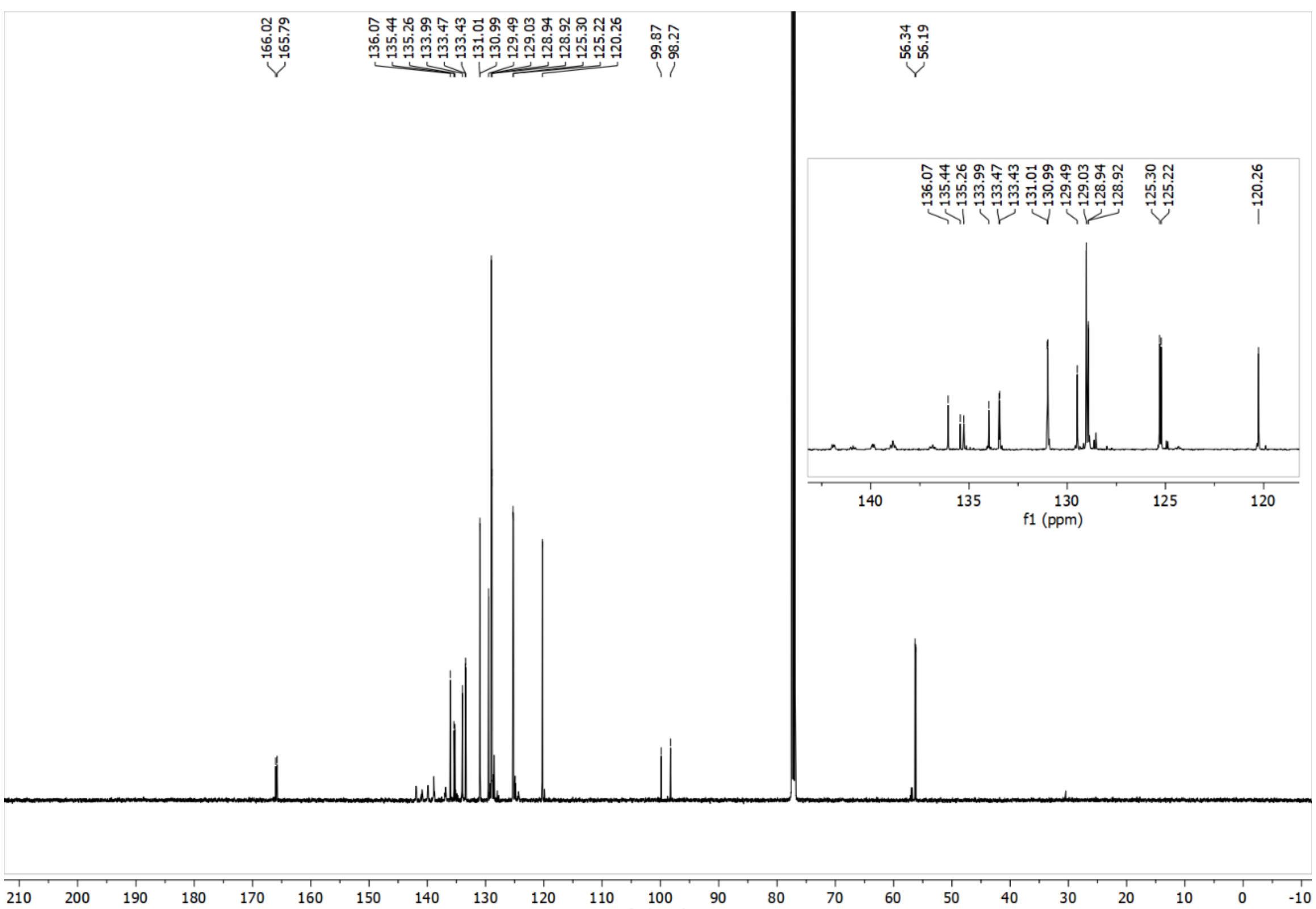

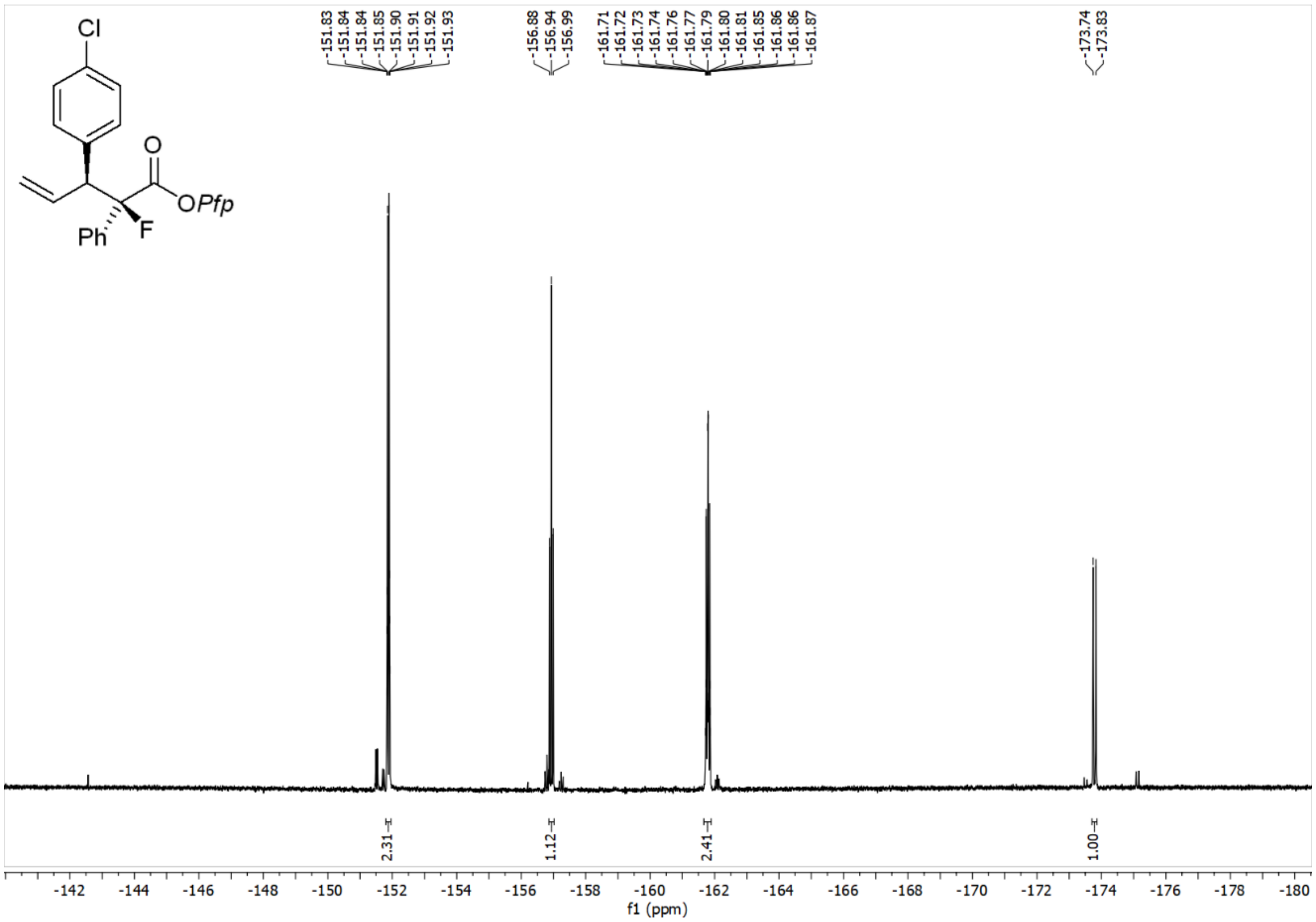

Cl
O
OPfp
Ph
F
-151.83
-151.84
-151.84
-151.85
-151.90
-151.91
-151.92
-151.93
-156.88
-156.94
-156.99
-161.71
-161.72
-161.73
-161.74
-161.76
-161.77
-161.79
-161.80
-161.81
-161.85
-161.86
-161.86
-161.87
-173.74
-173.83
2.31
1.12
2.41
1.00
-142 -144 -146 -148 -150 -152 -154 -156 -158 -160 -162 -164 -166 -168 -170 -172 -174 -176 -178 -180
f1 (ppm)

**Perfluorophenyl (2*R*,3*R*)-3-(4-bromophenyl)-2-fluoro-2-phenylpent-4-enoate (*R*,*R*)-19:**

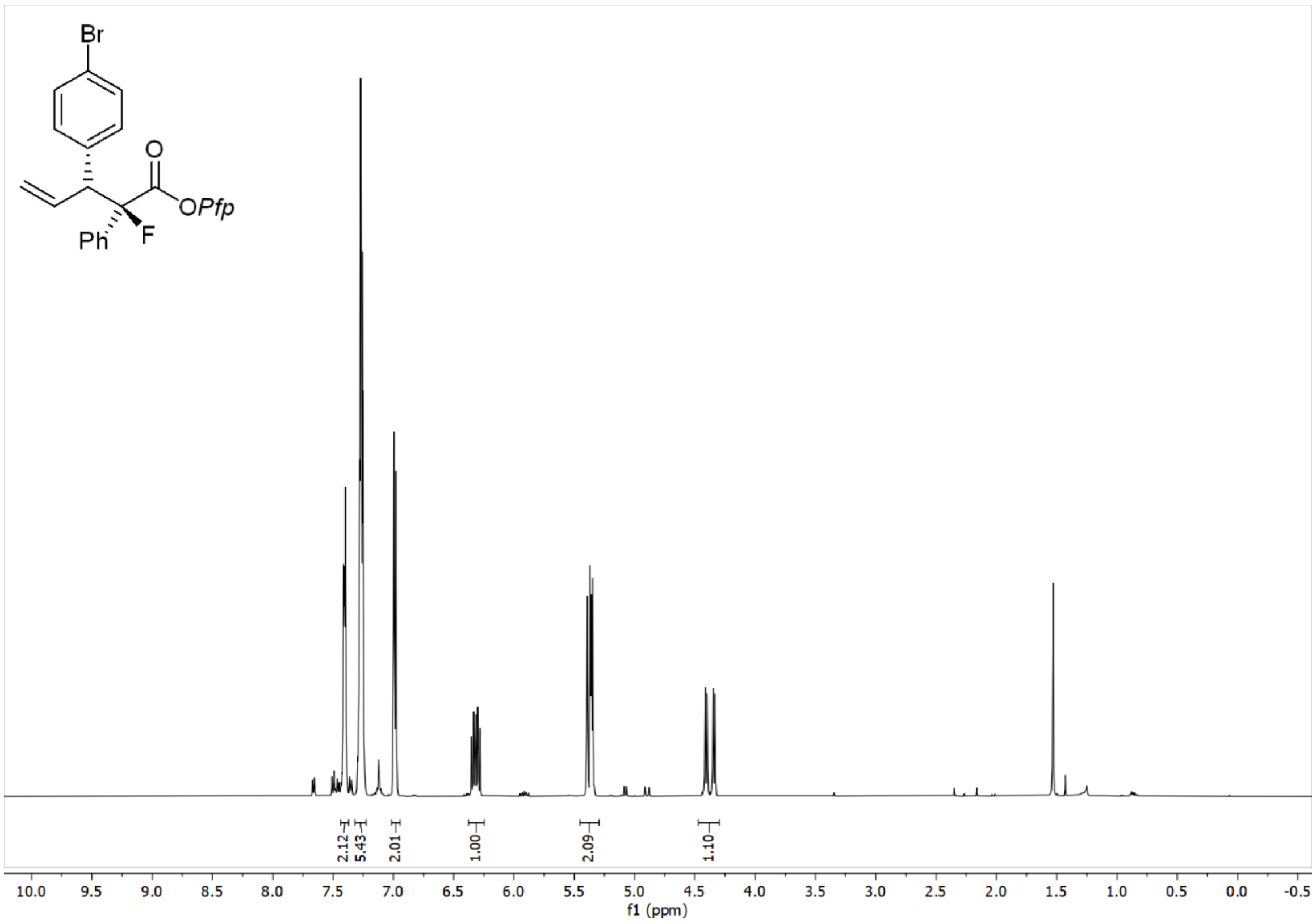


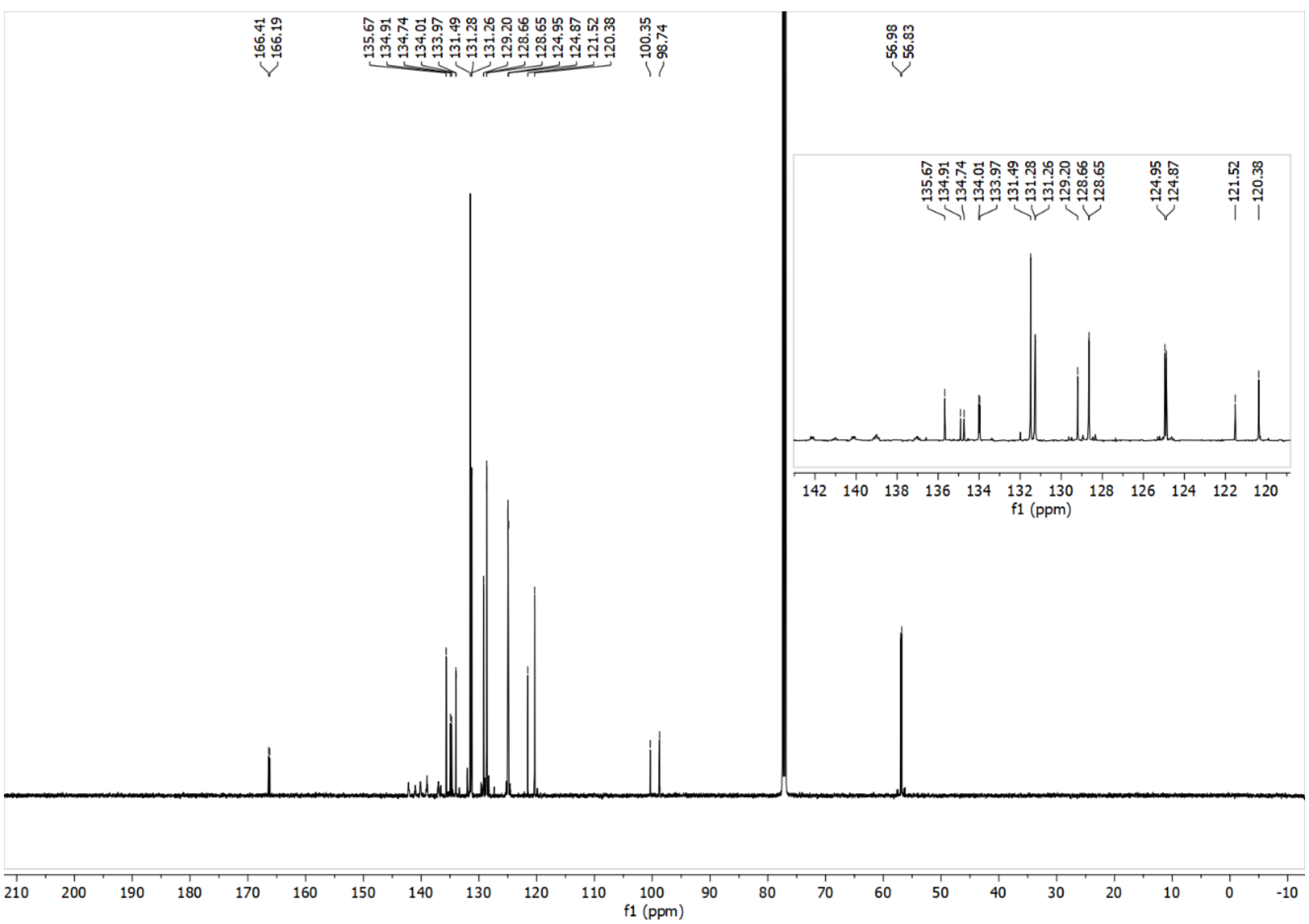

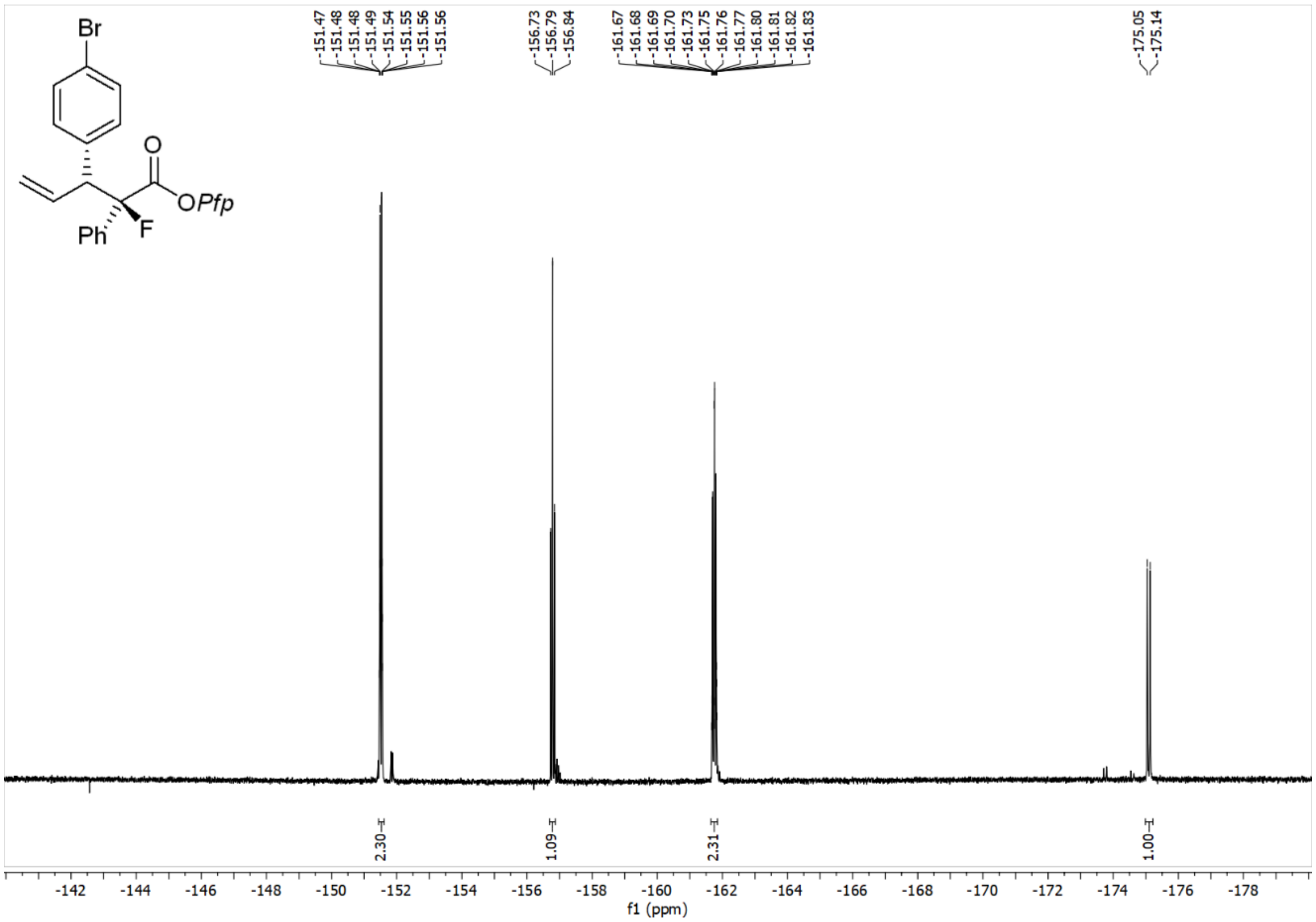

Br
O
OPfp
Ph
F
-151.47
-151.48
-151.48
-151.49
-151.54
-151.55
-151.56
-151.56
-156.73
-156.79
-156.84
-161.67
-161.68
-161.69
-161.70
-161.73
-161.75
-161.76
-161.77
-161.80
-161.81
-161.82
-161.83
-175.05
-175.14
2.30
1.09
2.31
1.00
-142
-144
-146
-148
-150
-152
-154
-156
-158
-160
-162
-164
-166
-168
-170
-172
-174
-176
-178
f1 (ppm)

**Perfluorophenyl (2*R*,3*S*)-3-(4-bromophenyl)-2-fluoro-2-phenylpent-4-enoate (*R*,*S*)-19:**

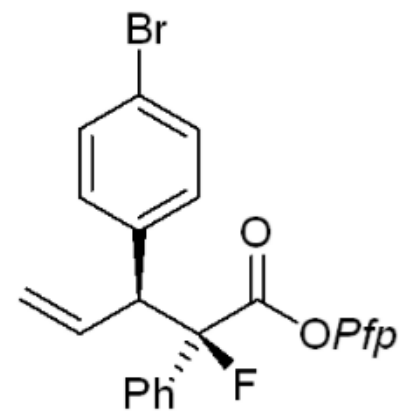


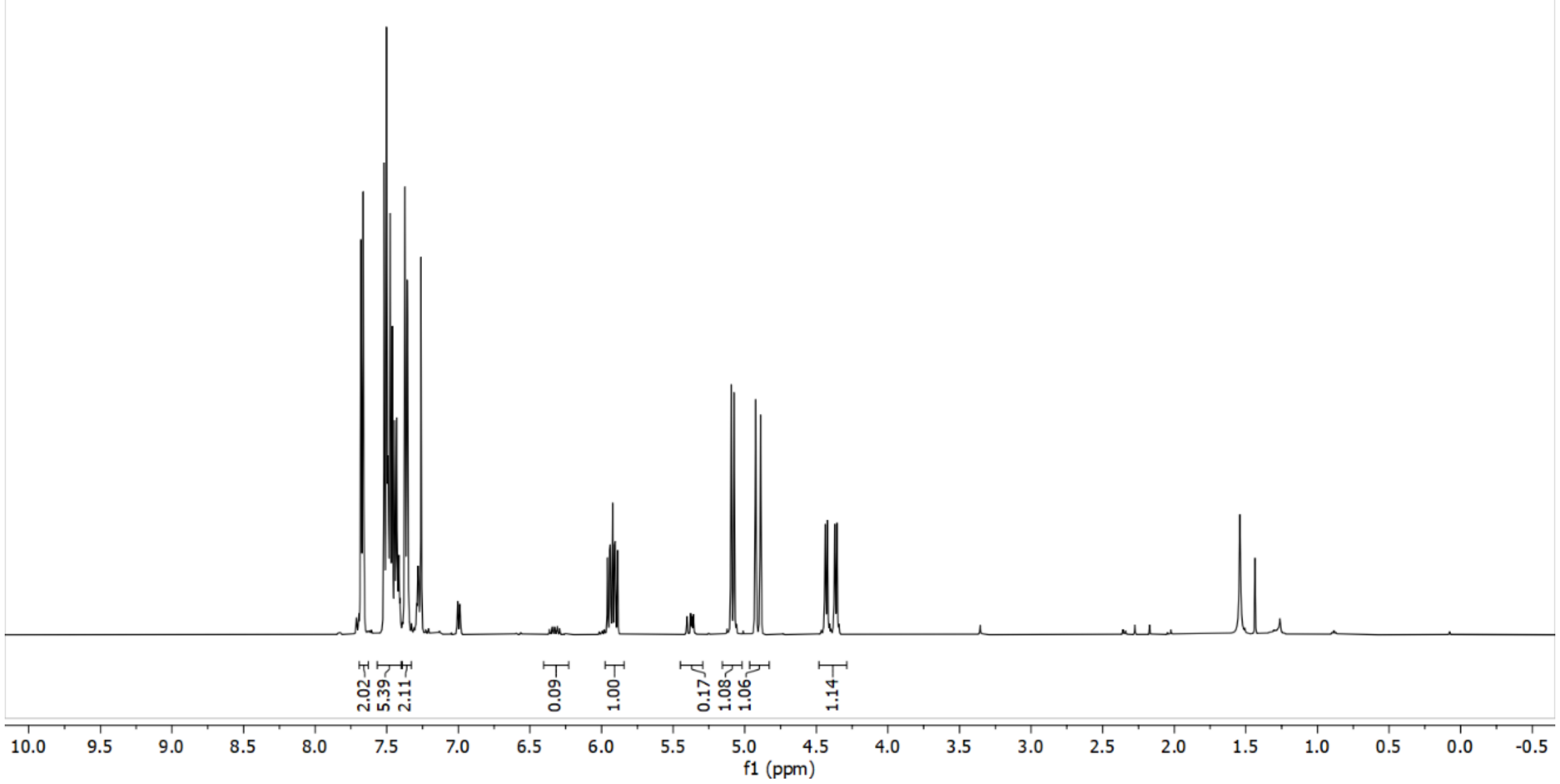


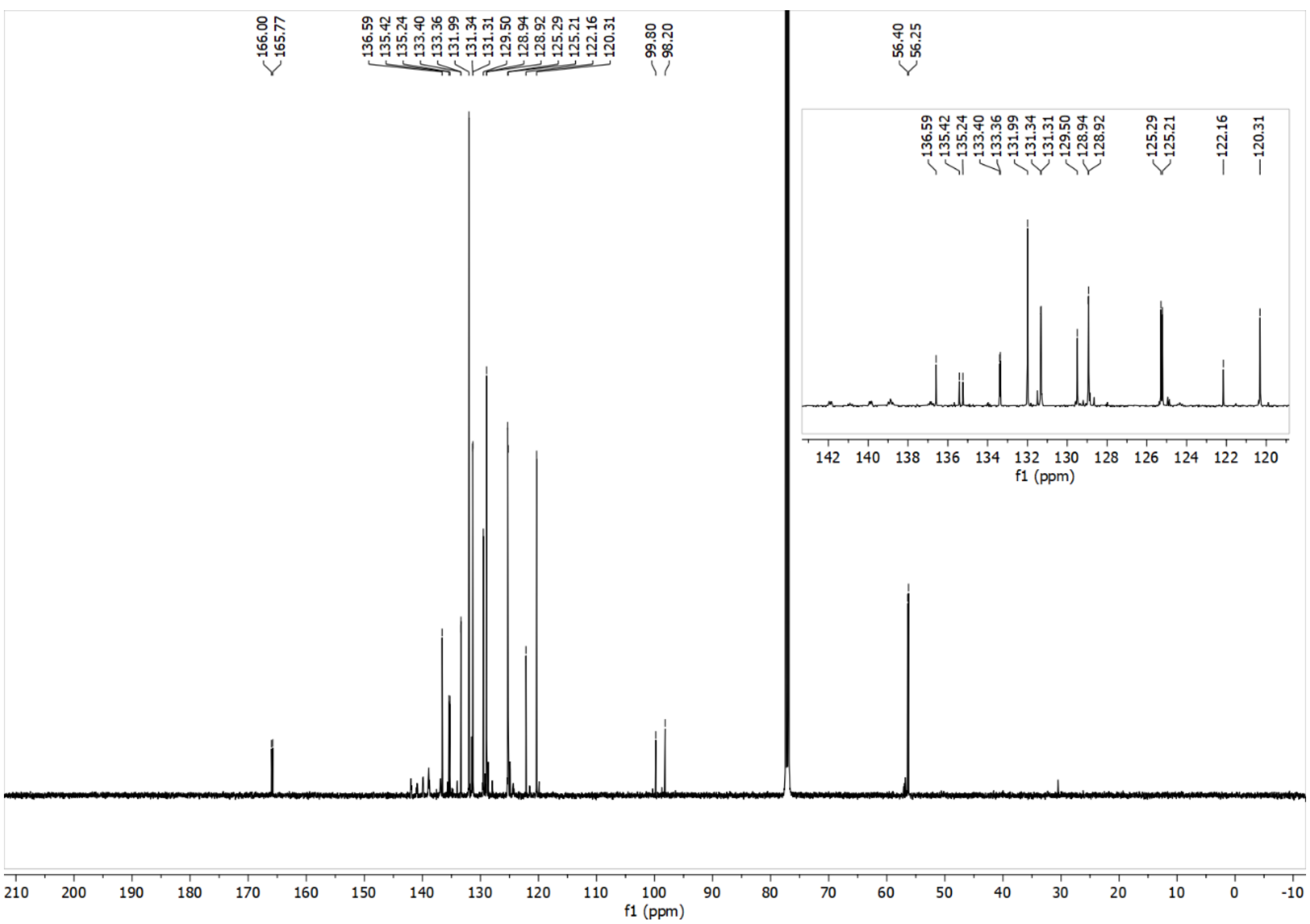

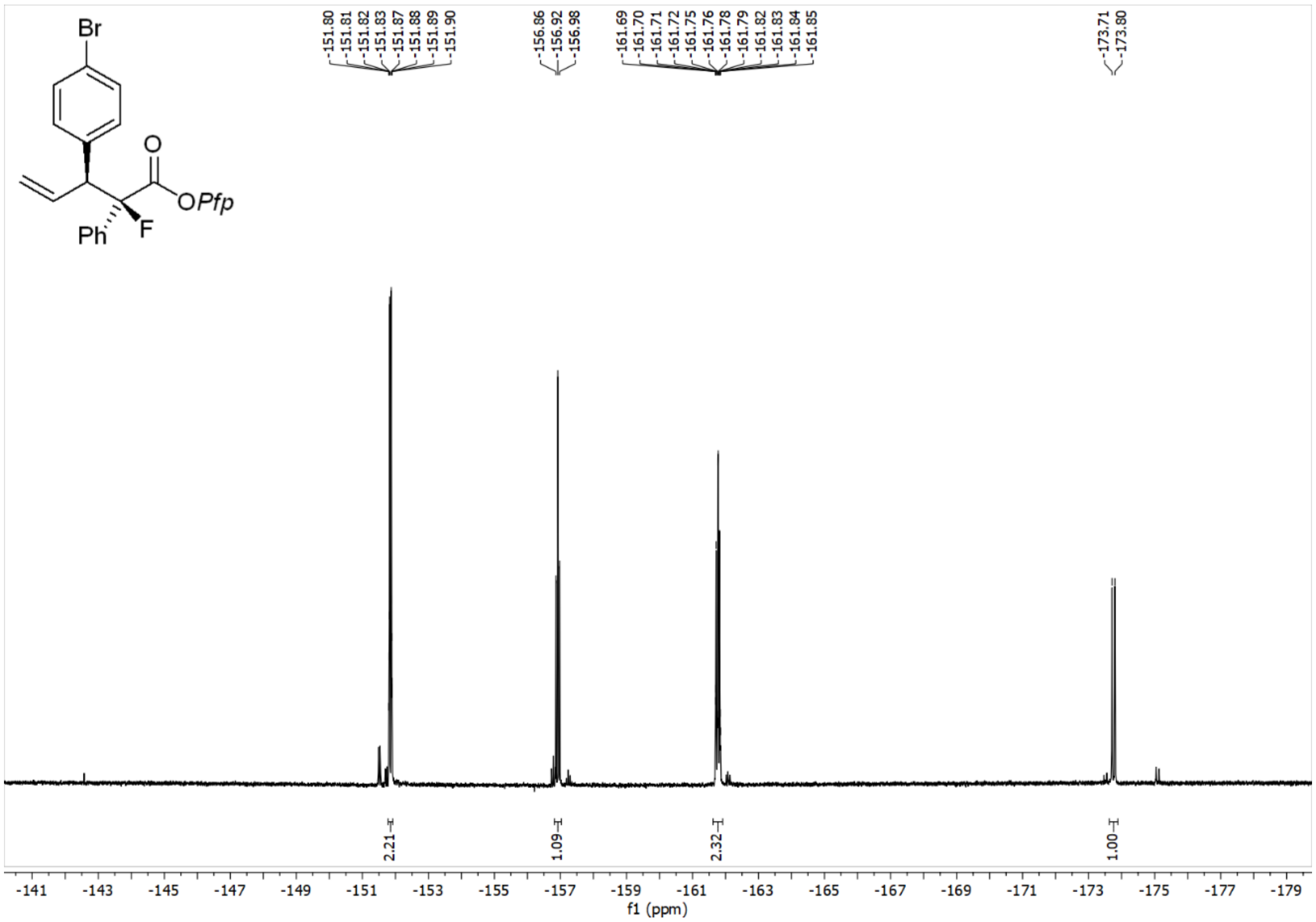

Br
O
OPfp
Ph
F
-151.80
-151.81
-151.82
-151.83
-151.87
-151.88
-151.89
-151.90
-156.86
-156.92
-156.98
-161.69
-161.70
-161.71
-161.72
-161.75
-161.76
-161.78
-161.79
-161.82
-161.83
-161.84
-161.85
-173.71
-173.80
2.21
1.09
2.32
1.00
-141 -143 -145 -147 -149 -151 -153 -155 -157 -159 -161 -163 -165 -167 -169 -171 -173 -175 -177 -179
f1 (ppm)

**Perfluorophenyl (2*R*,3*R*)-2-fluoro-3-(4-methoxyphenyl)-2-phenylpent-4-enoate (*R*,*R*)-20**

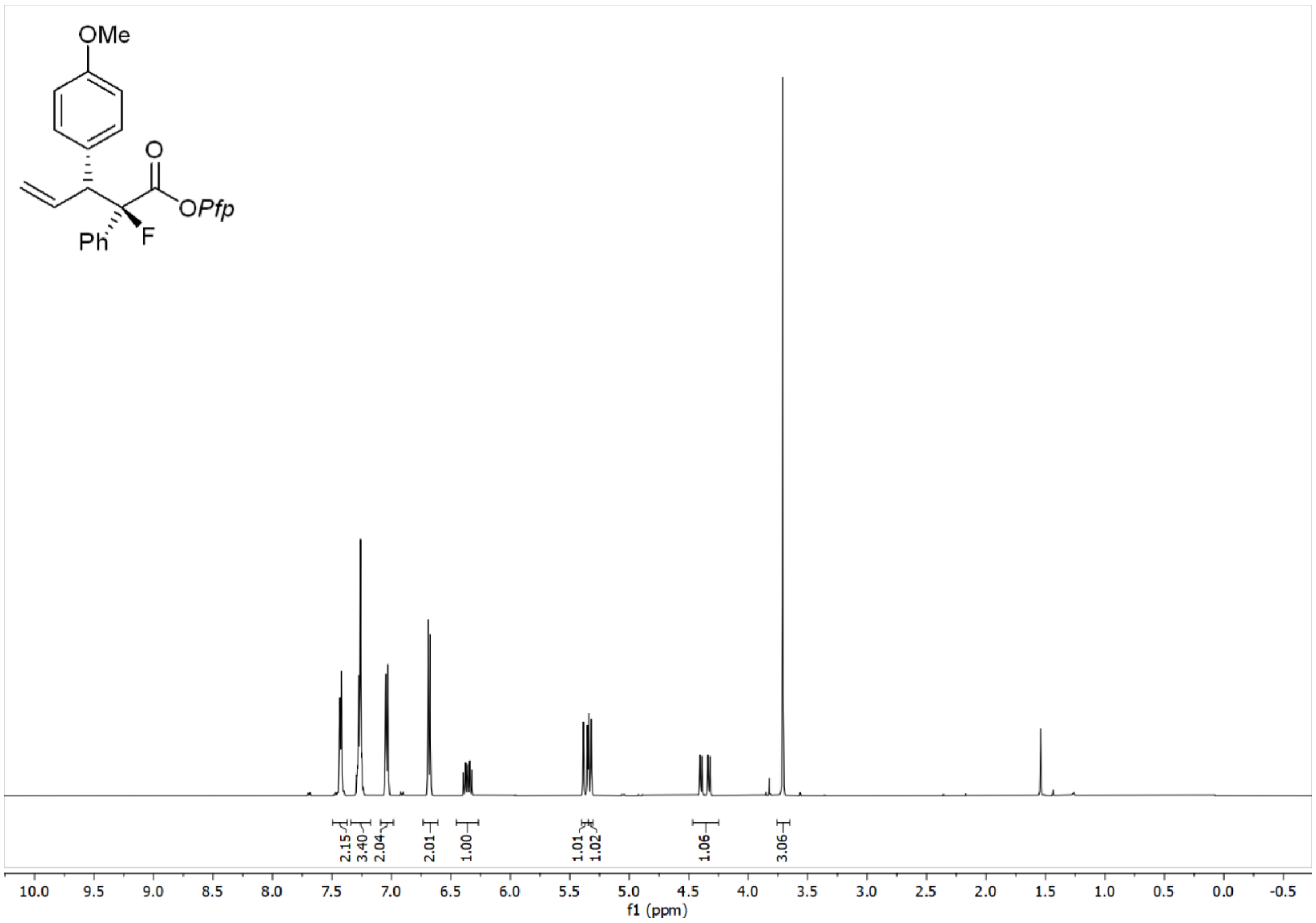


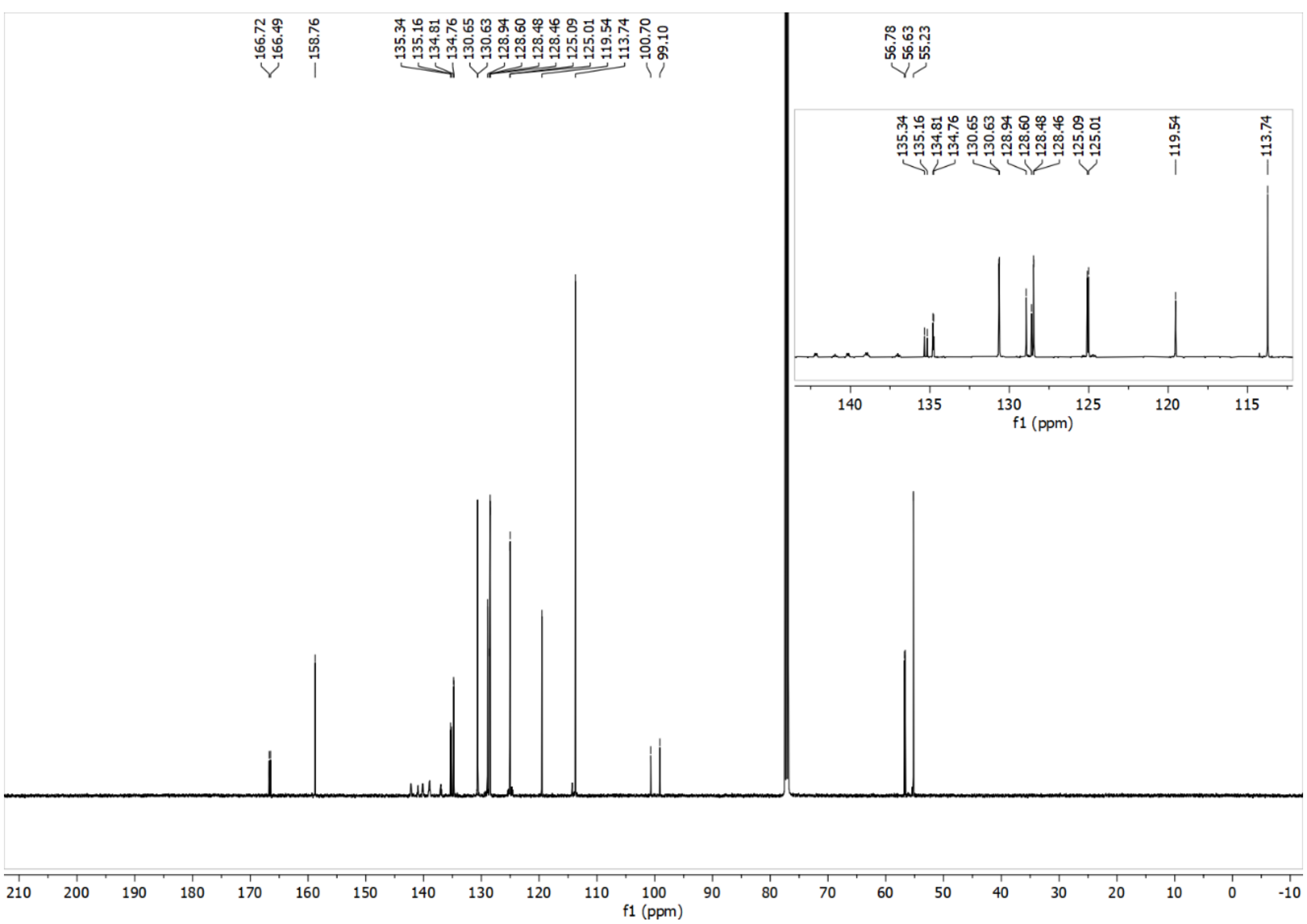

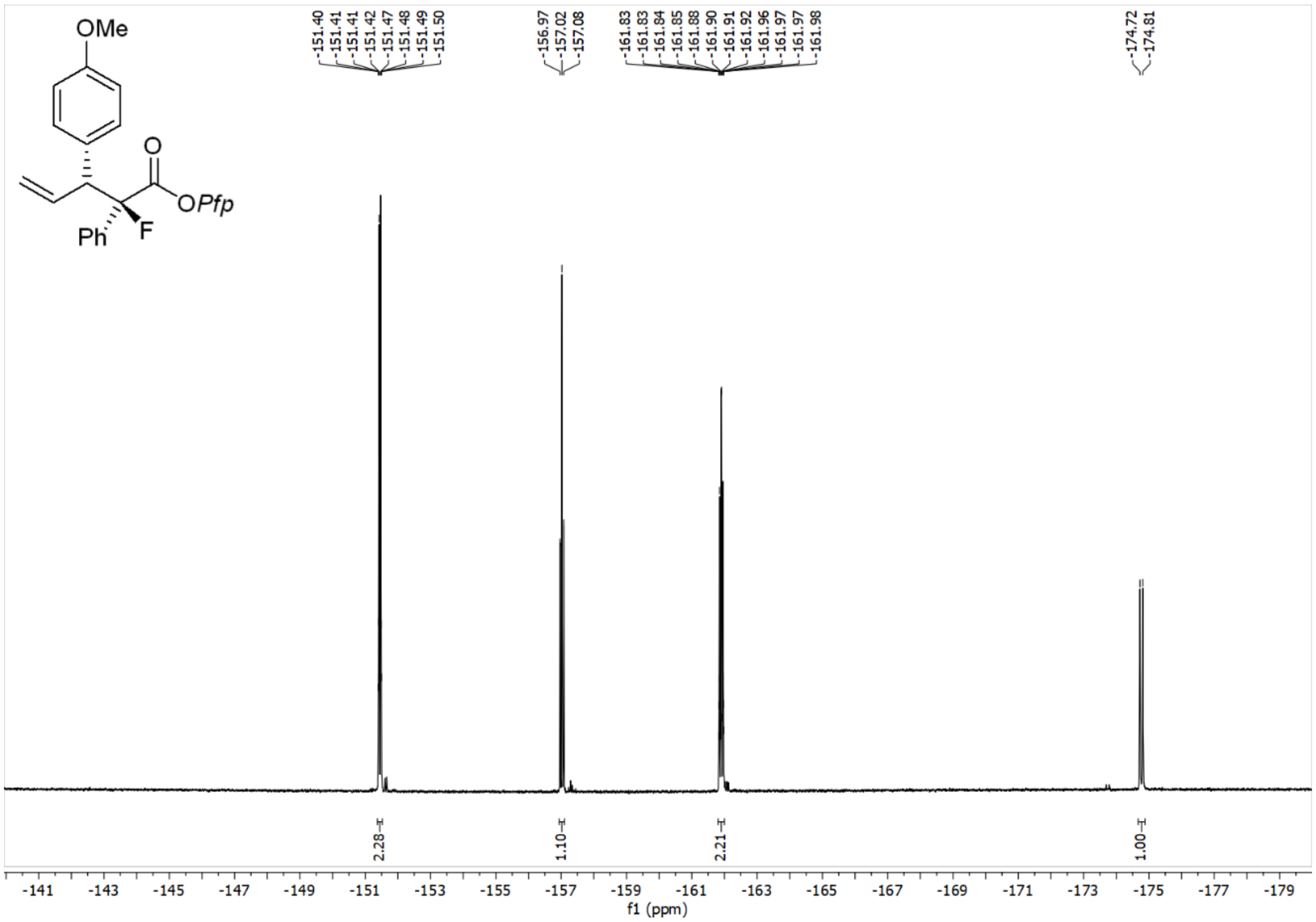

OMe
O
OPfp
Ph
F
-151.40
-151.41
-151.41
-151.42
-151.47
-151.48
-151.49
-151.50
-156.97
-157.02
-157.08
-161.83
-161.83
-161.84
-161.85
-161.88
-161.90
-161.91
-161.92
-161.96
-161.97
-161.98
-174.72
-174.81
2.28
1.10
2.21
1.00
-141 -143 -145 -147 -149 -151 -153 -155 -157 -159 -161 -163 -165 -167 -169 -171 -173 -175 -177 -179
f1 (ppm)

**Perfluorophenyl (2*R*,3*S*)-2-fluoro-3-(4-methoxyphenyl)-2-phenylpent-4-enoate (*R*,*S*)-20:**

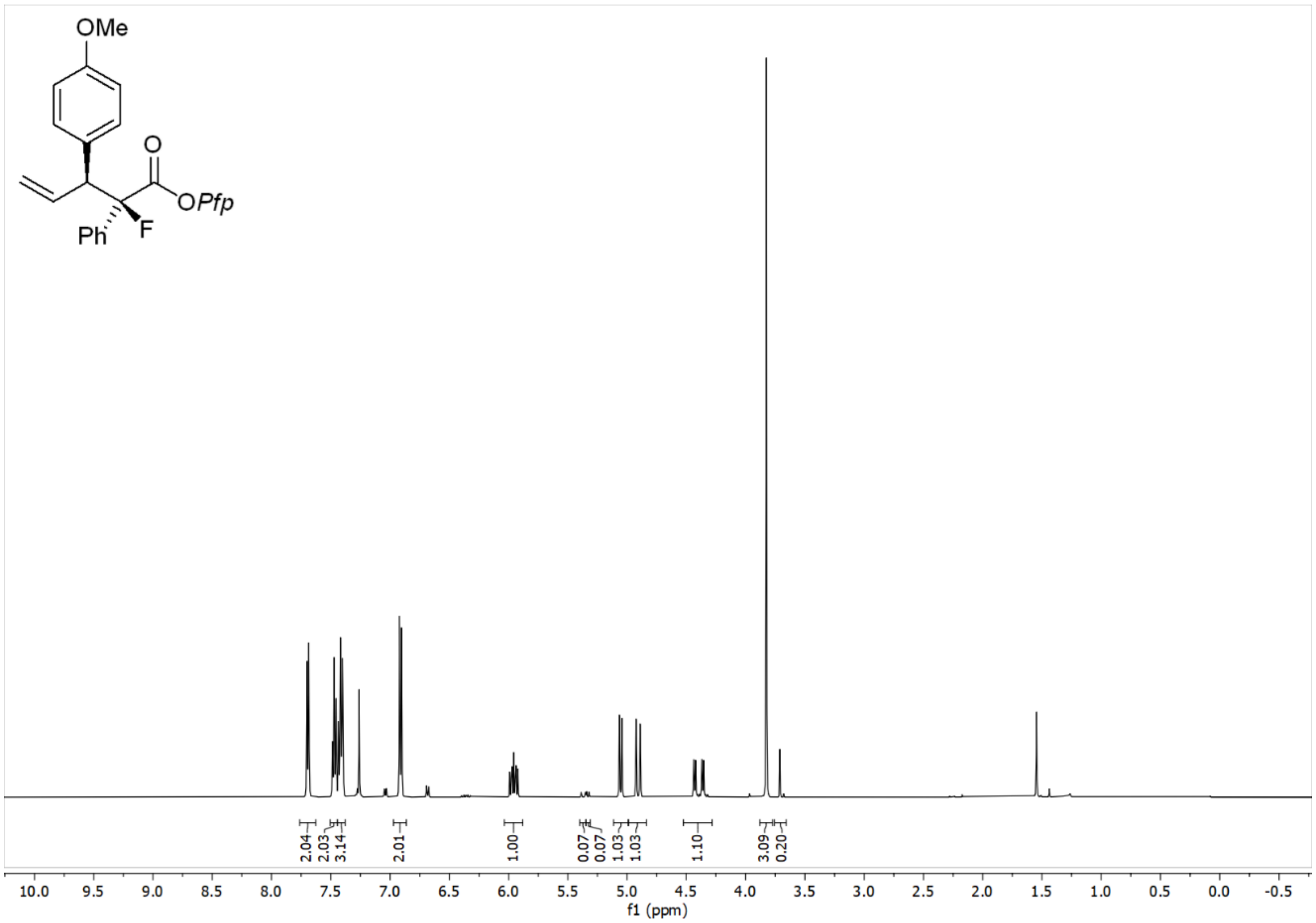


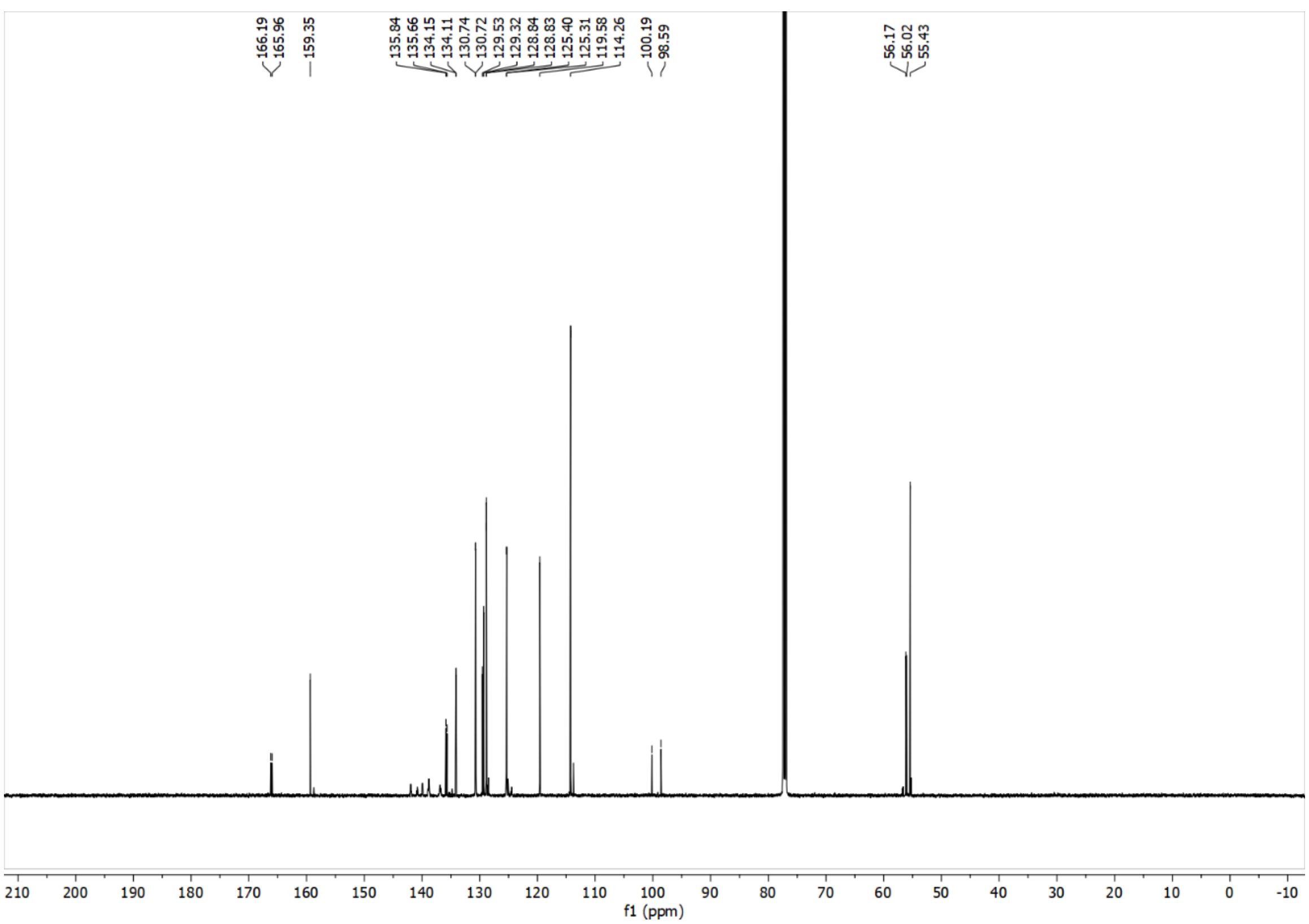

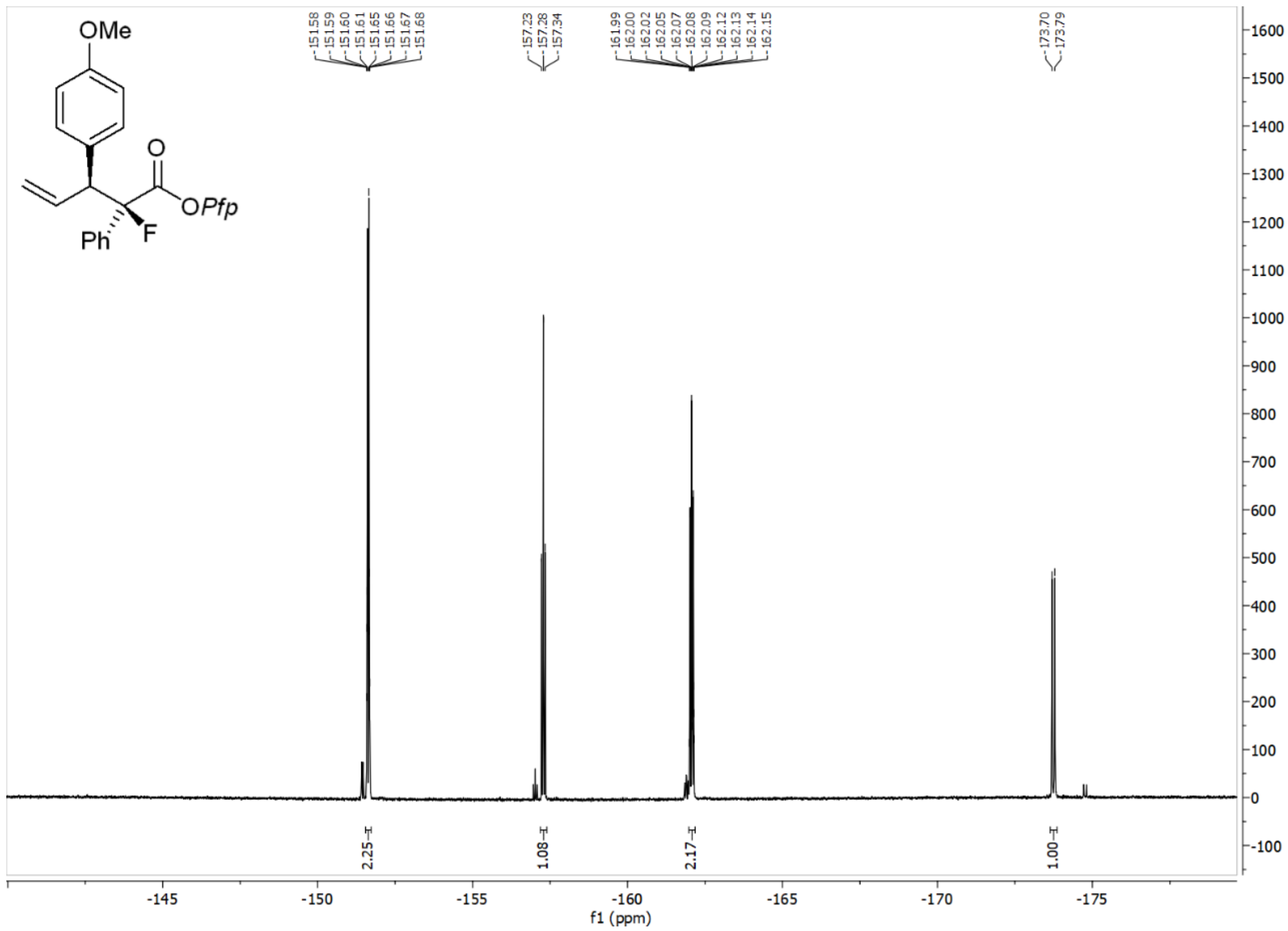

OMe
O
OPfp
Ph
F
-151.58
-151.59
-151.60
-151.61
-151.65
-151.66
-151.67
-151.68
-157.23
-157.28
-157.34
-161.99
-162.00
-162.02
-162.05
-162.07
-162.08
-162.09
-162.12
-162.13
-162.14
-162.15
-173.70
-173.79
2.25
1.08
2.17
1.00
-145
-150
-155
-160
-165
-170
-175
f1 (ppm)

**Perfluorophenyl (2*R*,3*R*)-2-fluoro-3-(4-nitrophenyl)-2-phenylpent-4-enoate (*R*,*R*)-21:**

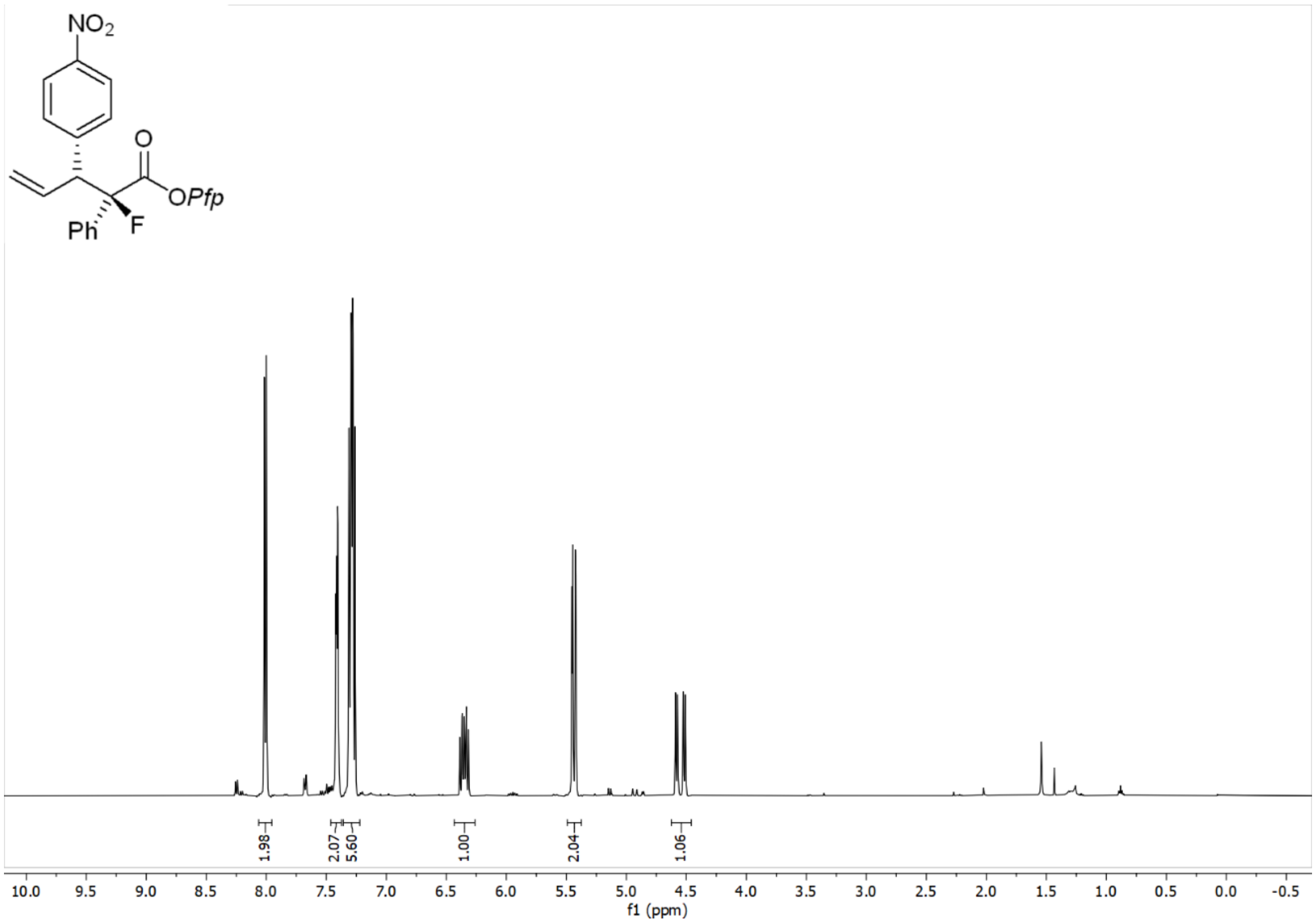

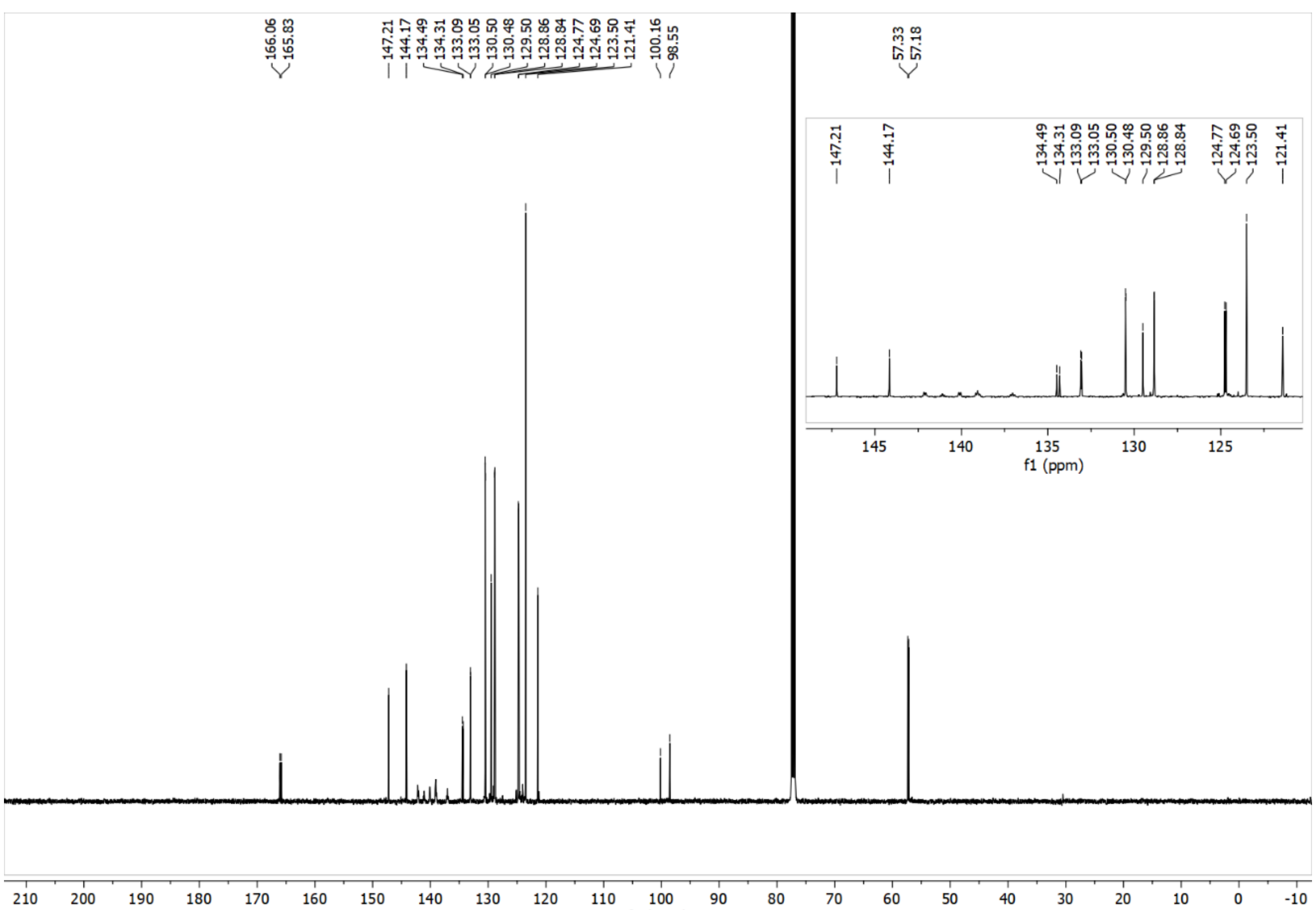

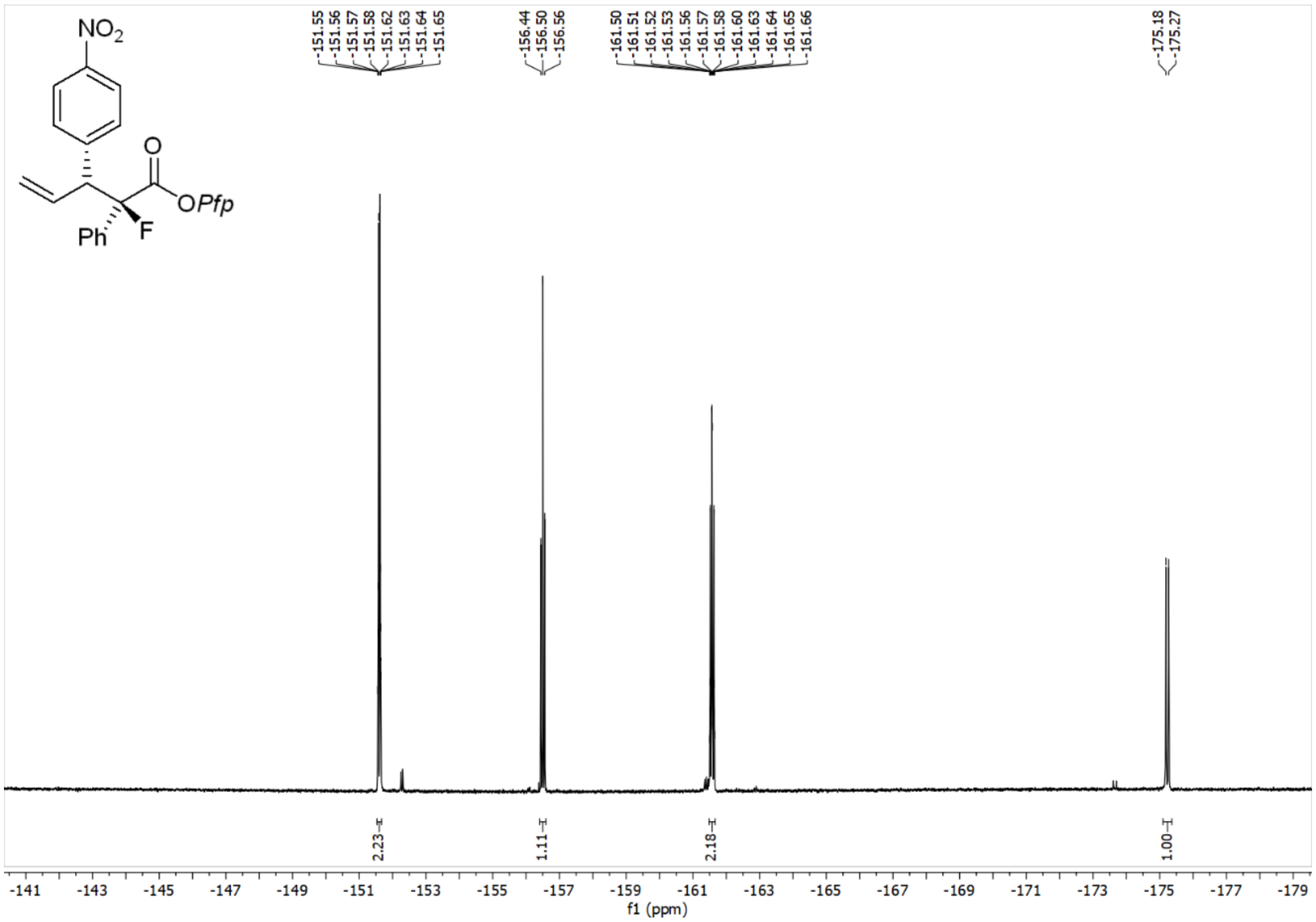

NO2
OPfp
Ph
F
-151.55
-151.56
-151.57
-151.58
-151.62
-151.63
-151.64
-151.65
-156.44
-156.50
-156.56
-161.50
-161.51
-161.52
-161.53
-161.56
-161.57
-161.58
-161.60
-161.63
-161.64
-161.65
-161.66
-175.18
-175.27
2.23
1.11
2.18
1.00
-141
-143
-145
-147
-149
-151
-153
-155
-157
-159
-161
-163
-165
-167
-169
-171
-173
-175
-177
-179
f1 (ppm)

**Perfluorophenyl (2*R*,3*S*)-2-fluoro-3-(4-nitrophenyl)-2-phenylpent-4-enoate (*R*,*S*)-21:**

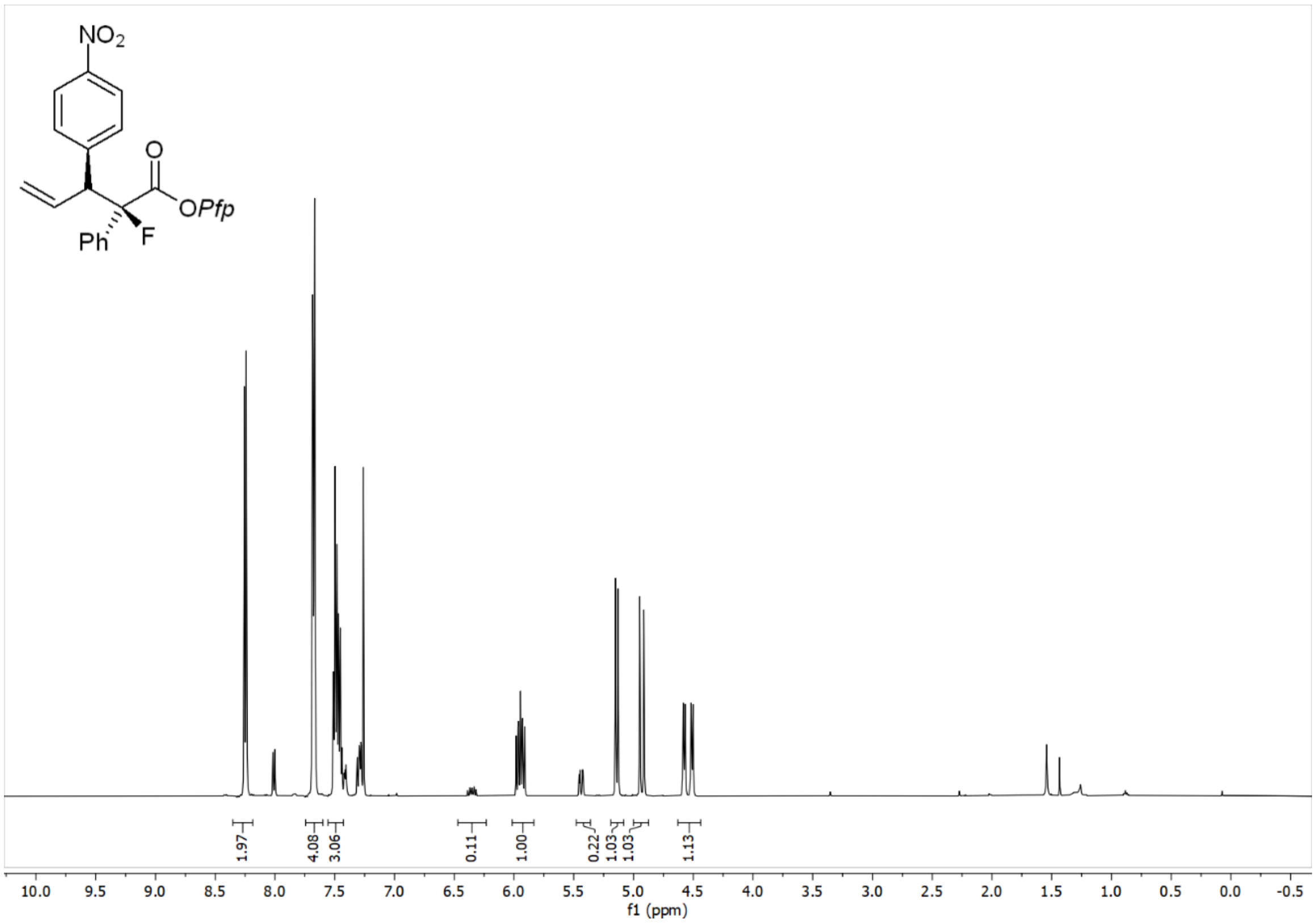


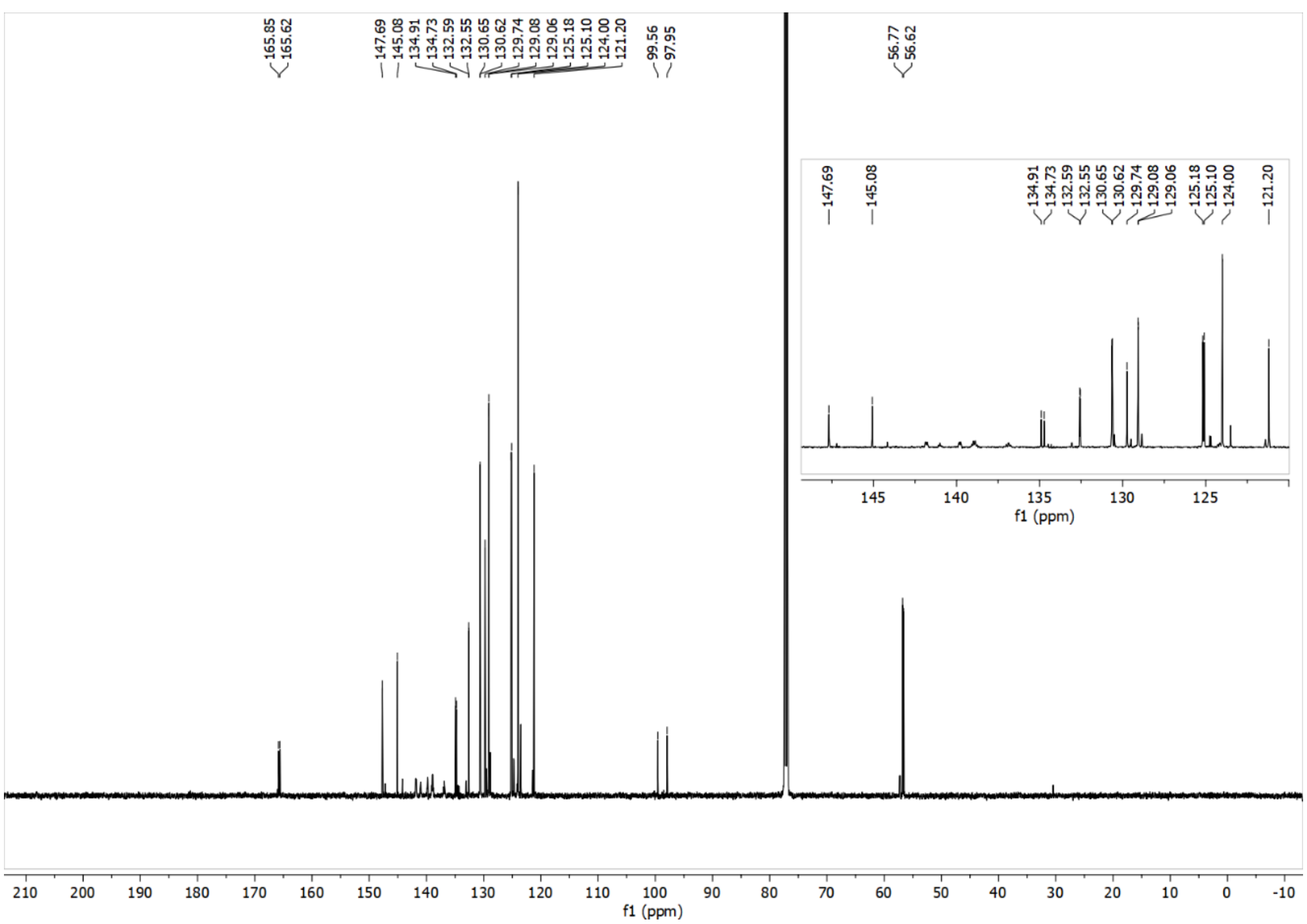

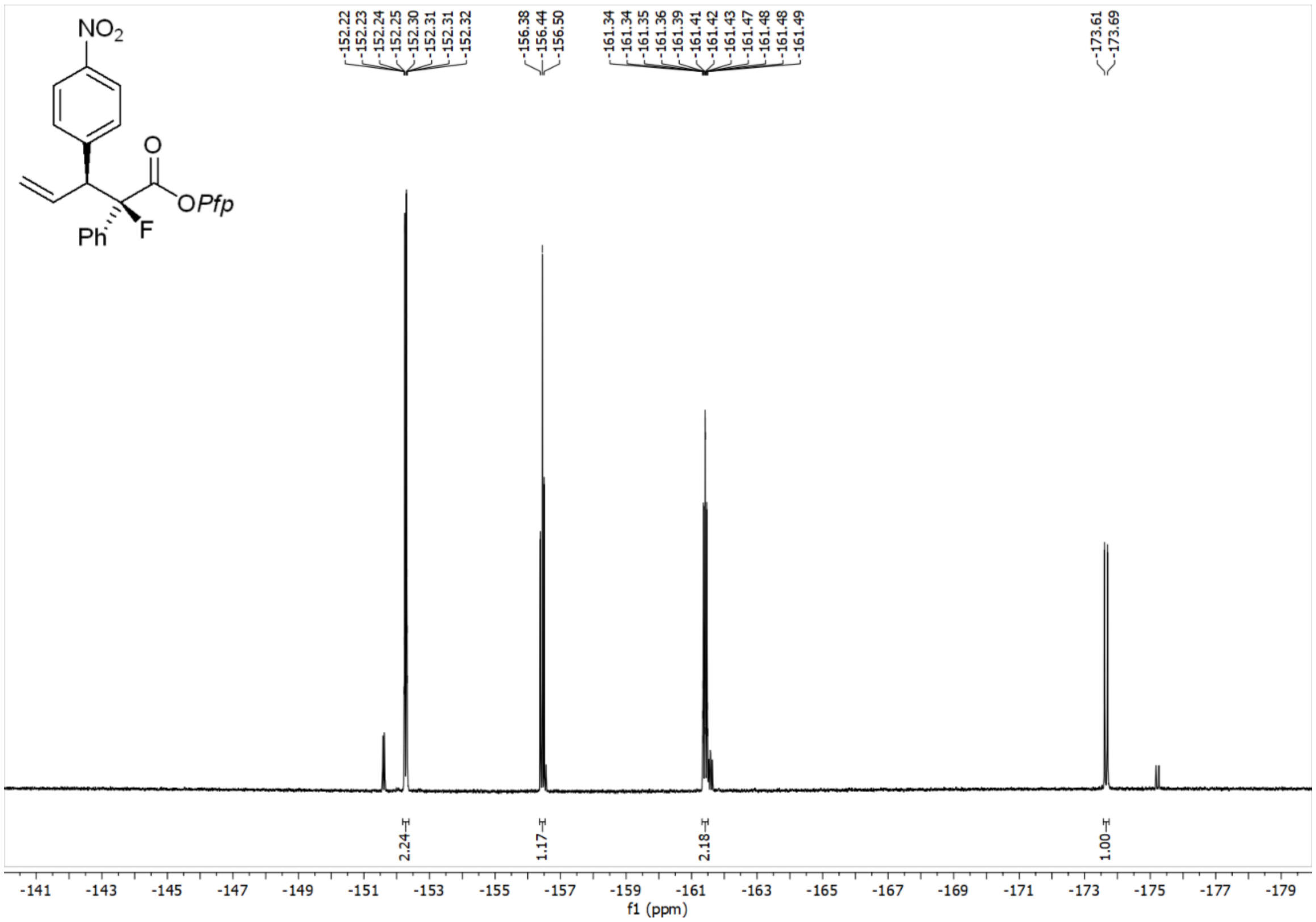

NO2
O
OPfp
Ph
F
-152.22
-152.23
-152.24
-152.25
-152.30
-152.31
-152.31
-152.32
-156.38
-156.44
-156.50
-161.34
-161.34
-161.35
-161.36
-161.39
-161.41
-161.42
-161.43
-161.47
-161.48
-161.48
-161.49
-173.61
-173.69
2.24
1.17
2.18
1.00
-141 -143 -145 -147 -149 -151 -153 -155 -157 -159 -161 -163 -165 -167 -169 -171 -173 -175 -177 -179
f1 (ppm)

**Perfluorophenyl (2*R*,3*R*)-2-fluoro-2-phenyl-3-(4-(trifluoromethyl)phenyl)pent-4-enoate (*R*,*R*)-22:**

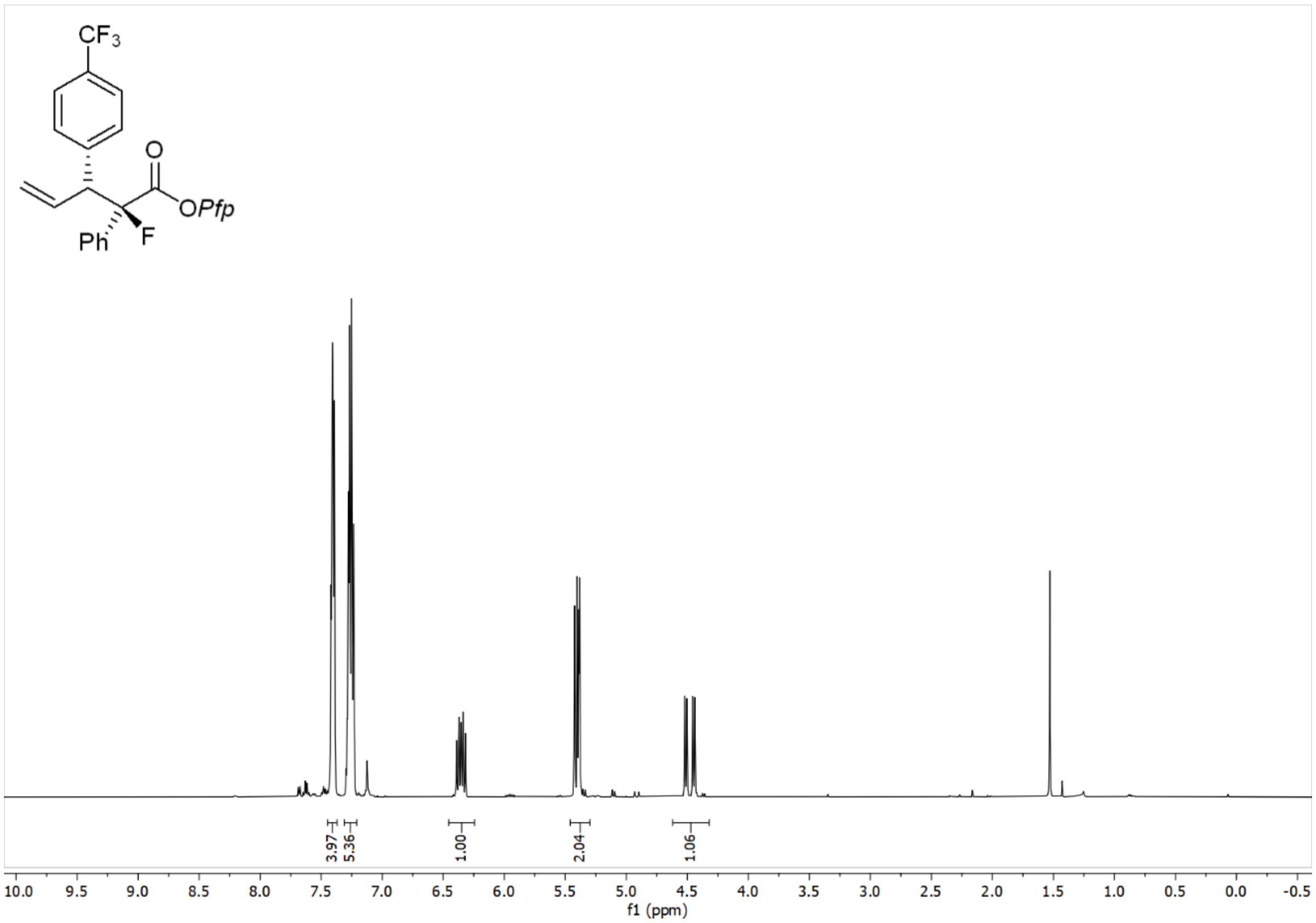


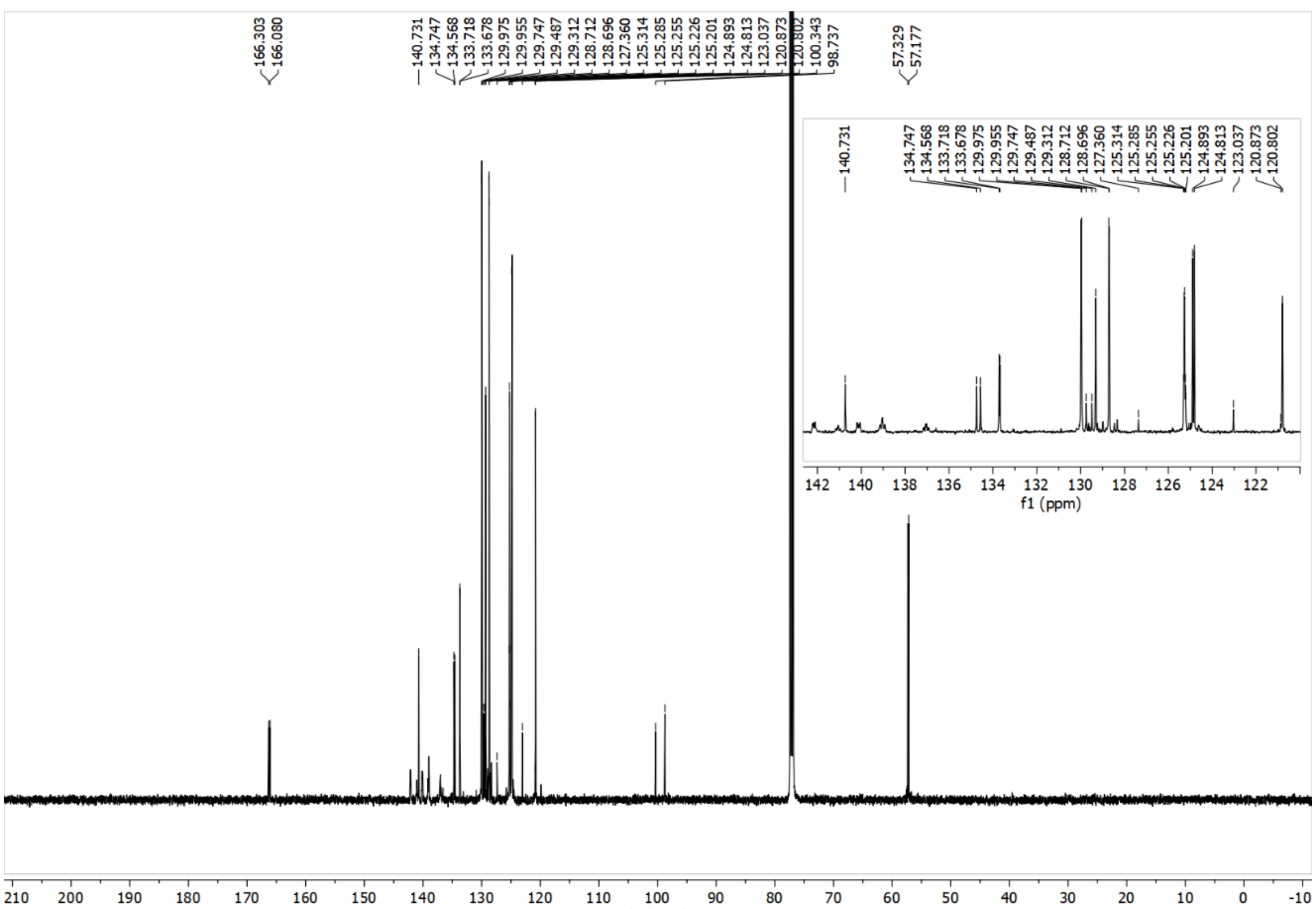

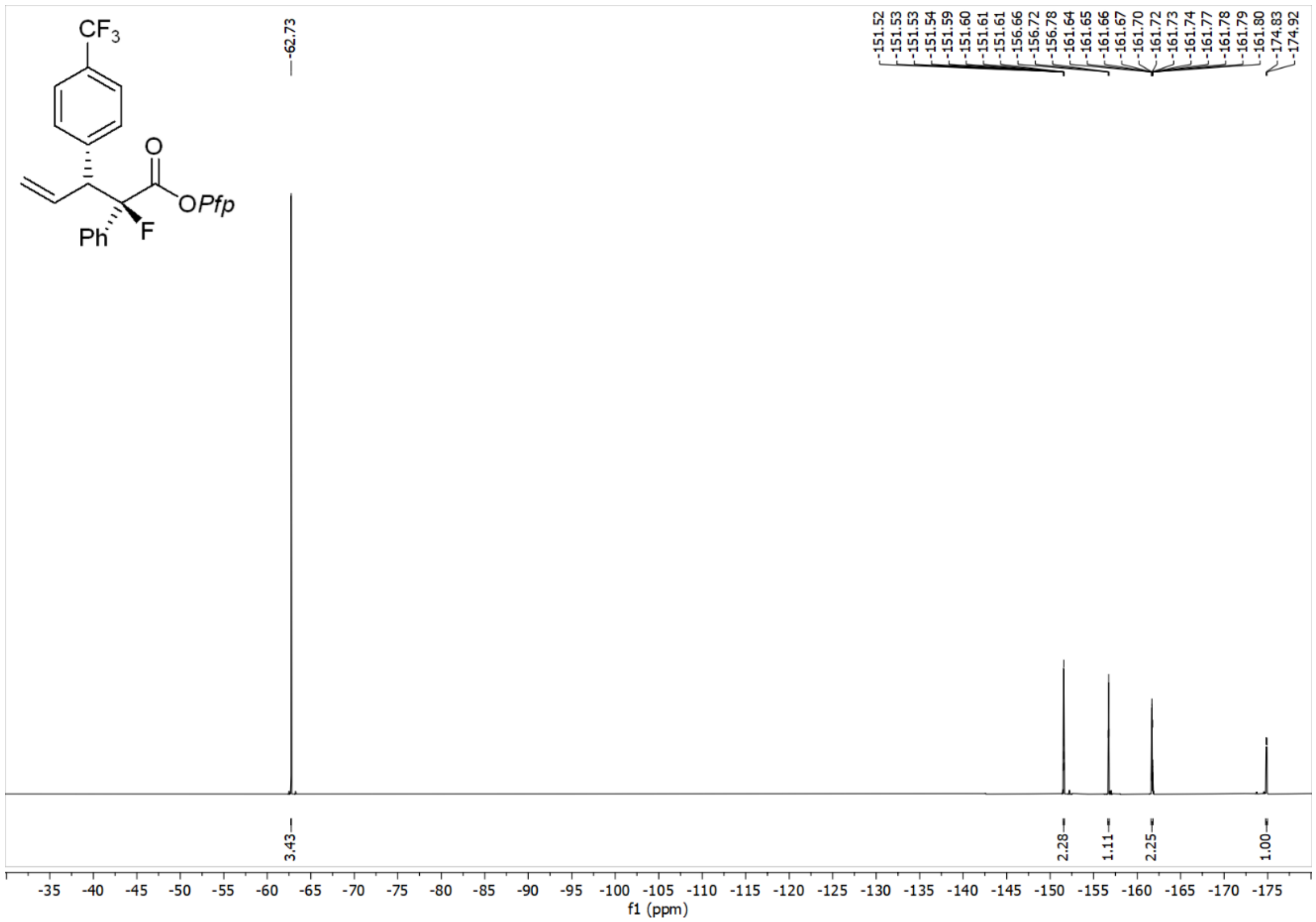
CF3
OPfp
Ph
F
O
-62.73
-151.52
-151.53
-151.53
-151.54
-151.59
-151.60
-151.61
-151.61
-156.66
-156.72
-156.78
-161.64
-161.65
-161.66
-161.67
-161.70
-161.72
-161.73
-161.74
-161.77
-161.78
-161.79
-161.80
-174.83
-174.92
3.43
2.28
1.11
2.25
1.00
-35 -40 -45 -50 -55 -60 -65 -70 -75 -80 -85 -90 -95 -100 -105 -110 -115 -120 -125 -130 -135 -140 -145 -150 -155 -160 -165 -170 -175
f1 (ppm)

**Perfluorophenyl (2*R*,3*S*)-2-fluoro-2-phenyl-3-(4-(trifluoromethyl)phenyl)pent-4-enoate (*R*,*S*)-22:**

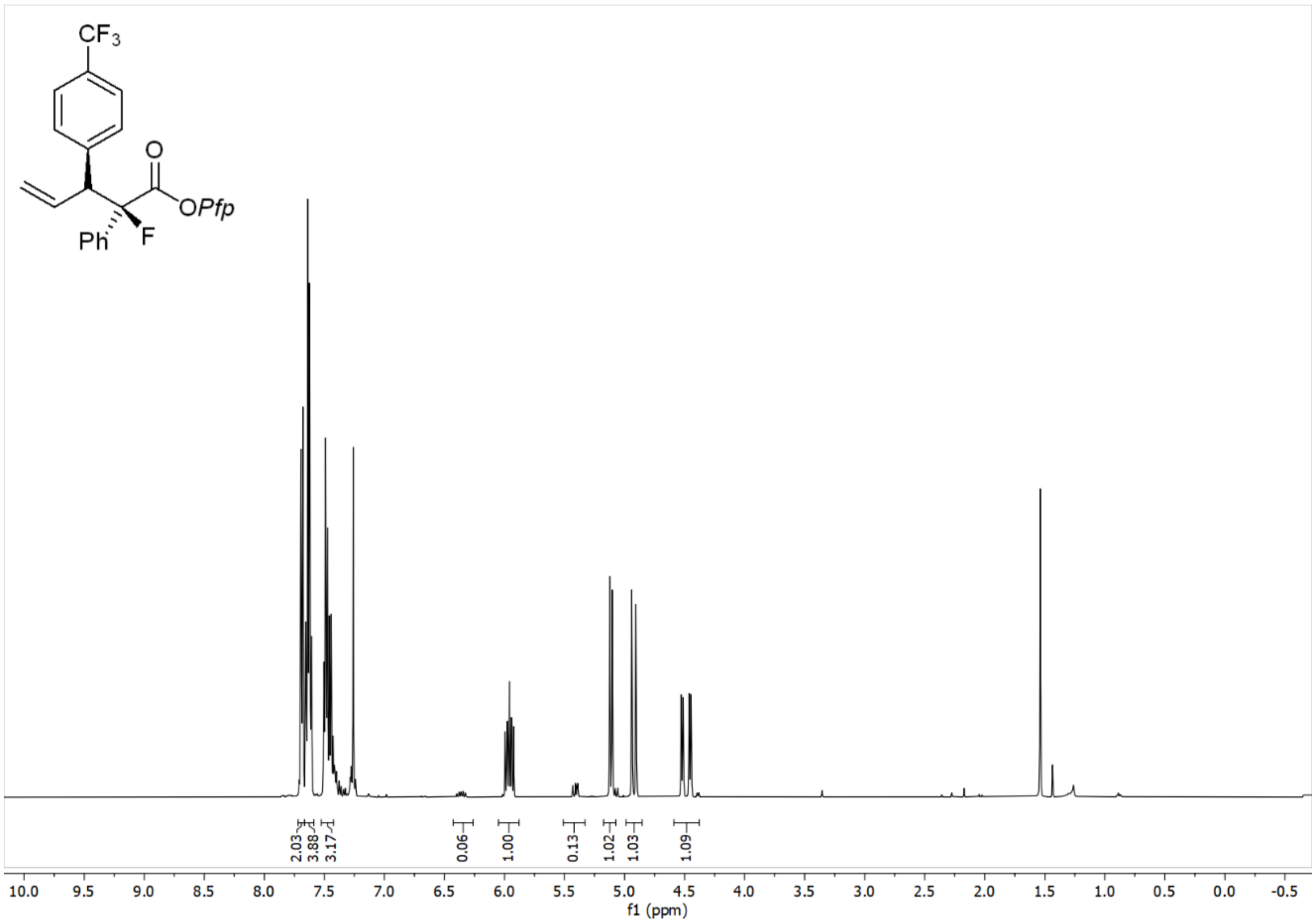

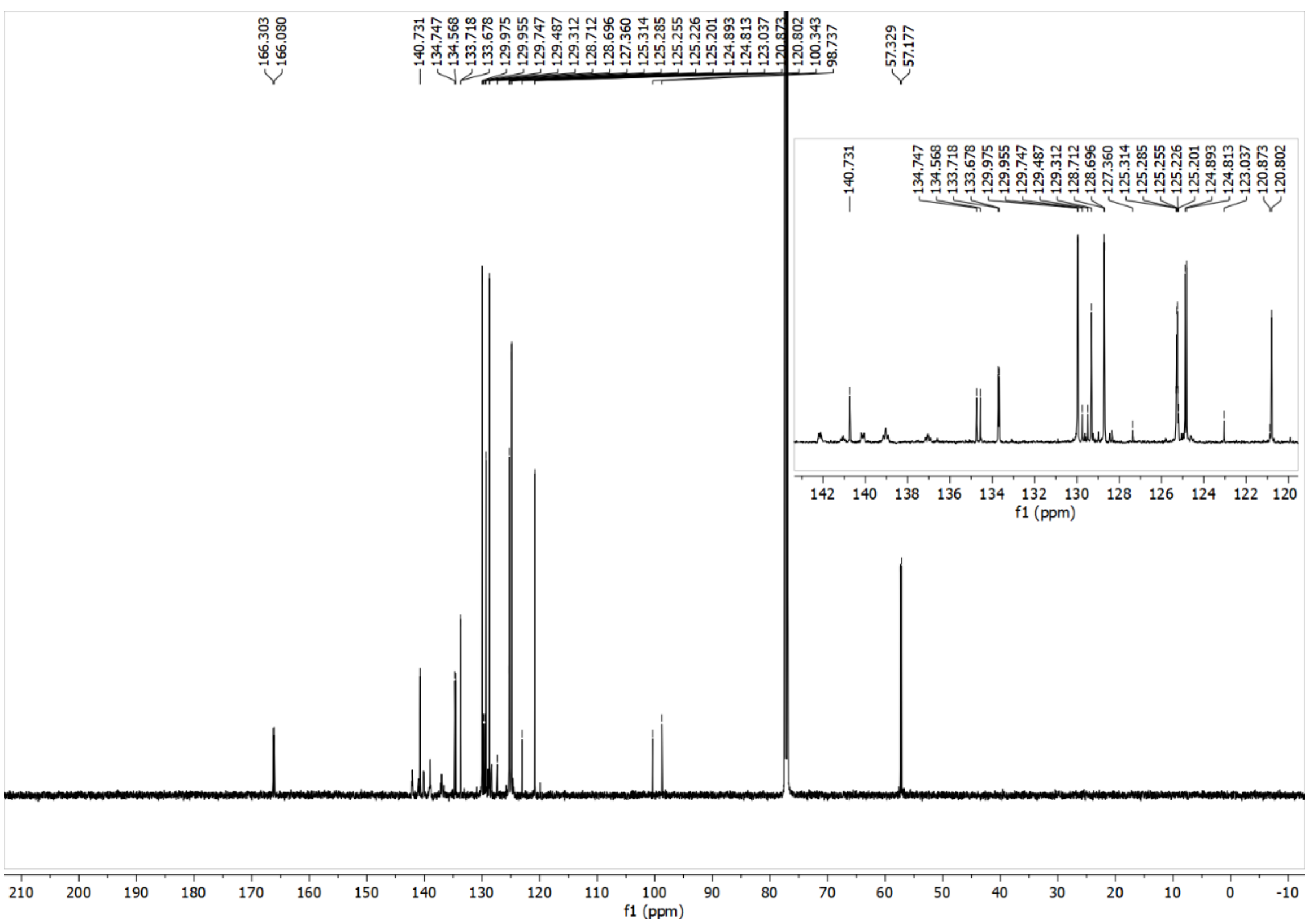

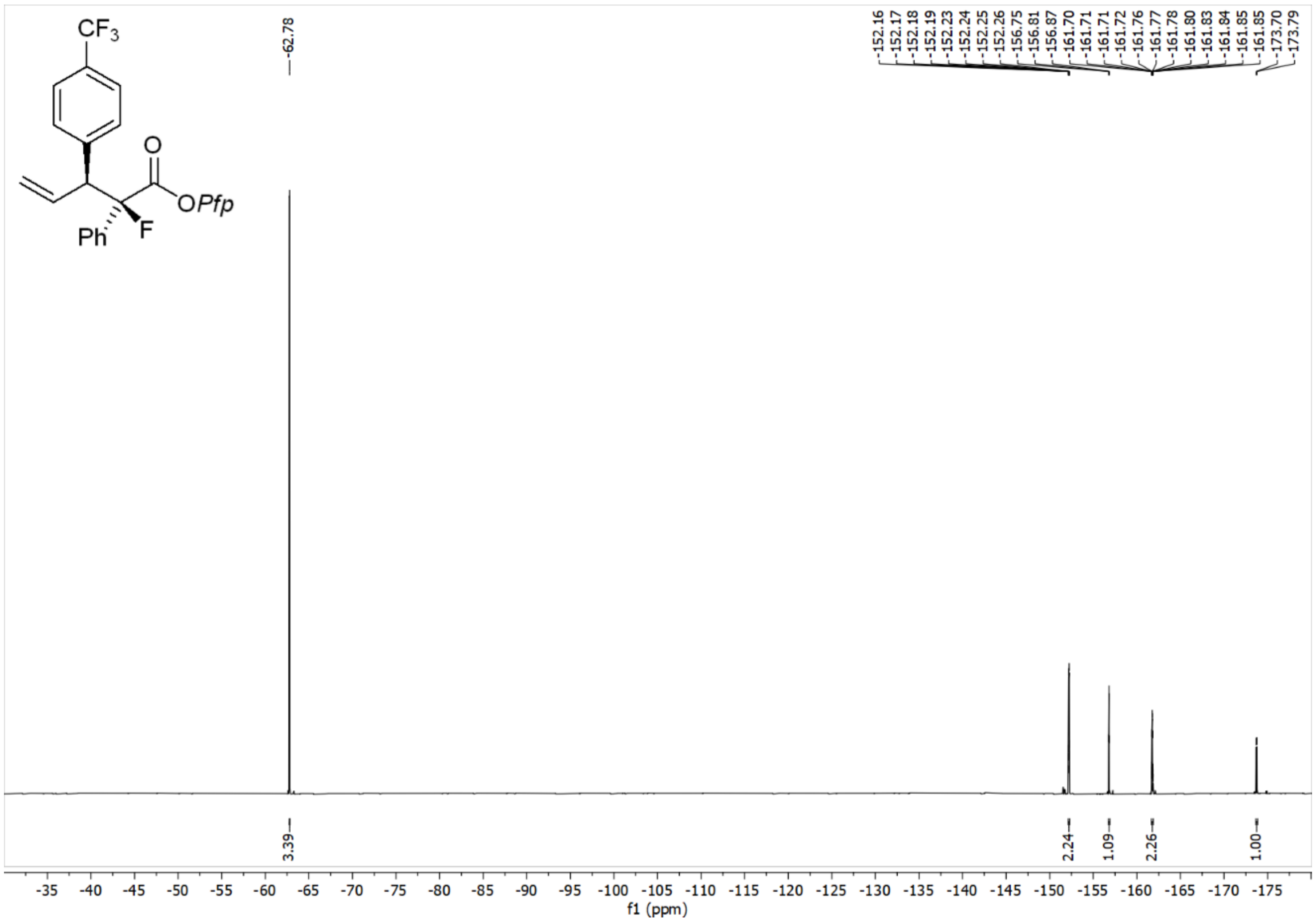
CF3
O
OPfp
Ph
F
-62.78
-152.16
-152.17
-152.18
-152.19
-152.23
-152.24
-152.25
-152.26
-156.75
-156.81
-156.87
-161.70
-161.71
-161.71
-161.72
-161.76
-161.77
-161.78
-161.80
-161.83
-161.84
-161.85
-161.85
-173.70
-173.79
3.39
2.24
1.09
2.26
1.00
-35 -40 -45 -50 -55 -60 -65 -70 -75 -80 -85 -90 -95 -100 -105 -110 -115 -120 -125 -130 -135 -140 -145 -150 -155 -160 -165 -170 -175
f1 (ppm)

**Perfluorophenyl (2*R*,3*R*)-2-fluoro-3-(naphthalen-2-yl)-2-phenylpent-4-enoate (*R*,*R*)-15:**

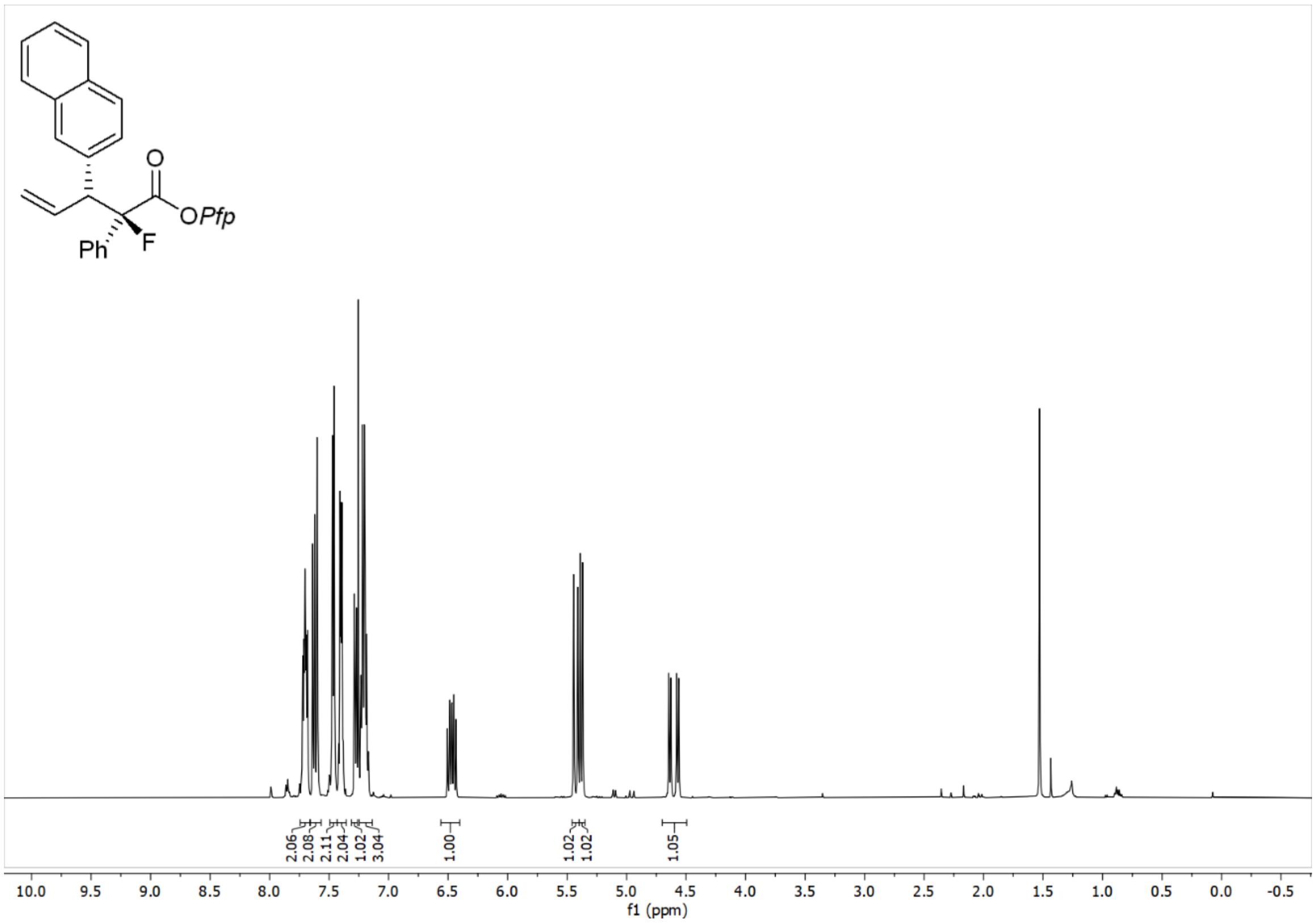

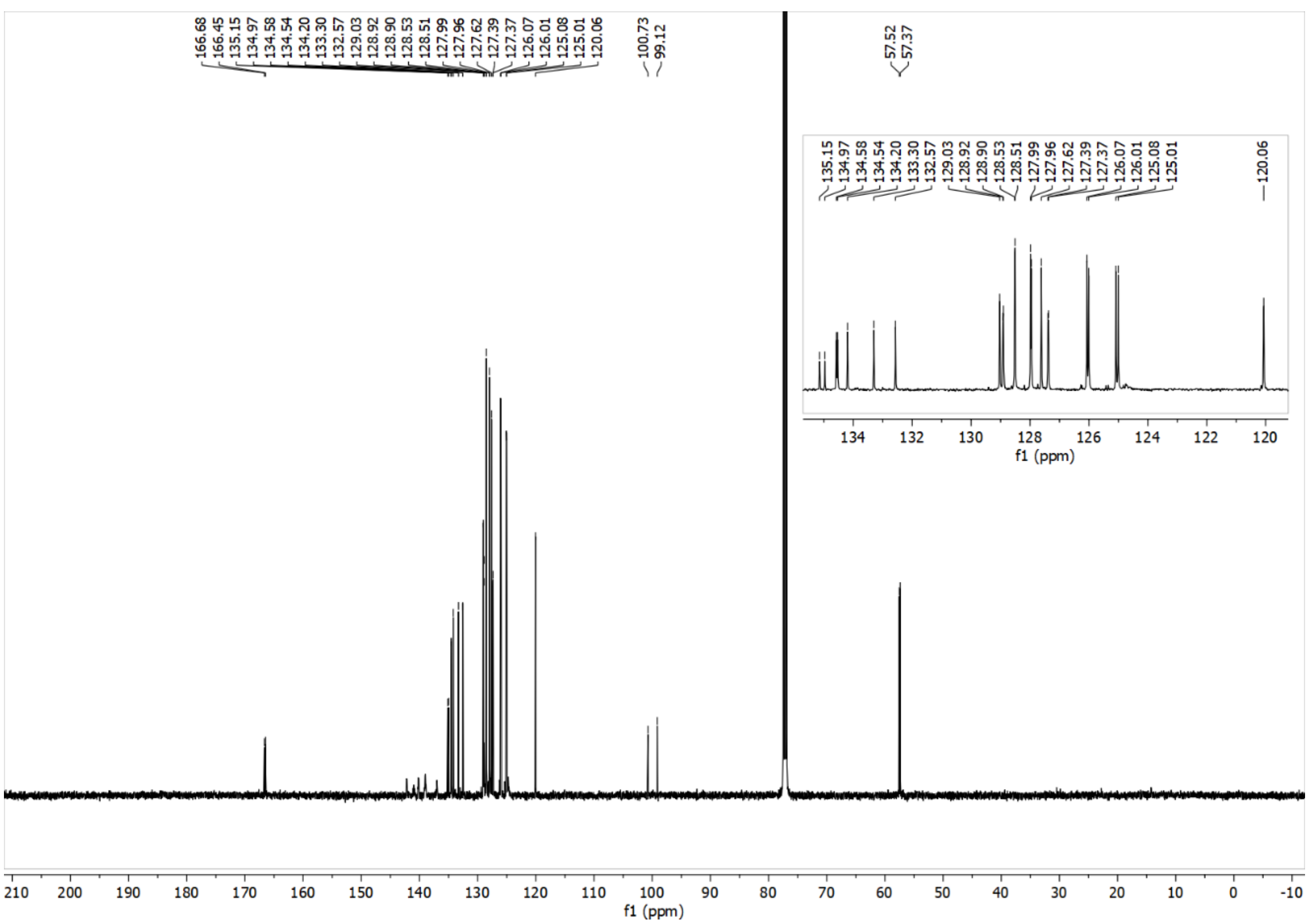

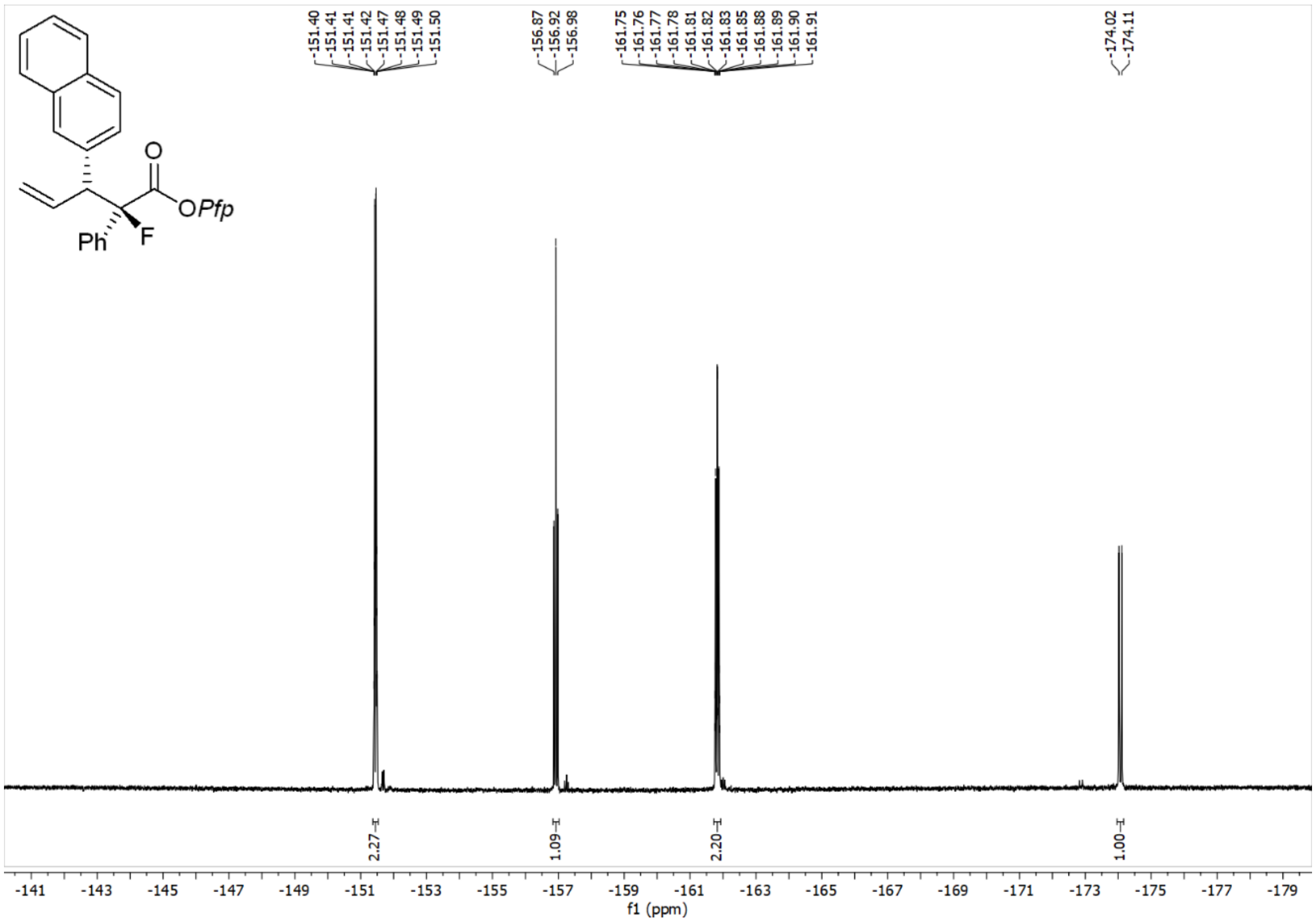
OPfp
Ph
F
O
-151.40
-151.41
-151.41
-151.42
-151.47
-151.48
-151.49
-151.50
-156.87
-156.92
-156.98
-161.75
-161.76
-161.77
-161.78
-161.81
-161.82
-161.83
-161.85
-161.88
-161.89
-161.90
-161.91
-174.02
-174.11
2.27
1.09
2.20
1.00
-141 -143 -145 -147 -149 -151 -153 -155 -157 -159 -161 -163 -165 -167 -169 -171 -173 -175 -177 -179
f1 (ppm)

**Perfluorophenyl (2*R*,3*S*)-2-fluoro-3-(naphthalen-2-yl)-2-phenylpent-4-enoate (*R*,*S*)-15:**

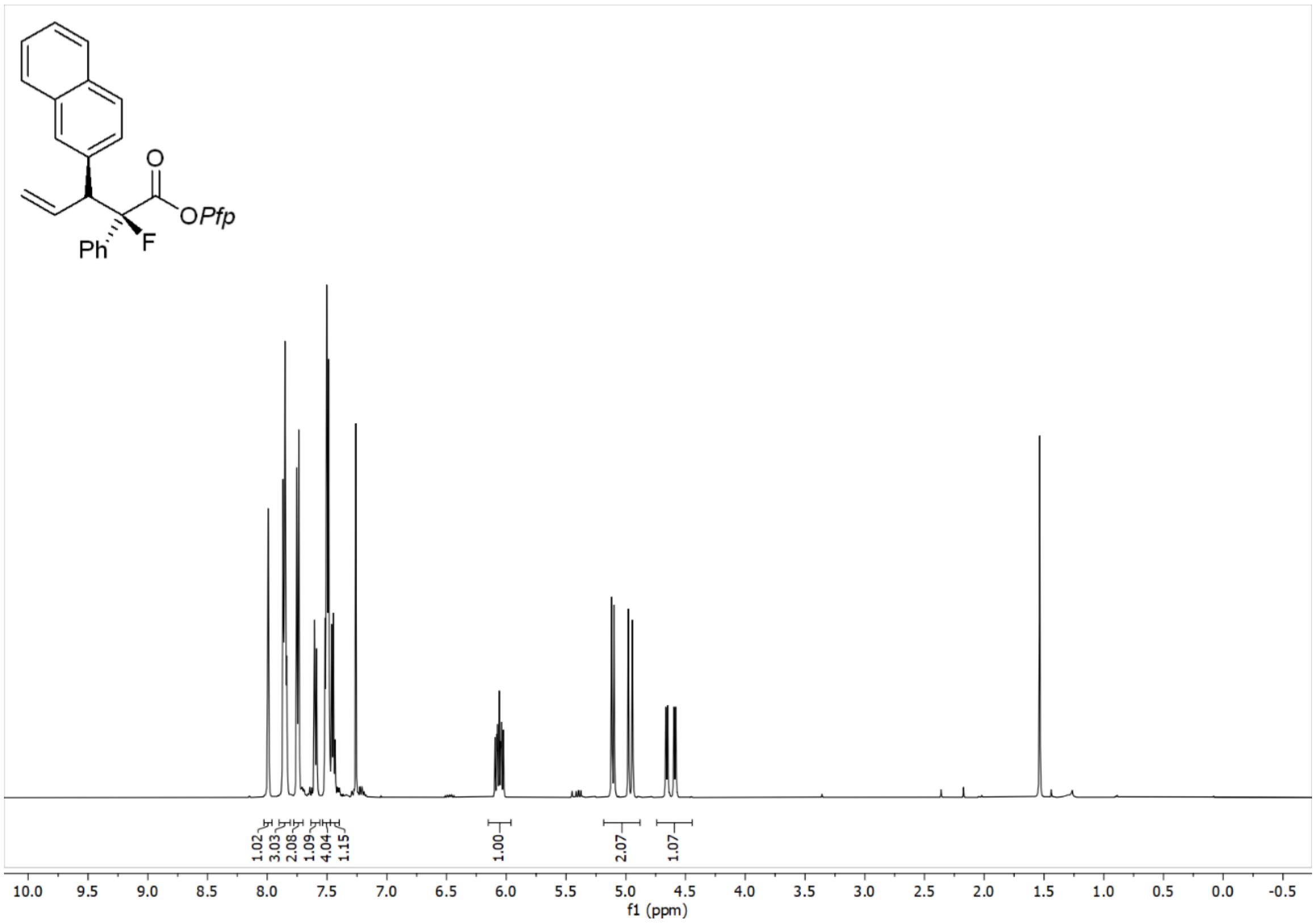


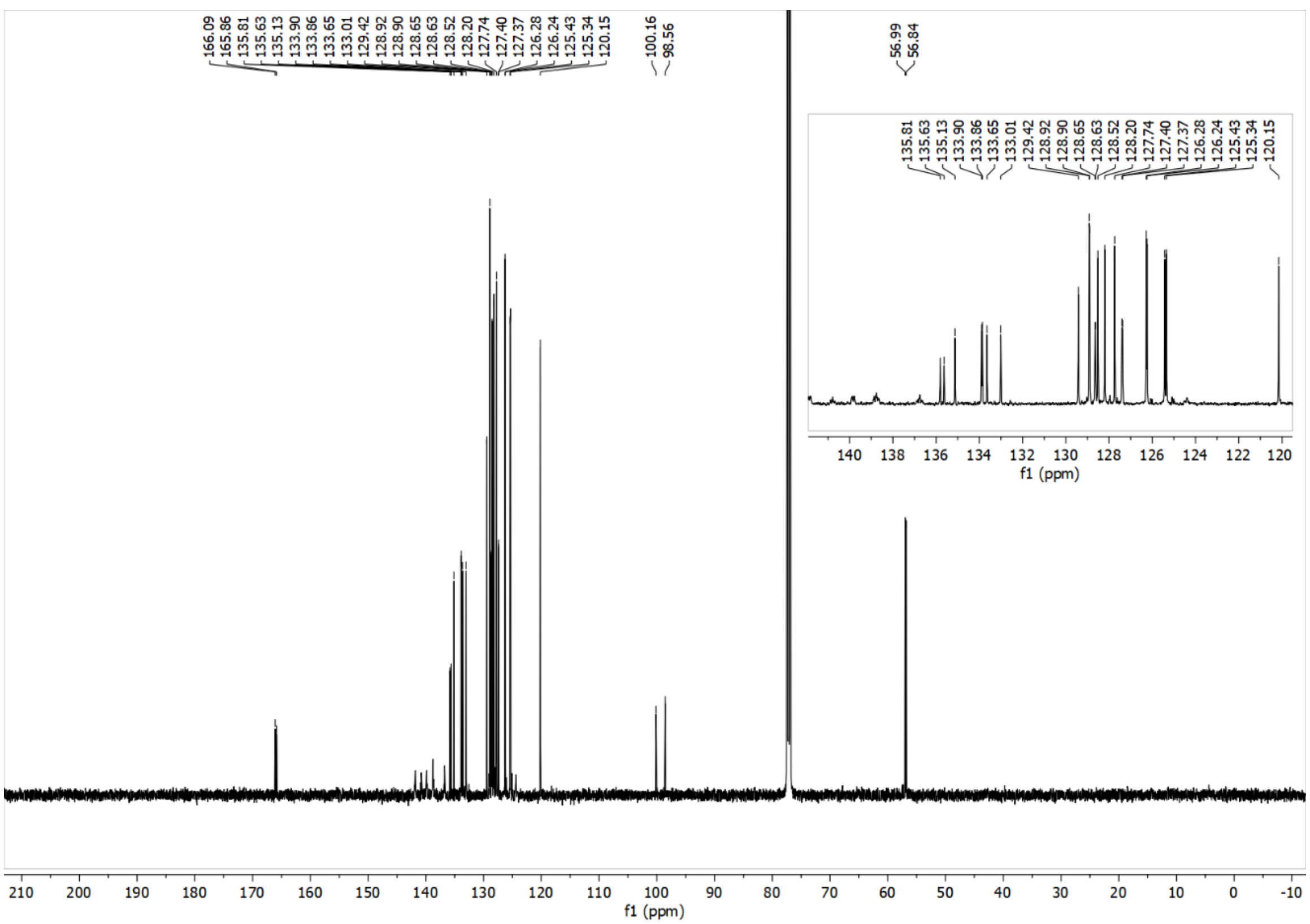

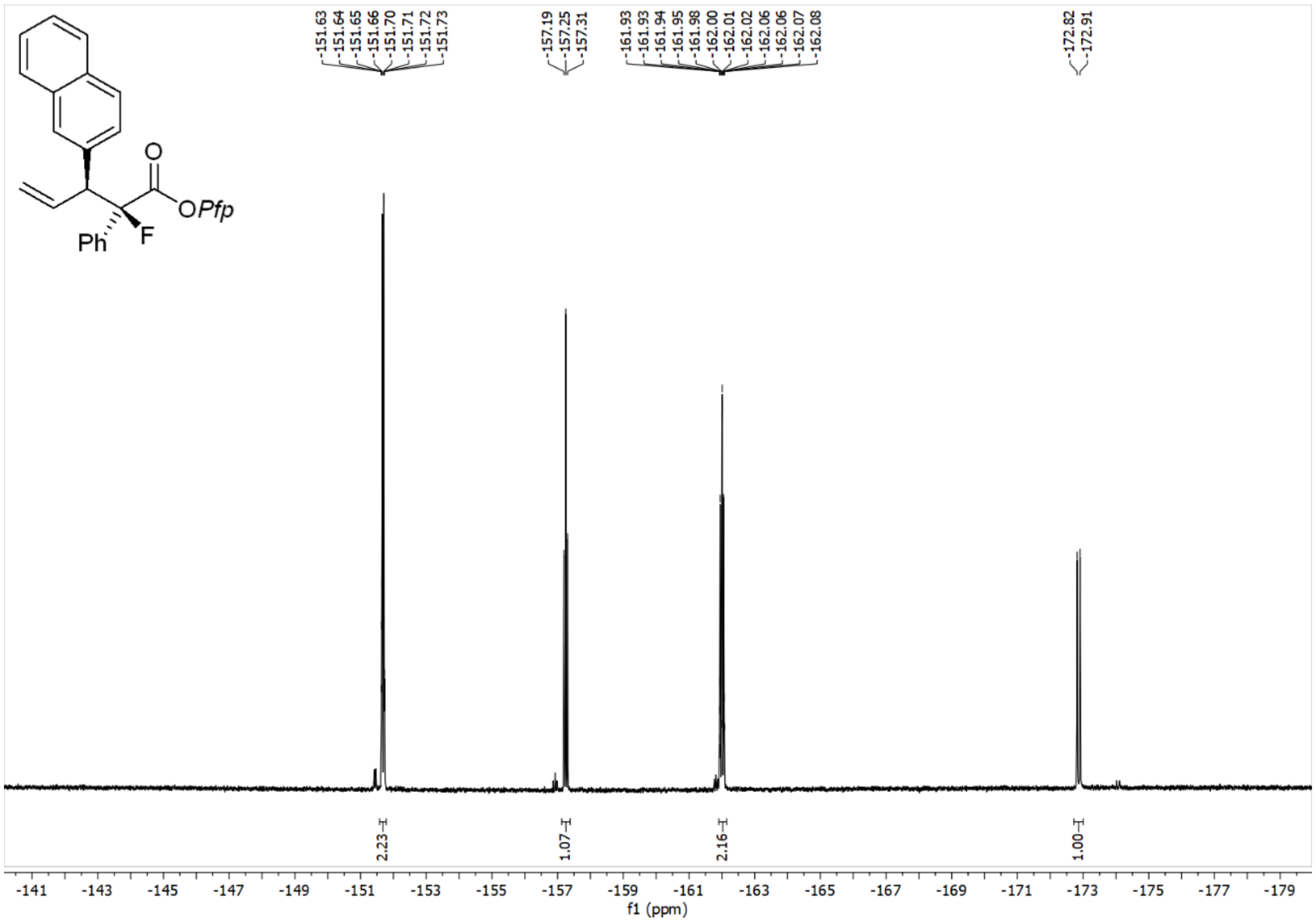
-151.63
-151.64
-151.65
-151.66
-151.70
-151.71
-151.72
-151.73
-157.19
-157.25
-157.31
-161.93
-161.93
-161.94
-161.95
-161.98
-162.00
-162.01
-162.02
-162.06
-162.06
-162.07
-162.08
-172.82
-172.91
OPfp
Ph
F
O
2.23
1.07
2.16
1.00
-141
-143
-145
-147
-149
-151
-153
-155
-157
-159
-161
-163
-165
-167
-169
-171
-173
-175
-177
-179
f1 (ppm)

**Perfluorophenyl (2*R*,3*R*)-3-(benzo[d][1,3]dioxol-5-yl)-2-fluoro-2-phenylpent-4-enoate (*R*,*R*)-23:**

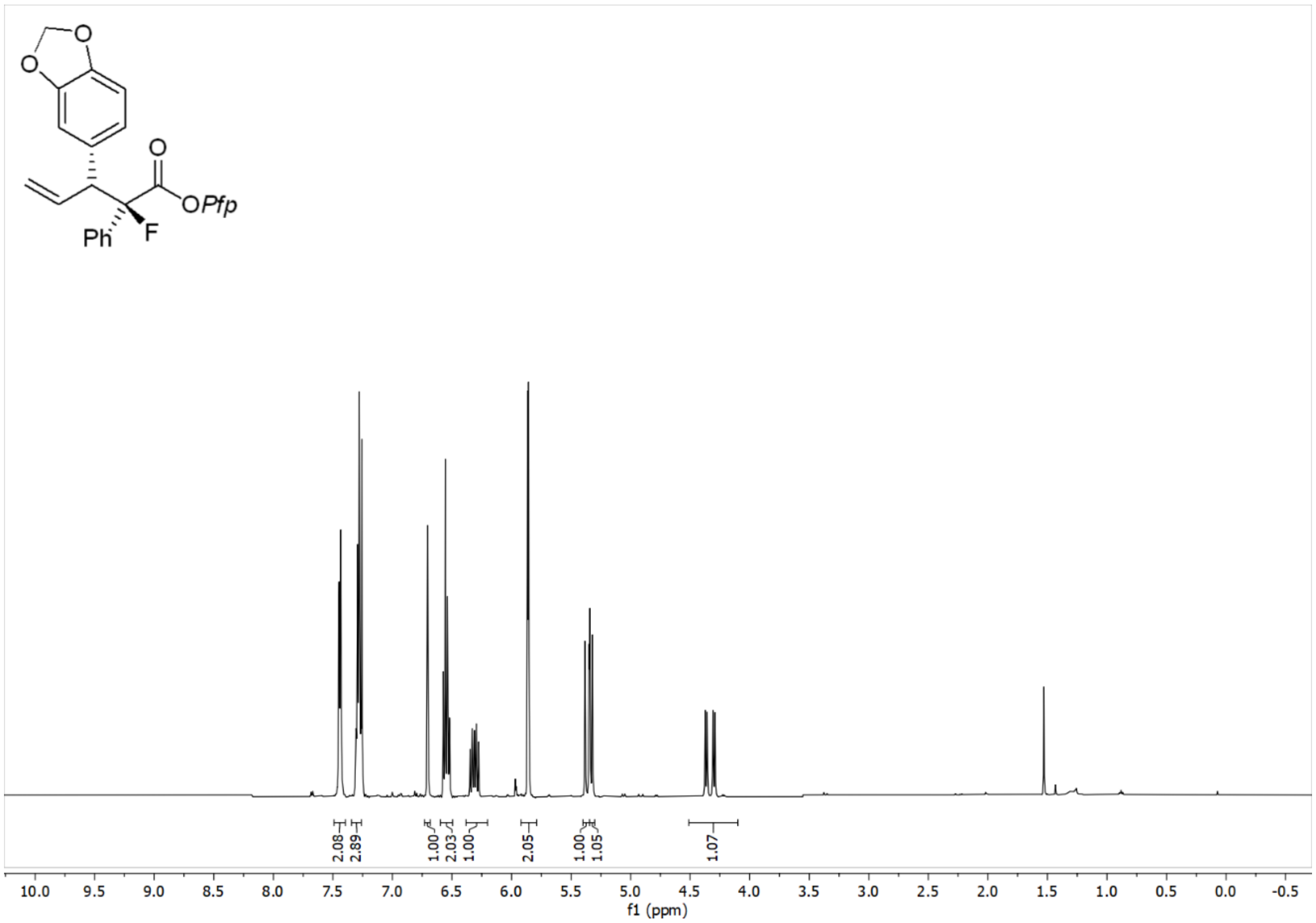


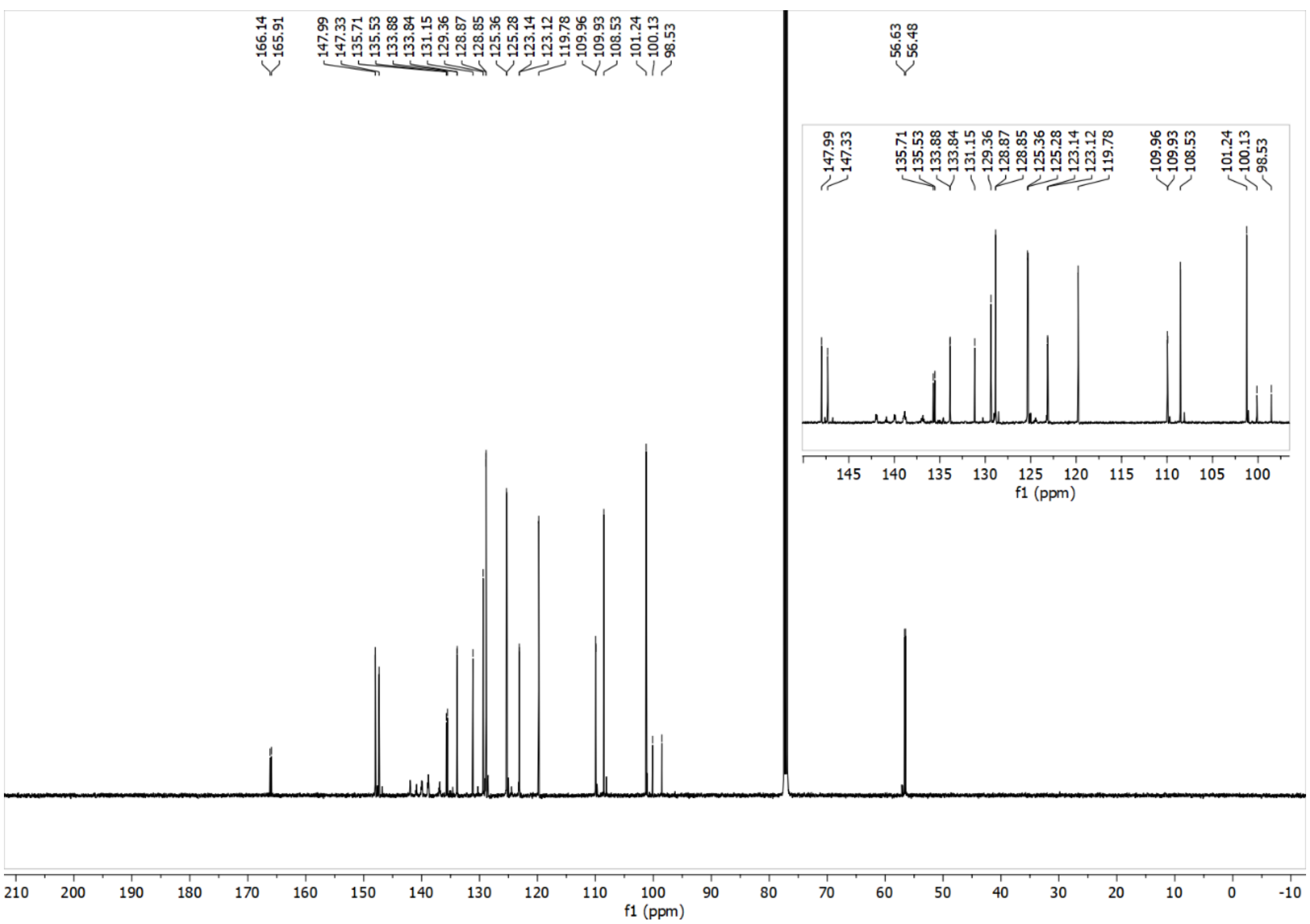

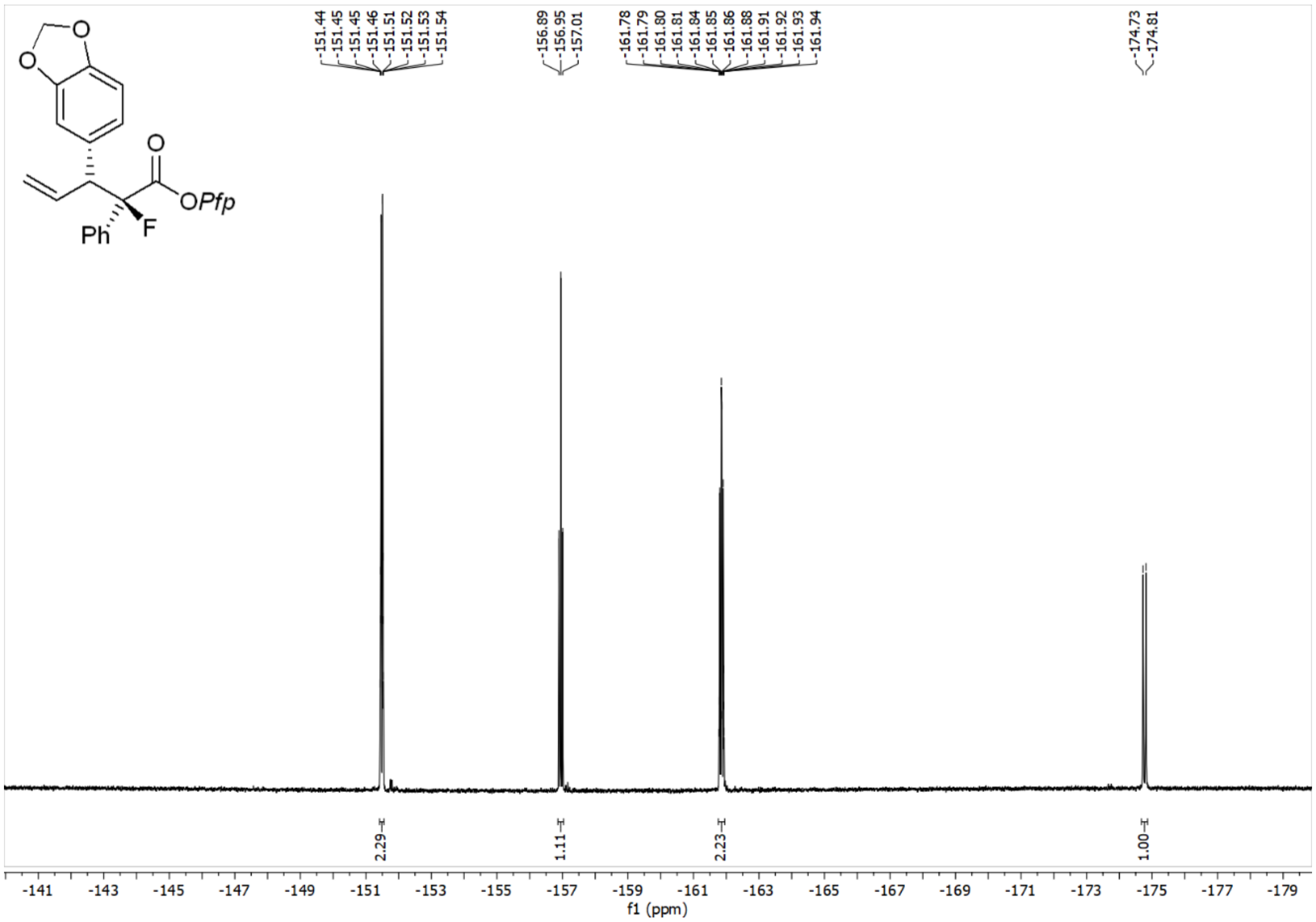

OPfp
Ph
F
-151.44
-151.45
-151.45
-151.46
-151.51
-151.52
-151.53
-151.54
-156.89
-156.95
-157.01
-161.78
-161.79
-161.80
-161.81
-161.84
-161.85
-161.86
-161.88
-161.91
-161.92
-161.93
-161.94
-174.73
-174.81
2.29
1.11
2.23
1.00
-141 -143 -145 -147 -149 -151 -153 -155 -157 -159 -161 -163 -165 -167 -169 -171 -173 -175 -177 -179
f1 (ppm)

**Perfluorophenyl (2*R*,3*S*)-3-(benzo[d][1,3]dioxol-5-yl)-2-fluoro-2-phenylpent-4-enoate (*R*,*S*)-23:**

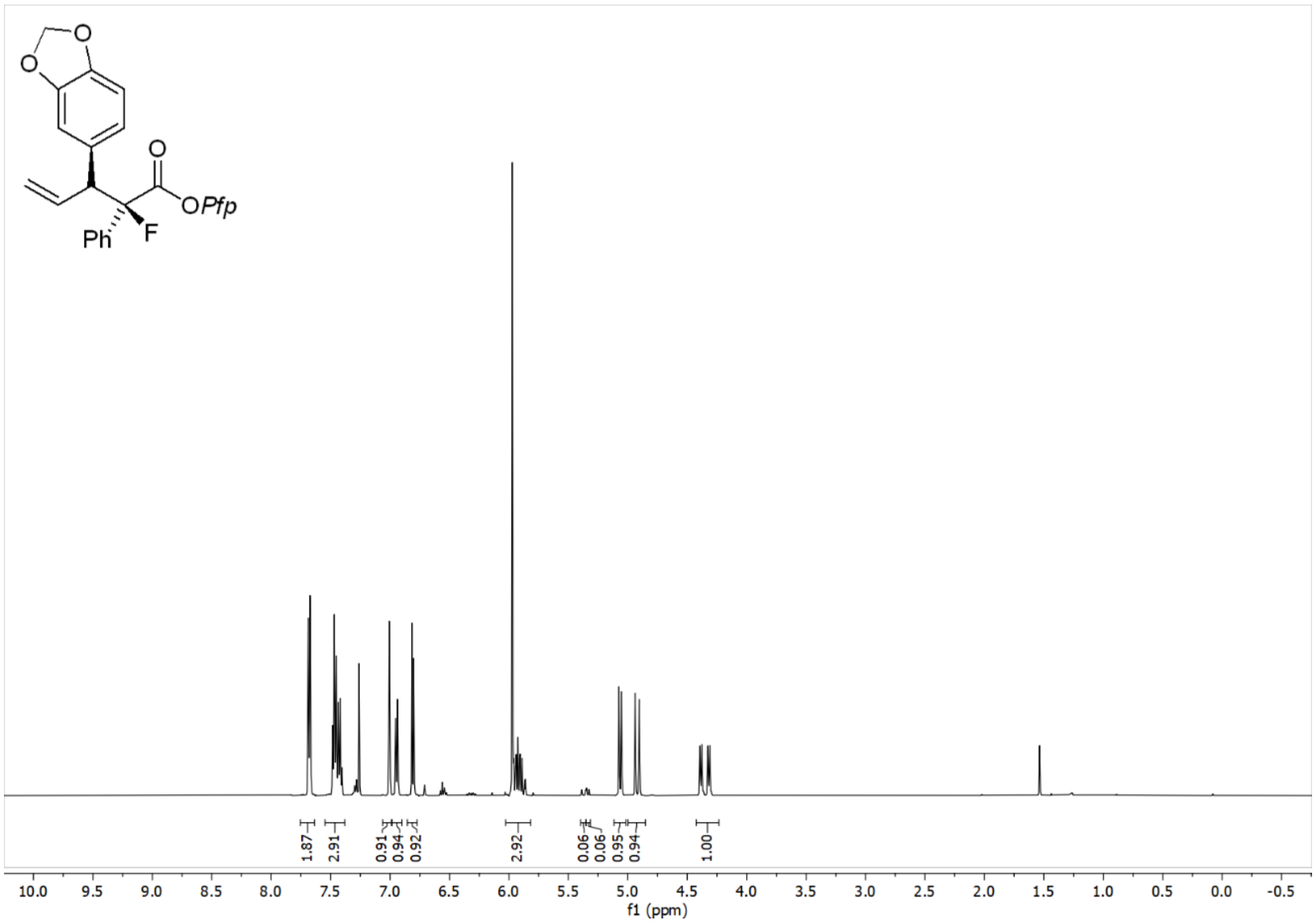


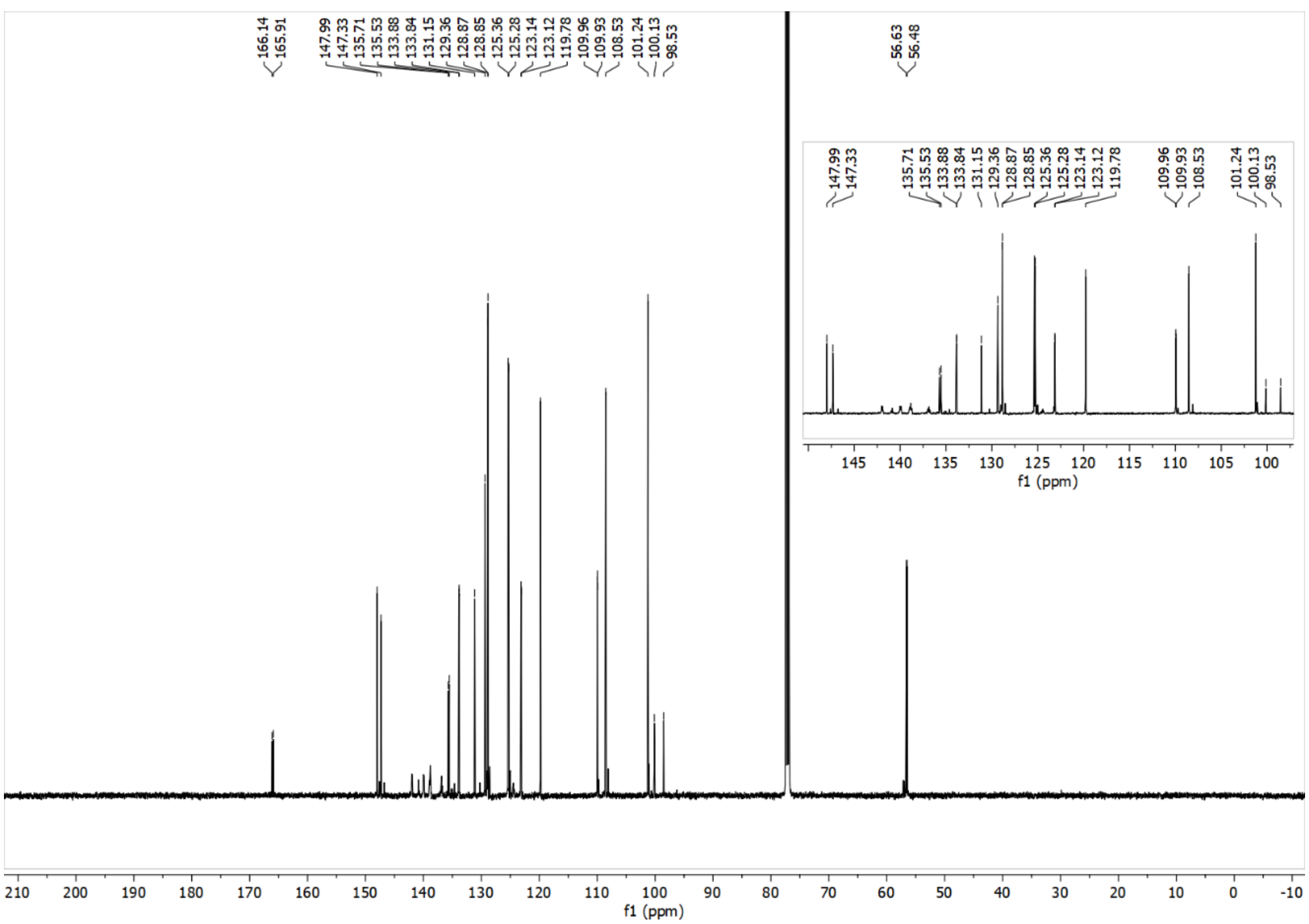

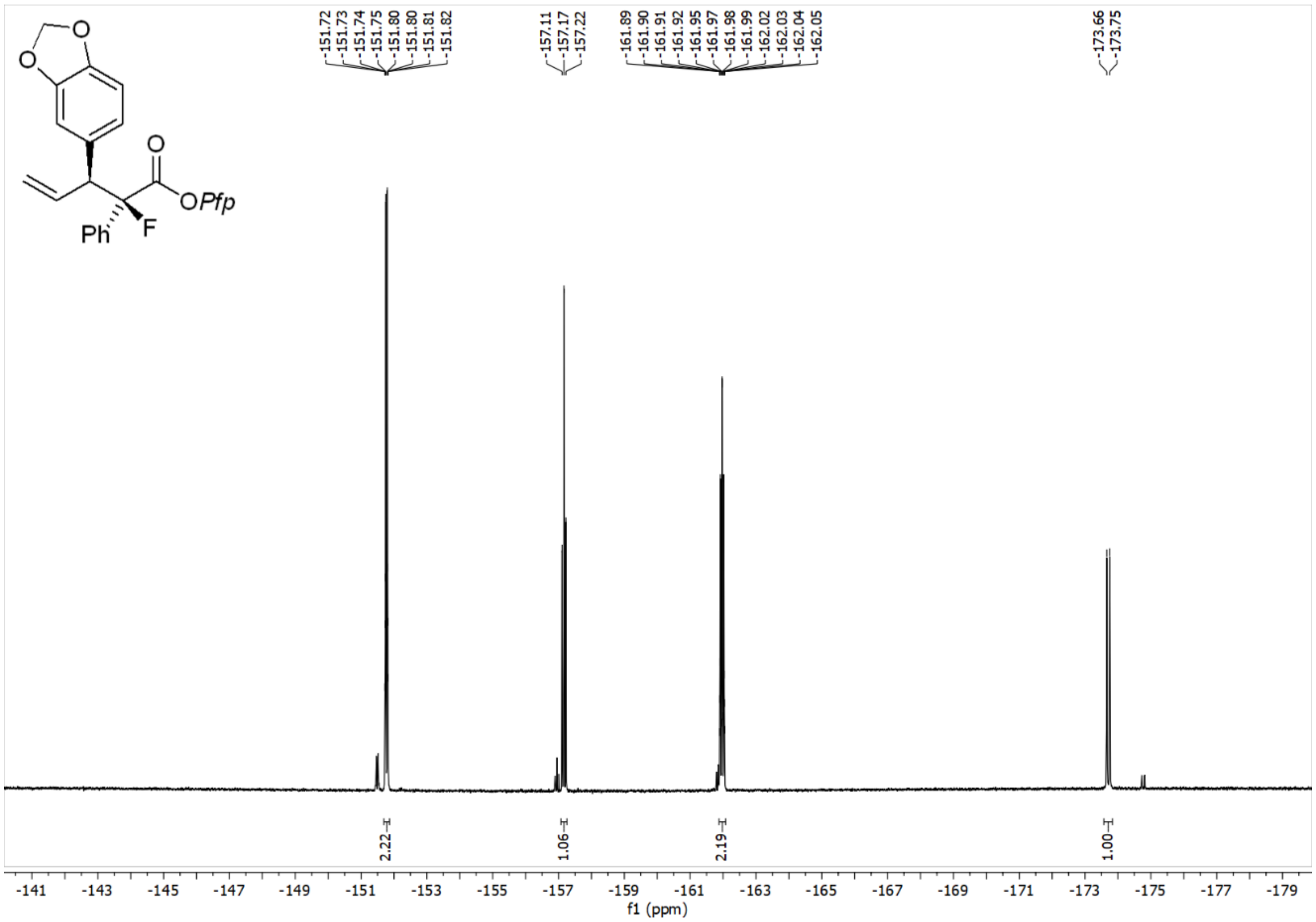
OPfp
Ph
F
-151.72
-151.73
-151.74
-151.75
-151.80
-151.80
-151.81
-151.82
-157.11
-157.17
-157.22
-161.89
-161.90
-161.91
-161.92
-161.95
-161.97
-161.98
-161.99
-162.02
-162.03
-162.04
-162.05
-173.66
-173.75
2.22
1.06
2.19
1.00
-141 -143 -145 -147 -149 -151 -153 -155 -157 -159 -161 -163 -165 -167 -169 -171 -173 -175 -177 -179
f1 (ppm)

**Perfluorophenyl (2*R*,3*S*)-2-fluoro-2-phenyl-3-(thiophen-2-yl)pent-4-enoate (*R*,*S*)-25:**

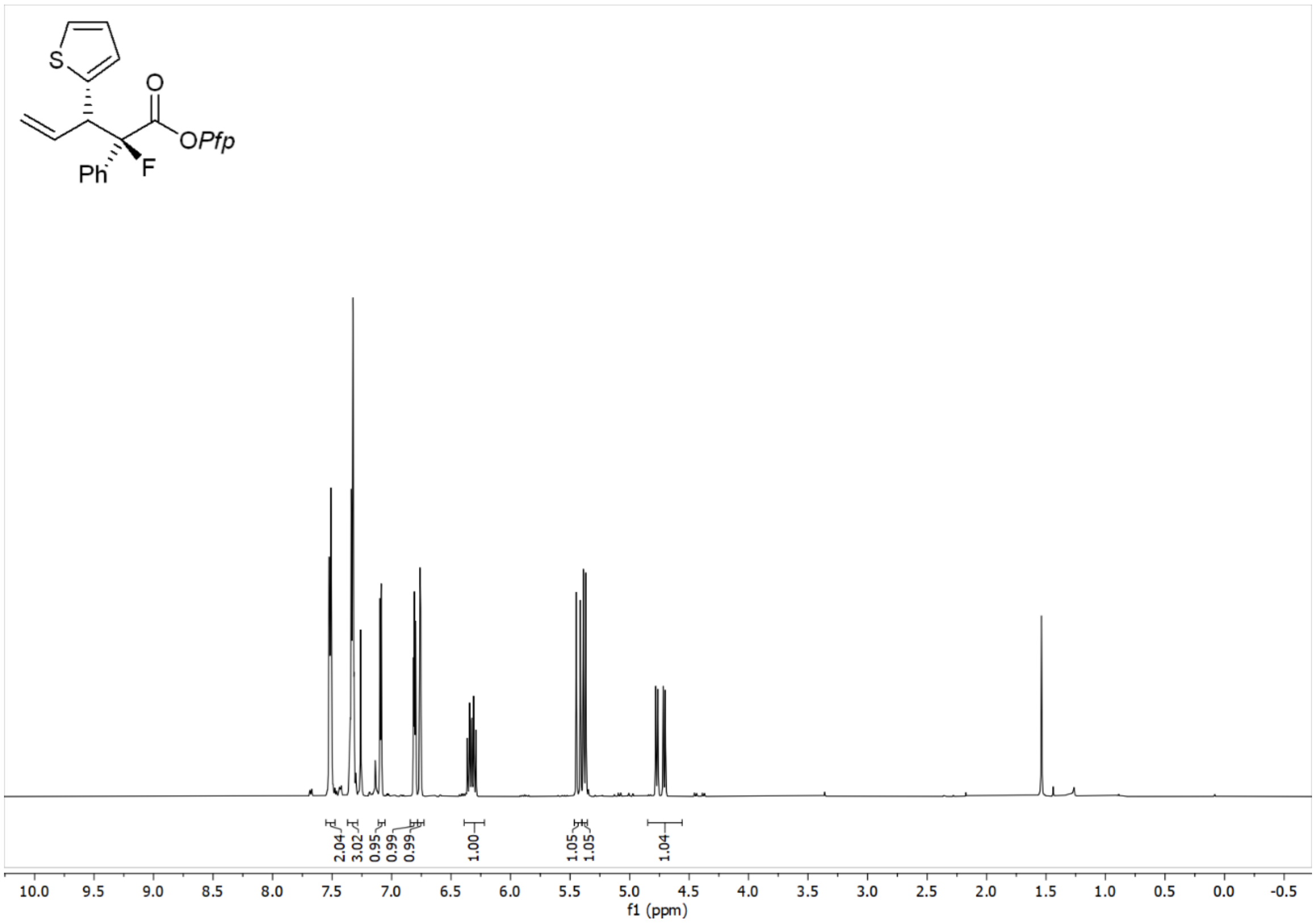


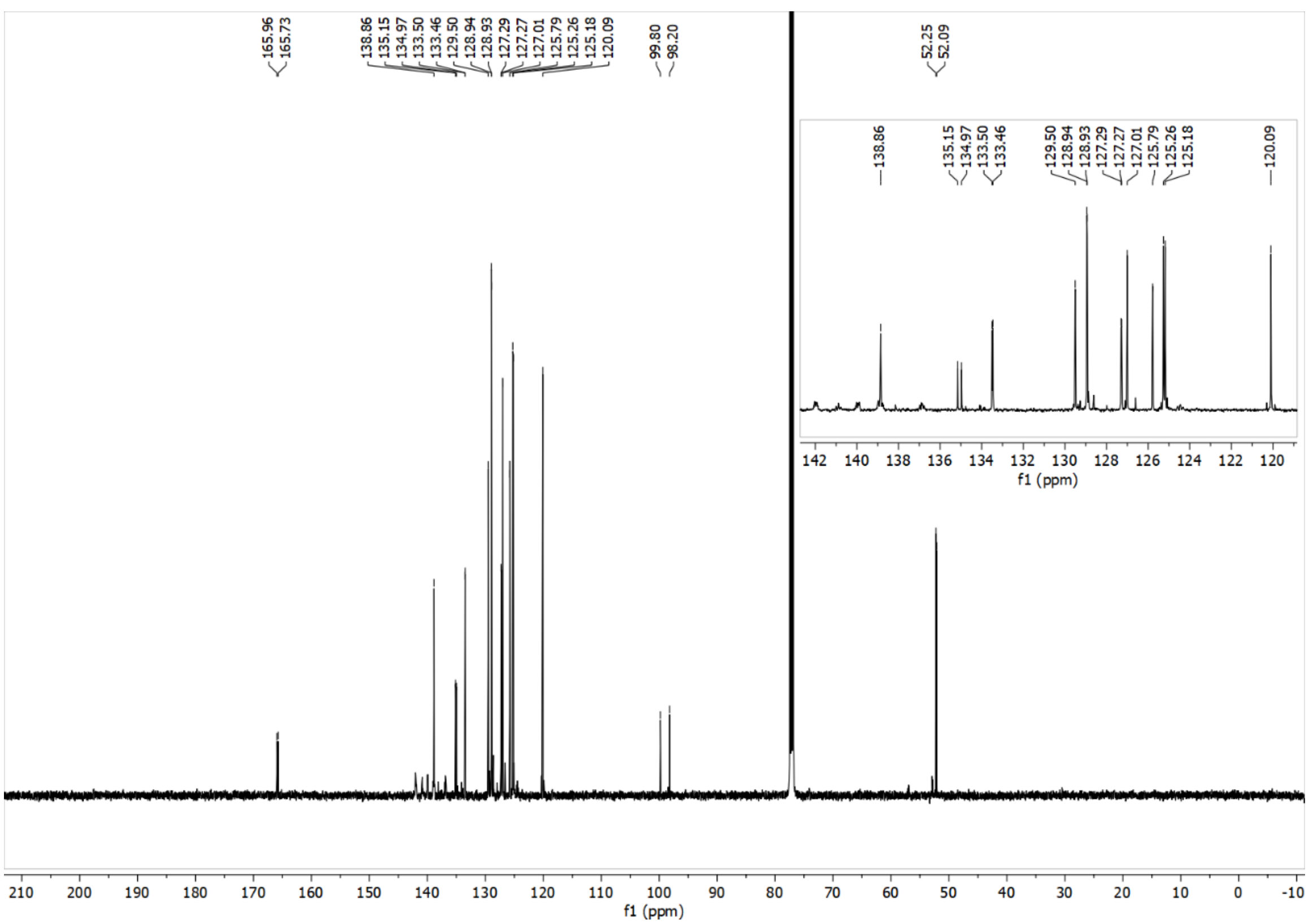

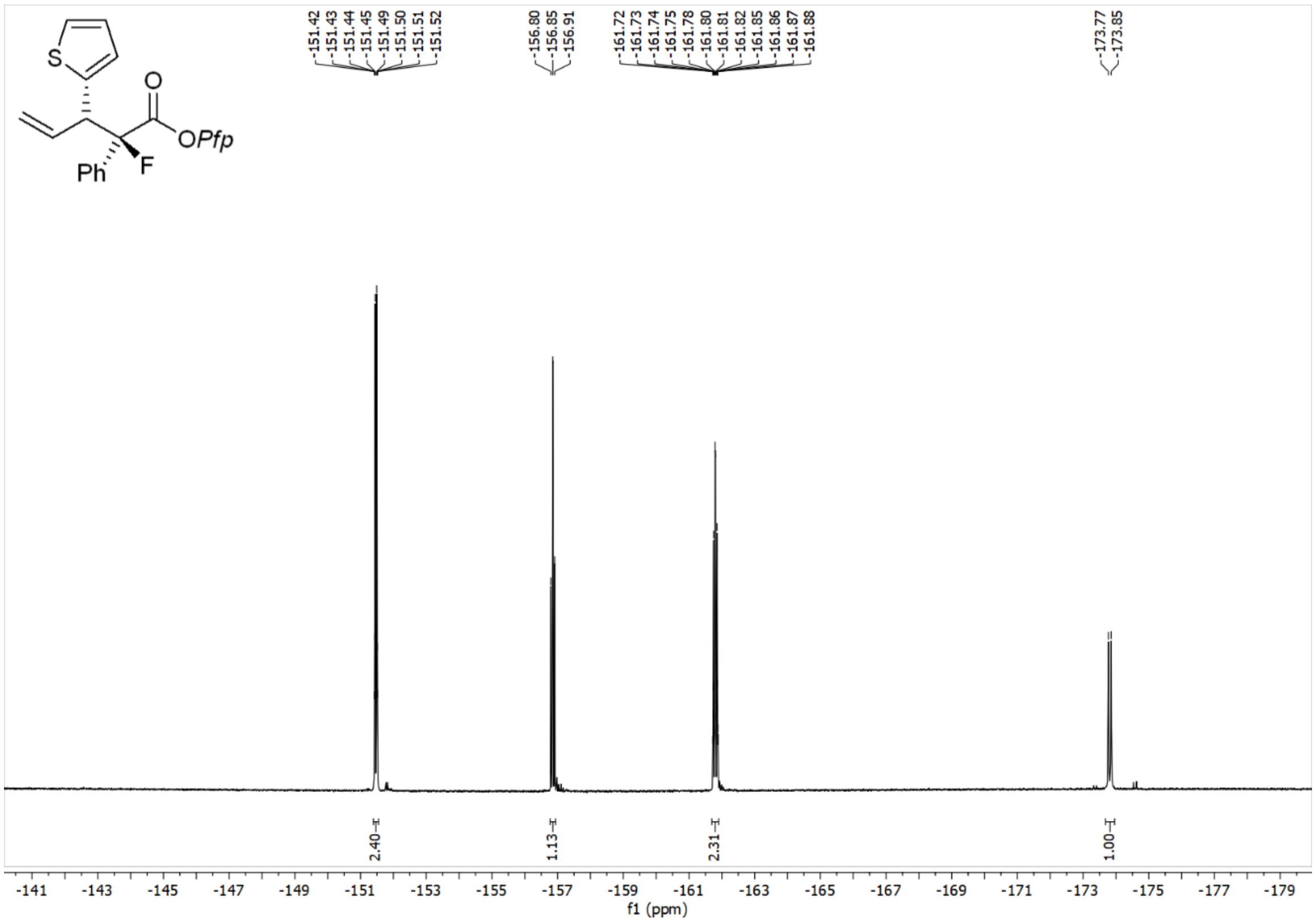
S
O
OPfp
Ph
F
-151.42
-151.43
-151.44
-151.45
-151.49
-151.50
-151.51
-151.52
-156.80
-156.85
-156.91
-161.72
-161.73
-161.74
-161.75
-161.78
-161.80
-161.81
-161.82
-161.85
-161.86
-161.87
-161.88
-173.77
-173.85
2.40
1.13
2.31
1.00
-141
-143
-145
-147
-149
-151
-153
-155
-157
-159
-161
-163
-165
-167
-169
-171
-173
-175
-177
-179
f1 (ppm)

**Perfluorophenyl (2*R*,3*R*)-2-fluoro-2-phenyl-3-(thiophen-2-yl)pent-4-enoate (*R*,*R*)-25:**

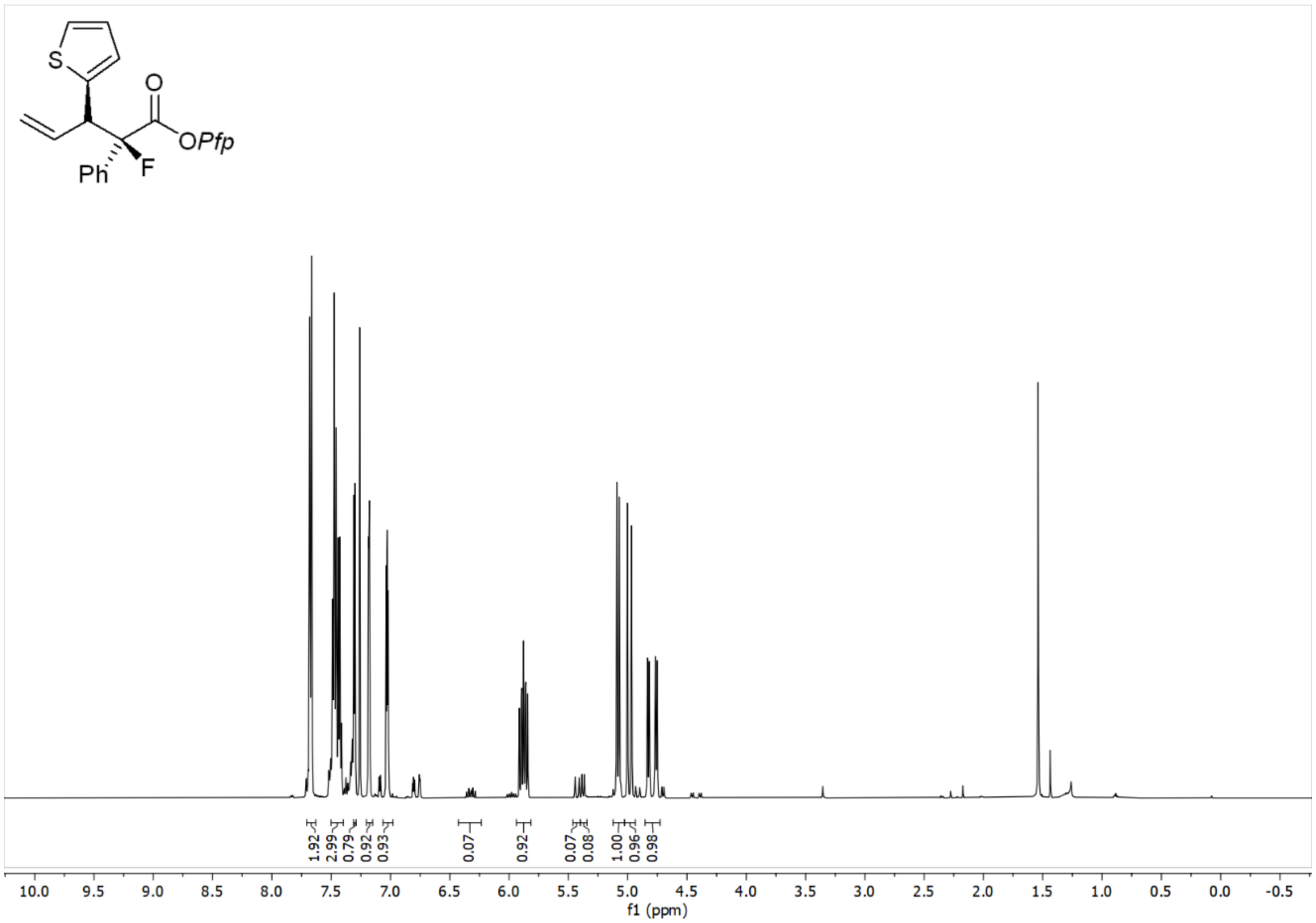


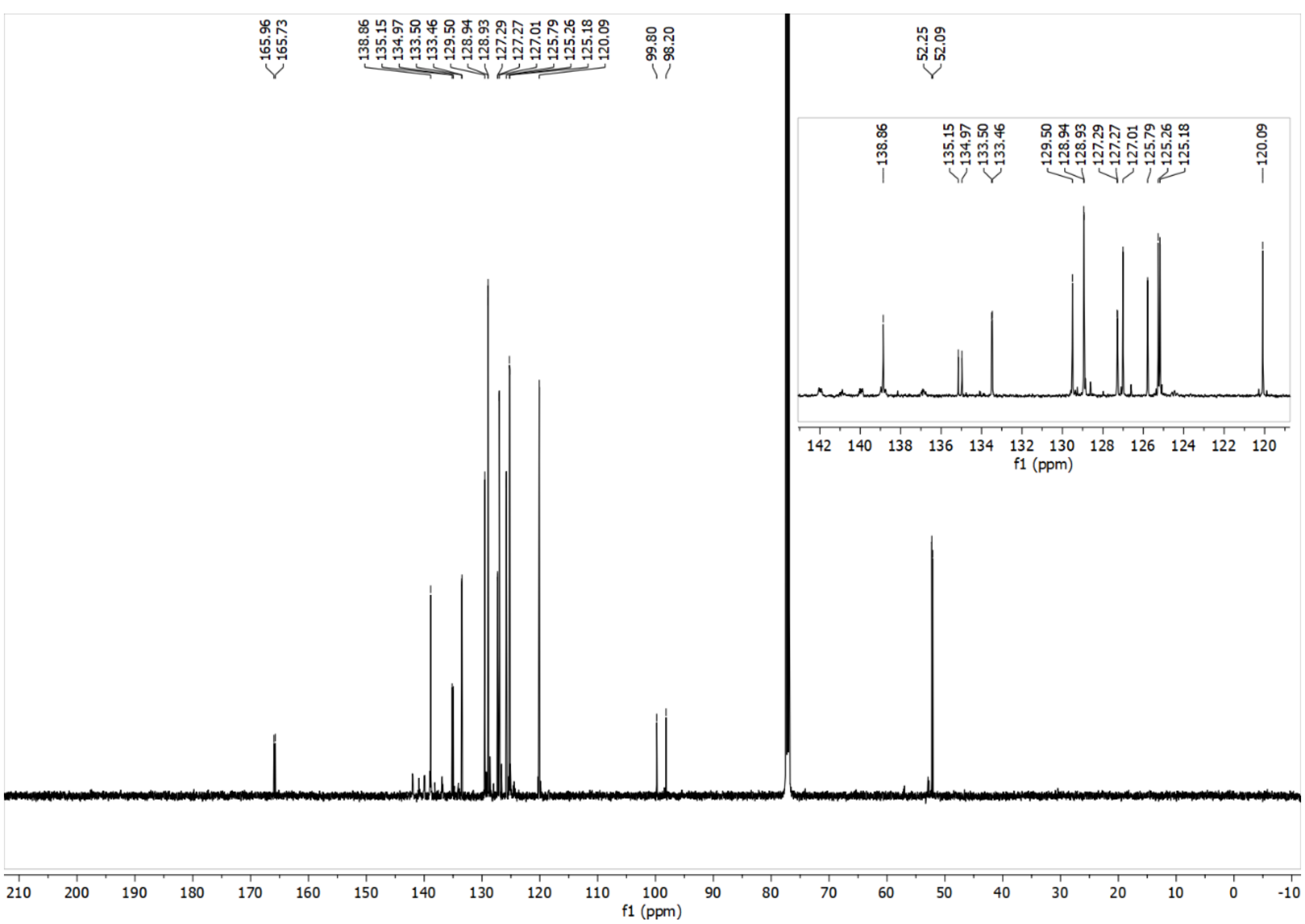

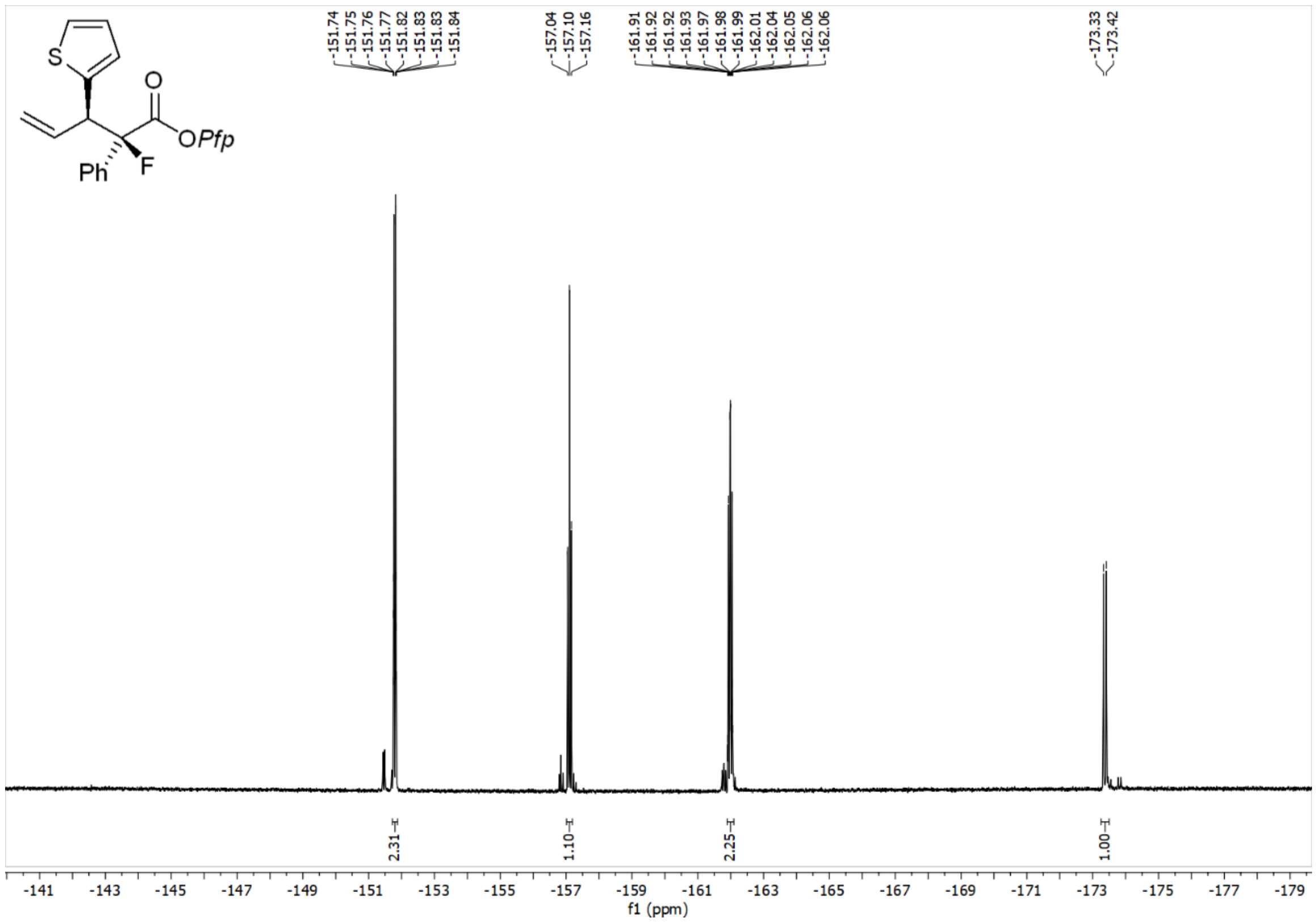

OPfp
Ph
F
S
-151.74
-151.75
-151.76
-151.77
-151.82
-151.83
-151.83
-151.84
-157.04
-157.10
-157.16
-161.91
-161.92
-161.92
-161.93
-161.97
-161.98
-161.99
-162.01
-162.04
-162.05
-162.06
-162.06
-173.33
-173.42
2.31
1.10
2.25
1.00
f1 (ppm)

**Perfluorophenyl (2*R*,3*R*)-2-fluoro-3-(furan-3-yl)-2-phenylpent-4-enoate (*R*,*R*)-24**

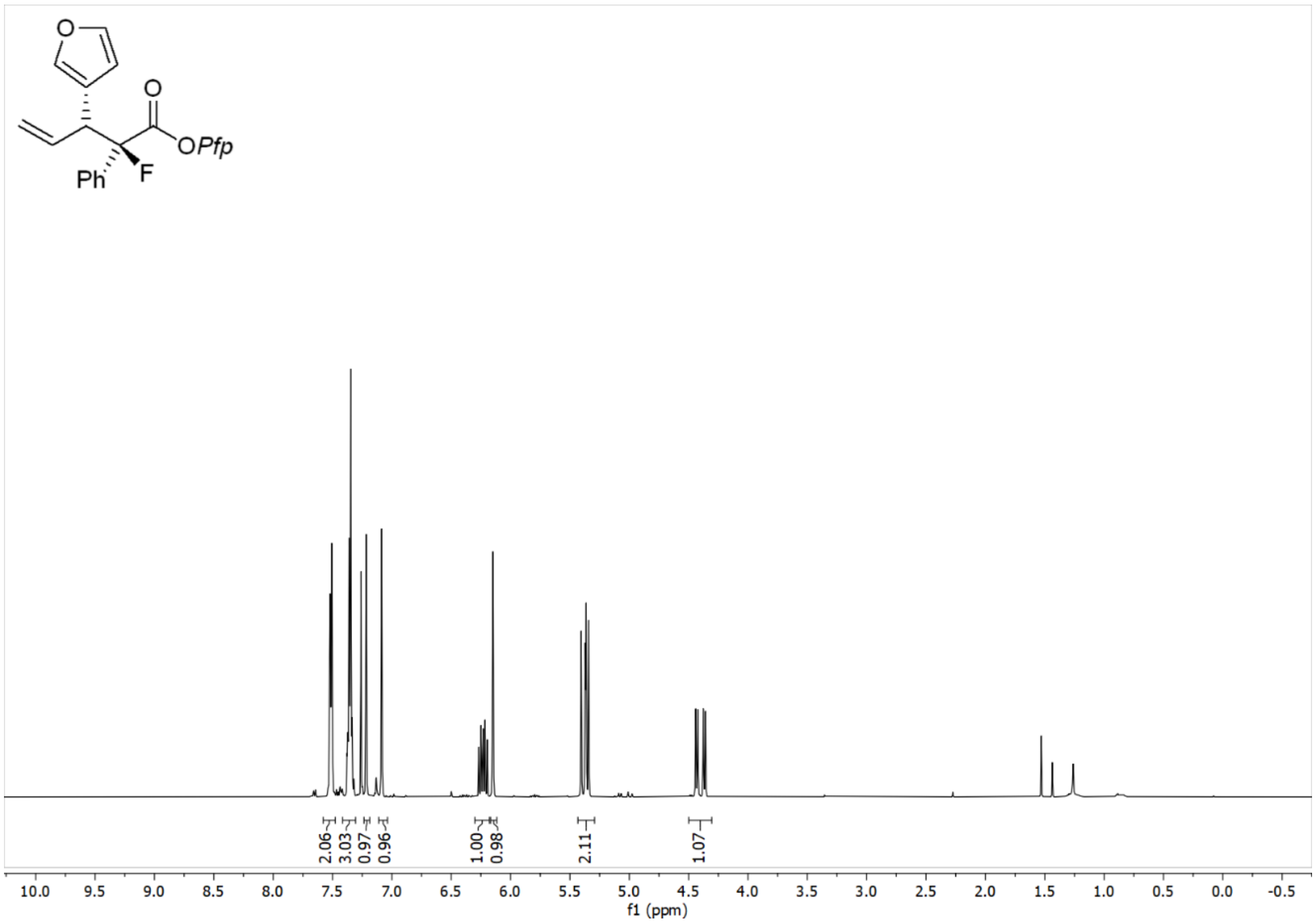


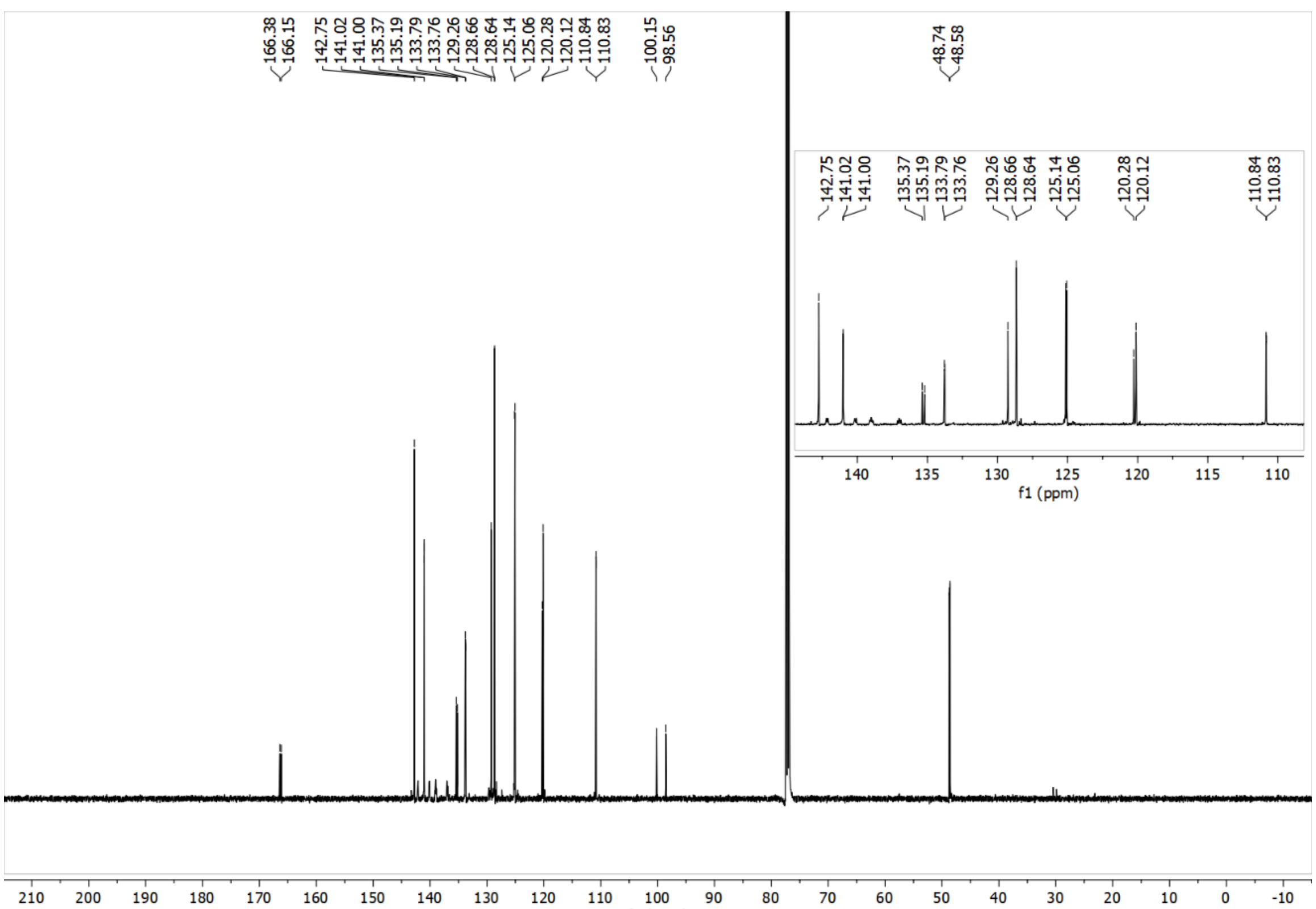

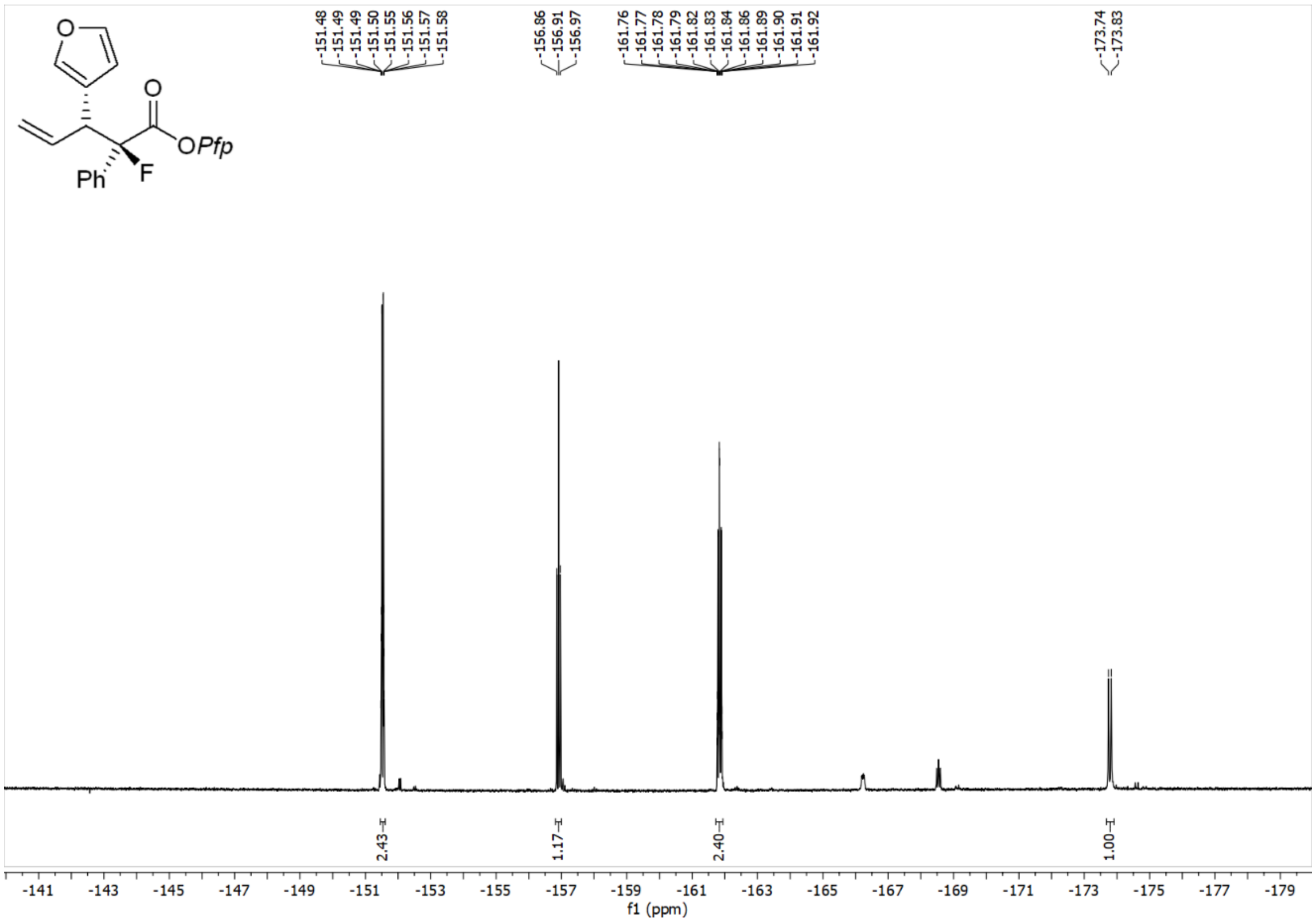
OPfp
Ph
F
-151.48
-151.49
-151.49
-151.50
-151.55
-151.56
-151.57
-151.58
-156.86
-156.91
-156.97
-161.76
-161.77
-161.78
-161.79
-161.82
-161.83
-161.84
-161.86
-161.89
-161.90
-161.91
-161.92
-173.74
-173.83
2.43
1.17
2.40
1.00
f1 (ppm)

**Perfluorophenyl (2*R*,3*S*)-2-fluoro-3-(furan-3-yl)-2-phenylpent-4-enoate (*R*,*S*)-24**

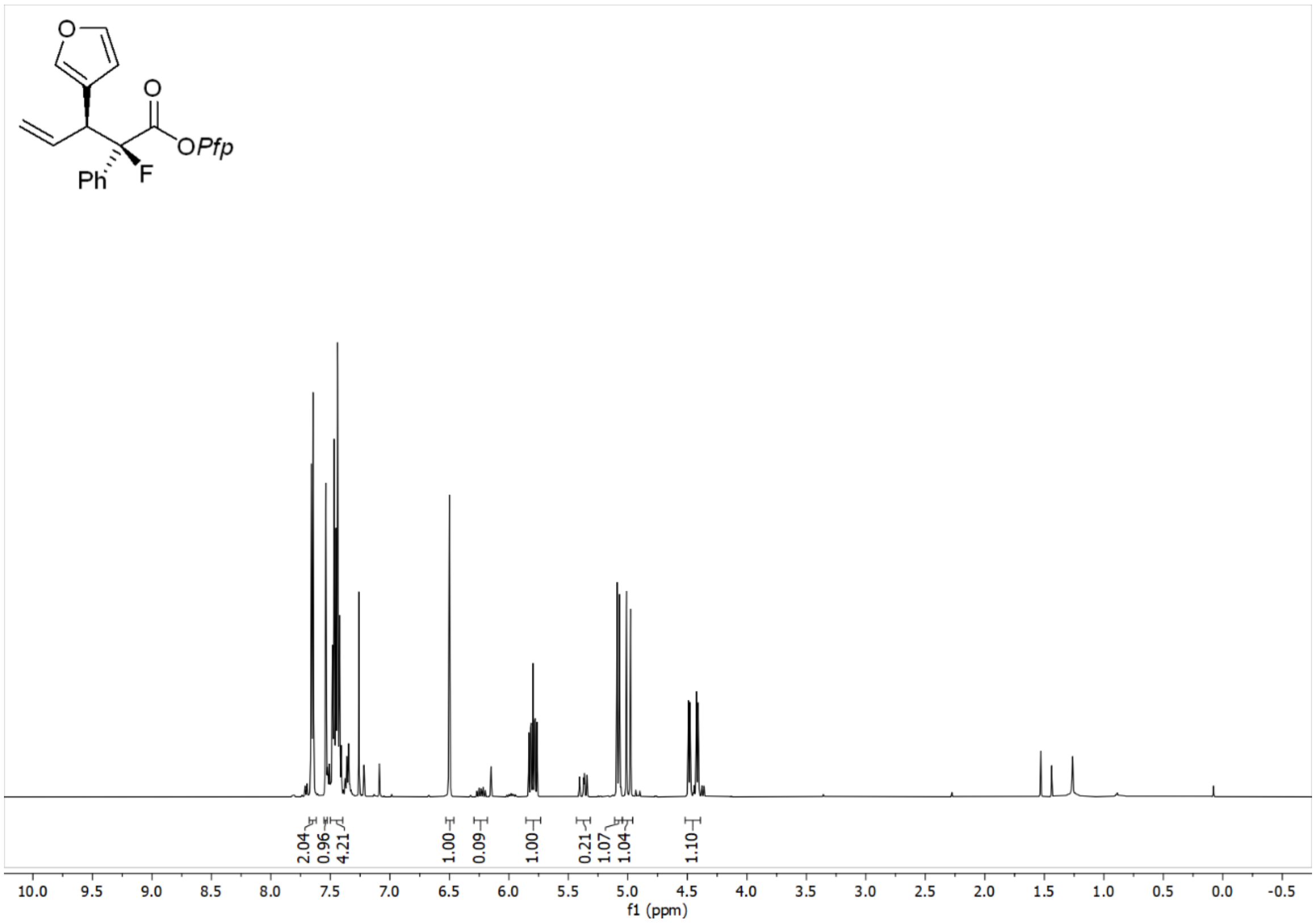


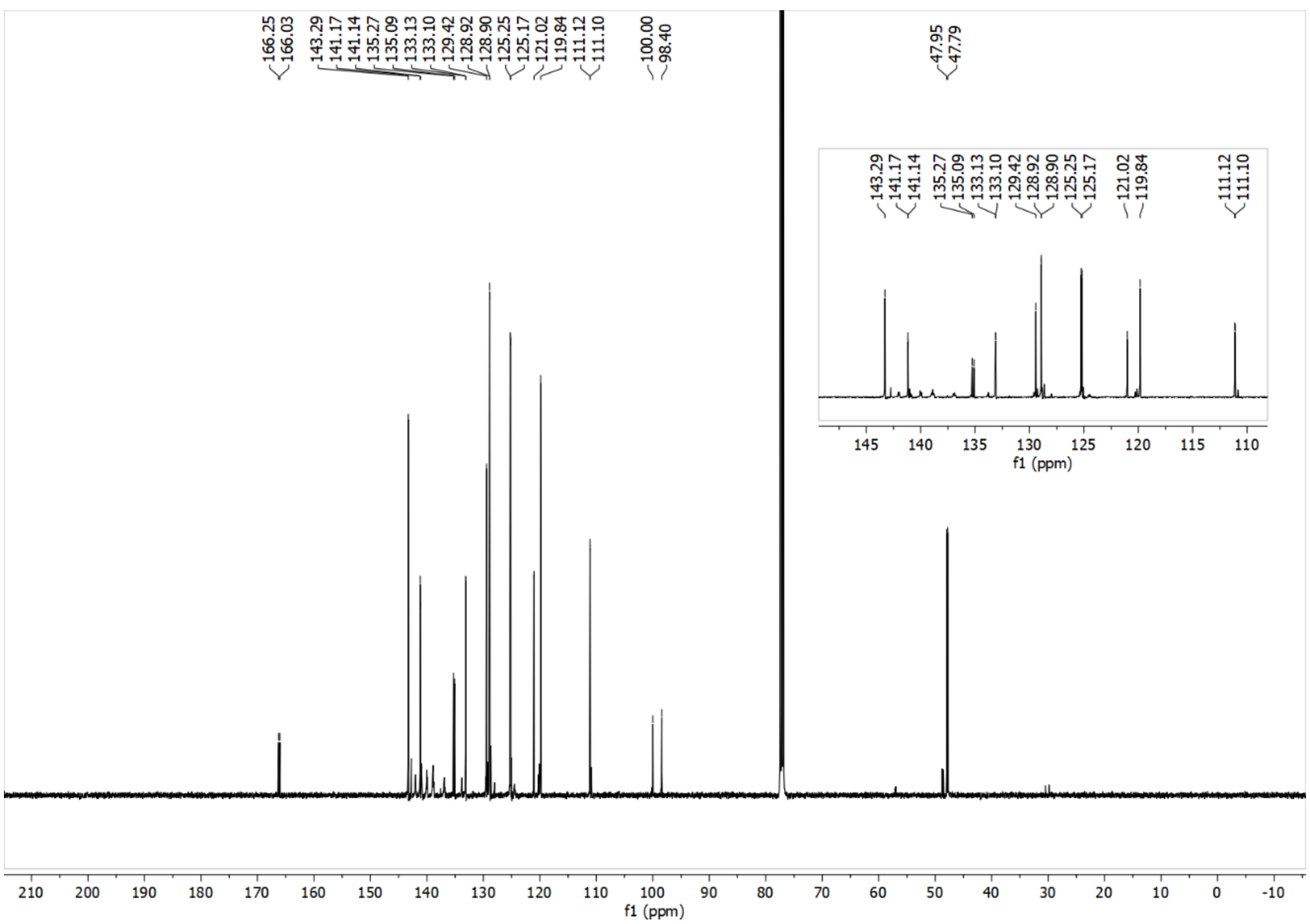

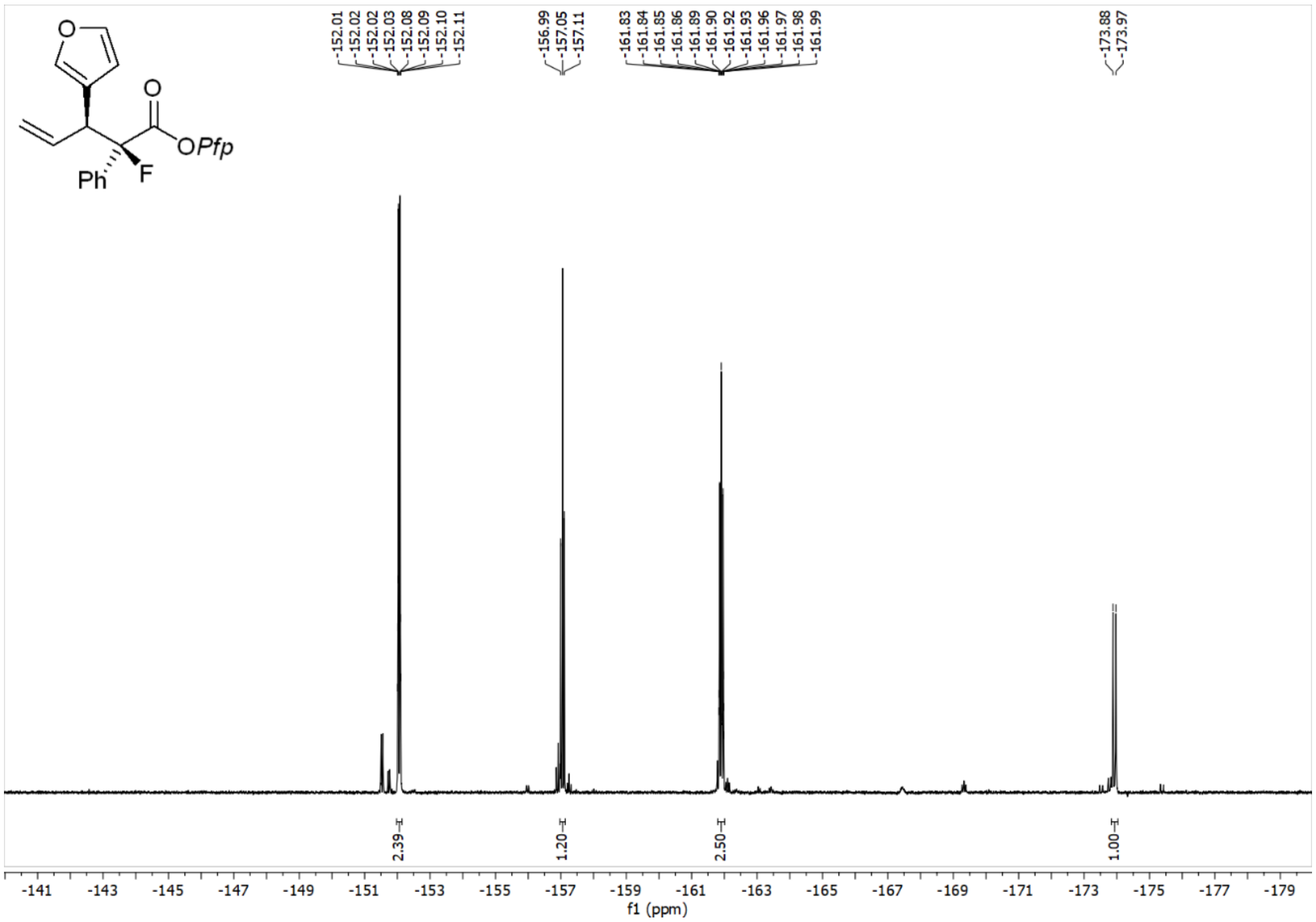
-152.01
-152.02
-152.02
-152.03
-152.08
-152.09
-152.10
-152.11
-156.99
-157.05
-157.11
-161.83
-161.84
-161.85
-161.86
-161.89
-161.90
-161.92
-161.93
-161.96
-161.97
-161.98
-161.99
-173.88
-173.97
2.39
1.20
2.50
1.00
-141
-143
-145
-147
-149
-151
-153
-155
-157
-159
-161
-163
-165
-167
-169
-171
-173
-175
-177
-179
f1 (ppm)

**Perfluorophenyl (2*R*,3*R*)-2-fluoro-2-phenyl-3-(1-tosyl-1H-indol-3-yl)pent-4-enoate (*R*,*R*)-26:**

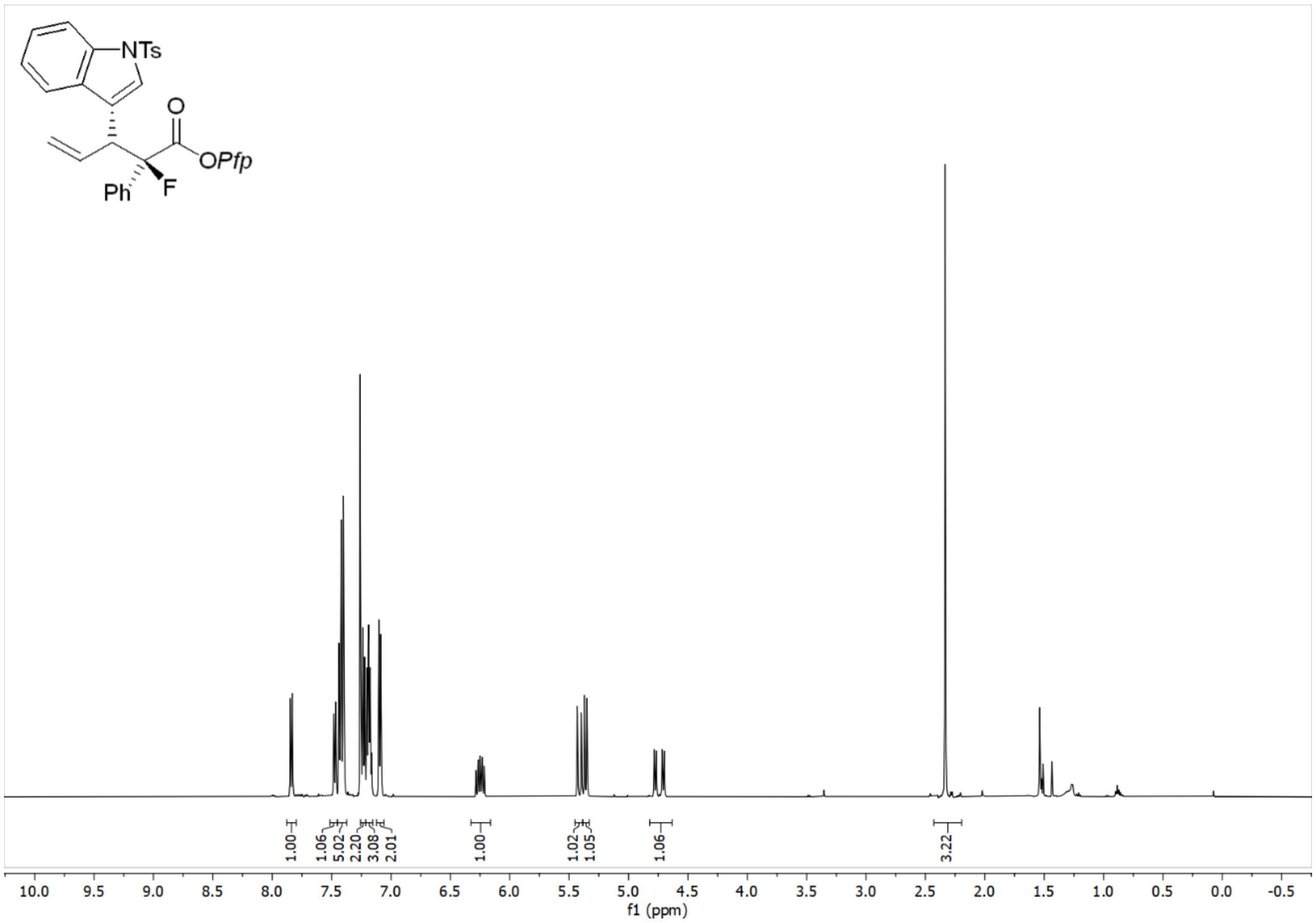


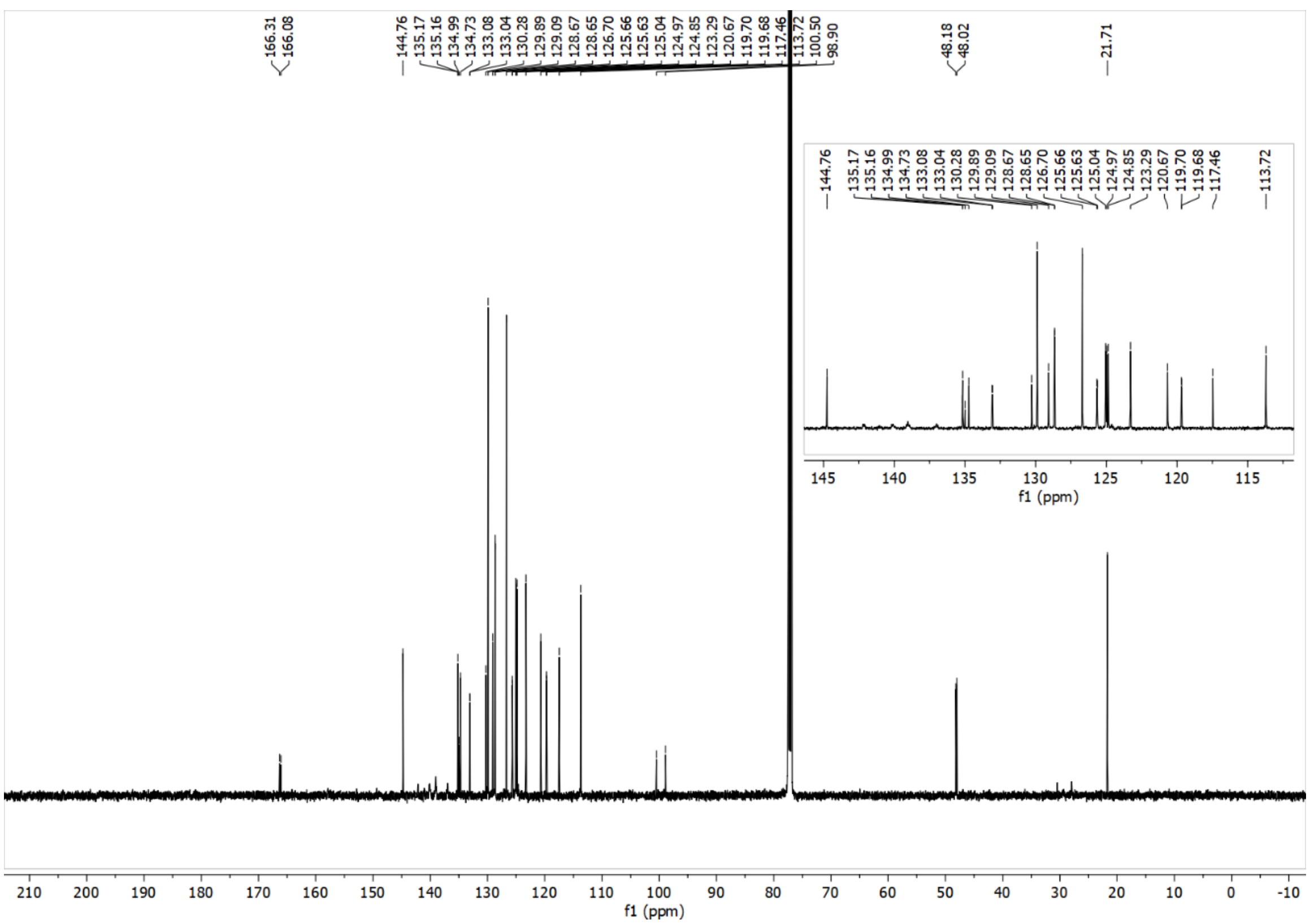

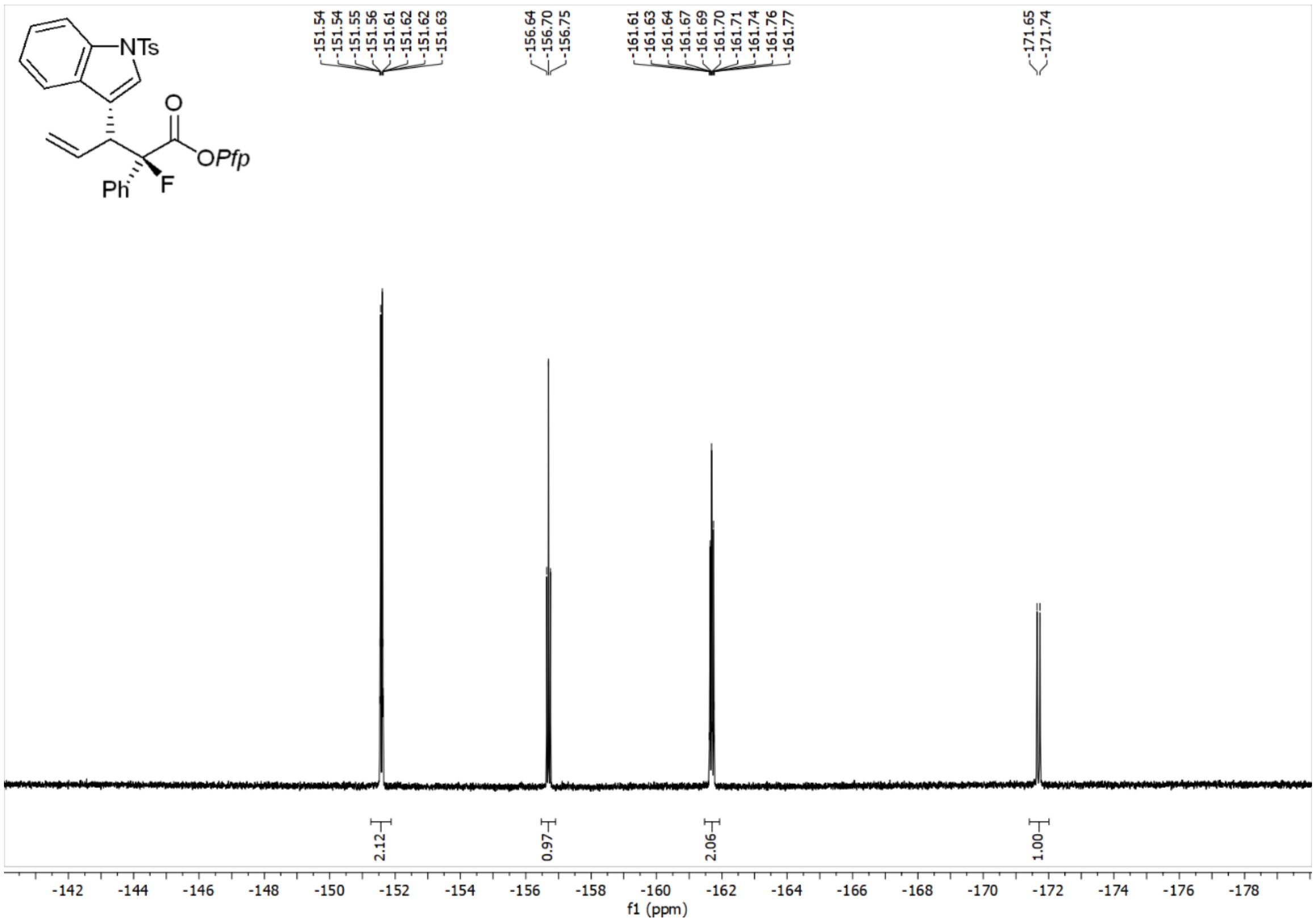
NTs
OPfp
Ph
F
-151.54
-151.54
-151.55
-151.56
-151.61
-151.62
-151.62
-151.63
-156.64
-156.70
-156.75
-161.61
-161.63
-161.64
-161.67
-161.69
-161.70
-161.71
-161.74
-161.76
-161.77
-171.65
-171.74
2.12
0.97
2.06
1.00
-142
-144
-146
-148
-150
-152
-154
-156
-158
-160
-162
-164
-166
-168
-170
-172
-174
-176
-178
f1 (ppm)

**Perfluorophenyl (2*R*,3*S*)-2-fluoro-2-phenyl-3-(1-tosyl-1H-indol-3-yl)pent-4-enoate (*R*,*S*)-26):**

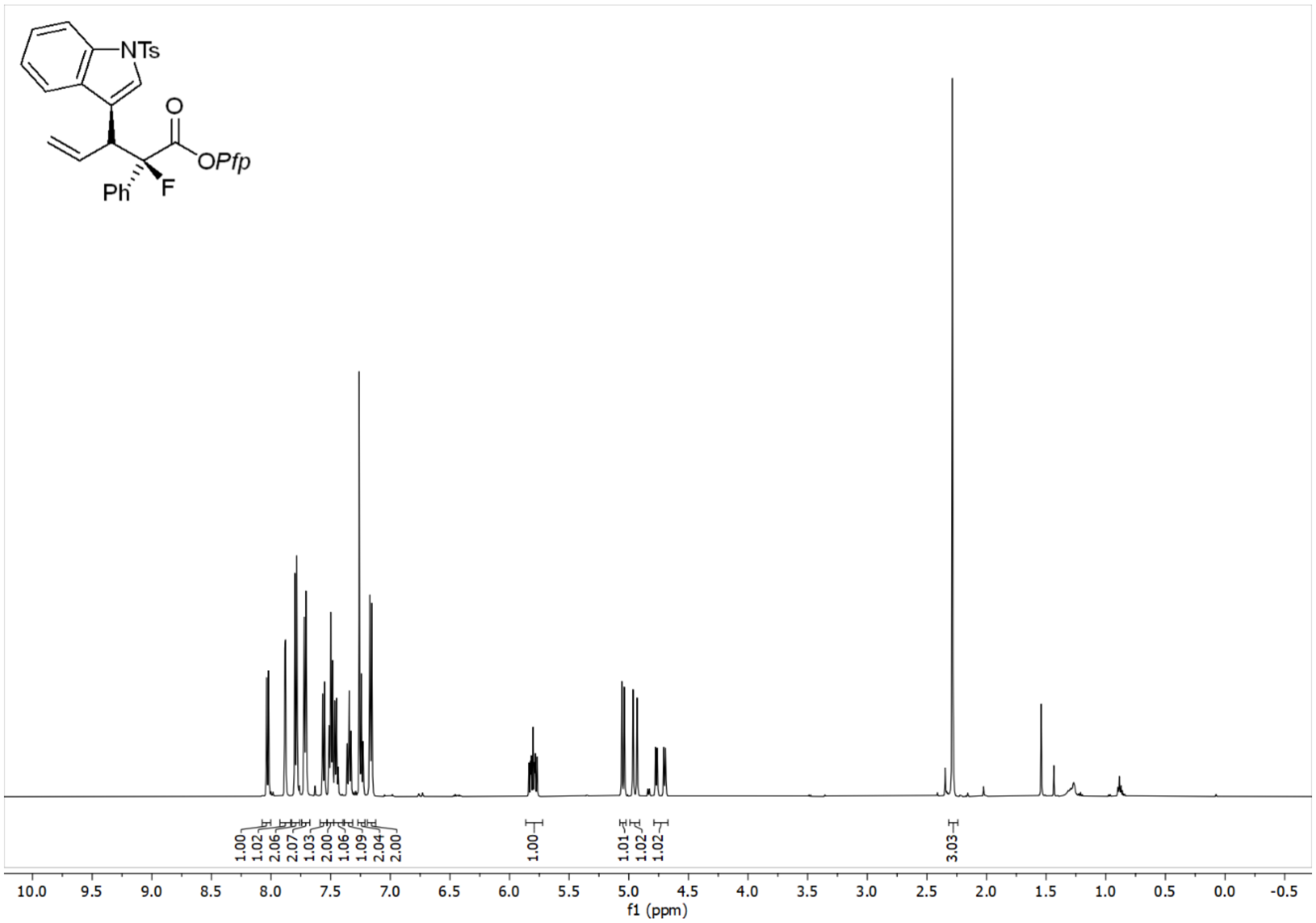


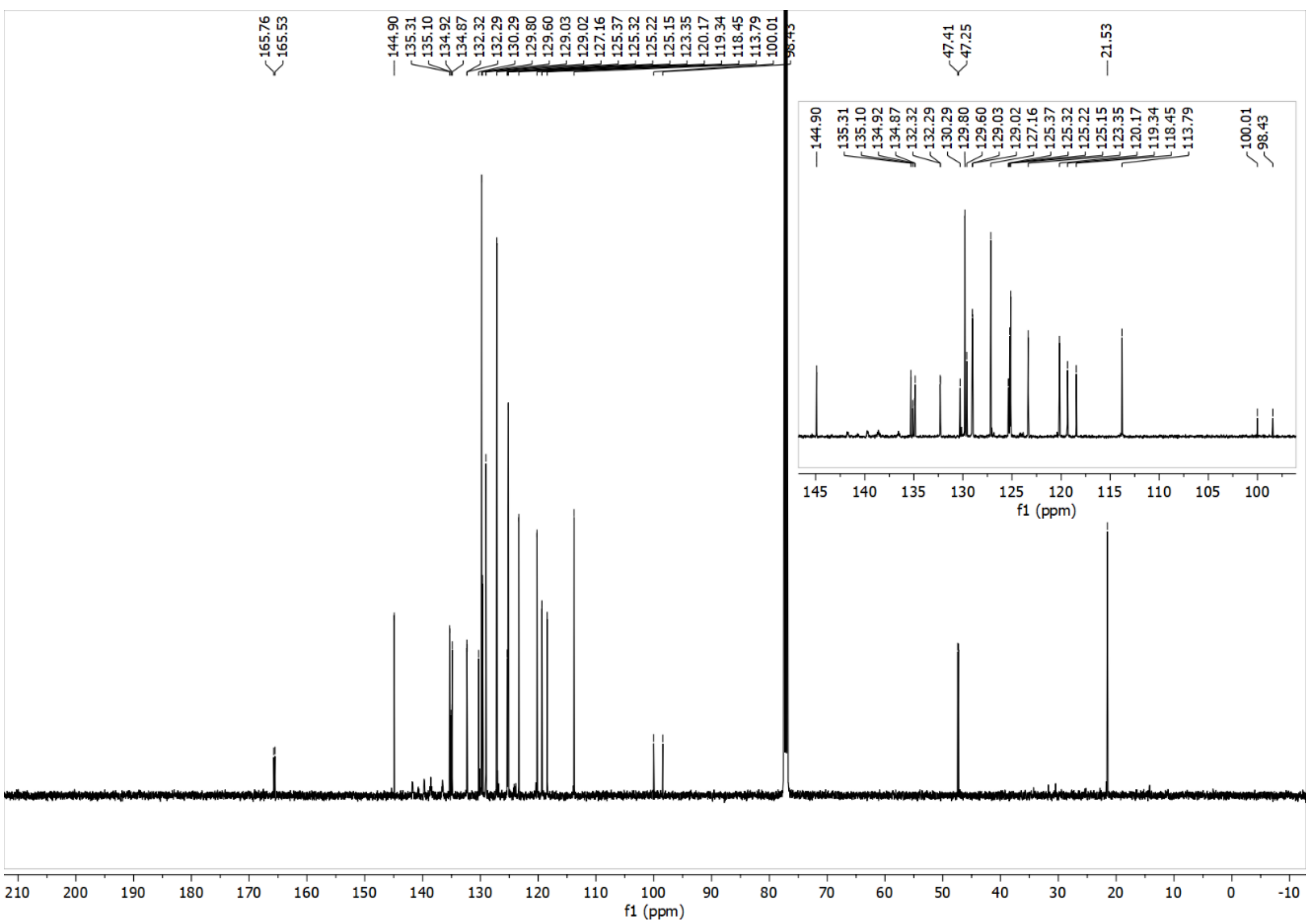

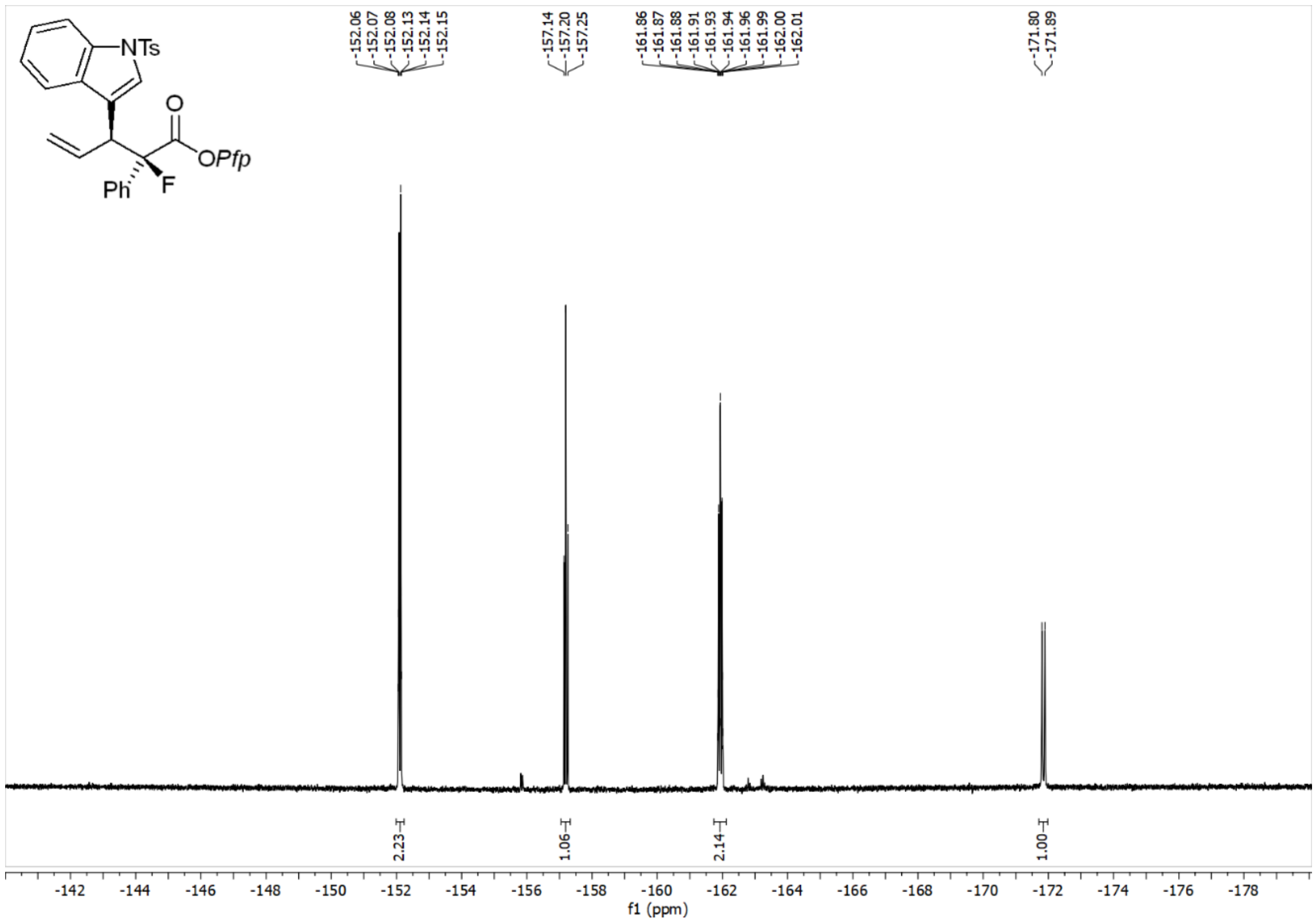
NTs
O
OPfp
Ph
F
-152.06
-152.07
-152.08
-152.13
-152.14
-152.15
-157.14
-157.20
-157.25
-161.86
-161.87
-161.88
-161.91
-161.93
-161.94
-161.96
-161.99
-162.00
-162.01
-171.80
-171.89
2.23
1.06
2.14
1.00
-142
-144
-146
-148
-150
-152
-154
-156
-158
-160
-162
-164
-166
-168
-170
-172
-174
-176
-178
f1 (ppm)

**(2*R*,3*R*)-2-fluoro-2,3-diphenylpent-4-enamide (*R*,*R*)-29:**

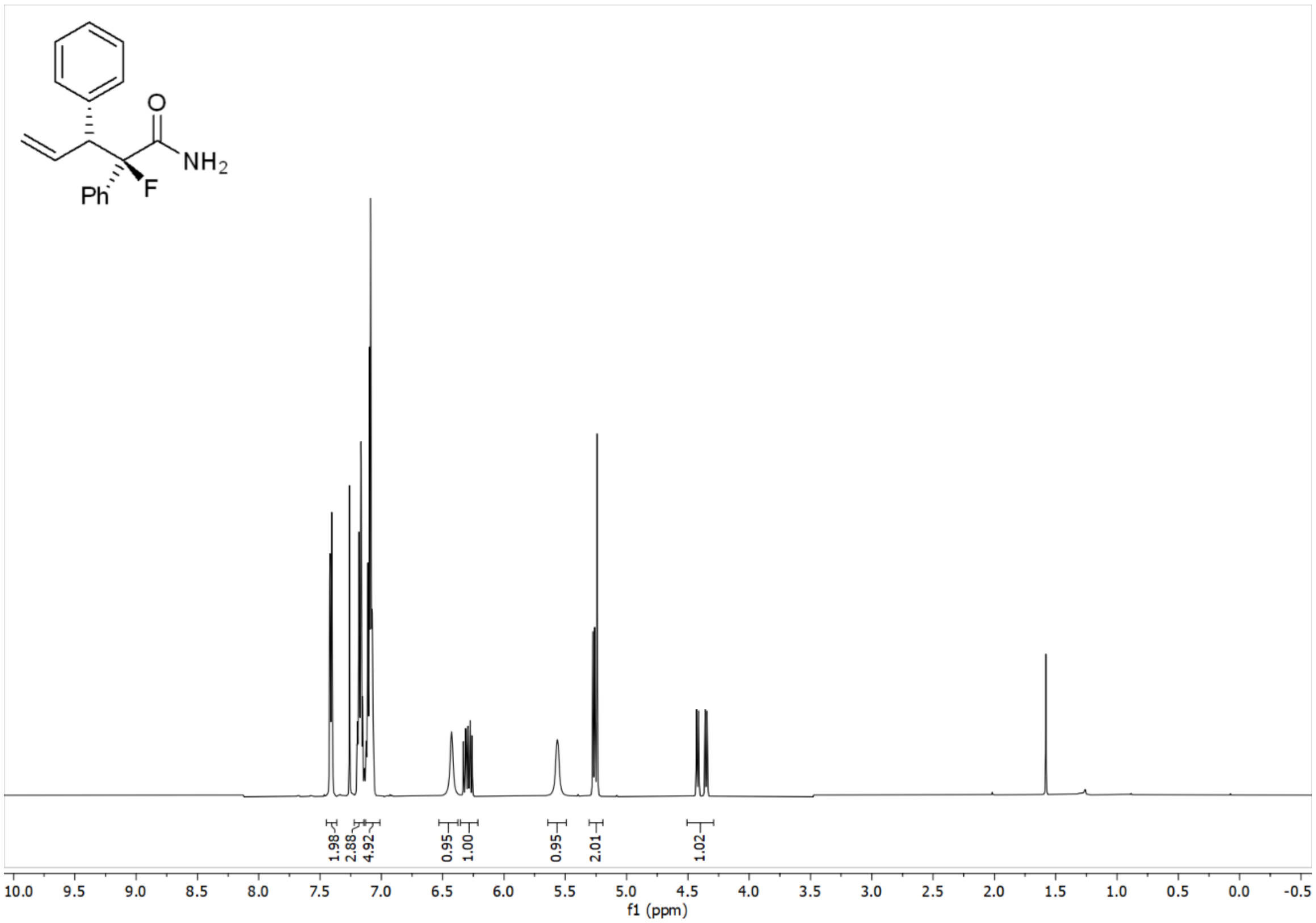

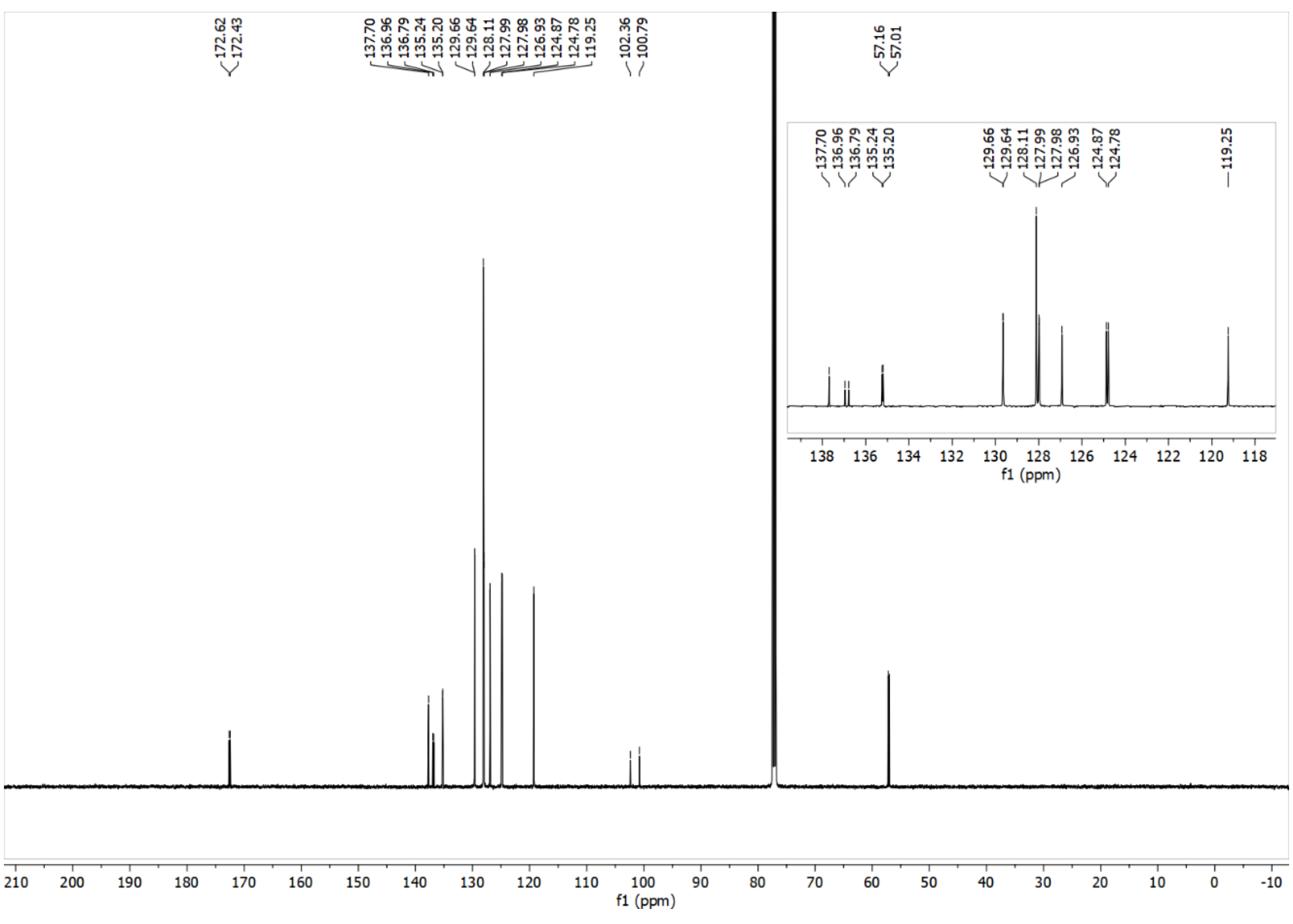

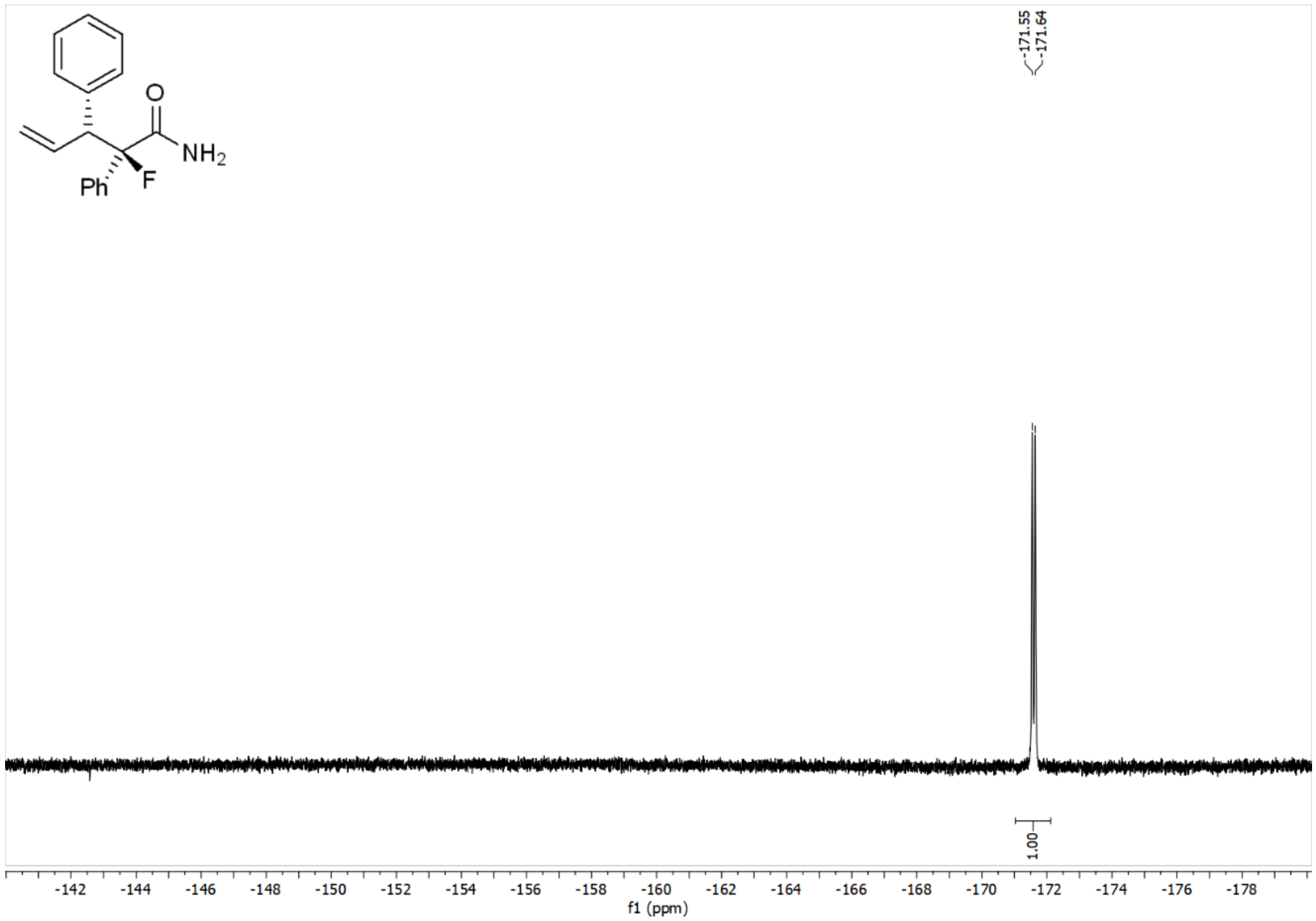
-171.55
-171.64
NH2
Ph
F
O
1.00
-142
-144
-146
-148
-150
-152
-154
-156
-158
-160
-162
-164
-166
-168
-170
-172
-174
-176
-178
f1 (ppm)

**(2*R*,3*S*)-2-fluoro-2,3-diphenylpent-4-enamide (*R*,*S*)-29:**

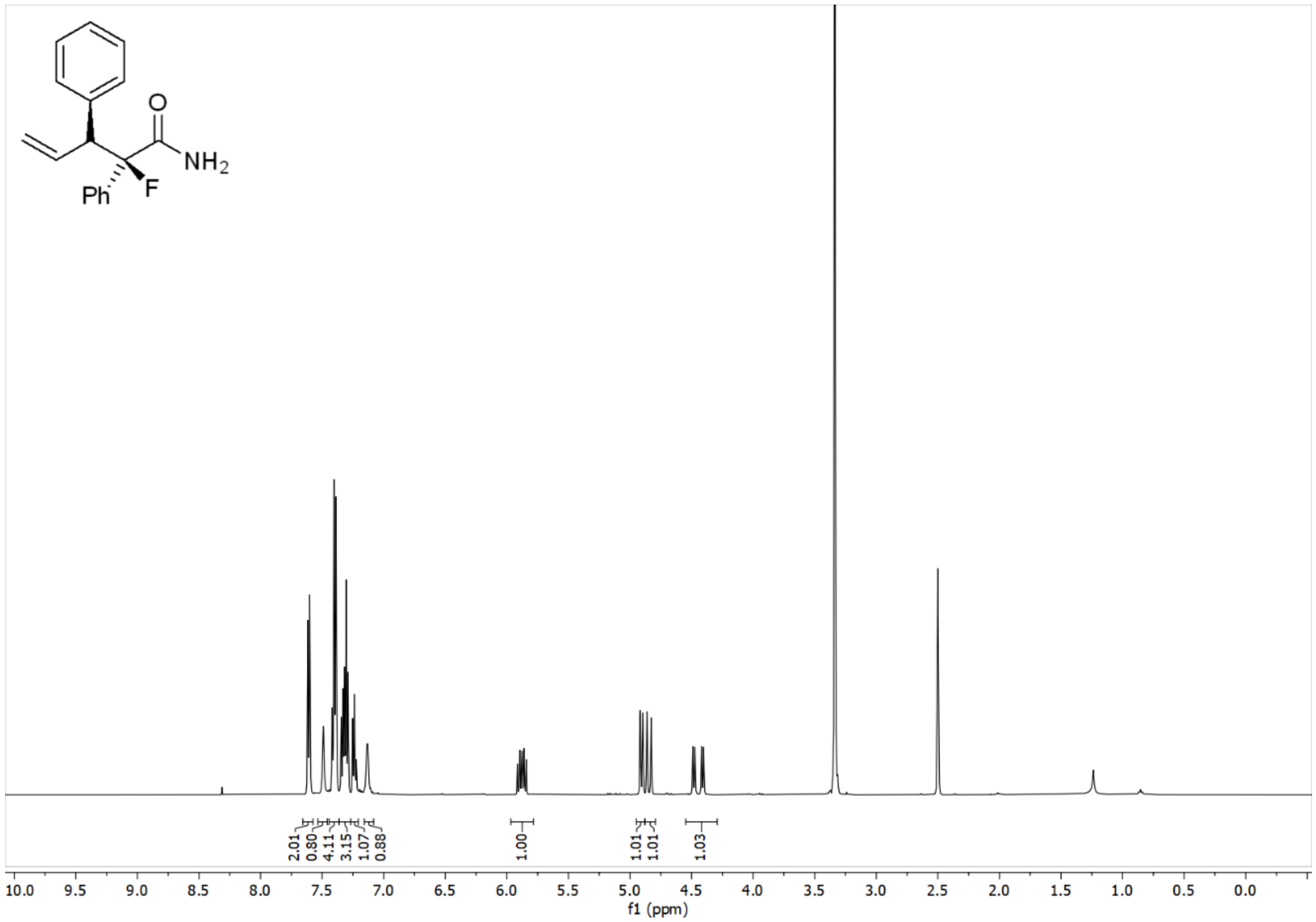


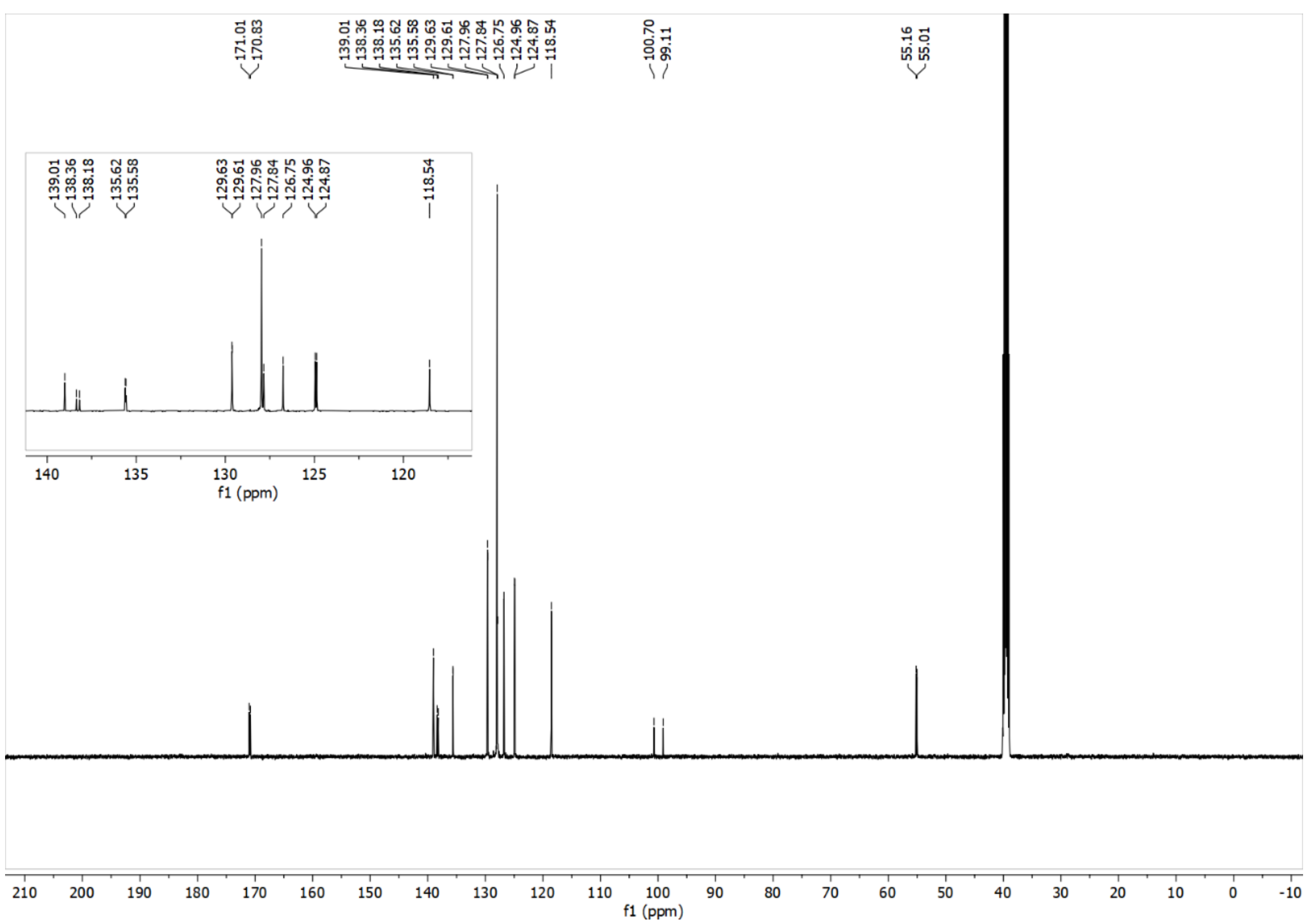

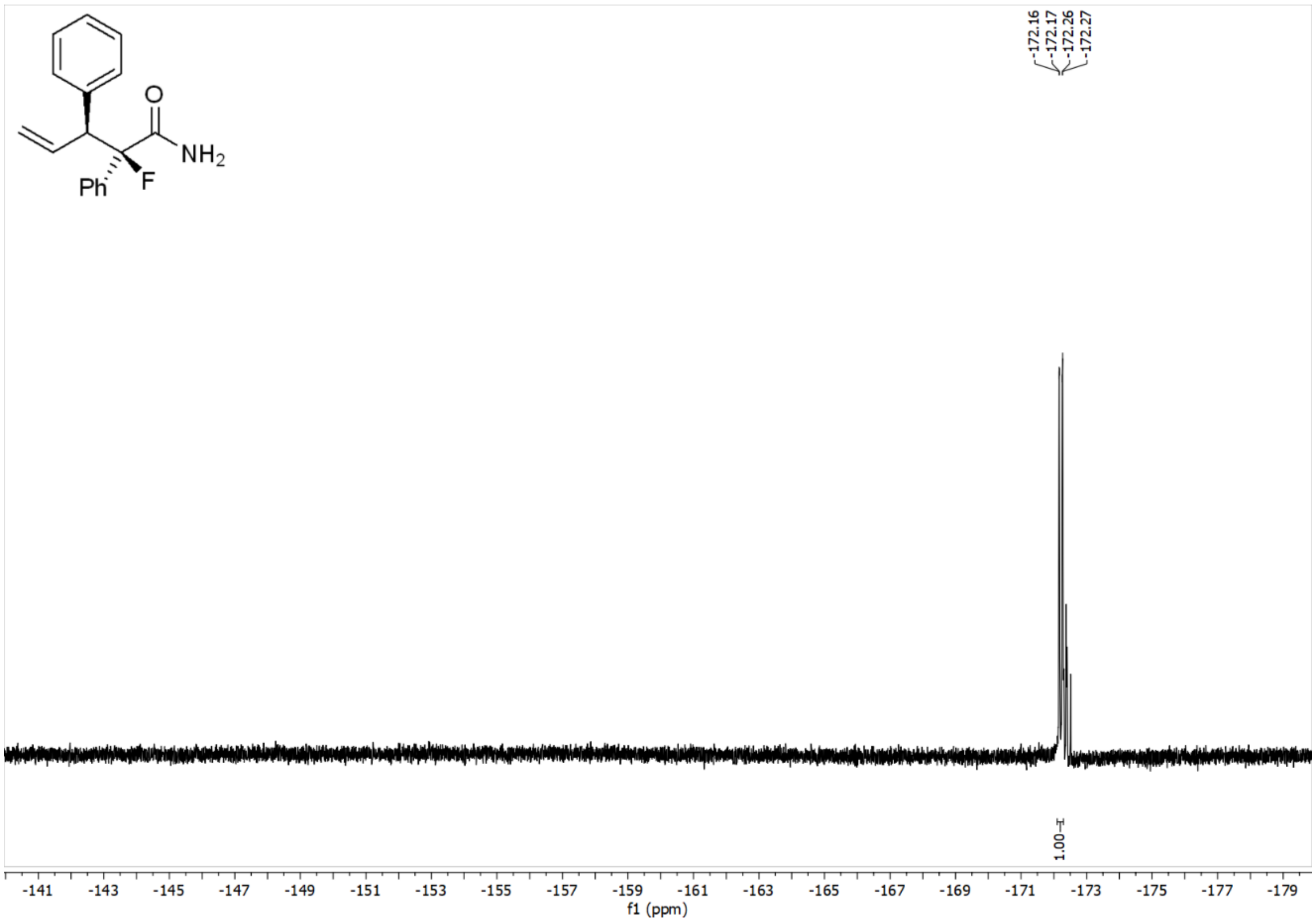

NH2
Ph
F
-172.16
-172.17
-172.26
-172.27
1.00
-141
-143
-145
-147
-149
-151
-153
-155
-157
-159
-161
-163
-165
-167
-169
-171
-173
-175
-177
-179
f1 (ppm)

**Methyl (2*R*,3*R*)-2-fluoro-2,3-diphenylpent-4-enoate (*R*,*R*)-28:**

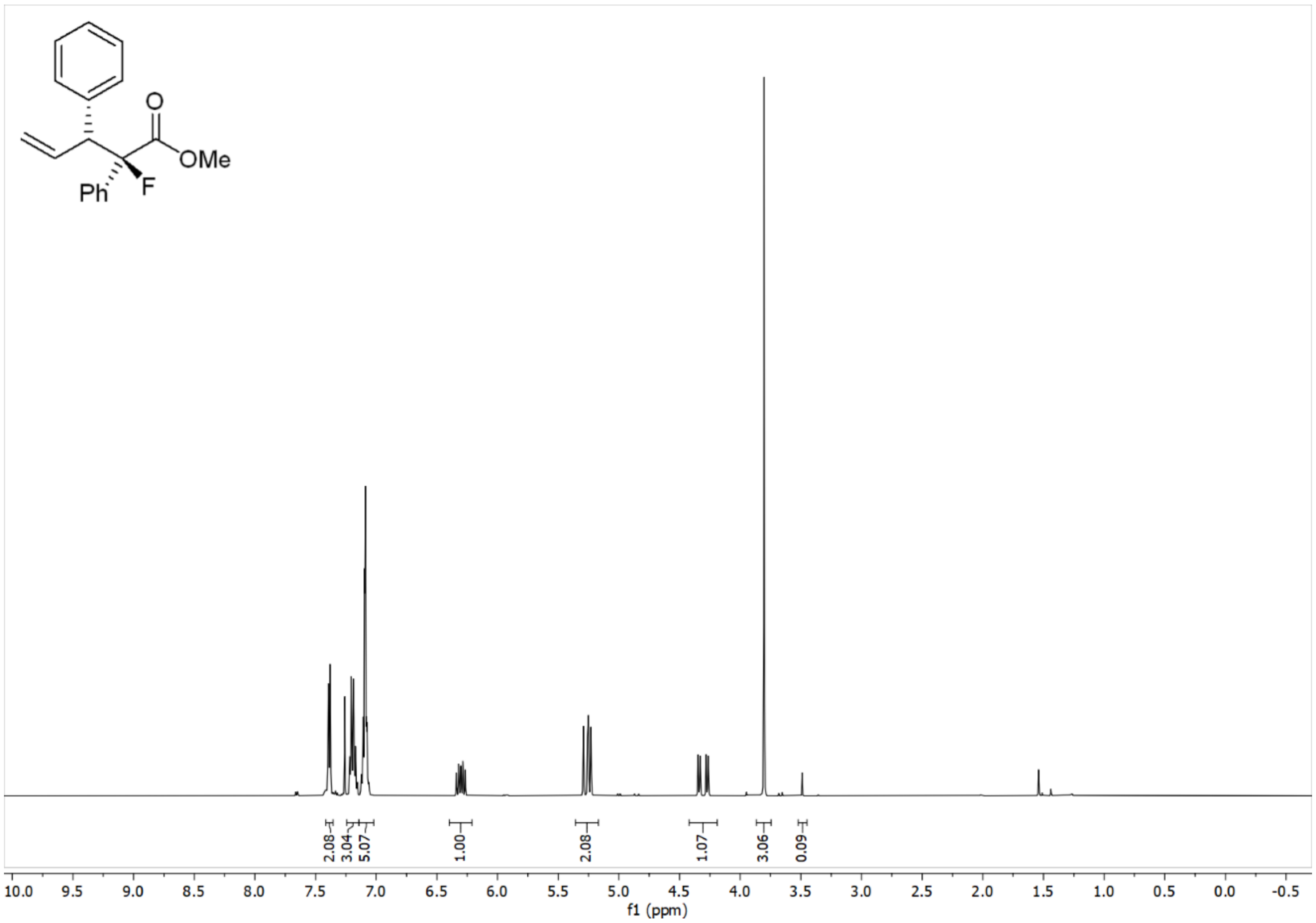

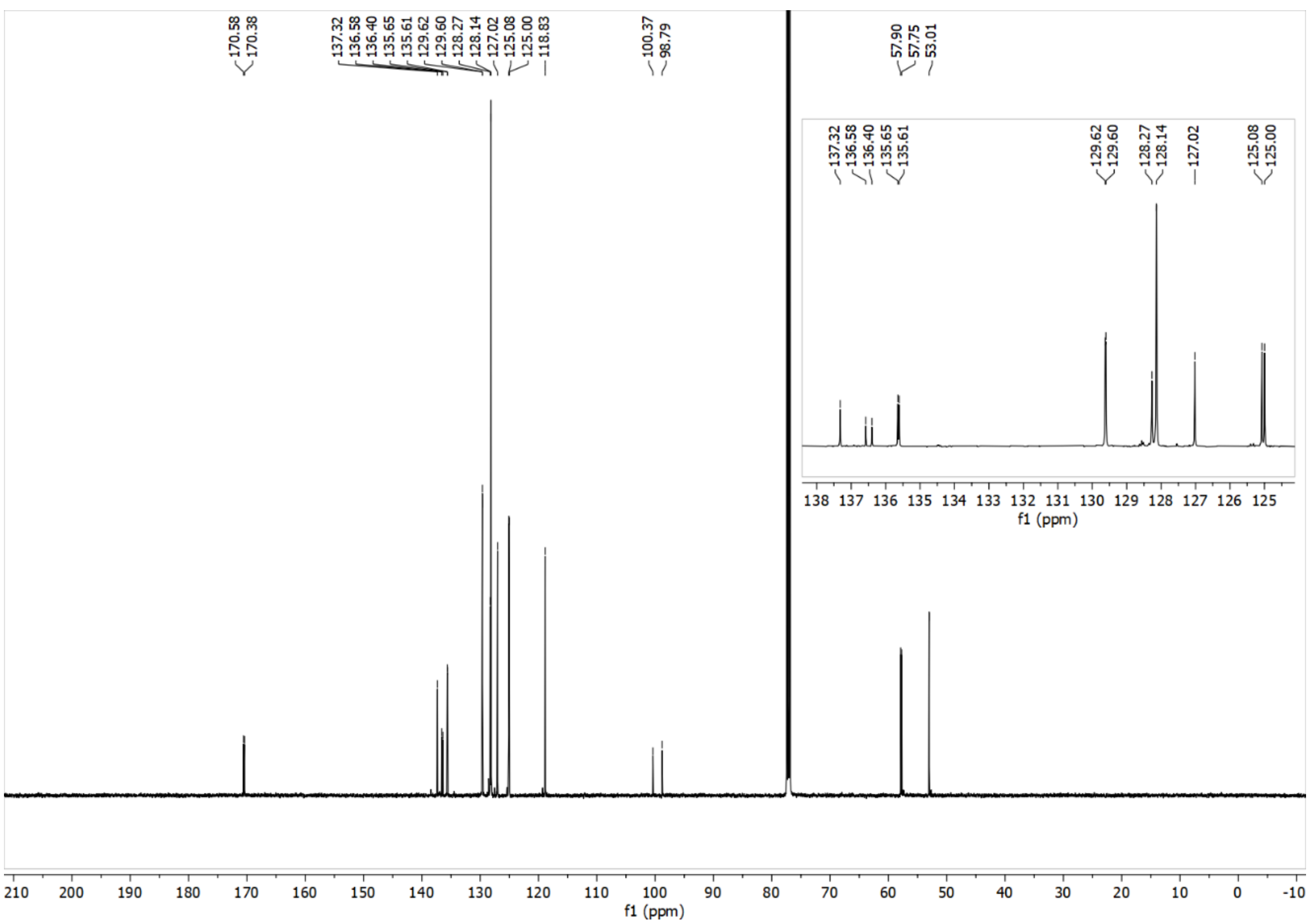

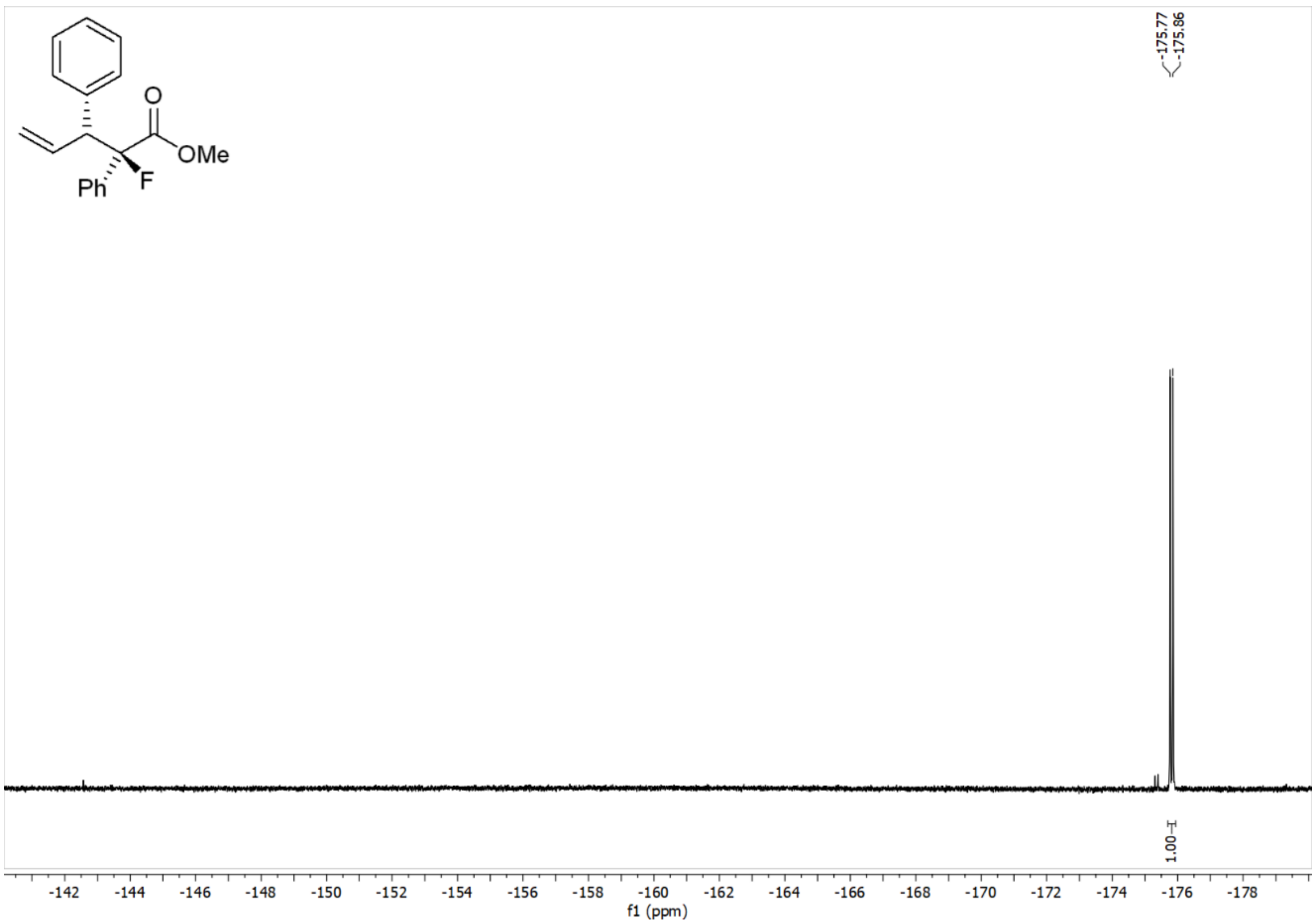
O
OMe
Ph
F
-175.77
-175.86
1.00
-142
-144
-146
-148
-150
-152
-154
-156
-158
-160
-162
-164
-166
-168
-170
-172
-174
-176
-178
f1 (ppm)

**Methyl (2*R*,3*S*)-2-fluoro-2,3-diphenylpent-4-enoate (*R*,*S*)-28:**

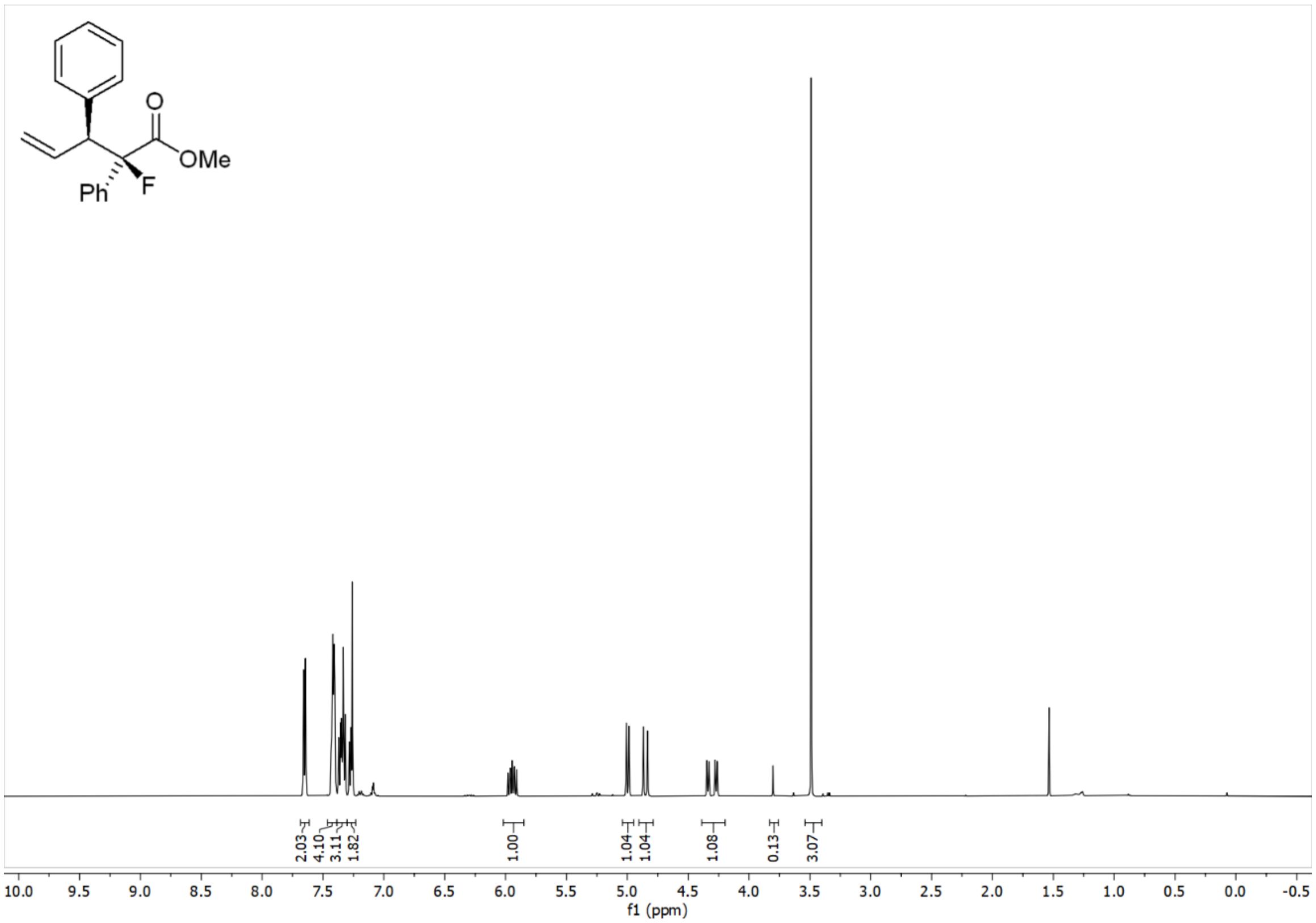


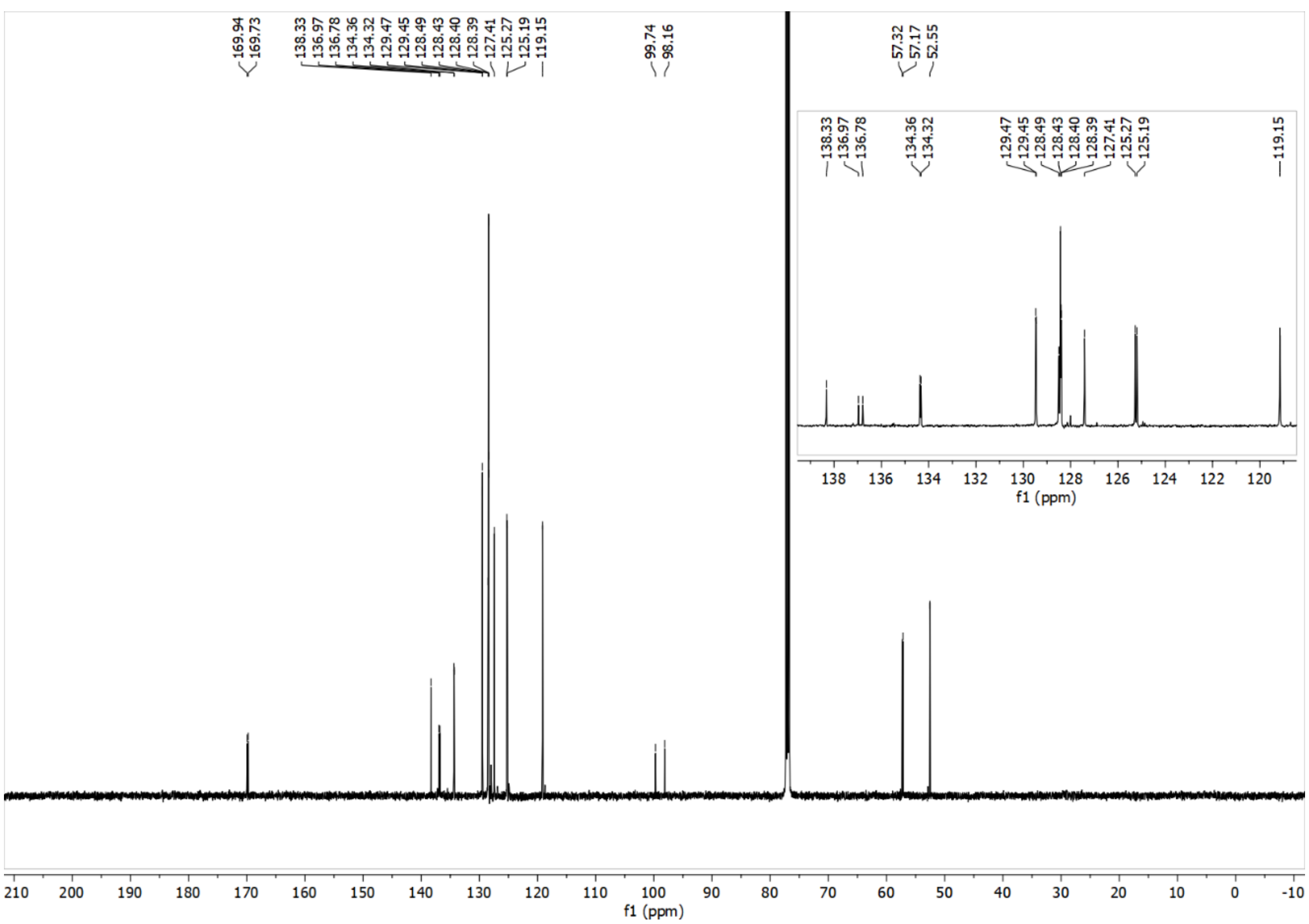

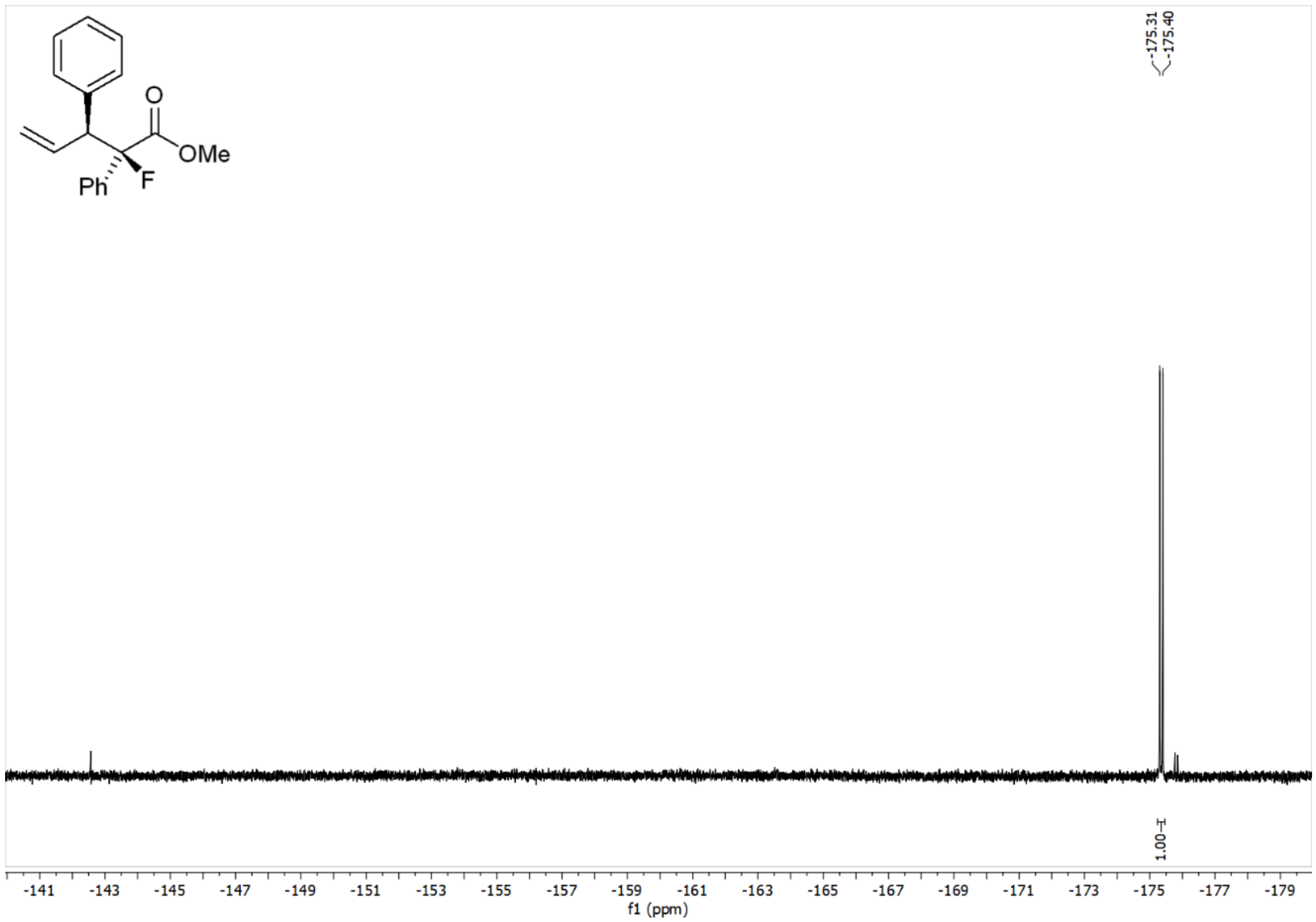
-175.31
-175.40
1.00
-141
-143
-145
-147
-149
-151
-153
-155
-157
-159
-161
-163
-165
-167
-169
-171
-173
-175
-177
-179
f1 (ppm)
OMe
Ph
F
O

**(2*R*,3*R*)-2-fluoro-2,3-diphenylpent-4-en-1-ol (*R*,*R*)-27:**

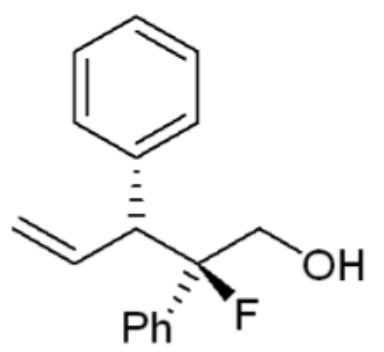


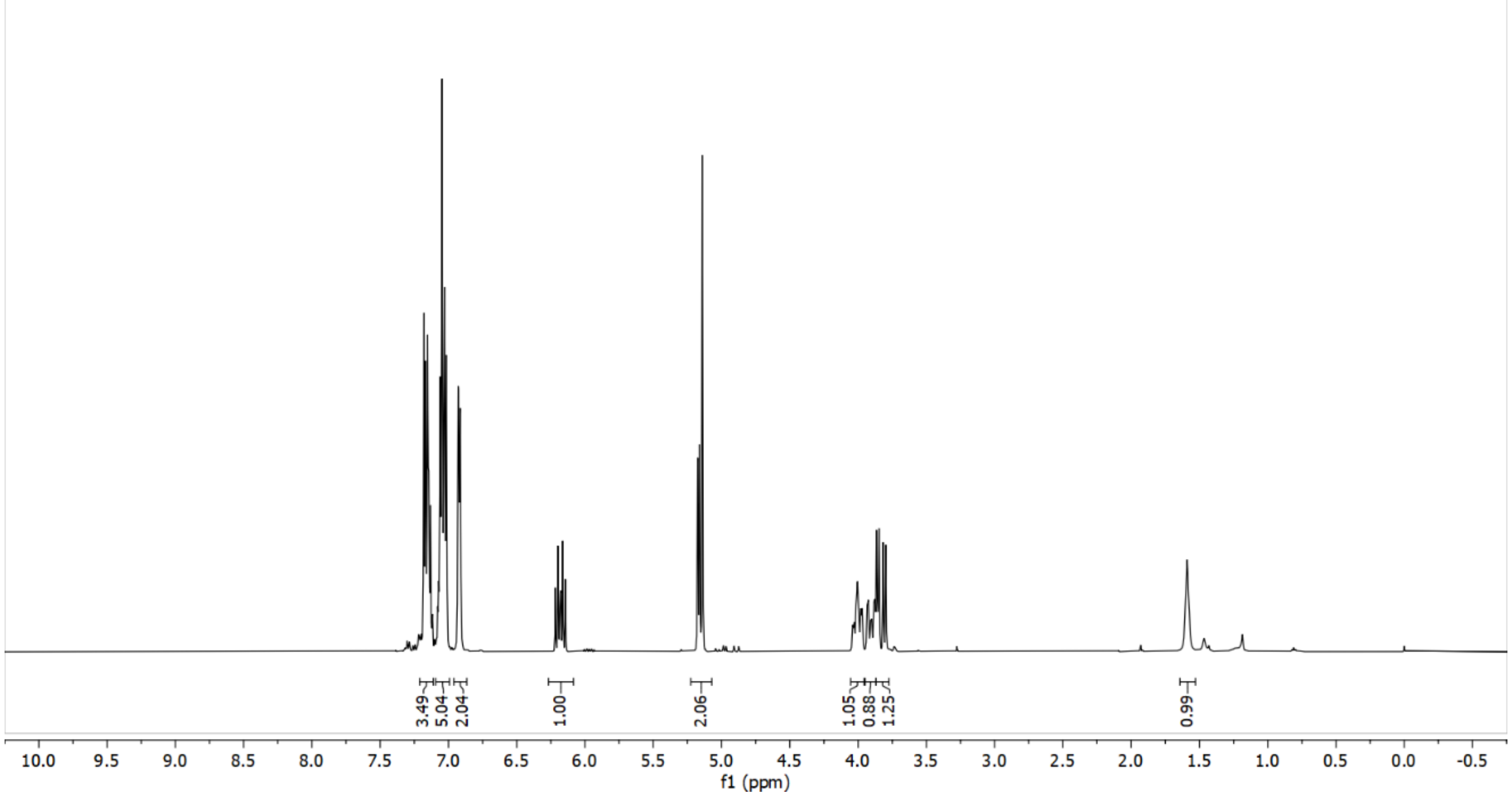


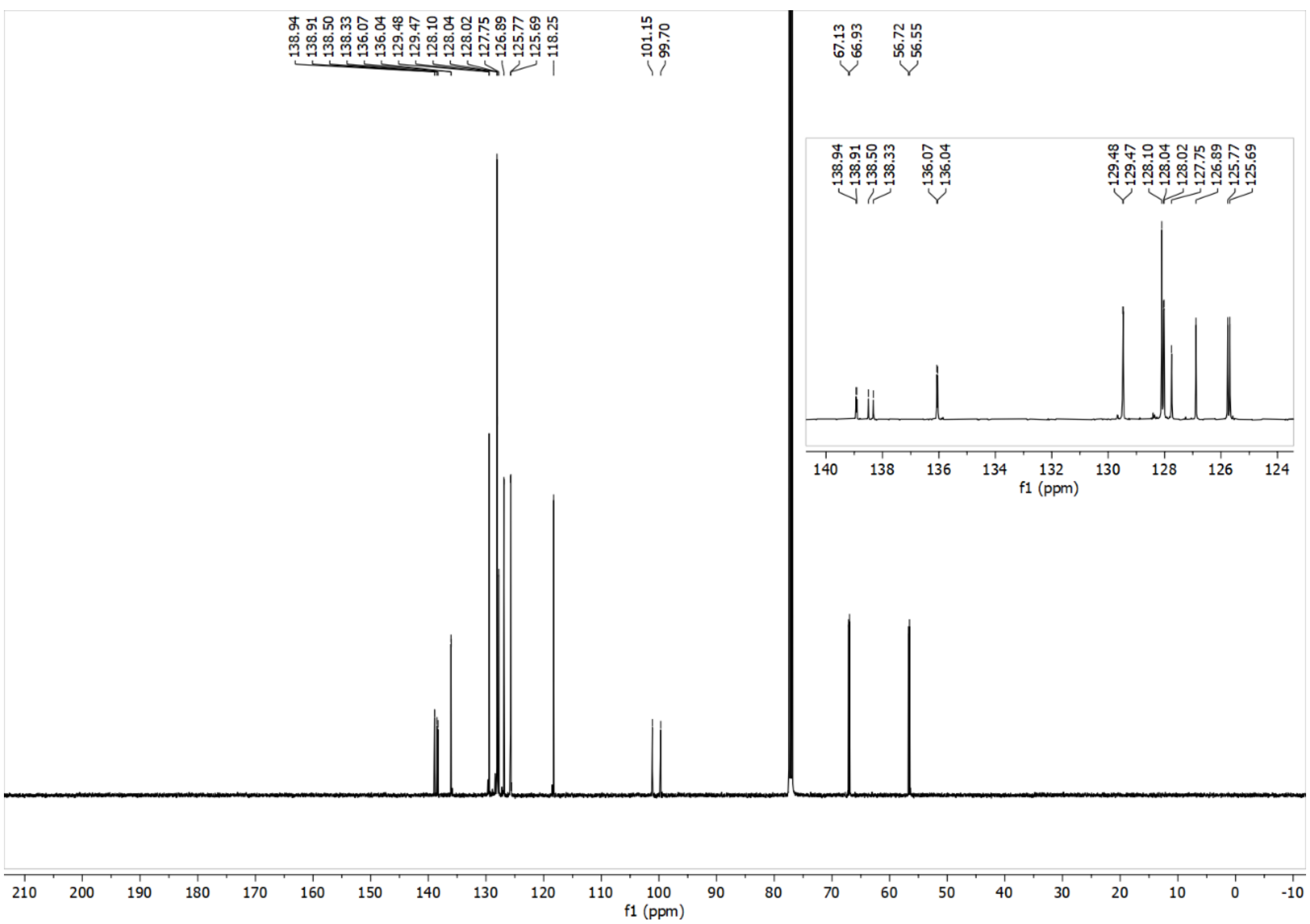

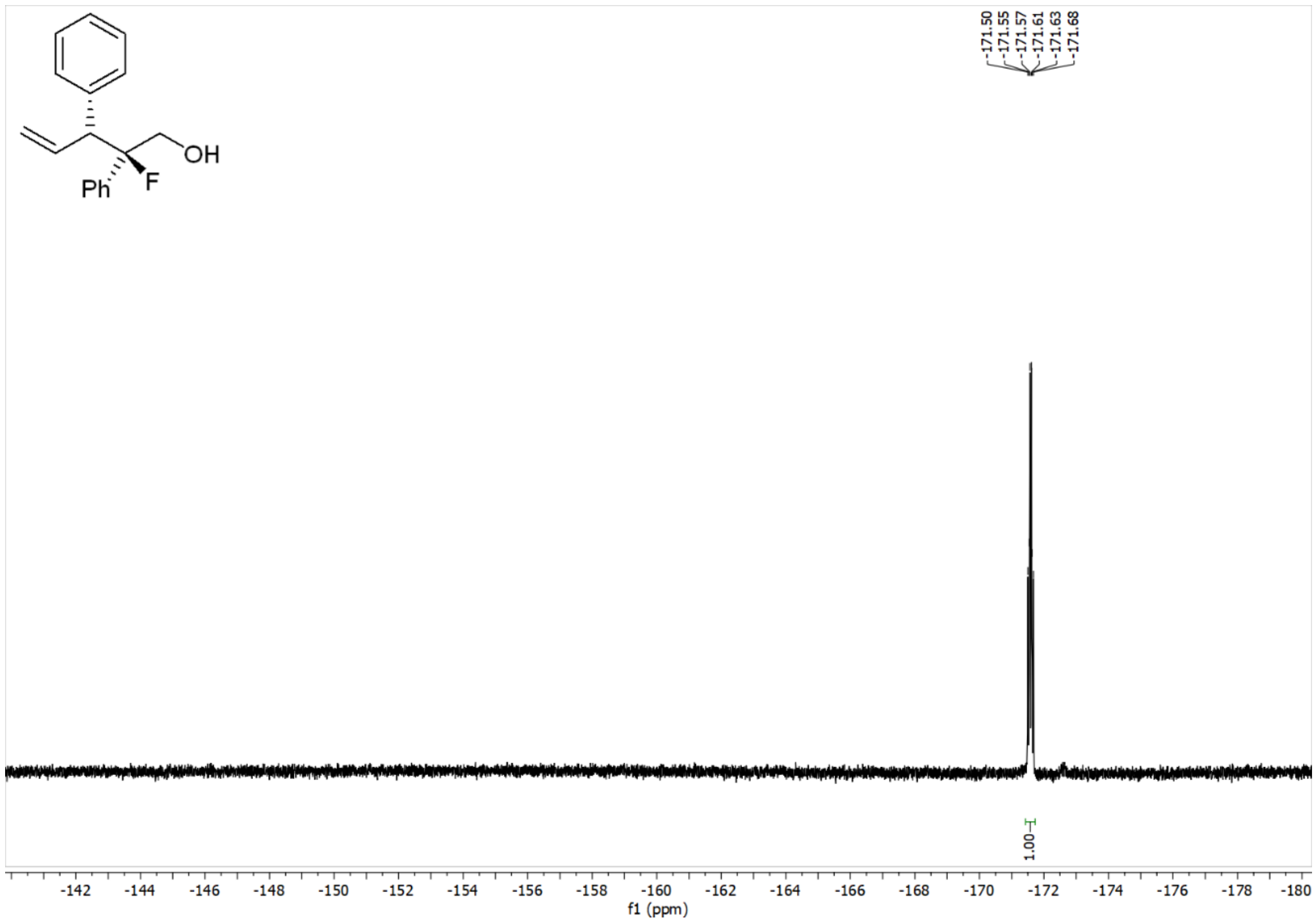

OH
Ph
F
-171.50
-171.55
-171.57
-171.61
-171.63
-171.68
1.00
-142
-144
-146
-148
-150
-152
-154
-156
-158
-160
-162
-164
-166
-168
-170
-172
-174
-176
-178
-180
f1 (ppm)

**(2*R*,3*S*)-2-fluoro-2,3-diphenylpent-4-en-1-ol (*R*,*S*)-27:**

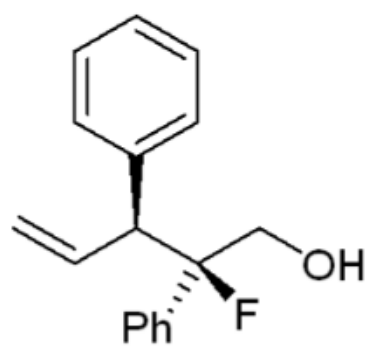


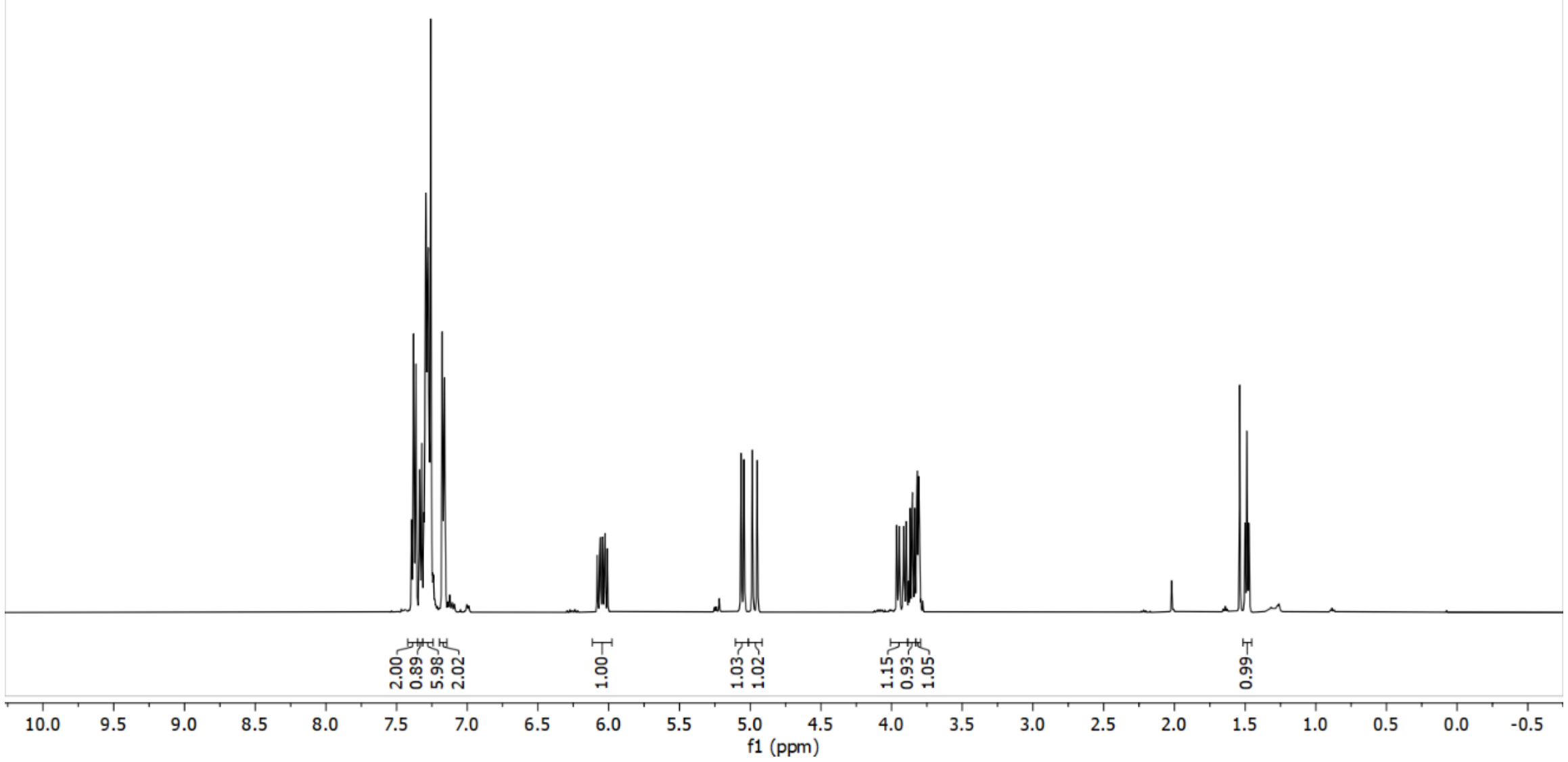


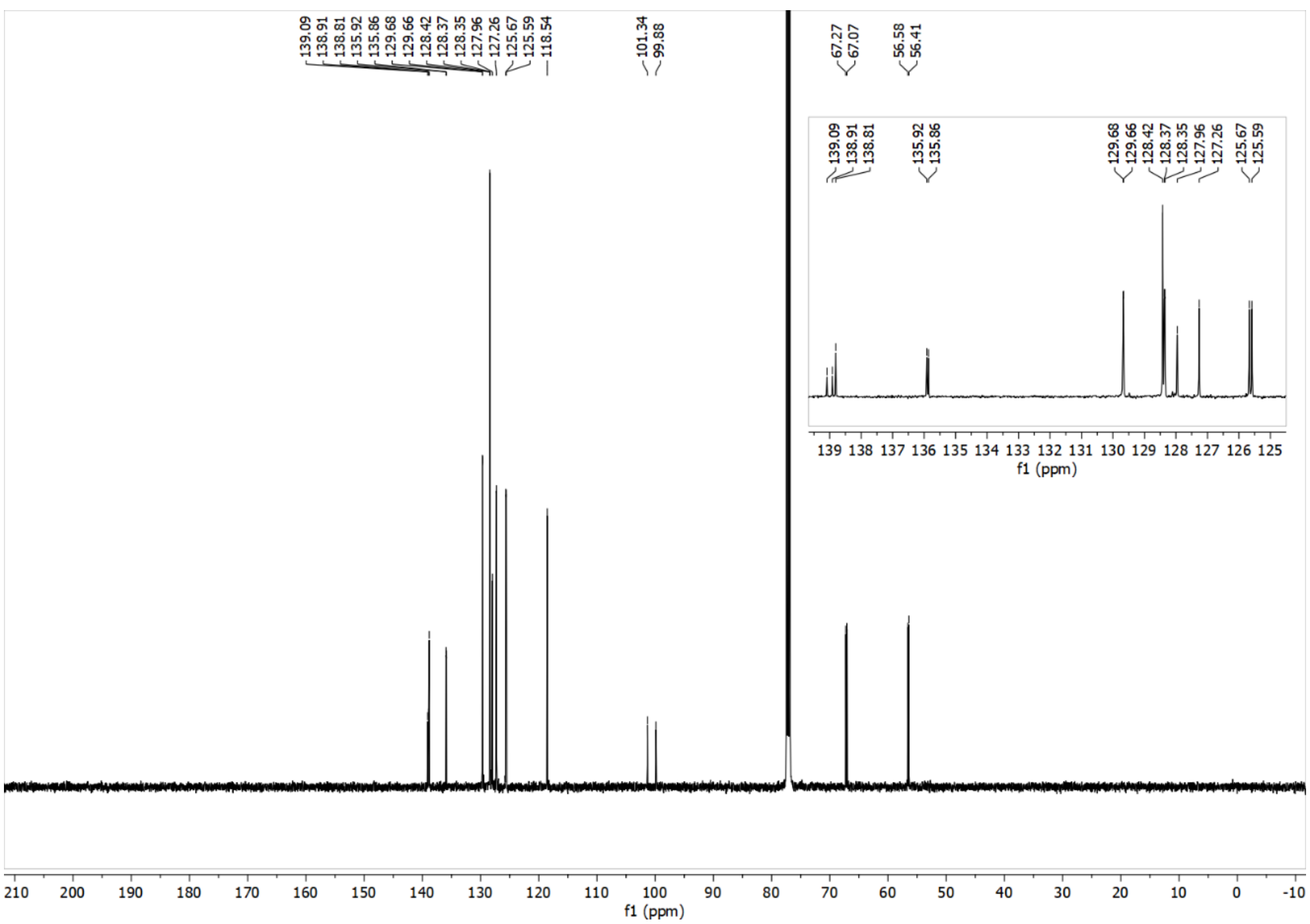

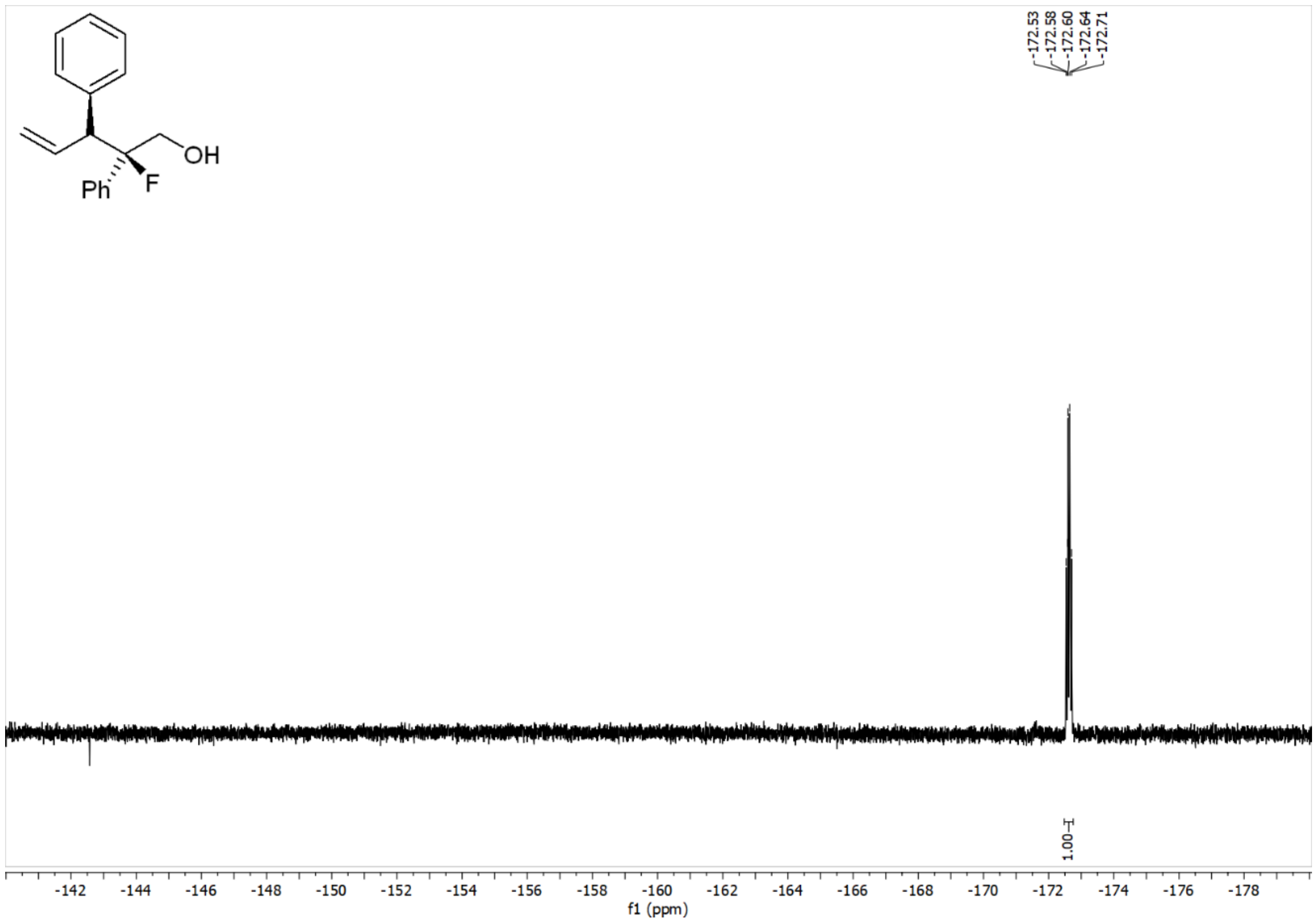
-172.53
-172.58
-172.60
-172.64
-172.71
OH
Ph
F
1.00
-142
-144
-146
-148
-150
-152
-154
-156
-158
-160
-162
-164
-166
-168
-170
-172
-174
-176
-178
f1 (ppm)

**(2*R*,3*R*)-N-allyl-2-fluoro-2,3-diphenylpent-4-enamide (*R*,*R*)-30:**

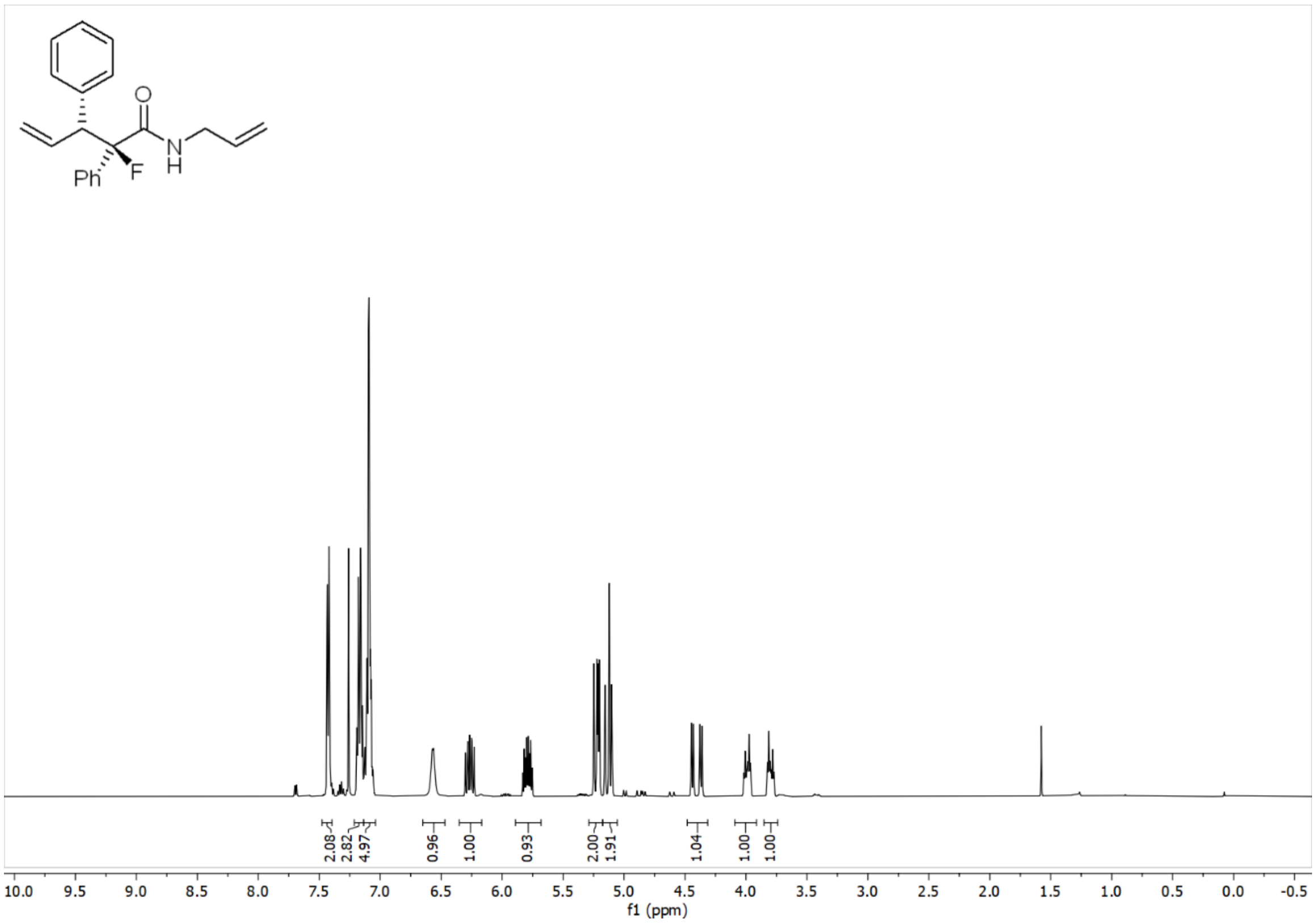


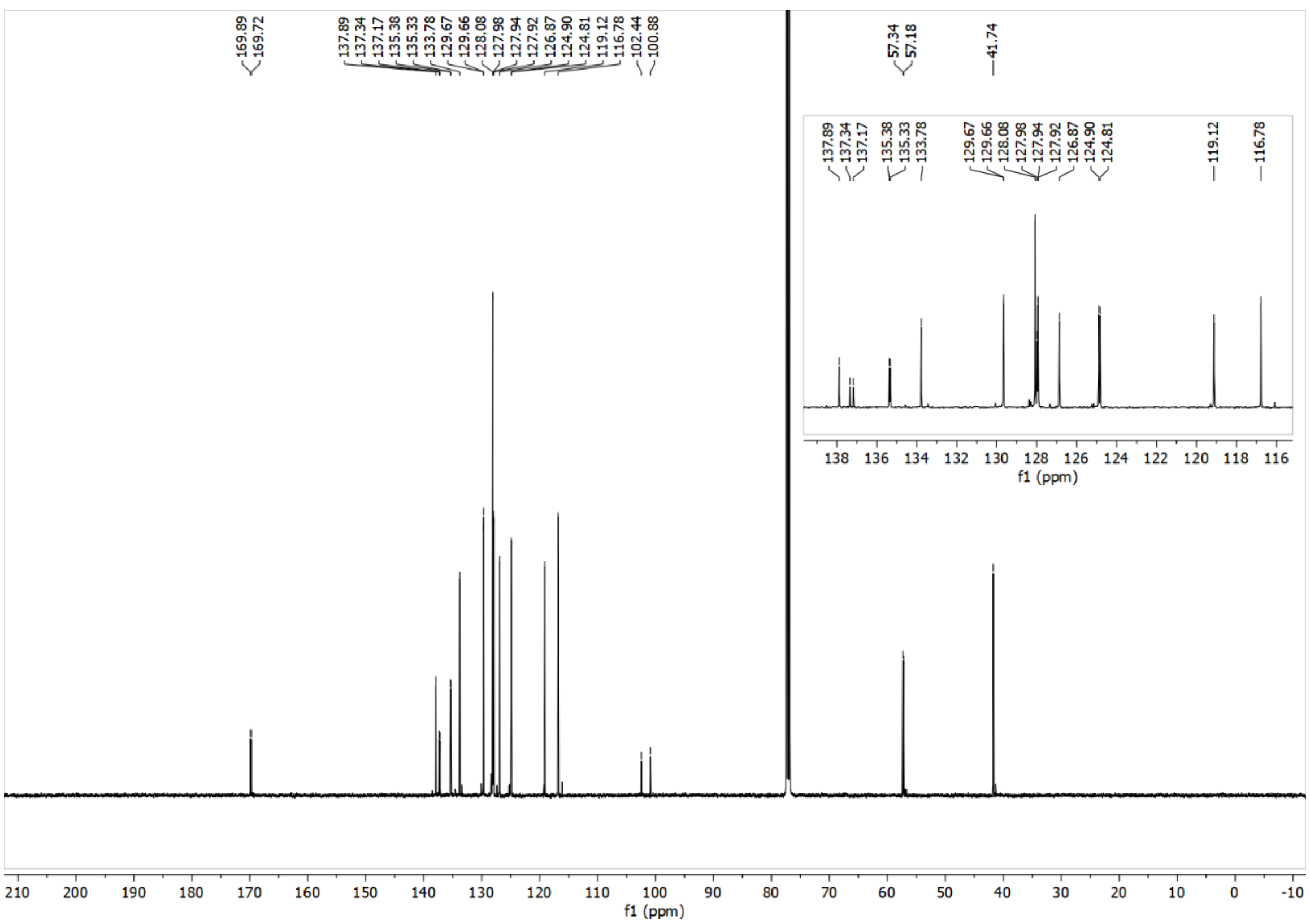

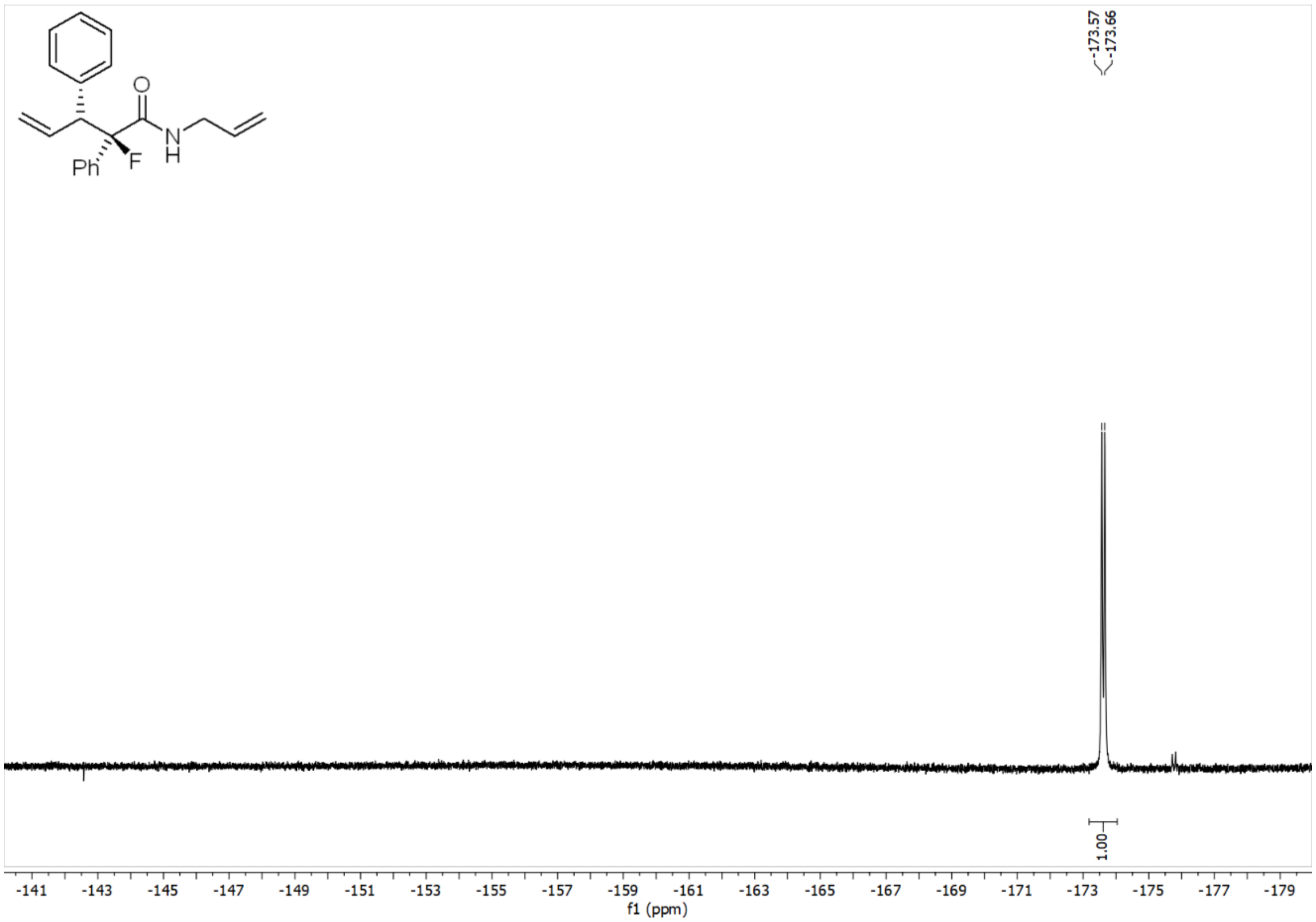

Ph
F
O
N
H
-173.57
-173.66
1.00
-141
-143
-145
-147
-149
-151
-153
-155
-157
-159
-161
-163
-165
-167
-169
-171
-173
-175
-177
-179
f1 (ppm)

**(2*R*,3*S*)-N-allyl-2-fluoro-2,3-diphenylpent-4-enamide (*R*,*S*)-30:**

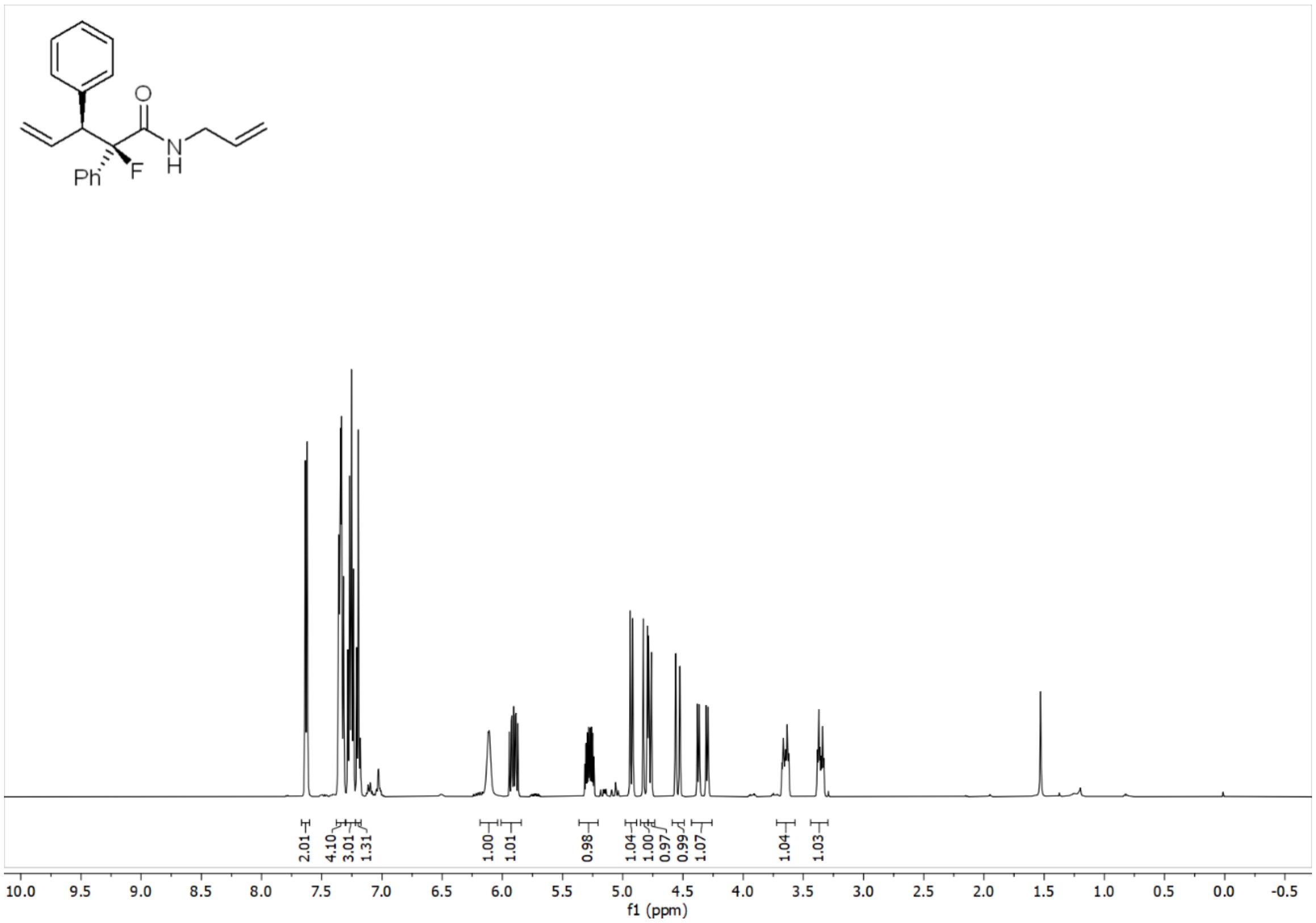


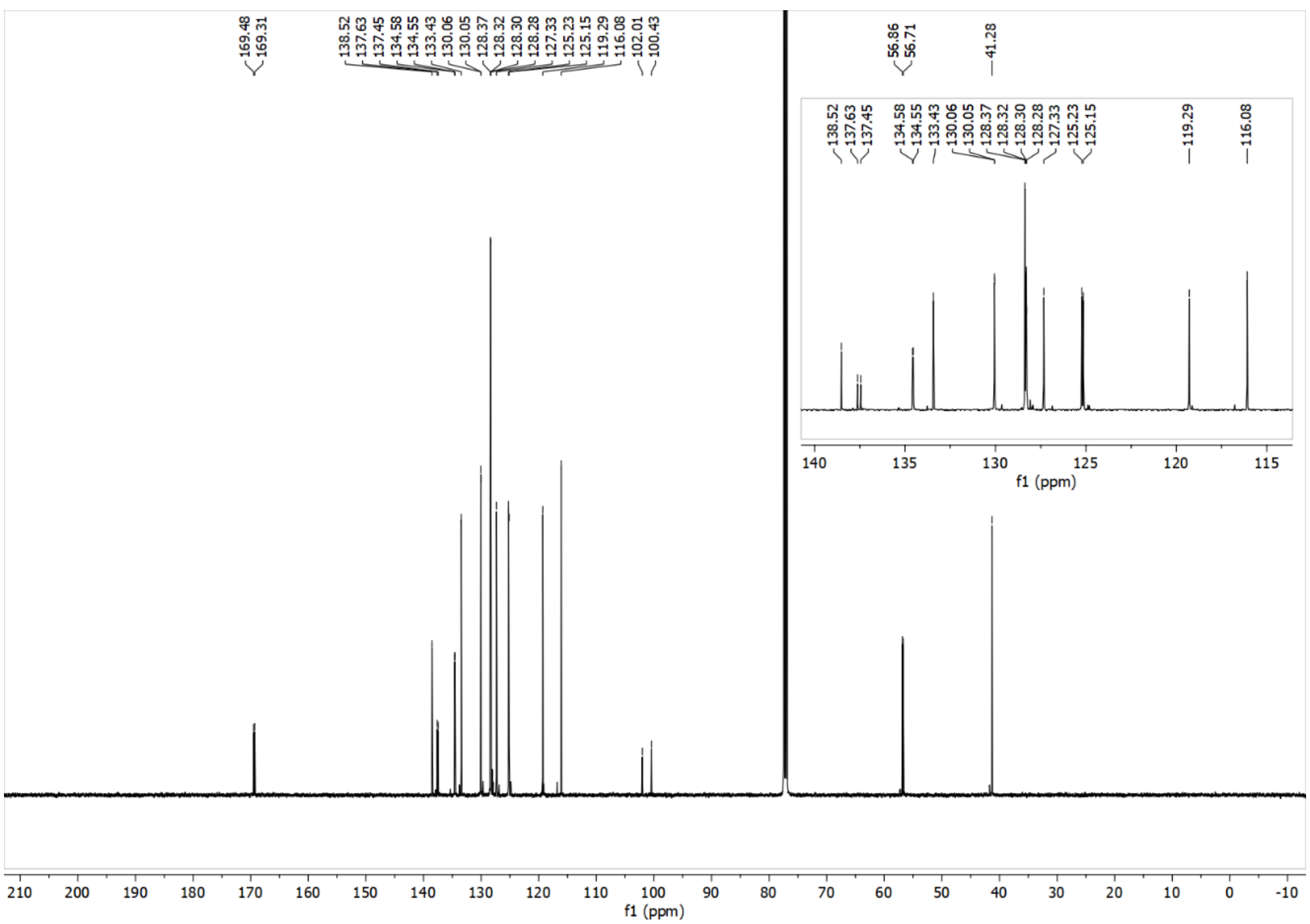

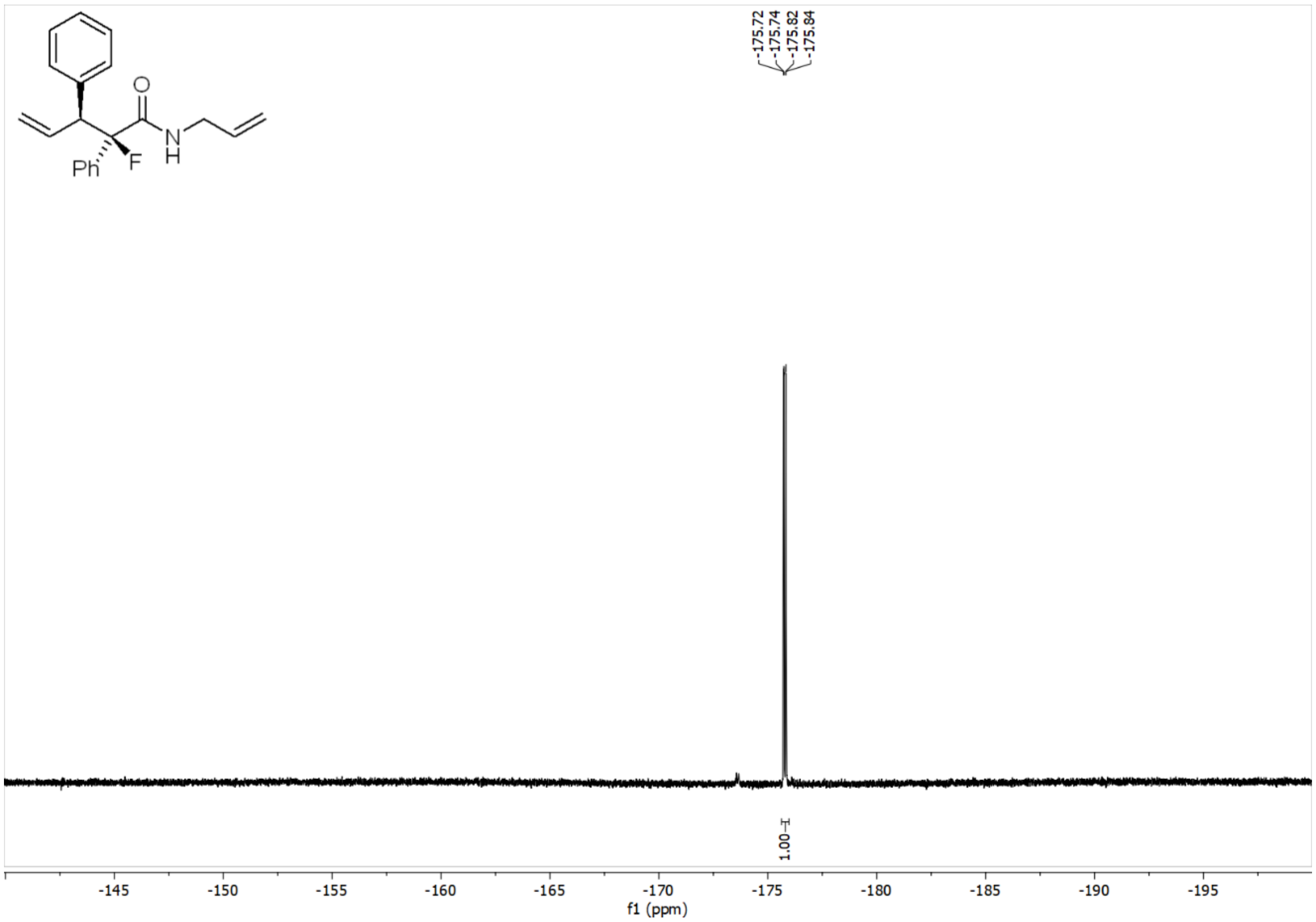

-175.72
-175.74
-175.82
-175.84
1.00
-145
-150
-155
-160
-165
-170
-175
-180
-185
-190
-195
f1 (ppm)
